# Synthesis of Six Natural Products Enabled by Selective Hydroxylation of Orcinaldehyde‐Like Substrates With an Enzyme Library

**DOI:** 10.1002/anie.4567477

**Published:** 2026-07-11

**Authors:** Gonzalo J. Villegas Rodríguez, Evan O. Romero, Jonathan C. Perkins, Diego N. Rojas, Maximilian S. Hrigora, Alison R. H. Narayan

**Affiliations:** ^1^ Department of Chemistry University of Michigan Ann Arbor Michigan USA; ^2^ Life Sciences Institute University of Michigan Ann Arbor Michigan USA; ^3^ Program in Chemical Biology University of Michigan Ann Arbor Michigan USA

**Keywords:** biocatalysis, bioinformatic tools, enzyme library, *o*‐quinone methides, total synthesis

## Abstract

Achieving selective oxygenation chemistry offers a powerful strategy for streamlining routes to complex target molecules, yet the development of site‐selective C–H functionalization reactions is challenging. Several enzyme families are capable of catalyzing C–H hydroxylation reactions and, in principle, provide an attractive solution for achieving selective oxidative transformations. In practice, however, the difficulty of identifying a biocatalyst suited to a specific synthetic target often renders chemoenzymatic strategies impractical. For example, biosynthetic enzymes such as CitB and ClaD can mediate site‐selective benzylic hydroxylation, enabling access to select natural products that share structural features with their native substrates. Nonetheless, such approaches typically lack generality and do not readily extend to broader collections of natural products. Here, we explore the previously unstudied natural sequence space surrounding biosynthetic hydroxylases CitB and ClaD to generate a bespoke panel of uncharacterized enzymes that collectively expand the substrate scope of biocatalytic benzylic hydroxylation reactions. Development of this curated biocatalyst panel enabled the hydroxylation of a set of orcinaldehyde‐like substrates, which were further elaborated to six natural products.

## Graphical abstract

Achieving selective (sp^3^)C–H hydroxylation can streamline the synthesis of complex organic molecules. Biocatalysis presents an opportunity to realize this transformation, however, identifying useful enzymes that enable synthetic strategies can be a challenge due to limitations in enzyme substrate scope. In this work, we explored the sequence space surrounding hydroxylases CitB and ClaD to broaden the substrate scope of a benzylic hydroxylation reaction, enabling the synthesis of six natural products.

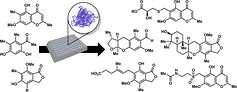

## 1 Introduction

Chemoenzymatic synthesis weaves together the best from chemical and biocatalytic methods to achieve the synthesis of a target molecule. This approach can benefit from the selectivity and efficiency possible through biocatalysis [1, 2]. However, biocatalysis is not historically the first option planned into a synthetic route, often due to the limited substrate scope associated with individual enzymes. This is a particular challenge as intermediates within synthetic routes are often not natural compounds that enzymes have evolved to accept [1, 3].

**FIGURE 1:**
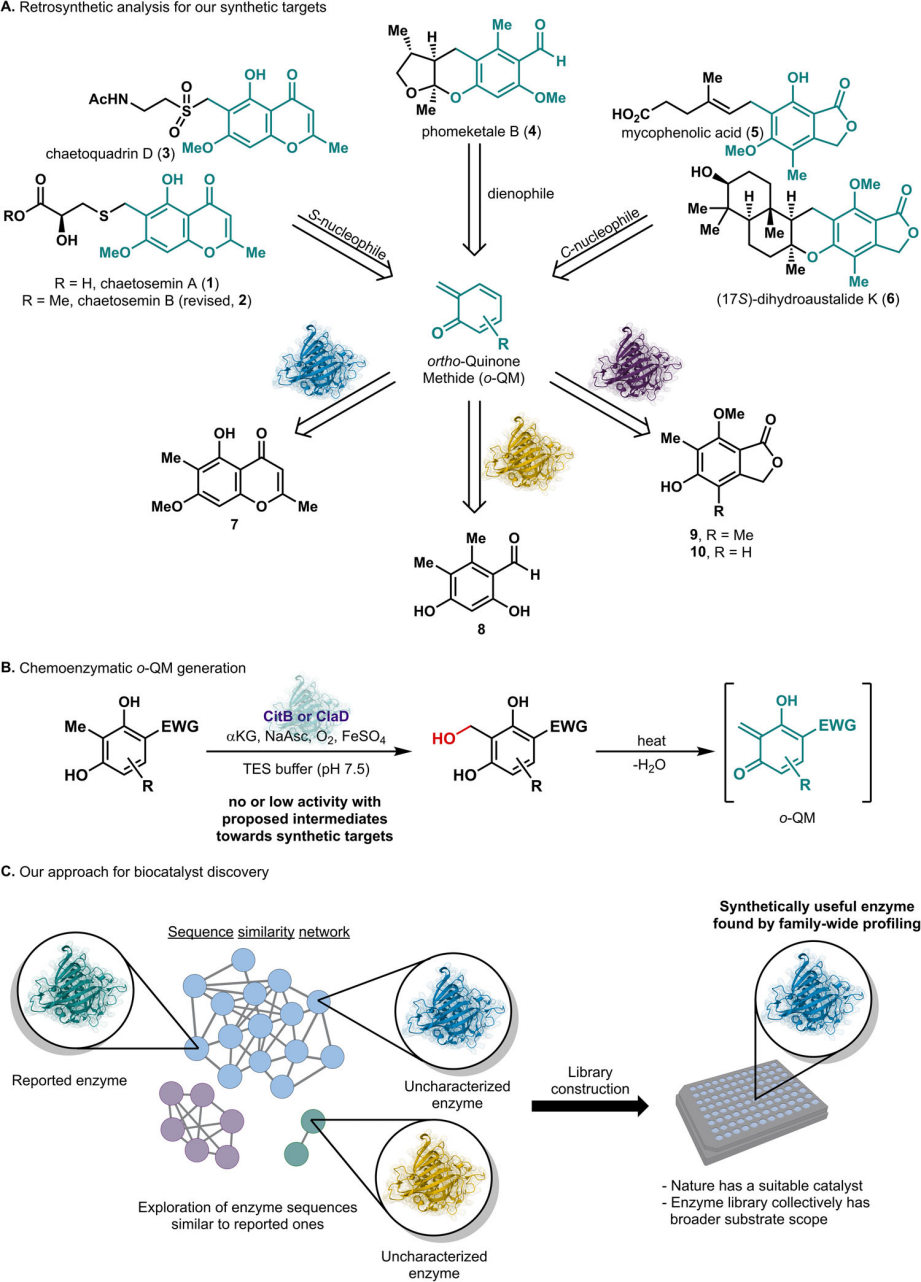
Overview of this work. (A). Synthetic targets in this work. Highlighted is the polyketide core of these molecules. Our synthetic strategy required trapping *o*‐QMs with either nucleophiles or dienophiles and further elaborating the adduct. We employed a chemoenzymatic *o*‐QM generation. (B). CitB and ClaD are previously reported enzymes that perform benzylic hydroxylation on orcinaldehyde‐like substrates [4, 5], subsequent non‐enzymatic dehydration produces *o*‐QMs [6]. (C). To discover the synthetically useful catalysts we profiled our substrates with a library of enzymes that was built based on a sequence similarity network (SSN). αKG: α‐ketoglutaric acid. NaAsc: Sodium ascorbate.

We recently encountered this challenge when relying on a biocatalytic benzylic hydroxylation as a key step toward a suite of natural products (**1–6**, Figure 1A). Specifically, we envisioned using two biosynthetic enzymes, CitB [4] and ClaD [5], to achieve a site‐selective benzylic hydroxylation of orcinaldehyde‐like substrates. These types of benzylic alcohol products are setup to form reactive *ortho*‐quinone methide (*o*‐QM) intermediates upon loss of water (Figure 1B) [4, 5, 6]. The careful matching of *o*‐QM precursor with nucleophile or cycloaddition reaction partners has the potential to afford access to a panel of natural products, such as antifungal agents chaetosemin A (**1**) and chaetosemin B (**2**) [7], chaetoquadrin D (**3**) [8], phomeketale B (**4**) [9], immunosuppressant mycophenolic acid (**5**) [10], and (17*S*)‐dihydroaustalide K (**6**) [11, 12, 13]. Only two out of these six targets have been previously synthesized, **5** and **6**. Specifically, **5** has been accessed through a number of strategies generally involving key Diels‐Alder and Stille reactions [10]. For (*17S*)‐dihydroaustalide K (**6**), a racemic synthesis was accomplished by Barret and coworkers using a biomimetic polyketide aromatization to build the core of the molecule [14]. We anticipated that a chemoenzymatic approach with an α‐ketoglutarate and iron dependent oxygenase such as CitB or ClaD could provide a new avenue toward these molecules with distinct advantages. In their native biosynthetic pathways these enzymes hydroxylate orcinaldehyde‐like substrates to produce benzylic alcohols (Figure 1B) [6] and have been used in the chemoenzymatic syntheses of xyloketal D [6] and xyloketal B [15]. However, they showed low to no activity with substrates **7–10**, hampering the synthesis of natural products **1–6** (Figure 1A).

To overcome this challenge, we considered profiling natural sequences related to CitB and ClaD, a strategy, that is, proven for broadening the substrate scope beyond that of known biosynthetic enzymes. This approach uses accessible bioinformatic tools to canvas sequence space to identify uncharacterized enzymes that are similar in sequence to an enzyme with known function [16, 17]. Building libraries in this manner can lead the discovery of new biocatalysts with synthetic utility. For instance, by using previously uncharacterized P450 enzymes from thermophilic organisms, the Arnold group found better starting points for directed evolution [17]. Further, profiling natural sequence space has successfully broadened the substrate scope of transformations with cytochromes P450 [18] and flavin‐dependent enzymes [16, 19, 20].

Herein, a sequence profiling approach is used to build a bespoke library of benzylic hydroxylases. These efforts ultimately led to the identification of a suite of enzymes capable of site‐selective benzylic hydroxylation of orcinaldehyde‐like substrates, which in turn enable the synthesis of six fungal natural products (**1–6**) without the need for protein engineering.

## 2 Results and Discussion

### 2.1 Library Construction

Among *o‐*QM generation methods [21], we were interested in the chemoenzymatic approaches for the potential to avoid protecting groups and achieve site‐ and chemoselectivity [22]. There are a number of enzymes that are involved in the synthesis of *o*‐QMs. For example, in the biosynthesis of cannabinoids, berberine bridge enzyme‐like oxidases form *o*‐QMs by accepting a hydride from the substrate [22]. Alternatively, stipitaldehyde is advanced toward neosetophomone B by a short chain dehydrogenase (EupfE) in a tropolonic *o‐*QM formation [23]. In another example, the production of leporin C proceeds through *o*‐QM formation, enabled by a *S*‐adenosyl‐L‐methionine (SAM)‐dependent enzyme (LepI), which dehydrates a benzylic alcohol to generate an *o‐*QM [24]. Finally, CitB [4] and ClaD [5], which are involved in the biosynthesis of fungal metabolites citrinin, peniphenones, and penilactones, are α‐ketoglutarate and iron dependent oxygenases that perform C(sp^3^)–H hydroxylation to generate benzylic alcohols, which lead to *o‐*QMs after non‐enzymatic dehydration.

**FIGURE 2:**
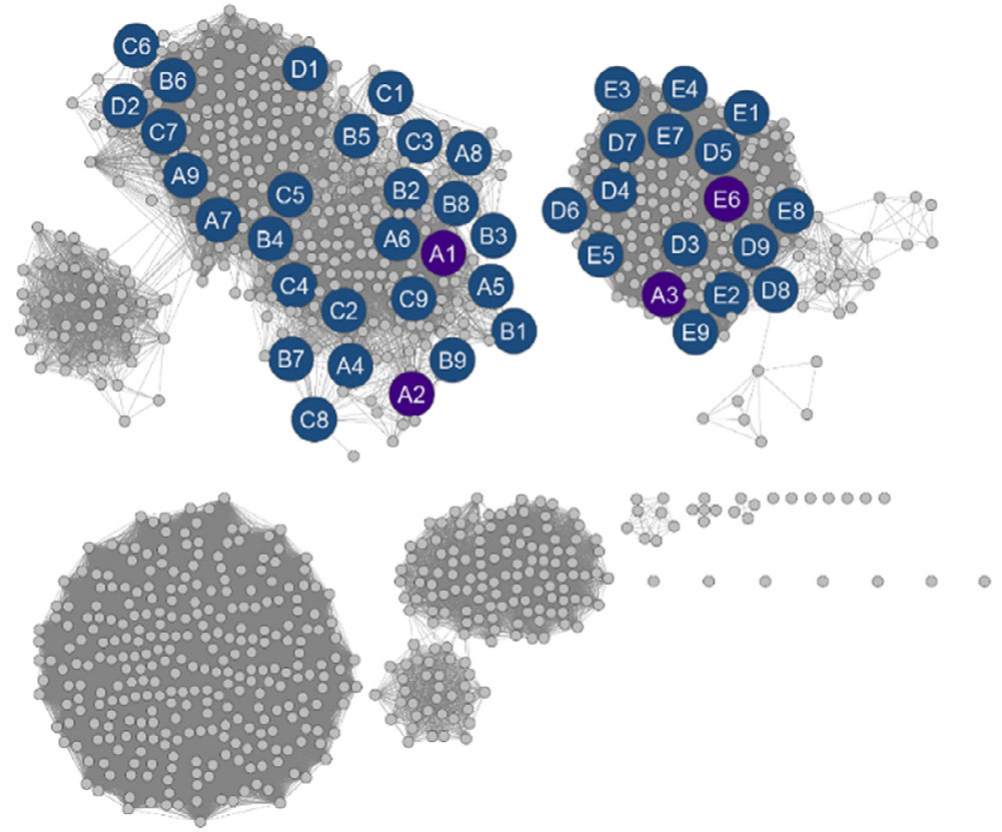
SSN built based on the sequence of CitB. Enzymes for library construction were selected from two clusters. Blue circles represent sequences of uncharacterized enzymes in our enzyme library and purple circles represent sequences of characterized enzymes in our enzyme library: CitB (A1), ClaD (A2), TropC (A3), and XenC (E6).

Although CitB and ClaD were not sufficient catalysts to enable the synthesis of targeted natural products **1–6**, the structural similarity between their natural substrates (Figures 1B and S2) [4, 5] and the targeted substrates **7–10** (Figure 1A) led us to build an enzyme library based on CitB and ClaD. To select enzymes for the library, a sequence similarity network (SSN) was used to visualize the relationships between protein sequences most closely related to CitB and ClaD. SSNs have been demonstrated as a useful tool for showing trends in sequence‐function relationships and are a user‐friendly bioinformatic tool [16, 25, 26]. The reported amino acid sequence of CitB (UniProt accession number: A0A159BP93) [4] was queried into bioinformatic tools BLAST [25] and EFI‐EST [26] to generate a SSN (Figure 2). Only four enzymes in the resulting SSN had been previously characterized: CitB [4] and ClaD [5], which are known for benzylic hydroxylation, as well as TropC [27] and XenC [28], which mediate an oxidative ring‐expansion within biosynthetic pathways (purple circles, Figure 2). From the constructed SSN, 41 uncharacterized proteins and four characterized enzymes were selected to build a library (Figure 2). Each protein was assigned a name based on the position in a well‐plate (rows A–E and columns **1–9**). The enzymes in the library share an average sequence identity of 46%; with the lowest sequence identity being 32%, and the highest 98%. Specifically, CitB and ClaD share a sequence identity of 55%. To access each enzyme, *E. coli* BL21(DE3) cells were transformed with recombinant plasmids encoding each of the 45 enzyme sequences. The resulting strains were used to produce protein in 96‐well plate format. Additionally, an empty vector control and a no enzyme control were included in each plate. Based on sodium dodecyl‐sulfate polyacrylamide gel electrophoresis (SDS‐PAGE), 29 out of the 45 proteins in the library (64%) were clearly overproduced in soluble form (Figure S3).

### 2.2 Reactivity Profiling of Curated Enzyme Library

**FIGURE 3:**
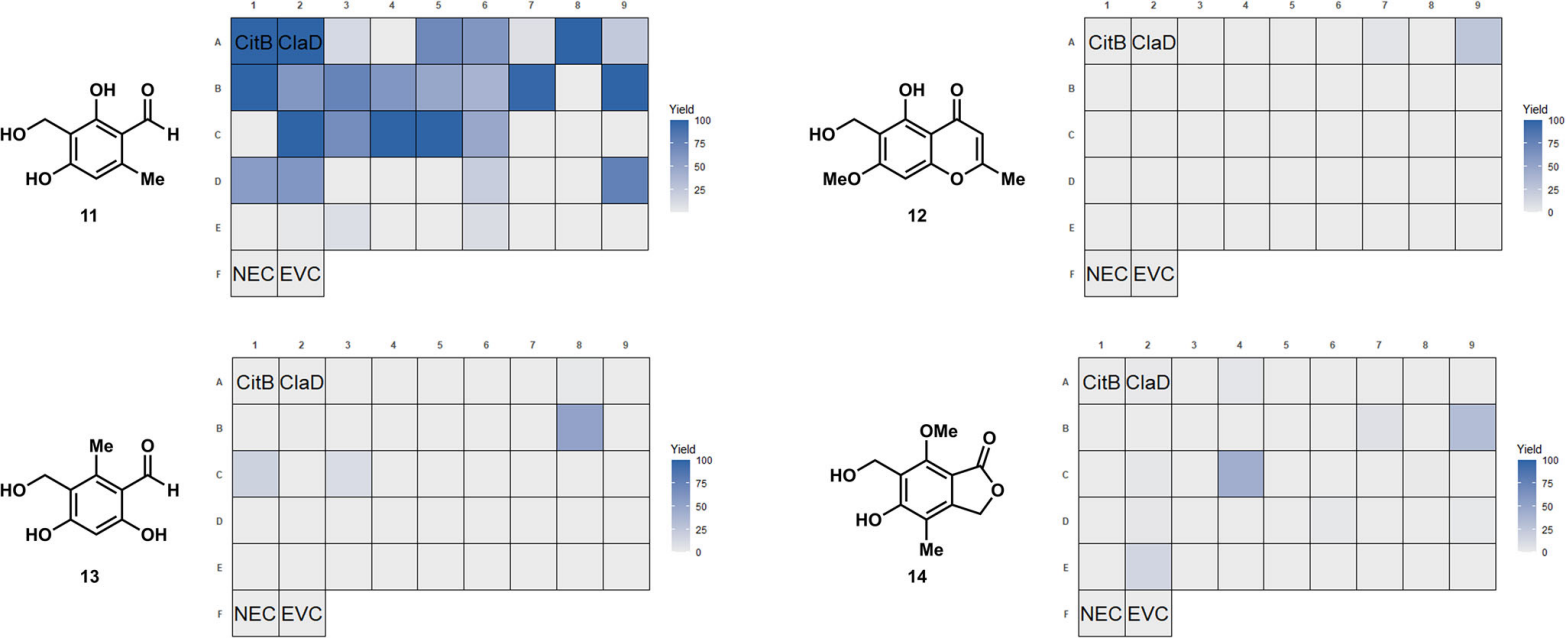
Profiling data for the production of alcohols **11–14** with the enzyme library. In each heatmap the best yielding enzyme is highlighted has a bolder blue color. Reactions are biological duplicates. Reactions were run in duplicate. Conditions: Substrate (2.5 mM), enzyme in the form of cell pellet, sodium ascorbate (4 mM), α‐ketoglutaric acid (4 mM), FeSO_4_ (0.2 mM), TES buffer (50 mM, pH 7.5), 10% v/v toluene, and 5% v/v dimethyl sulfoxide (DMSO).

With the library of putative hydroxylases in hand, we next assessed their activity in analytical‐scale reactions. Each enzyme/substrate combination was tested in reactions run in 96‐well plates along with control reactions. Specifically, each well contained substrate (2.5 mM), enzyme in the form of cell pellet, sodium ascorbate (4 mM), α‐ketoglutaric acid (4 mM), FeSO_4_ (0.2 mM), TES buffer (50 mM, pH 7.5), 10% v/v toluene, and 5% v/v dimethyl sulfoxide (DMSO). Each reaction plate was covered with a breathable film and shaken at 300 rpm (30° C) for 2–4 h. This whole‐cell format provided an operationally simple workflow which was previously demonstrated to work for CitB and ClaD [6]. We first tested the production of benzylic alcohol **11** by the library (Figure 3), because **11** is known to be produced by both CitB and ClaD from substrate **S8** [6]. The positive control reactions with CitB and ClaD showed substrate consumption and formation of **11** by high‐performance liquid chromatography‐diode array detector (HPLC‐DAD). Out of the 45 enzymes in the library, seven produced **11** with at least 80% yield, nine afforded 80%–50% yields, 10 yielded less than 50% of the product, and no hydroxylated product was detected for 17 enzymes. With confirmation of hydroxylation activity across several enzymes in the library (26 in total), we proceeded with assessing the activity of each enzyme with substrates **7–10**).

**FIGURE 4:**
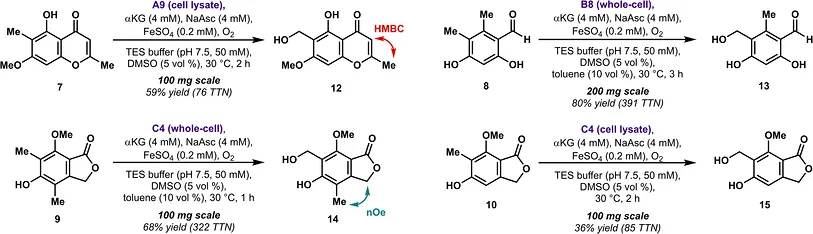
100–200 mg scale of enzymatic reactions with top enzymes identified through library profiling. αKG: α‐ketoglutaric acid. NaAsc: Sodium ascorbate. DMSO: Dimethyl sulfoxide.

As the site‐selective hydroxylation of substrates **7–10** is central toward accessing a suite of natural products, these compounds were next evaluated as substrates for each enzyme in the library. We started with chromone **7**, which is the precursor to the benzylic alcohol that would give access to chaetoquadrin D (**3**) and other *Chaetomium* chromone natural products [7, 8]. Chromone **7** was synthesized in four steps from commercially available materials and tested with CitB and ClaD to find that it was not productively oxidized to **12** (see Supporting Information). However, when the entire enzyme library was tested, A9 showed formation of a new product. The gene for the discovered enzyme A9 (UniProt accession number: A0A2J6SBY6) is from *Hyaloscypha variabilis*, which belongs to a family of fungi that grows on decaying plant matter [29]. The protein shares a sequence identity of 55% and 42% with CitB and ClaD, respectively. Carrying out a reaction of **7** with enzyme A9 on 100 mg scale afforded a single benzylic alcohol **12**, which could be isolated in 59% yield (76 TTN, Figure 4). We propose the structure of **12** based on the HMBC spectrum which shows a cross peak between a benzylic methyl‐^13^C and the aromatic C─^1^H that is next to the ketone (see Supporting Information). Thus, we observed the desired selectivity with just one out of the three methyl groups in **7** being hydroxylated.

In a parallel experiment, **8**, a potential precursor to phomeketale B (**4**), was tested as a substrate for each library enzyme. This compound was synthesized in one step (see Supporting Information). In analytical‐scale reactions, substrate **8** was oxidized to **13** by three previously uncharacterized enzymes (Figure 3), with one enzyme (B8) showing full conversion of starting material to a single product. Enzyme B8 (UniProt accession number: D4D5Z2) is native to *Trichophyton verrucosum*, an organism known as the “cattle ringworm fungus.” [30] The protein shares a sequence identity of 52% and 44% with CitB and ClaD, respectively. When this reaction was scaled to 200 mg of **8** in a whole‐cell format, a single product (**13**) was isolated in 80% yield (391 TTN, Figure 4). The site‐selectivity was high, affording just one of the two possible sites of benzylic hydroxylation of **8**. Specifically, the methyl group, that is, meta to the formyl group was the favored site of hydroxylation, which is consistent with the previously reported selectivity trend [6].

Toward the syntheses of mycophenolic acid (**5**) and austalide natural products, we envisioned the site‐selective benzylic hydroxylation of a lactone, such as **9** or **10**. Toward this goal, substrate **9** was accessed in five steps through a key base‐mediated benzylic hydroxylation and subsequent lactonization (see Supporting Information). Substrate **10** was synthesized in seven steps with a key formylation and subsequent Pinnick oxidation to form the lactone moiety (see Supporting Information). In analytical‐scale reactions, eleven enzymes, including ClaD, showed activity with substrate **9** to produce **14** (Figure 3). Similar results were observed for substrate **10** (see Supporting Information). Amongst the enzymes with activity, a previously uncharacterized enzyme (C4) produced the largest amount of product. The gene associated with enzyme C4 (UniProt accession number: A0A0U1LTB7) was sequenced from the genome of *Talaromyces islandicus*, which is a mold that grows on grain crops [31]. C4 shares a sequence identity of 56% and 49% with CitB and ClaD, respectively. The structure of benzylic alcohol product **14**, was confirmed based on nuclear Overhauser effect spectroscopy (NOESY) of the single isolated product from a 100 mg scale reaction (Figure 4). The structure of **14** was proposed based on its NOESY spectrum, which shows a cross‐peak between a benzylic methyl and the lactone methylene. This suggests that, both groups are adjacent in the aromatic ring. If there was hydroxylation at the other possible benzylic methyl, we would not observe the mentioned cross‐peak. Remarkably, despite the presence of four oxidation sites (three methyl groups as well as a benzylic methylene group), the biocatalytic transformation was selective for **14**, enabling studies toward mycophenolic acid. This site‐selectivity is consistent with that reported with related enzymes [6], in which the methyl group in between the two oxygen substituents was selectively oxidized. The same enzyme, C4, effectively oxidized substrate **10** to provide benzylic alcohol **15** in 36% yield (85 TTN). Benzylic alcohol **15** was envisaged to give access to (17*S*)‐dihydroaustalide K.

Overall, the curated enzyme library collectively demonstrated a broader substrate scope than the characterized enzymes associated with biosynthetic pathways, CitB and ClaD. We found that sixteen enzymes produced benzylic alcohol **11** with similar or moderate activity compared to CitB and ClaD. Additionally, eleven enzymes accepted non‐native substrates, such as **7–10**, which were not efficiently hydroxylated by CitB and ClaD (Figure 3). Product isolation and characterization further confirmed that of the best performing enzymes for each substrate (A9, B8, and C4) produced a single benzylic alcohol with site‐selectivity consistent with that demonstrated by CitB and ClaD (Figure 4) as exemplified by the site‐selective hydroxylation of substrates **8** and **9** [6].

### 2.3 Natural Product Syntheses

With compounds **12–15** in hand, we sought to advance these enzymatically generated benzylic alcohols to natural product targets **1–6**. The planned routes required capturing in situ‐generated *o‐*QMs with sulfur nucleophiles to arrive at chaetoquadrin D (**3**), chaetosemin A (**1**) and chaetosemin B (**2**), a dienophile to access phomeketale B (**4**), or carbon nucleophiles to afford mycophenolic acid (**5**) and (17*S*)‐dihydroaustalide K (**6**). Considering that sulfinates are nucleophiles [32] that can participate in sulfa‐Michael reactions [33, 34, 35], we synthesized sulfinate **17** by oxidation of *N*‐acetyl cysteamine. Heating **17** with benzylic alcohol **12** under acidic conditions furnished chaetoquadrin D (**3**) in 61% yield as the major product. No product formation was observed in the absence of acid.

**FIGURE 5:**
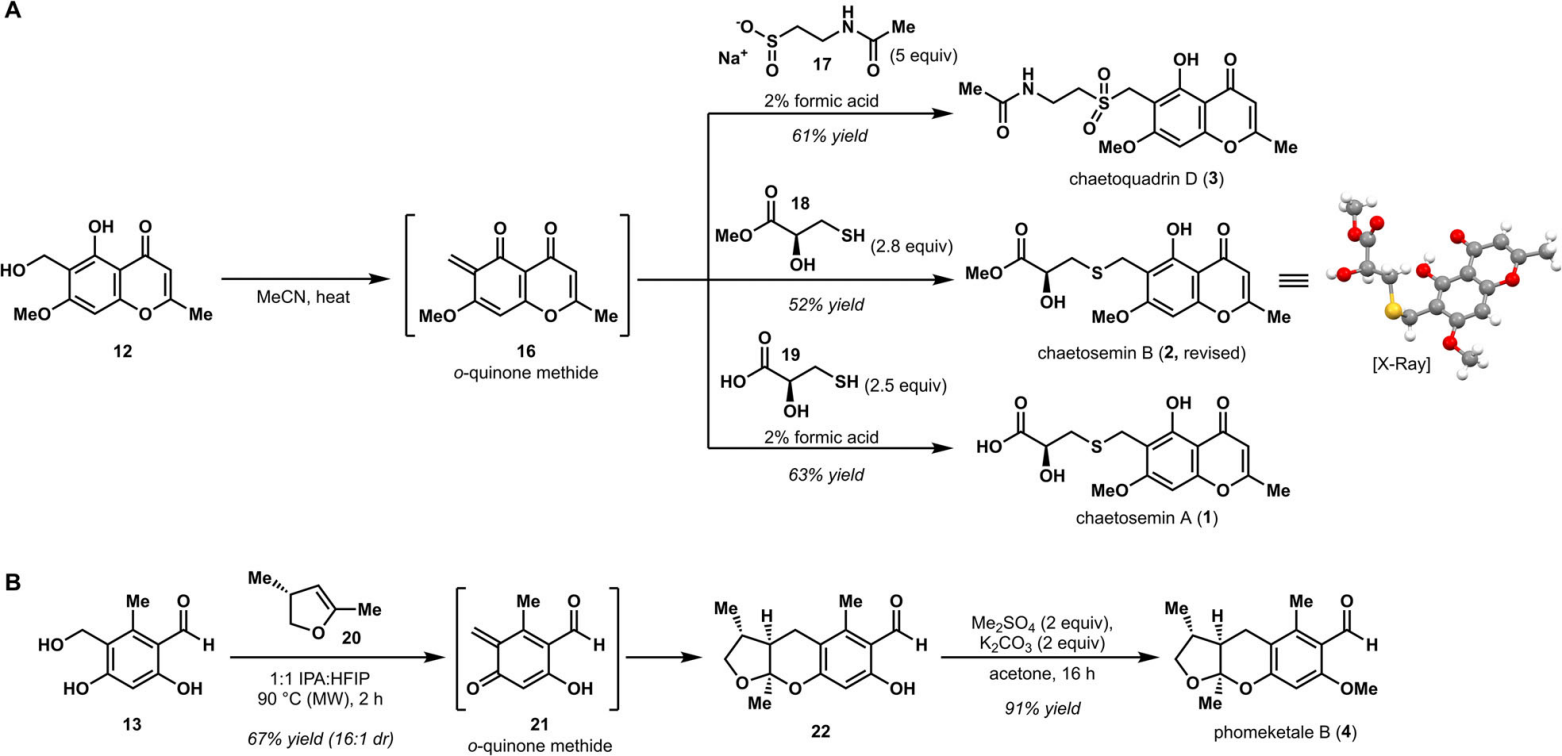
(A) Divergent approach toward *Chaetomium* chromones involves nucleophilic additions to *o*‐QM **16**. (B). Key cycloaddition with dienophile **21** and further methylation led to phomeketale B. IPA: Isopropyl alcohol. HFIP: Hexafluoroisopropanol.

Toward chaetosemin A (**1**) and chaetosemin B (**2**), thiols **18** and **19**, and their respective enantiomers (see Supporting Information, Compounds **S13** and **S14**), were accessed using a key diazotization of a *S*‐sulfonyl‐cysteine [36] and a phosphine‐mediated deprotection. Heating of benzylic alcohol **12** with thiol **S13** in acetonitrile for three days at 60° C led to the proposed structure of chaetosemin B (**S16**) as the main product. All characterization data for the obtained product agreed with the isolation report, except for the optical rotation (${\left[\alpha \right]}_{D}^{23}$: +7.7, isolation report ${\left[\alpha \right]}_{D}^{28}$= −6.3) [7]. Intrigued by this finding, we synthesized **2** by combining benzylic alcohol **12** and thiol **18**. The union of **12** and **18** afforded a product with the correct optical rotation (${\left[\alpha \right]}_{D}^{23}$: −6.6, isolation report ${\left[\alpha \right]}_{D}^{28}$= −6.3) [7]. Additionally, we confirmed the absolute stereochemistry of **2** with a crystal structure (Figure 5A) [37]. These findings suggest that **2** is the correct structure for chaetosemin B, revising the absolute stereochemistry of this natural product. For the synthesis of chaetosemin A (**1**), thiol **19** was heated with benzylic alcohol **12** for 16 h under acidic conditions to provide **1** in 63% isolated yield. The measured optical rotation value of ${\left[\alpha \right]}_{D}^{23}$= +11.1 agreed with the isolation report (${\left[\alpha \right]}_{D}^{28}$= +9.3) [7]. Overall, the total syntheses of three natural products were accomplished in a divergent fashion from **12**.

After accessing natural products by capture of an *o*‐QM with sulfur nucleophiles, we transitioned to the synthesis of phomeketale B (**4**). We envisioned this could be accomplished by capturing an *o*‐QM with a dienophile in an inverse demand Diels‐Alder reaction. Based on previously reported conditions [15], dienophile **20** and benzylic alcohol **13** were combined in hexafluorosopropanol:*i*PrOH (1:1) with microwave heating to furnish adduct **22** in 67% yield as a single diastereomer after purification (Figure 5B). Subsequent methylation with Me_2_SO_4_ led to **4** (**[α]_D_^23^** = –3.2, isolation report **[α]_D_^20^** = –22.3) [9]. As we observe the same optical rotation sign, we did not further investigate the absolute configuration assignment of **4**.

**FIGURE 6:**
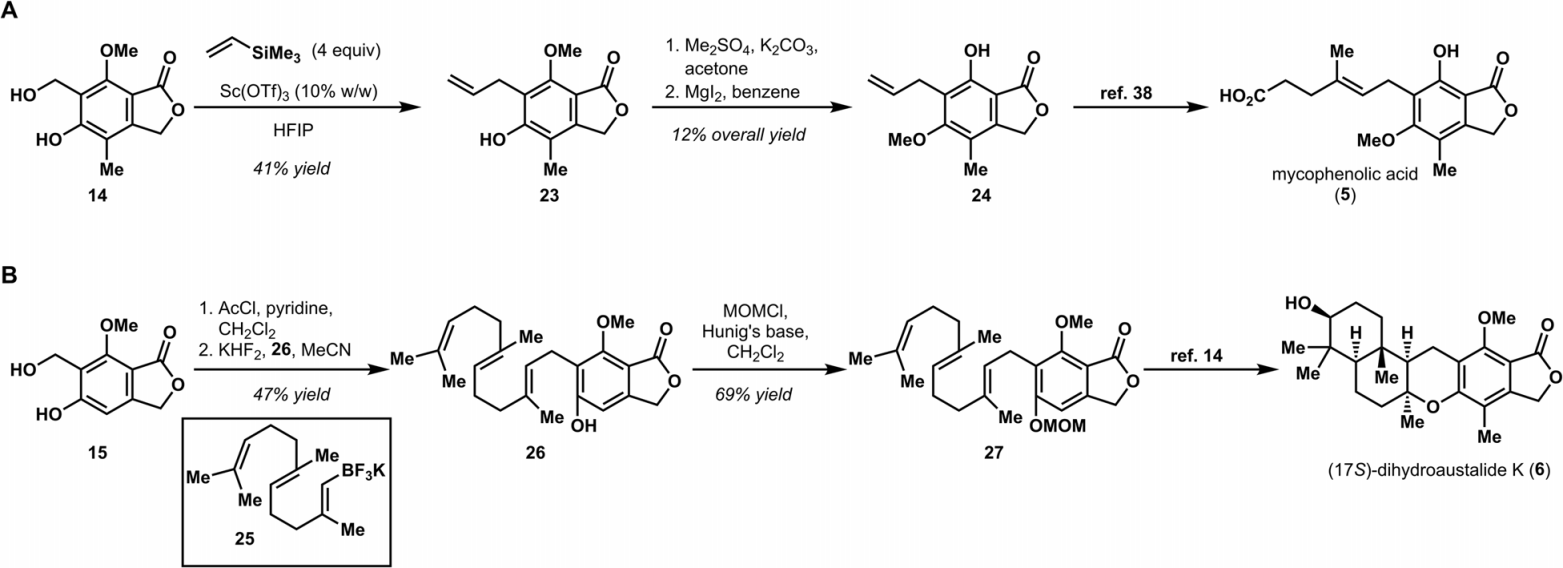
Formal syntheses of meroterpenoids mycophenolic acid and (17*S*)‐dihydroaustalide K. (A). Vinyl group addition to **14** and protecting group manipulation accomplished the formal synthesis of mycophenolic acid. (B). Addition of **25** to **15** and MOM protection accomplished the formal synthesis of (17*S*)‐dihydroaustalide K. HFIP: Hexafluoroisopropanol. MOMCl: Chloromethyl methyl ether.

Finally, we explored the elaboration of **14** and **15** with carbon nucleophiles to access meroterpenoid natural products mycophenolic acid (**5**) and (17*S*)‐dihydroaustalide K (**6**). Toward this goal, addition of trimethyl vinyl silane to **14** successfully furnished adduct **23** (Figure 6A). Adjustment of the methylation pattern of **23** led to compound **24**, an intermediate in a previously reported synthesis of **5** [38], accomplishing the formal synthesis of mycophenolic acid. For the synthesis of (17*S*)‐dihydroaustalide K (**6**), we attempted the addition of **25** to **15** (Figure 6B); however, only trace amounts of desired adduct **26** were observed when heating with KHF_2_ in Me_3_CCN at 100° C. Hypothesizing the thermal stability of **25** could be problematic, lower temperatures were investigated for *o‐*QM generation from benzylic alcohol **15**. Accordingly, **15** was first acetylated and the addition was attempted at 80° C, which successfully furnished adduct **26** in 47% yield. A subsequent MOM protection led to intermediate **27** that has been previously reported in the synthesis of **6** [14].

## 3 Conclusion

The selectivity and scalability of biocatalytic transformations hold untapped potential for target‐oriented synthesis. However, a common challenge to implement enzymes in a synthetic campaign is identifying a suitable enzyme to mediate a specific transformation on a target substrate. In this work, we explored the enzyme sequence space of previously characterized enzymes CitB and ClaD to build a bespoke enzyme library for benzylic hydroxylation of orcinaldehyde‐like substrates. By profiling this library, over 20 new enzymes with benzylic hydroxylation activity were discovered. Further, the best‐performing catalysts (A9, B7, C4) for each targeted substrate were instrumental in enabling the syntheses of six natural products and the structural revision of chaetosemin B.

## Author Contributions

**Gonzalo J. Villegas Rodríguez**: writing – original draft, writing – review & editing, conceptualization, methodology, validation, formal analysis, & investigation. **Evan O. Romero**: investigation, methodology, writing – review & editing, formal analysis, & conceptualization. **Jonathan C. Perkins**: investigation & data curation. **Diego N. Rojas**: investigation, methodology, & formal analysis. **Maximilian S. Hrigora**: investigation, methodology, & formal analysis. **Alison R. H. Narayan**: conceptualization, writing – original draft, writing – review & editing, funding acquisition, supervision, project administration, & formal analysis.

## Conflicts of Interest

The authors declare no conflicts of interest.

## Supporting information




**Supporting File**: anie73554‐sup‐0001‐SuppMat.pdf.

## Data Availability

The data that supports the findings of this study are available in the supplementary material of this article.
