# Supplementary material for: Synthesis of Six Natural Products Enabled by Selective Hydroxylation of Orcinaldehyde‐Like Substrates With an Enzyme Library
Source: Angew Chem Int Ed Engl. 2026 Jul 11;65(38):e4567477. doi: 10.1002/anie.4567477 (PMC13573132; doi:10.1002/anie.4567477)

Supporting Information for:

Synthesis of Six Natural Products Enabled by Selective Hydroxylation of Orcinaldehyde-like Substrates with an Enzyme Library

Gonzalo J. Villegas Rodríguez,[1,2] Evan O. Romero,[1,2] Jonathan C. Perkins,[1,2] Diego N. Rojas,[1,2] Maximilian S. Hrigora,[1,2] Alison R. H. Narayan*[1,2,3]

[1]Department of Chemistry, University of Michigan, Ann Arbor, Michigan 48109, USA. [2]Life Sciences Institute, University of Michigan, Ann Arbor, Michigan 48109, USA. [3]Program in Chemical Biology, University of Michigan, Ann Arbor, Michigan 48109, USA.

*Corresponding author, email: arhardin@umich.edu

Table of Contents

1. General information 3
2. Enzymatic library construction 4
3. Enzymatic library expression 5
4. Enzyme sequences 7
5. Enzymatic library profiling 21
6. TTNs for scale-ups 30
7. TTN and timecourse for production of 11 32
8. Chemical oxidations of target substrates 34
9. Synthetic Procedures and Characterization Data 59
10. X-ray data 91
11. References 93
12. ^{1}H and ^{13}C NMR spectra 95

1. General information

All reagents were used as received unless otherwise noted. Anhydrous solvents were degassed and dried over alumina columns on an MBraun solvent system (Innovative Technology, Inc., Model PS-00-3). Column chromatography was performed using SiliCycle SiliaFlash® P60 silica gel (40-63 μM). Preparatory HPLC purifications were conducted on a Waters 1525 Binary HPLC pump with a Waters 2489 UV/Visible detector. Diastereomeric ratios were determined by ^{1}H NMR spectroscopy, unless otherwise noted. ^{1}H and ^{13}C NMR spectra were obtained in $CDCl_3$ at 25 °C, unless otherwise noted, on Varian 400 MHz or Bruker 600 MHz spectrometers. Chemical shifts of NMR spectra were recorded in parts per million (ppm) on the δ scale. ^{1}H data are reported using the following format: magnetic field strength, solvent, δ chemical shift in ppm (multiplicity, coupling constant *J* in Hz, integration). Multiplicities for ^{1}H NMR are abbreviated as follows: s = singlet, d = doublet, t = triplet, q = quartet, quint = quintet, sext = sextet, sep = septet, dd = doublet of doublets, ddd = doublet of doublet of doublets, br = broad, m = multiplet, app = apparent. ^{13}C data are reported using the following format: magnet strength, solvent, δ chemical shift in ppm. Residual solvent peaks were used as internal reference for spectra using $CDCl_3$ (^{1}H: 7.26 ppm, ^{13}C: 77.2 ppm), DMSO-d_6 (^{1}H: 2.50 ppm, ^{13}C: 39.5 ppm), acetone-d_6 (^{1}H: 2.05 ppm, ^{13}C: 29.8 ppm), methanol-d_4 (^{1}H: 3.31 ppm, ^{13}C: 49.0 ppm), and acetonitrile-d_3 (^{1}H: 1.94 ppm, ^{13}C: 1.3 ppm). High resolution mass spectra were obtained on an Agilent 6230 Series Time-of-Flight (TOF) mass spectrometer. Liquid chromatography was performed on an Agilent 1290 HPLC system or a Waters Acuity H-class UPLC. Infrared spectra were recorded on a Perkin-Elmer Spectrum BX FT-IR spectrometer at 25 °C. Optical rotations were measured at room temperature (25 °C) using a 100 mm path length cell on a Jasco P-2000 polarimeter. For microwave reactions, a CEM Discover 2.0 microwave synthesizer was used. All microwave reactions were conducted in a sealed Pyrex glass microwave vial with a stir bar. After a short ramping period, reactions were held at a set temperature or microwave power and cooled to room temperature after the defined hold period.

Optimization of vinyl group addition to benzylic alcohol **14**.

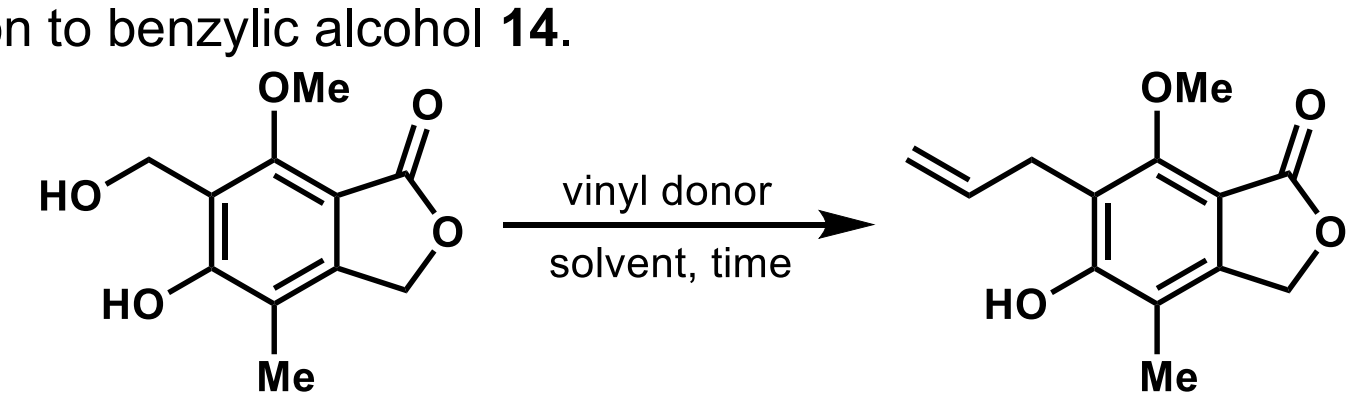

14 **23**

Entry	Vinyl donor	Solvent	Additive	Temperature (°C)	Time	Yield
1	Vinylboronic acid MIDA ester (5 equiv)	toluene	-	90	16 h	n.d.
2	Vinylboronic acid pinacol ester (4 equiv)	HFIP	-	80	16 h	n.d.
3	Vinylboronic acid pinacol ester (4 equiv)	toluene	-	80	16 h	n.d.
4	Vinylboronic acid pinacol ester (4 equiv)	HFIP	$Sc(OTf)_3$ 17 mol %	25	16 h	<1%
5	Trimethylvinyl silane (4 equiv)	HFIP	$Sc(OTf)_3$ 10% w/w	25	16 h	41%[a]
6	Vinylboronic acid dibutyl ester (5 equiv)	toluene	-	80	16 h	n.d.
7	Potassium vinyl trifluoroborate (5 equiv)	MeCN	-	80	15 h	<10%
8	Potassium vinyl trifluoroborate (5 equiv)	isopropanol	-	80	15 h	n.d.
9	Potassium vinyl trifluoroborate (5 equiv)	HFIP	-	80	15 h	31%[b]

Yield was calculated based on the areas of product and starting material, as analyzed by HPLC-DAD. All reactions were run on 5 mg scale in 250 μL of solvent. [a]Isolated yield on 40 mg scale. [b]Isolated yield on 5 mg scale. HFIP: hexafluoroisopropanol. n.d. = not determined.

2. Enzymatic library construction

To build the enzymatic library for our synthetic endeavors, we first built a Sequence Similarity Network (SSN)[1] using publicly available bioinformatic tools BLAST[1] and EFI-EST.[2] The reported amino acid sequence of CitB (UniProt: A0A159BP93)[3] was queried into BLAST (10^{-5} E-value) to find 1046 sequences and these were uploaded to the EFI-EST to generate the SSN that was visualized in the program Cytoscape.[1] Within SSNs, individual protein sequences are shown as a node (circles in Supporting Fig. S1) and lines between nodes are present if the protein sequences meet a defined alignment score threshold. For Supporting Fig. S1, we set an alignment score so that the connected protein sequences shared an alignment score of 98.

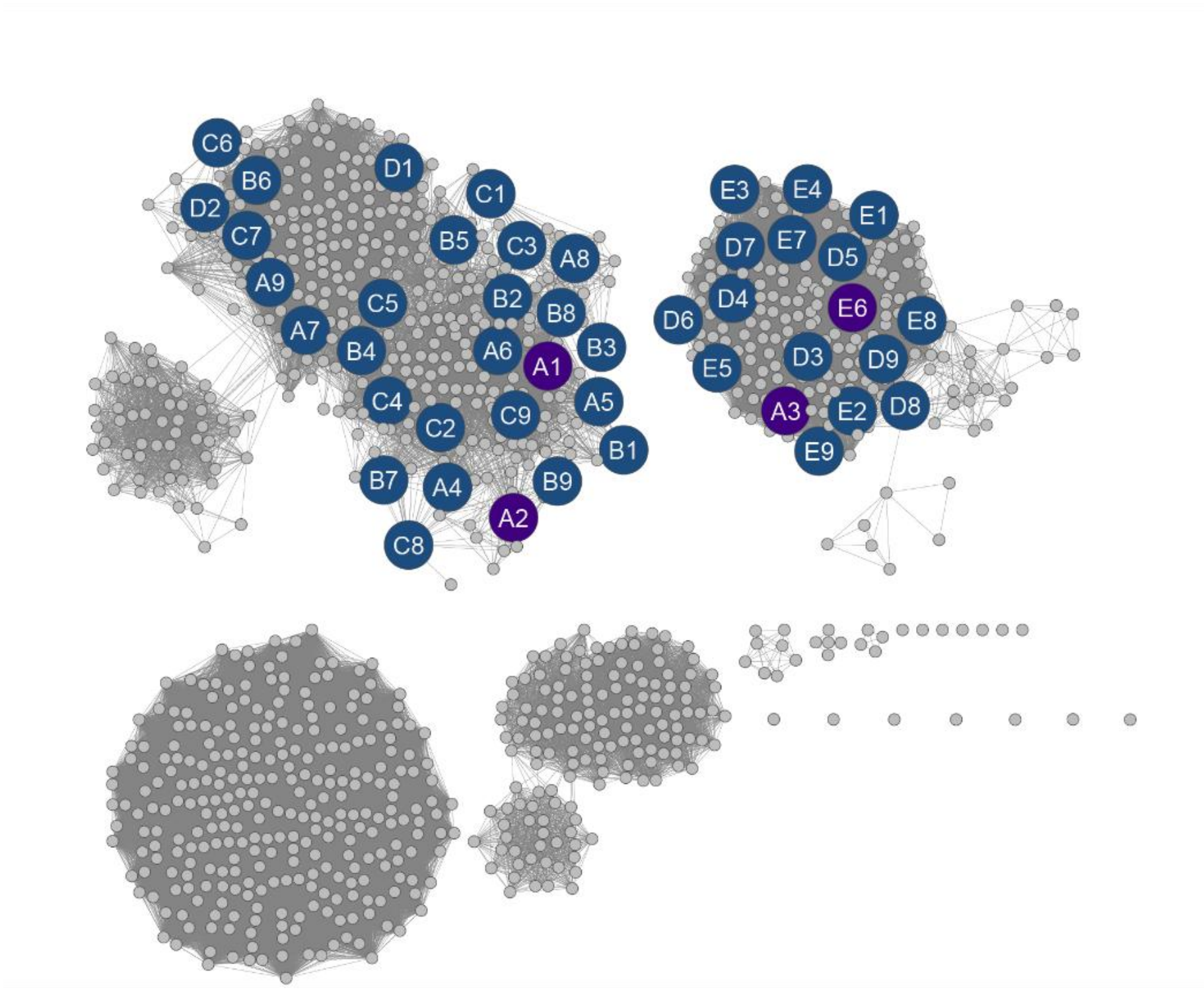

Supporting Figure S1: SSN built based on the sequence of CitB and visualized in Cytoscape. Enzymes for library construction were selected from two cluster. Purple circles represent enzyme sequences in the library that have been previously characterized. Blue circles represent uncharacterized enzyme sequences in the library.

Out of all the protein sequences in the network, only four have been studied: CitB[3] (NHI_A1), ClaD[4] (NHI_A2), TropC[5] (NHI_A3) and XenC[6] (NHI_E6). The remaining sequences had no known function or native substrate and were classified by the UniProt databases as putative Fe(II)/α-ketoglutarate dependent dioxygenases. We observed that the enzymes that perform benzylic hydroxylation on phenolic substrates (CitB, ClaD) are in the same cluster. Native substrates and reactivities of characterized enzymes can be observed in Supporting Fig. S2.

To build the library we selected 28 enzyme sequences from the CitB/ClaD cluster and 17 sequences from the TropC/XenC cluster (blue and purple circles in Fig. S1), giving a total of 45 fungal enzymes. We decided to pick enzymes from different clusters to have more diversity. The chosen enzymes share an average sequence identity of 46%; with the lowest sequence identity being 31%, and the highest, 98%. We also had an empty well for a noenzyme control and a well with an empty vector control (pET-28a(+) with no expression gene). In total we had 47 occupied wells, which allowed us to have two copies of the same library per 96-well plate.

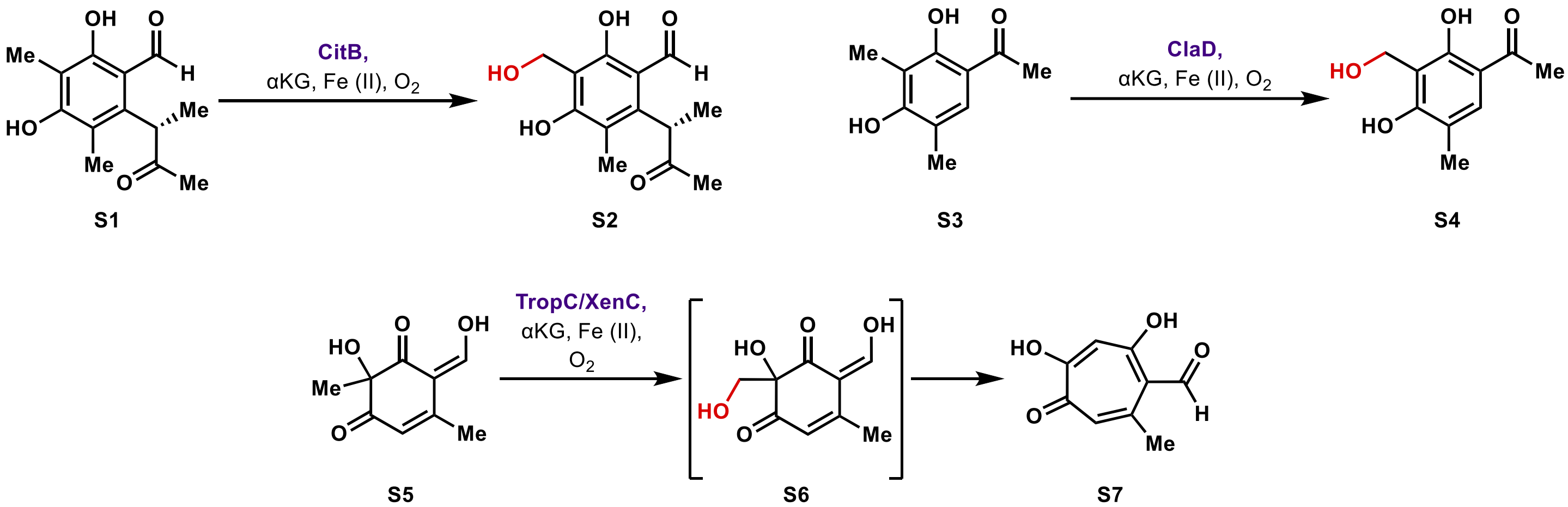

Supporting Figure S2: Native reactions for characterized enzymes that were included in this work's enzyme library. CitB[3] (NHI_A1), ClaD[4] (NHI_A2), TropC[5] (NHI_A3) and XenC[6] (NHI_E6).

3. Enzymatic library expression

Plasmid information: All genes for enzymes in the library were codon-optimized, synthesized, and cloned into a pET-28a(+) vector by Twist Biosciences. Each vector contains a T7 promoter, kanamycin resistance gene, and N-terminal 6xHis tag.

Transformation: Chemically competent BL21(DE3) *E. coli* cells were transformed with a pET-28a(+) vector encoding for each enzyme using a standard heat-shock transformation protocol. Included in this transformation was a pET-28a(+) plasmid that does not encode for a specific protein of interest, to serve as an empty-vector control. The transformed cells were used to inoculate 1 mL portions of Luria Broth (LB) containing 50 µg/mL kanamycin at 37°C while shaking at 200 rpm for 1 h. The cultures were plated on agar plates with kanamycin (50 µg/mL), which were incubated overnight at 37 °C. The obtained colonies were used for culture inoculation.

Glycerol stock library preparation: In a sterile 2 mL deep-well 96-well plate, to each well was added 500 µL of Luria Broth (LB) media containing kanamycin (50 µg/mL) and each well was inoculated with the colonies from the transformation step. The inoculated plate was incubated at 37 °C while shaking at 200 rpm overnight, the plate was covered with a breathable film. The overnight growths were used to make a plate of glycerol stocks (100 µL of culture and 100 µL of 50% v/v glycerol aqueous solution) which were stored at –80 °C for future use.

Expression: A plate with LB media was inoculated as described in **Glycerol stock library preparation**. 10 µL of the overnight growth were used to inoculate 450 µL portions of 4% v/v glycerol Terrific Broth (TB) containing kanamycin (50 µg/mL) in 2 mL deep-well 96-well plates. Given the size of our library (45 enzymes, empty-vector control, and no enzyme control) we had two sets of enzymes per plate. Cultures were covered with breathable films and grown at 37 °C while shaking at 300 rpm until the optical density at 600 nm (OD_{600}) reached 0.6–0.8 for several wells. Then, the cultures were cooled to 20 °C for 20 min. Protein expression was induced with isopropyl-β-D-1-thiogalactopyranoside (IPTG, 0.2 mM final concentration) and the cultures were allowed to continue at 20 °C while shaking at 300 rpm overnight. The plates were removed from the incubator and centrifuged at 1250 x g for 20 min. Then the supernatant media was carefully poured off the plates and the plates were inverted onto paper towels to absorb any excess supernatant. The resulting plates that contained cell pellets were covered with adhesive foil covers and stored at –80 °C for up to 6 months. These were the plates that were used for reactions and SDS-PAGE experiments.

SDS-PAGE experiment: Cell lysis was accomplished with four freeze-thaw cycles with liquid nitrogen, followed by incubation in 400 µL of 50 mM Tris-HCl (pH 7.5), 300 mM NaCl, and 10% glycerol containing 1 mg/mL of lysozyme at 25 °C (200 rpm) for 1 h. SDS-PAGE analysis of clarified lysate is shown in Supporting Fig. S3. Gels were prepared by polyacrylamide polymerization. Samples were mixed 2x Laemmli sample buffer, heated at 95 °C for 10 minutes and loaded into gels. Electrophoresis was run for 60 minutes at 180 V. Gels were stained with Protein Ark Quick Coomassie Stain.

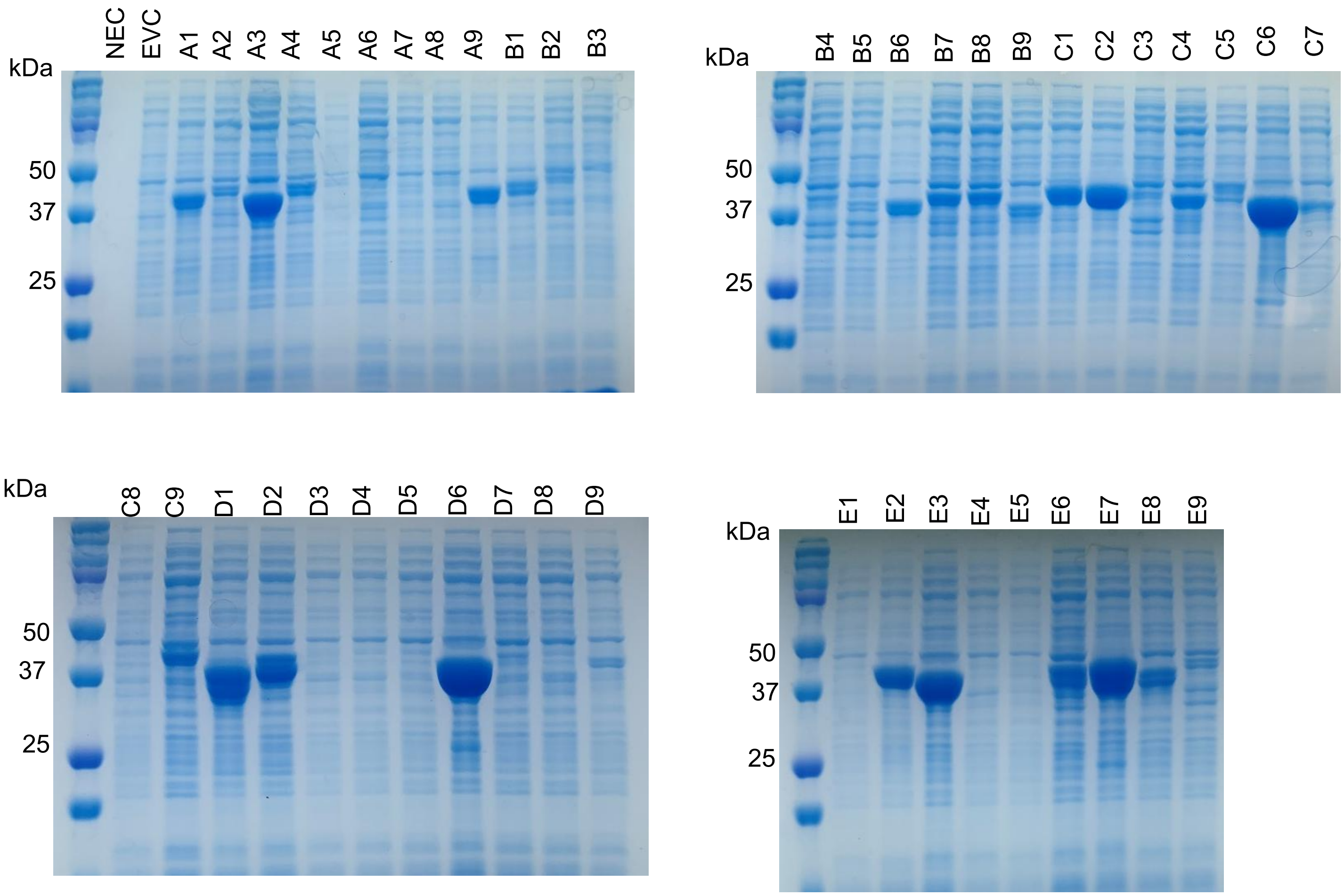

Supporting Figure S3: SDS-PAGE gels of the clarified lysate for all the enzymes in our library. NEC: No enzyme control. EVC: Empty vector control. Enzymes that show an overproduced band: A1, A2, A3, A4, A9, B1, B2, B4, B5, B6, B7, B8, B9, C1, C2, C3, C4, C5, C6, C7, C9, D1, D2, D6, E2, E3, E6, E7, E8.

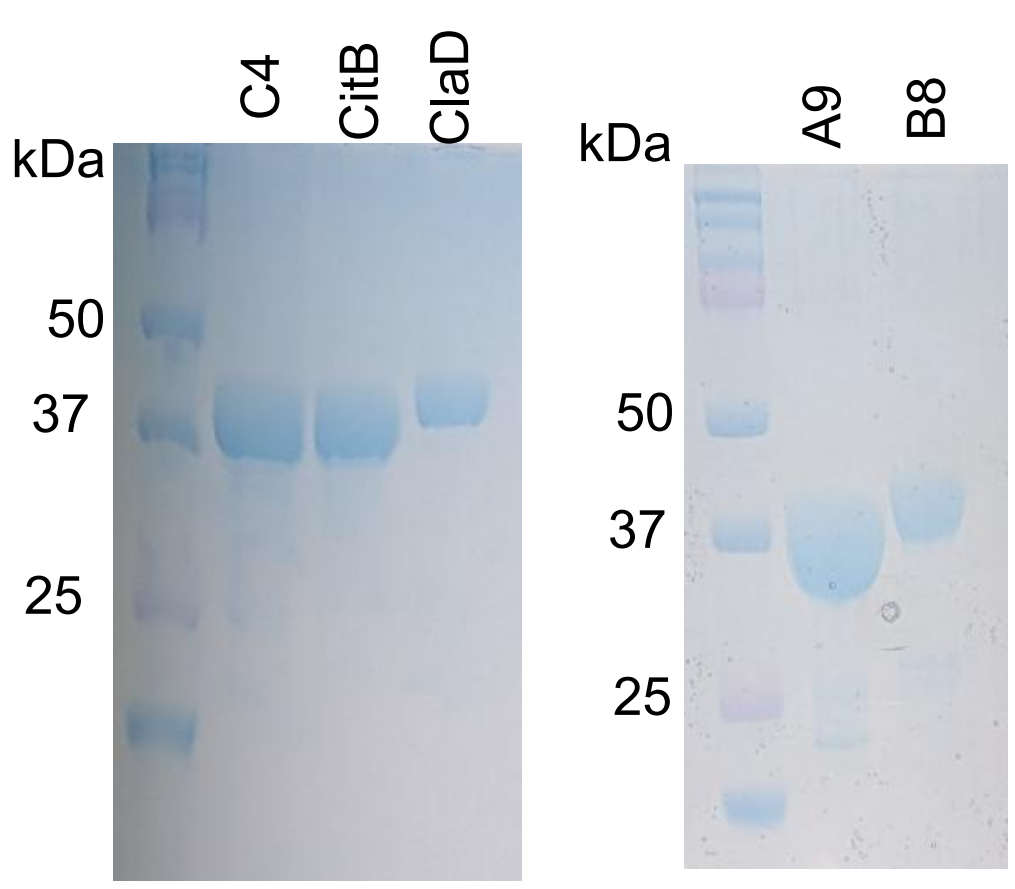

Supporting Figure S4: SDS-PAGE gels of purified enzymes.

4. Enzyme sequences

A1 (CitB) codon-optimized sequence:

ATGCCTATTAGCACTAAGTCGAGTTTCTACCTTCCCGCCGTTGATATCAGTCCATATTTACAAGACCCCAACTCTGACGCAGCAAGAAA
GGTTATAGACGACGTGCGGGCCGCCTGCACTAGCACGGGTTTCTTCCAATTACTTGGACATGGGATTTCGCCTGCACTTCAACAGTCGG
TATTCGCGGCAGCTGCCAAGTTCTTTGCCTTGCCGTCAGATGTTAAGTCACGTTGCCGCAACGTAGGTTTCCGCGGTTACGACCCAATG
GCAAGCCAGTCTTACGAGTTAGGAGTGCTGCCCGATCTGAAAGAGGGGTTCATCGCCGGCAAAGACATCCCTCTTGACGATCCGCGGGT
CGCATCTCAGCGGTTCTTCATGGGTCAGAACGCTTGGCCGCCTTCCGAGCTGCTCCCCGAGGCGAACTTTCGCCGACCTATTGAGGAGT
ATTACCAGGCCATGCTCAAACTGTGTTGGGTAGTCCTGGACTTAGTAGCAGCAACTTTACCTTACGGTCCGCATGTTTTCGACGAGTTC
AAAGAGAACGACCCTGCTTGCCCCTTACGGCTTCTGCACTACCCACCTGCTCCAGCGCCTGATGTGGCTAAGGGGCGTCAACTCGGGTC
CTCAGCCCATACAGACTTTGGCGCTATTACACTTTTGCTTCAGGACGACCATAGCGGACTTGAGGTGCAAGACTGCGAAACCGGTGAAT
GGATCGGCGTGCCACCTAACAAGGATGCGTACGTGGTGAATTTAGGCGACATGATGTCGCGTATCACCAGAGGCCATTATAAGTCTTCC
ATTCATCGTGTGATCAACCAAAACTTGACAGACCGTTATAGCGTCGTGTTCTTCTTCGATGGGAATCTTGACTACCGGCTTCGACCCTT
GGATAGAGTTGGTCAAAACTGGGACGAAGAGGACACACTTACTGTGGAAGAACACATGCTCGAACGTACAACCACGACATATAACCTGA
AGGTGAAGTAG

A1 (CitB) protein sequence (UniProt A0A159BP93):

MPISTKSSFYLPAVDISPYLQDPNSDAARKVIDDVRAACTSTGFFQLLGHGISPALQQSVFAAAAKFFALPSDVKSRCRNVGFRGYDPM
ASQSYELGVLPDLKEGFIAGKDIPLDDPRVASQRFFMGQNAWPPSELLPEANFRRPIEEYYQAMLKLCWVVLDLVAATLPYGPHVFDEF
KENDPACPLRLLHYPPAPAPDVAKGRQLGSSAHTDFGAITLLLQDDHSGLEVQDCETGEWIGVPPNKDAYVVNLGDMMSRITRGHYKSS
IHRVINQNLTDRYSVVFFFDGNLDYRLRPLDRVGQNWDEEDTLTVEEHMLERTTTTYNLKVK

A2 (ClaD) codon-optimized sequence:

atgccggtgctgtcgaatccgagcttttatctgccactggtggatatcaccccgttcctggagaacccgcatggcgcagcggcgcaggatgtcatcgaaagcgtgcgtaccg
cctgcaaaagcaccggtttttttcagatcaaaggtcatcaggtgccgctgcgtttgcaaaaatcggtgttcgaagccagcgcgcgtttctttgcgctgcctctcaaaaacaaact
cgaactcgacagccgcaaaaccgttggcttccgcggttacgatgtcatggaaacgcaaagctacgaactggaatttggcgcggtgcaggaagcggatgcgttgcgcgat
atcaaagaaggctttttattgccaccgacctgccgccggatcatccgcatgtcgccaacggtcgtttttttgcagggcccgaacgtctggccgaaaccagaacagctggcgc
cggaagatttccagagcgtactggaagaatattacaccgaaatgcagcgtctgtctcatgtggtgttgtcgctgctggcagcaacgctgccgtatggtccgcacgttttcgacg
agctggaaacctgcgatccgatgtcgttgctgcgcctgctgcattatccgcgcggcttggaaaaacaggatggcaaaaaactgcaactgggtgccggggaacataccgat
tttggtacctttaccctgttactgcaggacgagcatcctggcctggaagtgcaagacagtgtcaccggcgaatggcacggcgtgccaccgcaggaggatgtttatatcgtcaa
cgttgccgatattctgtccaccatgaccgagggcgattacaagtcttctgtgcatcgcgtgtggaacatcaagagcaacgatcgctacagcgtcgtgtttttctacgacggcaa
cctggattacaaagtcaaaccgttacgcagcagcggtcaggacgaaaacgaagaaatcgatgcaccgaccatcgaagagcacgtccgttcgcgtctcactgccagttat
gccatttaa

A2 (ClaD) protein sequence (UniProt A0A481WNL2):

MPVLSNPSFYLPLVDITPFLENPHGAAAQDVIESVRTACKSTGFFQIKGHQVPLRLQKSVFEASARFFALPLKNKLELDSRK
TVGFRGYDVMETQSYELEFGAVQEADALRDIKEGFFIATDLPPDHPHVANGRFLQGPNVWPKPEQLAPEDFQSVLEEYYT
EMQRLSHVVLSLLAATLPYGPHVFDELETCDPMSLLRLLHYPRGLEKQDGKKLQLGAGEHTDFGTFTLLLQDEHPGLEVQ
DSVTGEWHGVPPQEDVYIVNVADILSTMTEGDYKSSVHRVWNIKSNDRYSVVFFYDGNLDYKVKPLRSSGQDENEEIDAP
TIEEHVRSRLTASYAI

A3 (TropC) codon-optimized sequence:

atgtccattggtgacgaagtcataccgacggttgatataagtgcgtggctctcgtccaccgcgagcccggagtcaaagaataaggttgtcgaggaagttcgttcggcgtgtaa
taaatatggattcttcaatcttgtaggtcacggtatcccggctgaggcccgcgagaaaattttcgggtgcactaagaaattcttcgacctgcccctggaagaaaagatgaagat
ctccgttgacaagtcacttggcaagtctttccgcgggtatgagccttccctgatccaaactcaccaagacggtttgttaccagacacgaaggagtgcttcattactggggcaga
aattccggctgaccaccctgatgctggtaaattctcaactgggcccaatctgtggccagagggcctgtcagacaaggaattccgtcaacccgtgatggagtatagagcgctg
atgctggacctcgtgtcaaccattgtgcgcatcttaggtcagggcattcataaggctttcggccatcctagtgatgtacttaatgacatcctcattaatcctagcatccccatgcga
ctgcttcactatgctcctcaagagaacccggatccacgacaatttggagttggcgaccacactgattttggctgtgtctctatactgctgcagcaaaagggtacgaaaggcctg
gaagtgtggtacccacccaaagagacgtggataccagtacctgtcatcgaagacgccttcgttattaacatgggcgatactatgcatcgttggaccggcggttactacagaa
gtgcccgccaccgtgtatacataacgggtgagcgtcggtacagcgttgcattcttcttgaacggtaatctgaatctgaagataaagcctttggatggaagtggcggagaagct
tcagtaggagaacacatcaatagtagactcgcacatactttgggagacaatgctaaatacctgcgttga

A3 (TropC) protein sequence:

MSIGDEVIPTVDISAWLSSTASPESKNKVVEEVRSACNKYGFFNLVGHGIPAEAREKIFGCTKKFFDLPLEEKMKISVDKSLG
KSFRGYEPSLIQTHQDGLLPDTKECFITGAEIPADHPDAGKFSTGPNLWPEGLSDKEFRQPVMEYRALMLDLVSTIVRILGQ
GIHKAFGHPSDVLNDILINPSIPMRLLHYAPQENPDPRQFGVGDHTDFGCVSILLQQKGTKGLEVWYPPKETWIPVPVIEDA
FVINMGDTMHRWTGGYYRSARHRVYITGERRYSVAFFLNGNLNLKIKPLDGSGGEASVGEHINSRLAHTLGDNAKYLR

A4 codon-optimized sequence:

atgccagttcccacgaaaagctcctttacctgcctgccgtggacatttcgccatacctgcaagaccccaattctgacggcgctcgcaaagttattaacgacgtccgggctgc
atgtactagcacggggtttttcagttgctgggccacgggatctctcccgagttgcaaagatccgtgttcgcagccgcagcgaagttctttgctctgccaacggatgtgaaaagt
tcctgtagaaatgtcggctttcggggctacgaccccatggctagccaatcatatgagcccggcgtcttgcctgacttaaaagaatcttttatatccgggaccgatattccccttga
cgatccaagagtaatgtcgagacgtttctttatgggccagaatgcatggcccccaggcgagttattgccggaaaccgactttcgtcgtcccgtagaagagtattaccaggcg
atgatgaagctttgttgggccgtcttggacttagtcgcagcaactttaccgtatggcccacacgtctttgacgagtttaaagaaaatgatcccgcttgcccattgagattgctgcat
tatcccccaacaccggctcctgatgtcgttaaggggcgtcagttggggtcatccgctcacaccgatttggcgccatcacattgcttctgcaagacgaccatcccggactgga
agtacaagacagagaaaccggggactggataggtgtcccaccgaaaagtgatgcttacgtggttaacttaggagacatgatgagcagaattacccgtgggcattacaagt
cgtctattcatcgcgtaataaacaagaaccttactgatcgctacagcgttgtattttttttgatggcaaccttgactacaagcttcgtccactggatcgcgtcgagcaagaccaag
atgaggagaacgtcttgactgtcgaagaacatatgctggaacggacaacgacgacatataacaccaaagctaagtga

A4 protein sequence:
MPVPTKSSFYLPAVDISPYLQDPNSDGARKVINDVRAACTSTGFFQLLGHGISPELQRSVFAAAAKFFALPTDVKSSCRNV
GFRGYDPMASQSYEPGVLPDLKESFISGTDIPLDDPRVMSRRFFMGQNAWPPGELLPETDFRRPVEEYYQAMMKLCWA
VLDLVAATLPYGPHVFDEFKENDPACPLRLLHYPPTPAPDVVKGRQLGSSAHTDFGAITLLLQDDHPGLEVQDRETGDWIG
VPPKSDAYVVNLGDMMSRITRGHYKSSIHRVINKNLTDRYSVVFFFDGNLDYKLRPLDRVEQDQDEENVLTVEEHMLERTT
TTYNTKAK

A5 codon-optimized sequence:
atgccttcttctaccaaatcgagcttttatttgccttcggtaaatatcacgccttatttgcaggaccccgattcaagtgaggctcaaaaggtgatagacgacatcagaaccgcctg
ccgtagtacggggtttttcagttactgggacacgggattccgcgcactttacagcagtccgtgtttgatgcgggaaaaagattctttgccttacctactgacgttaaattagggat
ggattgtcgtcggaatgtaggatttcggggctatgatgcgatggcgagccaatcatatgaatcgggcgtgctgccagatttaaaagaaggttttatgatgggcaccgatatgtc
cgagaacgacttccgtgtgattaatagacgtttcttcatggggccgaatgtatggcccccggaatctcttttaaagaaggaagacttccgtcagccgttggaaacttactttgaa
gcaatgatgaaactgtgttggactgtaatggatctggtcgccgcgacacttccatatggaccgaacatatttgacgagtggaaggctaatgatcctgcttgcccattcagattgc
tgcattacccgccaaccccagaacctgtagaaggacaaaaacggcaattgggaagctcggcccataccgatttcggagctataaccttgcttttgcaggacgaacacgct
ggtttggagatccaggatcgcgagacaggagaatggattcccgttccacccaaggaagatgcctatgtaattaacatgggagatatgatttcccggataacggacggacat
tataagtcctcgatacaccgtgtcattaacagaaattctaatgatcgctactcaatagtatttttctttgacggtaacattgactacaagctgagacgcttagactccaaagtgcag
agtgaggacgaggcccttactgtggaggagcatatggaggaaagaagaaacactacgtacgggcttaaaaaaaaatga

A5 protein sequence:
MPSSTKSSFYLPSVNITPYLQDPDSSEAQKVIDDIRTACRSTGFFQLLGHGIPRTLQQSVFDAGKRFFALPTDVKLGMDCR
RNVGFRGYDAMASQSYESGVLPDLKEGFMMGTDMSENDFRVINRRFFMGPNVWPPESLLKKEDFRQPLETYFEAMMKL
CWTVMDLVAATLPYGPNIFDEWKANDPACPFRLLHYPPTPEPVEGQKRQLGSSAHTDFGAITLLLQDEHAGLEIQDRETG
EWIPVPPKEDAYVINMGDMISRITDGHYKSSIHRVINRNSNDRYSIVFFFDGNIDYKLRRLDSKVQSEDEALTVEEHMEERR
NTTYGLKKK

A6 codon-optimized sequence:
atgcccagcagtaccaaaaattcttctttctatttgccccttgtgaacatcaccccttacctgcaagatccctcgagcatcgctgggcaacaagtactggacgatgtacgcaga
gcttgcacgagcactggattctttcaattggttgggcacggcgtaccggcaacgcttcagaagaatctttacaatgcggctgccaagtttttcgccttaccaatggacgtcaaatt
acctttggatgtgcggaagaatataggcttcagaggatatgatgttatgtcgagccagagttatgagaaggacgtcttacccgatttgaaagaaggctatatcgtgggcaccg
acattcccttagacgatccaagagtcatgaaacgtcggtttttcatgggccgtaacatttggcccccgacggagttcttgccatacgaggacttccaaaaggtggtagaggaa
tataatgatgcgatgttgcggttatcatgggtagttgtcgatttgattgcagcgacgttgccctacggaccccatgtattcgatgaacttaaggagaacgatccagcatgtccgtt
gcgcttattacactatcccccaacccctaaacaagtagggaatgagcgcagacaatttggcagctctgcgcatacggactttggctttatcactcttttgcttcaagacgagaat
cctggtcttgaggtgcaagatagagagactggtgagtggatcggtgtacccccgaacgaagaggcgtacgttgtcaacatgggtgacatgatcagcagagtcacaggga
acttatacaagtccagcctgcaccgcgtccttaataaagcacccactgaccgctattccgtggtgtttttcgtggatggtaacctggattacaaattgcgcagattagataaaatc
ggacagccgggggaagatgaagaggcgctgacggttgaggaacacatggaagaacgccgtcgcaccacatatggtcttcggaaaaaatga

A6 protein sequence:
MPSSTKNSSFYLPLVNITPYLQDPSSIAGQQVLDDVRRACTSTGFFQLVGHGVPATLQKNLYNAAAKFFALPMDVKLPLDV
RKNIGFRGYDVMSSQSYEKDVLPDLKEGYIVGTDIPLDDPRVMKRRFFMGRNIWPPTEFLPYEDFQKVVEEYNDAMLRLS
WVVVDLIAATLPYGPHVFDELKENDPACPLRLLHYPPTPKQVGNERRQFGSSAHTDFGFITLLLQDENPGLEVQDRETGE
WIGVPPNEEAYVVNMGDMISRVTGNLYKSSLHRVLNKAPTDRYSVVFFVDGNLDYKLRRLDKIGQPGEDEEALTVEEHME
ERRRTTYGLRKK

A7 codon-optimized sequence:
atggcctcaccaatacccttttcagtaccaaccatcgacttcaccgcatatttatcagaccccacgtccgctgaatcatcgaagattgtctccgacattaaaactgcgtgtacga
ctactggatttttcagcttatcggtcatgggatcccggcatctttacaagaggacgtctttaagggttcaaaagccttgttcaagttgccgatggaggagaaggtcaaacttgac
cgcggcaagtctgtcggtgcgtcaaaccgcgggtatgagttgataggagggcaaggtttgcaagagggtacgttaccggatttgaaggaaggattttatgttggccaggata
tccctgccacggacccgcgtgtccaaaataacgcgtttttgatgggtccaaacctttggccgtcgagtgacttggtatcagaggcggtattcaaaaagccgatggaaaagtat
ttcagtaagatgttcacgcttacccttaaagtcatggacgccatagcagcaggaatgccatatggtagttcagtattcgatgaatttacgtcaaacgacgcggtatcgtctatac
gtcttttacactatccccggacaaaagtaacaacgagttgcagctgggtgcgggggcgcatacagactttggtgcgattactttactgttacaagaccagatcggtggcttac

aagtctgggattatgggaacaaagagtggaaggatgtgacaccggtcaaggacgcatacgtcgtcaacgtcggagatatgcttcaaatgtggacctccggggtctataa
agctcgttgcaccgggtattgaacagatccggtaaggaccgctacagcgtgccatttttcttcgacggtaacgtagattgtgtacttactccacttggaggcggggaggaagg
caaaaagtatccaactgtggaagagcacatgaaggaacgctttggccagacttacgggcgcggaaaaaagagagaagatggtggcgaaaacgtcaagttctag

A7 protein sequence:
MASPIPFSVPTIDFTAYLSDPTSAESSKIVSDIKTACTTTGFFQLIGHGIPASLQEDVFKGSKALFKLPMEEKVKLDRGKSVGA
SNRGYELIGGQGLQEGTLPDLKEGFYVGQDIPATDPRVQNNAFLMGPNLWPSSDLVSEAVFKKPMEKYFSKMFTLTLKVM
DAIAAGMPYGSSVFDEFTSNDAVSSIRLLHYPPDKSNNELQLGAGAHTDFGAITLLLQDQIGGLQVWDYGNKEWKDVTPV
KDAYVVNVGDMLQMWTSGVYKSSLHRVLNRSGKDRYSVPFFFDGNVDCVLTPLGGGEEGKKYPTVEEHMKERFGQTYG
RGKKREDGGENVKF

A8 codon-optimized sequence:
atgaaaacaatgttcgtttcctacagattaaatatctgcgtgcctaccgtagacataagtccctatctggccgatccgaagagtgacgcggcccagaaggtcatcgaggatgt
gcgcgaagcctgcatttcgactggctttttcagatcactggccacggtatttcaaaatcgttgcaacagaatatatttgatgcagcccattcgttcttcgcccttcctctggaagaa
aaaaagaagttgaacgccgccaacttcatcgggcatagaggctacgacgtcttagcctcgcaatcctacgaggaaggcgttttgcctgacttaaaagaaggctactatgttg
ggtcagacgtgcttccttccgacccgttgtacggtagattcttcatgggccagaacgtgtggccaagcacagaacttttgtcgatcattcaattccaggaaccctgcgagcggt
atcatgagtcagttcaacacttggcctttaaagtgttgcagctggtaggtgatacaatgataccgcatggacactcgacagttggaatgacggaaaatatgcctacagtgttac
acaacttgatccgtttggacaaaatcccagcctgccctcttcggctgcttcactacccgtcagcaacccgcaatggggggttcagttaccggaaagcctcaatacggagcgtct
gctcacaccgactttggggtaataaccttacttctgcaagatgataatcccggcttagaagtgttagtcgaaaaagatgggaaacaggtgtggtggccaatcgatcctaaccc
ctccgcatacgtggtcaacataggggatatgatcagcatggtgaccaatgggatctacaaatcatctatgcatcgggtcgtttgcaagcgccctgagcgcgaacggtattcc
atagtcttctttctggatggatgcctgaacgctaacttagagcctgtcgagggcttcggtcatccagaggagggtgtgggattctcccacaagactgtagaacagcacatgac
ggaacgtttatcaatgagctatgcaaaagaggggaaagacccccgtcataatgaagacaccttctccgcctga

A8 codon-optimized sequence:
MKTMFVSYRLNICVPTVDISPYLADPKSDAAQKVIEDVREACISTGFFQITGHGISKSLQQNIFDAAHSFFALPLEEKKKLNAA
NFIGHRGYDVLASQSYEEGVLPDLKEGYYVGSDVLPSDPLYGRFFMGQNVWPSTELLSIIQFQEPCERYHESVQHLAFKV
LQLVGDTMIPHGHSTVGMTENMPTVLHNLIRLDKIPACPLRLLHYPSATRNGGSVTGKPQYGASAHTDFGVITLLLQDDNP
GLEVLVEKDGKQVWWPIDPNPSAYVVNIGDMISMVTNGIYKSSMHRVVCKRPERERYSIVFFLDGCLNANLEPVEGFGHP
EEGVGFSHKTVEQHMTERLSMSYAKEGKDPRHNEDTFSA

A9 codon-optimized sequence:
atggcgattgcgagttcattttccgttcccacgatagacatatcgccttatctgtctgacgccacaagcgaggctgggctgaaggtgatcgaggatgttaaagcggcgtgcac
cggaaccgggtttttcaacttatagggcacggcatatccccggaacttcaggaaaaggtcttcaagggtgccgaacaattgtttgctttaccgatggaggaaaaagttaagtt
agataaatcgcagagcgtgggagcgtccaaccgcggctatgaactgataggcgggcagggacttcaagagggcaccttaccagatttgaaggagggtttctacgtgggc
caagacatccccgcttcggacccgagagtccagaagaaagctttcttaatgggtcccaatttatggccgccctccgatcttgttcccgaagatgtgtttaaggaaccaatgac
atcgtactacgcgcagatcttcgctttgagccttaaagttttggaggtaatagcagcgggaatggaatatccaggcgtcaaaacggtcttcgaggaattcacggccaatgatg
ctgttgcctcgatacggcttttacactatccacctgataagggtaacgacgcacggcaactgggtgctggcgctcataccgattttggcgccatcacgttattgttgcaggacga
tatcggaggtcttcaggtctgggactacgtcggcaaagagtggcgggatgtcaaaccggttaaggaggcatatgttgtaaacgtcggagacatgcttcagatgtggacgag
cggcgtctacaaatcctcacttcatcgtgtagtaaatcgctctggccgtgaccggtattcagttccatttttcttcgacgggaatgtagattttgagttgcgtccgttggatggaaga
gaacgcgaggggggtgttttaacagtcgaggcgcatatgaaggaaagattcgcaagcacttacggacgcggcgctcagaaagtggcaggtctttga

A9 protein sequence:
MAIASSFSVPTIDISPYLSDATSEAGLKVIEDVKAACTGTGFFQLIGHGISPELQEKVFKGAEQLFALPMEEKVKLDKSQSVG
ASNRGYELIGGQGLQEGTLPDLKEGFYVGQDIPASDPRVQKKAFLMGPNLWPPSDLVPEDVFKEPMTSYYAQIFALSLKVL
EVIAAGMEYPGVKTVFEEFTANDAVASIRLLHYPPDKGNDARQLGAGAHTDFGAITLLLQDDIGGLQVWDYVGKEWRDVK
PVKEAYVVNVGDMLQMWTSGVYKSSLHRVVNRSGRDRYSVPFFFDGNVDFELRPLDGREREGGVLTVEAHMKERFAST
YGRGAQKVAGL

B1 codon-optimized sequence:
atgcctgccgccagcgcaactaccagctctttttatctgcctgacatcaacatagcgccttggttggaagacccggggtccccatcgggccagcacgtagtaaatgaagtcc
gccgtgcttgcacgtcaaccggtttcttccagttgacgggtcacggtttcccgcccgcagtcattaaagatatttttgcggcagccgccaaatttttcgctttaccaaaggagatta
aacaagacctgaatttcacgaaaaatgcgggatttagaggttatgagctggtaggcgcgcaagtctatgagactgatgtcctgcccgacttgaaagaggggtttctggctggt
atagacttaccgcgcgacgacgttcgcgtgcaaaataaaagatttttgcggggcaaaacgtctggcccagtccggacgtcttaccttacagtgagttcagaaccccccgtgg
agacctattataaaagcatcatgcaattatgttggacggtgatggatctgatcgcttccactttgccctatggtccaaatgtcttcgatgaatggaaagctcacgagcctgcgtgt
ccgcttagactgctgcactatccaccaacgccaaagcatgtggcaggaaagacccgccagcttggatccagcgctcatacagactttggcggtgtaacacttcttctgcaag
actcccacgacggtcttgagattctggatcgcgaaacccgttcttgggtccctgtgccccccaaaccgggtgcgtttgtgataaacgtggcggacatgatgaccatgataacc
ggaggggagtacaagtcgagcatgcaccgtgtaataaaccagaatgaaacggatcggtattctgtcgtgtttttatggatggaaatgttgattacaaattaagacgcttggac
aagattggtcagcctgttggggaagacgaggaacttataactgtagaggagtacatgttgggaaagcggaatacgacgtacgtaaagtag

B1 protein sequence:
MPAASATTSSFYLPDINIAPWLEDPGSPSGQHVVNEVRRACTSTGFFQLTGHGFPPAVIKDIFAAAAKFFALPKEIKQDLNF
TKNAGFRGYELVGAQVYETDVLPDLKEGFLAGIDLPRDDVRVQNKRFFAGQNVWPSPDVLPYSEFRTPVETYYKSIMQLC
WTVMDLIASTLPYGPNVFDEWKAHEPACPLRLLHYPPTPKHVAGKTRQLGSSAHTDFGGVTLLLQDSHDGLEILDRETRS
WVPVPPKPGAFVINVADMMTMITGGEYKSSMHRVINQNETDRYSVVFFMDGNVDYKLRRLDKIGQPVGEDEELITVEEYM
LGKRNTTYVK

B2 codon-optimized sequence:
atgaccaaaggcagcagcccaaatgtgccaacggtggacatctcaccatatctggaagatccaaaaagcaaagccgcccgtaaagtgatcgatgatgttcgcgaagcg
tgcacgtcaaccggctttttccaaatcacgggtcacggcattagcaaagagcaacaaaaatcggtgtttgatgcggcgcacgccttcttcgcgttaccattagaagagaaaa
agaaactgaatgccgcgcactttatcggccatcgtgggtatgatgttttagcgtcgcaatcatacgaggaaggtgtattaccggatttaaaagagggttattatgtaggctcgga
tattctgccgagcgatccactgtatggccgtttctttatgggtagcaacgtgtggccgccggcgaccttactgagcgcctcacagtttcaggaaccgtgcgaacgctatcataa
aaacgtgcagcaactggcgtttaaagtgctgcaattaattggtgatacgatgatcccatgtgagcgtagcacgatctcgatgacgggtggcgtaccgacggttctgcacgattt
aattcgcttagatgaaacgccggcgtgcccgctgcgtctgttacactatccgtcagcggcgcgtcacggtgatgttgccggtaaaccgcaatatggtgcgagcgcgcacac
ggatttcggcgtaatcacgttactgctgcaagatgataacccaggcctggaagttctggttgagaaagatgggaaacaggtttggctgccaattaacccgaacccagatgcc
tatgtggtgaatatcggggatatggcgtcgatggtgacgaatggggtatataaatcgagcatccaccgtgtggtgtgcaaacgcccggaacgcgagcgttatagcatcgtatt
tttcctggatggctgtttagatgcgtgtctgcgtccgatcgaggagttcgggcatccggagggtgggatcgcggatagccacaaaaccgttgagcagcacatgatcgaacgtt
tatcaatgagctatgcgaaagagggcaaagatccacgtcataacgaggatacgttttcggtgtaa

B2 protein sequence:
MTKGSSPNVPTVDISPYLEDPKSKAARKVIDDVREACTSTGFFQITGHGISKEQQKSVFDAAHAFFALPLEEKKKLNAAHFIGHRGYDVLASQSYEEG
VLPDLKEGYYVGSDILPSDPLYGRFFMGSNVWPPATLLSASQFQEPCERYHKNVQQLAFKVLQLIGDTMIPCERSTISMTGGVPTVLHDLIRLDETPA
CPLRLLHYPSAARHGDVAGKPQYGASAHTDFGVITLLLQDDNPGLEVLVEKDGKQVWLPINPNPDAYVVNIGDMASMVTNGVYKSSIHRVVCKRP
ERERYSIVFFLDGCLDACLRPIEEFGHPEGGIADSHKTVEQHMIERLSMSYAKEGKDPRHNEDTFSV

B3 codon-optimized sequence:
atgaaaacgatgttcgtttcgtatcgtctgaacatttgtgagttatattttgtgattctgcaacaaccaacgcacaaagttaaagatcgctcaccaacgatccatccgtcgcgcga
tatagcctgcatcgtatcacaatttattctgaatagcgataaaatgacgaaaggtagcttctcaggtgtgccaacggtggacatctcgccatatctggcggatccaaaaagcg
atgccagccgcaaagtgatcgaagatgttcgtgaagcgtgcatctcaacggggtttttccagattacgggtcatggcattagcaaatcgctgcaacaaaacatttttgatgcgg
cgcatagcttcttcgccttaccattagaagagaaaaaaaaactgaatgccgcgaattttatcggtcatcgtgggtatgatgttttagccagccaaagctatgaagaaggcgtat
taccagatttaaaagagggttattatgtaggcagcaacgtgctgccaagcgatccattatatggtcgtttttttatgggccagaacgtgtggccatcaacggaattactgagcac
gatccgctttcaagaaccatgcgagcgctatcacgaaagcgtgcagcacctggcctttaaagtgctgcaattagtaggtgatacgatgatcccacacgggcatagcacggt
gggtatgacggaatcgatgccaacggtgctgcacaatttaattcgtttagataaaatcccggcgtgtccgctgcgtctgctgcactatccaagcgccacgcgtaacggtgggt
cggttacgggcaaaccgcaatatggtgcgtcagcgcacacggatttcggtgtaattacgttactgctgcaagatgataatccaggcctggaagtgctggtggaaaaagatg
gtaaacaagtttggtggccaatcgatccgaatccaagcgcctatgttgtgaatatcggtgatatgatcagcatggtgacgaacggcatctataaaagcagcatgcatcgtgtg
gtatgcaaacgtccagaacgcgagcgctatagcatcgtatttttctggatggctgtttaaacgcgaatctggagccagtagagggtttcgggcacccagaagaaggcgtgg
gtttttcgcacaaaaccgtagaacaacacatgacggaacgtttatcaatgagctatgcgaaagagggcaaagatccacgtcataacgaggatacgttttcggcgtaa

B3 protein sequence:
MKTMFVSYRLNICELYFVILQQPTHKVKDRSPTIHPSRDIACIVSQFILNSDKMTKGSFSGVPTVDISPYLADPKSDASRKVIE
DVREACISTGFFQITGHGISKSLQQNIFDAAHSFFALPLEEKKKLNAANFIGHRGYDVLASQSYEEGVLPDLKEGYYVGSNV
LPSDPLYGRFFMGQNVWPSTELLSTIRFQEPCERYHESVQHLAFKVLQLVGDTMIPHGHSTVGMTESMPTVLHNLIRLDKI
PACPLRLLHYPSATRNGGSVTGKPQYGASAHTDFGVITLLLQDDNPGLEVLVEKDGKQVWWPIDPNPSAYVVNIGDMISM
VTNGIYKSSMHRVVCKRPERERYSIVFFLDGCLNANLEPVEGFGHPEEGVGFSHKTVEQHMTERLSMSYAKEGKDPRHN
EDTFSA

B4 codon-optimized sequence:
atgagctcaacgacggatcggagcccacatcagaaaaaattttatattccacaggttaatattgaaccattcctgaaagatccagatagcgccacgagcaaaacgattattg
aagaagtgcgtgccgcctgccgttcaaccggtttttttgaaatcacgggccacggcatcccaaaaagccttcagaaagaagttttcggtgccgcgtcgcagttttttaaactga
gcttcgatgaaaaaaaacgtttagatgcgtcaaaacagattggttatcgtggttatgatatgcttgcgtcacaggcctatggcaaagatctgcttccggatttaaaagaaggtttt
tatattggcatagatataccaaaaacggatcctcgtgcgggtcgtttttttacgggcccaaatgtgtggccagatgaaagcctgctgccagccagccagtttaaagatccatgc
accatgtatttttcagccatgcttcagctgagcttccgtataatggccctgctggcgcgtacgctgccatacggtccacatattttcgatgaatttatcgccaacgatccagccgcc
ccacttcgtctgttacactatccgccaccaccaatggcctcagttggcccagaacagcagaaaaaacagttcggcgcctcggcgcataccgatttcggtgccataaccatat
tacttcaggatgaaaatccgggtctggaagttctggatcaggatgccgatagctgggttccaatcgatccgaatccaggtgcctatgttgtgaatgttggtgatatgctgtcgttat
ggacgtcgggtgaatataaatcgagcgttcatcgcgttatcagcaaaaatccgacggatcggtatagcgttgtttttttcttagatgggaatttagattgttcattaaacccattaga
tggctcggaaaaccggggtccgaaaacggttgaagaacacatgacccggcggttaacggcctcgtatggggttgaaaaagaaaaagattaa

B4 protein sequence:

MSSTTDRSPHQKKFYIPQVNIEPFLKDPDSATSKTIIEEVRAACRSTGFFEITGHGIPKSLQKEVFGAASQFFKLSFDEKKRL
DASKQIGYRGYDMLASQAYGKDLLPDLKEGFYIGIDIPKTDPRAGRFFTGPNVWPDESLLPASQFKDPCTMYFSAMLQLSF
RIMALLARTLPYGPHIFDEFIANDPAAPLRLLHYPPPPMASVGPEQQKKQFGASAHTDFGAITILLQDENPGLEVLDQDADS
WVPIDPNPGAYVVNVGDMLSLWTSGEYKSSVHRVISKNPTDRYSVVFFLDGNLDCSLNPLDGSENRGPKTVEEHMTRRL
TASYGVEKEKD

B5 codon-optimized sequence:
atgggaagcgtggctcctcctctggttccaacgattgatattgcggcgtttctggcggattcaacctcagaagaggcggaagatatagtagatgcggttcgtcaggcgtgcac
gaaaaccggctttttcagattaccggtcatggatttccagaagcggttcaggatgcgatgttccaggcgagcaaaaaatttttcgcgctgccaatggaagaaaaagtgaaa
ctggatgctaaaacgacgattggctatcggggctatgatgtgctggcgtcacaggtgtatgatacctcaggagaagtattacctgacctgaaagaaggcttttatgtaggccat
gatctgccgctgtcagatccagtggtagtagcgcgccgctttttatgggcccaaacgtgtggccaccagtgagctcactggcggctgcggaatttcagcatccagtggaggc
gtatcatggcgcggtgaacagcctggcgctgaaaatgctggatctggtggcgcgcaccctgccatacgatacgaaacaaattttcgcggaatttgcgaccggctatgttattg
cgccgctgcgtctgctgcattatccgccggcggataccgatgctgcgacccagacccgtccgcgcaccggggcgggcgcgcatacggatatgggcgcgattaccctgctg
ctgcaaaacgatcagccagggctggaagttctgcatccaggcaccggcgaatttgtggcggtggatccaaccccaggcgcgttcgtggtgaacgtgggcgatatgcttagc
gcgtggacgggcgggctgtataaaagcagcgttcatcgcgtgattaaccgtaacccagtggatcgctattcagctgcgttctttttgatggctcactggattgcccgctggacc
cgctggatgggagcggcaccggaccaaaaggggaactgaccgtggaaaaacacgttataaatcgtattatgcagagctattaa

B5 protein sequence:
MGSVAPPLVPTIDIAAFLADSTSEEAEDIVDAVRQACTKTGFFQITGHGFPEAVQDAMFQASKKFFALPMEEKVKLDAKTTI
GYRGYDVLASQVYDTSGEVLPDLKEGFYVGHDLPLSDPVVVARRFFMGPNVWPPVSSLAAAEFQHPVEAYHGAVNSLAL
KMLDLVARTLPYDTKQIFAEFATGYVIAPLRLLHYPPADTDAATQTRPRTGAGAHTDMGAITLLLQNDQPGLEVLHPGTGEF
VAVDPTPGAFVVNVGDMLSAWTGGLYKSSVHRVINRNPVDRYSAAFFFDGSLDCPLDPLDGSGTGPKGELTVEKHVINRI
MQSY

B6 codon-optimized sequence:
atgccatcagcgattgaacaaccaggatttcatataccaacggtcgatatagcgagctatcttgctgatccgagcagcgctgatgctcaacaggtggtagacgctgtacggc
tggcttgcacaacgagcggctttttcaactggtgggccacggcatacctataacactgcgggctacgatgtttgcgggctcaaaagctctgtttgagttaccgtttgaccagaa
aaaagcgctgaaacgggctggaaatcggggctatgaggtgatgggctcacaggcgctgcaagatggcaccctgccagatatgaaggaaggctattatattggcgagga
aataccatttgaagaaggcaaagcgtaccgcccgtttatggaaccaaacatttggccatcagaagaacagattccacatagcatttttcggctgccaatgaacgaatattata
aacgggtgtcagcgctgggcgtggtggtgatggatattttttgcggcggcgcttccttatggcccaaacgtgtttgataaattaaaacaagaaccttcagcggcgagcatacgc
ctgcttcattatccgcctcagcggacgacggatgaaaagcagctgggcgcgggggcgcatacggactttggcgtgctgaccctgctgctgaccgatggccatccaggcctg
caagtcctgaacgctgcgaccgacacctgggtgcctgtaccaccggatgcggacgcgtatgtggtgaatgtaggagatatgttacagcagataacggacggatattttaaa
agcaacacgcatcgggtggtgaacttaggagacgacgaccggtatagcgtaccgtattttttcgatgggtgtctggactgtcggttagaacggttagatggaaagcacgaag
agaaagtattaacggtcgaggaacacatgaacgaacggttcgctaccacctacggacggggccagcaaaaaaccgcttaa

B6 protein sequence:
MPSAIEQPGFHIPTVDIASYLADPSSADAQQVVDAVRLACTTSGFFQLVGHGIPITLRATMFAGSKALFELPFDQKKALKRA
GNRGYEVMGSQALQDGTLPDMKEGYYIGEEIPFEEGKAYRPFMEPNIWPSEEQIPHSIFRLPMNEYYKRVSALGVVVMDIF
AAALPYGPNVFDKLKQEPSAASIRLLHYPPQRTTDEKQLGAGAHTDFGVLTLLLTDGHPGLQVLNAATDTWVPVPPDADA
YVVNVGDMLQQITDGYFKSNTHRVVNLGDDDRYSVPYFFDGCLDCRLERLDGKHEEKVLTVEEHMNERFATTYGRGQQK
TA

B7 codon-optimized sequence:
atggcgagctcagcgacgagcagcccatatattccggttgttgatattaccccatttttagagaatccacaggggacggccgcgcaacaggttgtggataacgttcggcgtg
cctgcatgtcaacggggtttttctgatgaaaggccacggcatcagcccaaaactgcaaaactcagtgtttcaggcgtcagccaaatttttgcgttaccgcacgaggccaaa
ctgagcttagatgcgcggaaaaatgtgggctttcgtggctatgatgttatggagtcacaatcatacgagctggaaacccgtagcggcccacaagatgggtcagaagtaatg
cgcgatacgaaagaaggctttttgccagcaccgatgtgccactggatcacccacgcgttgccgccggccgtttcctggttggtccaaatgtttggccggatgccgatgtgctg
agccacgaagatttccgtgccccactggaagaatatttaggggagatgaaacgtctgagccacgttgtgctgaatctgattgccgcgacgctgccatacgggccgcacgtat
tcgatgaactggaagccgatgatccaatgtgcctgctgcgtctgctgcactatccgccgaccccacacagcgtagatggcatgaaattaggcgccggcgcccacacggatt
tcggtgccattaccctgctgttacaggatgagcatccaggcctggaggtgctggatgagaaaaccggcgagtggcagggcgttccgccacaaaaagatgtgtatatcgtta
acatggccgatattatgagcattctgaccaaaggtgcctataaaagcagcgttcatcgggtttggaacaaaaatacggaagatcgctatagcgtggttttttctttgatggcaa
cctggatctgaaactgaaaccgctggatgggacggaacaacgccaggataataaccaggagattctgacgctggaggagcatgtgcgcggccgtatgaccggtagctat
agcatcccaaaaaattaa

B7 protein sequence:
MASSATSSPYIPVVDITPFLENPQGTAAQQVVDNVRRACMSTGFFLMKGHGISPKLQNSVFQASAKFFALPHEAKLSLDAR
KNVGFRGYDVMESQSYELETRSGPQDGSEVMRDTKEGFFASTDVPLDHPRVAAGRFLVGPNVWPDADVLSHEDFRAPL
EEYLGEMKRLSHVVLNLIAATLPYGPHVFDELEADDPMCLLRLLHYPPTPHSVDGMKLGAGAHTDFGAITLLLQDEHPGLE
VLDEKTGEWQGVPPQKDVYIVNMADIMSILTKGAYKSSVHRVWNKNTEDRYSVVFFFDGNLDLKLKPLDGTEQRQDNNQ
EILTLEEHVRGRMTGSYSIPKN

B8 codon-optimized sequence:
atggcgaaaggtaacttctcgggggtgccaaccgtggatataagcccatatctgacggatccaaaaagcgatgccgcccgtcgtgtgatcgaagatgtccgtgaagcgtg
cgtgtcaacggggtttttccagattacgggtcatggcattagcgaatcgctgcaacaaaaaatttttgatgcggcgcatgccttcttcgcgctgccattagaagagaaaaaaa
actgaatgccgcgaactttatcggtcatcgtgggtatgatgtcttagccagccaaagctatgaagaaggtgtattaccagatttaaaagagggttattatgtaggcagcgatgt
gctgccaagcgatccattatatggtcgtttttttatgggccagaacgtgtggccatcaacgggtttactgagcccaatccagtttcaagaaccatgcgagcgttatcacgagag
cgtgcagcacctggcctttaaagtgctgcaattaatcggtgatacgatgatcccacacgggcatagcacggtgggtatgacggaaaatatgccaacggtgctgcacaattta
attcgtttagataaaattccggcgtgtccgctgcgcctgctgcactatccaagcgccacgcgcaacggtggttcggtcgccggcaaaccacaatatggtgcgtcagcgcaca
cggatttcggtgtaattacgctgctgctgcaagatgataatccaggcctggaagtgctggtggaaaaagatggtaaacaagtctggtggccaatcgatccgaatccaagcg
cctatgtcgtgaatatcggtgatatgatcagcatggtgacgaacagcatctataaaagcagcatgcatcgcgtggtatgcaaacgtccagaacgtgaacgttatagcgtggt
attttttctggatggctgtttaaacgcgaatttagagccaatcgagggctttggtcacccagaggaagggttcgggttttcacacaaaaccgtagaacaacacatgacggaac
gtttatcaatgagctatgcgaaagagggtaaagatccacgtcataacgaggatacgttttcggcgtaa

B8 protein sequence:
MAKGNFSGVPTVDISPYLTDPKSDAARRVIEDVREACVSTGFFQITGHGISESLQQKIFDAAHAFFALPLEEKKKLNAANFIG
HRGYDVLASQSYEEGVLPDLKEGYYVGSDVLPSDPLYGRFFMGQNVWPSTGLLSPIQFQEPCERYHESVQHLAFKVLQLI
GDTMIPHGHSTVGMTENMPTVLHNLIRLDKIPACPLRLLHYPSATRNGGSVAGKPQYGASAHTDFGVITLLLQDDNPGLEV
LVEKDGKQVWWPIDPNPSAYVVNIGDMISMVTNSIYKSSMHRVVCKRPERERYSVVFFLDGCLNANLEPIEGFGHPEEGF
GFSHKTVEQHMTERLSMSYAKEGKDPRHNEDTFSA

B9 codon-optimized sequence:
atggcgtcgccagccagcacgccagccagtagctcgtcactggtgccaactatcgatattagcccattcttattaaacccaacgagcgatgcagcaaacgacgtatgccag
gcgatccgggcggcctgtctgcatacgggctttttcaggtgatcggccacgccgtgagcaccagcacgctgactggtggctttgaagcatcacggcgtttttttactttaccact
ggaacaaaaacagaaattagacattaccaaacaactgggttttcggggctatgacggccttggcactcagtcatacagcggtgacacgcttccagacctgaaagaatcgtt
ctttctgggccgtgatataccaccaagccacccagactatggtcgcatactgacgggcccaaacatttggcctagcttcgcggtgctgccggcaacgacgtttaaagaacca
gtggaagcactttattcagcactgatgcaactggcatgcaaaattctggaaattctggcccgcacccttccacacggtgcaggcgaaggcatttttgatcggttcgtgcgcgac
ccggccgccccaatgcggctgcttcattacccaccaatggatgtgattgatgaacgccagctgggtgcctcagcgcataccgattttggcgcaattactctgcttctgcaagac
caggtcccgggtttacaggtgcacgattcagacaccggtgcctggctggatgtgccaccatgtcaggactcgattgtggtgaatgtcggtgacatgatcactcggtggactgc
gggcgaatacaaatcgagcgtgcaccgtgtcattaaccggtcaggtacccaccggtattcaatcgcgttcttctttgacggcaatgtggattgtctgttagacccgttagacggt
accactgccccgggtggttcaatcactgtggaaaaacacatgatcgaatgtatacgttcatcatacgcgtcgaaataa

B9 protein sequence:
MASPASTPASSSSLVPTIDISPFLLNPTSDAANDVCQAIRAACLHTGFFQVIGHAVSTSTLTGGFEASRRFFTLPLEQKQKLD
ITKQLGFRGYDGLGTQSYSGDTLPDLKESFFLGRDIPPSHPDYGRILTGPNIWPSFAVLPATTFKEPVEALYSALMQLACKIL
EILARTLPHGAGEGIFDRFVRDPAAPMRLLHYPPMDVIDERQLGASAHTDFGAITLLLQDQVPGLQVHDSDTGAWLDVPPC
QDSIVVNVGDMITRWTAGEYKSSVHRVINRSGTHRYSIAFFFDGNVDCLLDPLDGTTAPGGSITVEKHMIECIRSSYASK

C1 codon-optimized sequence:
atggttaaaggtaacttctgtggtgtgccaacggtggacatctcaccatatctggaagatccagggagcaacgccgcccgcaaagtgatcgaagatgttcgcgaagcgtgc
atctcaaccggctttttccaaatcacgggtcatggcattccaaaaaatctgcaacagaatgtgtttgatgcggcgcatgcctttttcgccttaccattagaagagaaaaaaaaa
ctgaacgccgcgaactttatcggtcatcgcgggtatgatgttttagccagccaaagctatgaggaaggtgtattaccagatttaaaagagggttattatgtaggcagcgatgtg
ctgccatcagatccattatatggccgtttttttatgggccgtaacgtgtggccatcaacgaaatttttaagcacggcgcaatttcaagaaccatgcgagcgttatcacgaatcggt
gcagcaactggcctttaaagtgttacaattagtgggtgatacgatgatcccacgcggtcatagcagcatcggtatgacggataatatgccaacggtgctgcatgatttaattcg
tttagataacatcccggcgtgcccgctgcgcctgctgcactatccatcggccgtgcgtaaccgcggggatgttgccggtaaaccgcaatatggtgcgtcagcgcatacggatt
ttggtgtaattacgttactgctgcaagatgataatccagggctggaagttctggtagaaatcgatggtaaacaagtttggtggccagtggatccgaatccaggcgcctatgtggt
gaatatcggggatatgacgagcatggtgacgaatggcacgtataaaagcagcatgcatcgtgtggtttgcaaacaaccagaacgcgagcgttatagcatcgtatttttttctgg
atggttgtttaaatgcgaaattaaaaccaatcgagggcttcgggcatccggaagaagatattgaaacgtcgcacaaaaccgtagagcaacacatgacggaacgtttatca
atgagctatgcgaaagagggtaaagatccgcgtcacaacgaggatgcctttaacgcgtaa

C1 protein sequence:
MVKGNFCGVPTVDISPYLEDPGSNAARKVIEDVREACISTGFFQITGHGIPKNLQQNVFDAAHAFFALPLEEKKKLNAANFI
GHRGYDVLASQSYEEGVLPDLKEGYYVGSDVLPSDPLYGRFFMGRNVWPSTKFLSTAQFQEPCERYHESVQQLAFKVLQ
LVGDTMIPRGHSSIGMTDNMPTVLHDLIRLDNIPACPLRLLHYPSAVRNRGDVAGKPQYGASAHTDFGVITLLLQDDNPGL
EVLVEIDGKQVWWPVDPNPGAYVVNIGDMTSMVTNGTYKSSMHRVVCKQPERERYSIVFFLDGCLNAKLKPIEGFGHPEE
DIETSHKTVEQHMTERLSMSYAKEGKDPRHNEDAFNA

C2 codon-optimized sequence:
atgcatagcagcttttatctgccactggtggacattacgccatttctggatgatccaagcagcccagcggcccagcaggtgattgatgacgtgcgtcgggcctgccgttcgac
cggctttttcagatgaaaggtcatggcgttccactgaaactgcaaaaaagcattctggccgccgccgccaaatttttgccctgccgaccgaaacgaaactggcgctggatg

cgcgcaaaaatgtggggtttcgcgggtatgatgtaatggaaacgcagagctatgaactggaaattggcgtaccacagggccaggaagcccaggtggtgcgcgatattaa
agaaggctttttattagcagcgatctgccattagatcatccgcgtgttcgcgacggccgtttttacaggggccaaacgtgtggccggataaagaagttctggccccgcagga
ttttcgtgtcgttctggaagaatatctgagcgaaatgcagcgtctgtcacaggttgttctgtcactggttgccgcgacgctgccgtatggcccgcgtgttttcgatgaactggactc
agatgatccaatgtgtctgctgcgtctgctgcattacccgccggatggggaacaggcccggcgtgaacgggggcagctggggagcggtgaacatacggactttggtaccc
tgaccctgctgctgcaagacgaacatgcggggctggaagttcaggaccatgaaaccggcgaatggcatggtgttccaccgcaggaagatgtttatatcgtgaacatggcg
gatctgatggaaattatgaccagcggggaataccggtcgtcgtggcatcgtgtcctgaatcgtaacccggaagaccgctattcggttgtctttttctatgacggcaacctggact
acaaattacagccgctggacccggcgaaaaaagtcgaagaagaaaatgttccaacgatcgaagagcatgtgcgtctgcgccttaccagtagctatagcatcgcgcaga
aataa

C2 protein sequence:
MHSSFYLPLVDITPFLDDPSSPAAQQVIDDVRRACRSTGFFQMKGHGVPLKLQKSILAAAAKFFALPTETKLALDARKNVGF
RGYDVMETQSYELEIGVPQGQEAQVVRDIKEGFFISSDLPLDHPRVRDGRFLQGPNVWPDKEVLAPQDFRVVLEEYLSEM
QRLSQVVLSLVAATLPYGPRVFDELDSDDPMCLLRLLHYPPDGEQARRERGQLGSGEHTDFGTLTLLLQDEHAGLEVQD
HETGEWHGVPPQEDVYIVNMADLMEIMTSGEYRSSWHRVLNRNPEDRYSVVFFYDGNLDYKLQPLDPAKKVEEENVPTI
EEHVRLRLTSSYSIAQK

C3 codon-optimized sequence:
atgacgaaaggtagcttctcaggtgtgccaacggtggacatctcgccatatctggcggatccaaaaagcgatgccgcccagaaagtgatcgaagatgttcgtgaagcgtg
catctcaacggggtttttccagattacgggtcatggcattagcaaatcgctgcaacaaaacatttttgatgcggcgcatagcttcttcgcgttaccattagaagagaaaaaaaa
actgaatgcggcgaactttatcggtcatcgtgggtatgatgttttagccagccaaagctatgaagaaggcgtattaccagatttaaaagagggttattatgtaggcagcgatgt
gctgccaagcgatccattatatggtcgtttttcatgggccagaacgtgtggccatcaacggaattactgagcattatccagtttcaagaaccatgcgagcgctatcacgaaag
cgtgcagcacctggcctttaaagtgctgcaattagtaggtgatacgatgatcccacacgggcatagcacggtgggtatgacggaaaatatgccaacggtgctgcacaattta
attcgtttagataaaatcccggcgtgtccgctgcgcctgctgcactatccaagcgccacgcgcaacggtgggtcggttacgggcaaaccacaatatggtgcgtcagcgcac
acggatttcggtgtaattacgctgctgctgcaagatgataatccaggcctggaagtgctggtggaaaaagatggtaaacaagtttggtggccaatcgatccgaatccaagcg
cctatgttgtgaatatcggtgatatgatcagcatggtgacgaacggcatctataaaagcagcatgcatcgcgtggtatgcaaacgtccagaacgtgagcgttatagcatcgta
tttttctggatggctgtttaaatgcgaatctggagccagtagagggtttcgggcacccagaaggcgtgggtttttcgcacaaaaccgtagaacaacacatgacggaacgttta
tcaatgagctatgcgaaagagggtaaagatccacgtcataacgaggatacgttttcggcgtaa

C3 protein sequence:
MTKGSFSGVPTVDISPYLADPKSDAAQKVIEDVREACISTGFFQITGHGISKSLQQNIFDAAHSFFALPLEEKKKLNAANFIG
HRGYDVLASQSYEEGVLPDLKEGYYVGSDVLPSDPLYGRFFMGQNVWPSTELLSIIQFQEPCERYHESVQHLAFKVLQLV
GDTMIPHGHSTVGMTENMPTVLHNLIRLDKIPACPLRLLHYPSATRNGGSVTGKPQYGASAHTDFGVITLLLQDDNPGLEV
LVEKDGKQVWWPIDPNPSAYVVNIGDMISMVTNGIYKSSMHRVVCKRPERERYSIVFFLDGCLNANLEPVEGFGHPEGVG
FSHKTVEQHMTERLSMSYAKEGKDPRHNEDTFSA

C4 codon-optimized sequence:
atgagcccaaacgtgattgtggatgaaaacattaccagcaaaaccaccacggtggatagctttcatattccaacggttgatatttcagcgtttctggcggatccggattcacca
gaagcgcagagcattattccaattgttcggagcgcgtgccgctcaaccggtttttttcagattaccggtcatggcatcagcaaagatctgcaacaggcggtttttgcgggcgcg
aaacagtttttttagcctgccatttgaaacgaaaaaagcgctggatgcgcatttaaatcagggcctgcggggttatgatgtattaagctcacagagctatgaagatgggattctg
ccggatctgaaagaaggctttttaccggtgttgatctgccggcggatgatcctcgtgttcaggcgcgcaaatttctgatgggtagcaatgtgtggccagatgcgagcctgctgc
cagaagatgaatttcgcaaaccaattaaagaatatcatgatgttctgatgaaactgagcattaaactgctgaaactgatcgaacgcaccctgccatacggtccgggtatttttg
atgaatttattggcaacgatccaattatgccgatgcgcatgctgcattatccgccggcgcgcccagaacatgaacggaacggtaaaacccagtatggtgcgagcgcgcata
ccgattttggcgcgatcacgctgctgctgcaagaaaaaaatccagggctggaagtttttgattcacgtaccgatgaatggaaaccaatcccgccgaatccggatgtttatgttg
tgaatatcggcgatatgctgtcgttttggaccaaaaacgaatataaaagctcggttcatcgcgttattaatcgcgaaccaggcgatcggtattcgattgttttttttgttgatgggaat
ggggatgcgaaactgtcaccgctggatgggtcagaagaagataatccgctgacggtggaacagcatctgatccgtcggctggcggaaagctatggcaaaaaataa

C4 protein sequence:
MSPNVIVDENITSKTTTVDSFHIPTVDISAFLADPDSPEAQSIIPIVRSACRSTGFFQITGHGISKDLQQAVFAGAKQFFSLPFE
TKKALDAHLNQGLRGYDVLSSQSYEDGILPDLKEGFFTGVDLPADDPRVQARKFLMGSNVWPDASLLPEDEFRKPIKEYH
DVLMKLSIKLLKLIERTLPYGPGIFDEFIGNDPIMPMRMLHYPPARPEHERNGKTQYGASAHTDFGAITLLLQEKNPGLEVFD
SRTDEWKPIPPNPDVYVVNIGDMLSFWTKNEYKSSVHRVINREPGDRYSIVFFVDGNGDAKLSPLDGSEEDNPLTVEQHLI
RRLAESYGKK

C5 codon-optimized sequence:
atgccgcccttacagtacacctggaaaattctccgcggcgcggcccttcagaacgctttgtcaccgtctagtacgaccagccagctgcgtgtgacgcggccgattgctgcatt
taacccttttagcctagccacgtcacgcaccttcaaagcacctgctcgccgtcgtcaatcattcgcggcaacggaaacgacatctaccgcgaccccgtcatcgagtgtgccg
ccgaccctgcccacgccgcgcgctgcgaccaccaccccacctagcttcaccattccgactgtagacatcgcccccctatttgagcaatccaagctcgccggcagctcagcg
cgtaattgagcagattaaccaagcatgccgtagcacgggcttcttccaaatagttaatcatggtgtccctgcggccctgcaatcagaggtgtttagagctgccgaacgcttcttt
cgcttaccgctggaagaaaagaagaaactggatgcgaaaaccaccgtgggtcatcgtggctacgatgtgctggaatcccagagctatgaagaaggtgtgttgcctgactt
aaaagaaggcttttatgtgggtaacgacattccagcgagcgacagccgtgcccaagcgcgccgcttctttatgggcccgaatgtgtggccgtcgtcactgcaacctcatgaa

ctgcaacaaccggcagaagcgtatttcgcagcagtgtataccctgtcattacgcatacttgacatgattgcggcatcactcccgtacgatgtctcgcctatgttcgcacgtttcac
ccagccagctgacctggttgccgcgccactacgtctgcttcactatccgcctgcccgacgctctaaacagaggcagctgggggcgtctgcgcatactgatttggcgctatca
ccctgctgctacaggatggtaatccgggcctgcaagtgcttgatcagcatagcgaaagctgggtggacgtgccctcgacgcgtaatacgtatgtcgtgaatgttggtgacatg
cttgagaaatggacgggtggctactaccgcagtagcgttcatcgtgtgctgaatccgcagccaaaagatcgctattcagtcgttttcttctttgatggtaacttggactgtccctta
gttccgctcaatccgaatgatgccgcgaaatcgtcagccctgccgggtggcgagggcggtaaagaagatggtgtgctgactgtcgagaaacacatgattaaccgcatgac
aactagctatggtaagggctcgaagaagtaa

C5 protein sequence:
MPPLQYTWKILRGAALQNALSPSSTTSQLRVTRPIAAFNPFSLATSRTFKAPARRRQSFAATETTSTATPSSSVPPTLPTPR
AATTTPPSFTIPTVDIAPYLSNPSSPAAQRVIEQINQACRSTGFFQIVNHGVPAALQSEVFRAAERFFRLPLEEKKKLDAKTT
VGHRGYDVLESQSYEEGVLPDLKEGFYVGNDIPASDSRAQARRFFMGPNVWPSSLQPHELQQPAEAYFAAVYTLSLRILD
MIAASLPYDVSPMFARFTQPADLVAAPLRLLHYPPARRSKQRQLGASAHTDFGAITLLLQDGNPGLQVLDQHSESWVDVP
STRNTYVVNVGDMLEKWTGGYYRSSVHRVLNPQPKDRYSVVFFFDGNLDCPLVPLNPNDAAKSSALPGGEGGKEDGVL
TVEKHMINRMTTSYGKGSKK

C6 codon-optimized sequence:
atgcctagcgcgatcgaacaaccacagtttcacattccgacggtggacatcggtccatatttggctgacccgtctagtgcggaagcacatcatgttgtcgatgcggttcgcctg
gcgtgtacgacttcgggcttcttccaattaacgggtcatggcattgagccggagattcgcgatgcaatgtttagcggttcgaaagccttattcgatctgccgttcgatcagaaga
aagcgctgcggcgcgggaagaaccgcggttatgaagtcattggctcgcaggccttacaagatgggaccttgcctgatctgaaagaagggtattatatcggtgaagatacc
acagattttgaagaggggaaaacctaccgcccgtttatggatcctaacgtttggccgtcagaggatcaggtcccgagtagtgtgtttcggaaaccgatgaatcaatattatga
acgtgtagctgcgctcagcaaaaccgtaatggacattattgcggcagcgctcccgtatgggcccacggtgtttgatcaatacaagctggaaccaattgccgcgagcatccg
cctgctgcattatccgccgcagcgaaccaacgacggtaaacagctgggtgccggcgcgcataccgacttcggctggatcactctgctgctcaccgacggacatccaggcc
tggaagtgttaaatcagagcaccggcgaatgggtacaagtcccgccgaacaaggatgcctacattgtgaatgtcggcgatatgctgcaacagattaccgggggctatttta
aatcgaactttcatcgtgttttgaacttaggtgaatcagatcgttatagcgttccgtacttctttgatggttgcctcgacgcgaaactcgcgcgccttgacggtcgcgacgaaggta
aagtactgacagtggaagaacacatgctggagcgttttgcgacgacttatggtcgcggccaggcaaaagattaa

C6 protein sequence:
MPSAIEQPQFHIPTVDIGPYLADPSSAEAHHVVDAVRLACTTSGFFQLTGHGIEPEIRDAMFSGSKALFDLPFDQKKALRRG
KNRGYEVIGSQALQDGTLPDLKEGYYIGEDTTDFEEGKTYRPFMDPNVWPSEDQVPSSVFRKPMNQYYERVAALSKTVM
DIIAAALPYGPTVFDQYKLEPIAASIRLLHYPPQRTNDGKQLGAGAHTDFGWITLLLTDGHPGLEVLNQSTGEWVQVPPNKD
AYIVNVGDMLQQITGGYFKSNFHRVLNLGESDRYSVPYFFDGCLDAKLARLDGRDEGKVLTVEEHMLERFATTYGRGQAK
D

C7 codon-optimized sequence:
atgagtccaagcaagaccccgagctttaccattccgacggtagacattagcagctatgtcgccgacccgacctcgcccgctgcgaaatccgttattgaggatgttagaaac
gcatgcatcactagcggtttctttcagctggtgggtcatggagttccacgcgaactgcaagaagccgtgctgaacggcgctaaagcattctttaatttaccgcaagacgaaaa
gctgaagtatgagaaaagcacagtcggcaaagggaatcgtggctacgagatcatggggaaacagatcttacagaaaggagcgctgccagatctaaaagaaggtttcta
tgtgggcgaagattttgagccaagcccggatcccaaccataagtatagctggttcaatccgcccaatgtgtggcccacgtcgttggctgacagcgaaatcgtatttcgcaaac
ctatcaacgaatactattcccacatgttcccattaagcacgacgattctggacattatggccgcaggtatgccgtatggtccttccgttttcgatgagttcaaaagcgatccagtg
gtagcagtgatgcgcctgttacattatccaccgcagcctgaatcttctgacgccttgcagttcggtgttggcgcacataccgatttggctctatcacactgctcctacaggatatg
gctggcggtttagaagtgttgaaccacgacacgaacgaatgggtccctgttaccccctaatccggacgcgtatgtagtgaacctcggtgacatgatgcagcgttggacgcgc
ggtgcttaccgctctaacttgcatcgtgtaaagaatatgggtgggggggatcgctacagcgtaccgttcttcttcgatggcaacatgaaaacaaagctcgccccgttcgacgg
cggagaaaccgatgaggaagtaattaccgccgaagatcacatgatggaacgatttagaaccaccgcaggcttttaa

C7 protein sequence:
MSPSKTPSFTIPTVDISSYVADPTSPAAKSVIEDVRNACITSGFFQLVGHGVPRELQEAVLNGAKAFFNLPQDEKLKYEKST
VGKGNRGYEIMGKQILQKGALPDLKEGFYVGEDFEPSPDPNHKYSWFNPPNVWPTSLADSEIVFRKPINEYYSHMFPLST
TILDIMAAGMPYGPSVFDEFKSDPVVAVMRLLHYPPQPESSDALQFGVGAHTDFGSITLLLQDMAGGLEVLNHDTNEWVP
VTPNPDAYVVNLGDMMQRWTRGAYRSNLHRVKNMGGGDRYSVPFFFDGNMKTKLAPFDGGETDEEVITAEDHMMERF
RTTAGF

C8 codon-optimized sequence:
atgcctagcttcacggaaaattcgggctcgaccagctttcagattccgacaattgatatttccccgtttattaccagcccagcatctcttaaagcgggcaaagtaattcaagcca
tccaactggcatgtcgctccacgggcttctttcagattacaggtcacggcgtgccggaatcggtgcaggaacaagctctggatgcctcgaaacgcttcttcagcatgccgcttg
aggacaaagaaactttgaacattaaacttaatccgggctttcgcggctacgatccgttgtgcacgcagacttatgggccggatgccctgcccgatctgaaagaaagcttcttc
gttggccacgacatcccgctcgacgatgtgcgggtgaaagcccgcaggctcctgaccggccccaatgtctggccgagcgggctgccagcgtccagcttcaaagattccat
cgaaacgtattataatgcgcttcatgatttgacgctacgtattttagaaatgattgcccttggtctgccgtatggggccgacgtatttagtaatttcatctcaaacagcccggcggct
cctatgcgtctgttgcactacccaccgcaggaaatcgacgatgacaaacgtcagctgggtgcatccgctcacactgacttcggcgcaattactctcttgctccaggataagaa
cgaaggactccaagtgcatgatgttcaaacggattcctggatgactgtaccgccgcgcaaggatgcgtttgtcgttaatgtaggggacatgttagaaaagtggacggcggg

cgatcttaaaagttctcttcatcgcgtcctcaaccaggggggcgcgaccgctactcgatcgccttcttcttcgatgggaacctggactgcacactcagcccgctggatggga
cgcgcgtcgaaggcgaaccgctgactgttgagaaacacatgattcaatgtctatctgctagctataaggaaacggtcgagtaa

C8 protein sequence:
MPSFTENSGSTSFQIPTIDISPFITSPASLKAGKVIQAIQLACRSTGFFQITGHGVPESVQEQALDASKRFFSMPLEDKETLNI
KLNPGFRGYDPLCTQTYGPDALPDLKESFFVGHDIPLDDVRVKARRLLTGPNVWPSGLPASSFKDSIETYYNALHDLTLRIL
EMIALGLPYGADVFSNFISNSPAAPMRLLHYPPQEIDDDKRQLGASAHTDFGAITLLLQDKNEGLQVHDVQTDSWMTVPPR
KDAFVVNVGDMLEKWTAGDLKSSLHRVLNQGGRDRYSIAFFFDGNLDCTLSPLDGTRVEGEPLTVEKHMIQCLSASYKET
VE

C9 codon-optimized sequence:
atggcaagcagcggcttttacataccatctgttgatattgcgcctttcattcacgatccttccagtgacgccgcgaaagacgtgatcgaaaaggtcaagaaagcgtgcgtcag
caccggcttctttcagattttcggtcatggagtgccgaagcaggtacagaaatccgcatttgatgcatccgcggcgttctttagcctgccggtcgatacgaagatggcgctgaa
ggcaggccctagcactggctttaagggttatgaacagctggagagccaatcttacagctatgatgttaaaggagatattaaagaagggttcaccatttccaccgattatcctg
acggacatccactccgcacccctctgaatggccgccctcgttttctgacagctccgaatatttggcccaccaccgaaatgatgggcgatgcagagtggttccatgcttctttgg
aagcctaccaagaagcaatggttaccctgcgagcgcatgttttatctcttattgccgcgaccttaccgtacggcccgcatgtctttgatgaaatgaatcaggagccggcatgcc
cgctgcgttatctccactacccgccgatgtctgaagggaccgatattgtgaaacagcagggagcttccgcacacactgattttagcgcggtcactctgctgctccagggtcaa
caggacggccttgaggtgctggattacgacacgggtgaatggcatttggtaccgccgaacccggatgcctacgtaattaatcttggcgatatgatggcgaaattactgcgcg
gacagtataaaagtagcaaacaccgcgtcattaatcgcgcgaaagtggatcgttactcaattgtttatttctgggacggcaaccgcgattttgaactgaaaccgctggatggc
gcagaaatggatcaaaagcacatgacgtgtgaagaatacatgttcgatagattgaaattcagctttgggcgtcacgaaaccaaaccagaagtgcaggcgcagtattaa

C9 protein sequence:
MASSGFYIPSVDIAPFIHDPSSDAAKDVIEKVKKACVSTGFFQIFGHGVPKQVQKSAFDASAAFFSLPVDTKMALKAGPSTG
FKGYEQLESQSYSYDVKGDIKEGFTISTDYPDGHPLRTPLNGRPRFLTAPNIWPTTEMMGDAEWFHASLEAYQEAMVTLR
AHVLSLIAATLPYGPHVFDEMNQEPACPLRYLHYPPMSEGTDIVKQQGASAHTDFSAVTLLLQGQQDGLEVLDYDTGEWH
LVPPNPDAYVINLGDMMAKLLRGQYKSSKHRVINRAKVDRYSIVYFWDGNRDFELKPLDGAEMDQKHMTCEEYMFDRLK
FSFGRHETKPEVQAQY

D1 codon-optimized sequence:
atggcgaattttcatatcccgaccatagacattagcccatatctccaggatgccacgtcagatgcggcagaccaggtggtgcgtgatgtgggtaaagcgtgtgccaccgtgg
ggttctttagcttggttggtcatggcatcccccgaaacatccaagacaacgtgttcgaagcggcgaagaagttgtttaacttacccgccgaggagaagcgtgcgctgagaca
tccactgatgaagaatcgtggatatgagctaattgggagccaggcactccaggataacacgctgcccgatctgaaagaagggttttacattggcaagcatatcccagcgga
atcggaacggtcgcgcgcccatcccgaactgctgggagagaatatctttccggcctcacttcacgataccgtgtttaagaagcccaccgaagcctattataccgcggtcttgg
agctgtccttaaaagtcttagaaattctggcaaagggccttccttatggtgatgacattttcaaggaatttgtctctaacgatccagtttgtatcatgcgcctcttgcattacccgcca
caggcatcacaggacgaacgtcagctcggcgcgggcgcacataccgattttggtgcaatcactctgctgttacaggacacttccggcggtctggaagtcctggatcataata
caggaacgtggcacgcggtagctcctaacccagacgcgtacgtggtgaatataggtgatatgctggccctgtggactaaaggaatttacaaaagcgccgttcatagagttat
taataagtccggtggagatagatattccgtgccgttcttcttcgatggcaacgcggaagtaaagcttaagccctttgatggcagcgaaccagtcggcggcaaaattatcaccg
ttgaagatcacatgttagaacgtcttggcacttcgtatgcacgtgtcgaacaaggtgctggccaagcggtcagcgccagtgcgtaa

D1 protein sequence:
MANFHIPTIDISPYLQDATSDAADQVVRDVGKACATVGFFSLVGHGIPRNIQDNVFEAAKKLFNLPAEEKRALRHPLMKNRG
YELIGSQALQDNTLPDLKEGFYIGKHIPAESERSRAHPELLGENIFPASLHDTVFKKPTEAYYTAVLELSLKVLEILAKGLPYG
DDIFKEFVSNDPVCIMRLLHYPPQASQDERQLGAGAHTDFGAITLLLQDTSGGLEVLDHNTGTWHAVAPNPDAYVVNIGD
MLALWTKGIYKSAVHRVINKSGGDRYSVPFFFDGNAEVKLKPFDGSEPVGGKIITVEDHMLERLGTSYARVEQGAGQAVS
ASA

D2 codon-optimized sequence:
atggccgtcccgctgatgcatcagacaatgcacagccaggtgcatgaagccagcgatgcatcacacgaaatcccgaccattgacatctccggctttcgttccagcccgga
aactgcgcaggaacgtatcgagactcaacatgtggttgaccgggtgcgtagtgcgtgcctgacgaccgggttcttctgtctcacgggccatggcattccgcgcgaattgcag
gagcgtgttcttgatggtgcagagaaattcttcgacatgccgcttgaagaaaagctgaaacttgttcacccgactcagaagaaccgcggttatgaggtgcttggtggccaaac
tctgcaacgtggtgcaaaaccggatctgaaagaaggcttttacatcggcaaacacctggatggtaacgacccccgagtgcggcgccacccgcgcctgatgggcccaaat
atttcccgagcggcagtcttgaggatcatgaatttaagaacccactggaaacctattatgaagctatcttagcgttgtctctgacggttttcaaaatactggcgaaaggcctgcc
atacggggatgatgtgtttgaccgattcgtgagcgatgatccggtctgcattctcagactgctgcattatccgccgcagaccagtaaagatcctcgccagctgggcgccggcg
cgcatacagattttggcgcggttaccttgctataccaaacggcaccaggtttacaggtgaagtctccgagtggctcaggctcaagcggtgccgatcgttggattccggtgccg
ccaaaccgtgacgcgtacgttgtcaatataggcgacatgctgcacgcgtggacagaaggactctataaatcctcgatgcaccgcgtgctaaatctgggtggcagtcaccgtt
atagcattcctttcttcttcgatggtaacgcagactgtccgctgactccgttcggaaaatcagacgacgttggcgctgagactgccagcgagcatttactgcgtcgcctcgacg
aaacgtatgcaatggcagccaaaatctaa

D2 protein sequence:

MAVPLMHQTMHSQVHEASDASHEIPTIDISGFRSSPETAQERIETQHVVDRVRSACLTTGFFCLTGHGIPRELQERVLDGA
EKFFDMPLEEKLKLVHPTQKNRGYEVLGGQTLQRGAKPDLKEGFYIGKHLDGNDPRVRRHPRLMGPNIFPSGSLEDHEFK
NPLETYYEAILALSLTVFKILAKGLPYGDDVFDRFVSDDPVCILRLLHYPPQTSKDPRQLGAGAHTDFGAVTLLYQTAPGLQ
VKSPSGSGSSGADRWIPVPPNRDAYVVNIGDMLHAWTEGLYKSSMHRVLNLGGSHRYSIPFFFDGNADCPLTPFGKSDD
VGAETASEHLLRRLDETYAMAAKI

D3 codon-optimized sequence:
atgattcccacggtggatatttccgccttcaacgacgatagtgctaccgcggatagcaagaacgaagtggtgcaggctgtggcgcacacctgctctacgtatgggttcttctat
ctggtgggccatggtattccggaagcggatcgtcagcagattttggattgtgcccgcttattttcatccctgcccatggatgagaaaatgatcatctcggtgagcaagcgcatgg
ggcagtcgtttcgtggttatgaacccccagccctgcaattgcaccaggaaggtctacttcctgatacgaaagaggctttcattttcggtcgcgaggttagcgcggaccatccgg
atgccggccgtttcagcaccggcccgaaccagtggccgccgtcactgccggattcgacgttttgtatcccactgctggaataccagcgtaaaatggtgcagctggtgaaaac
cattctgaagattctggcctcgggcctgccggaagaatggaattgtccatgcgatgtgtttgatgccgctaccgtcgaaccaagcatccctatgcgcttactccattatgcgcctc
agtcagttaagagcgaaaaccaatttggcgttggcgatcacaccgattttggcaacgtgtccattcttctccaggaagaaggcacggaaggcttagaagtgtggtatccgttg
acagaggattggatcccagtgcccgttattccgggttcctacgtaatcaatatcgcggacatgatgcaaaagtggacggcgggcttctaccgtagcgctcgtcaccgagttgt
aaaccataataaacgtccgagatatagtgcagcgttctttcccaacggcaatattgacctgaagtaccgtgcgctggatggaagtggtgtcgagaaaacgattggcaaaca
catacgccagcgcctgtttgagacaataggtggcgaggcgggtaagaagctggagaacgacgccgtcaaacgctaa

D3 protein sequence:
MIPTVDISAFNDDSATADSKNEVVQAVAHTCSTYGFFYLVGHGIPEADRQQILDCARLFSSLPMDEKMIISVSKRMGQSFR
GYEPPALQLHQEGLLPDTKEAFIFGREVSADHPDAGRFSTGPNQWPPSLPDSTFCIPLLEYQRKMVQLVKTILKILASGLPE
EWNCPCDVFDAATVEPSIPMRLLHYAPQSVKSENQFGVGDHTDFGNVSILLQEEGTEGLEVWYPLTEDWIPVPVIPGSYVI
NIADMMQKWTAGFYRSARHRVVNHNKRPRYSAAFFPNGNIDLKYRALDGSGVEKTIGKHIRQRLFETIGGEAGKKLENDA
VKR

D4 codon-optimized sequence:
atgaaagaacaggtgaccccgggcatttaccaatccttggccagtctgtacttcttcagcattacgagcatccccattgttgacatatcgccgtttctcgatgaagaagtgtcgc
aggaaatgatgaaccaggtggtggaagccatgcgtgaggcttgtaccacgtgtgggttcttctatttactgggccatggcatattcgaggaagattgtaagagtgccatgggc
aaatcctttcgcggctacgaaccgcctgcgctgcaaatgcatcaggaagggctcctgcctgataccaaagaaggttttatcctgggttgcgaaattccggcagatgcgcccg
aagcgggcacgttccatactggcccgaatcaatggccggagagcattcctatgatggaataccatagcaaaatgatagaaatggtgaaggttgtggtcaaaattctggcgc
tgggtctgcctgatcagtggaactgctcgccggatgtattcgatgagatgacgcataatccgtccgcgcctctgcgtttgctgcactatgccagccaaccgctaaaatgcgaa
aatcagtatggtgtaggcgatcataccgattttgggaatgtgagtgttctgctccaggaagaaggacgtgatggtctggaagcgttttacccgccgacgcatgtgtgggtacca
gtccctgcgttggaacactcctatgttattaacatcggcaacatgatgcagaaatggaccggtggatattataaaagtgcccgtcaccgagtcatcaatcacaagactaaaa
gccaatatatcactccgttttctatgaacggtaacttaggcttaaaatgtcgcgcactggacggatccggcacggagactgtgattggtgatcatattcggcagcgcttggttga
aaccatcggtggtgaagctgggaagaaactgggttactaa

D4 protein sequence:
MKEQVTPGIYQSLASLYFFSITSIPIVDISPFLDEEVSQEMMNQVVEAMREACTTCGFFYLLGHGIFEEDCKSAMGKSFRGY
EPPALQMHQEGLLPDTKEGFILGCEIPADAPEAGTFHTGPNQWPESIPMMEYHSKMIEMVKVVVKILALGLPDQWNCSPD
VFDEMTHNPSAPLRLLHYASQPLKCENQYGVGDHTDFGNVSVLLQEEGRDGLEAFYPPTHVWVPVPALEHSYVINIGNM
MQKWTGGYYKSARHRVINHKTKSQYITPFSMNGNLGLKCRALDGSGTETVIGDHIRQRLVETIGGEAGKKLGY

D5 codon-optimized sequence:
atgggctctatcgaagccattcctacggtggacatctctgcgtggatcgatacatcctctactgaagaagaacgccagcgtgccgtggatgaaatgcgaaatgcttgcaccc
gctacggcttctttaatttaattgcccacggcgtcagtccccaggcgcaatcacaagcgttaaattgtgctaagttattctttaccttgtccgaacaggaaaagatgaaggtctgt
attagtaaatcggttggccgtagctttcgtgggtacgagccccctggcattcaagtacatcaagaaggcttactgccggatactaaagagtgtttcattatcggacacgaggtg
ccagagcatgatccggaatgcggcacattttcgactgggccgaatttatggcccaaatctctgcccgaagatgaatttaggatccctatcatggaatatcaggcccgcatggt
agccctggtgaaagtgcttttgaagatcctggcccgcgggctgccgaaggaatggaactgttctccagatgtactggatccgctcggcgagaataacccgagcattccgatg
cgtttgttgcattacggcccgcaaccaattcgtgatgaccgccagtttggcgttggcgatcacacagattttggatgtgtgaccatcttgttacaagacatgggtactgaaggcct
ggaagtgtggtatcccccaacggaaacctggatcccggttcccccacaagaaaatgcgtatgtaattaatgtgcgcgatatgttgcagaagtttacgggggggttactaccgta
gcgcccggcatcgtgtggtgacattcacggccaatcatcgctacagtgtccccttcttcttgaacggacaactcaaactcaaatgtaaaacgctggatggaagcggcaccg
aaaccattgtaggcgaacacatccgtgggcgcttgttggagacaatgggaaaggcgggggaaaagttaaagtaa

D5 protein sequence:
MGSIEAIPTVDISAWIDTSSTEEERQRAVDEMRNACTRYGFFNLIAHGVSPQAQSQALNCAKLFFTLSEQEKMKVCISKSVG
RSFRGYEPPGIQVHQEGLLPDTKECFIIGHEVPEHDPECGTFSTGPNLWPKSLPEDEFRIPIMEYQARMVALVKVLLKILAR
GLPKEWNCSPDVLDPLGENNPSIPMRLLHYGPQPIRDDRQFGVGDHTDFGCVTILLQDMGTEGLEVWYPPTETWIPVPPQ
ENAYVINVRDMLQKFTGGYYRSARHRVVTFTANHRYSVPFFLNGQLKLKCKTLDGSGTETIVGEHIRGRLLETMGKAGEKL
K

D6 codon-optimized sequence:

atggctgaggtgcctcagcagatcccgacgattgacatcagcgcgtgggtgaatccggcgtcgtccgccgcagataaacagcgggtcgttgatgaagtccaccatgccgc
gacgacttacgggttcttccagtgtgccgggcacggcattacacccgaaacccgtgaacagattttcaacctgagcaagcggttcttctcacttccaatggaagagcggatg
gaaatctccgtgaagaagagcttgggccagtctttccgcggctacgagccgccctttctccagaaccaccataaacatttgctcccggataccaaggaaacctttgtggtgg
gcgcggaaatcccggcggaccacccggacgctggcacctttctgaatggtccaaatttgtggcctacgagcattcctgaggacgagtttcgtaaaccgattatttcatatcag
agccaaatggttgcgttaggaaatacgttgttaaagattctggggcacggcttgccgaagagttggaattgcagccctgatgttttcgattcattagccgaaaacccggccattc
cgatgcgcatgctgcattatgcggcccagccggtacatgacgagaaacaattcggtgtcggtgctcatacagattttggcagcgttaccatactactgcaacagctgggcac
caaaggtctggaagtgtggtatccaccaacggaaacgtggattccggttccgccaaaagaaaataccttcgtgattaatatcggtgattttgtagaccggtatacgaacggtt
attaccggtctgcgcgccatcgcgtaattaccaccgataaggaacggtattcggtggcgttcttctttaatgggaacatgaagcttaaagccccagttttggacggctcaggag
atgaagtgaacttaggtgatcatatacgccagcgtgcgattgccacttatggtgcagctcctactctgatcgagagcaaagtgtaa

D6 protein sequence:
MAEVPQQIPTIDISAWVNPASSAADKQRVVDEVHHAATTYGFFQCAGHGITPETREQIFNLSKRFFSLPMEERMEISVKKSL
GQSFRGYEPPFLQNHHKHLLPDTKETFVVGAEIPADHPDAGTFLNGPNLWPTSIPEDEFRKPIISYQSQMVALGNTLLKILG
HGLPKSWNCSPDVFDSLAENPAIPMRMLHYAAQPVHDEKQFGVGAHTDFGSVTILLQQLGTKGLEVWYPPTETWIPVPPK
ENTFVINIGDFVDRYTNGYYRSARHRVITTDKERYSVAFFFNGNMKLKAPVLDGSGDEVNLGDHIRQRAIATYGAAPTLIES
KV

D7 codon-optimized sequence:
atgggatcgctcacggaaaccgccacaatccccacggtggacatctcagcatttcttgacccgaatgcttctgaccaggcgcgtcaggatattgttaaagctatgtcacatgc
ttgtcatgtctatggcttctttaatctggcaggtcatggcattccgcaagagacactgagaacagcgctggagcttaacaaaatgttcttcacgttaccggaagaaagcaagaa
ggaagtgcttatctctaagtcagtgggtcaaagtttccgtggatatgaacccccggggatccaaacccatcaccggggccttctgccggatattaaggaaacctttatggtgg
gccgcgaagtaccgctcgatgaccctgattgcggcacattctcacgtgatccgaatctctggccgaccagcttaccgaaagaaaagttccaggatcgtatcatggcctacca
gggcagcatgcttcaactggtgaaatccatcctggcaattctggcccagggtctgccgaaagaatgggaatgttctccgaccgtgttcgattcactgctggaaaagccctctat
cccgatgcgttttctgcattatgcaccagtgccgtcacagctggaagatgcacgtcagtttgaggtggcggaccacaccgactttggctgtgtttctattctgcttcaggaacccg
gaaccagtgggctggaagtgtattatcccccgtctgacagttggattccggtacccgtggttaatgatggctttgttatcaacatgggcgatatgatgcagcgttatacaggcgg
ttactatcgctcggcgcgtcatcgcgttctgaccaatagggagaaacatcgccacagcgtaactttcttccttaatgggaacctgagtcttaaagcgaaagctctggatgggtc
cgataccgaaactgtcgttggtgactggatccgcgggcgcctgttggaaacaatgggccagaccggtaaattgttgcagcgtgaatccagcaaaacccaataa

D7 protein sequence:
MGSLTETATIPTVDISAFLDPNASDQARQDIVKAMSHACHVYGFFNLAGHGIPQETLRTALELNKMFFTLPEESKKEVLISKS
VGQSFRGYEPPGIQTHHRGLLPDIKETFMVGREVPLDDPDCGTFSRDPNLWPTSLPKEKFQDRIMAYQGSMLQLVKSILAI
LAQGLPKEWECSPTVFDSLLEKPSIPMRFLHYAPVPSQLEDARQFEVADHTDFGCVSILLQEPGTSGLEVYYPPSDSWIPV
PVVNDGFVINMGDMMQRYTGGYYRSARHRVLTNREKHRHSVTFFLNGNLSLKAKALDGSDTETVVGDWIRGRLLETMGQ
TGKLLQRESSKTQ

D8 codon-optimized sequence:
atgaccgtgcagcaccagattccgaccatcgatattagcgcgttcctcgatcagaccgccagtgaaaaggcgcgggaagcagtggtggcagaggttcgcgatgcgtgtat
gacgtatggcttcttccagctgattggccatggcattcctctggctatccaggaacatgtccttcgctgtgccaaggtattcttcgaccagcccctagagaagaaacaggcact
gggcatggataaagccatgggtcagagtaatcgtggctacgaacggatcgggggccaagcgctccaggcagatatgctgcctgacctaaaagaaggtttctatattggcc
gcgaagtgccggccgacgatcctagagctggccagtttcaccaaggtccaaatctatggcccgaaattccggaagaagagttccgcgtacctctgtcggaatactatcgtat
gatgattcaattggattatcagctgctgaatatcatcgcattaggcctgccgttttccaacgacatctttgaagaatttaagaaggaaccggccgcgagtctgcgcttgttgcatta
ccccccctcagatcagtatcgatgagcgccagtttggtgcgggggcccacacggattttggttgtatgaccctgctgttacagcagccgggtcaggaaggcctgcaagttttcta
tccgccgaccaaagagtggattcctgttccagctgcggctaacacctttgtcgtcaacatcggtgacttgttacagctgtggacgaaaggtcagtatcgtagcgcgctccacc
gagtgttaaacgctactaacgaggatcgctactctgtgccgttcttctacaacggtaacctcgcatgtaaactggccccgctggacgggtccagtgatgtgaatacgctaaca
gtggaagaacacgtcaaacggaaatttcaaacatcttatgcggtgtaa

D8 protein sequence:
MTVQHQIPTIDISAFLDQTASEKAREAVVAEVRDACMTYGFFQLIGHGIPLAIQEHVLRCAKVFFDQPLEKKQALGMDKAMG
QSNRGYERIGGQALQADMLPDLKEGFYIGREVPADDPRAGQFHQGPNLWPEIPEEEFRVPLSEYYRMMIQLDYQLLNIIAL
GLPFSNDIFEEFKKEPAASLRLLHYPPQISIDERQFGAGAHTDFGCMTLLLQQPGQEGLQVFYPPTKEWIPVPAAANTFVV
NIGDLLQLWTKGQYRSALHRVLNATNEDRYSVPFFYNGNLACKLAPLDGSSDVNTLTVEEHVKRKFQTSYAV

D9 codon-optimized sequence:
Atgagcagcgagaaatcggaaggtgggattccaactgtggatattggcgccatccttagcccggccgcgaatgaagccgatcgtcgccaggttgtggaagccgtacgttat
gccagcactaccttcgggttcttcaacttggttggccacggcattcctcgcgctcagttgcgccagattttcgaatcttctaaactgttcttcgcactgccggaagagaaacgtcgt
gatgtgcatgttggtcaggcattaggtcgcagtttccgtggttgggaaccgccgctgatccaacagcatcaagaaggagtgctgccggatactaaagaaacctttatcatcgg
gcgagaggtcccggctgacgatccggaagcgggcacctttctaacgggtccgaatctgtggccagacctgcctactgaagatttccaagaagtcattctggcctaccaagc
gcgcatgattgagctggctcgtgtcgtagtgcgtattttagtacagggcctgcctgaagcgtggggatgtccaccggatgttctggatggtctgaccgtcgatccggccatcccg
atgcgcatgctgcactatggccccgtcaaagaaaaggatccacgtcaatttggtgttgctgcccataccgactttagtgctattaccattcttttgcaacagccgggaactgagg
gtctgcaagtttggtatccgccgacagacgagtggattccggtccctgtatctgaagatggttttgtgattaacattggagacttaatgcagcaatataccgcgggctattatcga

tctgcacgccaccgcgtgattaccttctcagaagaagataaacaccggtatagcgtagctttcttcctggatggtaatctgcaattcaagaccaaagcgctggatggctctggt
accgagatcgtcgttggagagtacgtatattcatgcctcaatcgtaccctgggagcgcgtggcccgtccctgtaa

D9 protein sequence:
MSSEKSEGGIPTVDIGAILSPAANEADRRQVVEAVRYASTTFGFFNLVGHGIPRAQLRQIFESSKLFFALPEEKRRDVHVGQ
ALGRSFRGWEPPLIQQHQEGVLPDTKETFIIGREVPADDPEAGTFLTGPNLWPDLPTEDFQEVILAYQARMIELARVVVRIL
VQGLPEAWGCPPDVLDGLTVDPAIPMRMLHYGPVKEKDPRQFGVAAHTDFSAITILLQQPGTEGLQVWYPPTDEWIPVPV
SEDGFVINIGDLMQQYTAGYYRSARHRVITFSEEDKHRYSVAFFLDGNLQFKTKALDGSGTEIVVGEYVYSCLNRTLGARG
PSL

E1 codon-optimized sequence:
atgacaagtccggtggaagccattccgactgtggatatttcgccgttcgtagccaacgcacctgccaaagcgctactggacgtggtaaaagcgatgcgtcacgcctgtacg
acctatggcttcttttatttggtcggtcatgggatttcgcttgaagaacaaaacggcgcgttagattgcgccaaactattcttcacgctgagtcaagaacagaaaatggatgtctg
gctgaagaaatctttaggtaaatcgcaccgagggtatgaaccacctggcatccaaacccaccagaagggtctgttaccggatacaaaagaagcatttatctttggtgctgaa
atccccgaggatgatcccgaagcaggtacctttagcaccggcccgaacctgtggccaaaagcgctgaagccgcatgagtttcgcaacccgatcatggaataccagggca
aaatgctggaactggtgaaaatcctacttaaaatcttggcactaggcctgccggaagaatgggaatgcgccccagacgtgtttgatgagcttgcgataaatccatctatgccg
atgcgcctgctacactatgccccaaacccgacgcataacaatactcagttcggggtgggggatcataccgactttgggtgtatcactgtgctgttacaagagccaaaccgtg
aggggctccaggtctggtatccgccgactgagacttggattccggtaccggttaaggaaggatcatacgttataaacatcggtgacatgatccagaaatacaccggtggcta
ctaccgttcggcgcgccaccgtgtggtcaatggcggtaaagagcaccggtactctgctccattcttcttaaatggtcagttaaaacttaaagttaccgcccttgacggcagcgg
cgtggagacagttgtcggcgaacatatccgtcaacgtttgatcgatacgatgggagaatccggtaacgtgttaaaataa

E1 protein sequence:
MTSPVEAIPTVDISPFVANAPAKALLDVVKAMRHACTTYGFFYLVGHGISLEEQNGALDCAKLFFTLSQEQKMDVWLKKSL
GKSHRGYEPPGIQTHQKGLLPDTKEAFIFGAEIPEDDPEAGTFSTGPNLWPKALKPHEFRNPIMEYQGKMLELVKILLKILAL
GLPEEWECAPDVFDELAINPSMPMRLLHYAPNPTHNNTQFGVGDHTDFGCITVLLQEPNREGLQVWYPPTETWIPVPVKE
GSYVINIGDMIQKYTGGYYRSARHRVVNGGKEHRYSAPFFLNGQLKLKVTALDGSGVETVVGEHIRQRLIDTMGESGNVLK

E2 codon-optimized sequence:
atgggtagcataaatgaaacccagaccacctcaattcccaccgttgacatcggcccatttctggatggacaggccgctcaggaagctaaggacgcagtcgtggccgcgat
gcgccatgcctgctctaccttcggtttcttctatctggtgggacacggcatcccagaagaagatcgcttgcagattctgaaatgcacgaaacgctactttgatctgccgatggaa
gaaaagatggaaacatggattggcaaagcgataggccgatcattccgcggctatgagccgccagcgctgcaagtgcaccaggaaggcctgctgcctgatacaaagga
aggtttcatcctgggtcgcgagactcccgcggacgcccccgaggcaggtacattccatacgggccctaatcagtggcccaaaaccctgccggacgaggaatttcgcgtgc
caatcatgagataccacgcgaagatgattgagatggtgaaagtgattctcaaaatcttagctcttggactgccggcagaatggaattgtccatctgatgtctttgacgaattgac
gataaacccatcggctcccttacgtttactctactatgccccccagccggtcaagcacgaaaatcagtttggcgtcggtgctcataccgactttggaaatgtttctgtgctgctac
aagaagaagattccgaaggcctagaagtgttttatcccccactcagacgtgggtgccggtcccagccaaacctcatagttatgttattaatatgggcgatatgatgcagaaa
tggaccgcgggctattaccgttccgctctgcatcgtgtagttaattcaaatacgaagcctcgttattcggccccgttcttcatgaatgggaacctggatgtgaagtgttacgcgttg
gacggttctggggaagtgactacgattggcgagcatattcgcaaacgtttaatggaaacgatcgggggcgaagaaggtaagaagctgggcctgtaa

E2 protein sequence:
MGSINETQTTSIPTVDIGPFLDGQAAQEAKDAVVAAMRHACSTFGFFYLVGHGIPEEDRLQILKCTKRYFDLPMEEKMETWI
GKAIGRSFRGYEPPALQVHQEGLLPDTKEGFILGRETPADAPEAGTFHTGPNQWPKTLPDEEFRVPIMRYHAKMIEMVKVI
LKILALGLPAEWNCPSDVFDELTINPSAPLRLLYYAPQPVKHENQFGVGAHTDFGNVSVLLQEEDSEGLEVFYPPTQTWVP
VPAKPHSYVINMGDMMQKWTAGYYRSALHRVVNSNTKPRYSAPFFMNGNLDVKCYALDGSGEVTTIGEHIRKRLMETIG
GEEGKKLGL

E3 codon-optimized sequence:
atggggagcgttgaaaccattccaaccgttaatatctccgcctggctcgatccgtcaagtaccgaggaagaaaggcagaacgtggtcgaagagatgcgtcatgcttgtacc
acttacggtttcttatccctggttggtcatggcgtttcgcctgaggagcagaaacaagcaatggactgcgccaaactcttcttcacattgtctgaggaagaaaagatggatgtgt
ggatcggtaaatgtaaaggtcgttcttttcgtggttacgaaccgcctggtattcaagtacatcaggaaggtctgcttcctgataccaaagaatgttttattattggccatgaagttcc
agaggatcaccctgactctggcacctttagtactggtccgaacttgtggccgaaatcgcttccagaaaatcagttccgcattccgattatgcaataccaggcgcgtatggtgca
acttgttaaagttcttctcaaaatcttggctcgtggcctgccgtcggaatggaattgccccccggatgcgctggacgccttagctgaaaatgaaccctctatcccaatgcgcttgc
tccattatgcgccgcagccggtccgcgatgagcggcagttcggcgttggcgatcataccgacttcggttgtattaccattctgctccaggaaatgggtactgagggcttggaag
tgtggtatccacccacggaaacatggattccggtgccgcctcaggagaacgcgtatgtaattaacataggagatatgattcagaaatttaccggggggtattatcgctctgcg
cgtcatcgtgtgattacttttacggagaaacatcggtattccgttccattctttctcaatggccaactgaaattgaaatgtacggccctggacggtagtggcgtcgaaaccatagt
cggcgatcatatccgaggtcgcctcatcgaaaccatgggcaaaaccggcgagaaattgaagtaa

E3 protein sequence:
MGSVETIPTVNISAWLDPSSTEEERQNVVEEMRHACTTYGFLSLVGHGVSPEEQKQAMDCAKLFFTLSEEEKMDVWIGKC
KGRSFRGYEPPGIQVHQEGLLPDTKECFIIGHEVPEDHPDSGTFSTGPNLWPKSLPENQFRIPIMQYQARMVQLVKVLLKIL

ARGLPSEWNCPPDALDALAENEPSIPMRLLHYAPQPVRDERQFGVGDHTDFGCITILLQEMGTEGLEVWYPPTETWIPVP
PQENAYVINIGDMIQKFTGGYYRSARHRVITFTEKHRYSVPFFLNGQLKLKCTALDGSGVETIVGDHIRGRLIETMGKTGEK
LK

E4 codon-optimized sequence:
atgggctcgattgggactgcacctgaagcgattccaactgttgatatttcgccgttcattagcaatgcatcagagaaaaccttacgtgaagtggtggaagcagtgcggcacgc
gtgcaccacttacggcttcttctatctggcgggccatggtgtaagccttgaggaccagaacaaagcacttgagtgcgcaaaattattctttactctgccgatggaagatcgtatg
gaagcgtttattattggtgccgaagtgcctgccgacgatccagaagcgggtacttttagcacgggtccgaacctgtggccgaaatctctgccaaaagaggatttccaaatccc
catcatggaatatcagcgcaagatggttcagttggtgaaagtcttgttacagattttagctctggggctgcctaaagaatggggatgcctgccgtcagtgtttgacgaactggcc
gttaatccgagcatccccatgcgtctgctgcacagcgcgccacaaacggtccgtgatgaacgtcagttcggcgtcggcgaccataccgactttggttgtattgcaatcctgctg
caagaaccggcgcgcgaaggattacaggtgtggtacccgccgacggaaacctggatcccggtaccggtaaaagaaggttcctatgtggtgaacattggcgatatgatgc
agaaatacacagcgggctactaccgcagtgccaggcatcgggtggttaactctgcgaaggaacacagatacagtgtgccgttctttctcaatggccagttaaagctgaatgt
taaagcactggatggatcgggggtggagacagttgtcggcgaacacatccgtcagcgtttagttgaacccatgggagaaagcggtaaagttctgggttaa

E4 protein sequence:
MGSIGTAPEAIPTVDISPFISNASEKTLREVVEAVRHACTTYGFFYLAGHGVSLEDQNKALECAKLFFTLPMEDRMEAFIIGA
EVPADDPEAGTFSTGPNLWPKSLPKEDFQIPIMEYQRKMVQLVKVLLQILALGLPKEWGCLPSVFDELAVNPSIPMRLLHS
APQTVRDERQFGVGDHTDFGCIAILLQEPAREGLQVWYPPTETWIPVPVKEGSYVVNIGDMMQKYTAGYYRSARHRVVN
SAKEHRYSVPFFLNGQLKLNVKALDGSGVETVVGEHIRQRLVEPMGESGKVLG

E5 codon-optimized sequence:
atgggatcgaactatggtaccggcgcgaccgcgattccgaccgttgacatctcggctttcctgagtcctacggccagcgacgcgacacgtcaggccgtgatcgaaggagt
atcaaaagcctgccacacctatggattctttaacctggttggccacggcatcccccaggatgtgatgcgacgagcgtttgaatgtaacaaaatgttcttcgaactgccccatga
caagaagatggaagtccgtataagtaaaagcgtgggtcgcagctttcgcggctatgagccgccgggcatccagacgcaccatgaagggcttctgccggacaccaaaga
gactttcatggttggtcgtgaagtgcccgaagatgatcctgattgtgggagcttctctacaggacccaacctgtggccttcgtccctttcgaaagagaaattccaggaccttatca
tggattatcaggggcgaatgcttgcgctggtgaagaacatcttagacatcctggtactgggtctgccgaaagcgtggaagtgcccaccaaatgtgttcgatagcctgctggat
aaaccgtcaatcccgatgcgttttctgcactatgctccggtgcaagctatggatccacgtcagttcggggttgccgaccataccgattttggctgcgtgtcaatcctgcttcaaga
gctgggcacggaaggtctggaagttttctacccgccgagtgagacttgggtaccggtcccagtggtgagcgatggcttcgtagtaaacatgggggacatgatgcagaaata
caccgggggctactaccggagcgcgcgccatcgcgtgctgacgaacaaggacaaacatcgtcatagtattgccttcttcctgaacggcaacttgaaacttaacgccaaag
ccctggatggttccggcgccgaaaccgtcgttggcgagcacatccgtcaacgccttatcgaaacgatgggcgacatggggaaattattgaaacgagaggtggcggcagc
tcaaaactaa

E5 protein sequence:
MGSNYGTGATAIPTVDISAFLSPTASDATRQAVIEGVSKACHTYGFFNLVGHGIPQDVMRRAFECNKMFFELPHDKKMEV
RISKSVGRSFRGYEPPGIQTHHEGLLPDTKETFMVGREVPEDDPDCGSFSTGPNLWPSSLSKEKFQDLIMDYQGRMLALV
KNILDILVLGLPKAWKCPPNVFDSLLDKPSIPMRFLHYAPVQAMDPRQFGVADHTDFGCVSILLQELGTEGLEVFYPPSET
WVPVPVVSDGFVVNMGDMMQKYTGGYYRSARHRVLTNKDKHRHSIAFFLNGNLKLNAKALDGSGAETVVGEHIRQRLIE
TMGDMGKLLKREVAAAQN

E6 (XenC) codon-optimized sequence:
atgggcagtttgaccgacaatgccgcgattcccaccgtggatatctcggcgttcttagatcctaatgcctcgcaggaggcacgtcaggacgttgtgaatgcgatgtcgaatgc
ctgccacgtatatggtttttcaatctggcgggtcatggcattccacaagagaccttgcgcgaagcattcgaacttaataagatgtttttcgccttgccagaggaatcaaaaaaa
gaagtgttgattagtaagtcaatcggacaatcattccgtggttacgagccgcccggcatccagacacatcacaagggccttcttcccgatattaaagagacgttcatggtcgg
gcgtgaagtaccgcttgacgaccctgattgtggcacattctcaacgggtcccaacctttggccctcatctttaccgaaagagaaatttcaggaccgcatcatggcttaccagg
gtagcatgttagaacttgtaaagaatattcttgctattttggcacaagggctgccaaaggaatggggatgctcaccgaccgttttcaactctttattggataagccgagtattcca
atgcgtttcttacactacgccccgtcccctcacaacttgaagatgtacgccagtttggggtagctgaccacactgactttggttgtgtaagtattttgttgcaggagcccggaact
tcgggcttagaggtctactatcccccgtcggactcgtggattcctgtcccagttatcgacgatggattcgtgatcaacatgggtgatatgatgcaacgttacaccgggggatatt
accgctcagctcgccatcgcgtccttacaaaccgcgagaaacatcgtcacagtgtggcgtttttctgaatgggaacttgggattaaaggcgaaggctttagatggcagcga
aacggagacagtagttggagattggattcgcgggcgtctgattgacactatgggacagacggggaagttattgcagcgtgagagtccgaaaccggtagtactgccg

E6 (XenC) protein sequence:
MGSLTDNAAIPTVDISAFLDPNASQEARQDVVNAMSNACHVYGFFNLAGHGIPQETLREAFELNKMFFALPEESKKEVLISK
SIGQSFRGYEPPGIQTHHKGLLPDIKETFMVGREVPLDDPDCGTFSTGPNLWPSSLPKEKFQDRIMAYQGSMLELVKNILAI
LAQGLPKEWGCSPTVFNSLLDKPSIPMRFLHYAPVPSQLEDVRQFGVADHTDFGCVSILLQEPGTSGLEVYYPPSDSWIPV
PVIDDGFVINMGDMMQRYTGGYYRSARHRVLTNREKHRHSVAFFLNGNLGLKAKALDGSETETVVGDWIRGRLIDTMGQ
TGKLLQRESPKPVVLP

E7 codon-optimized sequence:

atgggaagtatcgaaaagattccggaaacagccccggatgctatccctactgttgacatctctccatttacagacccgaatgccagtgaagaggcaaaaaacgctgtagtg
gatgccgtccgtcatgcgtgtacaacatatggcttcttttacctggctggccacggagtgtctgcggaacagcgtgaaggcattttgaactgtgcgaaaaaatttttgaactgcc
gcaggaggaacgtatggacgtctggattggaaagagcatgggcaagtcatttcgcggttatgaacccccaggcatccagacccaccagaaaggccttcttcctgatattaa
ggaatgcttcattattgggcatgaagtgccggcggatgacccggaagcagggaccttttcgaccggccccaatttgtggccgaagaatctgaaagacgaggagtttcgtac
accgatcatggattatcaagctacaatgttagccctgtcgaaagttcttctgaagattttggctcgcgggcttcccaaagcgtggggtcatcccccaacgtcatggatgaatttg
ctgttaatccatctatgccaatgcgtcttctgcactatgccccgcaggaggtcctggatgaacgtcaattcggtgttggagatcacactgattttggaggcatcgcaatcttattgc
aggaaatgaataccaagggacttgaagtatggtaccccccgacagagacatggattccggttccgccgaaggagaacacatatgttatcaacatgggagacatgatgca
aaaatggaccgctggttattaccgttccgcccgccaccgcgtcatcactagcggtaccaaccaccgttactccgtcccatggtttttgaatggccagttgaaattgaaatgtcag
agccttgacggctccggggtagaaaccattgtaggcgagcacatccgccaacgtcttattgcgacaatgccggaggctggtaaggccttacgc

E7 protein sequence:
MGSIEKIPETAPDAIPTVDISPFTDPNASEEAKNAVVDAVRHACTTYGFFYLAGHGVSAEQREGILNCAKKFFELPQEERMD
VWIGKSMGKSFRGYEPPGIQTHQKGLLPDIKECFIIGHEVPADDPEAGTFSTGPNLWPKNLKDEEFRTPIMDYQATMLALS
KVLLKILARGLPKAWGHPPNVMDEFAVNPSMPMRLLHYAPQEVLDERQFGVGDHTDFGGIAILLQEMNTKGLEVWYPPTE
TWIPVPPKENTYVINMGDMMQKWTAGYYRSARHRVITSGTNHRYSVPWFLNGQLKLKCQSLDGSGVETIVGEHIRQRLIA
TMPEAGKALR

E8 codon-optimized sequence:
atgactatcagcgcggagactattccaacagttgatttgtccccctggcttaaccccgatgcgagtgaagaggcaaaggatgcagtcgtaaaagccgtatcccatgcgtgca
gcacctacggcttcttcaatcttgtcggccacgggatcccagtggaggctcaaaataaaatctttgagtgtagcaaggagtttttgacttgccattgaacgagaagatgaaag
tttctgtcgataaatcattgggaaagagctttcgcgggtatgaacccagtcgtatccagacccatcaggaagggcttttgccggatacaaaggaatgtttcatcaccggggca
gaaaccccgccgatcacccggacgcaggtaaattcagtacgggccctaacttatggcctggtacccttccagatgagaacttccgtcagccagtgatggagtatcgtgcg
cgtatgttggacctggttggcatcctgattaagattctgggtcagggtttacctaaagcgtgggggtattcgcctgacgttcttaacgatatccttatcaacccatcgattcctatgc
gtcttcttcattacgctcccgagacaaacatcgacccacgccagttcggagtaggcgaccacaccgatttcggctgtgtctcaatcttgcttcagcaagcgaacacgaaggg
attagaagtttggtatccattaacgaaaacgtggattcccgtccccgtagtcgagaatgcttttgtgattaacatgggcgatacgatgcatcgctggacgggggggtcactatcgt
tctgcacgtcaccgcgttctgatttctggtaaccgccgctactctgtcgcatttttttcttaacggcaaccttaatttgaaaattaaaccgctggatggtagcggggaggaagcctct
gtcggtgaacatatcaacttacgtttggcccacaccctgggtgataacgcaaaataccttaaa

E8 protein sequence:
MTISAETIPTVDLSPWLNPDASEEAKDAVVKAVSHACSTYGFFNLVGHGIPVEAQNKIFECSKEFFDLPLNEKMKVSVDKSL
GKSFRGYEPSRIQTHQEGLLPDTKECFITGAETPADHPDAGKFSTGPNLWPGTLPDENFRQPVMEYRARMLDLVGILIKIL
GQGLPKAWGYSPDVLNDILINPSIPMRLLHYAPETNIDPRQFGVGDHTDFGCVSILLQQANTKGLEVWYPLTKTWIPVPVVE
NAFVINMGDTMHRWTGGHYRSARHRVLISGNRRYSVAFFLNGNLNLKIKPLDGSGEEASVGEHINLRLAHTLGDNAKYLK

E9 codon-optimized sequence:
atgggttctaattccgccgttttaccaacgacaattccgacggtagacatttcgccatttttagacgaaaacgcatcaccagaagcgaaagagcaagtagttgacgcaatgc
gcgatgcatgtacaacgttcggcttcttctacttagttggacatggaatcccagaggaagaccgtcaggctgtgttaaattgtgcgaagcgttacttttatttaccgaaagagga
caagatggagacatggattgggaaggccatggggcgctcttttcgcggctatgagccccctgcattgcaagtacatcaagagggcttattaccggacacaaaagaaggatt
cattattggacgcgagacccatgccgacgctccagaggctggcacttttcatacagggccaaatcaatggccgaaggccctggccgatgaagactttcgtattccggtgatg
aaatatcacgcaaagatgcttgaaatggtcaaagttattttgaaagtgctggctcgcggtttgcctgaacactgggggtgcagcccaaacgtctttgatgaactgacaattaat
ccgtctgccccgttacgtctgcttcattacgcaccccaacctgtaaagcacgacaatcaattcggggttggagatcatacagattttggtaacgtgtccgtgcttttgcaagagg
aggatacagaagggcttgaagtattttatcctccaactgaaacatgggttcctgttccagtcaaagagcattcttacgtgatcaatatgggagatatgatgcaaaaatggactg
cgggatattatcgttcagctcgccaccgcgtggtcaatcataacgttcgttctcgctactcggctccattctttcttaatggcaatttggatttgaaatgtcgcgcattagatggcagt
ggcgttgaaacagttattggagaacacattcgccgccgtttaatggaaacaattggtggcgaggcaggacgcaagttgggcctg

E9 protein sequence:
MGSNSAVLPTTIPTVDISPFLDENASPEAKEQVVDAMRDACTTFGFFYLVGHGIPEEDRQAVLNCAKRYFYLPKEDKMETW
IGKAMGRSFRGYEPPALQVHQEGLLPDTKEGFIIGRETHADAPEAGTFHTGPNQWPKALADEDFRIPVMKYHAKMLEMVK
VILKVLARGLPEHWGCSPNVFDELTINPSAPLRLLHYAPQPVKHDNQFGVGDHTDFGNVSVLLQEEDTEGLEVFYPPTET
WVPVPVKEHSYVINMGDMMQKWTAGYYRSARHRVVNHNVRSRYSAPFFLNGNLDLKCRALDGSGVETVIGEHIRRRLME
TIGGEAGRKLGL

5. Enzymatic library profiling

Procedure: 2.5 mM substrate, 50 mM TES buffer (pH 7.5), 4 mM sodium ascorbate, 4 mM α-ketoglutaric acid, 0.2 mM $FeSO_4$, 10% v/v toluene and 5% v/v dimethyl sulfoxide (DMSO). Reagents were pre-mixed (except substrate) to make a master mix, and 170 µL of this aqueous solution was added to each well of a 96-well plate from the protein production protocol. Then, 10 µL of a 50 mM substrate solution in DMSO were added, followed by 20 µL of toluene to give a final reaction volume of 200 µL per well. The plates were covered with a breathable film cover and shaken at 300 rpm (30 °C) for 2–4 h. The reactions were quenched with 200 µL of an internal standard solution in MeCN (described below). The reactions were centrifuged (2000 rpm for 20 min), filtered with plate-filters, and analyzed by HPLC-DAD. All reactions were biological duplicates.

<u>Enzyme relative activity:</u> Product peak area over internal standard peak area (P/IS), normalized based on highest P/IS.

<u>Results for orcinaldehyde **11**:</u> Reaction time: 3 h. Quenching solution: benzophenone (500 µM) in MeCN. Chromatography conditions. Column: Phenomenex Kinetex (1.7 µm C18, 2.1 x 50 mm). Mobile phase: 5% Solvent B for 1 min, 5% to 100% Solvent B over 1 min, 100% solvent B for 1 min. flow rate: 0.5 mL/min. Solvent A: deionized water + 0.1% formic acid: Solvent B: MeCN+0.1% formic acid. Analyzed at 280 nm.

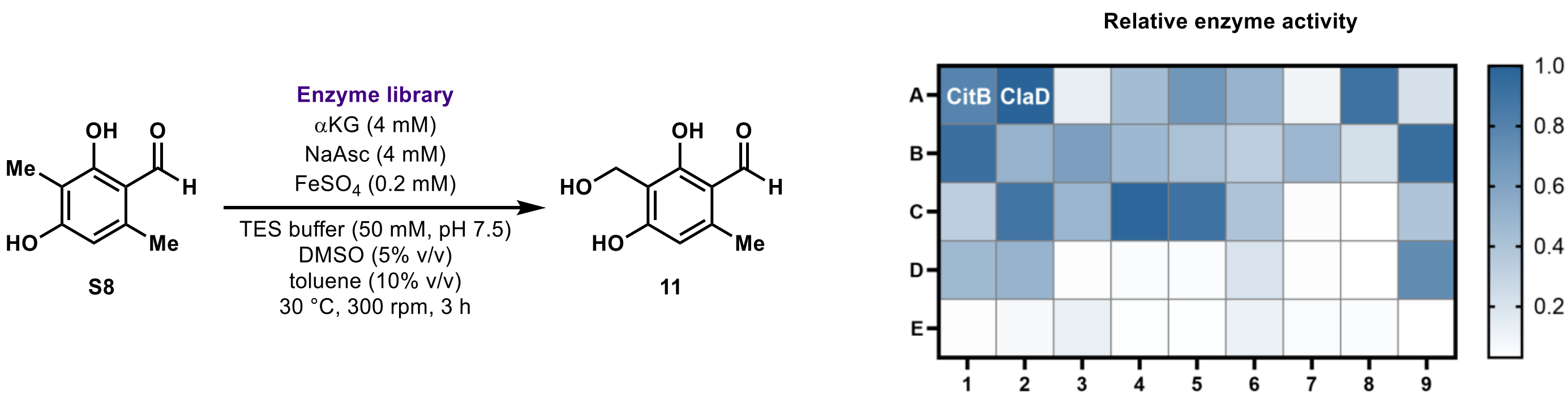

Supporting Figure S5: Enzyme profiling for orcinaldehyde **S8**.

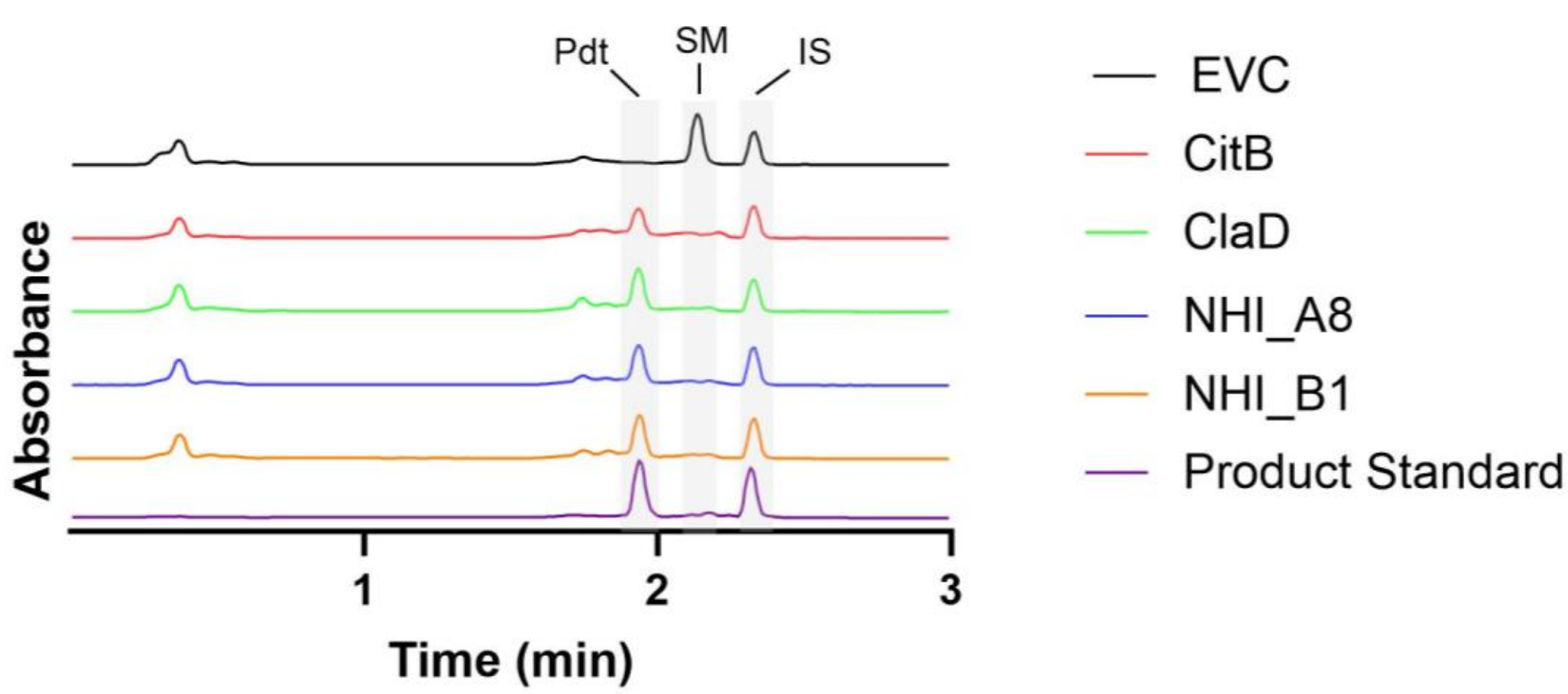

Supporting Figure S6: Chromatograms of several reaction wells, profiling for substrate **11**. Chromatograms show that CitB/ClaD form the benzylic alcohol **S8**. Other enzymes in the library like NHI_A8 and NHI_B1 show the same activity. EVC: Empty vector control.

Results for substrate **7:** Reaction time: 3 h. Quenching solution: L-tryptophan (100 µM) in MeCN. Chromatography conditions. Column: Phenomenex Kinetex (1.7 µm C18, 2.1 x 100 mm). Mobile phase: 5% Solvent B for 1 min, 5% to 100% Solvent B over 2.5 min, 100% solvent B for 1 min, 100 to 5% Solvent B for 0.5 min. Flow rate: 0.4 mL/min, Solvent A: deionized water + 0.1% formic acid: Solvent B: MeCN+0.1% formic acid. Analyzed at 280 nm.

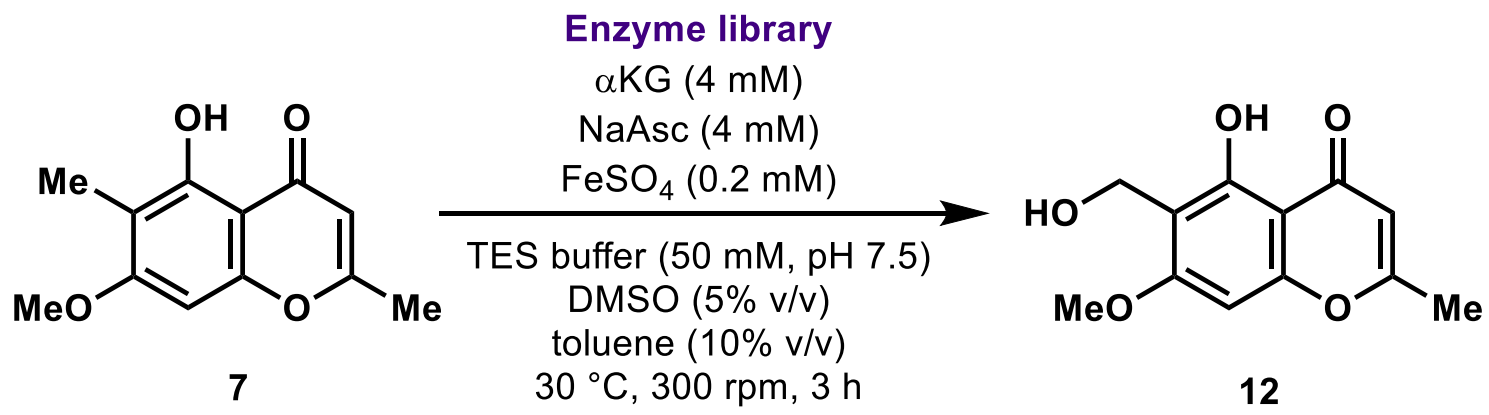

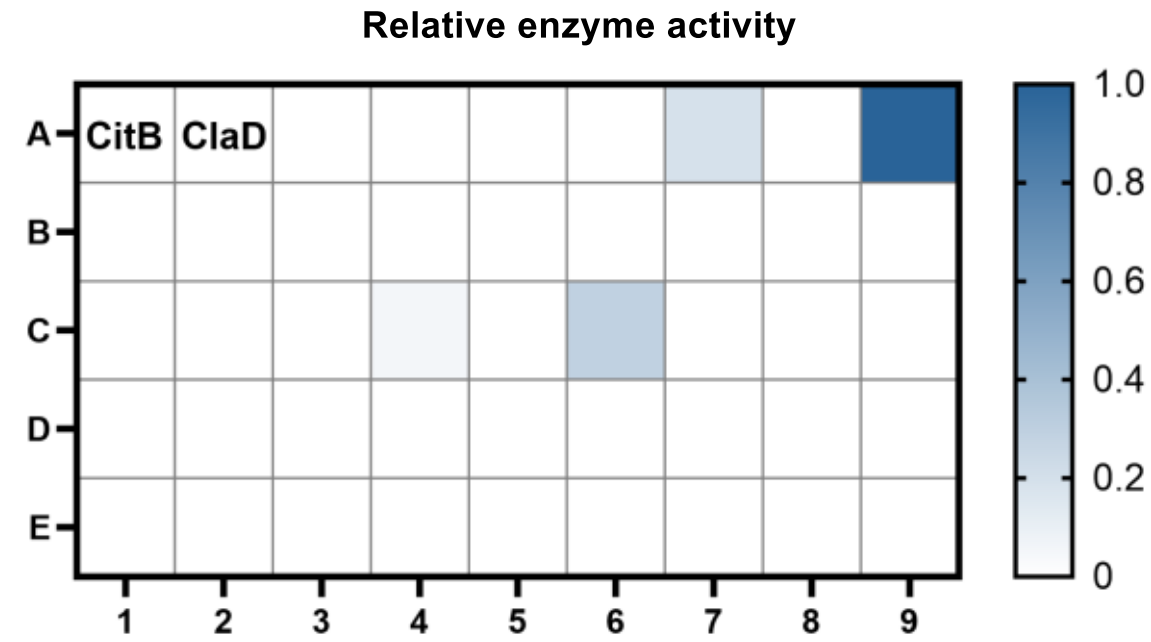

Supporting Figure S7: Enzyme profiling for substrate **7**.

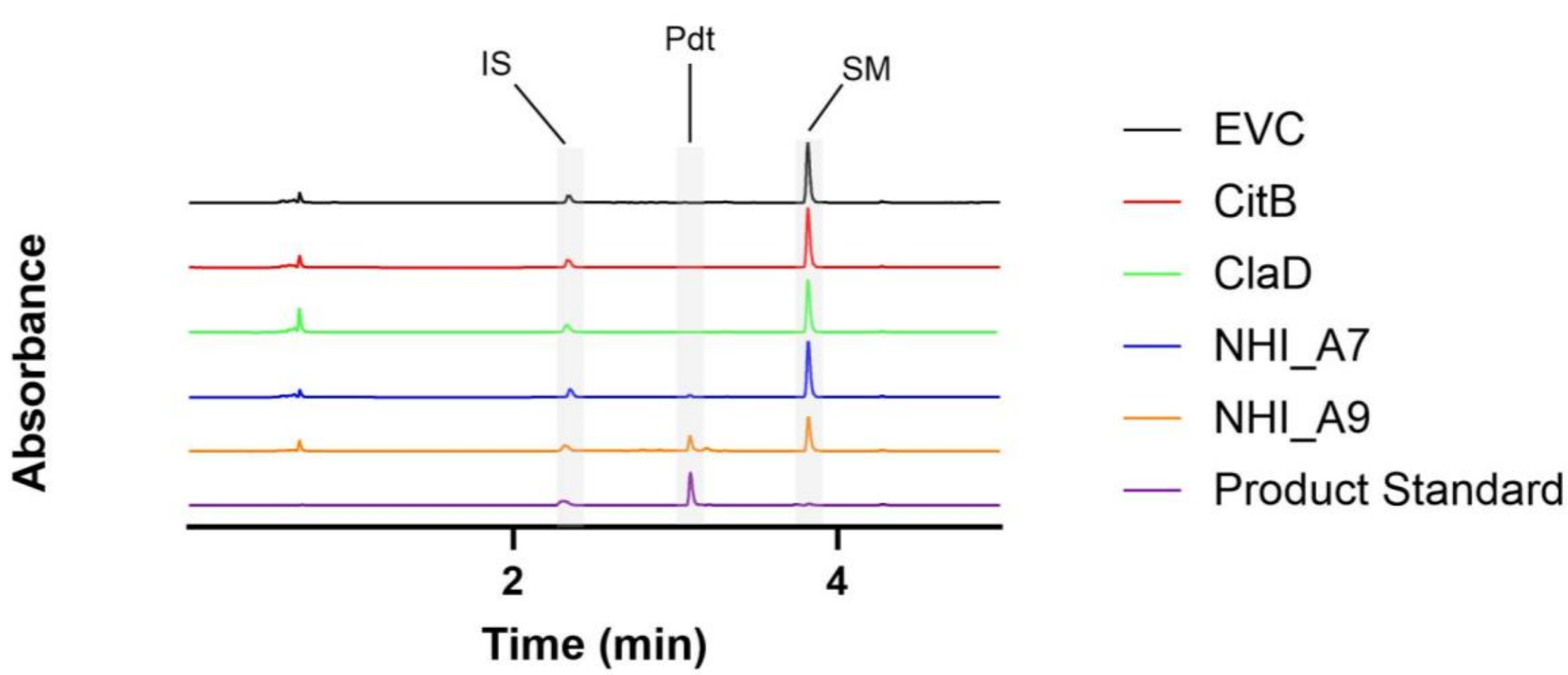

Supporting Figure S8: Chromatograms of several reaction wells, profiling for substrate **7**. Chromatograms show that CitB/ClaD do not produce benzylic alcohol **12**. Enzymes NHI_A7 and NHI_A9 produce the desired product. The latter showed the highest product peak; therefore, it was further investigated. EVC: Empty vector control.

Results for substrate **8**: Reaction time: 3 h. Quenching solution: 3,5-dimethoxytoluene (500 µM) in MeCN. Chromatography conditions. Column: Phenomenex Kinetex (1.7 µm C18, 2.1 x 100 mm). Mobile phase: 5% Solvent B for 1 min, 5% to 100% Solvent B over 2.5 min, 100% solvent B for 1 min, 100 to 5% Solvent B for 0.5 min. Flow rate: 0.4 mL/min, Solvent A: deionized water + 0.1% formic acid: Solvent B: MeCN+0.1% formic acid. Analyzed at 270 nm.

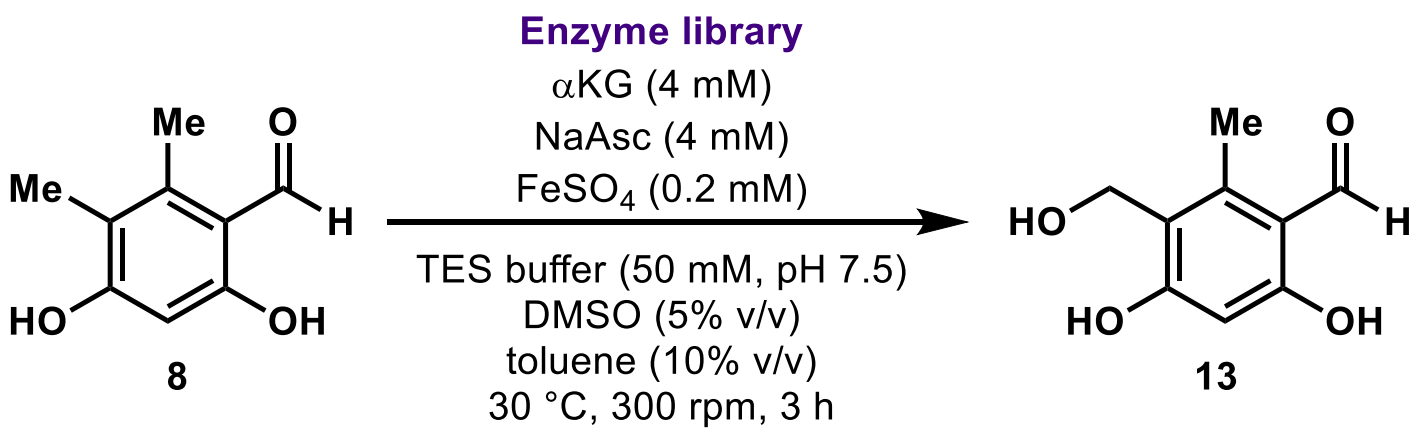

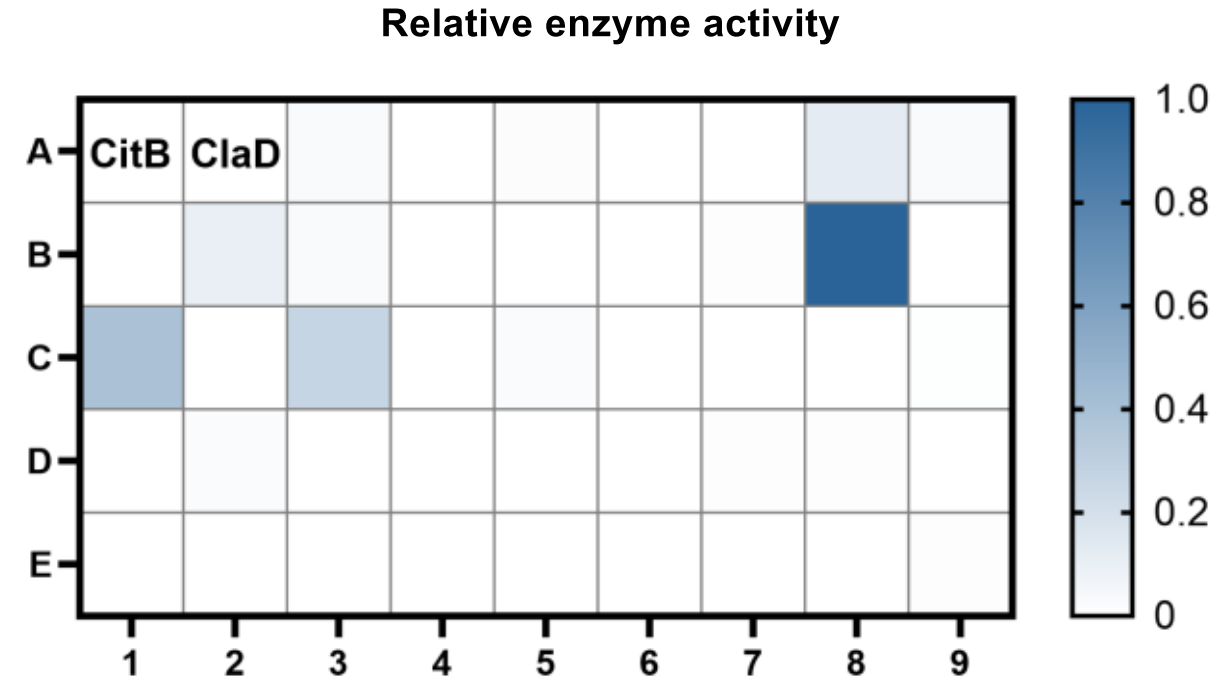

Supporting Figure S9: Enzyme profiling for substrate **8**.

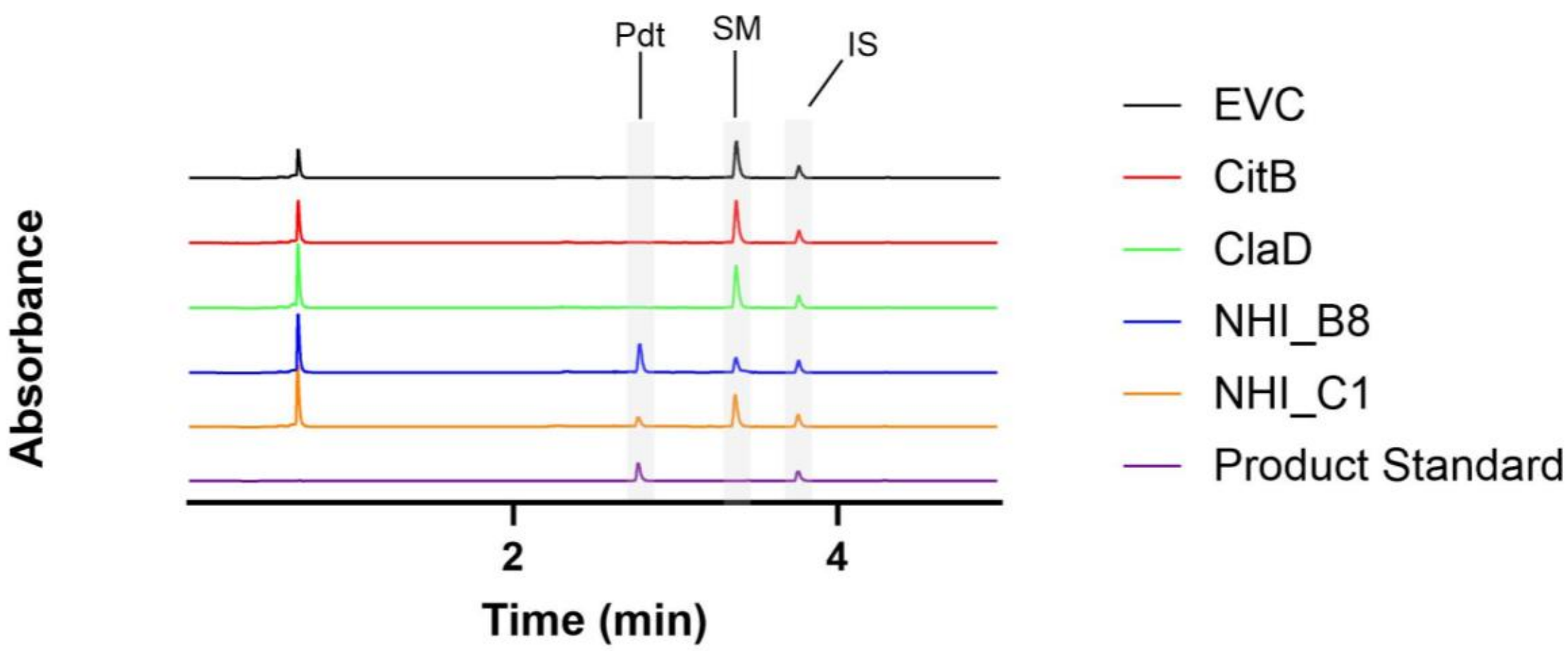

Supporting Figure S10: Chromatograms of several reaction wells, profiling for substrate **8**. Chromatograms show that CitB/ClaD do not produce benzylic alcohol **13**. Enzymes NHI_B8 and NHI_C1 produce the desired product. The former showed the highest product peak; therefore, it was further investigated. EVC: Empty vector control.

Results for substrate **9:** Reaction time: 3 h. Quenching solution: 3,5-dimethoxytoluene (500 µM) in MeCN. Chromatography conditions. Column: Phenomenex Kinetex (1.7 µm C18, 2.1 x 100 mm). Mobile phase: 5% Solvent B for 1 min, 5% to 100% Solvent B over 2.5 min, 100% solvent B for 1 min, 100 to 5% Solvent B for 0.5 min. Flow rate: 0.4 mL/min, Solvent A: deionized water + 0.1% formic acid: Solvent B: MeCN+0.1% formic acid. Analyzed at 270 nm.

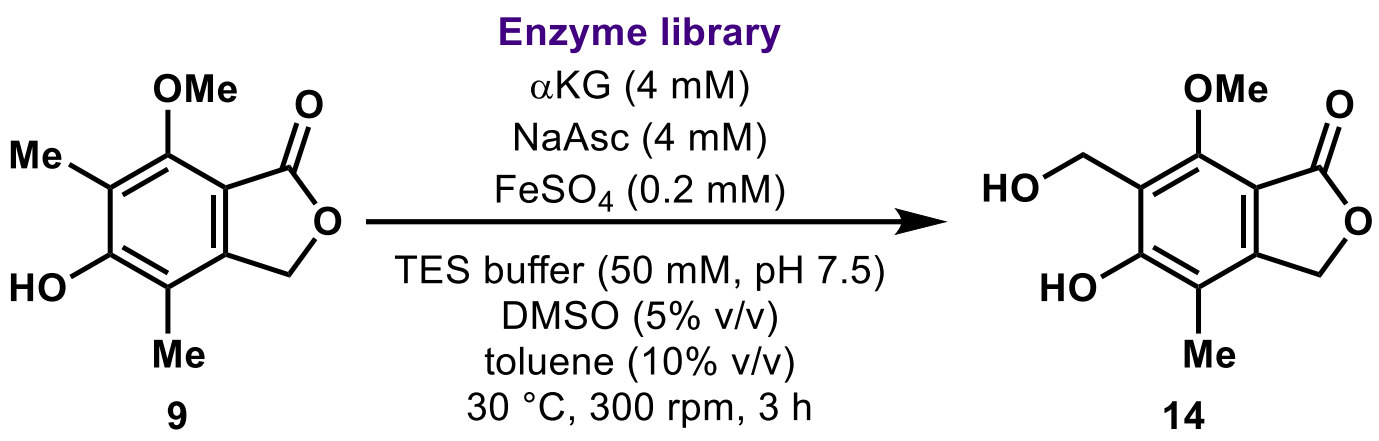

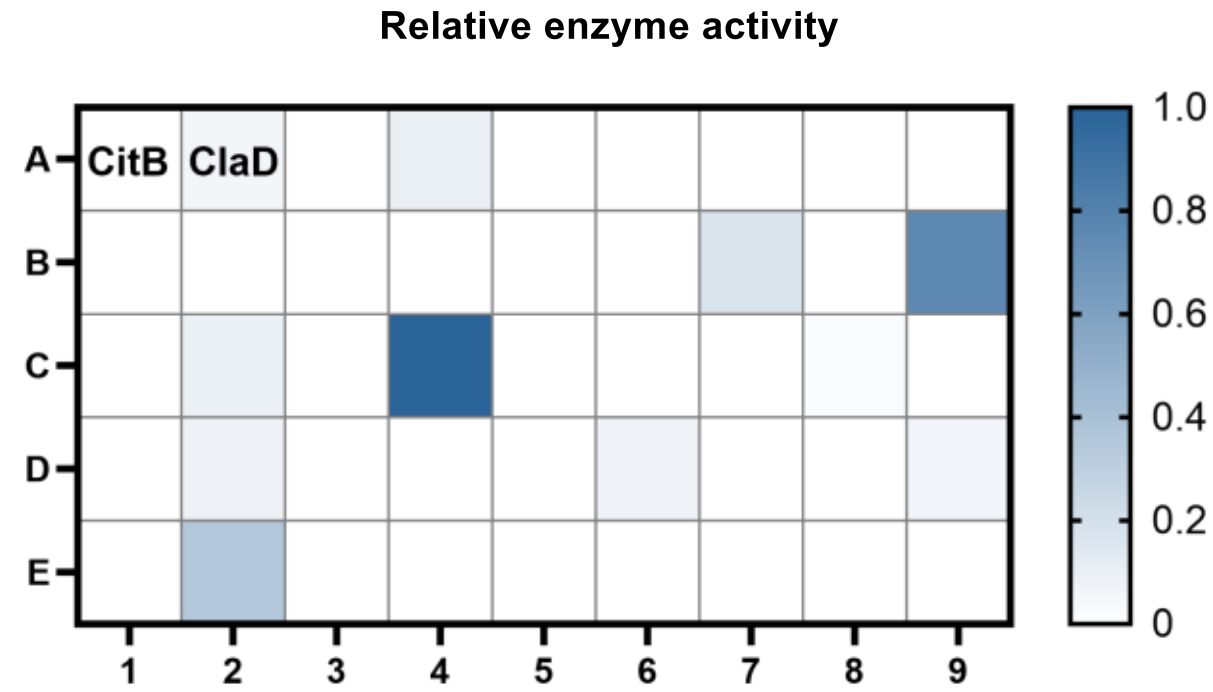

Supporting Figure S11: Enzyme profiling for substrate **9**.

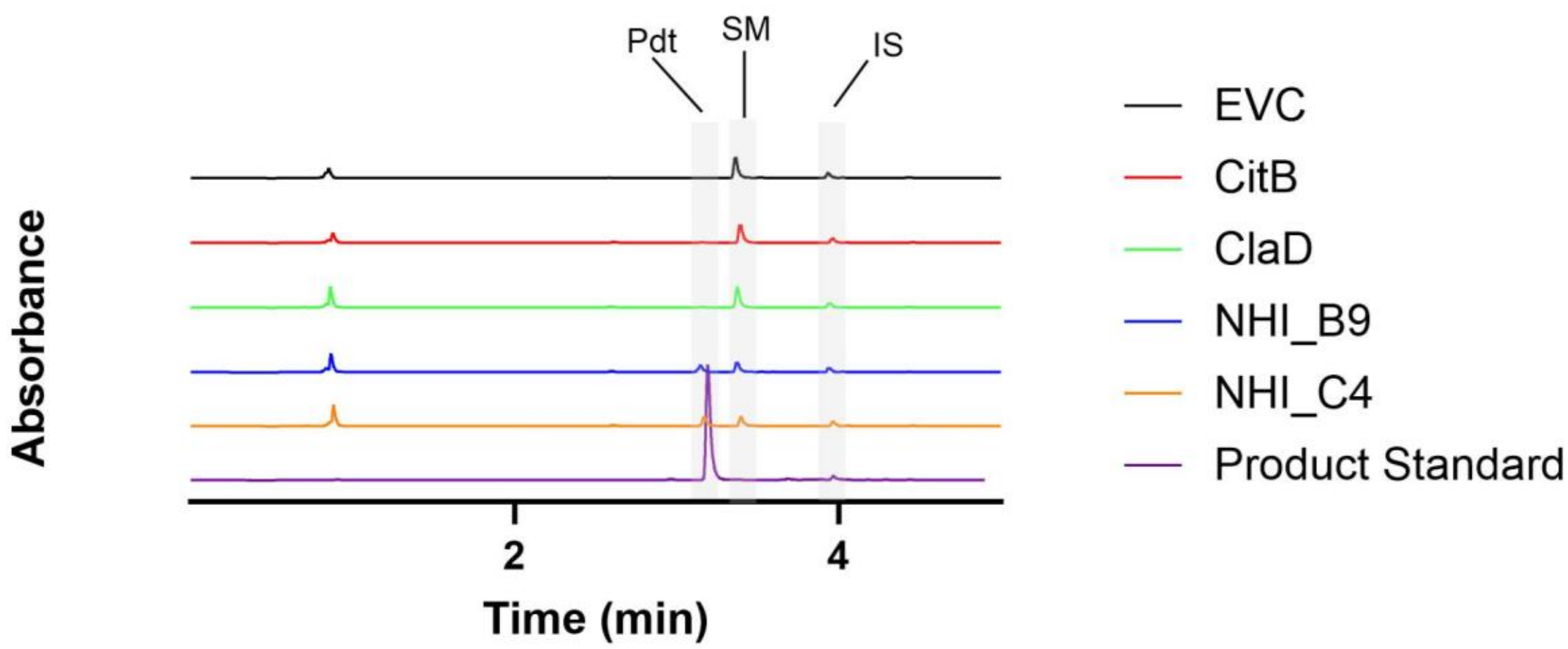

Supporting Figure S12: Chromatograms of several reaction wells, profiling for substrate **9**. Chromatograms show that CitB does not produce benzylic alcohol **14**, ClaD does produce the desired product, but in lower amounts than NHI_B9 and NHI_C4. NHI_C4 showed the highest product peak; therefore, it was further investigated. EVC: Empty vector control. Chromatogram of the product standard was shifted to align the IS.

Results for substrate **10:** Reaction time: 3 h. Quenching solution: benzophenone (500 µM) in MeCN. Chromatography conditions. Column: Phenomenex Kinetex (1.7 µm C18, 2.1 x 100 mm). Mobile phase: 5% Solvent B for 0.5 min, 5% to 65% Solvent B over 1 min, 65% solvent B for 1 min, 65% to 100% solvent B for 1 min, then 100% solvent B for 1 min, and 100 to 5% Solvent B for 0.5 min. Flow rate: 0.4 mL/min, Solvent A: deionized water + 0.1% formic acid: Solvent B: MeCN+0.1% formic acid. Analyzed at 280 nm.

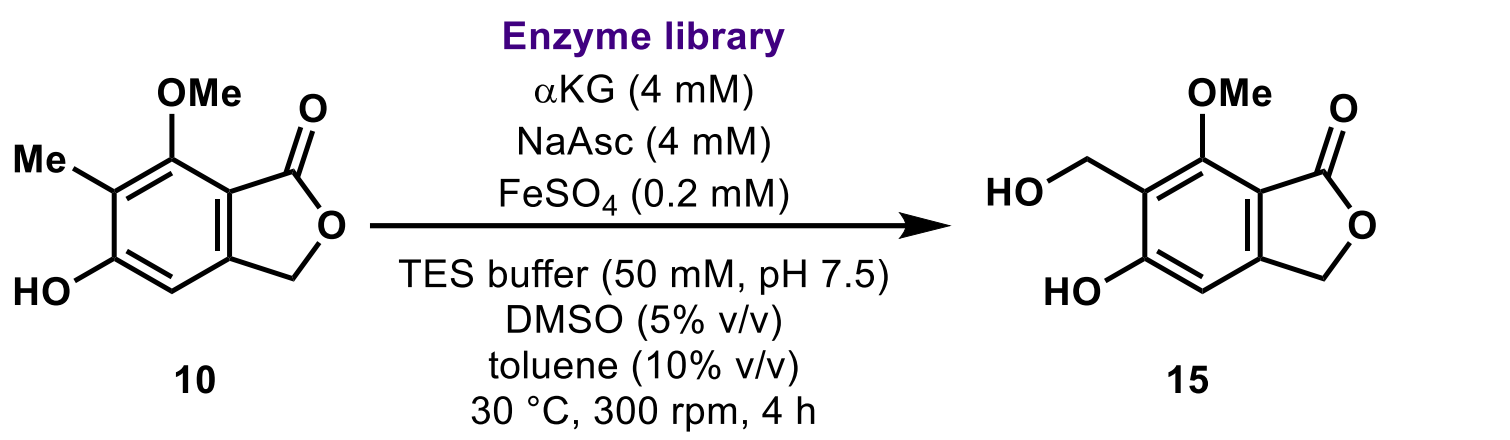

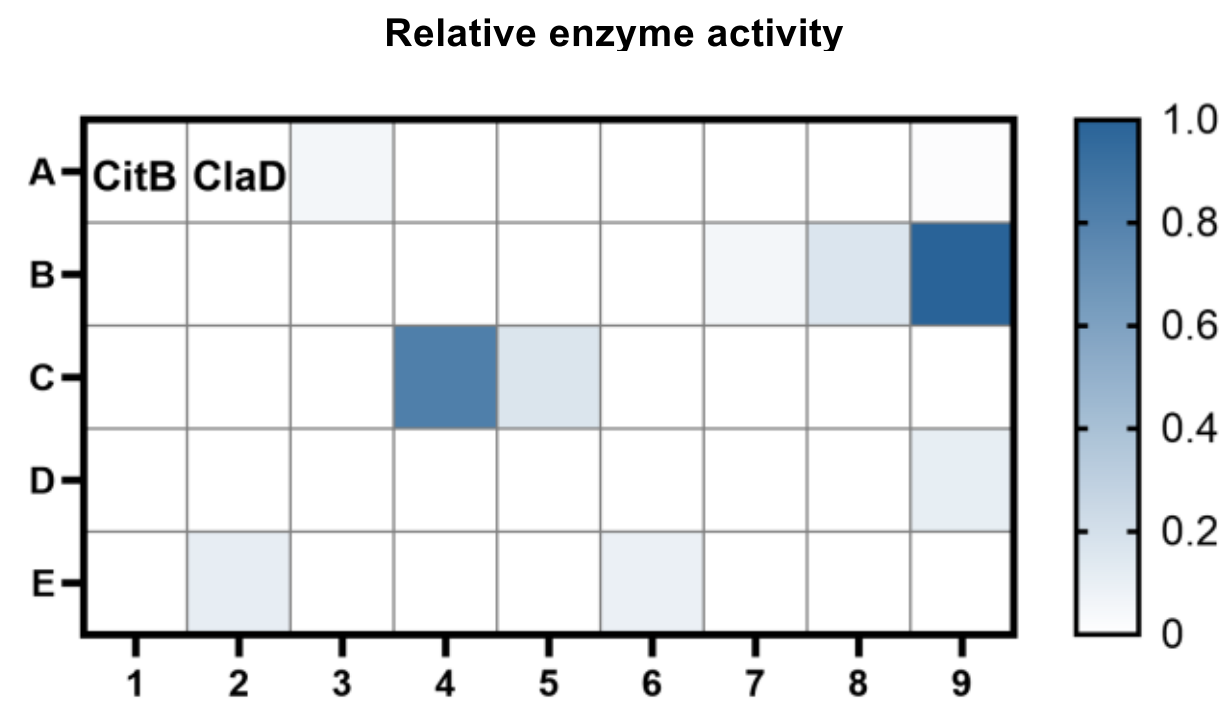

Supporting Figure S13: Enzyme profiling for substrate **10**.

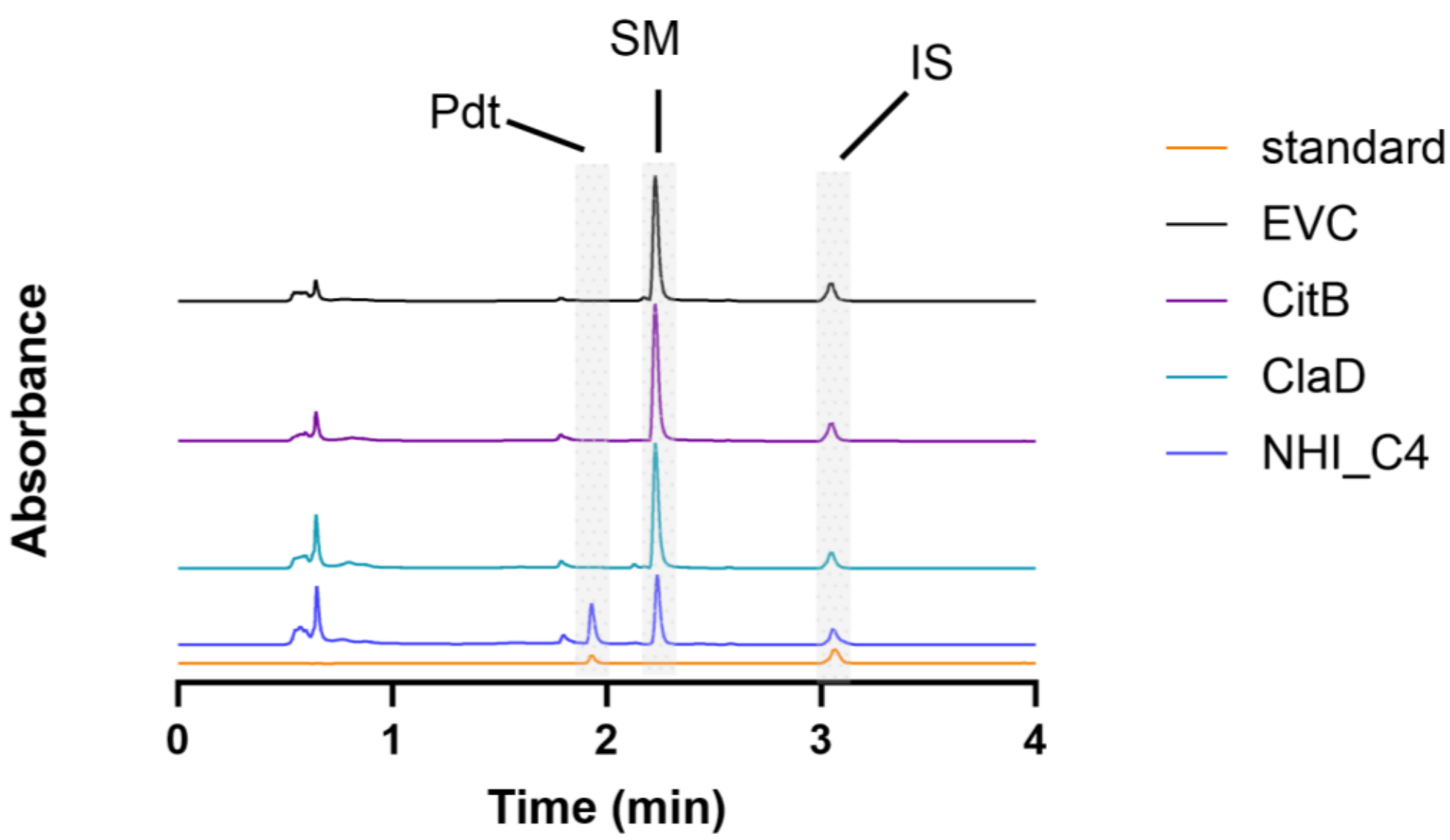

Supporting Figure S14: Chromatograms of several reaction wells, profiling for substrate **10**. Chromatograms show that CitB/ClaD do not produce benzylic alcohol **15**. NHI_C4 produces the desired product. EVC: Empty vector control.

Heatmaps with yields:

*For alcohol **11***

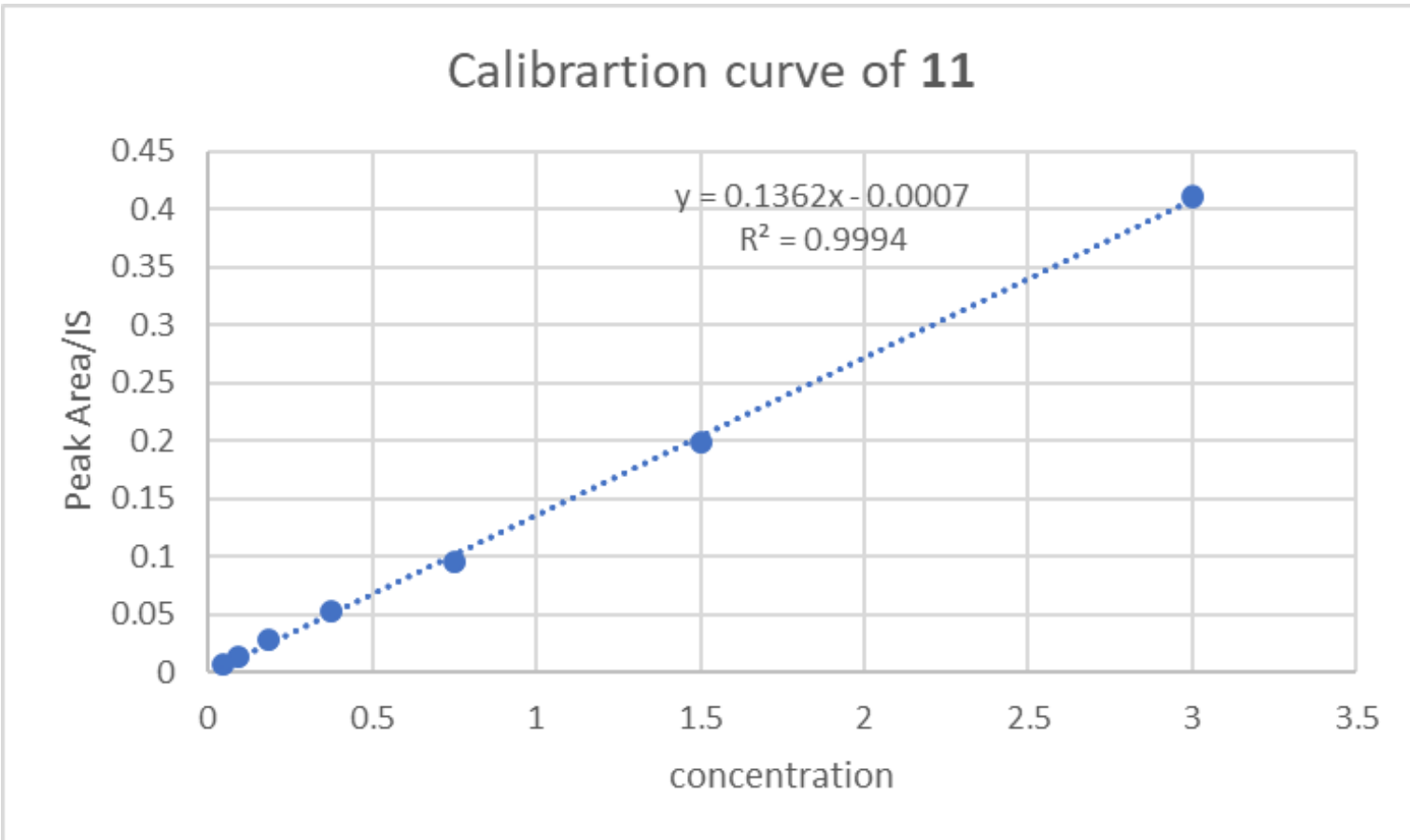

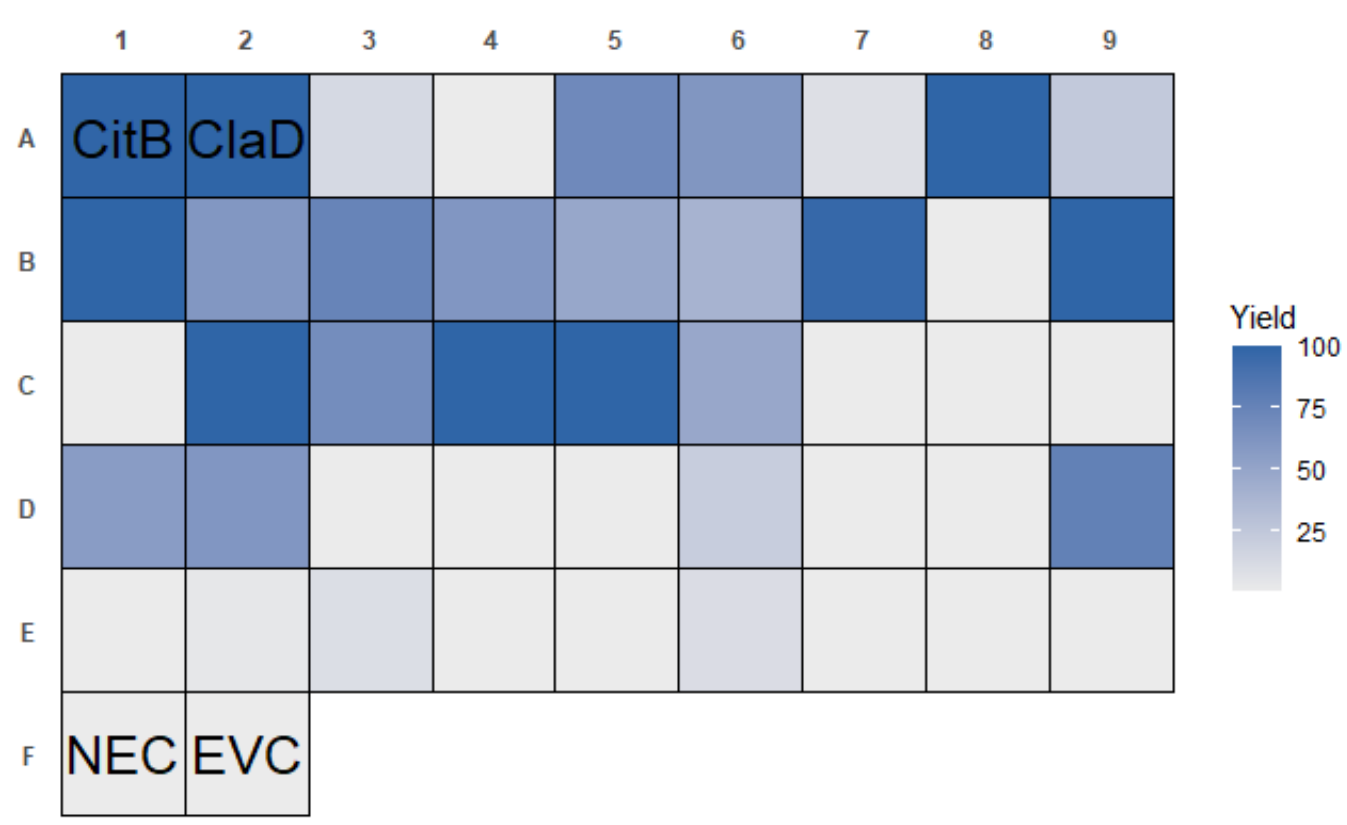

Supporting Figure S15: Quantification for formation of **11** with enzyme library.

well	***yield 1***	***yield 2***	***average***	***sd***
NEC	*0*	*0*	*0*	*0*
EVC	*0*	*0*	*0*	*0*
A1 (CitB)	*97*	*103*	*100*	*3*
A2 (ClaD)	*104*	*102*	*103*	*1*
A3	*15*	*11*	*13*	*2*
A5	*99*	*43*	*71*	*28*
A6	*54*	*69*	*62*	*7*
A7	*6*	*11*	*9*	*2*
A8	*106*	*107*	*106*	*0*
A9	*25*	*23*	*24*	*1*
B1	*102*	*105*	*103*	*2*
B2	*65*	*56*	*61*	*5*
B3	*69*	*82*	*75*	*6*
B4	*49*	*73*	*61*	*12*
B5	*53*	*44*	*49*	*4*
B6	*39*	*40*	*40*	*0*
B7	*92*	*102*	*97*	*5*
B9	*99*	*107*	*103*	*4*
C2	*99*	*109*	*104*	*5*

C3	*78*	*59*	*68*	*10*
C4	*106*	*105*	*105*	*0*
C5	*105*	*108*	*107*	*1*
C6	*45*	*52*	*49*	*4*
D1	*58*	*56*	*57*	*1*
D2	*62*	*61*	*61*	*0*
D6	*22*	*20*	*21*	*1*
D9	*67*	*89*	*78*	*11*
E2	*4*	*2*	*3*	*1*
E3	*10*	*9*	*9*	*0*
E6	*9*	*12*	*10*	*2*

*For alcohol **12***

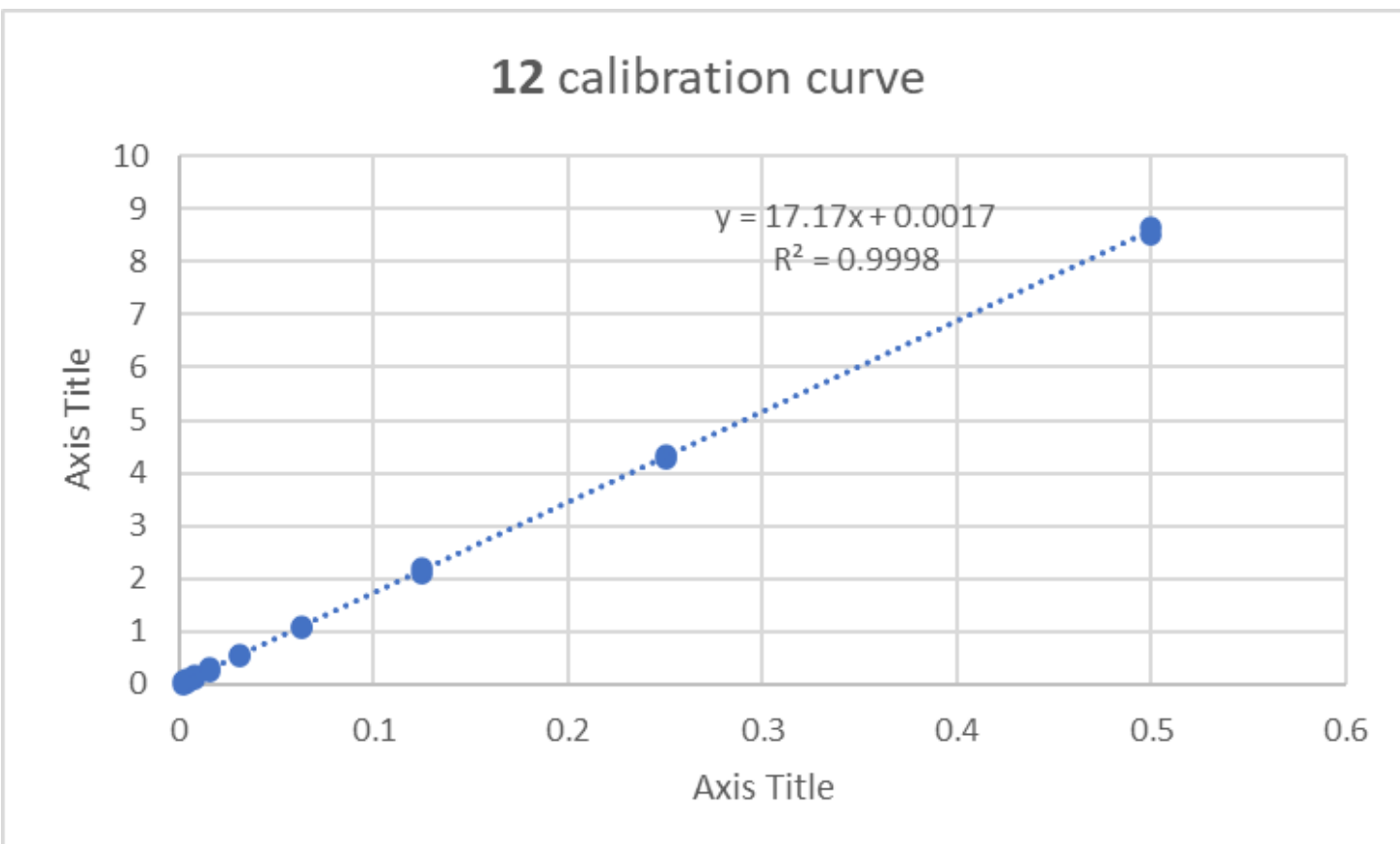

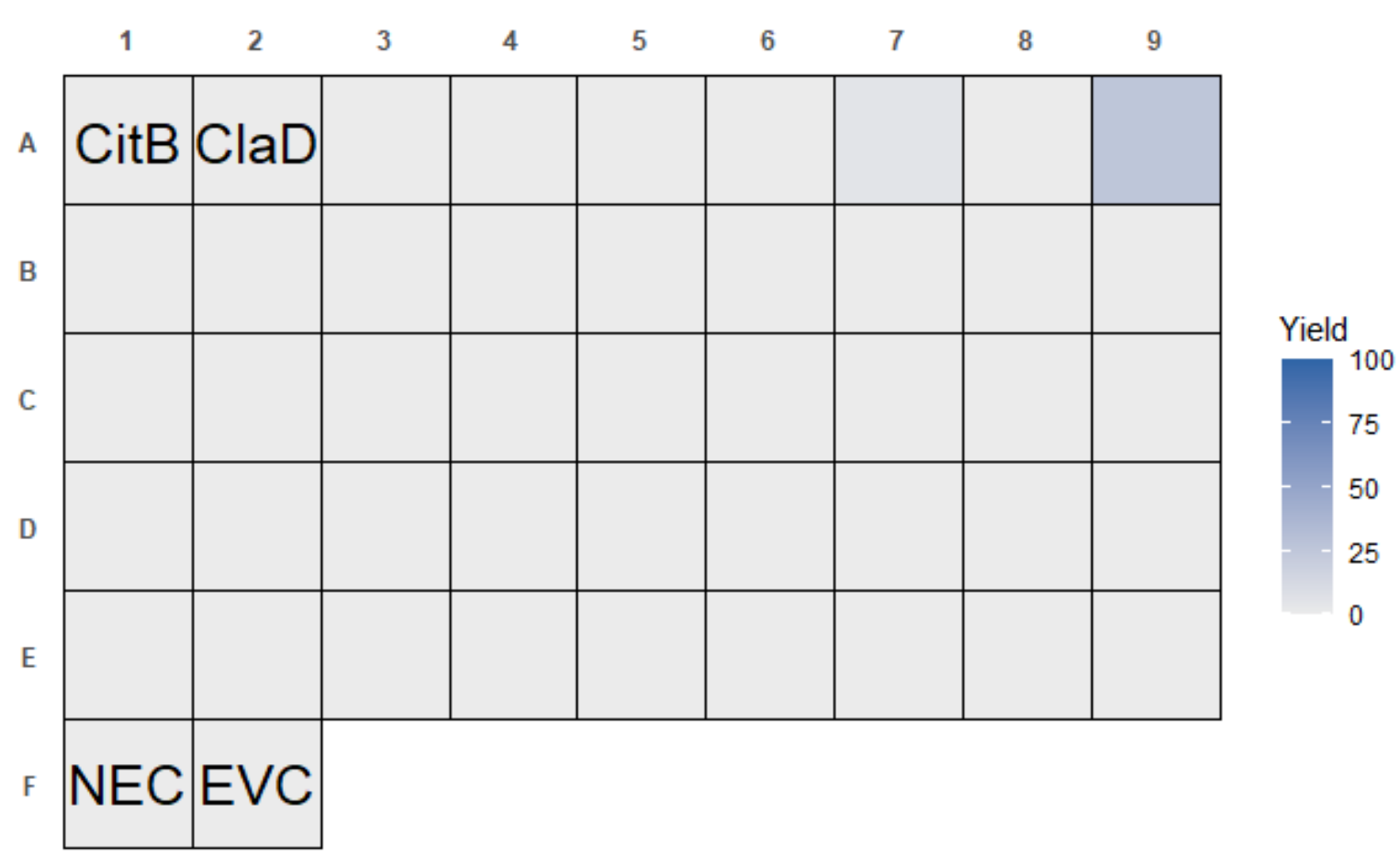

Supporting Figure S16: Quantification for formation of **12** with enzyme library.

Well	*yield 1*	*yield 2*	*average*	*sd*
A1	*0*	*0*	*0*	*0*
A2	*0*	*0*	*0*	*0*
A7	*5.801152*	*4.508988*	*5.15507*	*0.646082*

A9	*21.89582*	*30.26772*	*26.08177*	*4.18595*

*For alcohol **13***

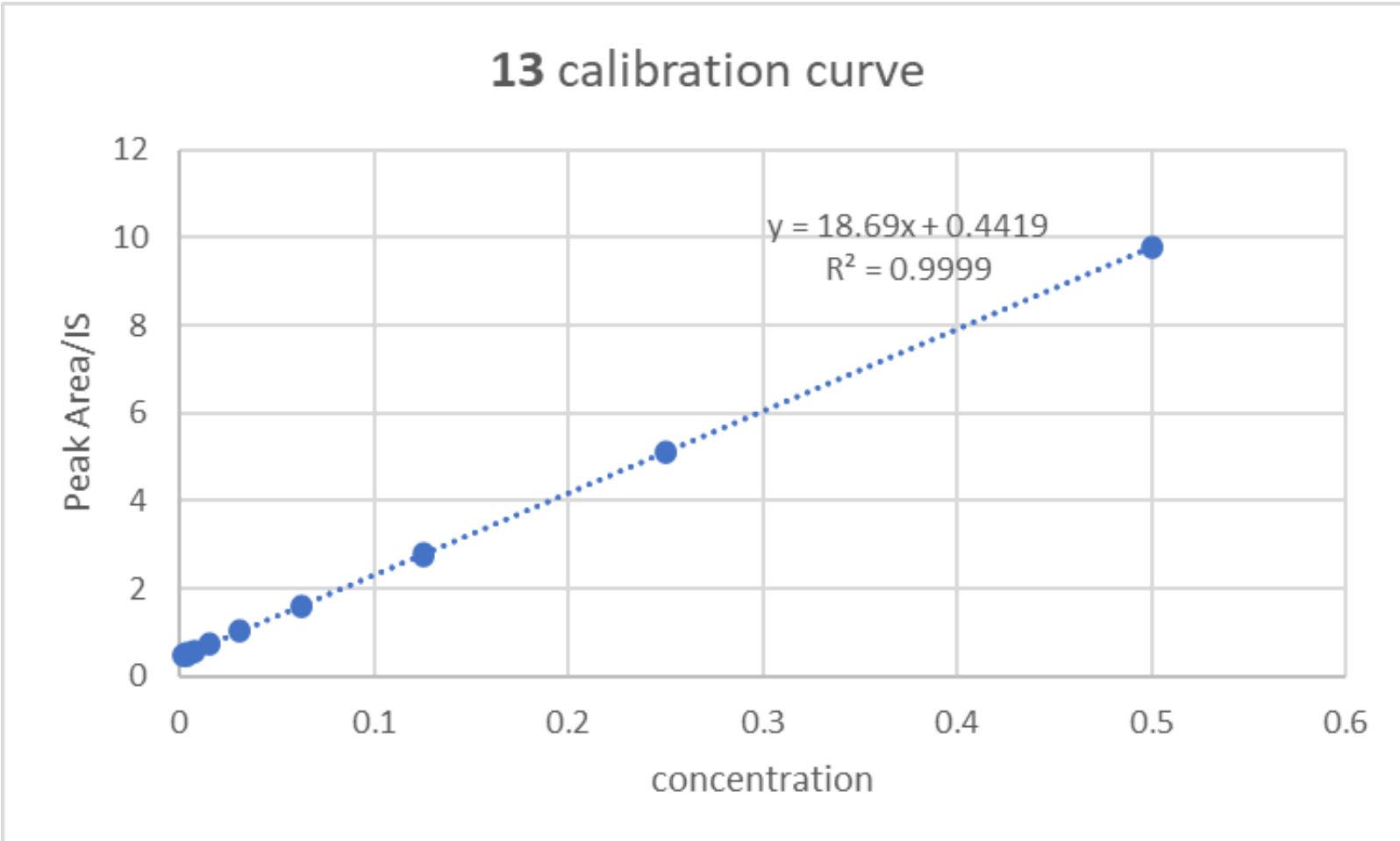

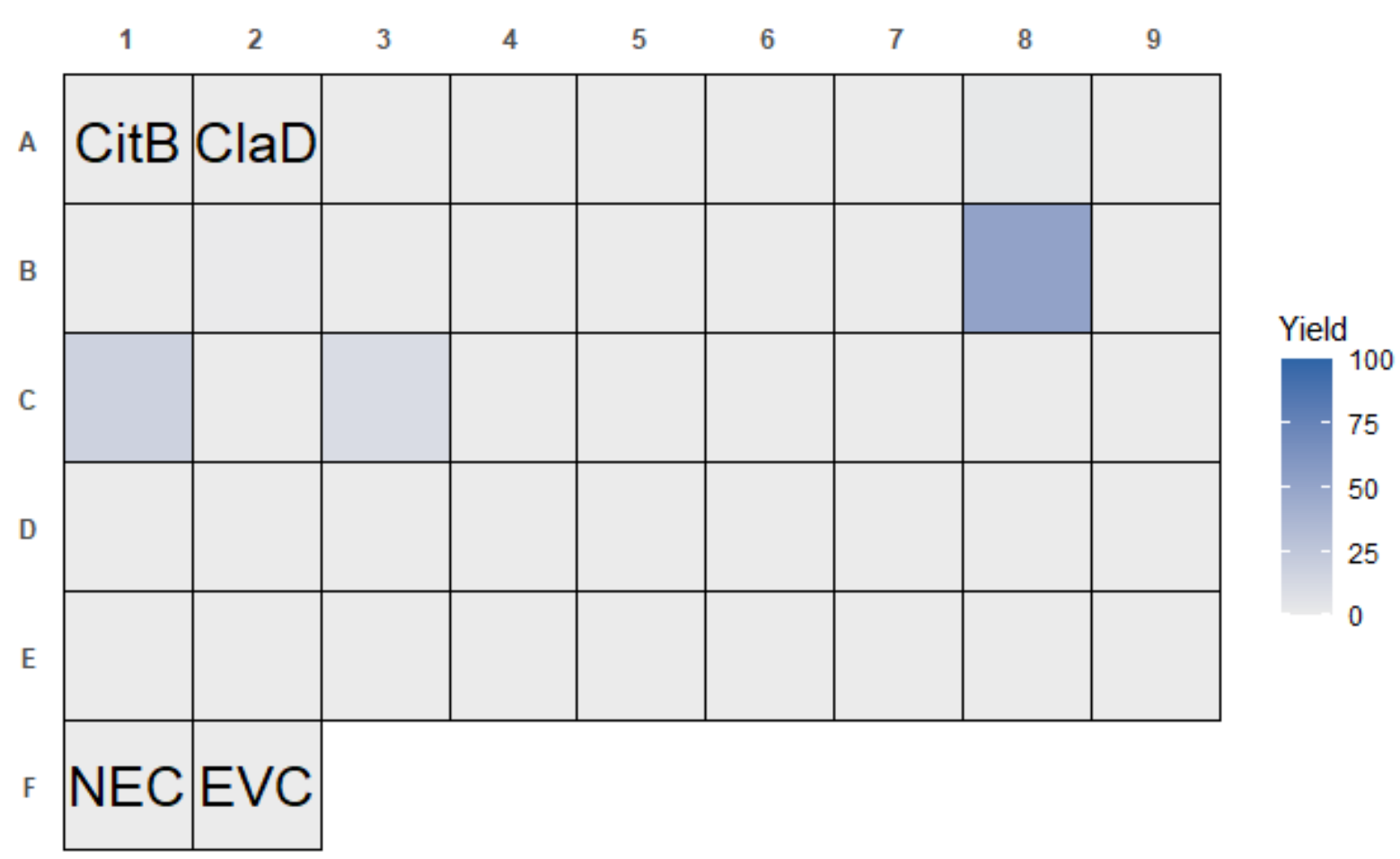

Supporting Figure S17: Quantification for formation of **13** with enzyme library.

Well	yield 1	yield 2	average yield	sd
A1	0	0	0	
A2	0	0	0	
B8	52.16264	51.77768	51.97016	0.192477
C1	21.07682	14.15623	17.61652	3.460295
C3	10.72721	10.12168	10.42445	0.302767

*For alcohol **14***

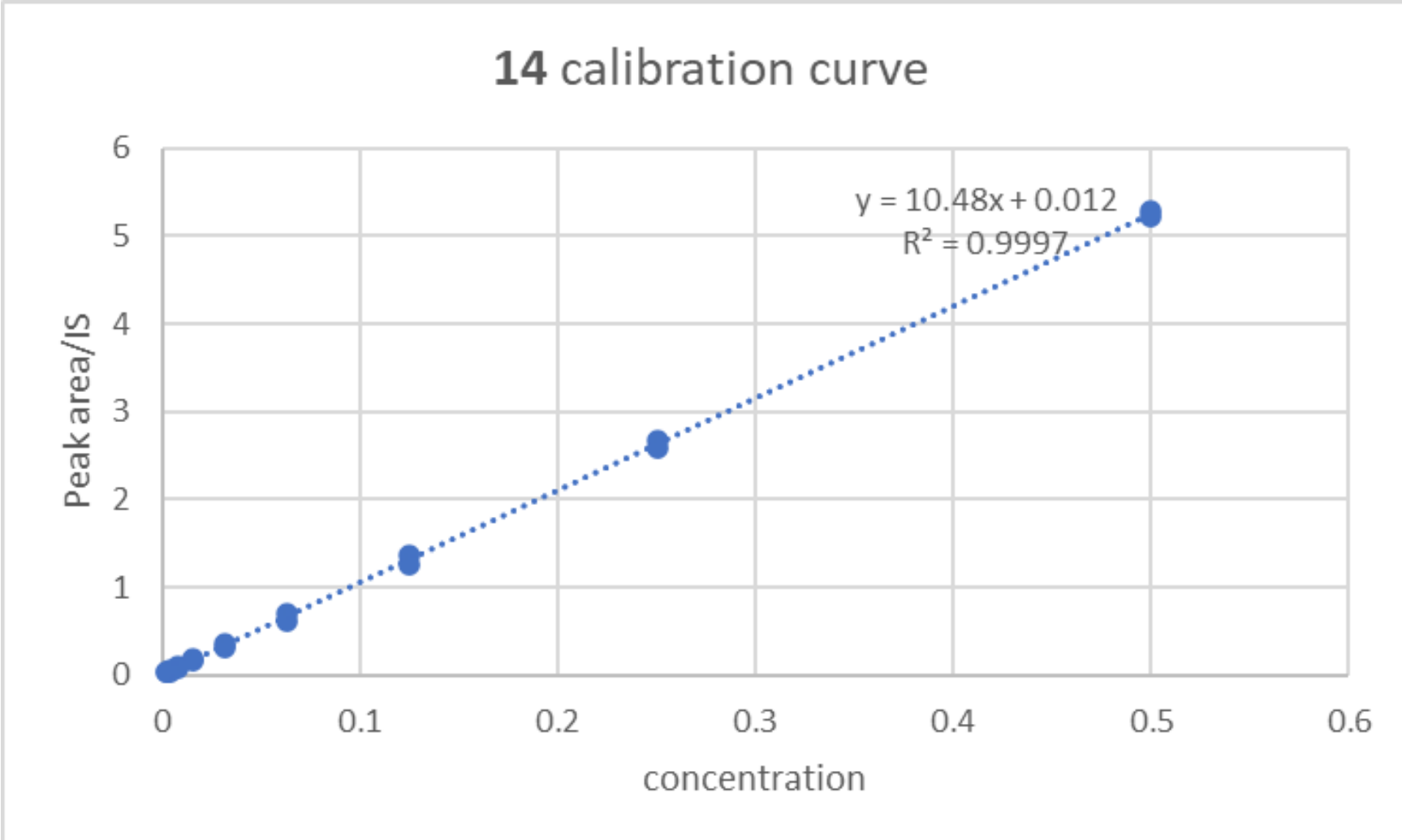

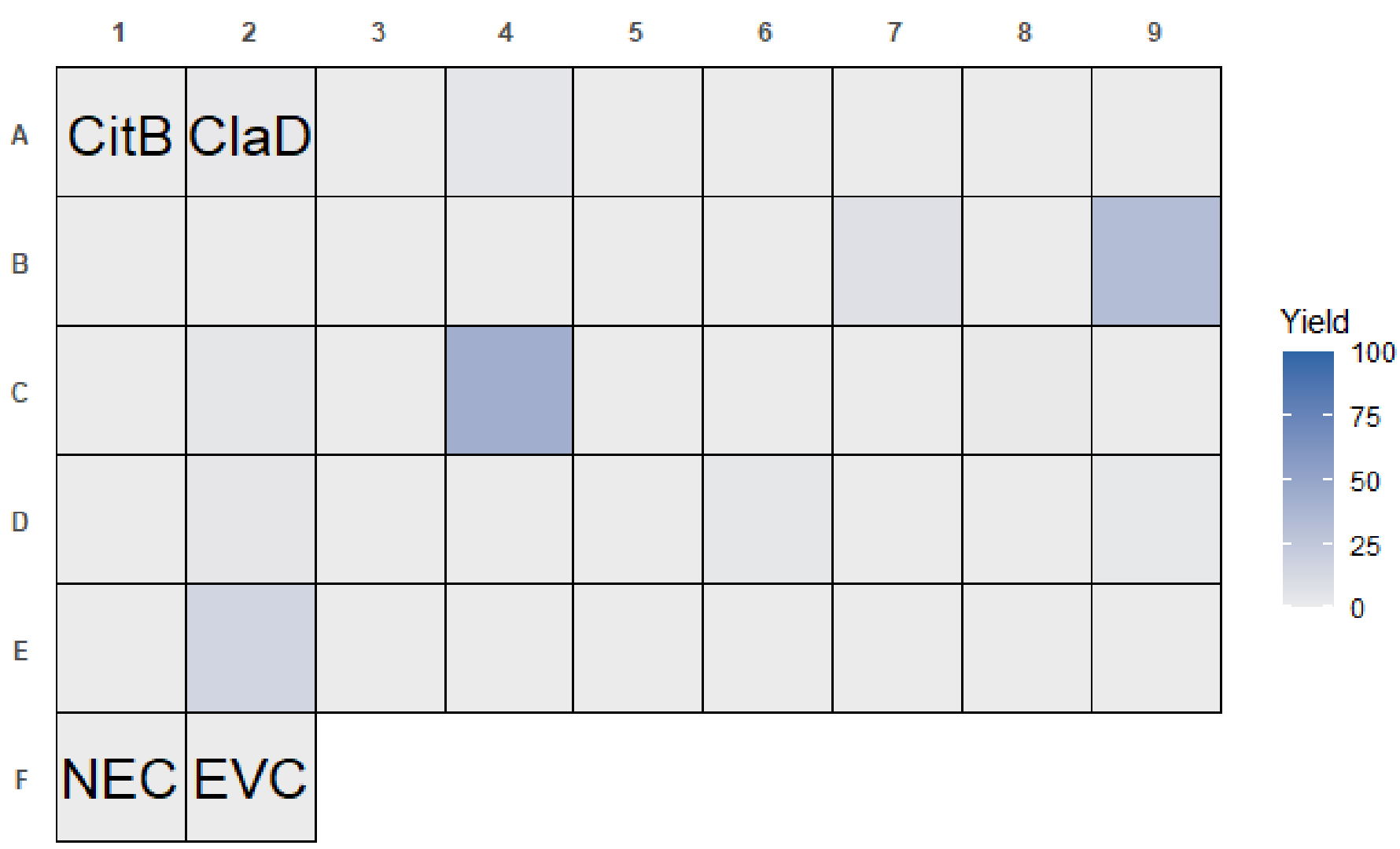

Supporting Figure S18: Quantification for formation of **14** with enzyme library.

Well	yield 1	yield 2	average	sd
A1	0	0		
A2	2.061481	1.963429	2.012455	0.049026
A4	4.288808	3.743072	4.01594	0.272868
B7	7.715077	7.21245	7.463764	0.251313
B9	31.96404	33.29344	32.62874	0.664699
C2	4.377188	3.194521	3.785854	0.591333
C4	43.47123	42.93794	43.20459	0.266646
D2	3.011026	3.302038	3.156532	0.145506
E2	14.99514	15.38125	15.18819	0.193054

6. TTNs for scale-ups

Enzyme concentrations based on protein purifications. Concentration was obtained by Pierce assay in triplicate.

Enzyme	stock concentration (μM)	stock volume (mL)	pellet mass (g)	Enzyme mol per gram of pellet	Enzyme concentration in reaction (M)
A9	561 ± 14	3.8	11	1.94E-07	9.70E-06
B8	313 ± 71	6.2	19	1.02E-07	5.11E-06
C4	431 ± 33	4.9	20	1.06E-07	5.28E-06

Pierce assay calibration curve with known protein concentrations

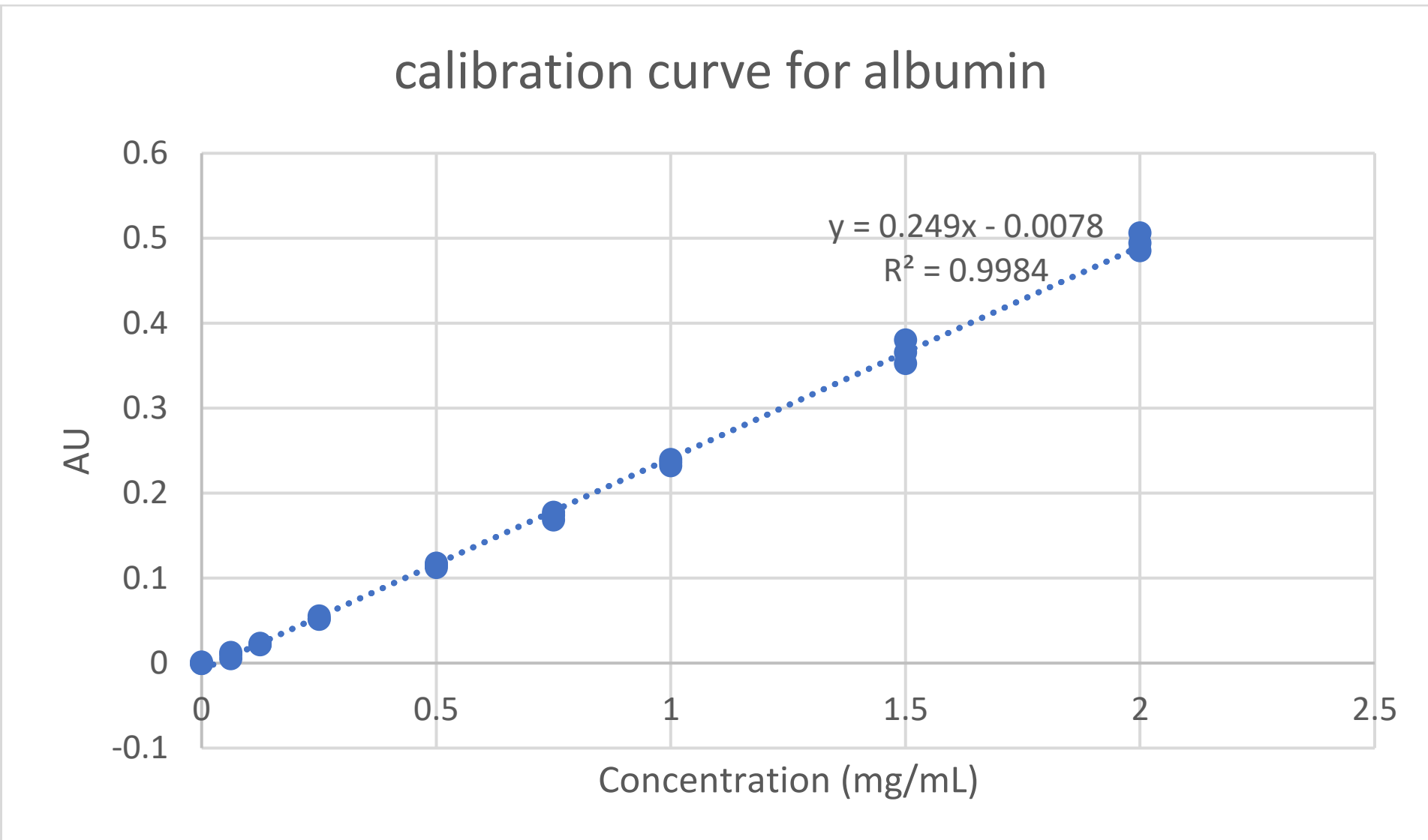

Obtained values

Sample	MW (g/mol)	dilution	measured value	Blank corrected	concentration (mg/mL)	concentration (uM)	average conc	sd
A9	36500	0.05	0.315	0.248333	1.028648	563.6427	561	14
A9	36500	0.05	0.306	0.239333	0.992503	543.8375		
A9	36500	0.05	0.321	0.254333	1.052744	576.8462		
B8	39920	0.1	0.373	0.306333	1.26158	316.027	313	71
B8	39920	0.1	0.455	0.388333	1.590897	398.5213		
B8	39920	0.1	0.283	0.216333	0.900134	225.4844		
C4	38030	0.1	0.509	0.442333	1.807764	475.3522	431	33
C4	38030	0.1	0.433	0.366333	1.502544	395.0943		
C4	38030	0.1	0.46	0.393333	1.610977	423.607		

Estimated TTN based on found enzyme concentration from protein purification.

Product	Enzyme	scale-up yield	product concentration (M)	TTN
**12**	A9	0.59	0.0015	76
**13**	B8	0.8	0.0020	391
**14**	C4	0.68	0.0017	322
**15**	C4	0.36	0.0009	85

Note: lysate of A9 and C4 were added twice, we calculated the TTN accordingly.

7. TTN and timecourse for production of 11

For all reactions 2.5 µL of enzyme stock was added to 200 µL solution unless noted. Concentrations of enzyme stocks are in Section 6.

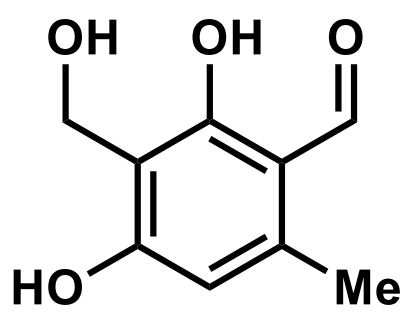

For formation of **11.** 4h timepoint.

Enzyme	average TTN	sd
NEC	0	
A9	70	2
B8	238	12
C4	58	6
CitB	31	1
ClaD	129	6

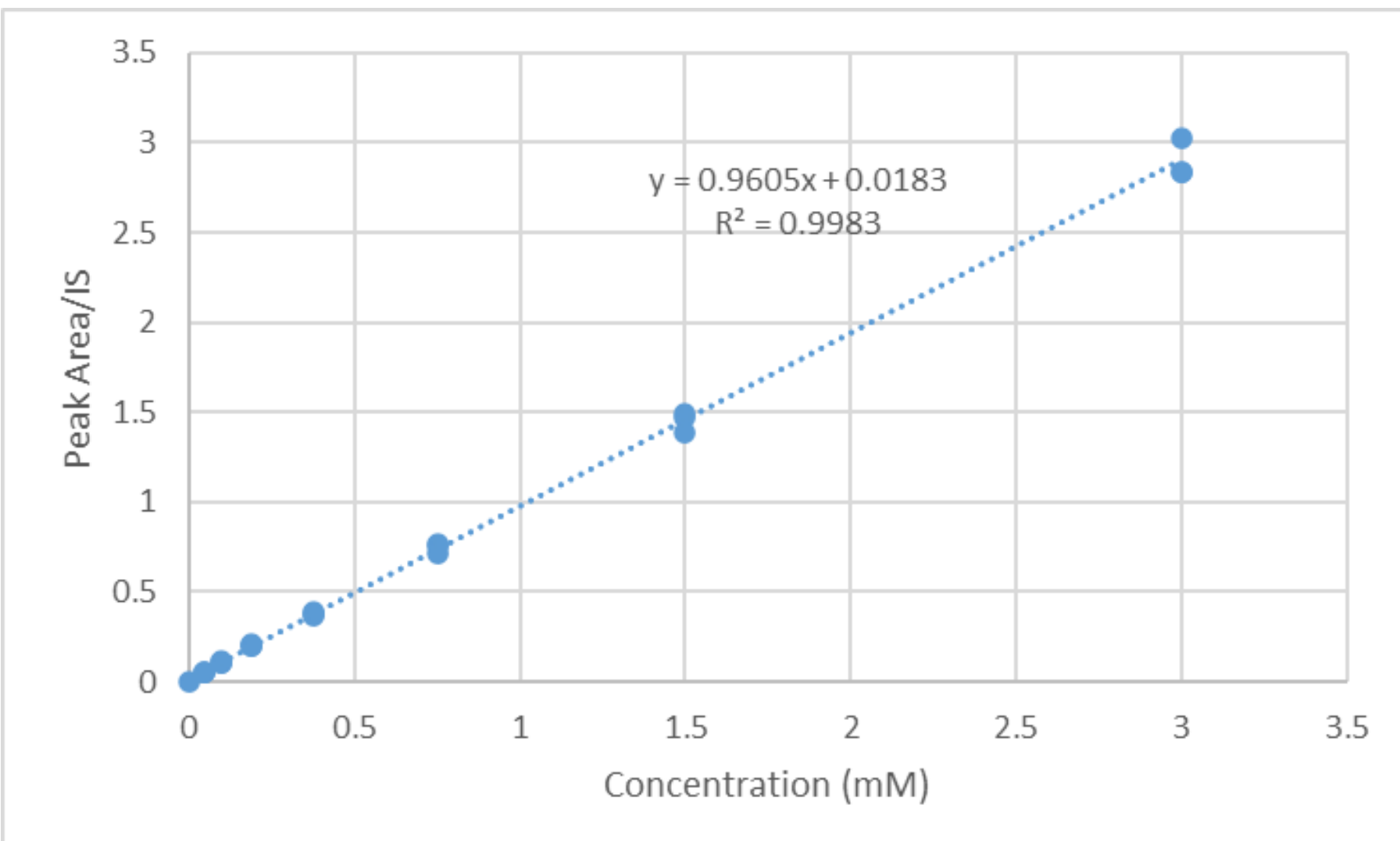

Raw data

enzyme	prod (2.93 min)	SM (3.5 min)	IS (3.96 min)	prod/IS	product concentration	TTN
NEC	0	588.3	294.5	0		
NEC	0	574.739	286.366	0		
NEC	0	588.6	291	0		
A9	140.738	307	301.2	0.467258	0.467421	66.77439
A9	146.4	276.2	296.1	0.494428	0.495708	70.81543
A9	153.1	217.5	305.4	0.50131	0.502873	71.83904
B8	284.5	40.6	297.071	0.957684	0.978015	250.7731
B8	273.6	67.7	294.9	0.927772	0.946874	242.7881
B8	254.3	11.1	299.8	0.848232	0.864063	221.5545
C4	106.147	320.627	310.357	0.342016	0.337028	62.41267
C4	84	343.8	303.4	0.276862	0.269195	49.85101
C4	101.6	310.1	305.9	0.332135	0.326741	60.50758
CitB	98.1	346.4	306.3	0.320274	0.314393	32.08089
CitB	92.7	366.5	299.8	0.309206	0.302869	30.90505
CitB	91.9	356.2	305.8	0.300523	0.293829	29.9826
ClaD	147	227.8	316.1	0.465043	0.465115	122.3986
ClaD	162.3	201	314.2	0.51655	0.51874	136.5106
ClaD	154.2	210	318.3	0.484449	0.485319	127.7155

Timecourse for production of **11**. Enzyme concentrations were 3.8 μM for this timecourse. Enzyme B8 has the highest initial rate.

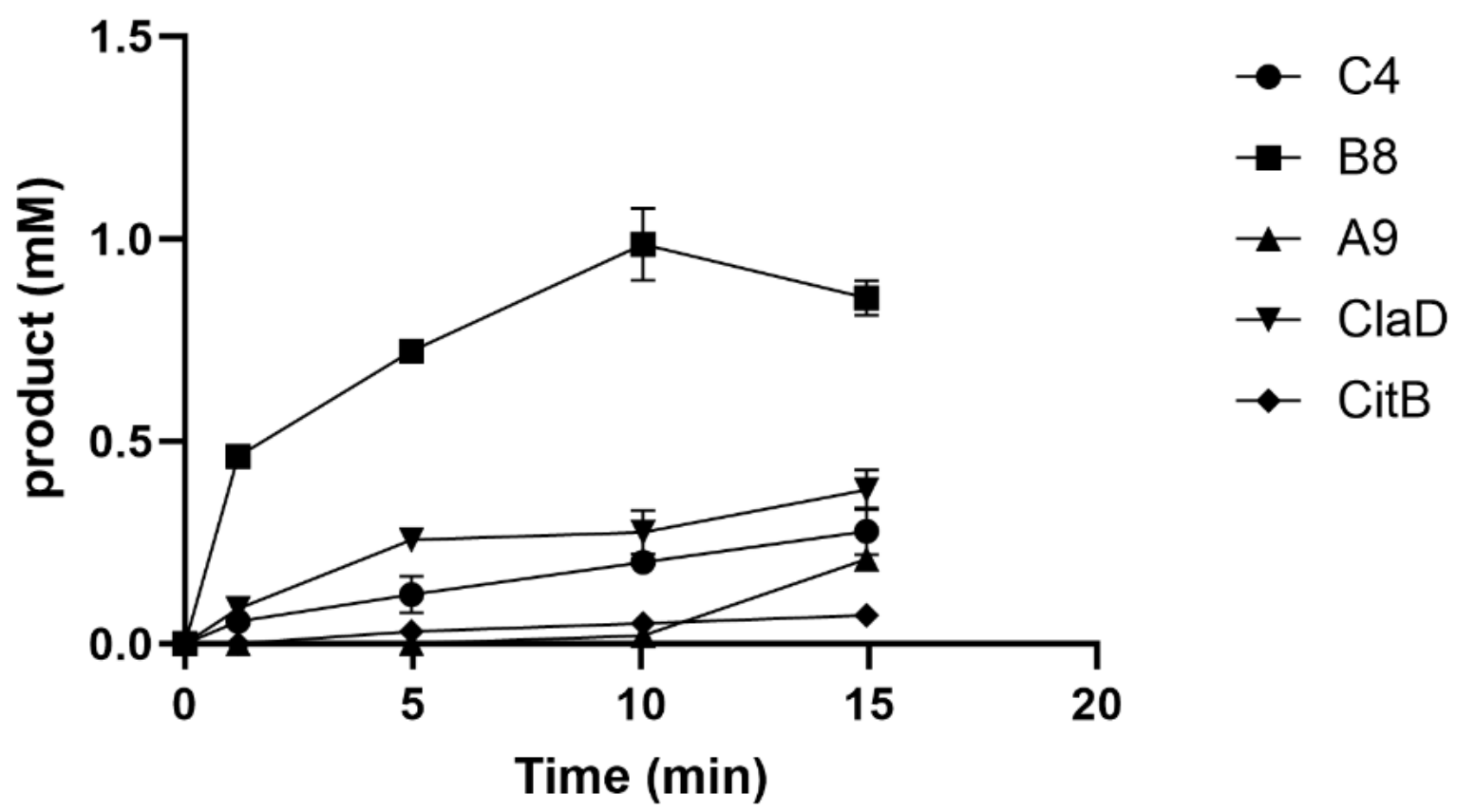

Calibration curve

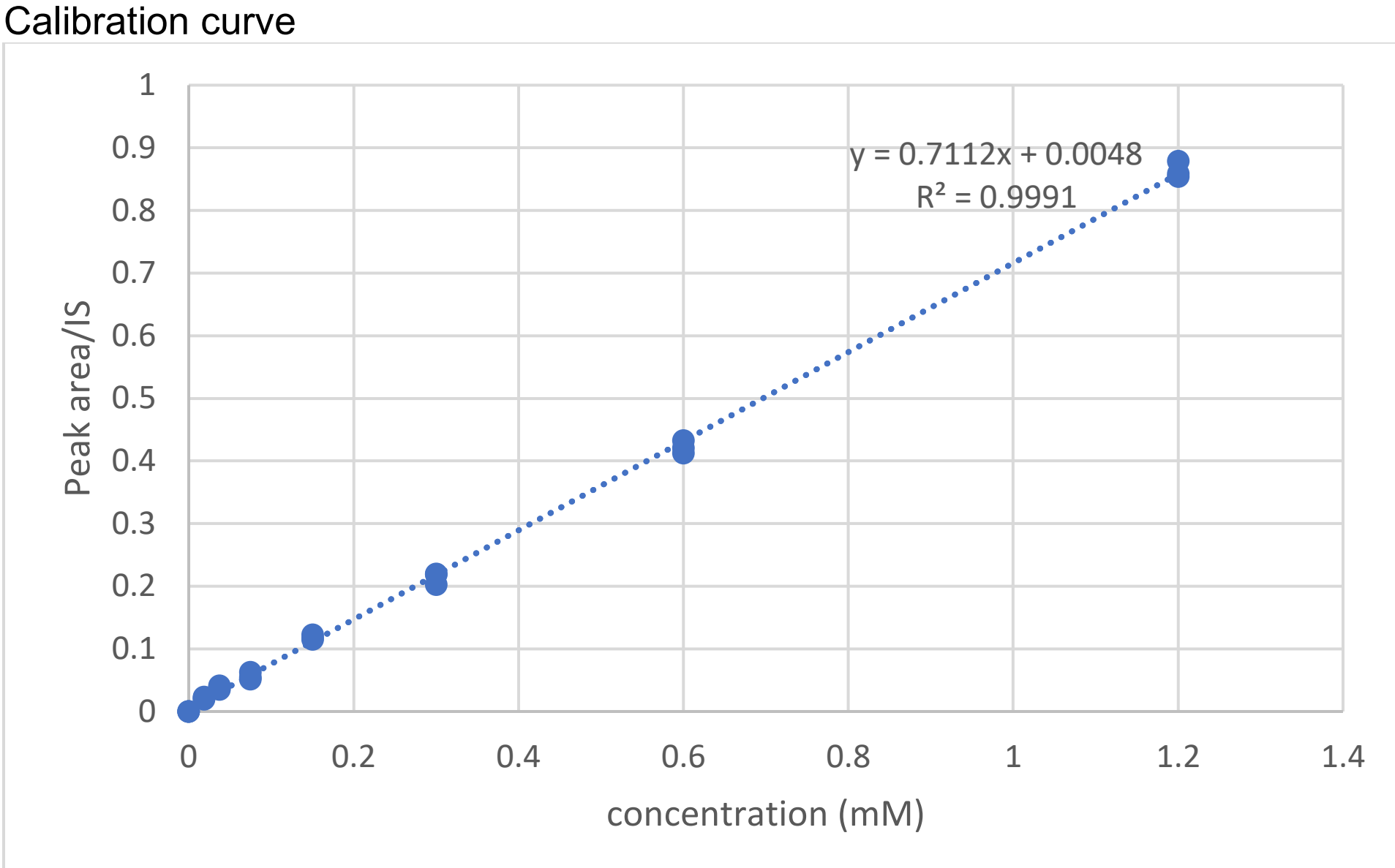

8. Chemical oxidations of target substrates

<u>Oxidation conditions</u>

AIBN, NBS:[7] In a 1 dram vial was placed 0.01 mmol of starting material, 0.01 mmol of N-bromosuccinimide (NBS) and 0.25 mL of benzene. The solution was degassed by bubbling nitrogen, then 1 mg of azobisisobutyronitrile (AIBN) was added. The reaction was closed and stirred for 12 – 16 h under blue LED irradation. The mixture was then evaporated and analized by NMR in the appropriate solvent. The sample was evaporated, dissolved in MeCN and analyzed by HPLC-DAD.

CAN:[8] In a 1 dram vial was placed 0.01 mmol of starting material, 0.02 mmol of ceric ammonium nitrate (CAN) and 0.25 mL of glacial acetic acid. The mixture was stirred at 60 °C for 12 – 16 h. The mixture was dissolved in water and extracted three times with EtOAc. The combined organic layers were dried with washed twice with brine, dried with Na_2SO_4, filtered and concentrated under reduced pressure. The obtained crude was dissolved in NMR-solvent and measured. Then it was evaporated, dissolved in MeCN and analyzed by HPLC-DAD.

$Na_2S_2O_8$:[9] In a 1 dram vial was placed 0.01 mmol of starting material, 0.01 mmol of $Na_2S_2O_8$ and 0.25 mL of DMSO. The solution was degassed by bubbling nitrogen and then it was stirred at 60 °C for 12 – 16 h. The mixture was dissolved in water and extracted three times with EtOAc. The combined organic layers were dried with washed twice with brine, dried with Na_2SO_4, filtered and concentrated under reduced pressure. The obtained crude was dissolved in NMR-solvent and measured. Then it was evaporated, dissolved in MeCN and analyzed by HPLC-DAD.

Ag_2O:[10] In a 1 dram vial was placed 0.01 mmol of substrate, 0.03 mmol of NaOMe, 0.015 mmol of Ag_2O and 0.25 mL of MeOH. The mixture was allowed to stir at room temperature for 12 – 16 h. Then, the mixture was filtered through a 0.22 µm filter and concentrated under reduced pressure. The obtained crude was dissolved in NMR-solvent and measured. Then it was evaporated, dissolved in MeCN and analyzed by HPLC-DAD.

MnO_2:[10] In a 1 dram vial was placed 0.01 mmol of substrate, 0.03 mmol of NaOMe, 0.015 mmol of MnO_2 and 0.25 mL of 1,2-dichloroethane. The mixture was allowed to stir at room temperature for 12 – 16 h. Then, the mixture was filtered through a 0.22 µm filter and concentrated under reduced pressure. The obtained crude was dissolved in NMR-solvent and measured. Then it was evaporated, dissolved in MeCN and analyzed by HPLC-DAD.

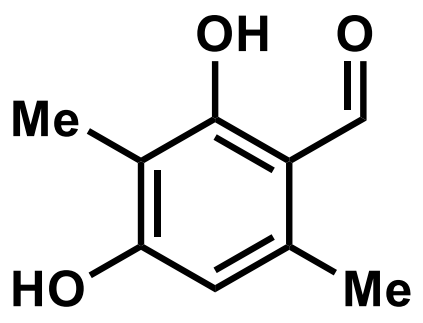

S8

Chemical oxidations on **S8** have been previously reported[11] (J. Am. Chem. Soc. 2019, 141, 20269−20277). The chemical methods did not selectively oxidize **S8** to produce **11**. We are reporting the obtained chromatograms and NMR's of the crude reactions.

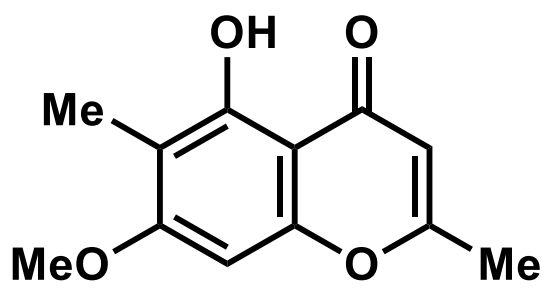

7

Conditions[7–10]	Result
Enzyme A9	Benzylic alcohol
AIBN, NBS	Several products produced
CAN	decomposition
$Na_2S_2O_8$	Several products produced, low conversion
Ag_2O	Starting material
MnO_2	Starting material

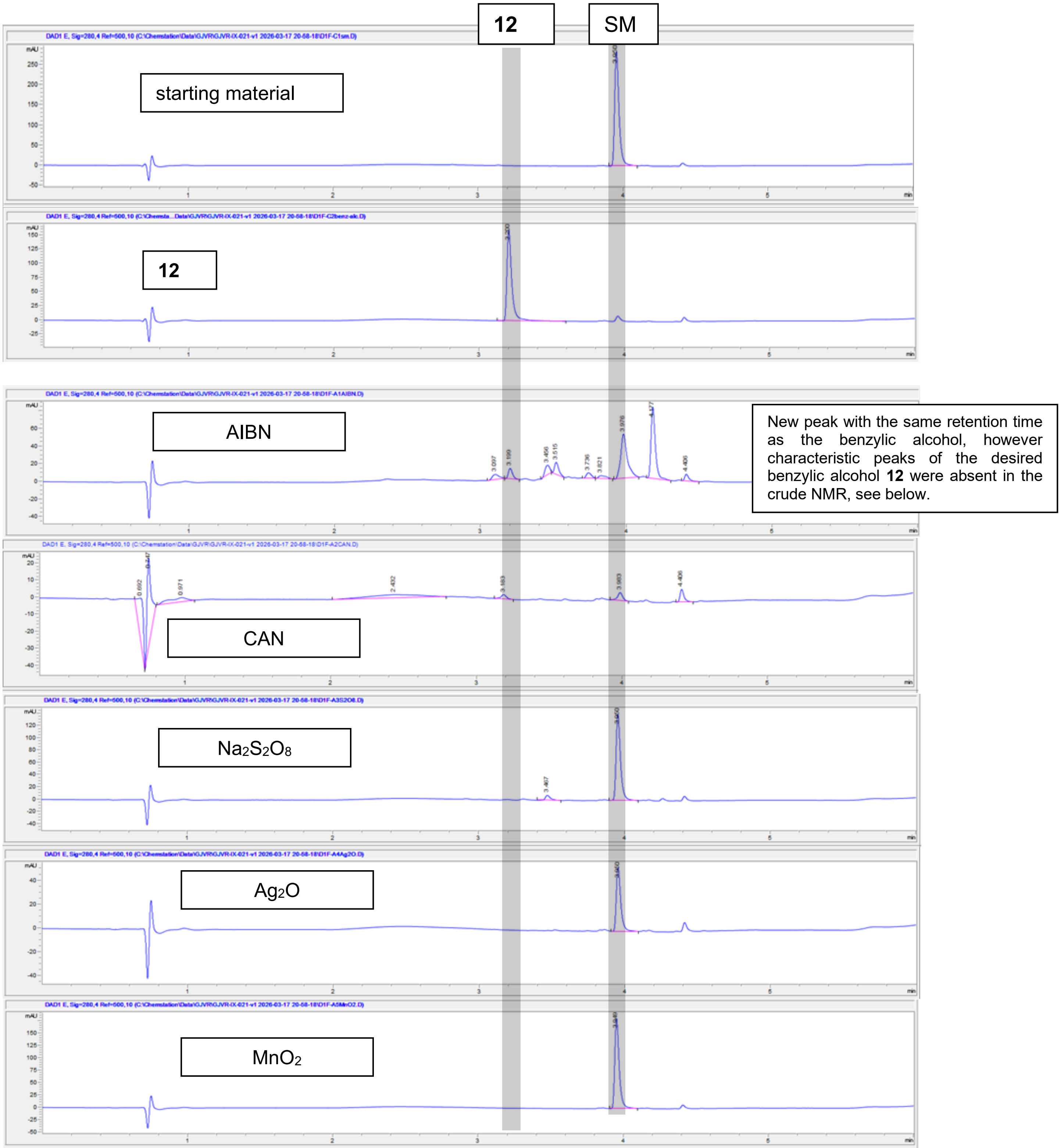
12
SM
starting material
12
AIBN
New peak with the same retention time as the benzylic alcohol, however characteristic peaks of the desired benzylic alcohol 12 were absent in the crude NMR, see below.
CAN
Na2S2O8
Ag2O
MnO2

Enzymatic reaction with A9

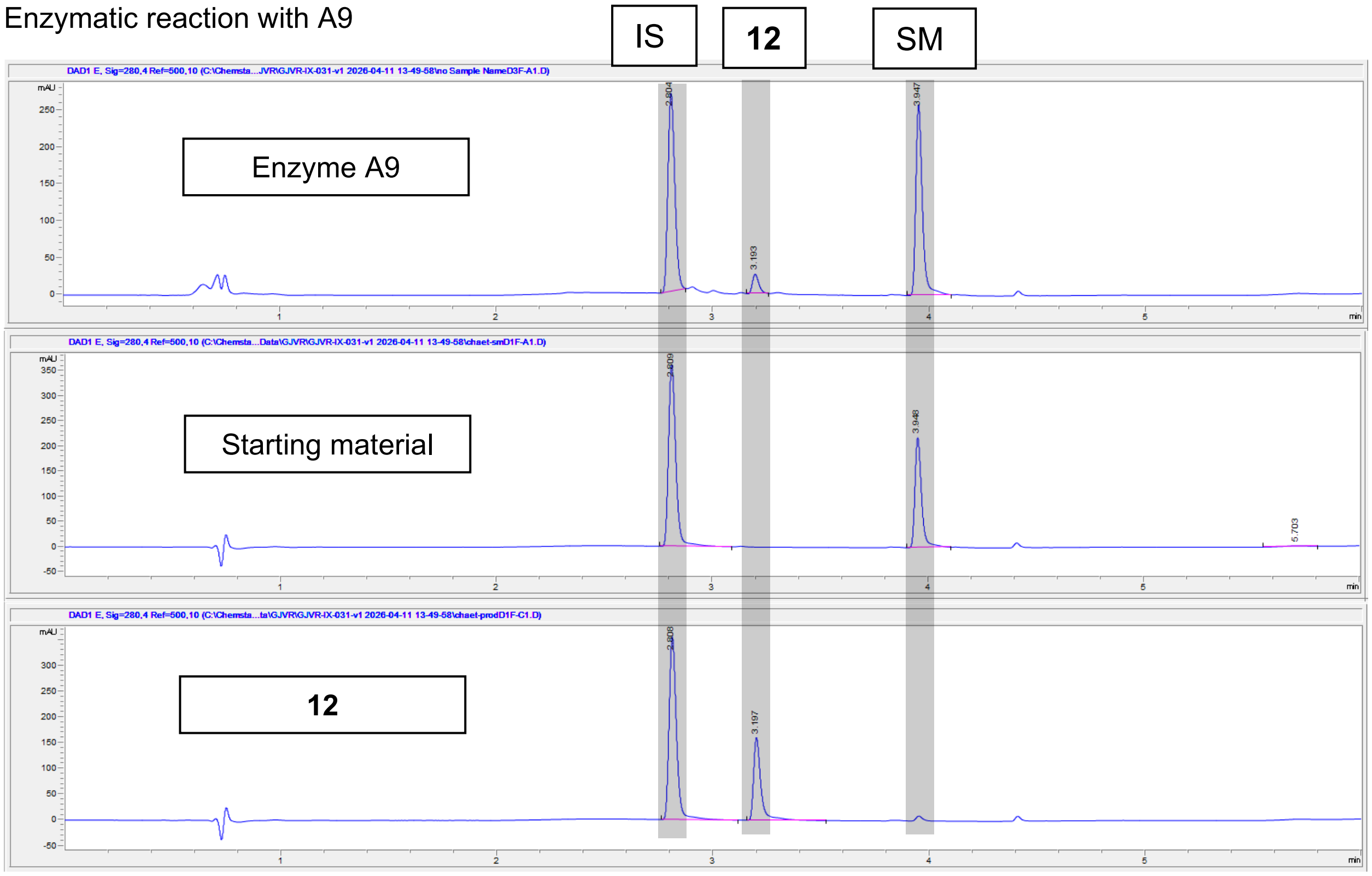

For the NMR we highlighted both the solvent ($CDCl_3$) and the internal standard peak from dibromomethane

AIBN, NBS
^{1}H-NMR (599 MHz, $CDCl_3$)

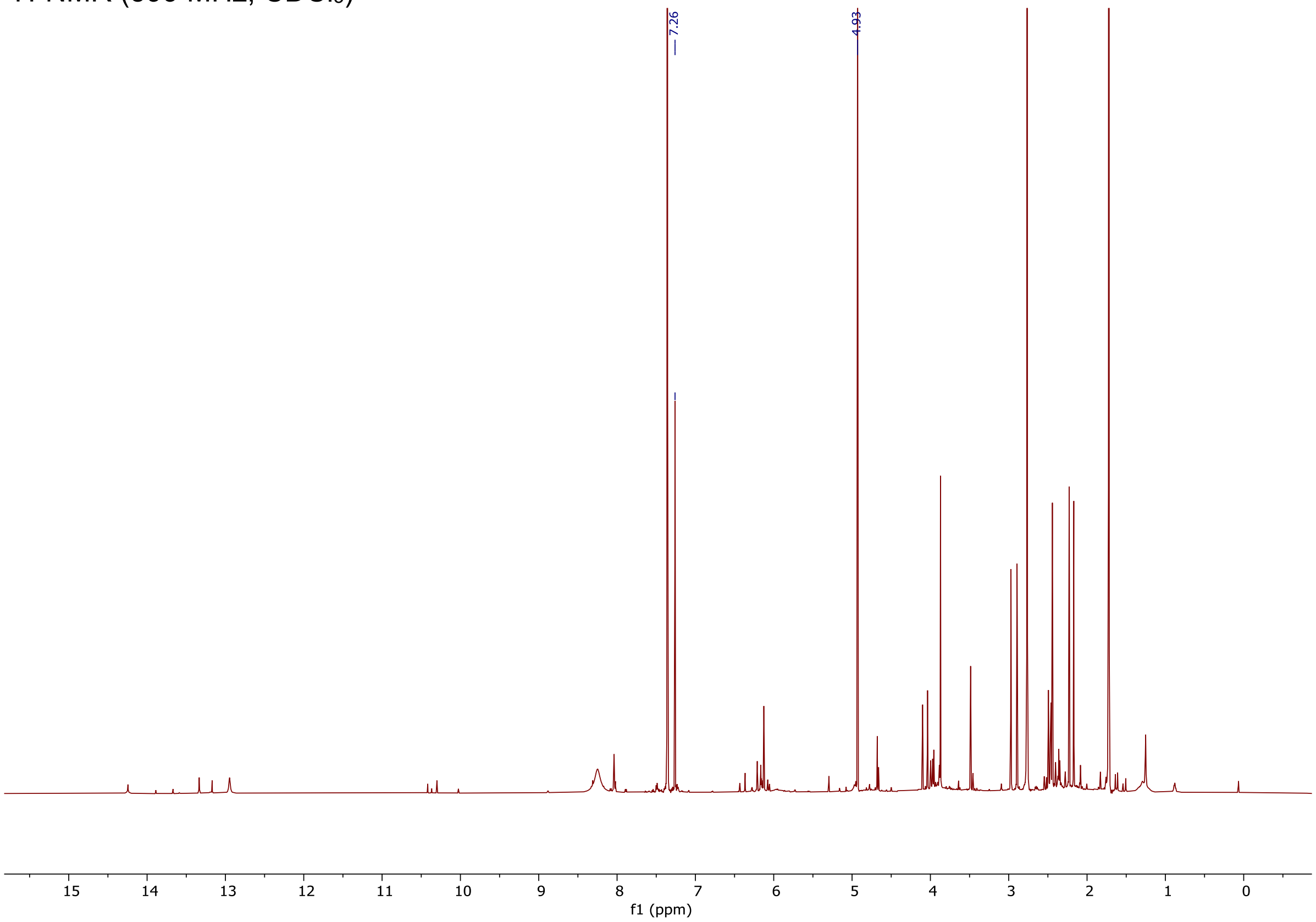

CAN
^{1}H-NMR (599 MHz, $CDCl_3$)

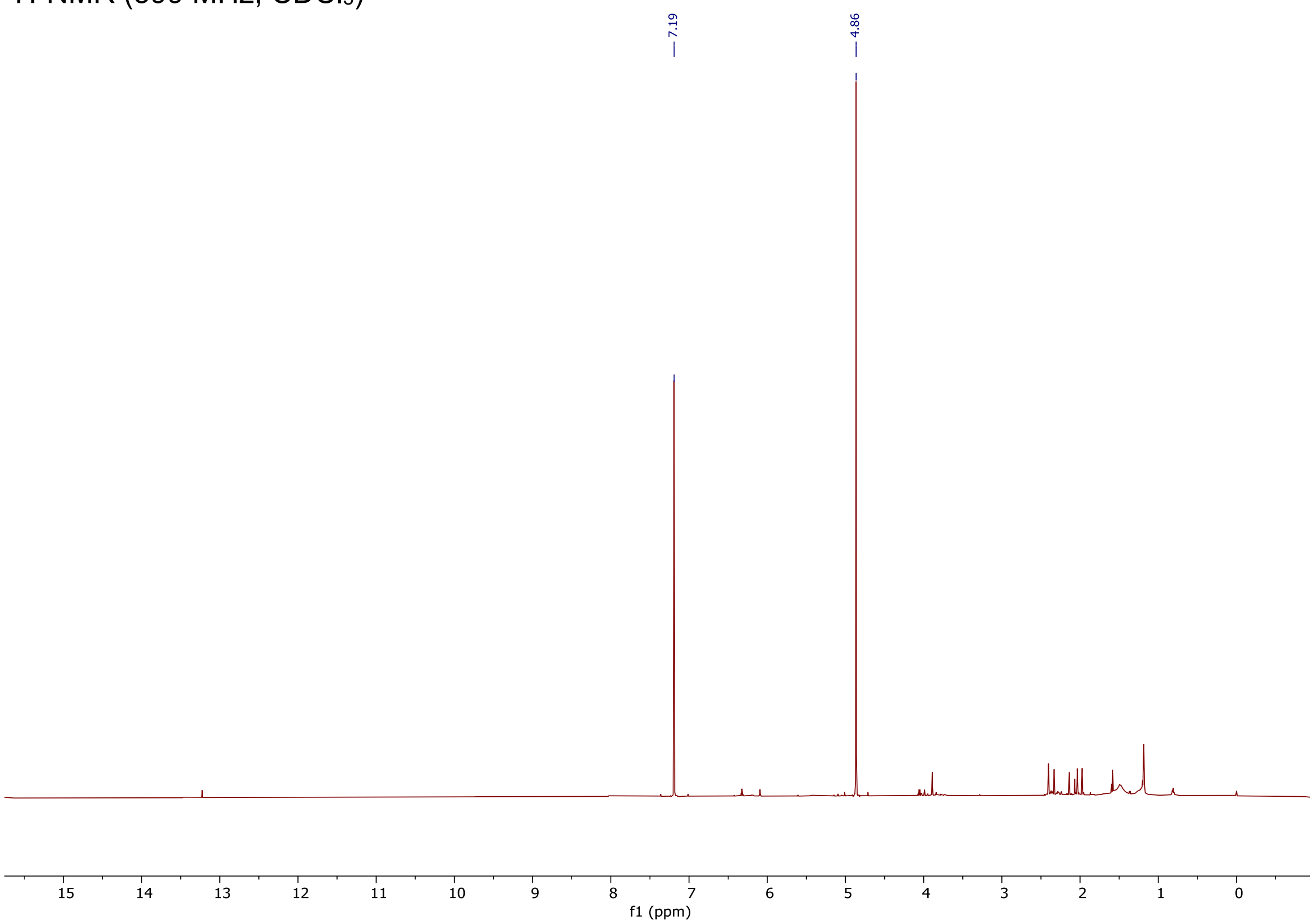

$Na_2S_2O_8$
^{1}H-NMR (400 MHz, $CDCl_3$)

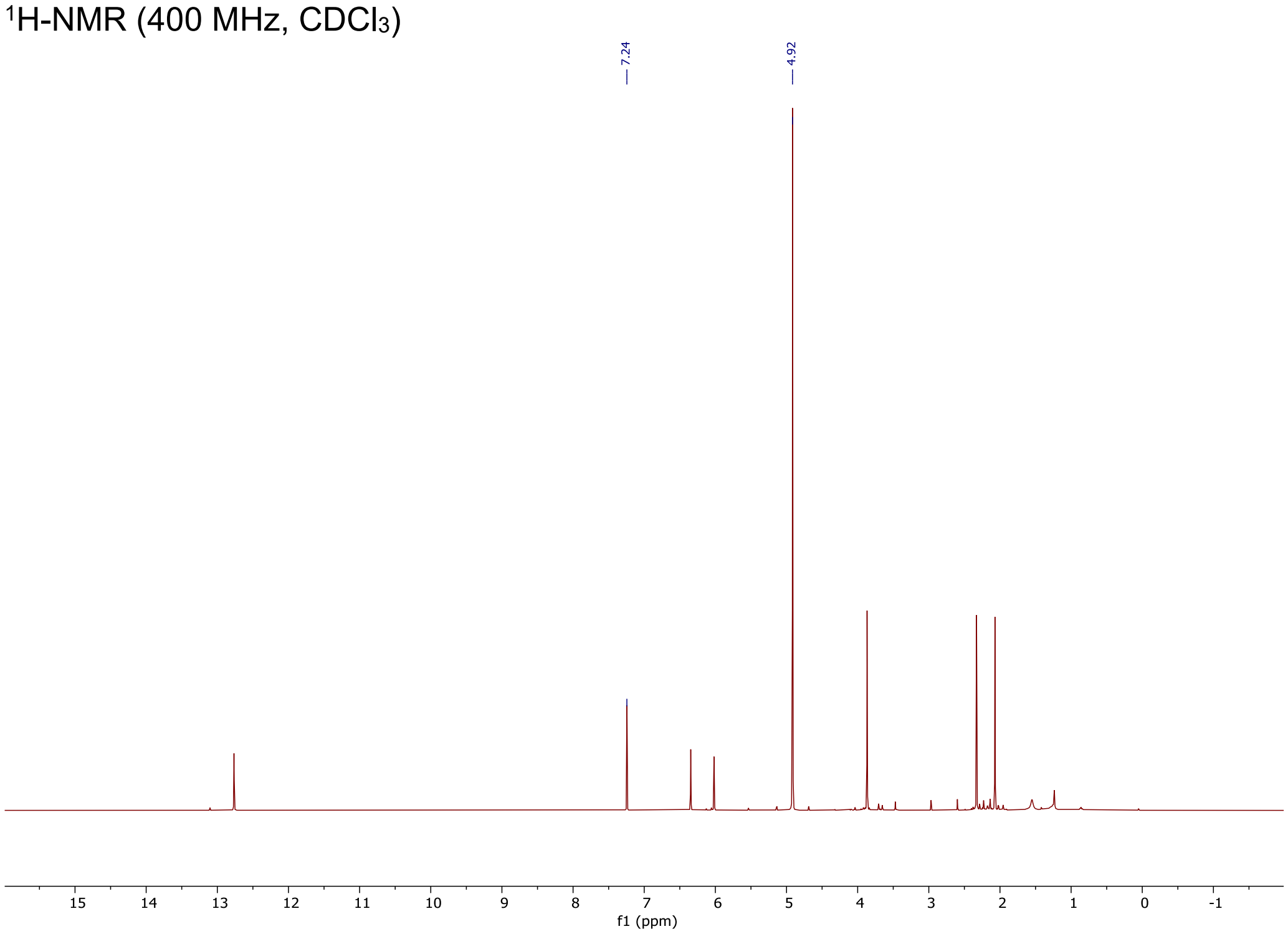

Ag_2O

^{1}H-NMR (400 MHz, $CDCl_3$)

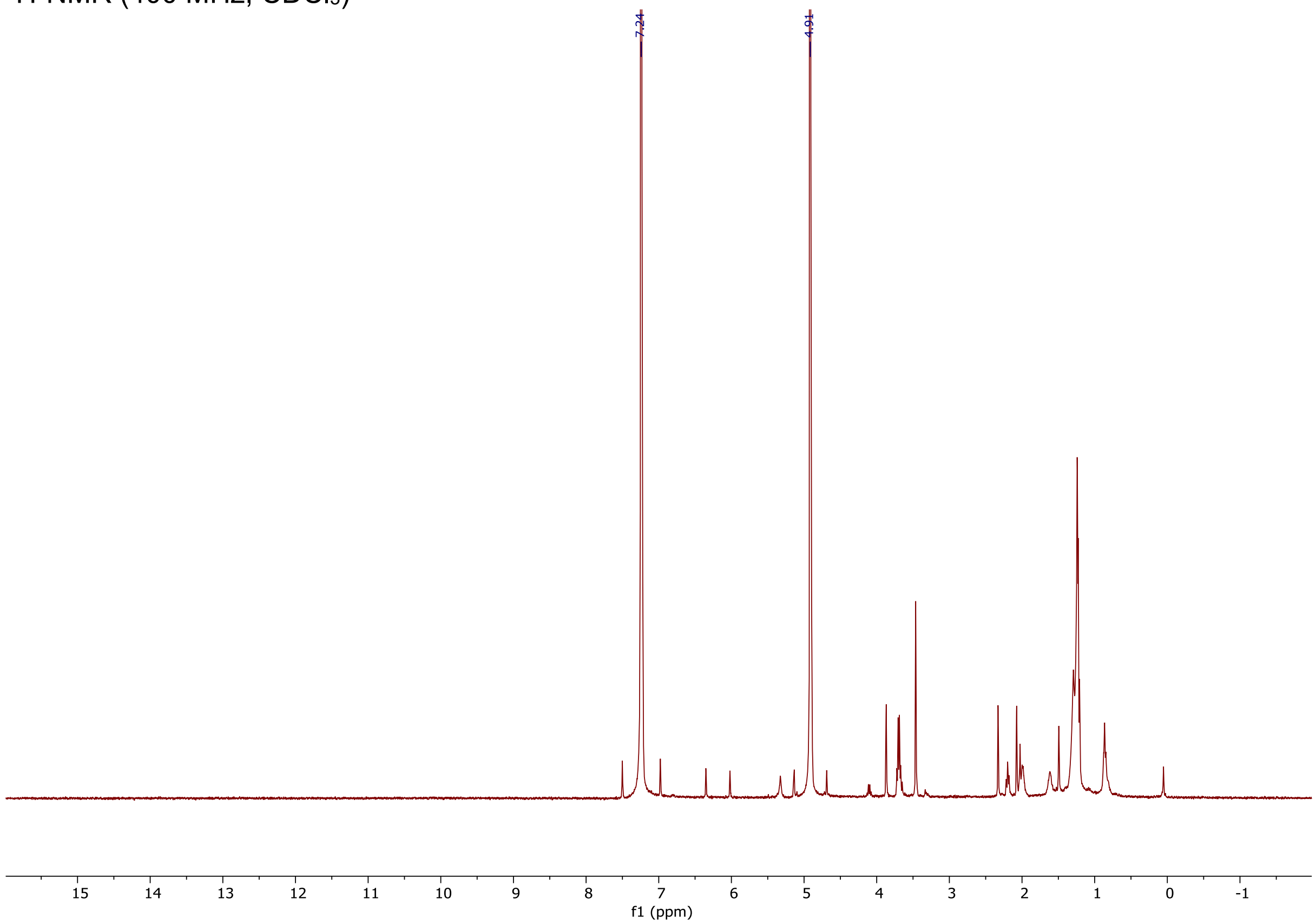

MnO_2

^{1}H-NMR (400 MHz, $CDCl_3$)

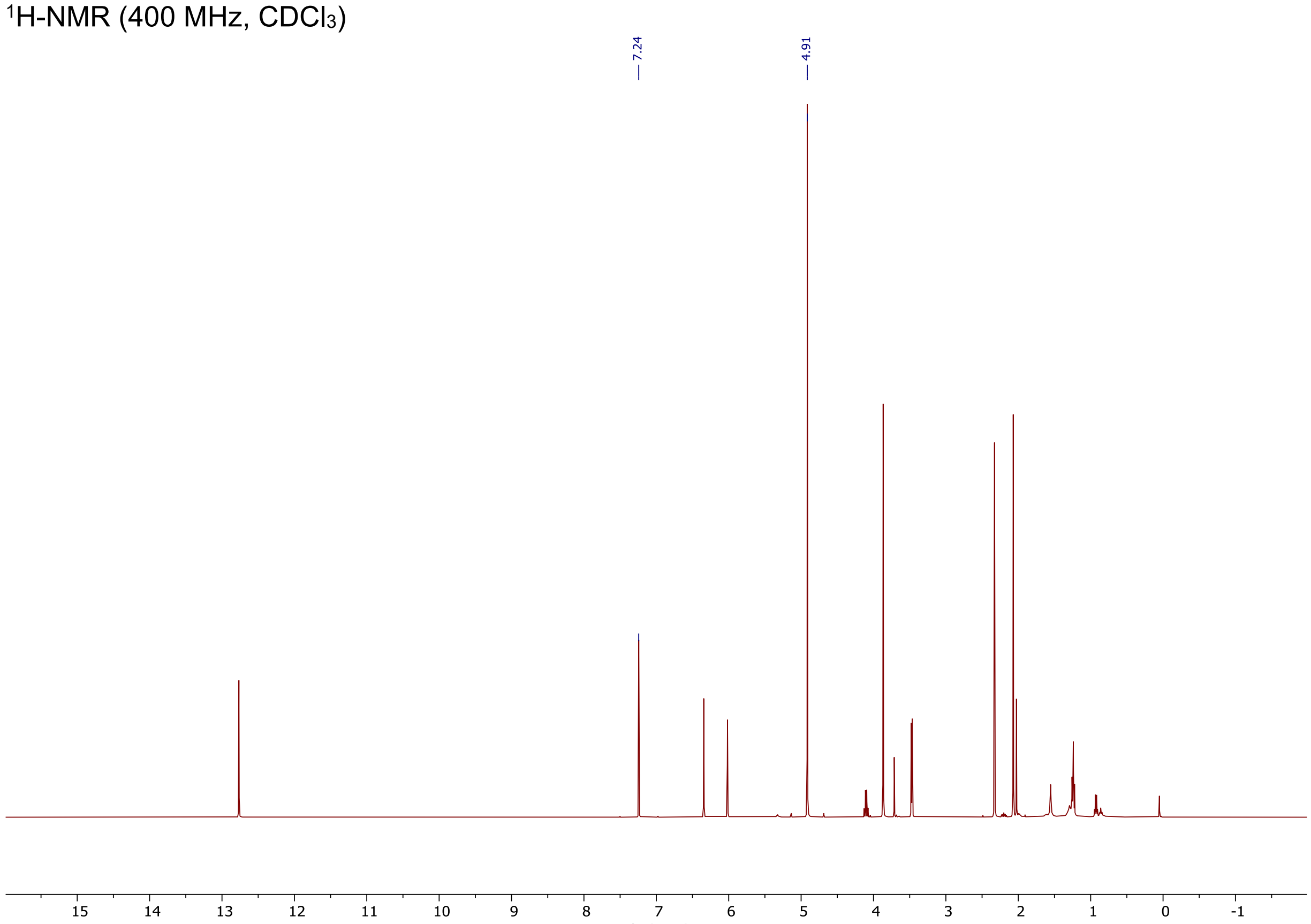

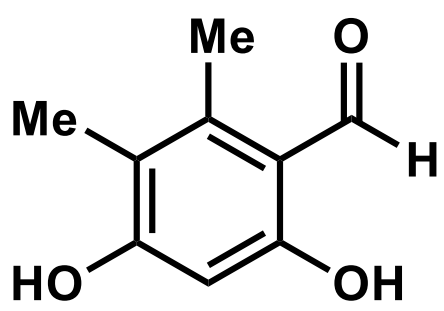

8

Conditions[7–10]	Result
Enzyme B8	Benzylic alcohol
AIBN, NBS	Several products produced, none are benzylic alcohol
CAN	Decomposition
$Na_2S_2O_8$	At least one new product, not benzylic alcohol
Ag_2O	At least two new compounds, not the benzylic alcohol
MnO_2	Starting material

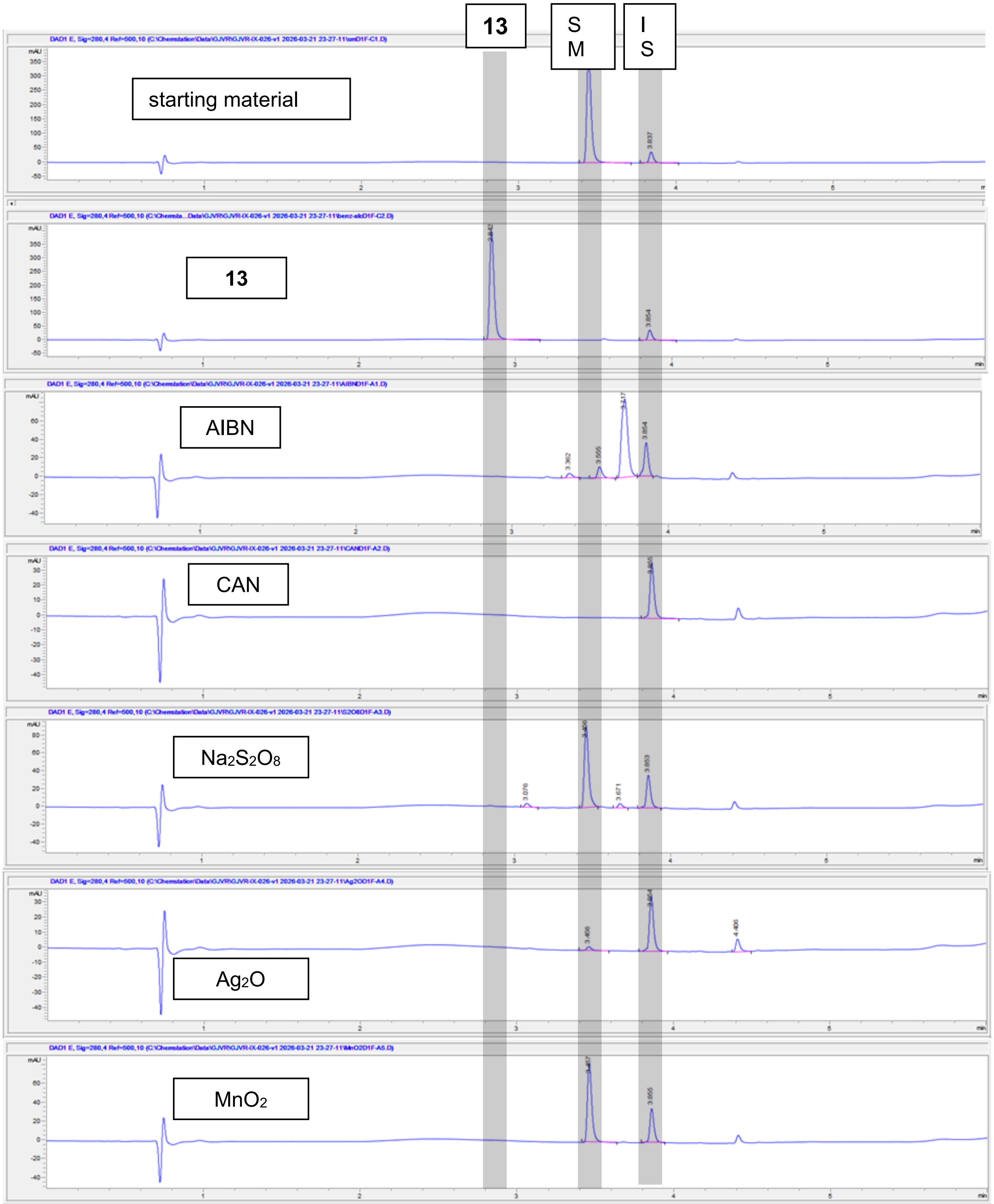
13
S
M
I
S
starting material
13
AIBN
CAN
Na2S2O8
Ag2O
MnO2

Reaction with B8

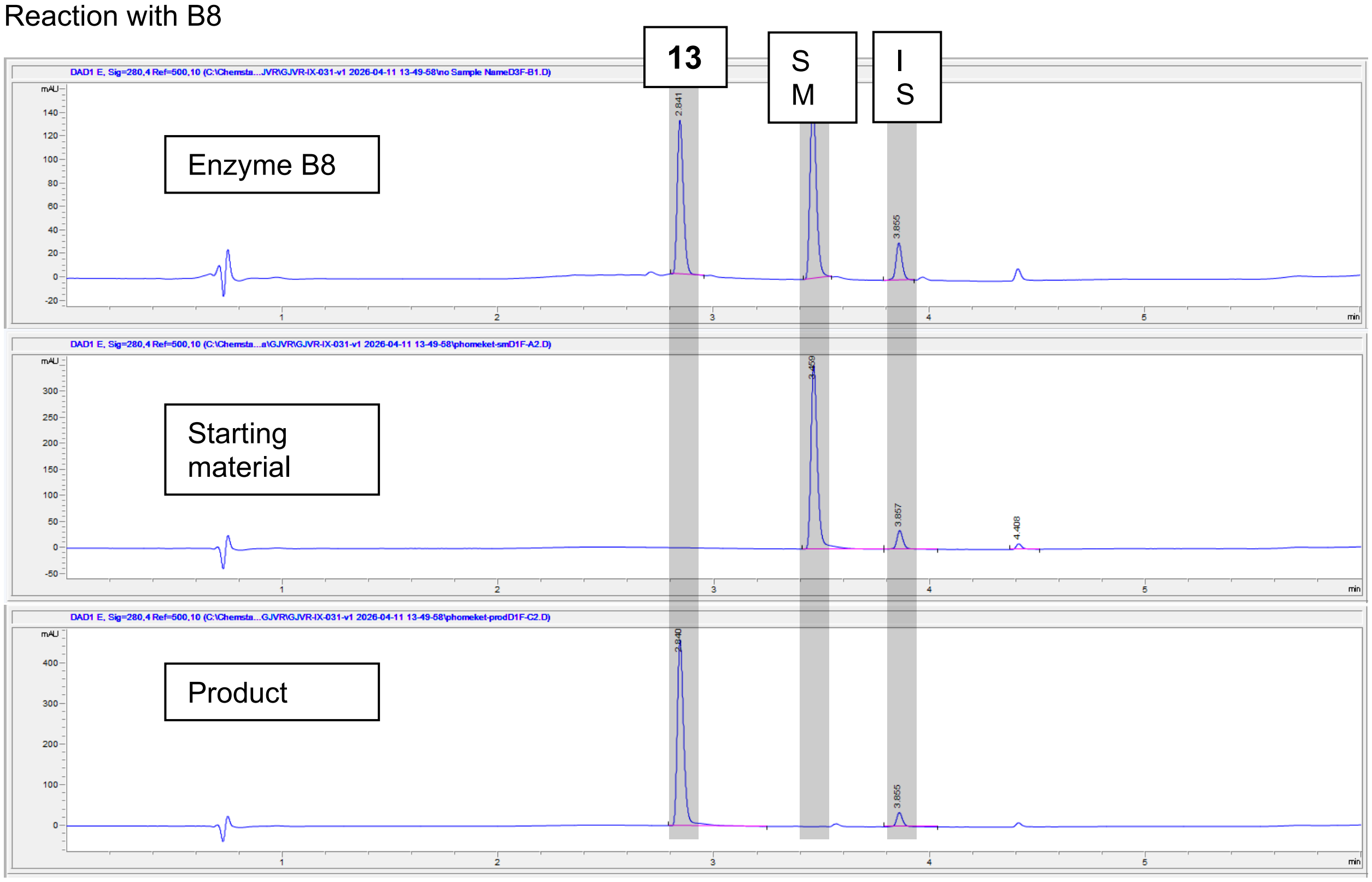

Highlighted peaks in crude NMR's are solvent (d_4-mehtanol) and internal standard (MeCN)
AIBN, NBS
^{1}H-NMR (599 MHz, d_4-MeOH)

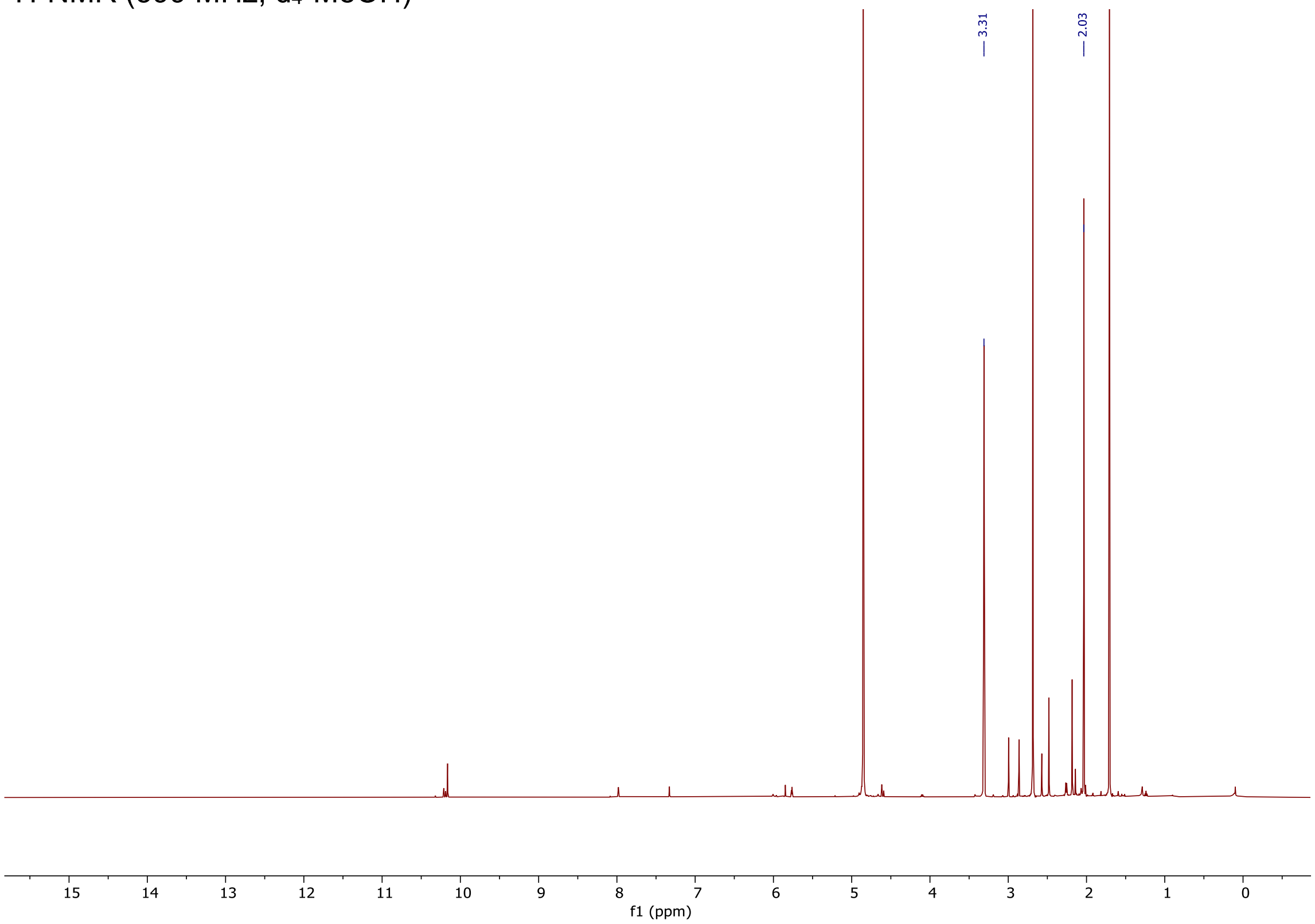

CAN
^{1}H-NMR (599 MHz, d_4-MeOH)

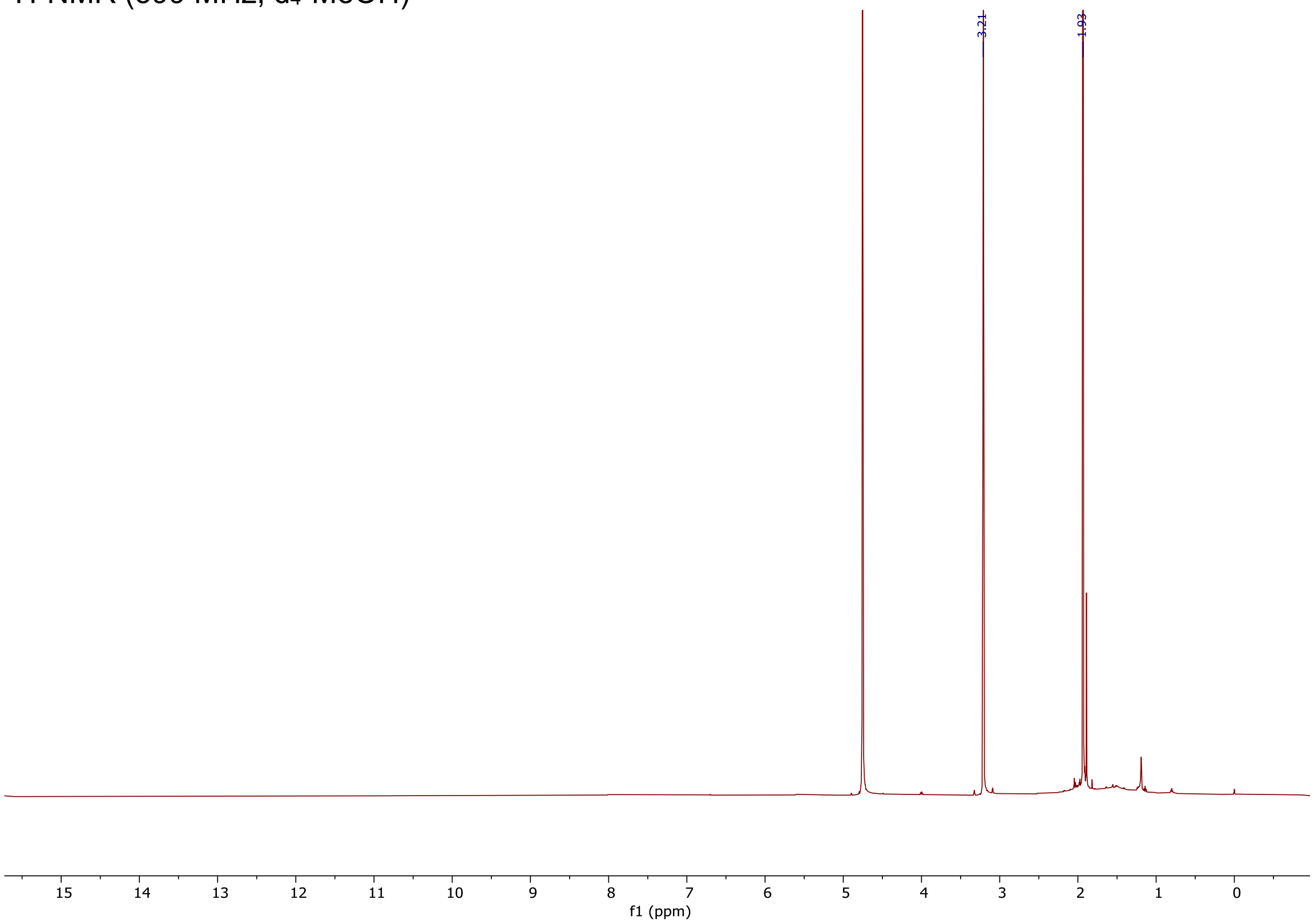

$Na_2S_2O_8$, Mainly remaining starting material, at least two new products, not benzylic alcohol **13**.
^{1}H-NMR (599 MHz, d_4-MeOH)

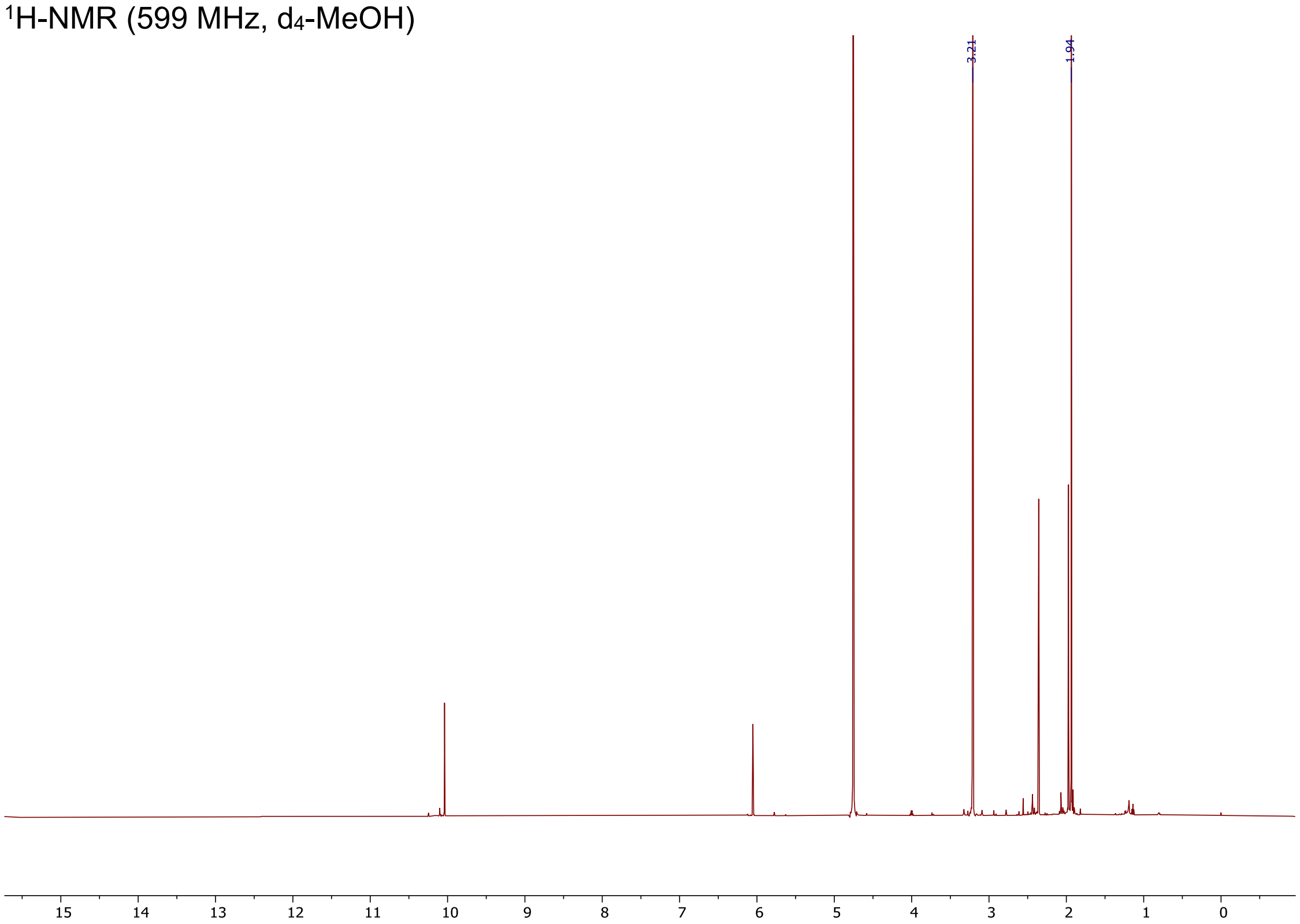

Ag$_2$O
^{1}H-NMR (599 MHz, d$_4$-MeOH)

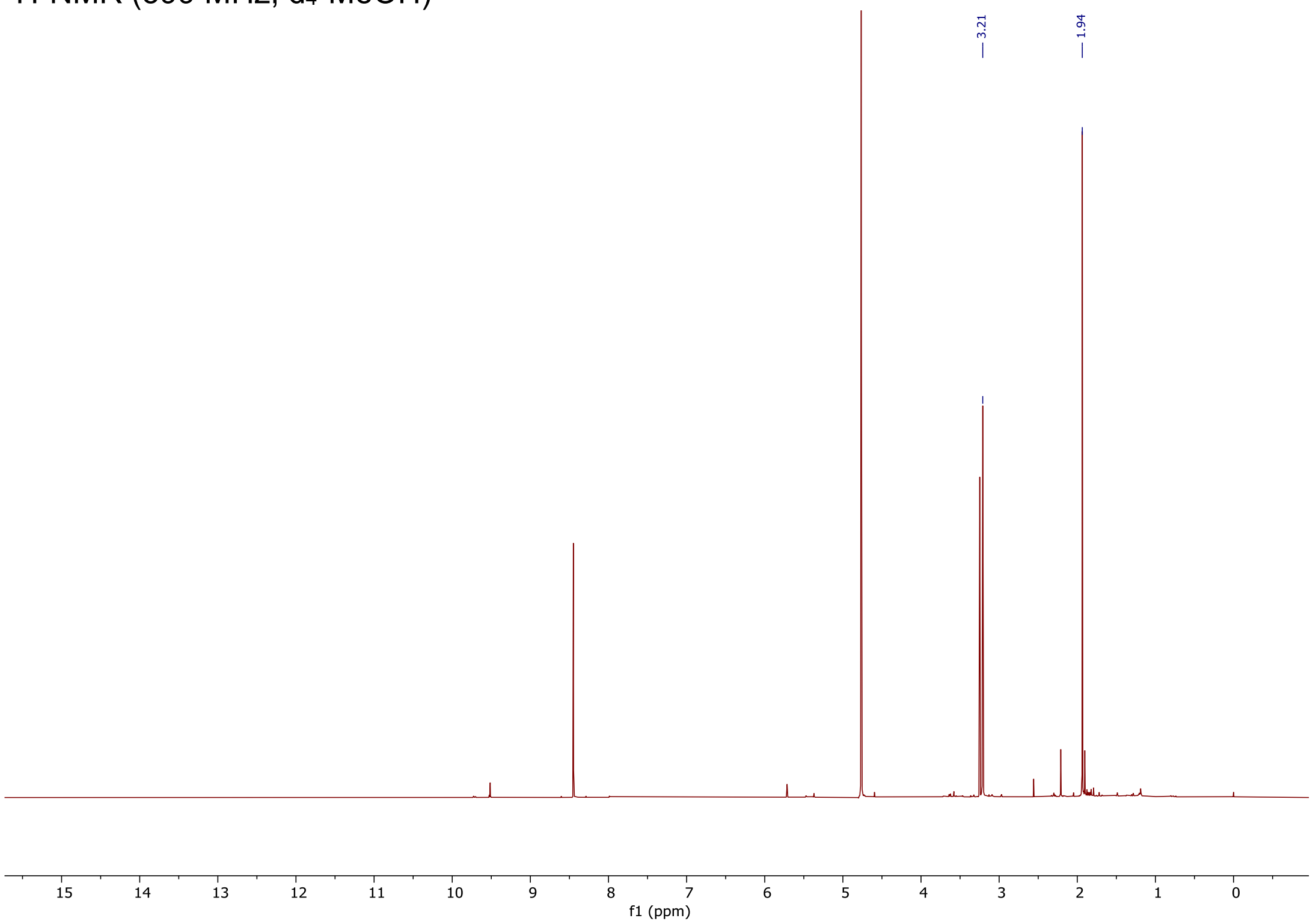

MnO$_2$
^{1}H-NMR (599 MHz, d$_4$-MeOH)

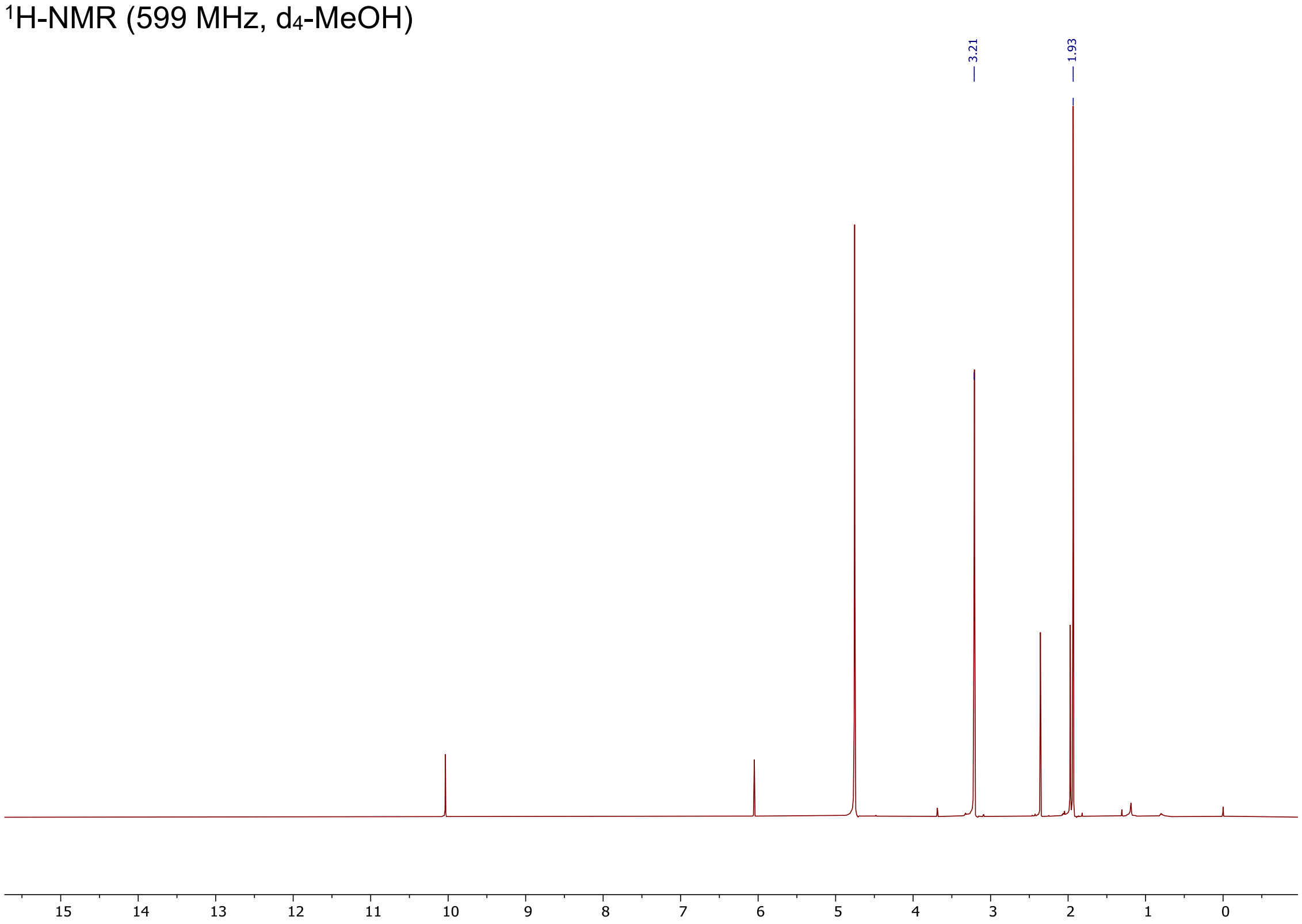

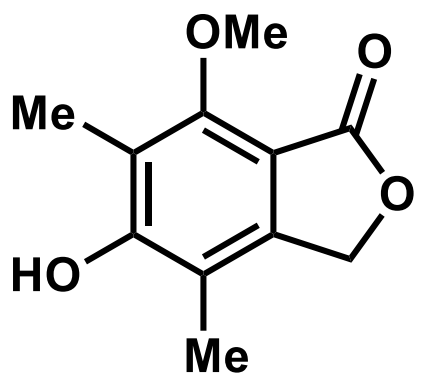

9

Condition	Result
Enzyme C4	Benzylic alcohol
AIBN, NBS	Several products produced, none are benzylic alcohol
CAN	decomposition
$Na_2S_2O_8$	One new product, not desired benzylic alcohol
Ag_2O	New product, not benzylic alcohol
MnO_2	Starting material

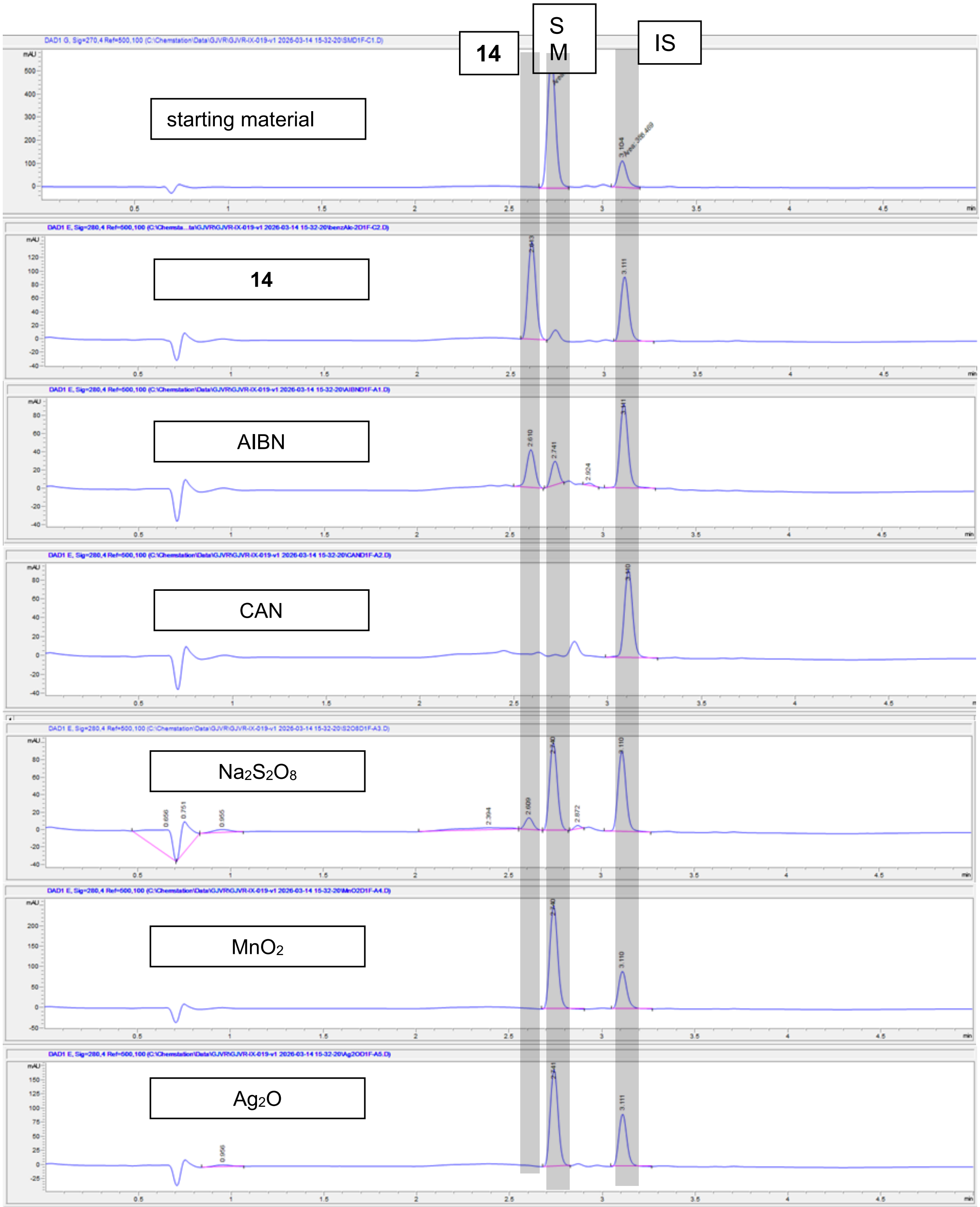
14
SM
IS
starting material
14
AIBN
CAN
Na2S2O8
MnO2
Ag2O

Enzymatic reaction with C4

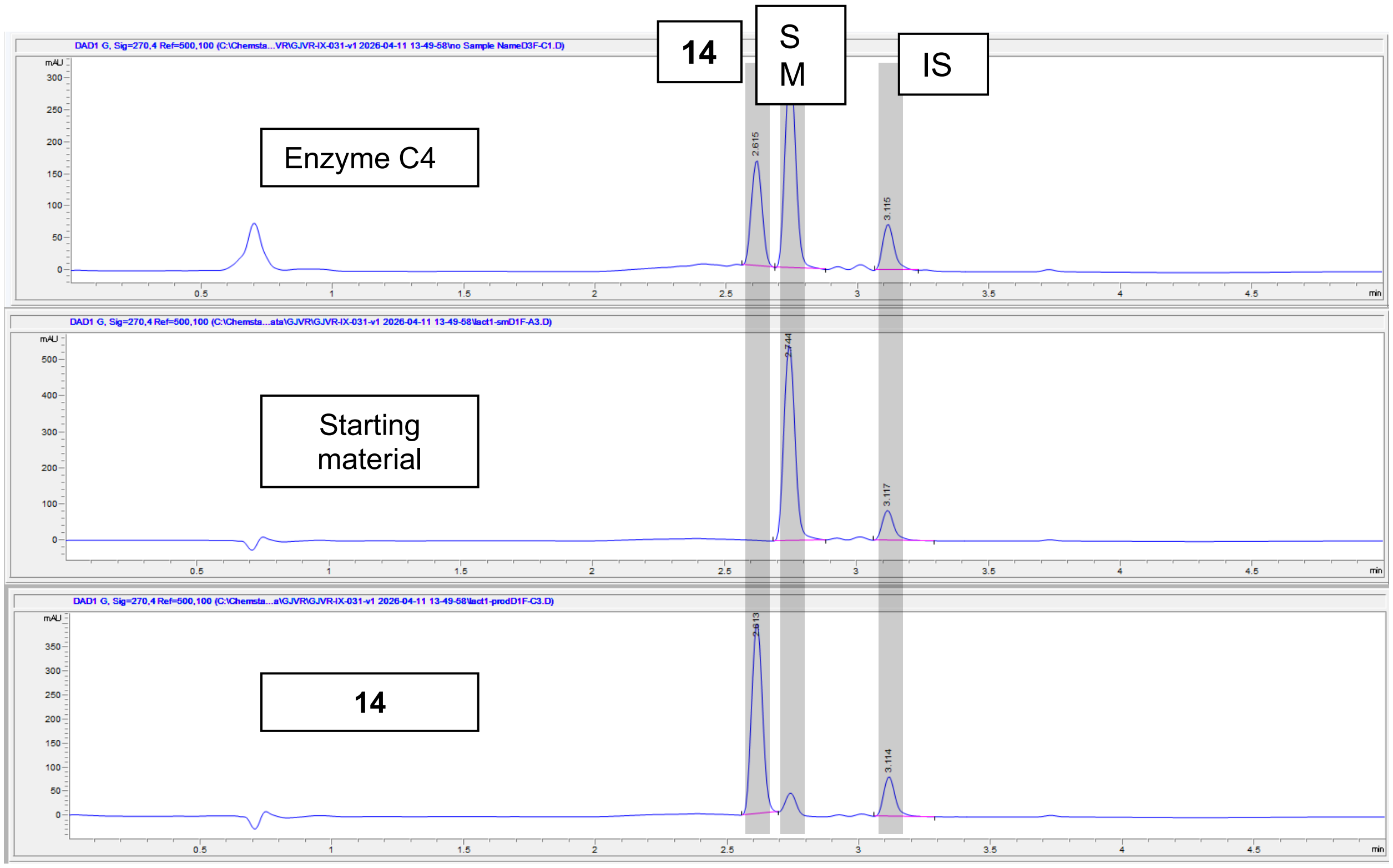

Both reactions with AIBN and $Na_2S_2O_8$ show a peak with the same retention time as the desired benzylic alcohol.

Crude NMR of AIBN, NBS reaction. Several methyl peaks (2.00 - 2.5- ppm) suggest there is at least 3 products. We could not find all the peaks of benzylic alcohol **14** in this crude.

AIBN, NBS
^{1}H-NMR (599 MHz, d_6-acetone)

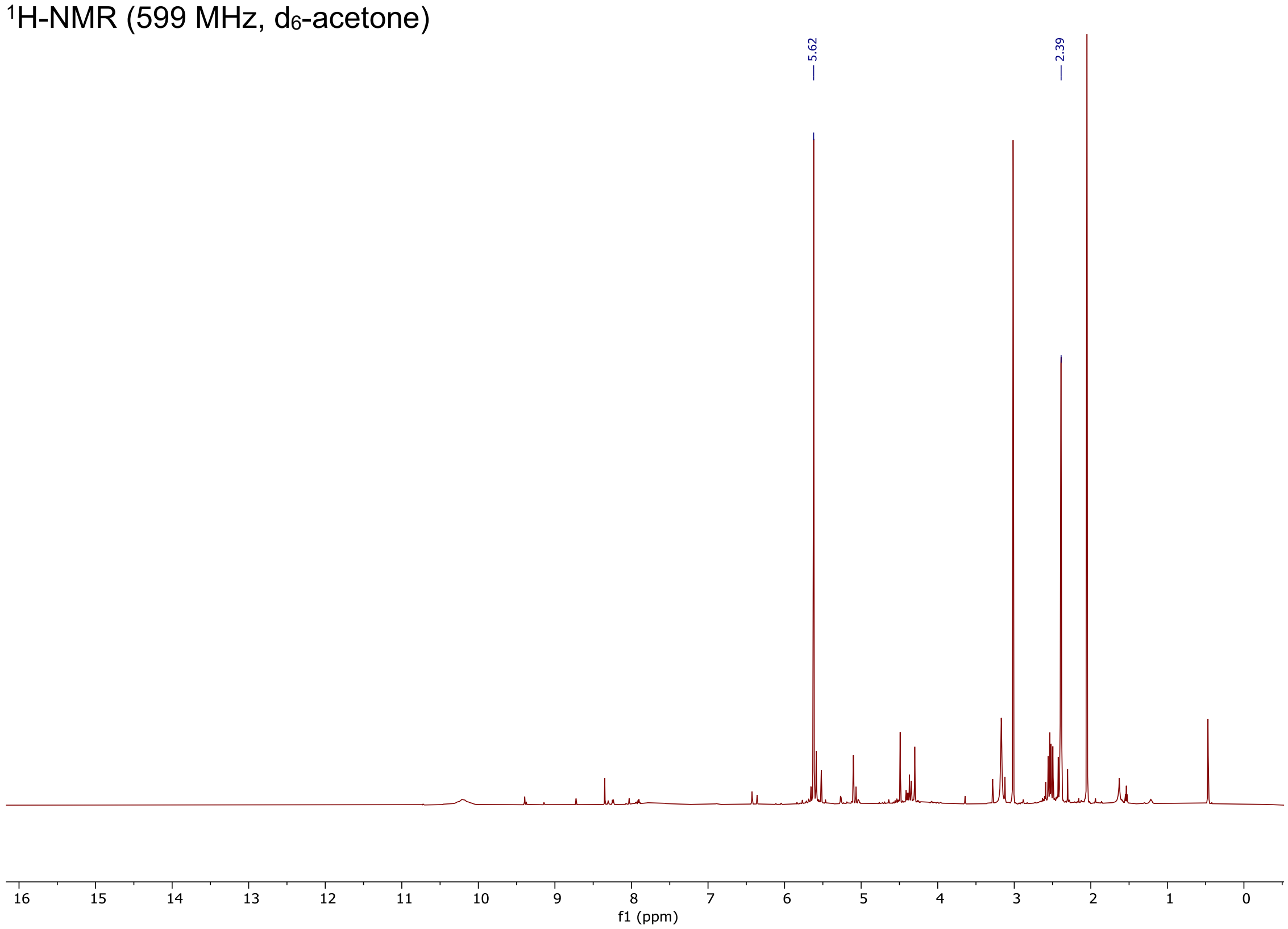

CAN
^1H-NMR (599 MHz, d_6-acetone)

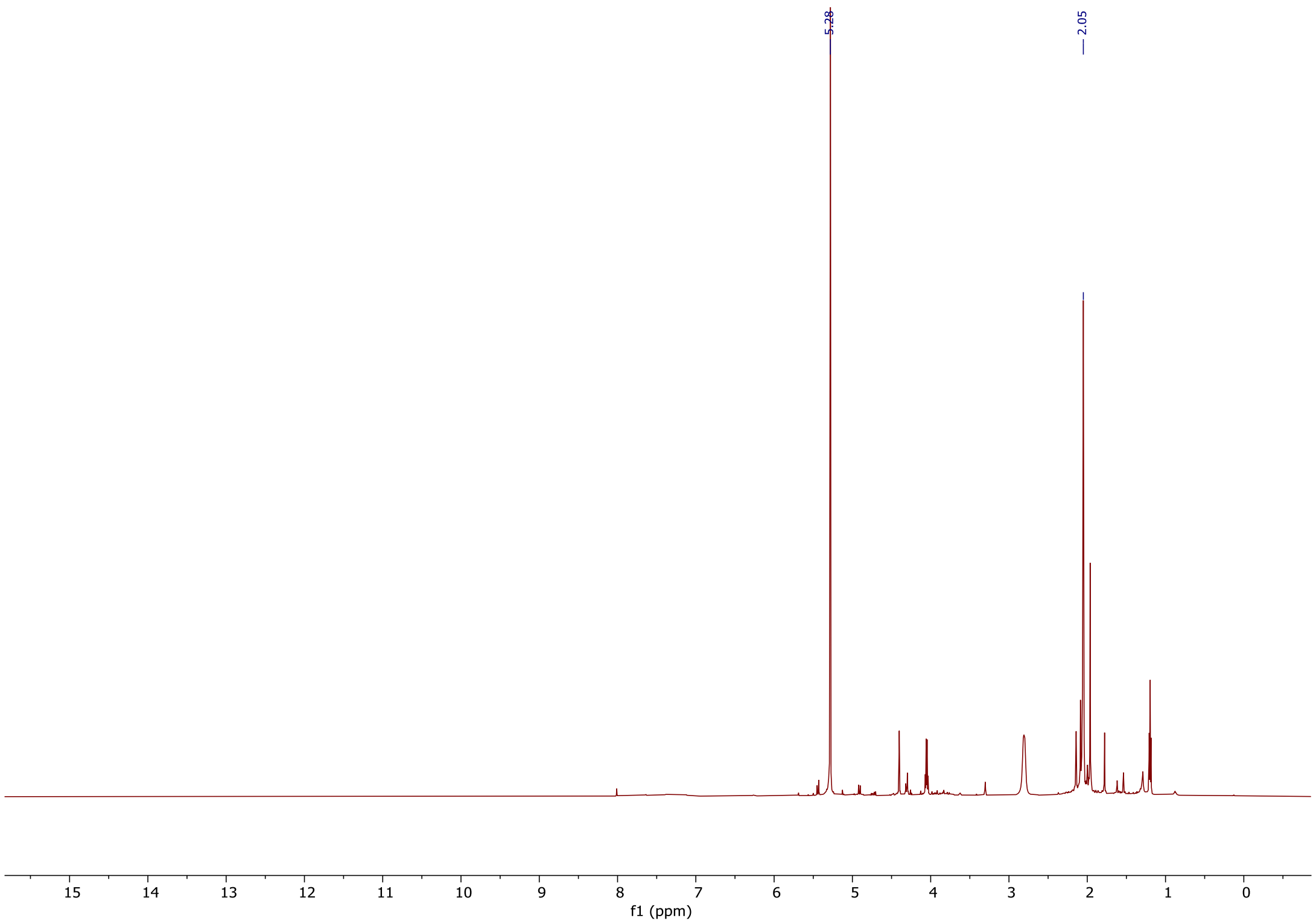

$Na_2S_2O_8$
^1H-NMR (599 MHz, d_6-acetone)

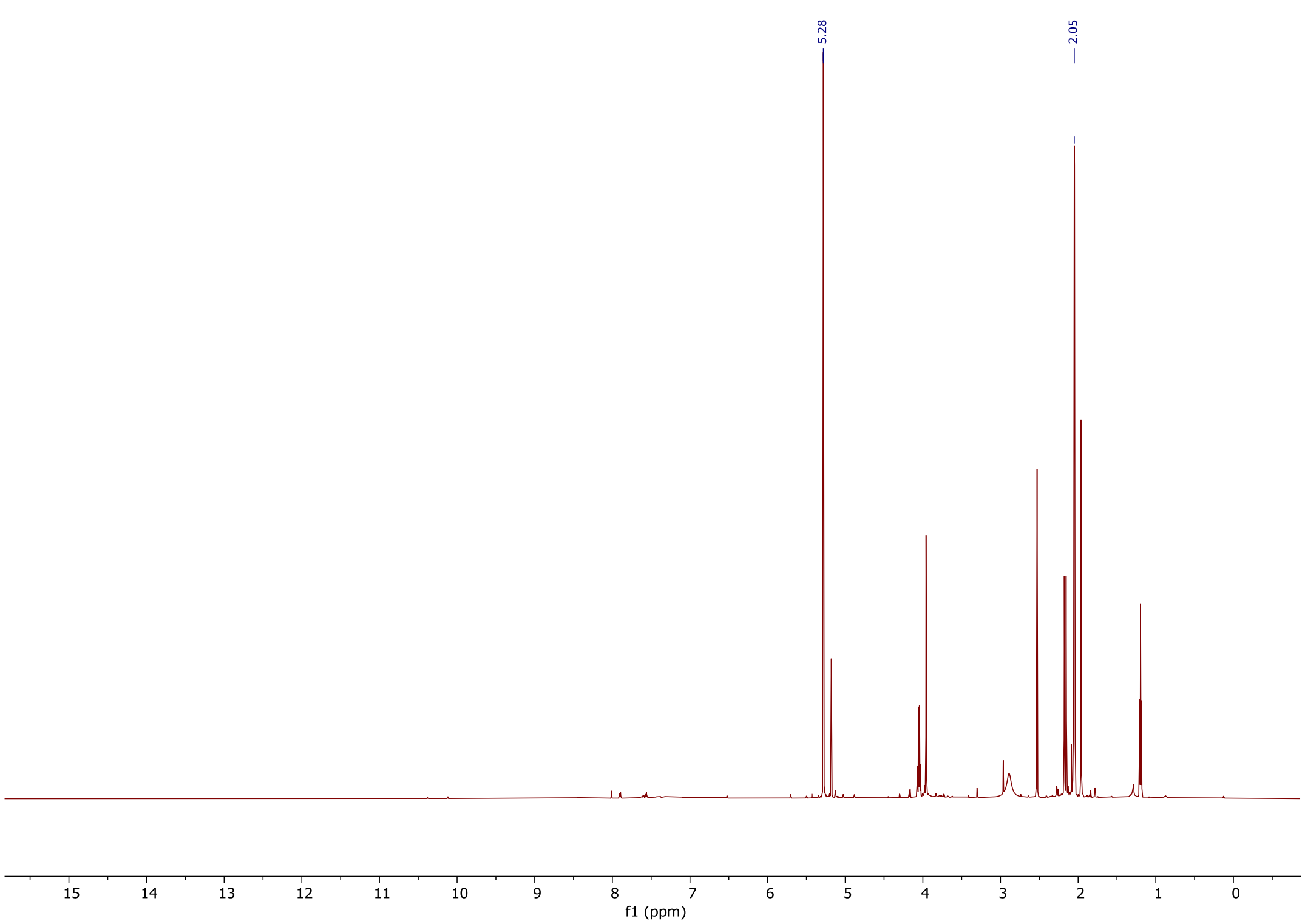

Ag_2O
^{1}H-NMR (599 MHz, d_6-acetone)

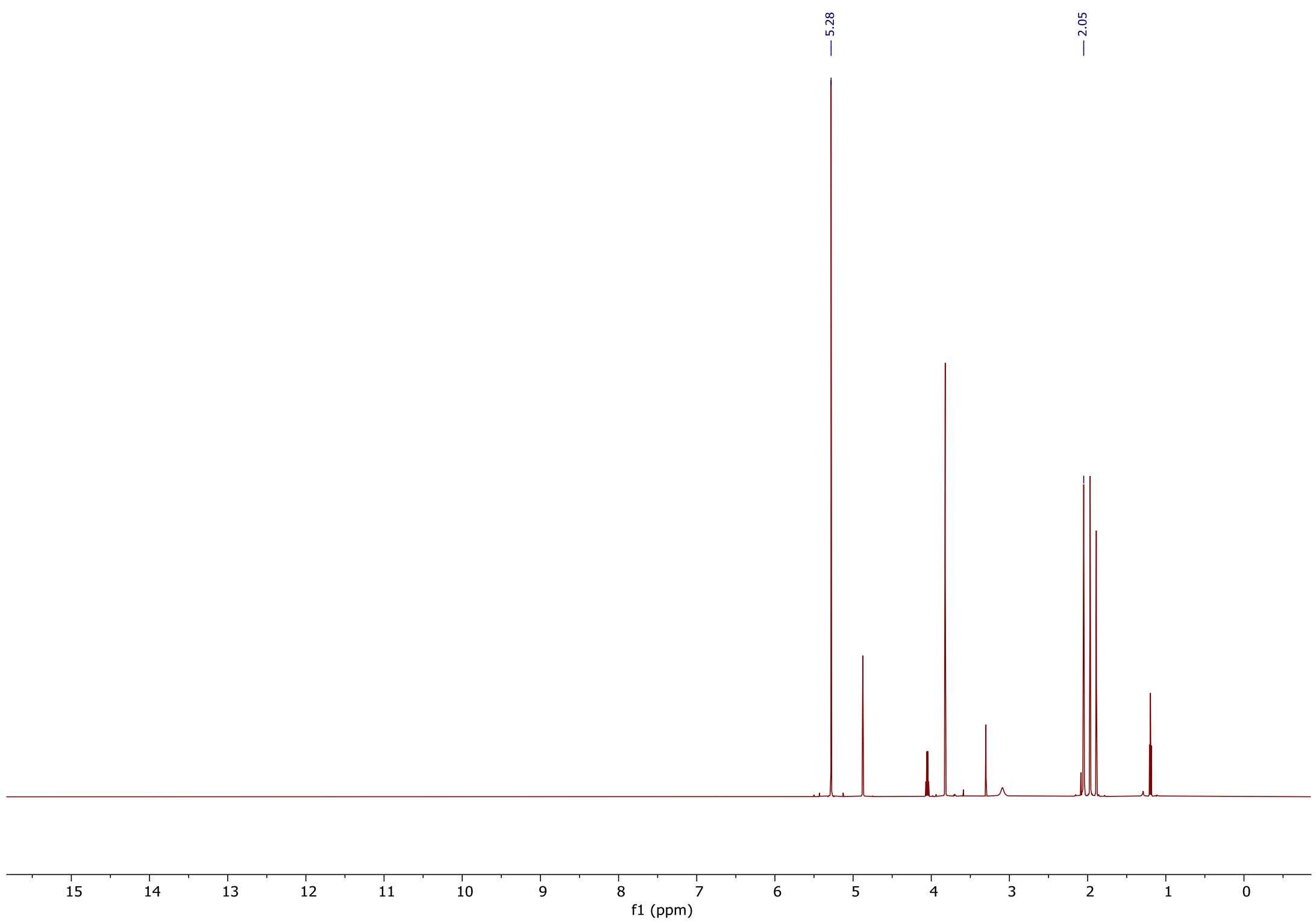

MnO_2
^{1}H-NMR (599 MHz, d_6-acetone)

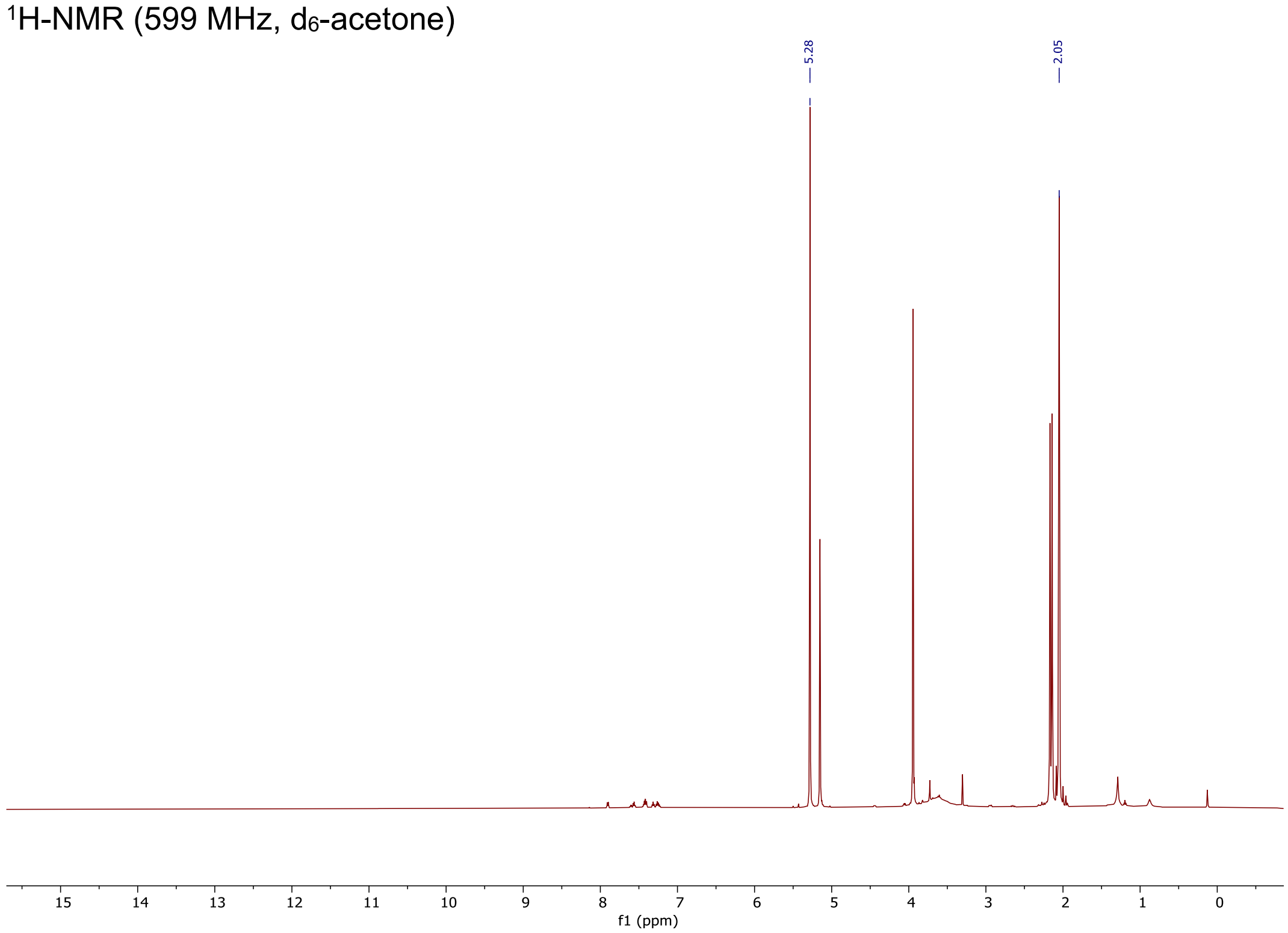

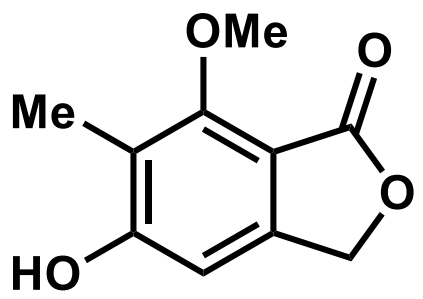

10

None of the chemical oxidations showed a peak with the same retention time as the desired benzylic alcohol **15**.

Condition	Result
Enzyme C4	Benzylic alcohol **15**
AIBN, NBS	At least two new products, none are benzylic alcohol
CAN	decomposition
$Na_2S_2O_8$	Starting material
Ag_2O	New product, not benzylic alcohol
MnO_2	Starting material

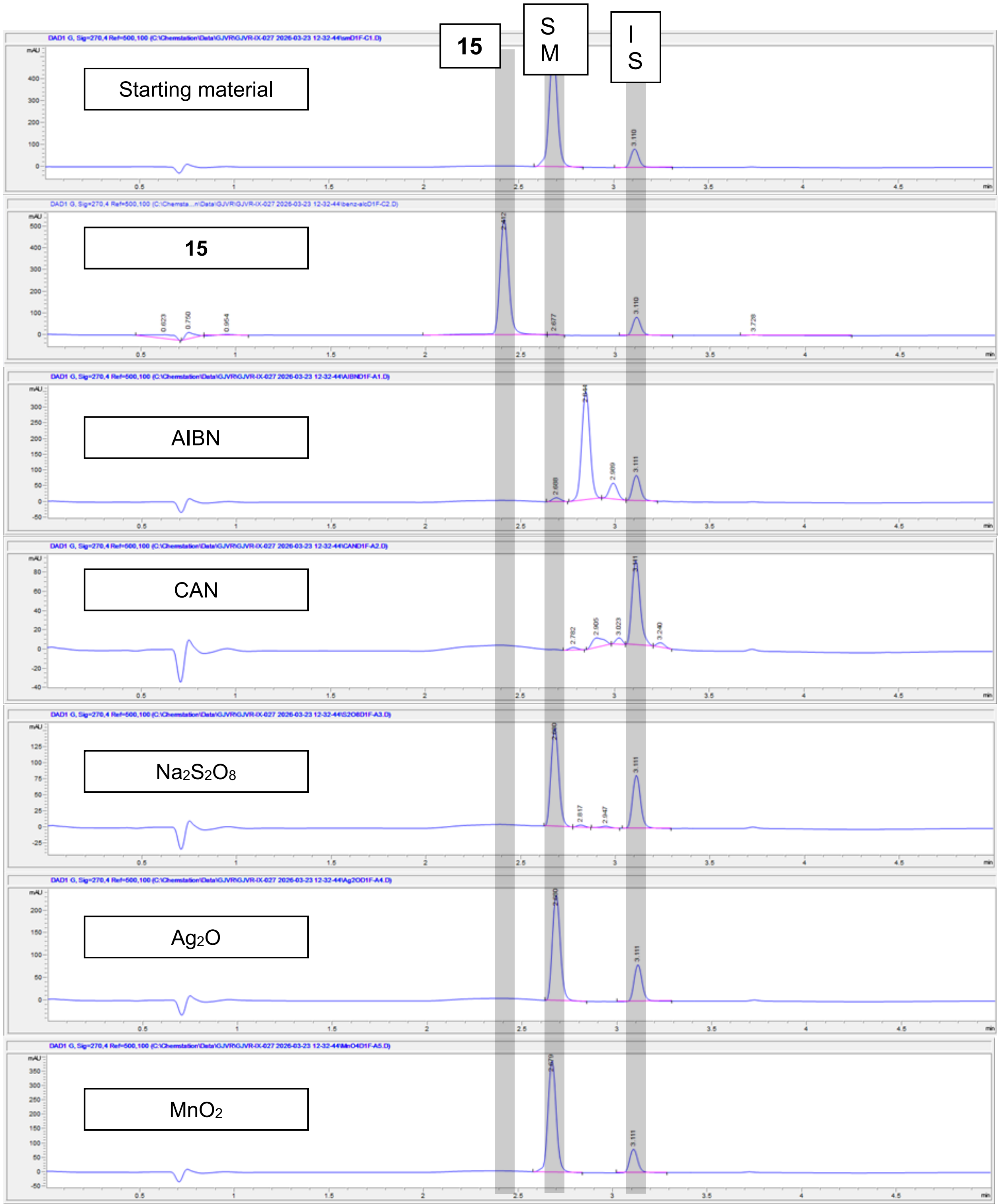
15
S
M
I
S
Starting material
15
AIBN
CAN
Na2S2O8
Ag2O
MnO2

Enzymatic reaction with C4

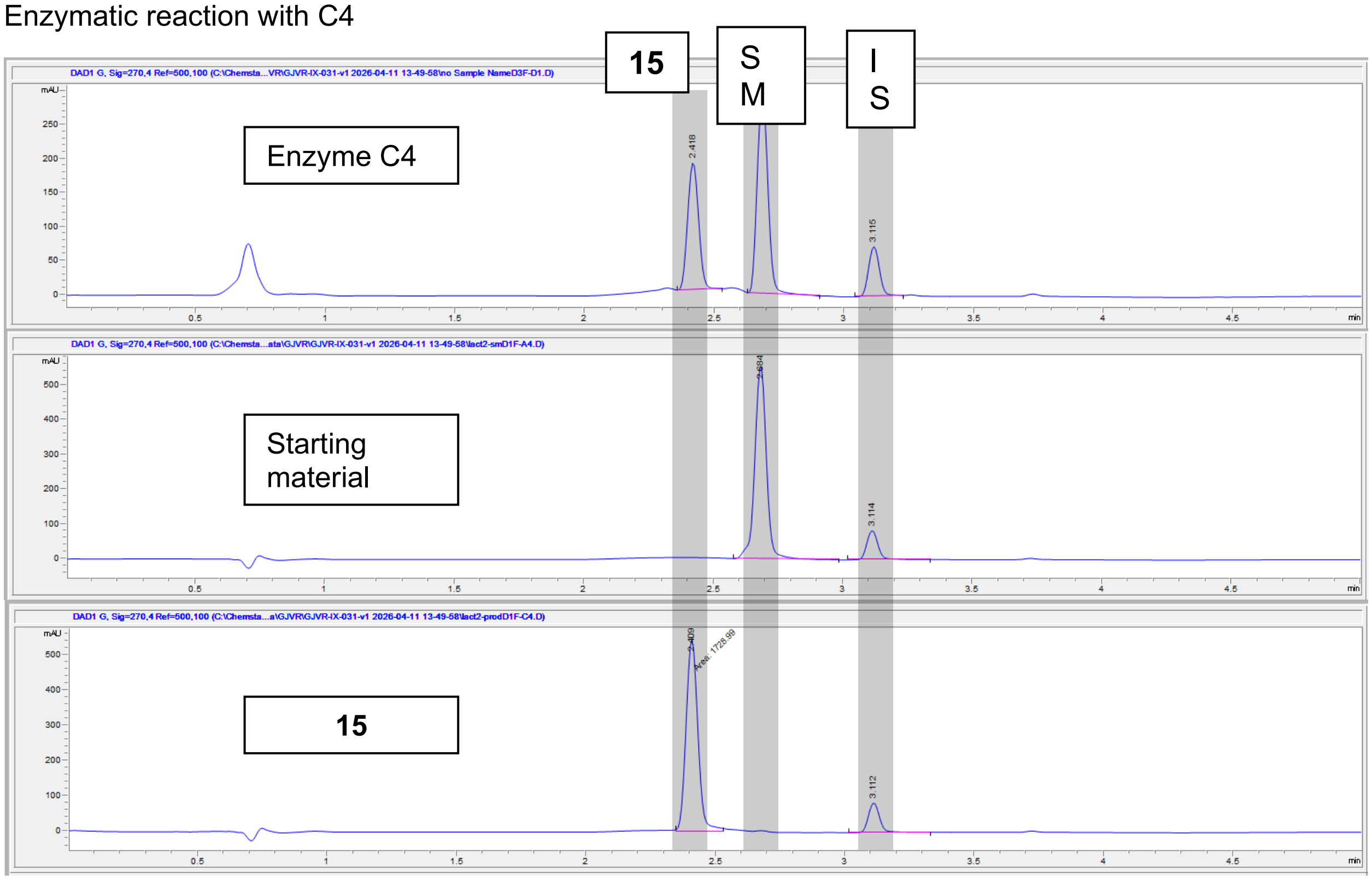

AIBN, NBS

^{1}H-NMR (599 MHz, d_6-acetone)

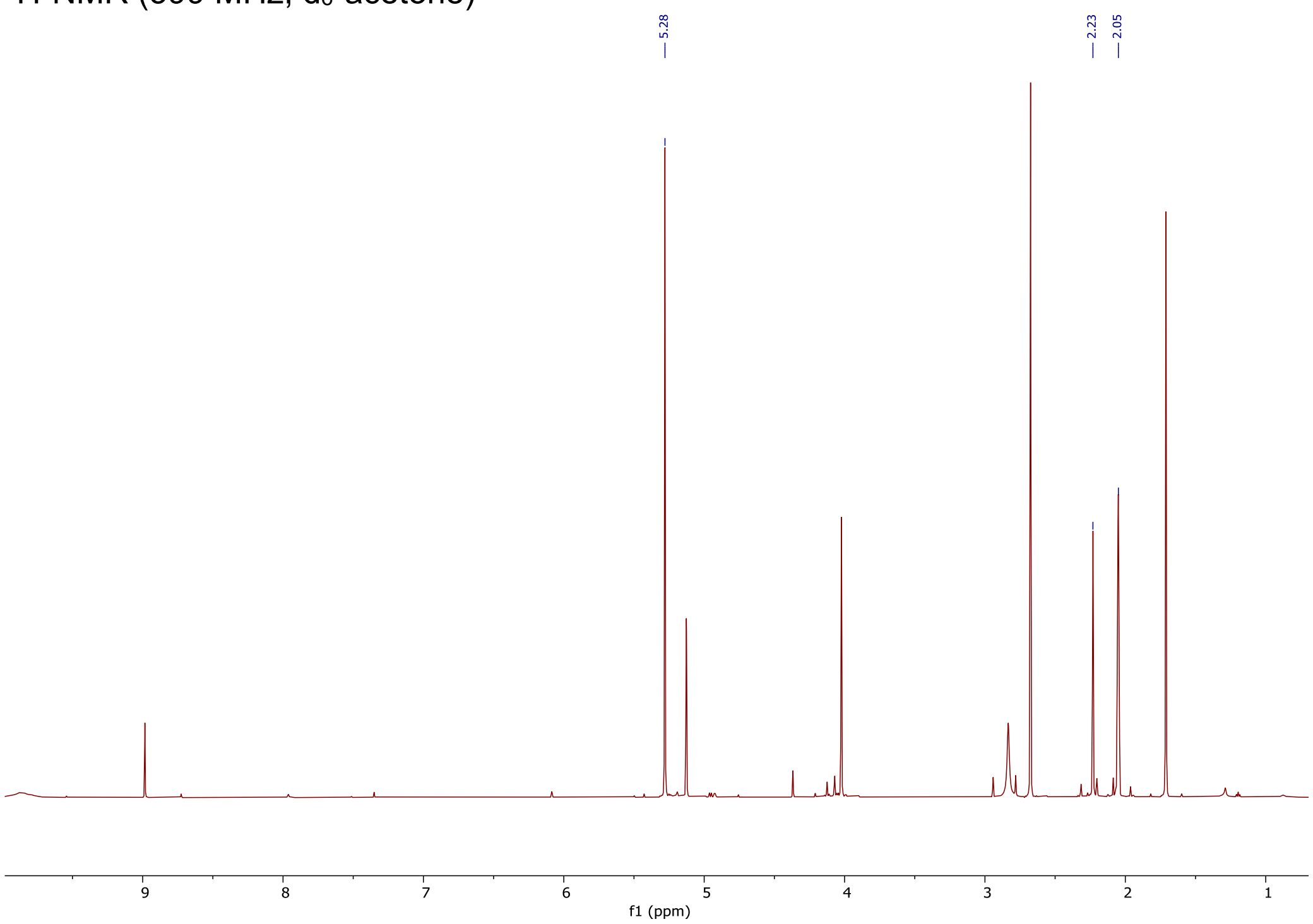

CAN

^{1}H-NMR (599 MHz, d_6-acetone)

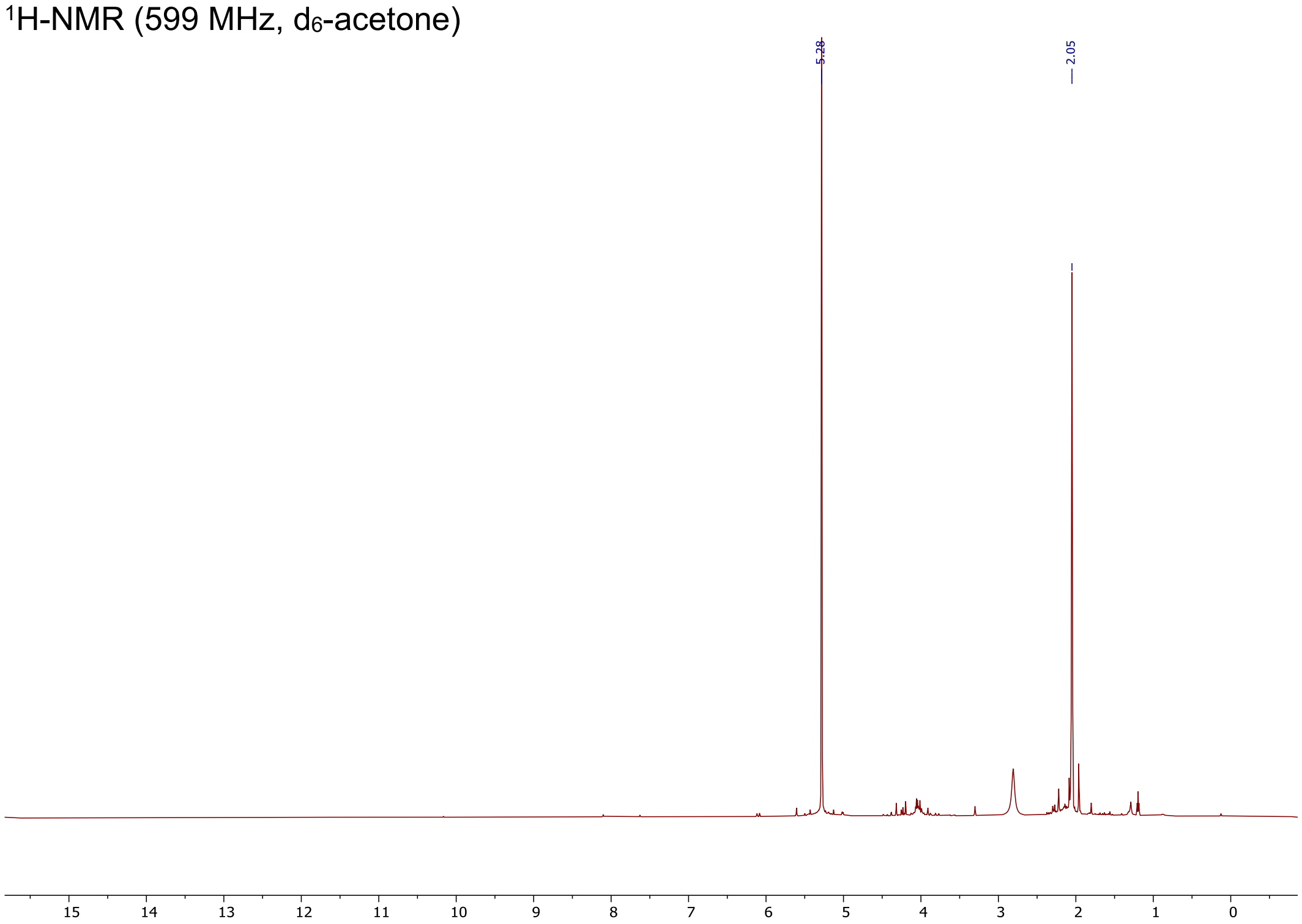

$Na_2S_2O_8$
^{1}H-NMR (599 MHz, d_6-acetone)

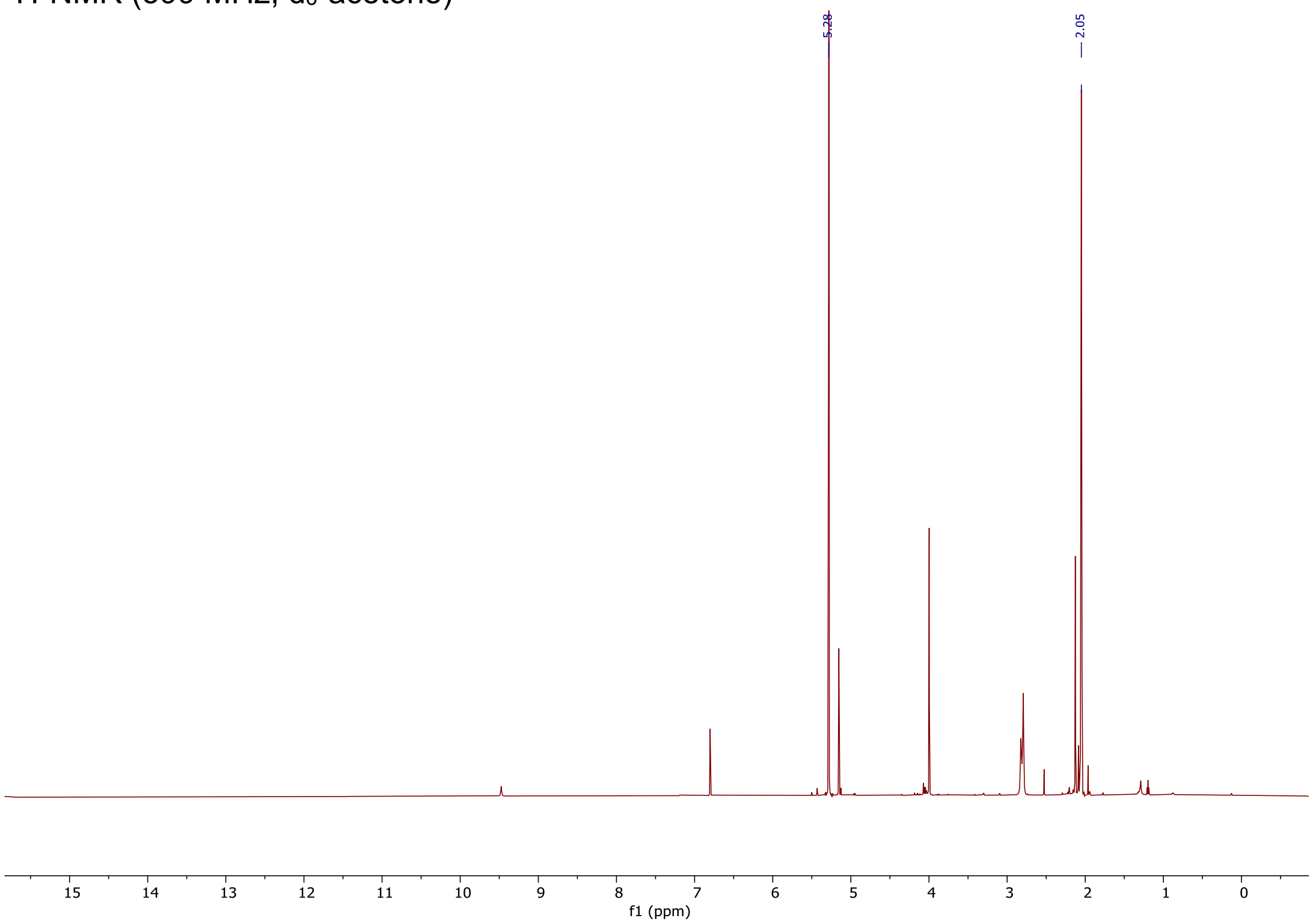

Ag_2O
^{1}H-NMR (599 MHz, d_6-acetone)

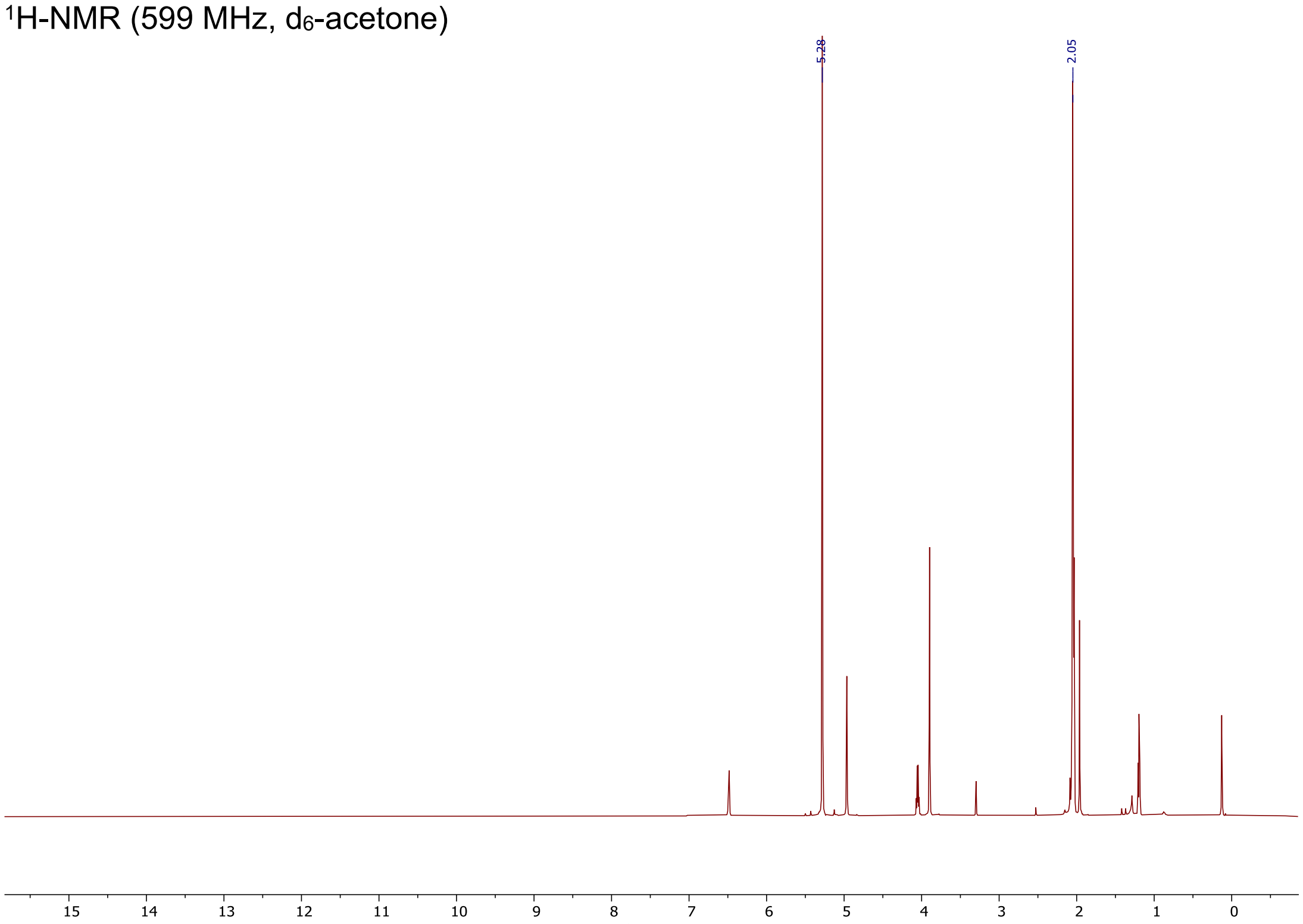

MnO$_2$

^{1}H-NMR (599 MHz, d_6-acetone)

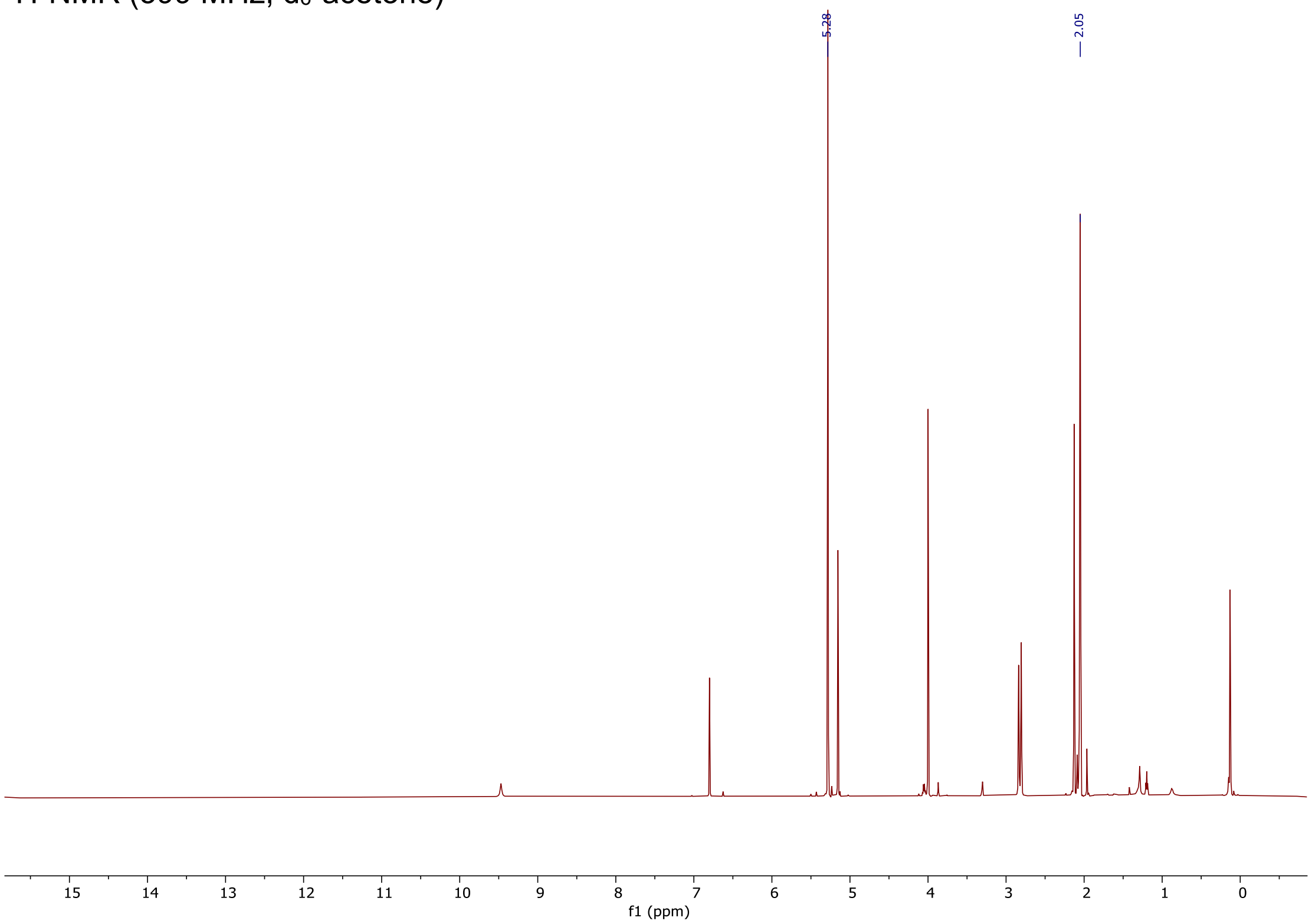

9. Synthetic Procedures and Characterization Data

Whole cell pellet preparation (production of enzyme in bacteria)**:** Overexpression of the gene encoding for a protein of interest was achieved from cultures grown using 4% glycerol (v/v) Terrific Broth (TB) with 50 µg/mL kanamycin in 2.8 L flasks. 1 L portions of autoclaved media were inoculated with 10 mL of overnight cultures that were prepared from the glycerol stocks described in the previous section and 50 µg/mL kanamycin. Cultures were grown at 37 °C and 200 rpm until the optical density at 600 nm (OD_{600}) reached 0.6–0.8. The cultures were then cooled to 20 °C and protein expression was induced with 0.2 mM isopropyl-β-D-1-thiogalactopyranoside (IPTG, Gold Biotechnology). Expression was continued at 20 °C overnight (approx. 18 h) at 200 rpm. The typical yield for 1 L culture cell pellet (wet cell pellet) ranged between 10-20 g. Whole cells were stored at –80 °C until used in biocatalytic reactions according to the procedures described below.

Clarified cell lysate preparation: The procedure must be done on ice and all solutions have to be precooled to ~4 °C. Whole cell pellet containing enzyme was dissolved in lysis buffer (Tris buffer pH 7.5, 300 mM NaCl, 10% glycerol) to make a mixture of 500 mg of pellet per mL of lysis buffer. The mixture was homogenized using a dounce tissue homogenizer. The mixture was transferred to a metal beaker surrounded by ice. Then, phenylmethanesulfonyl fluoride (serine protease inhibitor, 100 mM ethanol solution) was added slowly to final concentration of 1 mM. The homogenized mixture was lysed by sonication, each sonication cycle involved 5 s sonication, followed by a 10 s rest period, for a total of 5 min of sonication at 40% power. The resulting cell lysate was centrifuged at 30,000 x g for 30 min at 4 °C. The clarified supernatant was aliquoted in 10 mL portions, flash frozen in liquid nitrogen, and stored at –80 °C for future use in biocatalytic reactions.

Previously reported compounds: **11**,[12] **S8**.[11]

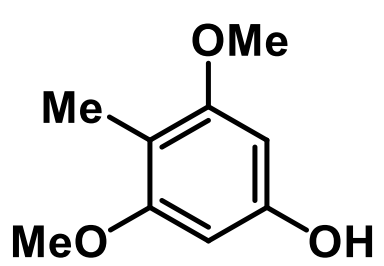

S9

3,5-dimethoxy-4-methylphenol (S9): To a 250 mL flask with stir bar was added 4-hydroxy-2,6-dimethoxybenzaldehyde (2.5 g, 14 mmol, 1.0 equiv), 55 mL of 200 proof ethanol and zinc powder (9.0 g, 10 equiv, 0.14 mol). The reaction was flushed with nitrogen and concentrated HCl (70 mL, 0.82 mol, 60 equiv) was added dropwise while stirring in an ice bath. The reaction mixture turned maroon in color after the addition of the acid. After 1 h, 230 mL of NaOH 3 M was added (final pH 2-3 with pH paper). Then, sat. aq. $NaHCO_3$ was added until the pH was ~6. The reaction mixture was extracted with EtOAc (3 x 200 mL), the combined organic layers were washed with 100 mL of brine, dried with anhydrous Na_2SO_4, concentrated, and purified by column chromatography 5 to 30% EtOAc/Hex. The title compound was obtained as a white solid in 74% yield (1.7 g). Characterization data matches the literature.[13–15]

TLC (30% EtOAc/Hexanes): Rf = 0.4 (UV, CAM stain);
^{1}H NMR (599 MHz, d_4-MeOH): δ 6.05 (s, 2H), 3.74 (s, 6H), 1.91 (s, 3H);
^{13}C NMR (151 MHz, d_4-MeOH): δ 160.2, 157.6, 105.9, 92.6, 55.9, 7.8;
HRMS (ESI-TOF): m/z calculated for $C_9H_{12}O_3$ $[M+H]^+$: 169.0859, found 169.0865.

Note: We found that the compound was more soluble in d_4-methanol than in d-chloroform. The NMR spectra in d-chloroform agrees with previous references.[13–15]

^{1}H NMR (599 MHz, $CDCl_3$) δ 6.07 (s, 2H), 3.78 (s, 6H), 2.00 (s, 3H);
^{13}C NMR (151 MHz, $CDCl_3$) δ 159.0, 154.7, 106.8, 91.9, 55.8, 7.8.

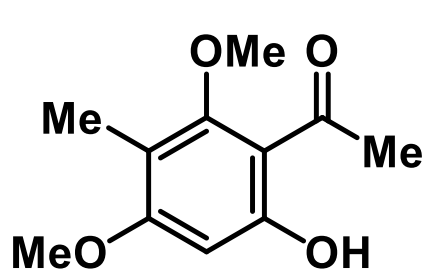

S10

2-hydroxy-4,6-dimethoxy-5-methylacetophenone (S10): Procedure based on reference.[16] To a 50 mL flame-dried flask with **3,5-dimethoxy-4-methylphenol (S9)** (1.5 g, 8.9 mmol, 1.0 equiv) and dry DCM (60 mL) was added 9.8 mL of a 1M

BCl_3 solution in DCM (9.8 mmol, 1.1 equiv) dropwise under an ice bath. Subsequently, AcCl (0.76 mL, 11 mmol) was added dropwise, and the reaction was allowed to stir at room temperature for 18 h.

The reaction was quenched with 45 mL of HCl 1 M under an ice bath and extracted with DCM (3 x 100 mL). The combined organic layers were washed with brine, dried with Na_2SO_4, filtered, and concentrated under reduced pressure. The crude reaction was purified by column chromatography 5% to 30% EtOAc/Hexanes to yield the title compound as a clear viscous oil that solidified to a white solid upon addition of hexanes (72% yield, 1.4 g). Spectroscopic data agrees with previous reference.[16]

TLC (30% EtOAc/Hexanes): R_f = 0.7 (UV, CAM stain);
^{1}H NMR (599 MHz, $CDCl_3$): δ 13.43 (s, 1H), 6.22 (s, 1H), 3.83 (s, 3H), 3.72 (s, 3H), 2.67 (s, 3H), 2.05 (s, 3H);
^{13}C NMR (151 MHz, $CDCl_3$): δ 203.4, 164.8, 164.6, 160.8, 111.5, 109.0, 95.8, 61.6, 55.9, 31.2, 8.7;
HRMS (ESI-TOF): m/z calculated for $C_{11}H_{15}O_4^+$ [M+H]$^+$: 211.0965, found: 211.0961.

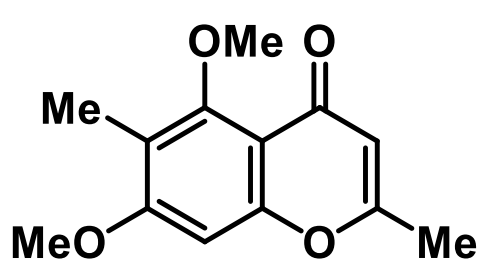

S11

5,7-dimethoxy-2,6-dimethyl-4H-chromen-4-one (S11): To a 100 mL flame-dried round-bottom flask was added 60% dispersion in parafilm of NaH (1.2 g, 30 mmol, 4.0 equiv), dry THF (38 mL) and **2-hydroxy-4,6-dimethoxy-5-methylacetophenone (S10)** (1.6 g, 7.6 mmol, 1.0 equiv) under an ice bath. The solution was allowed to stir for 10 min, then dry EtOAc (2.2 mL, 23 mmol, 3.0 equiv) was added dropwise and the reaction mixture was taken out of the ice bath and allowed to stir at room temperature.

After 19 h, conc. HCl (3.8 mL, 46 mmol, 6 equiv) was added at 0 °C, the reaction mixture was allowed to stir at room temperature for at least 6 h. Subsequently, the reaction mixture was poured into ice water (150 mL), this mixture was extracted with EtOAc (3 x 150 mL), the combined organic layers were dried with Na_2SO_4, filtered, and concentrated under reduced pressure. The crude reaction mixture was purified by column chromatography (20-80% EtOAc/Hexanes) to yield the title compound as a slightly yellow solid (88% yield, 1.6 g).

TLC (80% EtOAC/Hexanes): 0.4 (UV, permanganate);
^{1}H NMR (599 MHz, $CDCl_3$): δ 6.60 (s, 1H), 6.01 (s, 1H), 3.89 (s, 3H), 3.83 (s, 3H), 2.29 (s, 3H), 2.16 (s, 3H);
^{13}C NMR (151 MHz, $CDCl_3$): δ 177.1, 163.3, 162.2, 157.9, 157.8, 118.3, 112.0, 111.9, 94.9, 61.7, 56.0, 20.0, 8.3;
HRMS (ESI-TOF): m/z calculated for C13H15O4$^+$ [M+H]$^+$: 235.0965, found 235.0972;
IR (neat, ATR): 2922, 2855, 1656, 1599, 1334, 1206, 1120, 838, 823 cm^{-1}.

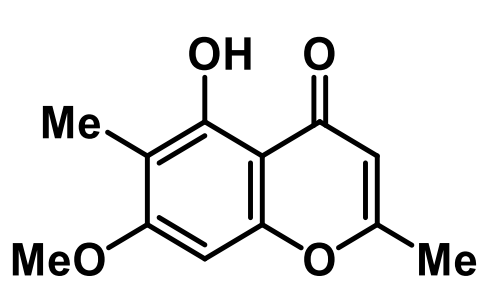

7

5-hydroxy-7-methoxy-2,6-dimethyl-4H-chromen-4-one (7): In a 100 mL round bottom flask was placed 1.2 g (5.1 mmol, 1.0 equiv) of **5,7-dimethoxy-2,6-dimethyl-4H-chromen-4-one (S11)**, 2.86 g (7.68 mmol, 1.50 equiv) of $CeCl_3 \cdot 7H_2O$, 1.15 g (7.68 mmol, 1.50 equiv) of NaI and 24 mL of acetonitrile. The mixture was refluxed for 4 h. The reaction was diluted with 20 mL of water and extracted with dichloromethane (3 x 50 mL). The combined organic layers were washed with brine, dried with Na_2SO_4, filtered, and concentrated under reduced pressure. The crude mixture was purified by column chromatography (elution started at 20% EtOAc/Hex) to yield 0.88 g (78% yield) of the title compound as a white solid.

TLC (50% EtOAc/Hex): R_f = 0.7 (UV, permanganate stain);
^{1}H NMR (599 MHz, $CDCl_3$): δ 12.78 (s, 1H), 6.36 (s, 1H), 6.03 (s, 1H), 3.88 (s, 3H), 2.35 (s, 3H), 2.09 (s, 3H);
^{13}C NMR (151 MHz, $CDCl_3$): δ 182.6, 166.4, 163.4, 158.6, 156.5, 109.0, 109.0, 105.1, 89.3, 56.0, 20.6, 7.4;

HRMS (ESI-TOF): m/z calculated for $C_{12}H_{13}O_4^+$ $[M+H]^+$: 221.0808, found: 221.0815;
IR (neat, ATR): 3082, 2921, 2849, 1654, 1621, 1583, 1501, 1439, 1416, 1399, 1338, 1300, 1210, 1131, 1091 cm^{-1}.

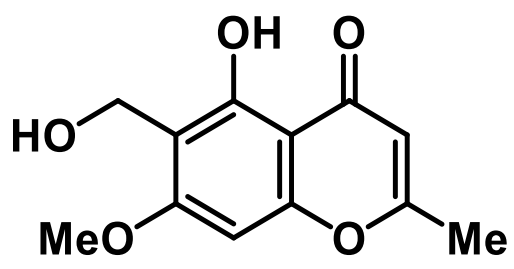

12

6-Hydroxymethyleugenin (12):
Clarified lysate: The procedure must be done in ice and all solutions have to be precooled around 4 °C. Whole cell pellet containing enzyme was dissolved in lysis buffer (Tris buffer pH 7.5, 300 mM NaCl, 10% glycerol) to make a mixture of 500 mg of pellet per mL of lysis buffer. The mixture was homogenized using a dounce tissue homogenizer. The mixture was transferred to a metal beaker surrounded by ice. Then, phenylmethanesulfonyl fluoride (serine protease inhibitor, 100 mM ethanol solution) was added slowly to final concentration of 0.1 mM. The homogenized mixture was lysed by sonication, each sonication cycle involved 5 s sonication, followed by a 10 s rest period, for a total of 5 min at 40% power. The resulting cell lysate was centrifuged at 30,000 x g for 30 min at 4 °C. The clarified supernatant was aliquoted in 10 mL portions, flash frozen in liquid nitrogen, and stored at –80 °C for future use in biocatalytic reactions.

Reaction procedure: A 2 L erlenmeyer flask was charged with MiliQ water (141 mL), 1 M TES buffer pH 7.5 (9.1 mL), sodium ascorbate (144 mg, 1.60 equiv), 2-oxoglutaric acid (106 mg, 1.60 equiv), ferrous sulfate heptahydrate (10 mg, 0.080 equiv), substrate **7** (100 mg pre-dissolved in 9.1 mL of DMSO), clarified lysate containing NHI_A9 (20 mL of a 500 mg/mL stock). The flask was shaken in an incubator at 200 rpm at 30 °C for 2 h, no stir bar was added. Then, sodium ascorbate (144 mg), α-ketoglutaric acid (106 mg), ferrous sulfate heptahydrate (10 mg), and 20 mL of cell lysate were added to the reaction mixture. After another 2 h the reaction was centrifuged for 10 min at 5 000 x g. The supernatant was extracted with EtOAc (3 x 200 mL), isopropanol was added whenever emulsions were formed. The combined organic layers were washed at least three times with 1:1 water:brine, and once with brine. The organic phase was evaporated under reduced pressure at a maximum temperature of 25 °C. The crude product was purified by column chromatography (50-80% EtOAc/Hex) to yield a cream-colored solid (63 mg, 59% yield).

Characterization data agreed with reported data.[17]

TLC (80% EtOAc/Hex): R_f = 0.4 (UV, permanganate stain);
^{1}H NMR (599 MHz, $CDCl_3$): δ 13.03 (s, 1H), 6.38 (s, 1H), 6.06 (d, J = 0.8 Hz, 1H), 4.79 (s, 2H), 3.91 (s, 3H), 2.36 (s, 2H);
^{13}C NMR (151 MHz, $CDCl_3$): δ 182.7, 166.9, 163.3, 159.7, 157.9, 112.0, 109.2, 105.3, 89.8, 56.2, 53.9, 20.6;
HRMS (ESI-TOF): m/z calculated for $C_{12}H_{11}O_4^+$ $[M - H_2O + H]^+$: 219.0652, found: 219.0658.

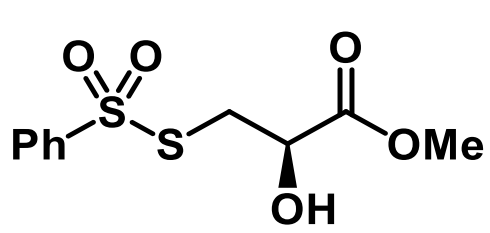

S12

methyl (*R*)-2-hydroxy-3-((phenylsulfonyl)thio)propanoate (S12): Procedure based on previous ref.[18]

L-Cysteine hydrochloride monohydrate (10.5 g, 60.0 mmol) was dissolved in 60 mL of 2 M HCl in a 250 mL round bottom flask, and then cooled in an ice bath. The reaction was run under ambient atmosphere. While stirring, sodium nitrite (4.14 g, 60 mmol) dissolved in 40 mL of water was added dropwise. The reaction mixture turned red; this mixture was allowed to stir for 40 min. Then a solution of sodium benzene sulfinate (19.7 g, 120 mmol) in 40 mL water was added dropwise. The reaction mixture was warmed to room temperature and allowed to stir for 4 h. During this time, the red color disappeared, and white solids precipitated. The suspension was briefly cooled in an ice bath, then filtered and washed with approximately 120 mL each of water, acetone, and hexanes. The obtained cream-colored solid (6.0 g of protected cysteine, 23 mmol, 38% yield) was used in the next step without further purification.

The protected cysteine (3.00 g, 11.5 mmol) was dissolved in 46 mL of 1 M sulfuric acid in a 250 mL round-bottom flask equipped with a magnetic stirrer. The reaction was run open to ambient atmosphere. The mixture was cooled in an ice bath; 46 mL of acetone was then added. Sodium nitrite (3.17 g, 45.9 mmol, 4.00 equiv) was dissolved in water (34 mL) and then

added dropwise. The solution allowed to stir for 2 h, then warmed to room temperature and allowed to stir for another 2 h. The reaction mixture was transferred to a separatory funnel. Saturated sodium sulfate was added (90 mL), and then the aqueous layer was extracted with ethyl acetate (4 × 50 mL). The combined organic extracts were dried (Na_2SO_4), filtered and concentrated under reduced pressure to yield a yellow oil (3.0 g, quantitative). A portion of the crude oil (1.0 g) was dissolved in dry methanol in a flame-dried round-bottom flask and stirred for 3 days at room temperature. The solvent was removed under reduced pressure, and the resulting residue was purified by column chromatography (20−40% EtOAc/hexanes) to yield a yellow oil (0.88 g, 83% yield).

Spectroscopic data agreed with previously reported data. ref.[18]

TLC (50% EtOAc/Hex): R_f = 0.4 (UV, permanganate stain);
^{1}H NMR (599 MHz, $CDCl_3$): δ 7.92–7.96 (m, 2H), 7.68–7.62 (m, 1H), 7.60 – 7.54 (m, 2H), 4.44 (m, 1H), 3.77 (s, 3H), 3.51 (dd, *J* = 14.0, 4.1 Hz, 1H), 3.32 (dd, *J* = 14.0, 6.2 Hz, 1H), 3.12 (d, *J* = 5.2 Hz, 1H, OH);
^{13}C NMR (151 MHz, $CDCl_3$): δ 172.5, 144.6, 134.1, 129.5, 127.2, 69.3, 53.3, 39.8;
$[\alpha]_D^{23}$: +7.0 (c = 2.3 in $CHCl_3$). Reported +7.8 (c = 0.57 in $CHCl_3$).[17]

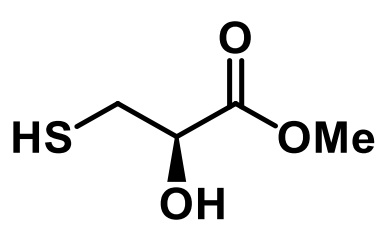

S13

methyl (*R*)-2-hydroxy-3-mercaptopropanoate (S13): In a 250 mL round-bottom flask was placed **methyl (*R*)-2-hydroxy-3-((phenylsulfonyl)thio)propanoate (S12)** (1.5 g, 5.4 mmol, 1.0 equiv), 55 mL of dichloromethane and 0.2 mL of water. The reaction mixture was placed in an ice bath, then tris(2-carboxyethyl)phosphonium chloride (3.4 g, 12 mmol, 2.2 equiv) and triethylamine (1.7 mL, 12 mmol, 2.2 equiv) were added. After stirring for 15 min, to the solution was added 15 mL of dichloromethane and 50 mL of 1 M HCl. The resulting mixture was extracted with dichloromethane, the combined organic layers were washed with brine, dried with Na_2SO_4, filtered, concentrated, and purified by column chromatography (20–50% EtOAc/Hex). A slightly yellow oil was obtained (0.35 g, 47% yield).

Note: The H-NMR shows a triplet that couples to two diastereotopic C–H's in the molecule (based on COSY-NMR). This suggests that the molecule is a free thiol. We were not able to find the mass of the proposed product in our HRMS measurements. This is because MS analysis of thiols generally requires derivatization,[18,19] we confirmed the proposed thiol structure by HRMS of (*ent*)-chaetosemin B.

TLC (30% EtOAc/Hex): R_f = 0.4 (permanganate stain);
^{1}H NMR (599 MHz, $CDCl_3$): δ 4.44 (t, *J* = 4.2 Hz, 1H), 3.83 (s, *J* = 0.8 Hz, 3H), 2.96 (ddd, *J* = 14.0, 7.9, 3.9 Hz, 1H), 2.85 (ddd, *J* = 14.1, 9.6, 4.6, 1H), 1.64 (dd, *J* = 9.6, 7.9 Hz, 1H);
^{13}C NMR (151 MHz, $CDCl_3$): δ 173.3, 70.9, 53.1, 28.9;
IR (neat, ATR): 3464, 2954, 1737, 1439, 1217, 1101, 1027 cm^{-1};
HRMS (ESI-TOF): We were not able to find the mass for the proposed structure. We were able to find the mass of the disulfide: m/z calculated for $C_8H_{15}O_6S_2^+$ $[M+H]^+$: 271.0305, found: 271.0311.
$[\alpha]_D^{23}$: – 9.2 (c = 1.6, in $CHCl_3$).

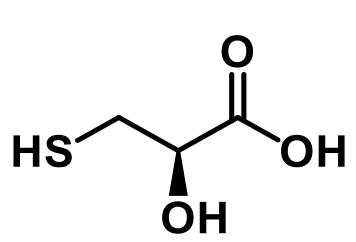

S14

(*R*)-2-hydroxy-3-mercaptopropanoic acid (S14): In a 25 mL round-bottom flask was placed 5 mL of methanol, **methyl (*R*)-2-hydroxy-3-mercaptopropanoate (S13)** (100 mg, 0.73 mmol, 1 equiv) and $Ba(OH)_2 \cdot 8(H_2O)$ (232 mg, 0.730 mmol, 1.00 equiv). After stirring at room temperature for 16 h, the reaction was partitioned between DI water and EtOAc. After extracting with EtOAc, the combined organic layers were dried with Na_2SO_4, filtered, and concentrated to yield a white solid (70 mg, 78% yield). The obtained crude was used without further purification.

^{1}H NMR (500 MHz, d_6-DMSO): δ 4.09 (dd, *J* = 6.1, 4.6 Hz, 1H), 2.81 – 2.72 (m, 1H), 2.72 – 2.63 (m, 1H), 2.23 (t, *J* = 8.4 Hz, 1H);
^{13}C NMR (126 MHz, d_6-DMSO): δ 173.7, 71.1, 28.3;
$[\alpha]_D^{23}$: –9.7 (c = 1.3 in MeOH).

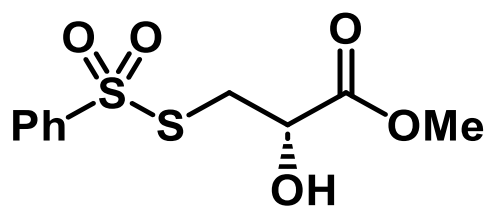

S15

methyl (*S*)-2-hydroxy-3-((phenylsulfonyl)thio)propanoate (S15): The same procedure was used as for **methyl (*R*)-2-hydroxy-3-((phenylsulfonyl)thio)propanoate (S12),** but using D-cysteine hydrochloride monohydrate as starting material. For the first and second steps, starting with 5.0 g which led to 5.5 g of crude product.
For the third step, 4.6 g of starting material led to 2.3 g of product (48 % yield).

TLC (50% ethyl acetate in hexanes): R_f = 0.4 (UV, permanganate stain);
^{1}H NMR (599 MHz, $CDCl_3$): δ 7.97–7.92 (m, 1H), 7.68–7.63 (m, 1H), 7.57 (dd, *J* = 8.5, 7.2 Hz, 1H), 4.47 – 4.41 (m, 1H), 3.78 (s, 1H), 3.52 (dd, *J* = 14.1, 4.1 Hz, 1H), 3.32 (dd, *J* = 14.0, 6.2 Hz, 1H), 3.06 (d, *J* = 5.4 Hz, 1H, -OH);
^{13}C NMR (151 MHz, $CDCl_3$): δ 172.5, 144.7, 134.1, 129.5, 127.2, 69.3, 53.4, 39.8;
$[\alpha]_D^{23}$: –3.2 (c = 3.1 in $CHCl_3$).

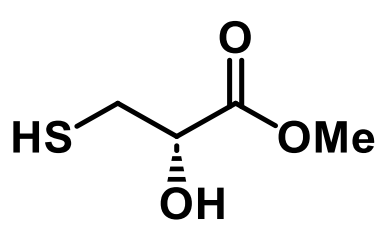

18

methyl (*S*)-2-hydroxy-3-mercaptopropanoate (18): In a 250 mL round-bottom flask was placed **methyl (*S*)-2-hydroxy-3-((phenylsulfonyl)thio)propanoate (S15)** (1.5 g, 5.4 mmol, 1.0 equiv), 55 mL of dichloromethane and 0.2 mL of water. The reaction mixture was placed in an ice bath, then tris(2-carboxyethyl)phosphonium chloride (3.4 g, 12 mmol, 2.2 equiv) and triethylamine (1.7 mL, 12 mmol, 2.2 equiv) were added. After stirring for 15 min, to the solution was added 15 mL of dichloromethane and 50 mL of 1M HCl. The resulting mixture was extracted with dichloromethane, the combined organic layers were washed with brine, dried with Na_2SO_4, filtered, concentrated, and purified by column chromatography (20-50% EtOAc/Hex). A slightly yellow oil was obtained (0.33 g, 44% yield).

Note: We were not able to find the mass of the proposed product in our HRMS measurements. This is because MS analysis of thiols generally requires derivatization,[19,20] we confirmed the proposed thiol structure by HRMS and X-ray of chaetosemin B.

TLC (30% EtOAc/Hex): R_f = 0.4 (permanganate stain);
^{1}H NMR (599 MHz, $CDCl_3$): δ 4.44 (t, *J* = 4.2 Hz, 1H), 3.83 (s, 3H), 2.96 (ddd, *J* = 14.0, 7.9, 3.8 Hz, 1H), 2.86 (ddd, *J* = 14.0, 9.6, 4.5 Hz, 1H), 1.62 (dd, *J* = 9.6, 7.8 Hz, 1H);
^{13}C NMR (151 MHz, $CDCl_3$): δ 173.3, 70.9, 53.1, 28.9;
IR (thin film): 3467, 2955, 1738, 1439, 1217, 1101, 1022 cm^{-1};
HRMS (ESI-TOF): We were not able to find the mass for the proposed structure. We were able to find the mass of the disulfide: m/z calculated for $C_8H_{15}O_6S_2^+$ [M+H]+: 271.0305, found: 271.0313;
$[\alpha]_D^{23}$: + 8.1 (c = 1.6 in $CHCl_3$).

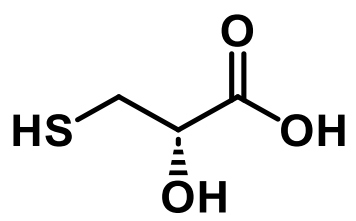

19

(*S*)-2-hydroxy-3-mercaptopropanoic acid (19): In a 25 mL round-bottom flask was placed 5 mL of methanol, **methyl (S)-2-hydroxy-3-mercaptopropanoate (18)** (100 mg, 0.73 mmol, 1 equiv) and $Ba(OH)_2 \cdot 8(H_2O)$ (232 mg, 0.730 mmol, 1.00 equiv). After stirring at room temperature for 16 h, the reaction was partitioned between DI water and EtOAc. After extracting with EtOAc, the combined organic layers were dried with Na_2SO_4, filtered, and concentrated to yield a white solid (60 mg, 66% yield). The obtained crude was used without further purification.

Spectroscopic characterization data agrees with a previous report.[21]

^{1}H NMR (599 MHz, d_6-DMSO): δ 4.12–4.07 (m, 1H), 2.81–2.75 (m, 1H), 2.72–2.63 (t, *J* = 6.6 Hz, 1H), 2.23 (t, *J* = 7.6 Hz, 1H);
^{13}C NMR (151 MHz, d_6-DMSO): δ 173.7, 71.1, 28.2;
$[\alpha]_D^{23}$: + 14.2 (c = 0.48 in MeOH), reported **$[\alpha]_D^{25}$:** + 20.3 (c= 0.93, MeOH).[21]

(*ent*)-chaetosemin B (**S16**)

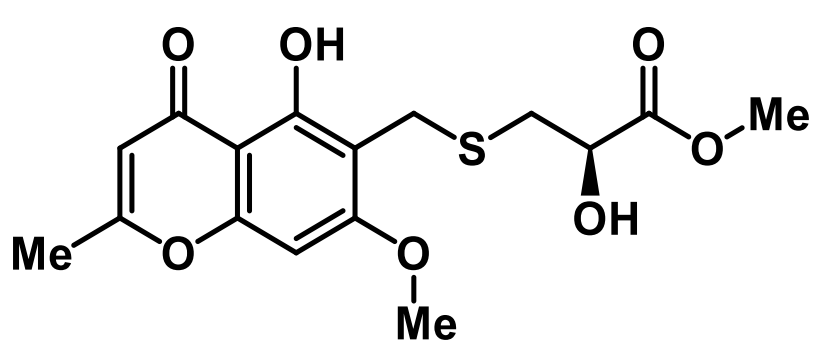

S16

(*ent*)-chaetosemin B (S16): In a 5 mL vial was dissolved benzylic alcohol **12** (34 mg, 0.14 mmol, 1.0 equiv), with 3 mL of acetonitrile. Then, thiol **S13** (50 mg, 0.37 mmol, 2.6 equiv) was added. The reaction vial was flushed with nitrogen, sealed and placed in a 60 °C oil bath for 3 d. The reaction mixture was concentrated under reduced pressure and the obtained crude was purified by column chromatography. 20% EtOAc/Hex was used to fully elute the thiol, then the polarity was gradually increased to 45% EtOAc/Hex until the title compound eluted. The title compound was obtained as a white solid (36 mg, 80% yield).

Note: $CDCl_3$ was eluted through basic alumina before preparing the NMR sample.

Spectroscopic data agrees with chaetosemin B,[22] except for the optical rotation.

TLC (80% ethyl acetate in hexanes): R_f = 0.55 (permanganate, UV). Spot appears between both starting materials (permanganate);
^{1}H NMR (599 MHz, $CDCl_3$): δ 13.07 (s, 1H), 6.38 (s, 1H), 6.05 (s, 1H), 4.45 (dd, *J* = 7.0, 3.9 Hz, 1H), 3.91 (s, 3H), 3.85 (d, *J* = 12.8 Hz, 1H), 3.90 (d, 1H, *J* = ? a peak is buried in the 3.91 ppm signal), 3.77 (s, 3H), 3.29 (s broad, 1H, -OH), 3.06 (dd, *J* = 14.2, 3.9 Hz, 1H), 2.86 (dd, *J* = 14.2, 7.0 Hz, 1H), 2.35 (s, 3H);
^{13}C NMR (151 MHz, $CDCl_3$): δ 182.5, 173.6, 166.9, 163.0, 159.0, 157.5, 110.1, 109.1, 105.2, 90.0, 70.5, 56.3, 52.7, 36.8, 23.8, 20.6;
HRMS (ESI-TOF): m/z calculated for $C_{16}H_{18}O_7S$ $[M+H]^+$: 355.0846, found: 355.0844. $[M+Na]^+$: 377.0665, found: 377.0664;
IR (neat, ATR): 3383, 3225 (broad), 2916, 2849, 1740, 1658, 1621, 1582, 1491, 1447, 1432, 1405, 1346, 1196, 1179, 1093, 808 cm^{-1};
$[\alpha]_D^{23}$: +7.7 (c = 0.016 g/mL in MeOH).

Total synthesis of chaetosemin B (**2**, revised)

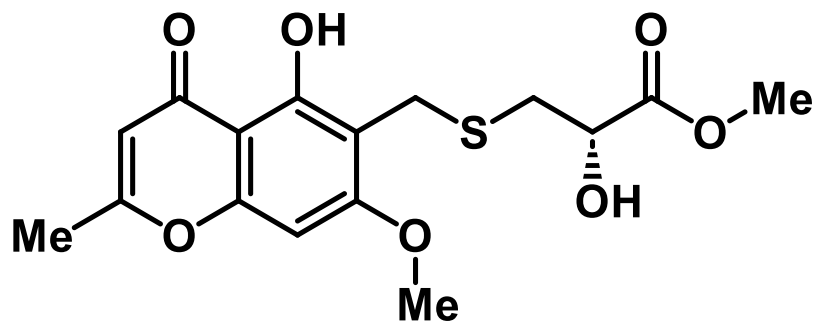

2

chaetosemin B (2, revised): In a 20 mL vial was mixed benzylic alcohol **12** (55 mg, 0.23 mmol, 1.0 equiv), 5.8 mL of acetonitrile and thiol **18** (90 mg, 0.66 mmol, 2.8 equiv). The reaction was flushed with nitrogen, sealed, placed in a 60 °C oil bath and allowed to stir for 2 d. Then, 15 mg (0.5 equiv) of thiol was added and after 1 d of stirring an additional 45 mg (1.4 equiv) of thiol was added and the reaction mixture was stirred for another day. The reaction mixture was concentrated under reduced pressure and the obtained crude was purified by column chromatography. 20% EtOAc/Hex was used to fully elute the thiol, then the polarity was gradually increased to 45% EtOAc/Hex until the title compound eluted. The title compound was obtained as a white solid (42.9 mg, 52% yield).

Note: $CDCl_3$ was eluted through basic alumina before preparing the NMR sample.

TLC (80% ethyl acetate in hexanes): R_f = 0.57 (permanganate, UV). Spot appears between both starting materials (permanganate);
^{1}H-NMR (599 MHz, $CDCl_3$): δ 13.08 (s, 1H), 6.39 (s, 1H), 6.06 (s, 1H), 4.45 (td, *J* = 6.6, 3.9 Hz, 1H), 3.92 (s, 3H), 3.88 (d, *J* = 16.1 Hz, 2H), 3.78 (s, 3H), 3.27 (d, *J* = 6.2 Hz, 1H, -OH), 3.07 (dd, *J* = 14.2, 3.9 Hz, 1H), 2.87 (dd, *J* = 14.2, 7.0 Hz, 1H), 2.36 (s, 3H);
^{13}C-NMR (151 MHz, $CDCl_3$): δ 182.5, 173.6, 166.9, 163.1, 159.0, 157.5, 110.1, 109.2, 105.3, 90.0, 70.5, 56.3, 52.7, 36.9, 23.8, 20.6;
HRMS (ESI-TOF): m/z calculated for $C_{16}H_{18}O_7S$ $[M+H]^+$: 355.0846, found: 355.0850. $[M+Na]^+$: 377.0665, found: 377.0668;
IR (neat, ATR): 3437, 3100-3300 (broad), 3009, 2978, 2958, 2849, 1743, 1662, 1618, 1588, 1496, 1445, 1404, 1390, 1343, 1307, 1203, 1104, 1084 cm^{-1};
UV (MeOH, 0.028 mg/mL): λ_{max} (log ε) 258 (4.01), 290 (3.59) nm;
$[\alpha]_D^{23}$: -6.6 (c = 0.5 , MeOH). Reported **$[\alpha]_D^{28}$**= -6.3 (c = 0.2, MeOH).[22]

^{1}H-NMR

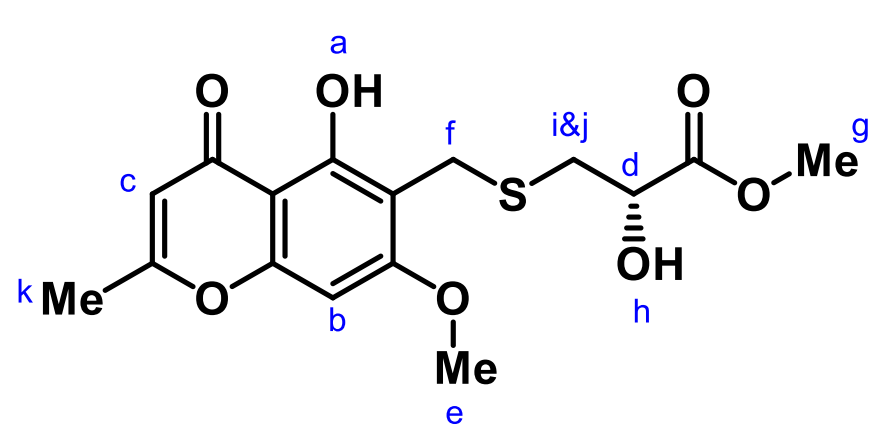

#H	Synthetic (ppm)	Isolated (ref [22] ppm)	Difference (ppm)
a	13.08 (s, 1H)	13.06 (s, 1H)	-0.02
b	6.39 (s, 1H)	6.36 (s, 1H)	-0.03
c	6.06 (s, 1H),	6.03 (s, 1H)	-0.03
d	4.45 (td, J = 6.6, 3.9 Hz, 1H)	4.44 (dd, J = 7.4, 3.4 Hz, 1H)	-0.01
e	3.92 (s, 3H)	3.90 (s, 3H)	-0.02
f	3.88 (d, J = 16.1 Hz, 2H)	3.85 (d, J=12.9 Hz, 2H)	-0.03
g	3.78 (s, 3H)	3.76 (s, 3H)	-0.02
h	3.27 (d, J = 6.2 Hz, 1H, -OH)	-	-
i	3.07 (dd, J = 14.2, 3.9 Hz, 1H)	3.04 (dd, J = 13.6, 3.4 Hz, 1H)	-0.03
j	2.87 (dd, J = 14.2, 7.0 Hz, 1H)	2.85 (dd, J = 13.6, 7.4 Hz, 1H)	-0.02
k	2.36 (s, 3H)	2.34 (s, 3H)	-0.02

Note: From 3.83 ppm to 3.90 ppm we observe three peaks that are the signals of diastereotopic protons "f", we should observe four peaks but one of them is buried within the singlet at 3.92. Instead of 3.88 (d, *J* = 16.1 Hz, 2H) it should be 3.85 (d, *J* = 12.7, 1H) and 3.90 (d, 1H, cannot calculate *J* of buried peak). We cannot discern if there are two or three peaks between 3.83 ppm to 3.90 ppm in the reported NMR from the natural product isolation.

^{13}C-NMR

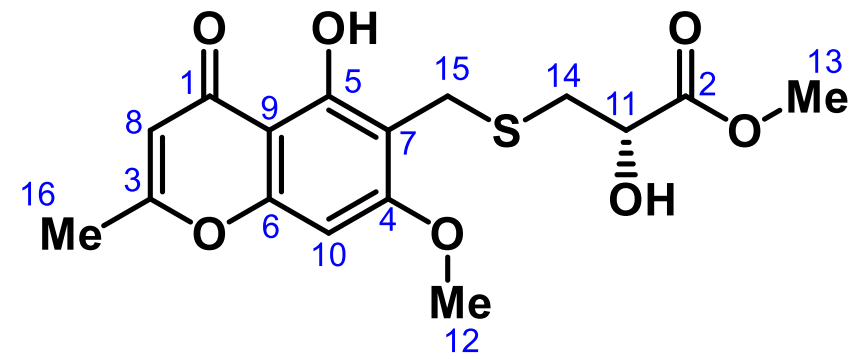

#C	Synthetic	Isolated (ref [22])	Difference
1	182.5	182.3	-0.2
2	173.6	173.4	-0.2
3	166.9	166.7	-0.2
4	163.1	162.8	-0.3
5	159.0	158.7	-0.3
6	157.5	157.2	-0.3
7	110.1	109.8	-0.3
8	109.2	108.9	-0.3
9	105.3	104.9	-0.4
10	90.0	89.8	-0.2
11	70.5	70.2	-0.3
12	56.3	56.1	-0.2
13	52.7	52.4	-0.3
14	36.9	36.6	-0.3
15	23.8	23.5	-0.3
16	20.6	20.4	-0.2

Chiral separations (UPLC-DAD) of chaetosemin B and (*ent*)-chaetosemin B
Column: Phenomenex Lux 3 µm cellulose-2, 100 x 4.6 mm
Method:

Time (min)	Solvent A %	Solvent B %
0	60	40
15	60	40
16	0	100
17	0	100
18	60	40
19	60	40

Solvent A: 20 mM aqueous ammonium bicarbonate with 0.1% diethylamine, solvent B: acetonitrile with 0.1% diethyl amine.
Flow: 1 mL/min

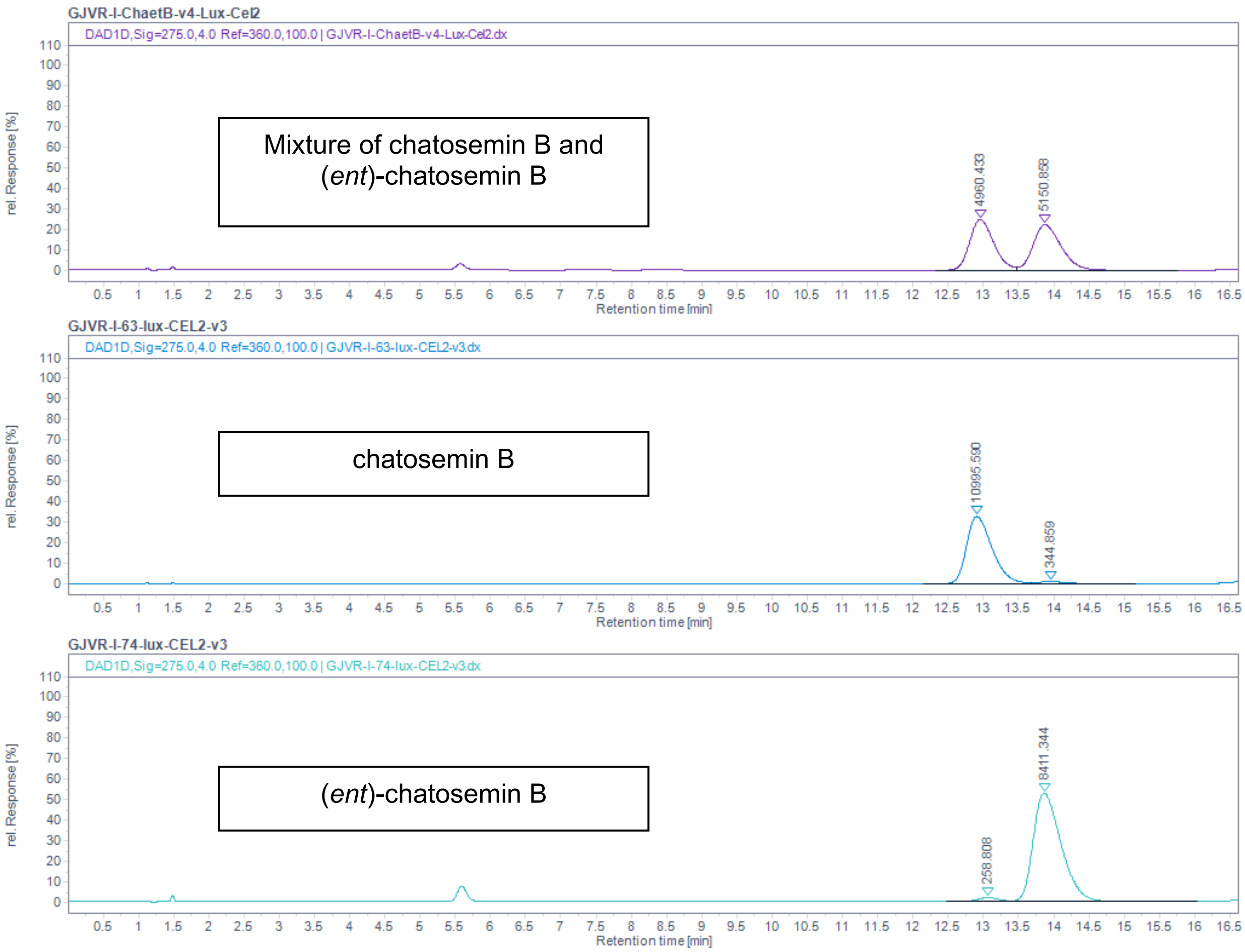

Chromatogram	Sample	RT (min)	Area (AU)	er (%)
1	Racemic mixture	13.0	4960.433	49.1 %
		13.9	5150.838	50.9 %
2	chaetosemin B	12.9	10995.590	97.0 %
		14.0	344.859	3.0 %
3	(*ent*)-chaetosemin B	13.1	258.808	3.0 %
		13.9	8411.344	97.0 %

Racemic mixture prepared by mixing chaetosemin B and (*ent*)-chaetosemin B samples. Chromatogram wavelength 275 nm.

UV-vis spectra of main peaks in chromatograms.

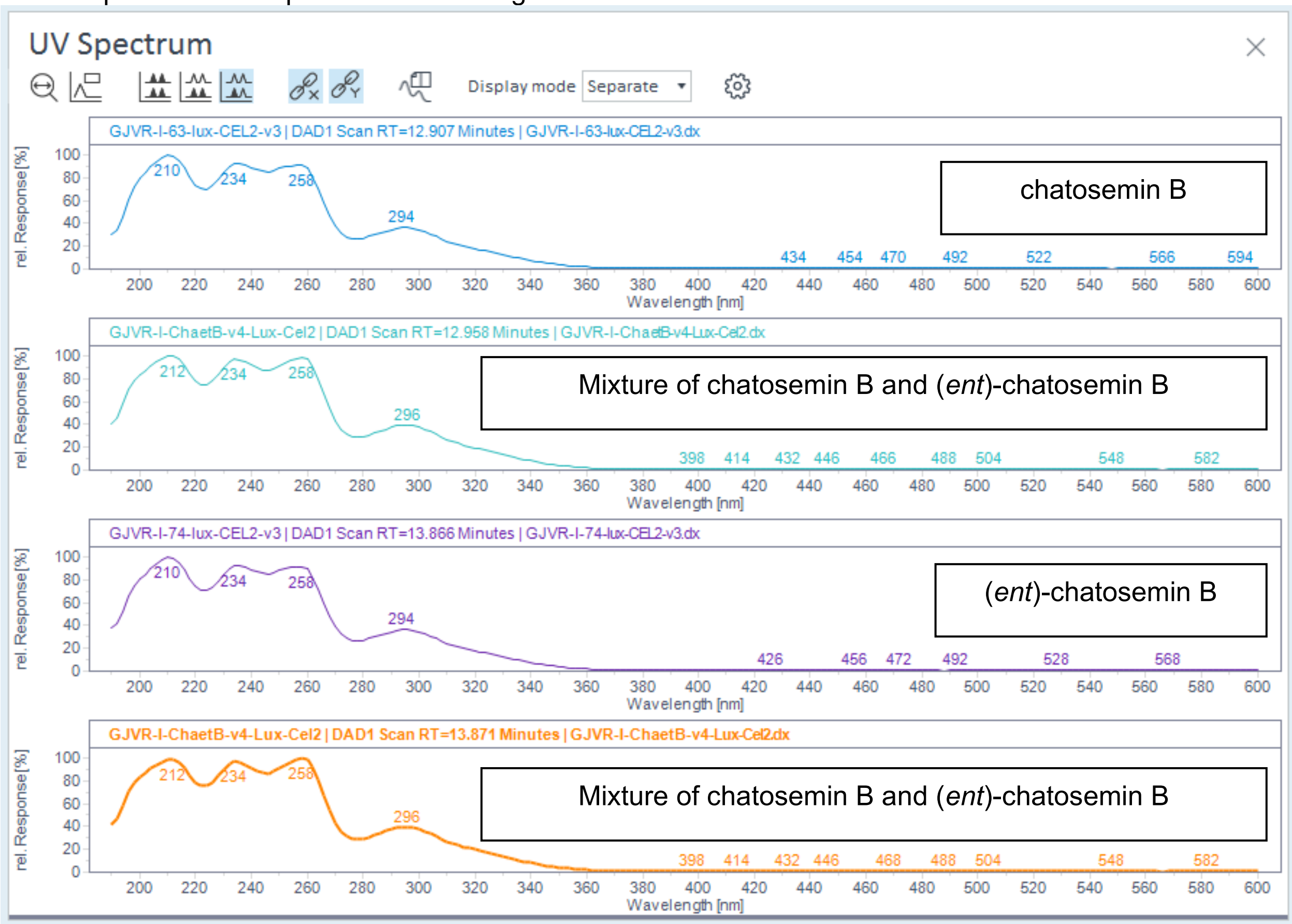

Chromatograms after recrystallization. The compounds were recrystallized by adding hexanes to a solution of chloroform.

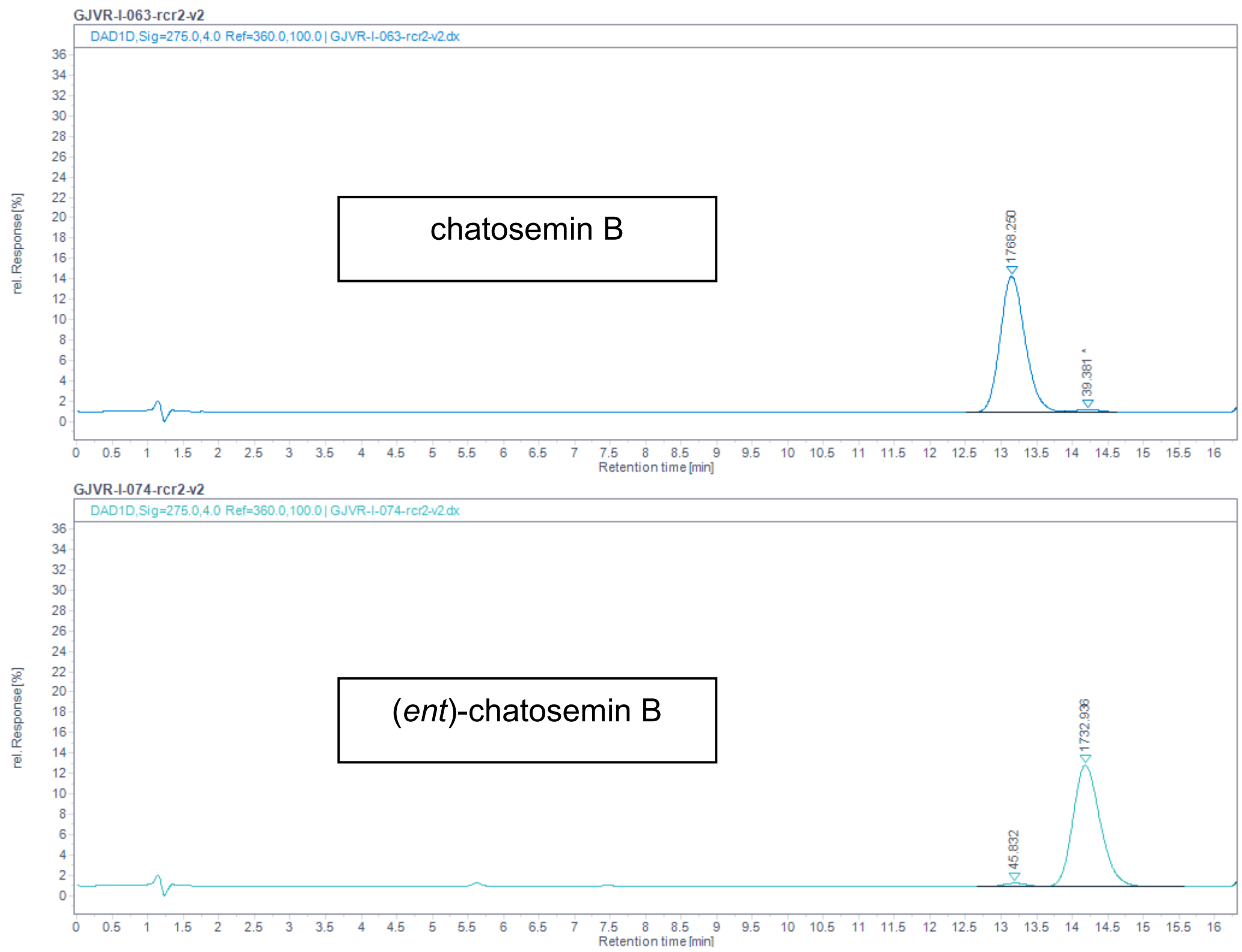

Chromatogram	Sample	RT (min)	Area (AU)	er (%)
1	chaetosemin B	13.2	1768.250	97.8 %
		14.2	39.381	2.2 %
2	(*ent*)-chaetosemin B	13.2	45.843	2.6 %
		14.2	1732.936	97.4 %

UV-vis spectra of main peaks in chromatograms.

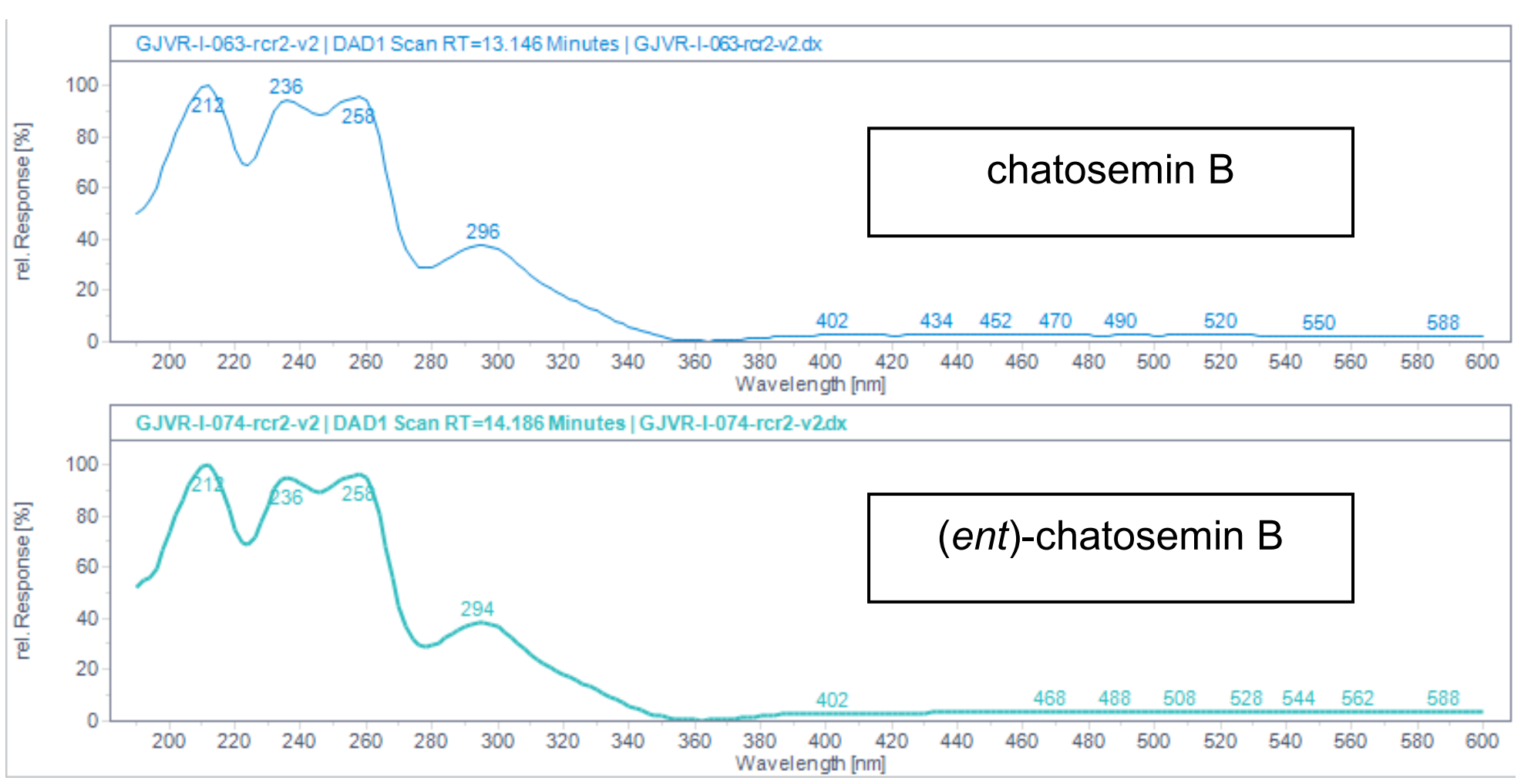

Total synthesis of chaetosemin A (**1**)

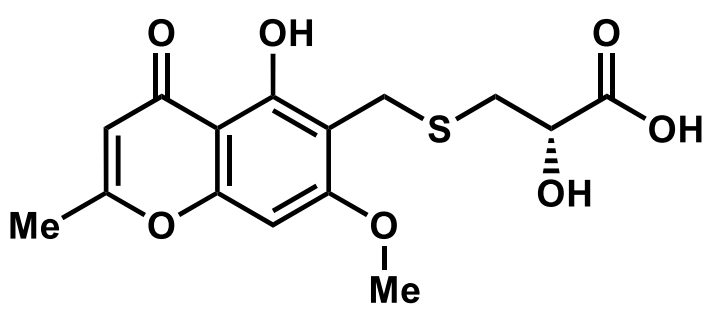

1

chaetosemin A (1): In a 20 mL vial was placed benzylic alcohol **12** (30 mg, 0.13 mmol, 1.0 equiv), thiol **19** (40 mg, 0.32 mmol, 2.5 equiv), 120 uL of formic acid, and 6 mL of acetonitrile. The reaction was flushed with nitrogen and stirred for 16 h at 65 °C. The reaction mixture was evaporated and purified by column chromatography (10–30% acetone in dichloromethane with 1% of formic acid) to yield a white solid (27 mg, 63% yield).

TLC (30% acetone in chloroform, with 1% formic acid): R_f = 0.3 (UV);
^{1}H-NMR (599 MHz, d^4-MeOH) δ 6.59 (s, 1H), 6.12 (s, 1H), 4.29 (dd, *J* = 7.6, 4.4 Hz, 1H), 3.93 (s, 3H), 3.85 (d, *J* = 13.1 Hz, 1H), 3.80 (d, *J* = 13.1 Hz, 1H), 3.01 (dd, *J* = 13.8, 4.4 Hz, 1H), 2.79 (dd, *J* = 13.8, 7.6 Hz, 1H), 2.39 (s, 3H);
^{13}C-NMR (151 MHz, d^4-MeOH) δ 184.0, 176.2, 169.6, 164.7, 159.5, 158.8, 111.8, 109.4, 105.8, 91.3, 71.6, 56.8, 37.5, 24.2, 20.3;
HRMS (ESI-TOF): m/z calculated for $C_{15}H_{17}O_7S^+$ [M+H]$^+$: 341.0690, found: 341.0691;
IR (neat, ATR): 3391(broad), 3076, 2924, 2849, 2577 (broad), 1729, 1656, 1619, 1577, 1493, 1449, 1405, 1382, 1346, 1276, 1242, 1205, 1110, 1088 cm^{-1};
$[\alpha]_D^{23}$= +11.1 (c 0.5, MeOH). Reported **$[\alpha]_D^{28}$**= +9.3 (c 0.2, MeOH).[22]

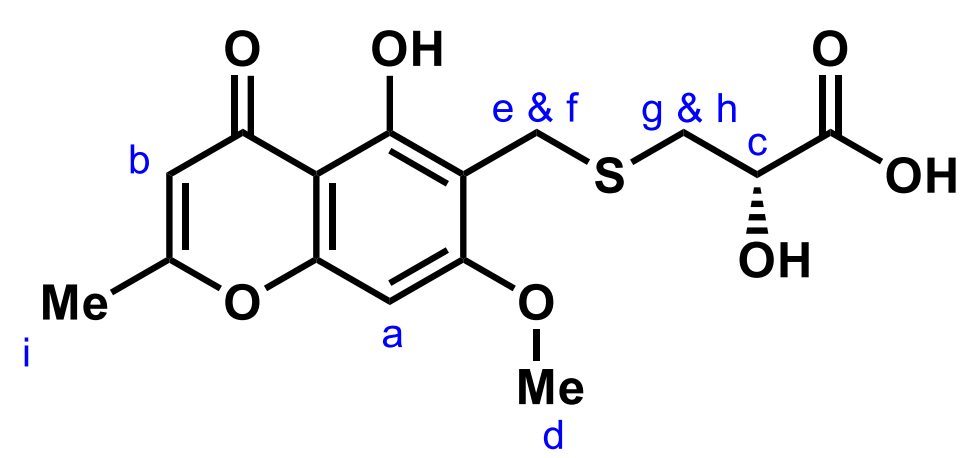

#H	Synthetic (ppm)	Isolated (ref [22] ppm)	Difference (ppm)
a	6.59 (s, 1H)	6.51 (s, 1H)	-0.08*
b	6.12 (s, 1H)	6.07 (s, 1 H)	-0.05
c	4.29 (dd, J = 7.6, 4.4 Hz, 1H)	4.30 (dd, J = 7.4, 3.4 Hz, 1H)	0.01
d	3.93 (s, 3H)	3.91 (s, 3H)	-0.02
e&f	3.85 (d, J = 13.1 Hz, 1H), 3.80 (d, J = 13.1 Hz, 1H)	3.79 (d 12.9, 2H)	-0.01**
g	3.01 (dd, J = 13.8, 4.4 Hz, 1H)	3.01 (dd, J = 13.6, 3.4 Hz, 1H)	0.0
h	2.79 (dd, J = 13.8, 7.6 Hz, 1H)	2.79 (dd, J = 13.6, 7.4, Hz, 1H)	0.0
i	2.39 (s, 3H)	2.36 (s, 3H)	-0.03

(*): The isolation report[22] shows a ^{1}H-NMR that still has a formic acid peak (8.07 ppm). Our synthesized *ent*-chaetosemin A matches with the reported data with and without formic acid. Our obtained ^{1}H-NMR s[ectra for chaetosemin A and *ent*-chaetosemin A agree within ±0.05 ppm.

(**): In the Isolation article,[22] the authors report a doublet, but their ^{1}H-NMR shows a dd at 3.75–3.84, which agrees with our results.

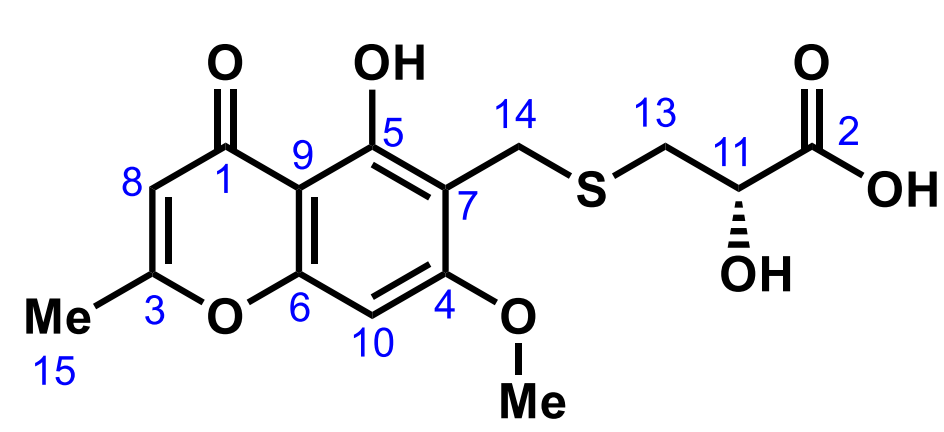

#C	Synthetic (ppm)	Isolated (ref,[22] ppm)	Difference (ppm)
1	184.0	183.9	-0.1
2	176.2	176.2	0
3	169.6	169.4	-0.2
4	164.7	164.5	-0.2
5	159.5	159.3	-0.2
6	158.8	158.6	-0.2
7	111.8	111.6	-0.2
8	109.4	109.2	-0.2
9	105.8	105.6	-0.2
10	91.3	91.2	-0.1
11	71.6	71.4	-0.2
12	56.8	56.8	0
13	37.5	37.5	0
14	24.2	24.1	-0.1
15	20.3	20.4	0.1

(*ent*)-chaetosemin A (S17)

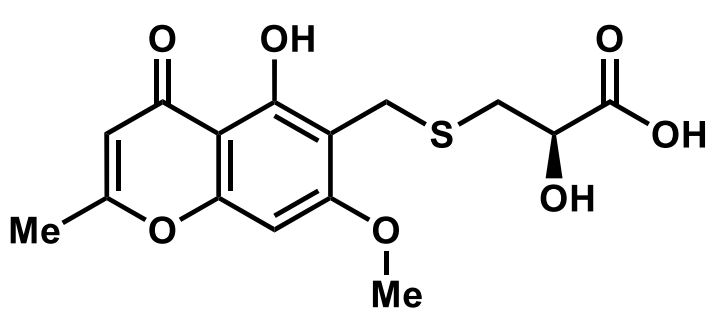

S17

(*ent*)-chaetosemin A (S17): In a 20 mL vial was placed benzylic alcohol **12** (20 mg, 0.08 mmol, 1.0 equiv), thiol **S14** (31 mg, 0.25 mmol, 3.0 equiv), 240 uL of formic acid, and 4 mL of acetonitrile. The reaction was flushed with nitrogen and stirred for 16 h at 65 °C. The reaction mixture was evaporated and purified by column chromatography (10–30% acetone in dichloromethane with 1% of formic acid) to yield a white solid (16 mg, 56% yield).

TLC (30% acetone in chloroform, with 1% formic acid): R_f = 0.3 (UV, permanganate);
^{1}H-NMR (599 MHz, MeOD) δ 6.54 (s, 1H), 6.09 (s, 1H), 4.29 (dd, *J* = 7.6, 4.4 Hz, 1H), 3.92 (s, 3H), 3.83 (d, *J* = 13.1 Hz, 1H), 3.78 (d, *J* = 13.1 Hz, 1H), 3.35 (s, 1H, OH), 3.01 (dd, *J* = 13.8, 4.4 Hz, 1H), 2.80 (dd, *J* = 13.8, 7.6 Hz, 1H), 2.38 (s, 3H);
^{13}C-NMR (151 MHz, MeOD) δ 184.0, 176.3, 169.5, 164.6, 159.5, 158.8, 111.7, 109.4, 105.7, 91.3, 71.6, 56.8, 37.6, 24.2, 20.4;
HRMS (ESI-TOF): m/z calculated for $C_{15}H_{17}O_7S^+$ [M+H]$^+$: 341.0690, found: 341.0684;
IR (neat, ATR): 3397 (broad), 3079, 2923, 2849, 2578 (broad), 1727, 1656, 1620, 1580, 1493, 1449, 1406, 1382, 1345, 1277, 1243, 1203, 1108, 1088 cm^{-1};
$[\alpha]_D^{24}$: –9.6 (c=0.005 g/mL, MeOH).

Chiral separations (UPLC-DAAD) of chaetosemin A and (*ent*)-chaetosemin A

Column: Phenomenex Lux 5 µm cellulose-4, 150 x 4.6 mm

Method:

Time (min)	Solvent A %	Solvent B %
0	60	40
1	60	40
8.5	60	40
9	0	100
9.5	0	100
10	60	40

Solvent A: Water with 0.1% trifluoroacetic acid, solvent B: acetonitrile with 0.1% trifluoroacetic acid. Flow: 1 mL/min

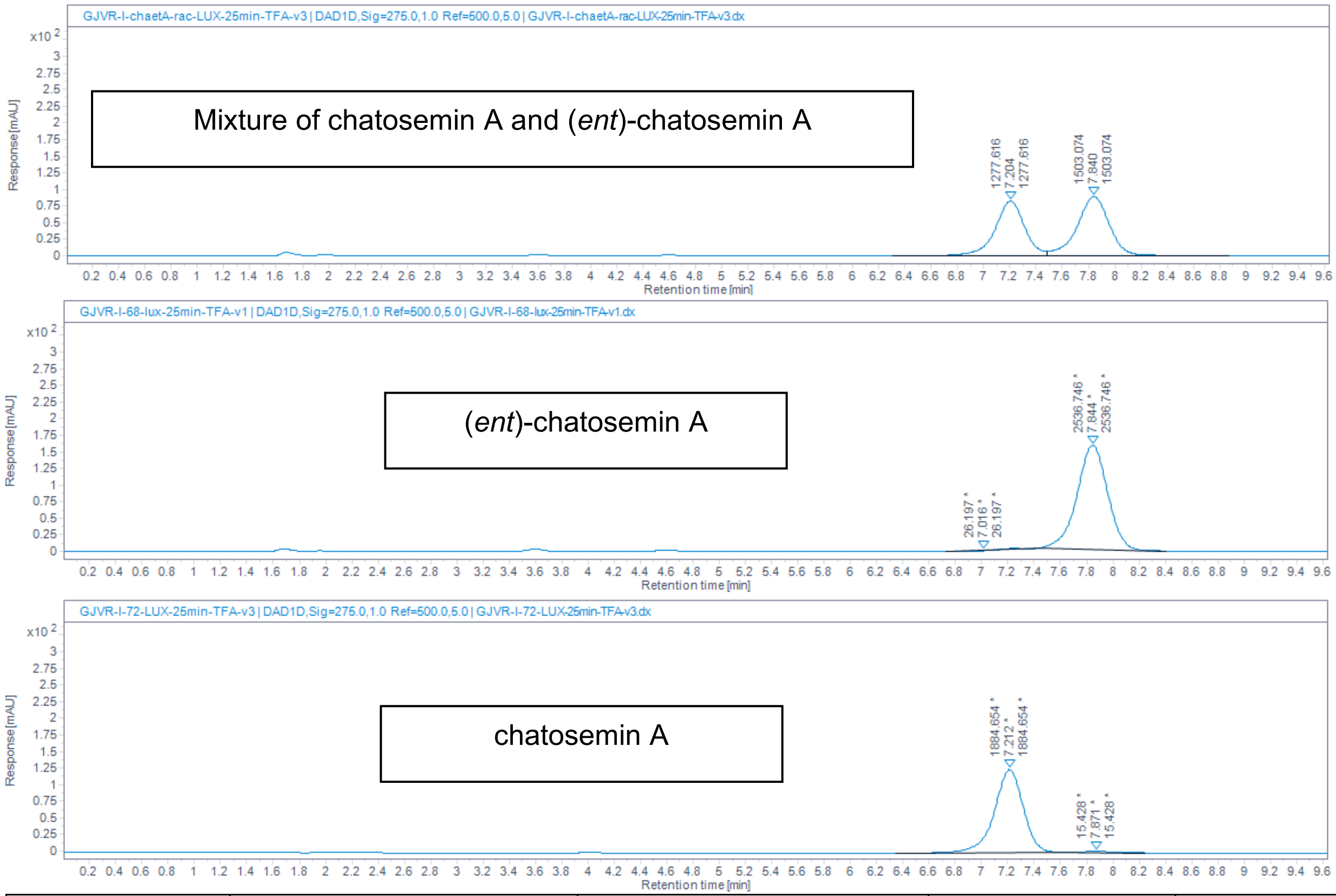

Chromatogram	Sample	RT (min)	Area (AU)	er (%)
1	Racemic mixture	7.2	1277.618	46.0 %
		7.8	1503.074	54.0 %
2	(*ent*)-chaetosemin A	7.0	26.197	1.0 %
		7.8	2536.746	99.0 %
3	chaetosemin A	7.2	1884.654	99.2 %
		7.9	15.428	0.8 %

Racemic mixture prepared by mixing chaetosemin A and (*ent*)-chaetosemin A samples. Chromatogram wavelength 275 nm.

UV-vis traces of the main 4 peaks in the chiral separation

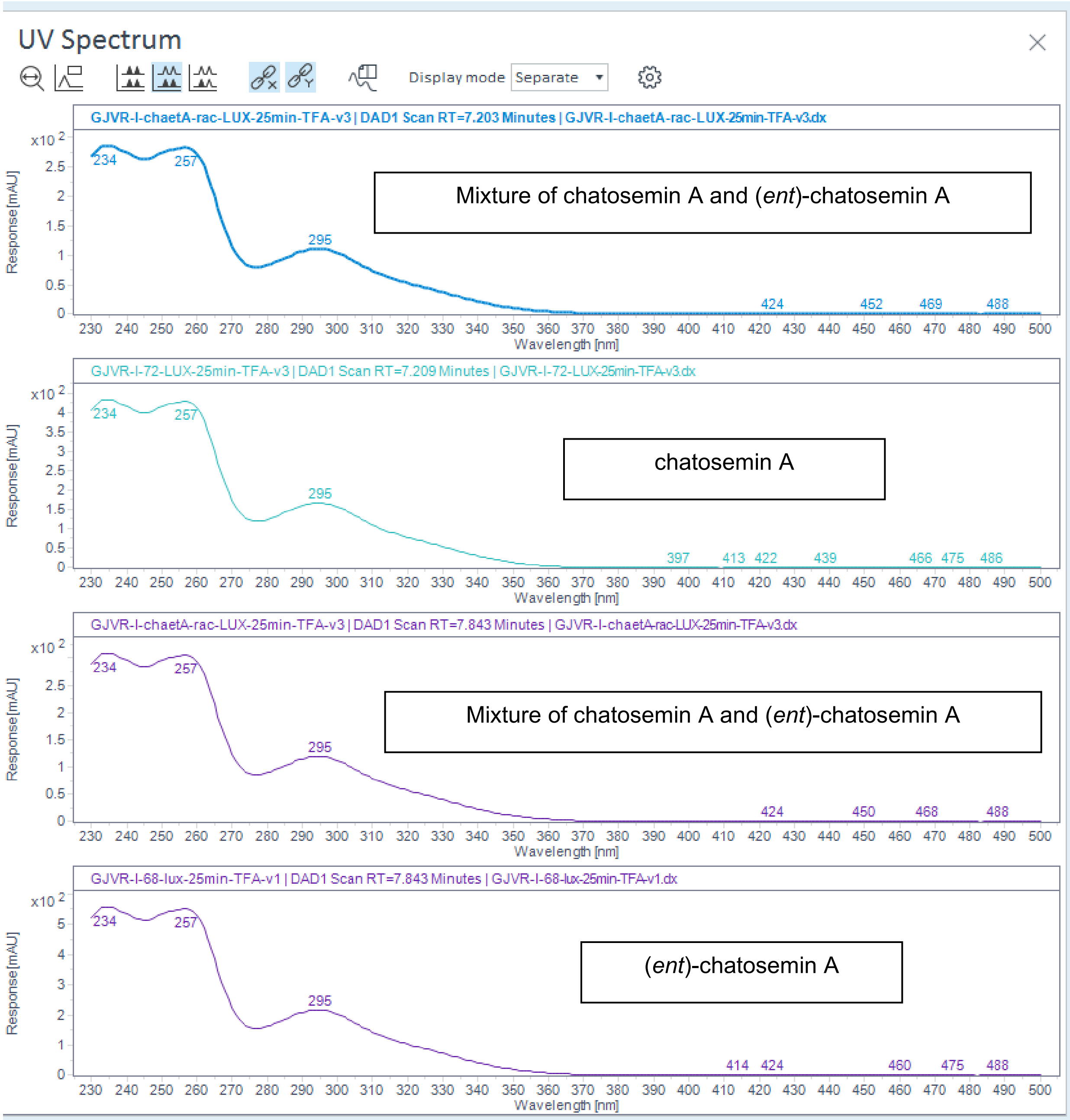

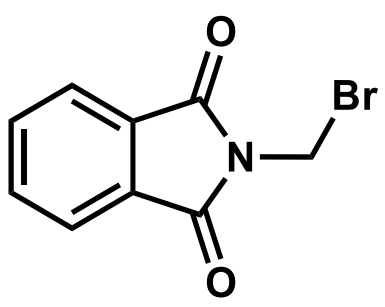

S18

2-(bromomethyl)isoindoline-1,3-dione (S18): The reactions were run under a nitrogen balloon. To a 250 mL round-bottom flask was added phtalimide (5.00 g, 136 mmol), paraformaldehyde (7.0 g, 0.23 mol, 6.9 equiv), 1 M HCl (3 mL) and DI water (35 mL). This mixture was stirred overnight at 100 °C. A white solid formed, which was filtered after cooling the mixture. The obtained white solid was placed in a 250 mL round-bottom flask with 47% aq. HBr (25 mL), and conc. H_2SO_4 (9 mL). The reaction mixture was allowed to stir at 70 °C for 5 h. The reaction was then placed in an ice bath and quenched with 20 mL of DI water. A solid precipitated, which was filtered, and washed with cold water (20 mL), 10% aq. ammonium hydroxide (10 mL) and cold water (20 mL). The obtained white solid (5.7 g, 70% yield) was used in the following step without further purification. Spectroscopic data agreed with a previous report.[23]

^{1}H-NMR (599 MHz, $CDCl_3$) δ 7.96–7.92 (m, 2H), 7.82–7.76 (m, 2H), 5.49 (s, 2H).

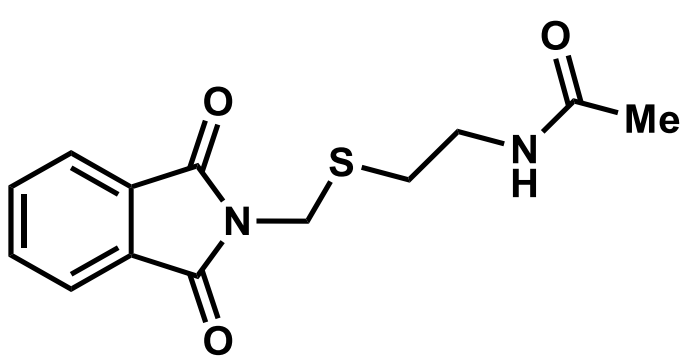

S19

***N*-(2-(((1,3-dioxoisoindolin-2-yl)methyl)thio)ethyl)acetamide (S19):** In a 250 mL round-bottom flask was placed 5.1 g of 2-(bromomethyl)isoindoline-1,3-dione (**S18**) (21 mmol, 1.0 equiv), 2.5 g of *N*-acetylcysteamine (21 mmol, 1.0 equiv), 5.8 g of potassium carbonate (42 mmol, 2.0 equiv), and 80 mL of acetonitrile. The reaction mixture was allowed to stir for 1 h. Then 100 mL of water was added, and the mixture was cooled under ice bath. A white solid precipitated, which was filtered, washed with water, acetone and hexanes. The obtained white solid (4.0 g, 68% yield) was used without further purification. A portion was recrystallized by dissolving in methanol and adding water, the obtained solid was used for characterization.

^{1}H-NMR (599 MHz, $CDCl_3$) δ 7.89 (dd, *J* = 5.5, 3.0 Hz, 2H), 7.76 (dd, *J* = 5.4, 3.0 Hz, 2H), 5.96 (bs, 1H), 4.78 (s, 2H), 3.56 (q, *J* = 6.0 Hz, 2H), 2.85 (t, *J* = 6.3 Hz, 2H), 1.99 (s, 3H);
^{13}C-NMR (151 MHz, $CDCl_3$) δ 170.4, 170.3, 167.7, 134.6, 132.1, 123.8, 38.9, 38.8, 38.7, 32.1, 32.1, 23.4, 23.3. note: We assume that we have extra carbon peaks because of the two possible amide conformers;
HRMS (ESI-TOF): m/z calculated for $C_{13}H_{15}N_2O_3S^+$ $[M+H]^+$: 279.0798, found: 279.0807;
IR (neat, ATR): 3280, 3063, 2986, 2937, 1707, 1638, 1551, 1416, 1380, 1307, 1286, 1087 cm^{-1}.

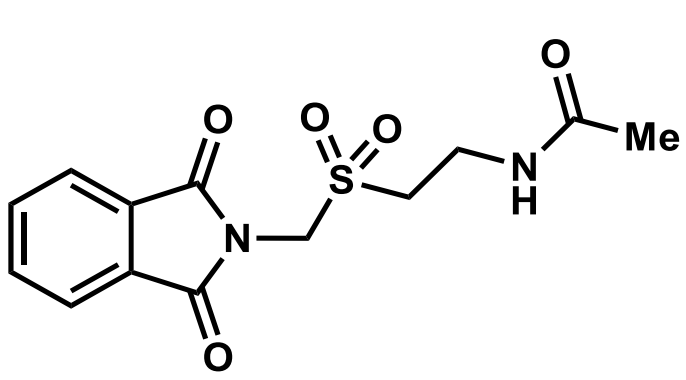

S20

***N*-(2-(((1,3-dioxoisoindolin-2-yl)methyl)sulfonyl)ethyl)acetamide (S20):** In a 50 mL round-bottom flask was placed N-(2-(((1,3-dioxoisoindolin-2-yl)methyl)thio)ethyl)acetamide (**S19**) (2.0 g, 7.2 mmol, 1.0 equiv), $MnSO_4 \cdot H_2O$ (12 mg, 0.072 mmol, 0.01 equiv), 150 mL of MeCN and allowed to stir under ice bath. Subsequently, 30% H_2O_2 (2.2 mL, 22 mmol, 3 equiv) and 0.2 M $NaHCO_3$ (2.0 g of $NaHCO_3$ in 120 mL of water) were combined under ice and added to the reaction mixture under ice. After 15 min, the solution was diluted with 1 volume of brine and extracted 3x with EtOAc. The combined organic layers were evaporated under reduced pressure to yield a crude solid, which was purified by column chromatography (silica, 10% methanol/dichloromethane) and used in further steps. The title compound was obtained as a white solid (1.9 g, 85% yield). A portion of the product was further purified by recrystallization with THF/methanol and a few drops of hexanes and used for characterization.

^{1}H-NMR (599 MHz, $CDCl_3$) δ 7.95 (dd, *J* = 5.5, 3.1 Hz, 2H), 7.83 (dd, *J* = 5.5, 3.0 Hz, 2H), 4.98 (s, 2H), 3.89 – 3.80 (m, 2H), 3.34 (dd, *J* = 6.6, 4.7 Hz, 2H), 1.99 (s, 3H);
^{13}C-NMR (151 MHz, $CDCl_3$) δ 166.4, 135.2, 131.5, 124.5, 56.4, 53.9, 33.0, 32.8, 23.3, 23.3. note: We assume that we have extra carbon peaks because of the two possible amide conformers. We did not find the low intensity amide peak.
HRMS (ESI-TOF): m/z calculated for $C_{13}H_{15}N_2O_5S^+$ $[M+H]^+$: 311.0696, found: 311.0705;
IR (neat, ATR): 3421, 2920, 2908, 1718, 1666, 1527, 1387, 1294, 1137, 1067, 916, 718 cm^{-1}.

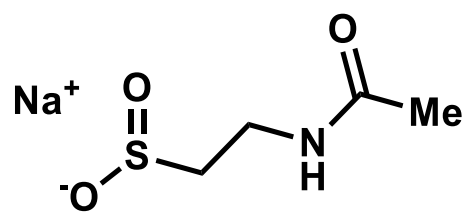

17

sodium 2-acetamidoethane-1-sulfinate (17): In a 100 mL oven-dried round-bottom flask was placed N-(2-(((1,3-dioxoisoindolin-2-yl)methyl)sulfonyl)ethyl)acetamide (**S20**) (1.9 g, 6.1 mmol, 1.0 equiv), NaOMe (500 mg, 9.3 mmol, 1.5 equiv), and 40 mL of dry methanol. The reaction was refluxed for 16 h. Methanol was evaporated after cooling down, then dichloromethane was added to the obtained solid. This mixture was filtered and washed with dichloromethane and hexanes. To transfer all the solid, it was dissolved with methanol and evaporated. Toluene was added to the obtained solid and the mixture was concentrated once again. The obtained white solid (875 mg, 83% yield) was used directly without further purification. A portion was recrystallized with methanol/acetone and used for characterization.

^{1}H-NMR (599 MHz, MeOH-d_4) 8.55 (s, 0.5H), 3.48 (t, *J* = 6.9 Hz, 2H), 2.42 (t, *J* = 6.9 Hz, 2H), 1.92 (s, 3H). Because of the 0.5 integration of the peak at 8.55 ppm we think there is another amide N–H peak. We might not be able to see the amide peak from this conformer because it is too broad;
^{13}C-NMR (151 MHz, MeOH-d_4) δ 173.0, 61.4, 35.0, 22.6;
HRMS (ESI-TOF): m/z calculated for $C_4H_{10}NO_3S$+ $[M+H]^+$= 152.0376, found: 152.0377;
IR (neat, ATR): 3275 (br), 3090, 2808, 1600 (br, strong), 1481, 1437, 1358, 1303, 1024, 960 cm^{-1}.

Total synthesis of chaetoquadrin D (**3**)

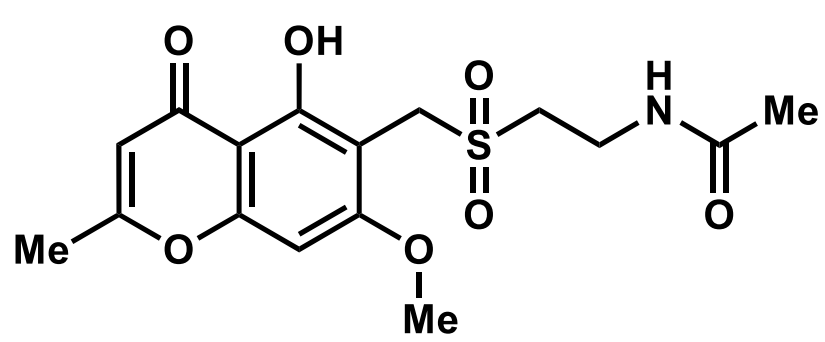

3

chaetoquadrin D (3): In a 20 mL vial was placed benzylic alcohol **12** (20 mg, 0.085 mmol, 1.0 equiv), sulfinate **17** (73 mg, 0.42 mmol, 5.0 equiv), 40 uL of formic acid and 3 mL of acetonitrile. The mixture was taken to 95 °C and allowed to stir for 14 h. The mixture was cooled down, concentrated under reduced pressure and partitioned with EtOAc and water. The aqueous layer was extracted 3x with EtOAc, the combined organic layers were dried with Na_2SO_4, filtered, concentrated, and purified by column chromatography (silica, EtOAc/Hex 20–50%, then acetone/Hex 50–70%). The obtained solid was further purified by prep-HPLC (MeCN/water) to yield the title compound as a white powder (19 mg, 61% yield). Spectroscopic data agree with the isolation report.[24]

TLC (60% acetone/hexanes): R_f=0.6 (permanganate, UV);
^{1}H-NMR (599 MHz, $CDCl_3$): δ 13.43 (s, 1H), 6.44 (s, 1H), 6.30 (br, 1H, N-H), 6.10 (s, 1H), 4.45 (s, 2H), 3.95 (s, 3H), 3.82 – 3.76 (m, 2H), 3.24 – 3.19 (m, 2H), 2.38 (s, 3H), 1.96 (s, 3H);
^{13}C-NMR (151 MHz, $CDCl_3$): δ 182.4, 170.3, 167.4, 163.8, 160.1, 158.7, 109.4, 105.2, 100.4, 90.6, 56.7, 52.9, 48.8, 33.0, 23.4, 20.7;
HRMS (ESI-TOF): m/z calculated for $C_{16}H_{20}NO_7S$ $[M+H]^+$=370.0955, found= 370.0959. m/z calculated for $C_{16}H_{19}NO_7SNa$ $[M+Na]^+$=392.0774, found=392.0778;
IR (neat, ATR): 3363, 3292, 3079, 2924, 2852, 1656, 1624, 1587, 1548, 1495, 1448, 1346, 1300, 1206, 1182, 1129, 1105, 1090, 842, 816, 733 cm^{-1}.

^{1}H-NMR

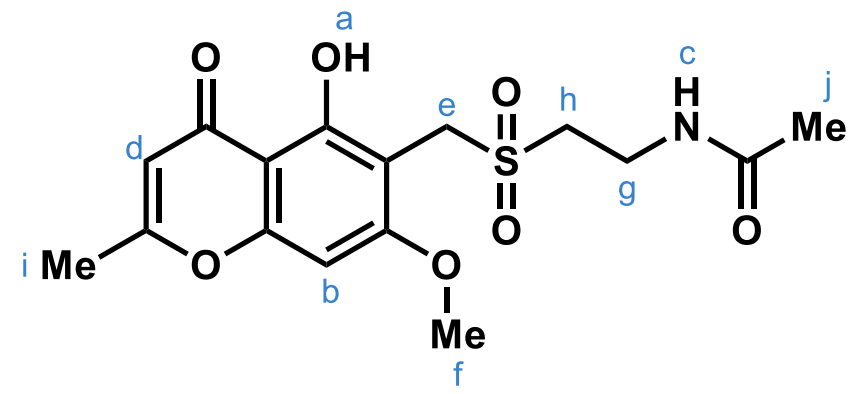

#H	Synthetic (ppm)	Isolated (ref[24], ppm)	Difference (ppm)
a	13.43 (s, 1H)	13.40 (s, 1H)	-0.03
b	6.44 (s, 1H)	6.43 (s, 1H)	-0.01
c	6.30 (s, 1H, N-H)	6.31 (br, s, 1H)	+0.01
d	6.10 (s, 1H)	6.08 (s, 1H)	-0.02
e	4.45 (s, 2H)	4.43 (s, 2H)	-0.02
f	3.95 (s, 3H)	3.93 (s, 3H)	-0.02
g	3.80 – 3.76 (m, 2H)	3.78 (m, 2H)	
h	3.24 – 3.19 (m, 2H)	3.20 (m, 2H)	
i	2.38 (s, 3H),	2.36 (s, 3H)	-0.02
j	1.96 (s, 3H).	1.94 (s, 3H)	-0.02

^{13}C-NMR

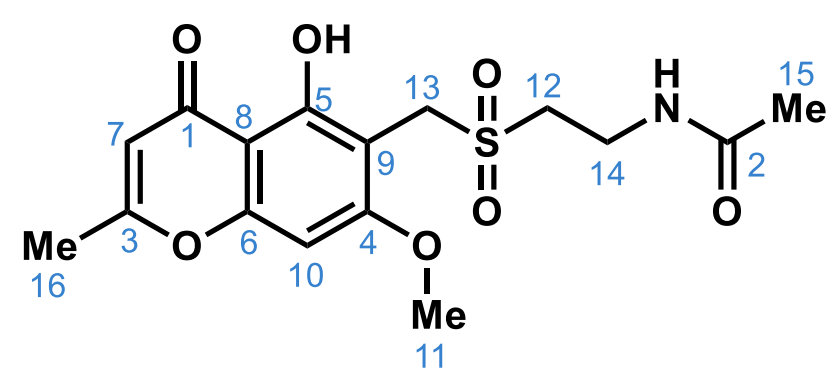

#C	Synthetic	Isolated (ref[24], ppm)	Difference
1	182.4	182.2	-0.2
2	170.3	170.2	-0.1
3	167.4	167.3	-0.1
4	163.8	163.6	-0.2
5	160.1	160.0	-0.1
6	158.7	158.5	-0.2
7	109.4	109.2	-0.2
8	105.2	105.1	-0.1
9	100.4	100.2	-0.2
10	90.6	90.4	-0.2
11	56.7	56.5	-0.2
12	52.9	52.7	-0.2
13	48.8	48.6	-0.2
14	33.0	32.8	-0.2
15	23.4	23.2	-0.2
16	20.7	20.5	-0.2

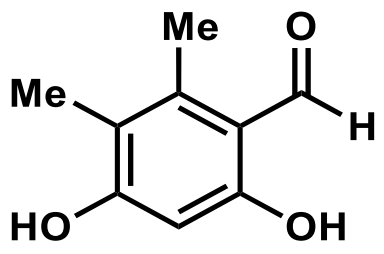

8

4,6-Dihydroxy-2,3-dimethylbenzaldehyde (8): To a dried flask with magnetic stir bar was added 4,5-dimethylbenzene-1,3-diol (200 mg, 1.45 mmol) and the atmosphere was replaced with nitrogen. Dichloromethane (15 mL) was added, and the mixture was cooled to –78 °C, followed by addition of dichloromethyl methyl ether (260 µL, 2.89 mmol, 2.0 equiv). $TiCl_4$ (960 µL, 8.68 mmol, 6 equiv) was added dropwise and the mixture was allowed to warm to room temperature and stired for 12 h. The reaction was quenched by the addition of water (15 mL). The layers were separated, and the organic phase was extracted with dichloromethane (2 x15 mL). The combined organic extracts were washed with brine (15 mL), dried over anhydrous sodium sulfate, filtered, and concentrated under reduced pressure. The product was purified by flash chromatography (silica, gradient elution 10% to 50% ethyl acetate in hexanes) to afford the title compound (193 mg, 80% yield) as an amorphous tan solid. The spectroscopic data agreed with a previous report.[25]

^{1}H-NMR (599 MHz, DMSO-d_6): δ 12.22 (s, 1H), 10.72 (s, 1H), 10.13 (s, 1H), 6.22 (s, 1H), 2.41 (s, 3H), 1.99 (s, 3H);
^{1}H-NMR (599 MHz, MeOH-d_4): δ 10.14 (s, 1H), 6.15 (s, 1H), 2.46 (s, 3H), 2.07 (s, 3H);
^{13}C-NMR (150 MHz, MeOH-d_4): δ 194.9, 165.4, 143.4, 117.7, 114.1, 100.6, 14.0, 10.9;
HRMS (ESI-TOF): m/z calculated for $C_9H_{11}O_3^+$ $[M+H]^+$= 167.0703, found= 167.0709.

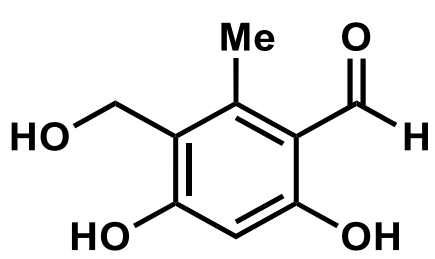

13

4,6-Dihydroxy-3-(hydroxymethyl)-2-methylbenzaldehyde (13): A 2.8 L flask was charged with water (376 mL), TES buffer pH 7.5 (24.1 mL of a 1 M aqueous solution, final concentration of 50 mM), sodium ascorbate (3.9 mL of a 0.5 M aqueous solution, final concentration of 4 mM, 1.6 equiv), α-ketoglutaric acid (3.9 mL of a 0.5 M aqueous solution, final concentration of 4 mM, 1.6 equiv), ferrous sulfate heptahydrate (1.9 mL of a 0.05 M aqueous solution, final concentration of 0.2 mM, 0.08 equiv), substrate **8** (200 mg pre-dissolved in 24 mL DMSO), whole cell wet pellet of *E. coli* containing with NHI_B8 (24.0 g, final concentration of pellet 50 mg/mL), and toluene (48 mL, 10% v/v). The flask capacity should be at least 3 times the total reaction volume to allow for sufficient oxygenation. The flask was shaken at 200 rpm at 30 °C for 3 h, then quenched with acetone (900 mL, ~2 volumes) and allowed to sit for 1 h. The mixture was centrifuged (20 min, 5000 x g) and the acetone was removed from the supernatant under reduced pressure. The resulting aqueous mixture was extracted with ethyl acetate (3 x 400 mL). The combined organic extracts were washed with brine (400 mL), dried over anhydrous sodium sulfate, filtered, and concentrated under reduced pressure. The product was purified by flash chromatography (silica, gradient elution 10% to 50% ethyl acetate in hexanes) to afford the title compound (174 mg, 80% yield) as an amorphous off-white solid.

^{1}H NMR (599 MHz, d_6-DMSO): δ 12.27 (s, 1H), 10.78 (s, 1H), 10.14 (s, 1H), 6.20 (s, 1H), 4.45 (s, 2H), 2.53 (s, 3H);
^{13}C NMR (151 MHz, d_6-DMSO): δ 194.4, 164.6, 164.3, 145.0, 120.7, 113.2, 100.2, 53.7, 14.1;
HRMS (ESI-TOF): m/z calculated for $C_9H_{11}O_4^+$ $[M+H]^+$: 183.0652, found: 183.0654.

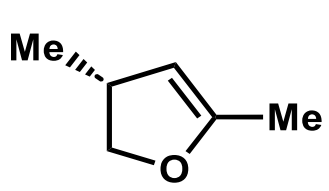

(*R*)-3,5-dimethyl-2,3-dihydrofuran (20): Prepared as previously reported.[26]

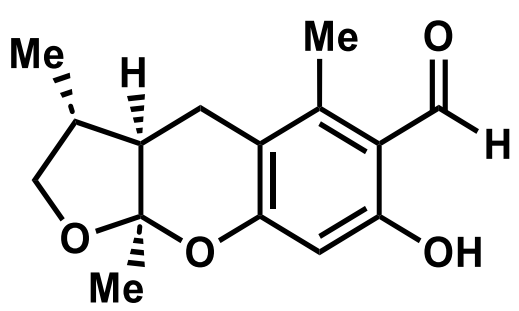

22

(3*R*,3a*R*,9a*R*)-7-hydroxy-3,5,9a-trimethyl-2,3,3a,9a-tetrahydro-4H-furo[2,3-b]chromene-6-carbaldehyde (22): A microwave vial equipped with a magnetic stir bar was charged with benzylic alcohol **13** (30 mg, 0.16 mmol, 1.0 equiv), HFIP (2.1 mL), 2-propanol (2.1 mL), and dienophile **20** (37 µL, 0.33 mmol, 2.0 equiv). The vial was sealed under air and heated to 90 °C (15 W) with stirring in a microwave reactor for 2 h. The reaction was monitored by TLC until full consumption of starting material was observed. Excess 2-propanol was removed under reduced pressure. Crude NMR indicated a 16:1 dr. The product was purified by flash chromatography (silica, gradient elution 1% to 5% ethyl acetate in hexanes) to afford the title compound (29 mg, 67% yield) as a single diastereomer.

TLC (15% EtOAc/hexanes): R_f = 0.3 (anisaldehyde, UV);
^{1}H NMR (599 MHz, $CDCl_3$): δ 12.24 (s, 1H), 10.20 (s, 1H), 6.26 (s, 1H), 4.19 (t, *J* = 8.4 Hz, 1H), 3.55 (t, *J* = 8.5 Hz, 1H), 2.80–2.72 (m, 2H), 2.46 (s, 3H), 2.13–2.04 (m, 2H), 2.00 (ddd, *J* = 11.0, 5.5, 2.3 Hz, 1H), 1.54 (s, 3H), 1.08 (d, *J* = 6.5 Hz, 3H);
^{13}C NMR (151 MHz, CDCl3): δ 193.3, 164.2, 161.3, 142.4, 114.2, 110.0, 108.2, 102.9, 74.3, 47.8, 35.2, 23.0, 21.7, 16.1, 13.6;
HRMS (ESI-TOF): m/z calculated for $C_{15}H_{19}O_4^+$ $[M+H]^+$: 263.1278, found 263.1271;
$[\alpha]_D^{23}$: –5.32 (c=0.4, $CHCl_3$);
IR (neat, ATR): 2960, 2920, 2893, 2850, 1641, 1582, 1473, 1351, 1293, 1243, 1118, 1004, 878 cm^{-1}.

Total synthesis of phomeketale B (**6**)

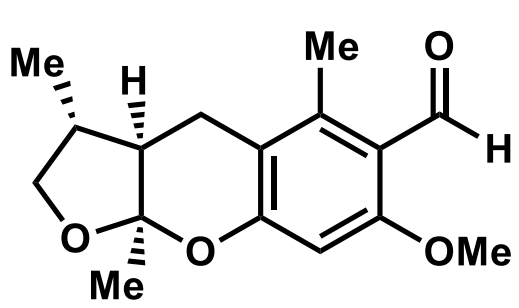

6

phomeketale B (6): To a flame dried vial with a stir bar, cycloadduct **22** (2.0 mg, 7.6 µmol), K_2CO_3 (2.1 mg, 15 µmol, 2.0 equiv), and acetone (0.19 mL, 40 mM) were added. Then, dimethyl sulfate (1.5 µL, 15 µmol, 2.0 equiv) was added, and the reaction was allowed to stir at 50 °C for 16 h. The mixture was evaporated and partitioned with DCM and water. A few drops of conc. ammonium hydroxide were added, and the mixture was stirred for 10 min. The aqueous layer was extracted with dichloromethane and the combined organic layers were washed with brine. The combined organic layers were evaporated, and the obtained crude was purified by column chromatography (silica, gradient elution 1% to 20% ethyl acetate in hexanes) to afford the title compound (1.9 mg, 91% yield). The characterization data was in agreement with the isolation report.[27]

TLC (15% EtOAc/hexanes): R_f = 0.4 (anisaldehyde, UV);
^{1}H NMR (599 MHz, DMSO-d6): 10.37 (s, 1H), 6.41 (s, 1H), 4.03 (t, *J* = 8.4 Hz, 1H), 3.80 (3H, s), 3.44 (1H, t, *J* = 8.4), 2.74 (2H, m), 2.44 (3H, s), 2.04 (1H, m), 1.88 (1H, m), 1.44 (3H, s), 1.02 (d, *J* = 6.5 Hz, 3H);
^{13}C NMR (151 MHz, DMSO-d6): 190.1, 162.8, 158.3, 141.3, 117.0, 110.8, 107.7, 98.0, 73.3, 55.9, 46.9, 34.8, 22.7, 20.7, 15.5, 15.4;
HRMS (ESI-TOF): m/z calculated for $C_{16}H_{21}O_4^+$ $[M+H]^+$: 277.1435, found 277.1430;
$[\alpha]_D^{23}$: –11.41 (c=0.5, $CHCl_3$). **$[\alpha]_D^{23}$**= –3.2 (c=0.2, MeOH). Reported **$[\alpha]_D^{20}$**= –22.25 (c=0.40, MeOH);[27]
IR (neat, ATR): 2955, 2930, 2853, 1674, 1593, 1465, 1451, 1382, 1323, 1198, 1116, 1071, 1071, 1007, 846 cm^{-1}.

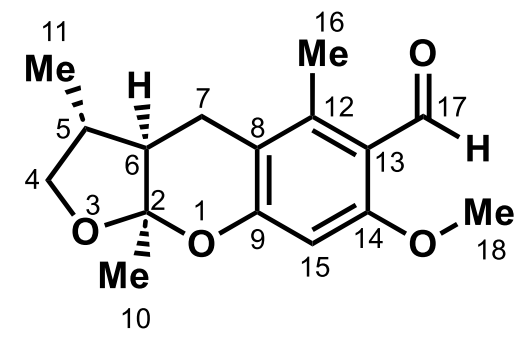

#H	Synthetic (ppm)	Isolated (ref[24,27], ppm)	Difference (ppm)
4	3.44 (1H, t, J = 8.4)<br>4.03 (1H, t, J = 8.4)	3.44 (1H, t, J=8.3),<br>4.02 (1H, t, J=8.2)	
5	1.88 (1H, m)	1.87 (1H, s)	-0.01
6	2.04 (1H, m)	2.04 (1H, m)	0
7	2.74 (2H, m)	2.73 (2H, m)	-0.01
10	1.44 (3H, s)	1.44 (3H, s)	0
11	1.02 (3H, d, J = 6.5)	1.01 (3H, d, J=6.4)	-0.01
15	6.41 (s, 1H)	6.41 (1H, s)	0
16	2.44 (3H, s)	2.44 (3H, s)	0
17	10.37 (s, 1H)	10.36 (1H, s)	-0.01
18	3.80 (3H, s)	3.80 (3H, s)	0

#C	Synthetic (ppm)	Isolated (ref[24,27], ppm)	Difference (ppm)
2	107.7	107.7	0
4	73.3	73.3	0
5	34.8	34.8	0
6	46.9	46.9	0
7	20.7	20.7	0
8	110.8	110.8	0
9	158.3	158.3	0
10	22.7	22.6	-0.1
11	15.5	15.4	-0.1
12	141.3	141.3	0
13	117.0	117.0	0
14	162.8	162.8	0
15	98.0	97.9	-0.1
16	15.4	15.5	0.1
17	190.1	190.1	0
18	55.9	55.9	0

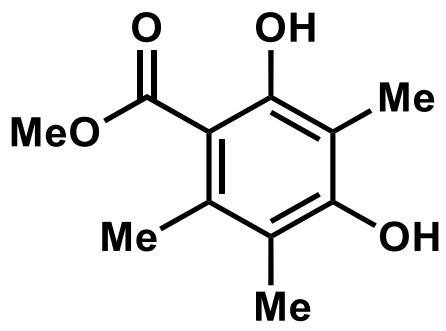

S21

methyl 2,4-dihydroxy-3,5,6-trimethylbenzoate (S21): Synthesized from methyl atratate as previously described,[28] to provide material with characterization data which agreed with a previous report.[29]

^{1}H-NMR (599 MHz, $CDCl_3$): δ 11.45 (s, 1H), 5.13 (br s, 1H), 3.93 (s, 3H), 2.43 (s, 3H), 2.14 (s, 3H), 2.14 (s, 3H).

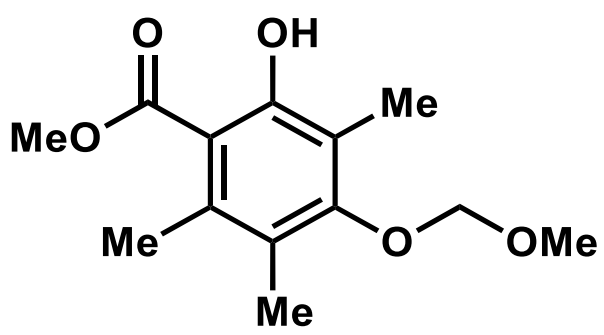

S22

methyl 2-hydroxy-4-(methoxymethoxy)-3,5,6-trimethylbenzoate (S22): In a 50 mL round-bottom flask, methyl 2,4-dihydroxy-3,5,6-trimethylbenzoate (**S21**) (4.6 g, 22 mmol, 1.0 equiv) was dissolved with 170 mL of dichloromethane. The mixture was placed in an ice bath, diisopropylethylamine (4.6 mL, 26 mmol, 1.2 equiv) and MOM-Cl (1.5 mL, 20 mmol, 0.90 equiv) were added. The reaction was allowed to stir overnight. The reaction was quenched by adding a volume of NH_4Cl sat aq, the aqueous layer was extracted 3x with dichloromethane. The combined organic layers were dried with Na_2SO_4, filtered, and concentrated. The crude mixture was purified by column chromatography (silica, 5-20% EtOAc/Hex) to afford the title compound as a slightly yellow solid (2.6 g, 47% yield).

TLC (20% EtOAc/hexanes): R_f = 0.6 (UV, CAM stain);
^{1}H-NMR (599 MHz, $CDCl_3$): δ 11.05 (s, 1H), 4.95 (s, 2H), 3.94 (s, 3H), 3.61 (s, 3H), 2.42 (s, 3H), 2.18 (s, 6H);
^{13}C-NMR (151 MHz, $CDCl_3$): δ 172.6, 159.4, 159.2, 137.4, 122.1, 116.7, 109.9, 99.5, 57.9, 52.2, 19.1, 13.5, 10.0;
HRMS (ESI-TOF): m/z calculated for $C_{13}H_{19}O_5^+$ $[M+H]^+$= 255.1227, found= 255.1233;
IR (neat, ATR): 2953, 2924, 2851, 1656, 1601, 1579, 1440, 1320, 1203, 1160, 1061, 971, 929, 804 cm^{-1}.

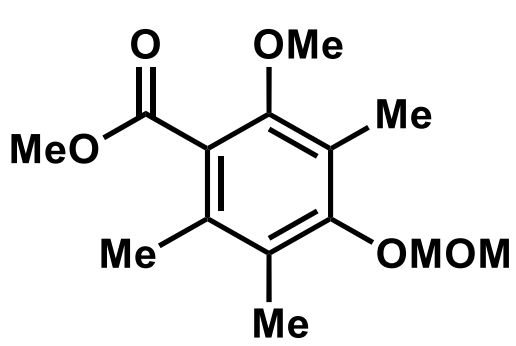

S23

methyl 2-methoxy-4-(methoxymethoxy)-3,5,6-trimethylbenzoate (S23): In 250 mL round-bottom flask was placed phenol **S22** (2.60 g, 10.2 mmol, 1.00 equiv), potassium carbonate (2.54 g, 18.4 mmol, 1.80 equiv), dimethyl sulfate (1.27 mL, 13.3 mmol, 1.30 equiv) and 34 mL of acetone. The mixture was stirred for 14 h at reflux, concentrated under reduced pressure, and diluted with water and 2 mL of conc. ammonium hydroxide. The aqueous solution was allowed to stir for 5 min and then was extracted with dichloromethane. The combined organic layers were dried with Na_2SO_4, filtered and concentrated. The obtained crude oil that was purified by column chromatography (silica, 5%–20% EtOAc/Hex) to yield the title compound as a transparent oil (2.69 g, 98% yield).

TLC (20% EtOAc/hexanes): R_f = 0.4 (UV, CAM stain);
^{1}H-NMR (599 MHz, $CDCl_3$): δ 4.92 (s, 2H), 3.92 (s, 3H), 3.74 (s, 3H), 3.61 (s, 3H), 2.21 (s, 3H), 2.17 (s, 3H), 2.16 (s, 3H);
^{13}C-NMR (151 MHz, $CDCl_3$): δ 169.5, 156.4, 153.9, 132.6, 126.4, 125.7, 122.4, 99.5, 62.0, 57.8, 52.4, 17.0, 13.2, 10.3;
HRMS (ESI-TOF): m/z calculated for $C_{14}H_{21}O_5^+$ [M+H]= 269.1384, found= 269.1385;
IR (neat, ATR): 2946, 2833, 1729, 1578, 1454, 1327, 1282, 1196, 1160, 1091, 1059, 965, 922 cm^{-1}.

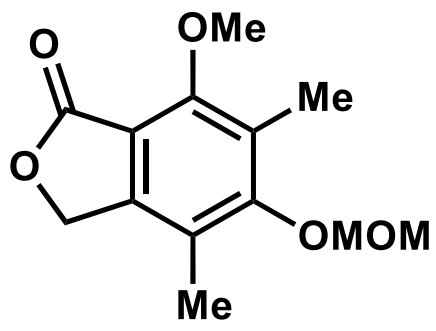

S24

7-methoxy-5-(methoxymethoxy)-4,6-dimethylisobenzofuran-1(3H)-one (S24): In a flame dried 250 mL round-bottom flask was placed 50 mL of dry THF, diisopropylamine (1.69 mL, 12.0 mmol, 1.20 equiv) and nBuLi (5.0 mL of a 2.4 M hexanes solution, 12 mmol, 1.2 equiv) at -78 °C. The mixture was warmed with an ice bath, allowed to stir for 5 min and then taken back to -78 °C. Then, **S23** (2.69 g, 10.0 mmol, 1.00 equiv) was added dropwise and the solution was allowed to stir for at least 10 min, the solution turned red. Davis oxaziridine (2.80 g, 10.7 mmol, 1.07 equiv) was dissolved in 5 mL of dry THF and added to the reaction mixture dropwise (note: the oxaziridine was dried under vacuum for 30 min before use). The solution lost its red coloration upon oxaziridine addition. After 30 min, the reaction was quenched by addition of 0.5 mL of isopropanol, and 50 mL of ammonium chloride sat. aq. solution. The mixture was extracted with EtOAc x3, the combined organic layers were washed with brine, dried with Na_2SO_4, filtered, and concentrated under reduced pressure. The crude mixture was purified by column chromatography (silica, 10-40% EtOAc/Hex) to yield the title compound as a cream-colored solid (1.8 g, 71% yield).

TLC (30% EtOAc/hexanes): R_f = 0.4 (UV);
^{1}H-NMR (599 MHz, $CDCl_3$): δ 5.14 (s, 2H), 5.02 (s, 2H), 4.04 (s, 3H), 3.63 (s, 3H), 2.24 (s, 3H), 2.19 (s, 3H);
^{13}C-NMR (151 MHz, $CDCl_3$): δ 169.1, 161.1, 156.8, 146.1, 125.7, 120.32, 112.9, 99.7, 77.4, 77.2, 77.0, 68.5, 62.4, 57.9, 12.2, 10.3;
HRMS (ESI-TOF): m/z calculated for $C_{13}H_{17}O_5^+$ [M+H]= 253.1071, found= 253.1074;
IR (neat, ATR): 2934, 1759, 1601, 1477, 1453, 1358, 1321, 1298, 1160, 1135, 1104, 1091, 1059, 991, 943 cm^{-1}.

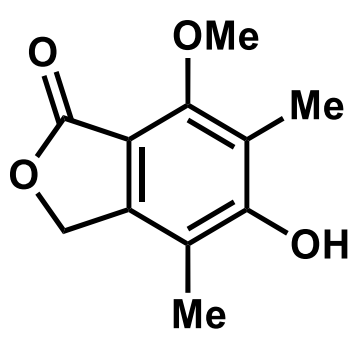

9

5-hydroxy-7-methoxy-4,6-dimethylisobenzofuran-1(3H)-one (9): In a 500 mL round-bottom flask was placed lactone **S24** (2.39 g, 9.47 mmol, 1.00 equiv), pyridinium p-toluenesulfonate (11.9 g, 47.4 mmol, 5.01 equiv), and 100 mL of isopropanol. The reaction mixture was refluxed for 2 h. Brine was added after the mixture was cooled down. The mixture was extracted with EtOAc x3, the combined organic layers were dried with Na_2SO_4, filtered, and concentrated under reduced pressure. The crude was purified by column chromatography (silica, 30-80% EtOAc/Hex) to yield the title compound as a cream-colored solid (1.6 g, 81% yield).

TLC (30% EtOAc/hexanes): R_f = 0.3 (UV, permanganate);
^{1}H-NMR (599 MHz, acetone-d_6): δ 5.18 (s, 2H), 3.96 (s, 3H), 2.18 (s, 3H), 2.16 (s, 3H);
^{13}C-NMR (151 MHz, acetone-d_6): δ 169.4, 160.3, 157.0, 147.7, 118.7, 113.7, 109.2, 68.7, 62.1, 11.2, 9.1;
HRMS (ESI-TOF): m/z calculated for $C_{11}H_{13}O_4^+$ [M+H]= 209.0808, found=209.0809;
IR (neat, ATR): 3255, 2923, 2849, 1717, 1594, 1488, 1322, 1229, 1199, 1155, 1109, 1036, 981, 910 cm^{-1}.

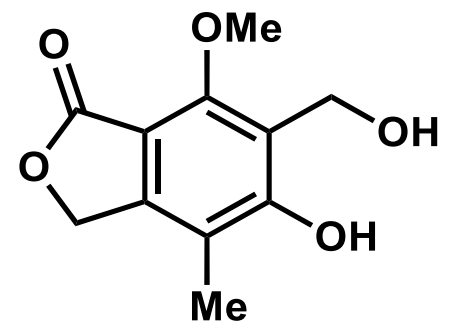

14

5-hydroxy-6-(hydroxymethyl)-7-methoxy-4-methylisobenzofuran-1(3H)-one (14): In a 2 L culture flask was placed 160 mL of MiliQ-water, 10 mL of 1 M TES buffer (final concentration 50 mM, pH=7.5), 159 mg of sodium ascorbate (final concentration 4 mM), 117 mg of α-ketoglutaric acid (final concentration 4 mM), 11 mg of $FeSO_4 \cdot 7H_2O$ (final concentration 0.2 mM) and 20 mL of toluene. 90 mg of substrate **9** were dissolved in 10 mL of DMSO and added to the prepared mixture dropwise while shacking (final substrate concentration 2.0 mM). After adding 10 g of harvested pellet for NHI_24, the reaction mixture was placed in the incubator (250 rpm, 30 °C) for 1.5 h. After confirming >90% conversion by LC-MS analysis, the reaction mixture was centrifuged (3000 x g for 15 min) and the supernatant was extracted with EtOAc x3. Isopropanol was added when an emulsion was formed. The combined organic layers were washed three times with 1:1 brine/water and twice with brine. Subsequently, the combined organic extracts were dried with Na_2SO_4, filtered, and concentrated under reduced pressure (*important*: avoid temperatures above 20 °C whenever the benzylic alcohol is concentrated). The obtained crude mixture was purified by column chromatography (silica 30-80% EtOAc/Hex) to yield the title compound as a cream-colored solid (66 mg, 68% yield).

TLC (50% acetone/hexanes): R_f = 0.5 (UV, permanganate stain);
^{1}H-NMR (599 MHz, acetone-d_6): δ 5.20 (s, 2H), 5.03 (s, 2H), 3.98 (s, 3H), 2.09 (s, 3H);
^{13}C-NMR (151 MHz, acetone-d_6): δ 169.3, 162.5, 155.6, 149.4, 118.4, 114.8, 108.4, 68.7, 62.7, 58.6, 10.5;
HRMS (ESI-TOF): m/z calculated for $C_{11}H_{11}O_4^+$ [M-H_2O+H]= 207.0652, found=207.0642;
IR (neat, ATR): 3299, 2928, 2850, 1712, 1608, 1494, 1451, 1414, 1328, 1148, 1103, 1082, 1033, 1013, 985 cm^{-1}.
Note: NOESY (*vide infra*) shows crosspeak between **^{1}H-NMR** peaks 5.20 (s, 2H) ppm and 2.09 (s, 3H) ppm, which supports the proposed structure.

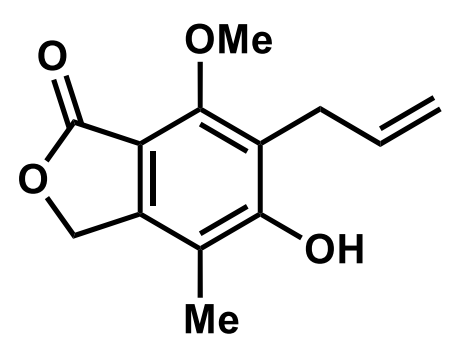

23

6-allyl-5-hydroxy-7-methoxy-4-methylisobenzofuran-1(3H)-one (23): In a 20 mL vial was placed benzylic alcohol **14** (40 mg, 0.18 mmol, 1.0 equiv), trimethyl vinyl silane (0.10 mL, 0.71 mmol, 3.9 equiv), scandium (**III**) triflate (8.0 mg, 0.016 mmol, 0.10 equiv) and 500 uL of hexafluoroisopropanol. The reaction was allowed to stir for 16 h at room temperature. The solvent was evaporated, then the mixture was partitioned with water and EtOAc, after extracting the aqueous layer with EtOAc x3, the combined organic layers were dried with Na_2SO_4, filtered and concentrated. The crude was purified by column chromatography (silica 10-30% EtOAc/Hex), to yield the title compound as a white solid (17 mg, 41% yield).

TLC (30% EtOAc/hexanes): R_f = 0.4 (UV, permanganate stain);
^{1}H-NMR (599 MHz, $CDCl_3$): δ 6.02 – 5.92 (m, 1H), 5.73 (bs, 1H), 5.24 – 5.16 (m, 2H), 5.14 (s, 2H), 4.04 (s, 3H), 3.55 (d, *J* = 6.1 Hz, 2H), 2.12 (s, 3H);
^{13}C-NMR (151 MHz, $CDCl_3$): δ 169.3, 159.1, 156.4, 147.4, 135.7, 118.5, 117.3, 113.5, 109.4, 68.4, 63.1, 28.1, 11.0;
HRMS (ESI-TOF): m/z calculated for $C_{13}H_{15}O_4^+$ [M+H]=235.0965, found=235.0972;
IR (neat, ATR): 3261, 2940, 2849, 1718, 1595, 1485, 1442, 1327, 1248, 1205, 1152, 1032, 985 cm^{-1}.

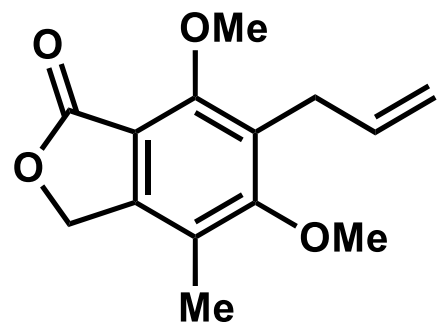

S25

6-allyl-5,7-dimethoxy-4-methylisobenzofuran-1(3H)-one (S25): In a 10 mL round-bottom flask was placed **23** (17 mg, 0.073 mmol, 1.0 equiv), potassium carbonate (19 mg, 0.14 mmol, 1.9 equiv), dimethyl sulfate (14 uL, 0.15 mmol, 2 equiv) and 1 mL of acetone. The reaction mixture was refluxed for 3 h, cooled down, and concentrated under reduced pressure. Water was added to the residue, followed by a few drops of conc. ammonium hydroxide. After stirring for less than 10 min, the aqueous solution was extracted with dichloromethane x3. The combined organic layers were dried with Na_2SO_4, filtered, and concentrated. The crude mixture was purified by column chromatography (silica, 10-20% EtOAc/Hex), to yield the title compound as a transparent oil (10 mg, 55% yield).

TLC (30% EtOAc/hexanes): R_f = 0.5 (UV);
^{1}H-NMR (599 MHz, $CDCl_3$): δ 6.03–5.93 (m, 1H), 5.15 (s, 2H), 5.05–4.95 (m, 2H), 4.07 (s, 3H), 3.79 (s, 3H), 3.47 (d, *J* = 6.0 Hz, 2H), 2.19 (s, 3H);
^{13}C-NMR (151 MHz, $CDCl_3$): δ 169.1, 163.0, 157.0, 147.2, 137.1, 127.5, 120.1, 115.4, 112.7, 68.5, 63.0, 61.4, 28.7, 11.7;
HRMS (ESI-TOF): m/z calculated for $C_{14}H_{17}O_4^+$ [M+H]=249.1121, found=249.1131;
IR (neat, ATR): 2935, 2888, 1753, 1635, 1599, 1471, 1358, 1309, 1210, 1129, 1106, 1071, 1033, 993, 964, 911 cm^{-1}.

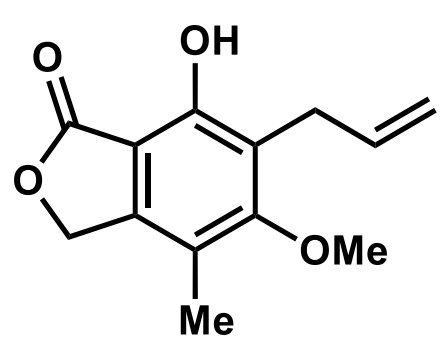

24

6-allyl-7-hydroxy-5-methoxy-4-methylisobenzofuran-1(3H)-one (24): In a 10 mL round-bottom flask was placed magnesium metal turnings (7 mg, 0.28 mmol, 13 equiv), resublimed iodine (65 mg, 0.26 mmol, 12 equiv), ether (420 uL), and benzene (700 uL). The mixture was allowed to stir for 1 h and 213 uL were transferred to a solution of **S25** (5.3 mg, 0.021 mmol, 1.0 equiv) in 1 mL of benzene. The mixture was refluxed for 1.5 h, concentrated under reduced pressure, treated with 1 M HCl aq., and extracted with dichloromethane x3. The combined organic layers were dried with Na_2SO_4, filtered, and concentrated. The crude mixture was purified by column chromatography (5-30% EtOAc/Hex) to yield the title compound as a white solid (1 mg, 20% yield). Characterization data agrees with a previously reported intermediate for the synthesis of mycophenolic acid.[30]

TLC (30% EtOAc/hexanes): R_f = 0.4 (UV). Note: the product has the same R_f as the starting material;
^{1}H-NMR (599 MHz, $CDCl_3$): δ 6.04–5.96 (m, 1H), 5.21 (s, 2H), 5.06–5.03 (m, 1H), 5.03–5.00 (m, 1H), 3.79 (s, 3H), 3.46 (d, *J* = 6.2, 2H), 2.16 (s, 3H);
^{13}C-NMR (151 MHz, $CDCl_3$): δ 173.1, 163.9, 153.9, 144.5, 136.0, 120.8, 117.0, 115.4, 106.5, 70.2, 61.4, 27.9, 11.7.
HRMS (ESI-TOF): m/z calculated for $C_{13}H_{15}O_4^+$ [M+H]=235.0965, found=235.0972;
IR (neat, ATR): 3427, 2923, 2853, 1736, 1624, 1454, 1411, 1367, 1328, 1216, 1142, 1105, 1075, 1027, 967 cm^{-1}.

Table comparing NMR data with previous report.

#H	This work (ppm)	Reference[30] (ppm)	Difference (ppm)
a	6.04–5.96 (m, 1H)	6.0 (m, 1H)	
b	5.21 (s, 2H)	5.22 (s, 2H)	0.01
c	5.06–5.03 (m, 1H)	5.06 (m, 1H)	
d	5.03–5.00 (m, 1H)	5.01 (m, 1H)	
e	3.79 (s, 3H)	3.80 (s, 3H)	0.01
f	3.46 (d, *J* = 6.2 Hz, 2H)	3.46 (dt, *J* = 6, 2 Hz, 2H)	0.00
g	2.16 (s, 3H)	2.17 (s, 3H)	0.01

#C	This work (ppm)	Reference[30] (ppm)	Difference (ppm)
1	173.1	172.9	-0.2
2	164.0	163.8	-0.2
3	153.9	153.7	-0.2
4	144.5	144.4	-0.1
5	136.0	135.9	-0.1
6	120.9	120.6	-0.3
7	117.0	116.9	-0.1
8	115.4	115.2	-0.2
9	106.5	106.4	-0.1
10	70.2	70.1	-0.1
11	61.4	61.3	-0.1
12	27.9	27.7	-0.2
13	11.7	11.6	-0.1

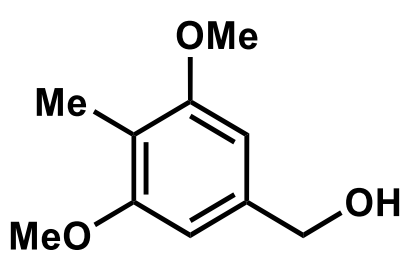

S26

(3,5-Dimethoxy-4-methylphenyl)methanol (S26): In a 100 mL flame dried flask was placed 1.0 g (5.1 mmol, 1.0 equiv) of 3,5-dimethoxy-4-methylbenzoic acid and 25 mL of dry THF. The mixture was placed in an ice bath and 8 mL of a borane-dimethylsulfide solution was added dropwise (2 M in THF, 16 mmol, 3.1 equiv). The reaction was taken to reflux and allowed to stir overnight. The reaction mixture was cooled with an ice bath and 30 mL of MeOH was added portionwise, bubbling was observed. The mixture was concentrated under reduced pressure to afford a white solid (680 mg, 70% yield). The spectroscopic data agreed with previous report.[12] The compound was used in following steps without further purification.

^{1}H-NMR (599 MHz, $CDCl_3$): δ 6.56 (s, 2H), 4.66 (s, 2H), 3.83 (s, 6H), 2.08 (s, 3H).

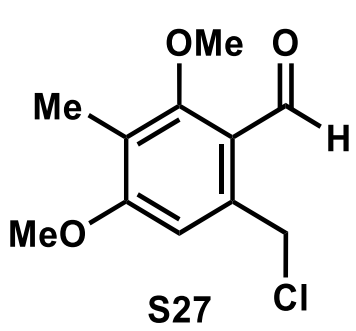

S27

6-(chloromethyl)-2,4-dimethoxy-3-methylbenzaldehyde (S27): In 500 mL flame dried flask was placed 200 mL of dry dichloromethane and cooled with brine/ice bath. To liquid was added dichloromethyl methyl ether (1.5 mL, 16 mmol, 1.5 equiv), and then 1.5 mL of $TiCl_4$ (13 mmol, 1.2 equiv). Subsequently, **S26** (2.0 g, 11 mmol, 1.0 equiv) was dissolved in 20 mL of DCM and the solution was added dropwise to the reaction mixture. The reaction was kept in an ice bath and after 30 min it was poured into ice and then extracted with DCM. The combined organic extracts were washed with saturated aqueous sodium bicarbonate, dried with $MgSO_4$, filtered and concentrated. The crude mixture was purified by column chromatography (silica, 5-20% EtOAc/Hex) to afford the title compound as a cream-colored solid (1.5 g, 60% yield).

TLC (20% EtOAc/hexanes): R_f = 0.54 (UV, DNP);
^{1}H-NMR (599 MHz, $CDCl_3$): δ 10.39 (s, 1H), 6.92 (s, 1H), 5.07 (s, 2H), 3.94 (s, 3H), 3.84 (s, 3H), 2.16 (s, 3H);
^{13}C-NMR (151 MHz, $CDCl_3$): δ 191.0, 164.5, 163.2, 139.2, 120.3, 120.1, 108.5, 63.5, 56.1, 44.8, 8.7;
HRMS (ESI-TOF): m/z calculated for $C_{11}H_{14}ClO_3^+$ [M+H] = 229.0626, found = 229.0643;
IR (neat, ATR): 2953, 2916, 2880, 2849, 1668, 1595, 1567, 1442, 1419, 1384, 1319, 1303, 1262, 1223, 1122, 1079, 1002, 939, 903 cm^{-1}.

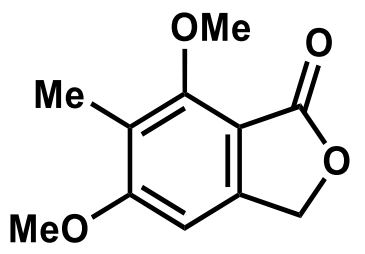

S28

5,7-dimethoxy-6-methylisobenzofuran-1(3H)-one (S28): In a 500 mL round bottom flask was placed 16.6 mL of water and 66.4 mL of THF, 3.8 g of aldehyde **S27** (17 mmol, 1.0 equiv), 7.8 g of $NaH_2PO_4{\cdot}2H_2O$ (50 mmol, 3.0 equiv) and 5.3 mL of 2-methylbut-2-ene (50 mmol, 3.0 equiv). The mixture was cooled with an ice bath, then 5.6 g of $NaClO_2$ (80% w/w purity, 50 mmol, 3.0 equiv) was added portionwise and the mixture was allowed to warm to room temperature. After stirring at room temperature for 3 h, 50 mL of THF was added and the mixture was washed twice with brine. To the THF layer was added 11 g of K_2CO_3 (83 mmol, 5.0 equiv) and the mixture was refluxed for 18 h. Subsequently, the THF was evaporated under reduced pressure, the remaining solid was partitioned with water and EtOAc, and the aqueous layer was extracted three times with EtOAc. The combined organic layers were dried with $MgSO_4$, filtered and concentrated. The crude product was purified by column chromatography (silica, 20-40% EtOAc/Hex) to furnish the title compound as a white solid (2.2 g, 64% yield). The spectroscopic data agreed with previous report.[31]

TLC (40% EtOAc/hexanes): R_f = 0.39 (UV);
^{1}H-NMR (599 MHz, $CDCl_3$): δ 6.63 (s, 1H), 5.18 (s, 2H), 4.04 (s, 3H), 3.91 (s, 3H), 2.15 (s, 3H);
^{13}C-NMR (151 MHz, $CDCl_3$): δ 169.1, 164.4, 157.9, 148.3, 120.7, 109.9, 98.6, 68.9, 62.3, 56.2, 8.7;
HRMS (ESI-TOF): m/z calculated for $C_{11}H_{13}O_4^+$ [M+H]= 209.0808, found=209.0820;
IR (neat, ATR): 2933, 2869, 2845, 1739, 1606, 1469, 1446, 1417, 1358, 1331, 1238, 1137, 1092, 1012, 987, 934 cm^{-1}.

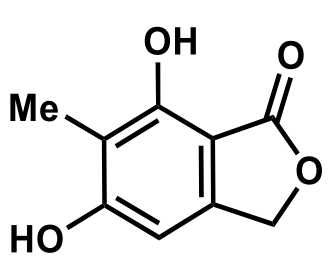

S29

5,7-dihydroxy-6-methylisobenzofuran-1(3H)-one (S29): In a 250 mL round bottom flask was placed 2.2 g of **S28** (11 mmol, 1.0 equiv) and 26 mL of dichloromethane. The mixture was cooled to -78 °C and 26 mL of 1 M solution of BBr_3 (26 mmol, 2.5 equiv) in dichloromethane was added dropwise. The reaction was allowed to warm to room temperature, and it was allowed to stir for 16 h. The reaction mixture was placed in an ice bath, and it was quench by adding 30 mL of water portionwise. The dichloromethane fraction was separated, and the aqueous layer was extracted with EtOAc, the organic layers were washed with aq. sat. $Na_2S_2O_3$, combined, dried with $MgSO_4$, filtered and concentrated. The crude mixture was flushed through a silica plug (silica, 50% EtOAc/Hex) to furnish a slightly brown solid quantitatively.

TLC (50% EtOAc/hexanes): R_f = 0.44 (UV);
^{1}H-NMR (599 MHz, $CDCl_3$): δ 7.87 (s, 1H, OH), 6.43 (s, 1H), 5.22 (s, 2H), 2.16 (s, 3H);
^{13}C-NMR (151 MHz, $CDCl_3$): δ 172.9, 161.5, 155.8, 145.5, 110.7, 104.2, 100.6, 70.4, 7.4;
HRMS (ESI-TOF): m/z calculated for $C_9H_7O_4^-$ [M-H] = 179.0350, found = 179.0348;
IR (neat, ATR): 3432, 3153 (br), 2923, 2853, 1683, 1632, 1610, 1438, 1335, 1302, 1251, 1142, 1091, 1048, 984, 975 cm^{-1}.

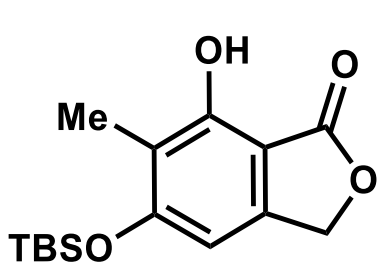

S30

5-((tert-butyldimethylsilyl)oxy)-7-hydroxy-6-methylisobenzofuran-1(3H)-one (S30): In a 250 mL round bottom flask was placed 2.5 g of **S29** (14 mmol, 1.0 equiv), and 150 mL of dichloromethane. The mixture was cooled with an ice bath followed by addition of dimethylaminopyridine (170 mg, 1.4 mmol, 0.10 equiv), imidazole (1.3 g, 19 mmol, 1.4 equiv), and *t*-butylchlorodimethylsilane (2.9 g, 19 mmol, 1.4 equiv). The reaction was allowed to warm to room temperature and was stirred for 2 h. The reaction was quenched by adding sat. aq. ammonium chloride, this mixture was extracted with dichloromethane three times. The combined organic layers were dried with $MgSO_4$, filtered and concentrated under reduced pressure to afford the crude product. The crude mixture was purified by column chromatography (silica, 5–20% EtOAc/Hex) to afford the title compound as a white solid in 36% yield (2.2 g).

TLC (20% EtOAc/hexanes): R_f = 0.51 (UV);
^{1}H-NMR (599 MHz, $CDCl_3$): δ 7.79 (s, 1H, OH), 6.39 (s, 1H), 5.22 (s, 2H), 2.11 (s, 3H), 1.03 (s, 9H), 0.26 (s, 6H);
^{13}C-NMR (151 MHz, $CDCl_3$): δ 173.0, 161.9, 155.8, 144.9, 116.1, 104.3, 104.0, 70.5, 25.8, 18.5, 8.6, -4.0;
HRMS (ESI-TOF): m/z calculated for $C_{15}H_{23}O_4Si^+$ [M+H] = 295.1360, found = 295.1375;
IR (neat, ATR): 3433, 2932, 2885, 2859, 1704, 1641, 1612, 1502, 1451, 1370, 1334, 1303, 1252, 1207, 1144, 1095, 1054, 988, 881 cm^{-1}.

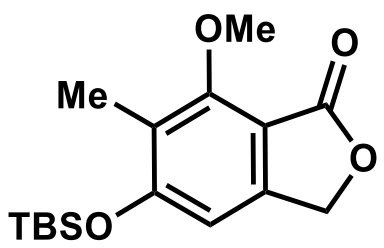

S31

5-((tert-butyldimethylsilyl)oxy)-7-methoxy-6-methylisobenzofuran-1(3H)-one (S31): In a 250 mL round bottom flask was placed 34 mL of tetrahydrofuran and 2.0 g of **S30** (6.8 mmol, 1.0 equiv). This solution was cooled with an ice bath, then 408 mg of NaH (60% w/w parafilm dispersion, 10.2 mmol, 1.50 equiv) was added portionwise, followed by 975 uL of dimethylsulfate (10.2 mmol, 1.5 equiv). The reaction mixture was allowed to warm up to room temperature and stirred for ~15 h. The reaction was quenched with sat. aq. ammonium chloride and then extracted with ethyl acetate three times. The combined organic layers were dried with $MgSO_4$, filtered, and concentrated under reduced pressure. The obtained crude mixture was purified by column chromatography (silica, 5–20% EtOAc/Hex) to afford the 510 mg of starting material and 753 mg of the title compound (48% yield based on recovered starting material).

TLC (20% EtOAc/hexanes): R_f = 0.4 (UV);
^{1}H-NMR (599 MHz, $CDCl_3$): 6.55 (s, 1H), 5.15 (s, 2H), 4.05 (s, 3H), 2.15 (s, 3H), 1.03 (s, 9H), 0.27 (s, 6H);
^{13}C-NMR (151 MHz, $CDCl_3$): δ 169.0, 161.1, 158.8, 147.4, 123.3, 110.2, 106.7, 68.7, 62.3, 25.8, 18.5, 9.6, -4.0;
HRMS (ESI-TOF): m/z calculated for $C_{16}H_{25}O_4Si^+$ [M+H] = 309.1517, found = 319.1516;
IR (neat, ATR): 2957, 2929, 2856, 1745, 1607, 1592, 1467, 1425, 1359, 1327, 1257, 1265, 1239, 1136, 1099, 1018, 881 cm^{-1}.

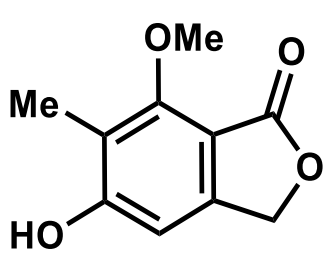

10

5-hydroxy-7-methoxy-6-methylisobenzofuran-1(3H)-one (10): In a 250 mL round bottom flask was placed 750 mg of **S31** (2.43 mmol, 1.00 equiv) and 15 mL of THF. The solution was cooled with an ice bath, then 4.9 mL of 1 M tetrabutylammonium fluoride (4.9 mmol, 2.0 equiv) was added and the mixture was allowed to warm to room temperature. After stirring for 1 h, the reaction was quenched by adding saturated aqueous ammonium chloride solution. This mixture was extracted with EtOAc three times, the combined organic layers were dried with $MgSO_4$, filtered, and concentrated under reduced pressure. The crude mixture was purified by column chromatography (silica, 40–60% EtOAc/Hex) to afford the title compound in 85% yield (400 mg). Spectroscopic data agreed with a previous report.[32]

^{1}H-NMR (599 MHz, $CDCl_3$): δ 6.60 (s, 1H), 5.16 (s, 2H), 4.08 (s, 3H), 2.19 (s, 3H).

Based on low solubility in $CDCl_3$, **10** was further characterized in d_6-acetone.

TLC (50% EtOAc/hexanes): R_f = 0.32 (UV);
^{1}H-NMR (599 MHz, d_6-acetone): δ 6.77 (s, 1H), 5.12 (s, 2H), 3.98 (s, 3H), 2.11 (s, 3H);
^{13}C-NMR (151 MHz, d_6-acetone): δ 169.3, 165.0, 159.1, 149.5, 118.9, 108.1, 104.3, 69.0, 61.9, 8.8;
HRMS (ESI-TOF): m/z calculated for $C_{10}H_{11}O_4^+$ [M+H] = 195.0652, found = 195.0656;
IR (neat, ATR): 3279 (br), 2931, 2846, 1718, 1601, 1496, 1457, 1426, 1337, 1279, 1237, 1132, 1090, 1010, 989, 942, 836 cm^{-1}.

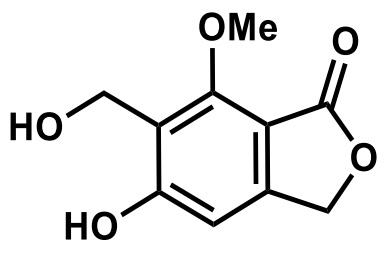

15

5-hydroxy-6-(hydroxymethyl)-7-methoxyisobenzofuran-1(3H)-one (15): In a 2 L culture flask was placed 168 mL of MiliQ-water, 10.5 mL of 1 M TES buffer (final concentration 50 mM, pH=7.5), 166 mg of sodium ascorbate (final concentration 4 mM), 123 mg of α-ketoglutaric acid (final concentration 4 mM) and 12 mg of $FeSO_4 \cdot 7H_2O$ (final concentration 0.2 mM). 80 mg of substrate **10** were dissolved in 10.5 mL of DMSO and added to the prepared mixture dropwise while shaking (final substrate concentration 1.8 mM). 21 mL of clarified cell lysate (500 mg/mL of NHI_C4 pellet in lysis buffer) was added, and the reaction mixture was placed in the incubator (100 rpm, 30 °C) for 1.5 h. Then, the same quantities of sodium ascorbate, α-ketoglutaric acid, $FeSO_4 \cdot 7H_2O$ and clarified cell lysate were added. The mixture was allowed to stir for another hour, then the aqueous mixture was extracted with EtOAc three times. Isopropanol was added when an emulsion was formed. The combined organic layers were washed 3 times with 1:1 brine/water and twice with brine. Subsequently, they were dried with Na_2SO_4, filtered, and concentrated under reduced pressure (*important*: avoid temperatures above 25 °C whenever the benzylic alcohol is concentrated). The obtained crude was purified by column chromatography (silica, 30-80% EtOAc/Hex) to yield the title compound as a white solid (31 mg, 36 % yield).

TLC (65% EtOAc/hexanes): R_f = 0.22 (UV);
^{1}H-NMR (599 MHz, d_6-acetone): δ 6.76 (s, 1H), 5.19 (s, 2H), 4.89 (s, 2H), 4.04 (s, 3H);
^{13}C-NMR (151 MHz, d_6-acetone): δ 169.6, 165.3, 159.4, 152.2, 121.2, 109.9, 106.1, 70.0, 63.7, 57.3;
HRMS (ESI-TOF): m/z calculated for $C_{10}H_9O_4^+$ [M+H-H_2O] = 193.0495, found=193.0504;
IR (neat, ATR): 3366 (br), 3192 (br), 2959, 2909, 2845, 1701, 1600, 1458, 1430, 1334, 1289, 1242, 1138, 1084, 984, 943, 856 cm^{-1}.

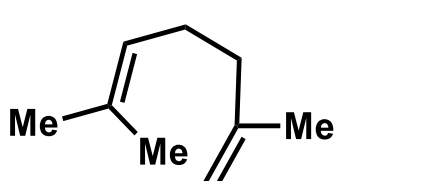

S32

(*E*)-6,10-dimethylundeca-5,9-dien-1-yne (S32): In a 250 mL round bottom flask was placed 70 mL of diethyl ether and 5.00 g of geraniol (32.4 mmol, 1.00 equiv). The mixture was placed in an ice bath, then 1.30 mL of PBr_3 (16.2 mmol, 0.500 equiv) was added. The reaction was allowed to stir for 30 min in the ice bath. Full conversion was confirmed by TLC (10% EtOAc/Hex). The reaction mixture was quenched by pouring into 60 mL of cold sat. aq. $NaHCO_3$. The mixture was allowed to shake for ~30 min and then was extracted with hexanes three times, the combined organic layers were washed with brine, dried with $MgSO_4$, filtered and concentrated under reduced pressure (*important*: avoid temperatures above 15 °C whenever the product is concentrated). The obtained oil, geranyl bromide, was used in the next step.

In a separate 500 mL round bottom flask was placed 130 mL of THF and 4.7 g of 1-(trimethylsilyl)propyne (42.1 mmol, 1.30 equiv). This mixture was cooled to -78 °C, then 19.4 mL of 2.5 M nBuLi solution (48.6 mmol, 1.5 equiv) was added. The mixture was taken to 0 °C with an ice bath and allowed to stir at that temperature for 1 h. Subsequently, the reaction mixture was cooled to -78 °C and geranyl bromide was added portionwise, the reaction was allowed to warm to room temperature and stirred for ~15 h. The reaction was quenched by adding sat. aq. NH4Cl, the mixture was extracted with hexanes three times and the combined organic layers were, dried with $MgSO_4$, filtered and concentrated under reduced pressure (important: avoid temperatures above 15 °C whenever the product is concentrated). The crude material was dissolved in 130 mL of MeOH, to this solution was added 22.0 g of K_2CO_3 (162 mmol, 5.00 equiv), and the final mixture was allowed to stir at room temperature for ~15 h. Methanol was evaporated under reduced pressure and the obtained solid was partitioned with water and hexanes. After extraction of the aqueous layer with hexanes three times, the combined organic layers were dried with $MgSO_4$, filtered, and concentrated under reduced pressure to yield the crude product. The crude product was purified by column chromatography (silica, 100% hexanes) to yield the title compound as a colorless oil (4.52 g, 79% yield). Spectroscopic data was in agreement with a previous report.[33]

^{1}H-NMR (400 MHz, $CDCl_3$): δ 5.18 (m, 1H), 5.10 (m, 1H), 2.21 (m, 4H), 2.07 (m, 2H), 2.00 (m, 2H), 1.94 (t, *J* = 2.4 Hz, 1H), 1.68 (s, 3H), 1.62 (s, 3H), 1.60 (s, 3H).

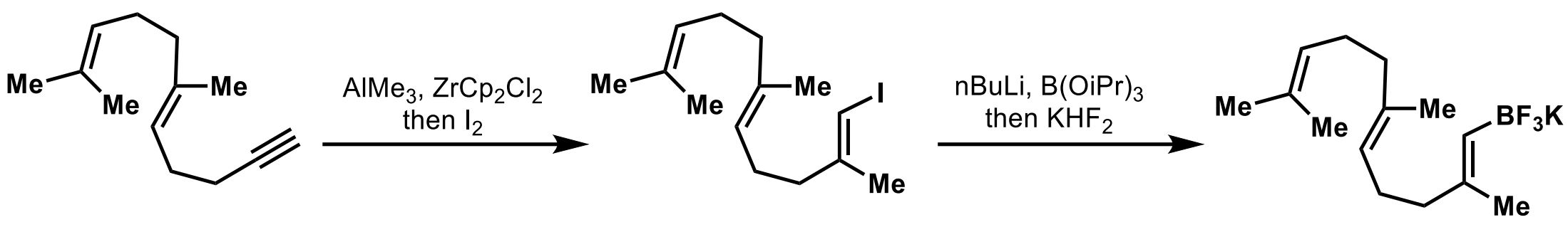

14% overall yield

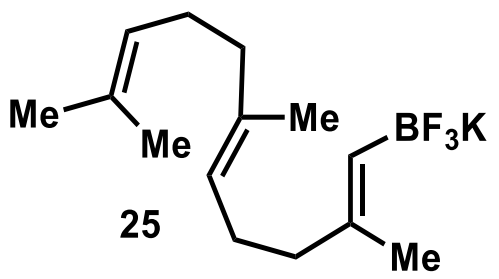

25

trifluoro((1*E*,5*E*)-2,6,10-trimethylundeca-1,5,9-trien-1-yl)-λ⁴-borane, potassium salt (25): In a 500 mL round bottom flask was placed 105 mL of dichloromethane and cooled in an ice bath. To the liquid was added 13.4 g of zirconocene dichloride (46.0 mmol, 1.10 equiv), and 46 mL of $AlMe_3$ solution (2 M in toluene, 92 mmol, 2.2 equiv). The mixture was allowed to stir for 15 min, then alkyne **S32** (7.37 g, 41.8 mmol, 1.00 equiv) was added dropwise. The reaction was allowed to reach room temperature and was left stirring overnight. 12.7 g of resublimed iodine (50.2 mmol, 1.20 equiv) was dissolved in 50 mL of THF and the solution was added dropwise to the reaction mixture under an ice bath. After 1 h, the reaction was quenched by addition of water, the mixture was extracted with dichloromethane and the combined organic layers were washed with a saturated aqueous solution of sodium thiosulfate. The crude obtained was flushed through a silica plug with hexanes to furnish 5.8 g of crude vinyl iodide (44% yield).

In a 250 mL round bottom flask, 4.49 g of crude vinyl iodide (14.1 mmol, 1 equiv) was dissolved in 50 mL of THF. The mixture was cooled down to -78 °C, 7.34 mL of nBuLi (2.5 M in hexanes, 18.3 mmol, 1.30 equiv) was added dropwise, and the mixture was allowed to stir at -78 °C for 1.5 h. Then, 4.23 mL of triisopropyl borate (18.3 mmol, 1.30 equiv) was added dropwise at -78 °C, then the mixture was placed in an ice bath and allowed to stir for 1 h. Subsequently, saturated aqueous NH_4Cl (~30mL) was added. After stirring at room temperature for 1 h, the mixture was extracted with diethyl ether. The combined organic layers were concentrated and the solvent was exchanged to methanol (*important: do not evaporate to dryness*). With a final volume of ~50 mL, this solution was placed in a pressure vessel and cooled in an ice bath. Subsequently, a solution of KHF_2 (3.86 g, 49.4 mmol, 3.50 equiv in 30 mL of water) was added, and the vessel was immediately closed. The reaction mixture was allowed to stir for 1.5 h in the ice bath, then it was frozen with liquid nitrogen and placed in the liophilizer for 2 days.

To the obtained white powder was added MeCN (about 300 mL) and the solution was filtered. The MeCN layer was washed with hexanes five times and evaporated to yield a crude BF_3K salt as a white solid (1.3 g, 31% yield), which was used in subsequent reactions. For characterization, the salt was dissolved in minimal amount of acetone, filtered and concentrated to yield a white solid.

^{1}H-NMR (599 MHz, acetonitrile-d_3): δ 5.16 (t, J=7.1 Hz, 1H), 5.13–5.09 (m, 1H), 5.02–4.97 (m, 1H), 2.09–2.03 (m, 4H), 2.00–1.89 (m, 4H, overlap with solvent signal), 1.69 (s, 3H), 1.66 (s, 3H), 1.61 (s, 3H), 1.60 (s, 3H);
^{13}C-NMR (151 MHz, acetonitrile-d_3): δ 135.2, 132.1, 126.2, 126.1, 125.3, 43.1, 40.5, 27.9, 27.5, 25.8, 19.7, 17.8, 16.0;
^{11}B-NMR (192 MHz, acetonitrile-d_3): δ 2.06 (s);
^{19}F-NMR (563 MHz, acetonitrile-d_3): δ -136.3 (m);
HRMS: Mass of the title compound was not found with different ionization types (ESI, EI, APCI);
IR (neat, ATR): 2967, 2915, 2855, 1639, 1447, 1378, 1341, 1170, 1103, 919, 840 cm^{-1}.

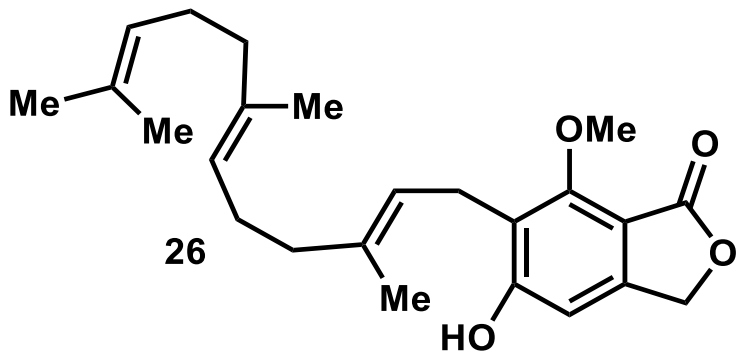

26

5-hydroxy-7-methoxy-6-((2*E*,6*E*)-3,7,11-trimethyldodeca-2,6,10-trien-1-yl)isobenzofuran-1(3H)-one (26): In a 1-dram vial was placed 0.5 mL of dichloromethane and 3 mg of benzylic alcohol **15** (28.5 µmol, 1.00 equiv). The vial was placed in an ice bath, followed by addition of pyridine (2.50 µL, 31.4 µmol, 1.10 equiv), and acetyl chloride (2.20 µL, 31.4 µmol, 1.10 equiv). The mixture was warmed to room temperature and allowed to stir for 2 h, quenched by addition of sat. aq. NH_4Cl and extracted with dichloromethane three times. The reaction was quenched even though not full conversion was observed by TLC 65% EtOAc/Hex. The combined organic layers were washed three times with sat. aq. $CuSO_4$, dried with $MgSO_4$, decanted and concentrated under reduced pressure. The obtained crude was dissolved in 0.5 mL of acetonitrile, to the obtained solution was added 21 mg of salt **25** (71.4 µmol, 5.00 equiv) and 17 mg of KHF_2 (0.21 mmol, 15 equiv). The reaction mixture was heated at 80 °C for 2 h, then it was cooled down to room temperature, and quenched by addition of brine. Subsequently, the mixture was extracted with EtOAc three times, dried with $MgSO_4$, filtered and concentrated under reduced pressure. The obtained crude was purified by column chromatography (silica, 40–60% ether/hexanes) to yield the title compound as a white solid in 47% yield (2.6 mg).

TLC (60% diethyl ether/hexanes): R_f = 0.20 (UV);
^{1}H-NMR (599 MHz, $CDCl_3$): δ 6.62 (s, 1H), 6.05 (s, 1H, OH), 5.18 (t, *J* = 7.2 Hz, 1H), 5.15 (s, 2H), 5.10 – 5.03 (m, 2H), 4.06 (s, 3H), 3.50 (d, *J* = 7.2 Hz, 2H), 2.10 (m, 4H), 2.03 (m, 2H), 1.96 (m, 2H), 1.84 (s, 3H), 1.67 (s, 3H), 1.59 (s, 6H);
^{13}C-NMR (151 MHz, $CDCl_3$): δ 169.0, 162.0, 158.3, 148.6, 140.2, 136.0, 131.5, 124.5, 123.5, 120.9, 120.8, 109.8, 104.6, 68.7, 63.0, 39.8, 39.8, 26.8, 26.4, 25.8, 22.6, 17.8, 16.5, 16.2;
HRMS (ESI-TOF): m/z calculated for $C_{24}H_{33}O_4^+$ [M+H] = 385.2373, found = 385.2372;
IR (neat, ATR): 3263 (br), 2920, 2848, 1710, 1693, 1600, 1528, 1459, 1432, 1337, 1286, 1234, 1137, 1081, 1016 cm^{-1}.

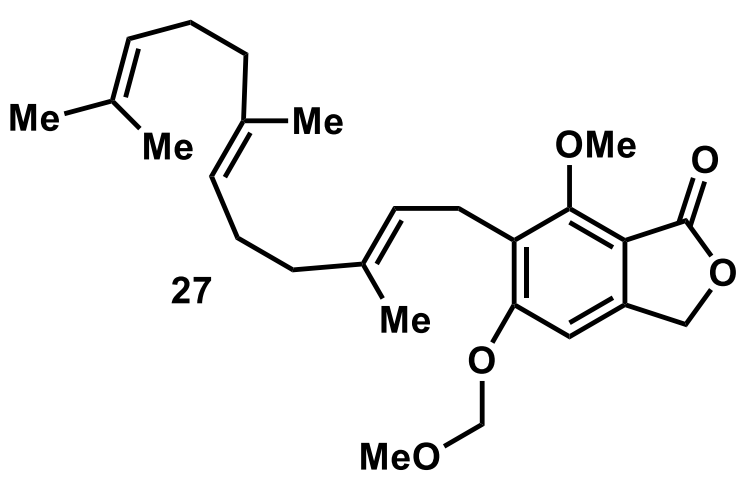

27

7-methoxy-5-(methoxymethoxy)-6-((2*E*,6*E*)-3,7,11-trimethyldodeca-2,6,10-trien-1-yl)isobenzofuran-1(3H)-one (27): In a 1 dram vial was placed 2.6 mg of phenol **26** (6.8 µmol, 1.0 equiv) with 0.5 mL of dichloromethane. Then, 1.7 µL of diisopropylethylamine (10 µmol, 1.5 equiv) and 770 µL of chloro(methoxy)methane (10.1 µmol, 1.50 equiv) were added. The mixture was allowed to warm up to room temperature and stirred for 2 h, then it was quenched by addition of sat. aq. NH_4Cl and subsequently extracted with dichloromethane three times. The combined organic layers were dried with $MgSO_4$, filtered, and concentrated under reduced pressure. The obtained crude mixture was purified by column chromatography (silica, 40-50% ether/hexanes) to yield the title compound as a colorless oil in 69% yield (2.0 mg). The spectroscopic data agreed with a previously reported intermediate in the synthesis of (17*S*)-dihydroaustalide K.[34]

TLC (60% diethyl ether/hexanes): R_f = 0.52 (UV);
^{1}H-NMR (599 MHz, $CDCl_3$): δ 6.88 (s, 1H), 5.27 (s, 2H), 5.17 (s, 2H), 5.14 (t, *J* = 7.4 Hz, 1H), 5.06 (t, *J* = 7.1 Hz, 2H), 4.07 (s, 3H), 3.48 (s, 3H), 3.42 (d, *J* = 7.2 Hz, 2H), 2.09–1.88 (m, 8H), 1.79 (s, 3H), 1.67 (s, 3H), 1.58 (s, 3H), 1.56 (s, 3H);
^{13}C-NMR (151 MHz, $CDCl_3$): δ 169.0, 161.5, 158.2, 148.3, 135.7, 135.2, 131.5, 125.0, 124.5, 124.2, 122.2, 110.7, 102.1, 94.4, 69.0, 62.8, 56.5, 39.9, 39.8, 26.9, 26.7, 25.8, 22.9, 17.8, 16.3, 16.2;
HRMS (ESI-TOF): m/z calculated for $C_{26}H_{37}O_5^+$ [M+H] = 429.2636, found = 429.2637;
IR (neat, ATR): 2920, 2849, 1756, 1692, 1605, 1528, 1465, 1430, 1401, 1361, 1328, 1235, 1151, 1118, 1080, 1042, 979, 943, 926, 833 cm^{-1}.

^{1}H-NMR

This work	Previous synthesis[34]	Difference (ppm)
6.88 (s, 1H)	6.88 (s, 1H)	0
5.27 (s, 2H)	5.27 (s, 2H)	0
5.17 (s, 2H)	5.17(s, 2H)	0
5.14 (t, J=7.4 Hz, 1H)	5.16−5.10 (m, 1H)	-
5.06 (t, J=7.1 Hz, 2H)	5.09−5.02 (m, 2H)	-
4.07 (s, 3H)	4.06 (s, 3H)	-0.01
3.48 (s, 3H)	3.48(s, 3H)	0
3.42 (d, J=7.2 Hz, 2H)	3.42 (d, J = 7.1 Hz, 2H)	0
2.09–1.88 (m, 8H)	2.09−1.88 (m, 8H)	-
1.79 (s, 3H)	1.79 (s, 3H)	0
1.67 (s, 3H)	1.66 (s, 3H)	-0.01
1.58 (s, 3H)	1.58 (s, 3H)	0
1.56 (s, 3H)	1.56 (s, 3H)	0

^{13}C-NMR

This work	Previous synthesis[34]	Difference (ppm)
169.0	168.8	-0.2
161.5	161.3	-0.2
158.2	158.0	-0.2
148.3	148.2	-0.1
135.7	135.5	-0.2
135.2	135.0	-0.2
131.5	131.3	-0.2
125.0	124.8	-0.2
124.5	124.3	-0.2
124.2	124.0	-0.2
122.2	122.0	-0.2
110.7	110.5	-0.2
102.1	101.9	-0.2
94.4	94.2	-0.2
69.0	68.8	-0.2
62.8	62.6	-0.2
56.5	56.3	-0.2
39.9	39.8	-0.1
39.8	39.7	-0.1
26.9	26.7	-0.2
26.7	26.5	-0.2
25.8	25.7	-0.1
22.9	22.7	-0.2
17.8	17.6	-0.2
16.3	16.1	-0.2
16.2	16.0	-0.2

10. X-ray data

X-ray data of chaetosemin B (**2**, revised)

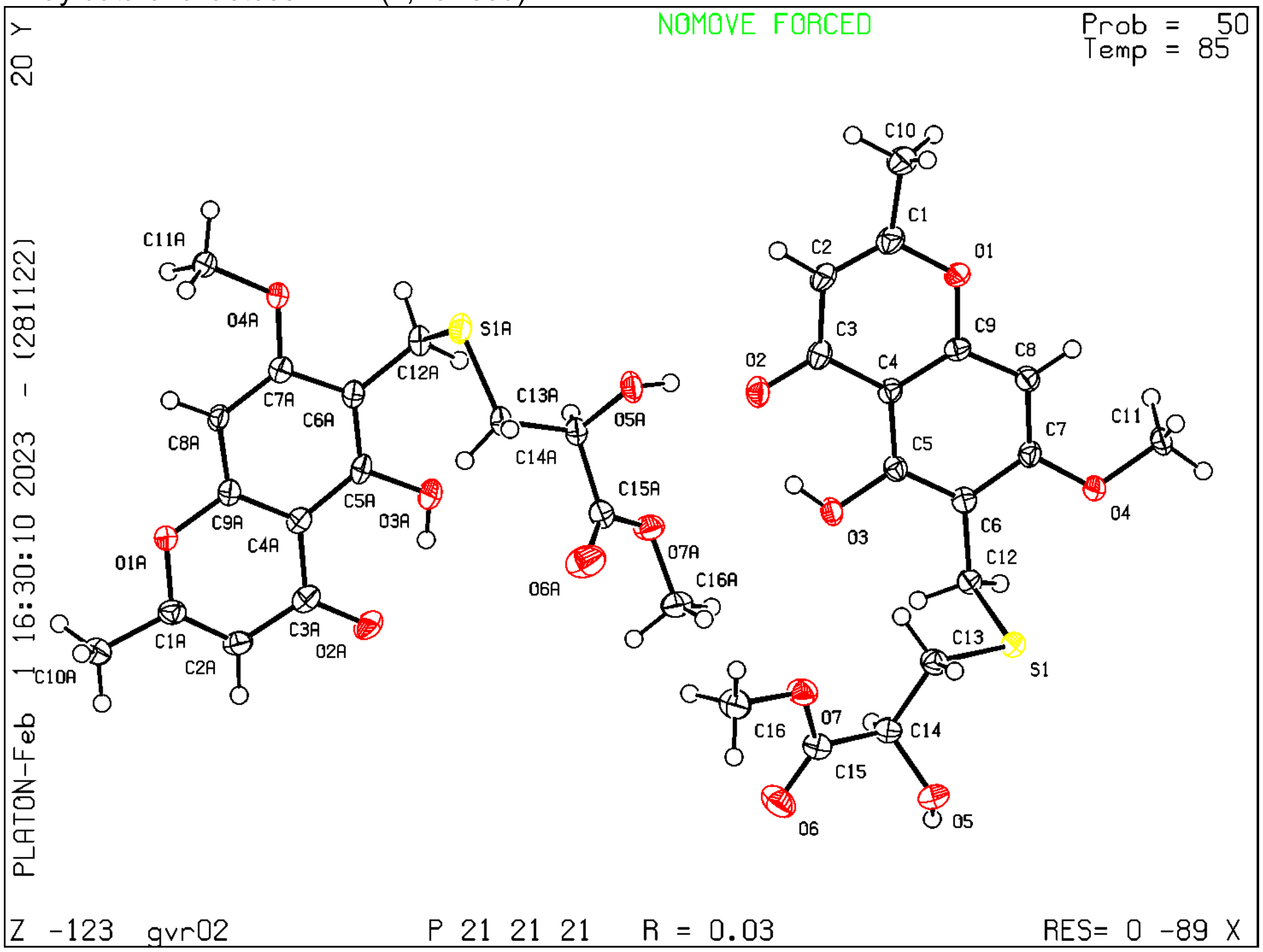

Chaetosemin B (**2**, revised) was recrystallized by adding hexanes to a solution of chloroform. We confirmed the enantiopurity of our samples was maintained. Then we grew crystals by slow diffusion of hexanes to a chloroform solution of the sample. A suitable crystal with dimensions 0.12 × 0.08 × 0.06 mm^3 was selected and mounted on a dtrek-CrysAlisPro-abstract goniometer imported rigaku-d*trek images diffractometer. The crystal was kept at a steady T = 85 K during data collection. The structure was solved with the ShelXT 2018/2[35] solution program using dual methods and by using Olex2 1.5-alpha[36] as the graphical interface. The model was refined with ShelXL 2018/3[35] using full matrix least squares minimization on $\boldsymbol{F}^2$.

X-ray data

Formula	$C_{16}H_{18}O_7S$
$D_{calc.}$/ g cm^{-3}	1.467
m/mm^{-1}	2.130
Formula Weight	354.36
Colour	colourless
Shape	block-shaped
Size/mm^3	0.12×0.08×0.06
T/K	85
Crystal System	orthorhombic
Flack Parameter	-0.033(7)
Hooft Parameter	-0.047(5)
Space Group	$P2_12_12_1$
a/Å	10.23290(10)
b/Å	13.41900(10)
c/Å	23.37570(10)
a/°	90
b/°	90
g/°	90
V/Å^3	3209.84(4)
Z	8
Z'	2
Wavelength/Å	1.54184
Radiation type	Cu K_a
Q_{min}/°	3.782
Q_{max}/°	69.528
Measured Refl's.	47496
Indep't Refl's	5991
Refl's I≥2 *s*(I)	5890
R_{int}	0.0676
Parameters	444
Restraints	0
Largest Peak	0.345
Deepest Hole	-0.244
GooF	1.059
wR_2 (all data)	0.0869
wR_2	0.0857
R_1 (all data)	0.0334
R_1	0.0328

11. References

[1] J. A. Gerlt, “Genomic Enzymology: Web Tools for Leveraging Protein Family Sequence–Function Space and Genome Context to Discover Novel Functions” *Biochemistry* **2017**, *56*, 4293–4308.

[2] J. A. Gerlt, J. T. Bouvier, D. B. Davidson, H. J. Imker, B. Sadkhin, D. R. Slater, K. L. Whalen, “Enzyme Function Initiative-Enzyme Similarity Tool (EFI-EST): A web tool for generating protein sequence similarity networks” *Biochim. Biophys. Acta BBA - Proteins Proteomics* **2015**, *1854*, 1019–1037.

[3] Y. He, R. J. Cox, “The molecular steps of citrinin biosynthesis in fungi” *Chem. Sci.* **2016**, *7*, 2119–2127.

[4] J. Fan, G. Liao, F. Kindinger, L. Ludwig-Radtke, W.-B. Yin, S.-M. Li, “Peniphenone and Penilactone Formation in *Penicillium crustosum* via 1,4-Michael Additions of *ortho* -Quinone Methide from Hydroxyclavatol to γ-Butyrolactones from Crustosic Acid” *J. Am. Chem. Soc.* **2019**, *141*, 4225–4229.

[5] J. Davison, A. Al Fahad, M. Cai, Z. Song, S. Y. Yehia, C. M. Lazarus, A. M. Bailey, T. J. Simpson, R. J. Cox, “Genetic, molecular, and biochemical basis of fungal tropolone biosynthesis” *Proc. Natl. Acad. Sci.* **2012**, *109*, 7642–7647.

[6] R. Schor, C. Schotte, D. Wibberg, J. Kalinowski, R. J. Cox, “Three previously unrecognised classes of biosynthetic enzymes revealed during the production of xenovulene A” *Nat. Commun.* **2018**, *9*, 1963.

[7] V. Vece, S. Jakkepally, S. Hanessian, “Total Synthesis and Absolute Stereochemical Assignment of the Insecticidal Metabolites Yaequinolones J1 and J2” *Org. Lett.* **2018**, *20*, 4277–4280.

[8] E. Baciocchi, L. Mandolini, C. Rol, “Oxidation by metal ions. 6. Intramolecular selectivity in the side-chain oxidation of p-ethyltoluene and isodurene by cobalt(III), cerium(IV), and manganese(III)” *J. Org. Chem.* **1980**, *45*, 3906–3909.

[9] A. D. Cort, L. Mandolini, S. Panaioli, “Selective One-Pot Oxidation of Methylarenes to Benzyl Alcohols with the Copper(II)-Peroxydisulfate System” *Synth. Commun.* **1988**, *18*, 613–616.

[10] L. Li, Y. Liu, Q. Wang, “Regioselective Oxidative Dehydrogenation under Nonenzymatic Conditions: A Synthetic Route to Gossypol” *Eur. J. Org. Chem.* **2013**, *2013*, 8014–8021.

[11] T. J. Doyon, J. C. Perkins, S. A. Baker Dockrey, E. O. Romero, K. C. Skinner, P. M. Zimmerman, A. R. H. Narayan, “Chemoenzymatic *o*-Quinone Methide Formation” *J. Am. Chem. Soc.* **2019**, *141*, 20269–20277.

[12] S. A. Baker Dockrey, A. L. Lukowski, M. R. Becker, A. R. H. Narayan, “Biocatalytic site- and enantioselective oxidative dearomatization of phenols” *Nat. Chem.* **2018**, *10*, 119–125.

[13] C. F. Carvalho, M. V. Sargent, “Naturally occurring dibenzofurans. Part 4. Synthesis of dibenzofurandiols by annelation of benzofurans” *J. Chem. Soc. Perkin 1* **1984**, 1605.

[14] H. Zhang, E. M. Ferreira, B. M. Stoltz, “Direct Oxidative Heck Cyclizations: Intramolecular Fujiwara–Moritani Arylations for the Synthesis of Functionalized Benzofurans and Dihydrobenzofurans” *Angew. Chem. Int. Ed.* **2004**, *43*, 6144–6148.

[15] A. Arnone, G. Nasini, O. Vajna De Pava, L. Merlini, “Constituents of Dragon’s Blood. 5. Dracoflavans B_1 , B_2 , C_1 , C_2 , D_1 , and D_2 , New A-Type Deoxyproanthocyanidins” *J. Nat. Prod.* **1997**, *60*, 971–975.

[16] W. Lee, D. Lee, Y. Lee, T. Lee, K.-S. Song, E.-J. Yang, J.-S. Bae, “Isolation, Synthesis, and Antisepsis Effects of a *C* -Methylcoumarinochromone Isolated from *Abronia nana* Cell Culture” *J. Nat. Prod.* **2018**, *81*, 1173–1182.

[17] Y. Feng, J. W. Blunt, A. L. J. Cole, M. H. G. Munro, “The Isolation of Two New Chromone Derivatives from the New Zealand Fungus *Tolypocladium e xtinguens*” *J. Nat. Prod.* **2002**, *65*, 1681–1682.

[18] S. J. Mear, T. F. Jamison, “Diazotization of *S* -Sulfonyl-cysteines” *J. Org. Chem.* **2019**, *84*, 15001–15007.

[19] K. Shimada, K. Mitamura, “Derivatization of thiol-containing compounds” *J. Chromatogr. B. Biomed. Sci. App.* **1994**, *659*, 227–241.

[20] S. Vichi, N. Cortés-Francisco, J. Caixach, “Determination of volatile thiols in lipid matrix by simultaneous derivatization/extraction and liquid chromatography–high resolution mass spectrometric analysis. Application to virgin olive oil” *J. Chromatogr. A* **2013**, *1318*, 180–188.

[21] B. Ferko, M. Zeman, M. Formica, S. Veselý, J. Doháňošová, J. Moncol, P. Olejníková, D. Berkeš, P. Jakubec, D. J. Dixon, O. Caletková, “Total Synthesis of Berkeleylactone A” *J. Org. Chem.* **2019**, *84*, 7159–7165.

[22] H. Li, J.-M. Tian, H.-Y. Tang, S.-Y. Pan, A.-L. Zhang, J.-M. Gao, “Chaetosemins A–E, new chromones isolated from an Ascomycete Chaetomium seminudum and their biological activities” *RSC Adv.* **2015**, *5*, 29185–29192.

[23] C.-P. Yu, Y. Tang, L. Cha, S. Milikisiyants, T. I. Smirnova, A. I. Smirnov, Y. Guo, W. Chang, “Elucidating the Reaction Pathway of Decarboxylation-Assisted Olefination Catalyzed by a Mononuclear Non-Heme Iron Enzyme” *J. Am. Chem. Soc.* **2018**, *140*, 15190–15193.

[24] H. Fujimoto, M. Nozawa, E. Okuyama, M. Ishibashi, “Five New Chromones Possessing Monoamine Oxidase Inhibitory Activity from an Ascomycete, Chaetomium quadrangulatum” *Chem. Pharm. Bull.* **2002**, *50*, 330–336.

[25] D. C. McKinney, F. Zhou, C. J. Eyermann, A. D. Ferguson, D. B. Prince, J. Breen, R. A. Giacobbe, S. Lahiri, J. C. Verheijen, “4,5-Disubstituted 6-Aryloxy-1,3-dihydrobenzo[*c*][1,2]oxaboroles Are Broad-Spectrum Serine β-Lactamase Inhibitors” *ACS Infect. Dis.* **2015**, *1*, 310–316.

[26] E. O. Romero, J. C. Perkins, J. E. Burch, D. A. Delgadillo, H. M. Nelson, A. R. H. Narayan, “Chemoenzymatic Synthesis of (+)-Xyloketal B” *Org. Lett.* **2023**, *25*, 1547–1552.

[27] X.-N. Sang, S.-F. Chen, G. Chen, X. An, S.-G. Li, X.-N. Li, B. Lin, J. Bai, H.-F. Wang, Y.-H. Pei, “Phomeketales A–F, six unique metabolites from the endophytic fungus Phoma sp. YN02-P-3” *RSC Adv.* **2016**, *6*, 64890–64894.
[28] Z. Powers, A. Scharf, A. Cheng, F. Yang, M. Himmelbauer, T. Mitsuhashi, L. Barra, Y. Taniguchi, T. Kikuchi, M. Fujita, I. Abe, J. A. Porco, “Biomimetic Synthesis of Meroterpenoids by Dearomatization-Driven Polycyclization” *Angew. Chem. Int. Ed.* **2019**, *58*, 16141–16146.
[29] J. W. Lockner, Studies in Meroterpenoid Synthesis (Doctoral dissertation), The Scripps Research Institute, **2011**. https://baranlab.org/wp-content/uploads/2020/11/Lockner-Thesis.pdf
[30] J. W. Patterson, “The synthesis of mycophenolic acid” *Tetrahedron* **1993**, *49*, 4789–4798.
[31] M. V. Sargent, “Naturally Occurring Dibenzofurans. Part 9.’ A Convenient Synthesis of Phthalides: The Synthesis of Methyl Di-O-methylporphyrilate” *J. Chem. Soc., Perkin Trans.* **1987**, *16*, 231–235.
[32] M. Fujita, M. Yamada, S. Nakajima, K.-I. Kawai, M. Nagai, “*O*-Methylation effect on the Carbon-13 nucelar magnetic resonance signald of *ortho*-disubstituted phenols and its application to structure determination of new phthalides from *Aspergillus silvaticus*” *Chem. Pharm. Bull.* **1984**, *32*, 2622–2627.
[33] B. H. Lipshutz, G. Bulow, F. Fernandez-Lazaro, S.-K. Kim, R. Lowe, P. Mollard, K. L. Stevens, “A Convergent Approach to Coenzyme Q” *J. Am. Chem. Soc.* **1999**, *121*, 11664–11673.
[34] T.-K. Ma, P. J. Parsons, A. G. M. Barrett, “Meroterpenoid Synthesis via Sequential Polyketide Aromatization and Radical Anion Cascade Triene Cyclization: Biomimetic Total Syntheses of Austalide Natural Products” *J. Org. Chem.* **2019**, *84*, 4961–4970.
[35] G. M. Sheldrick, “*SHELXT* – Integrated space-group and crystal-structure determination” *Acta Crystallogr. Sect. Found. Adv.* **2015**, *71*, 3–8.
[36] O. V. Dolomanov, L. J. Bourhis, R. J. Gildea, J. A. K. Howard, H. Puschmann, “*OLEX2* : a complete structure solution, refinement and analysis program” *J. Appl. Crystallogr.* **2009**, *42*, 339–341.

12. ^1H and ^{13}C NMR spectra

^1H-NMR (599 MHz, MeOD) of 3,5-dimethoxy-4-methylphenol (S9):

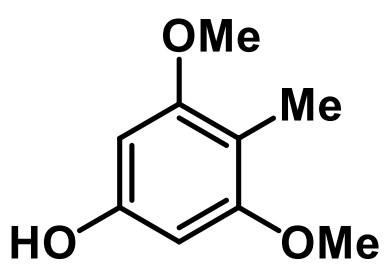

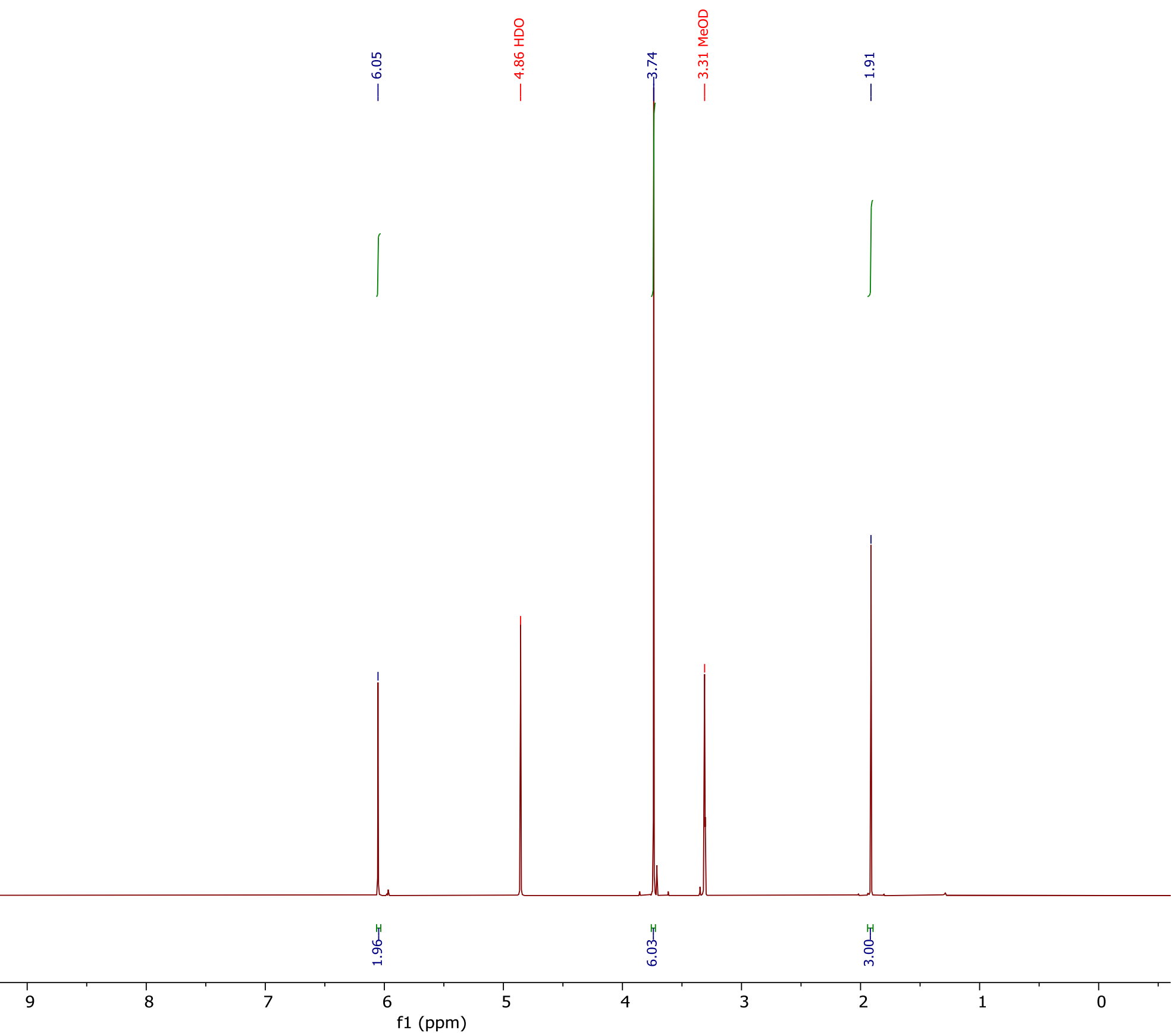

^{13}C-NMR (151 MHz, d_4-MeOH) of 3,5-dimethoxy-4-methylphenol (S9):

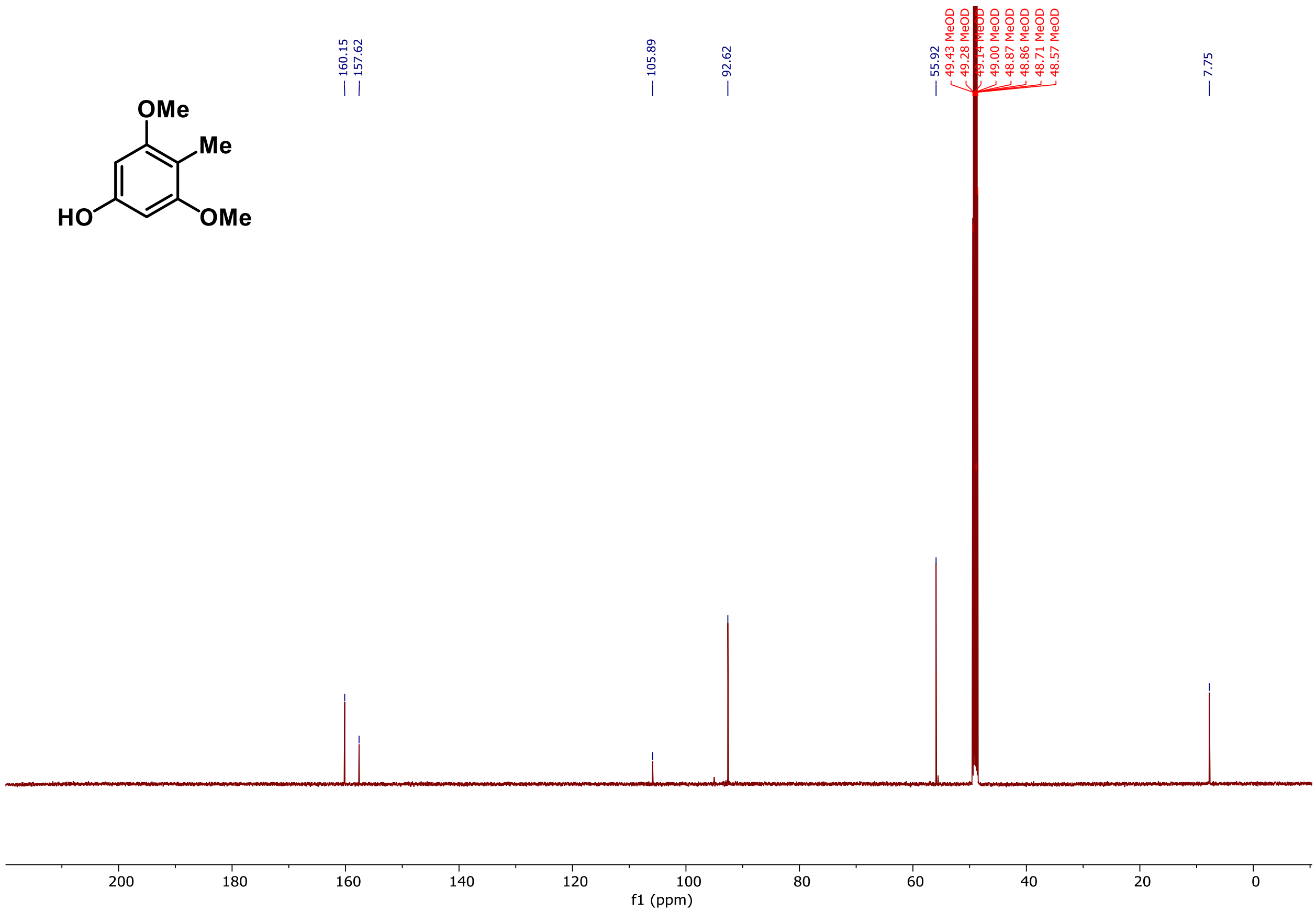

^{1}H-NMR (599 MHz, $CDCl_3$) of 2-hydroxy-4,6-dimethoxy-5-methylacetophenone (S10):

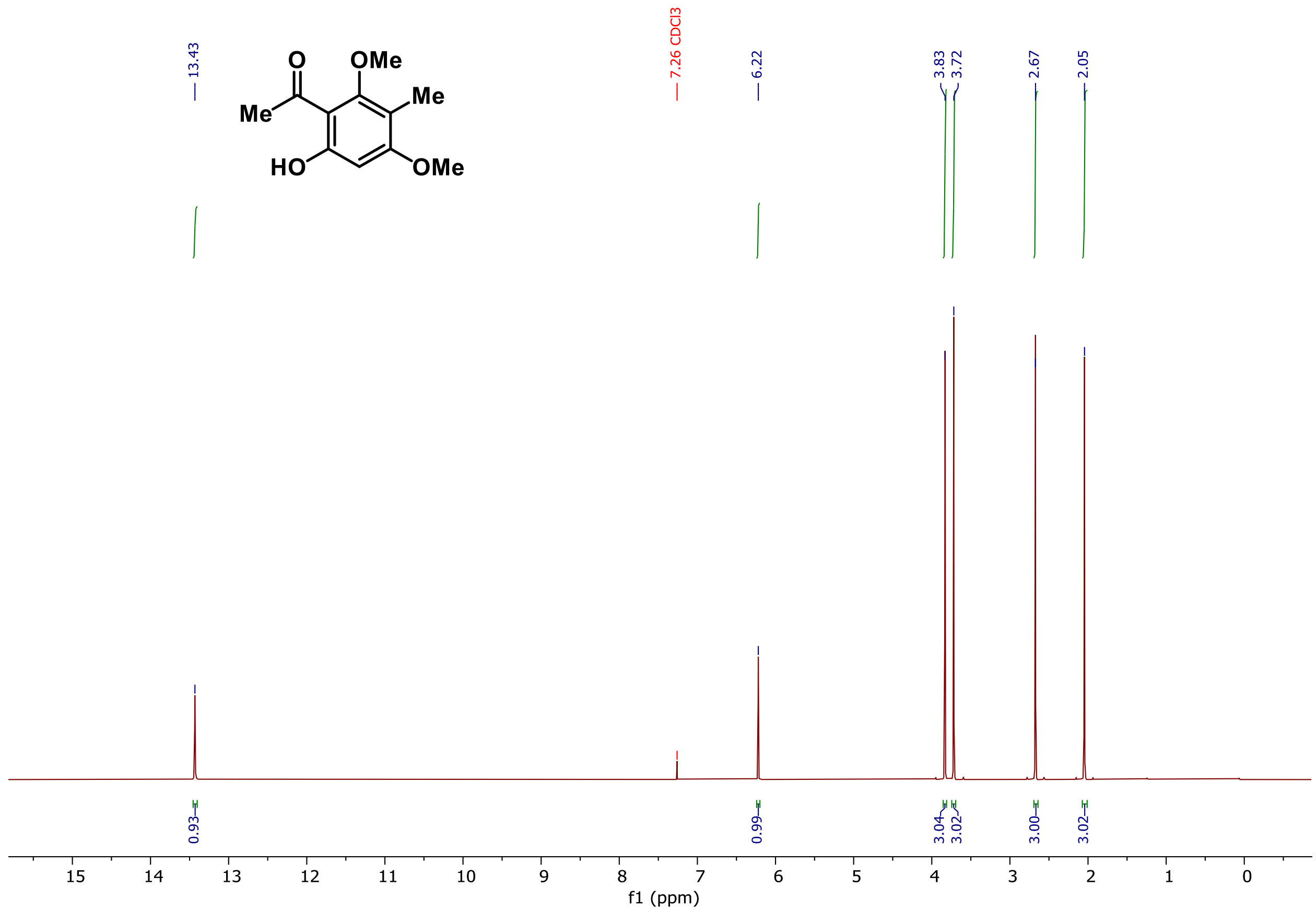

^{13}C-NMR (151 MHz, $CDCl_3$) of 2-hydroxy-4,6-dimethoxy-5-methylacetophenone (S10):

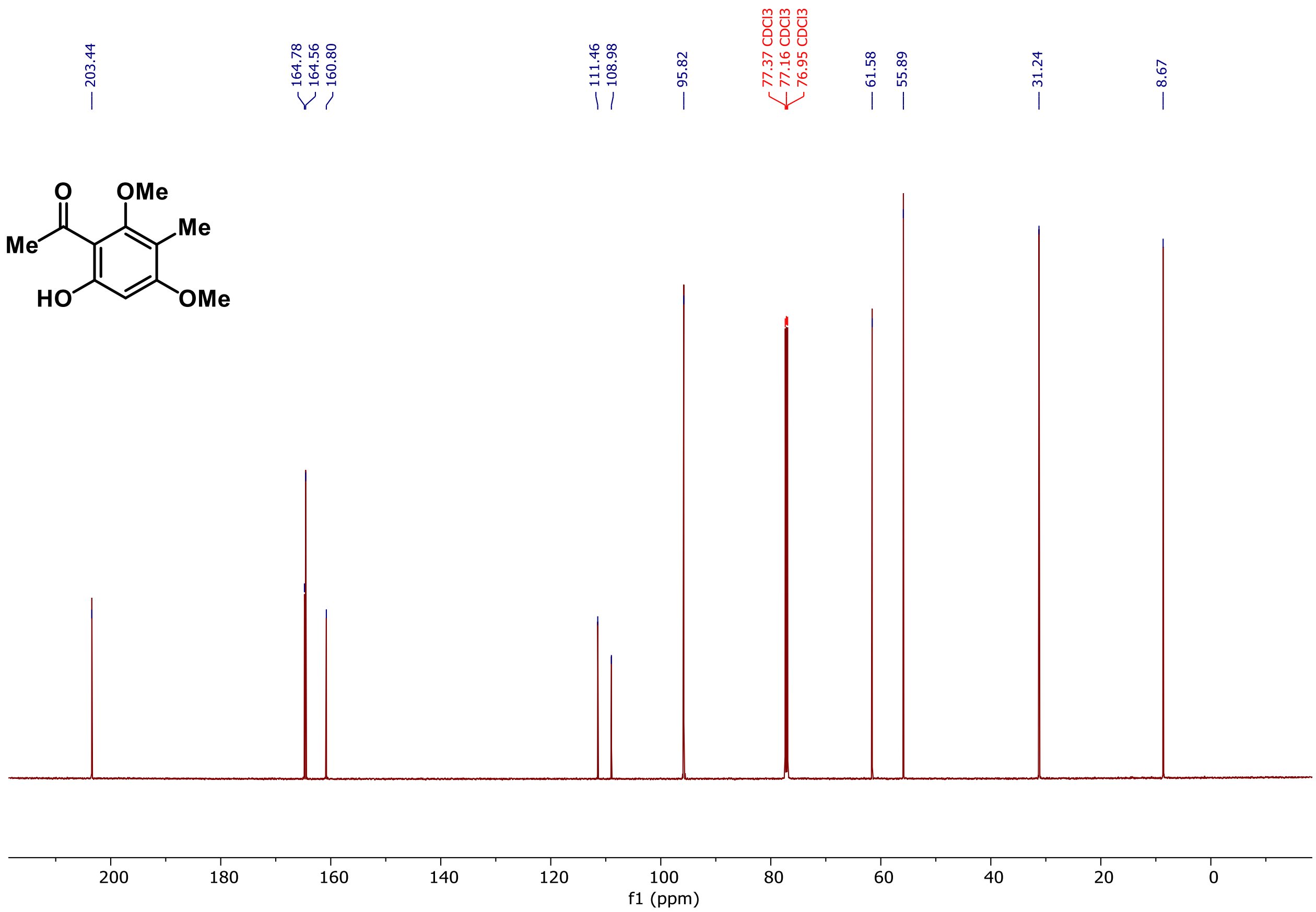

^{1}H-NMR (599 MHz, $CDCl_3$) of 5,7-dimethoxy-2,6-dimethyl-4H-chromen-4-one (S11)

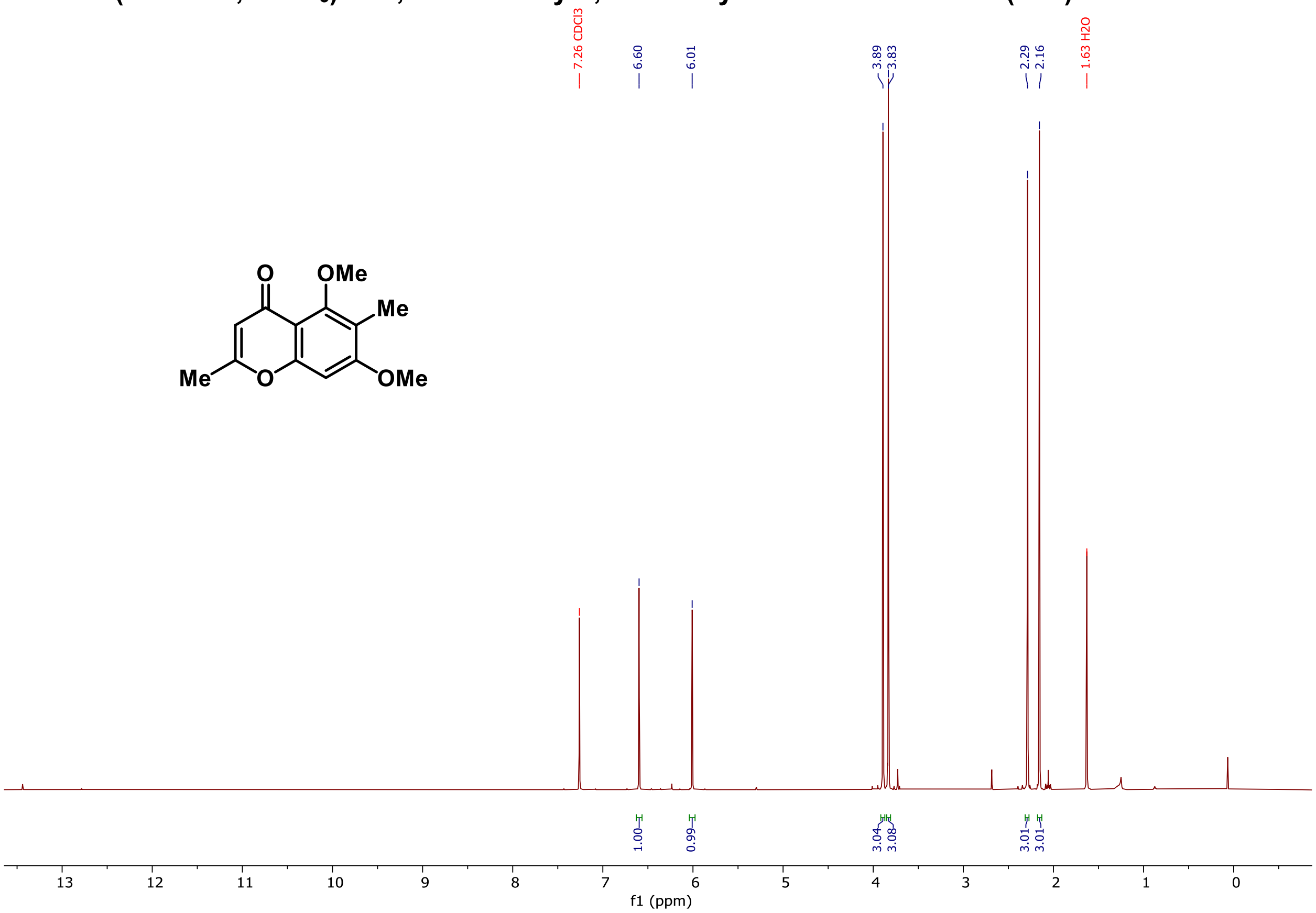

^{13}C-NMR (151 MHz, $CDCl_3$) of 5,7-dimethoxy-2,6-dimethyl-4H-chromen-4-one (S11)

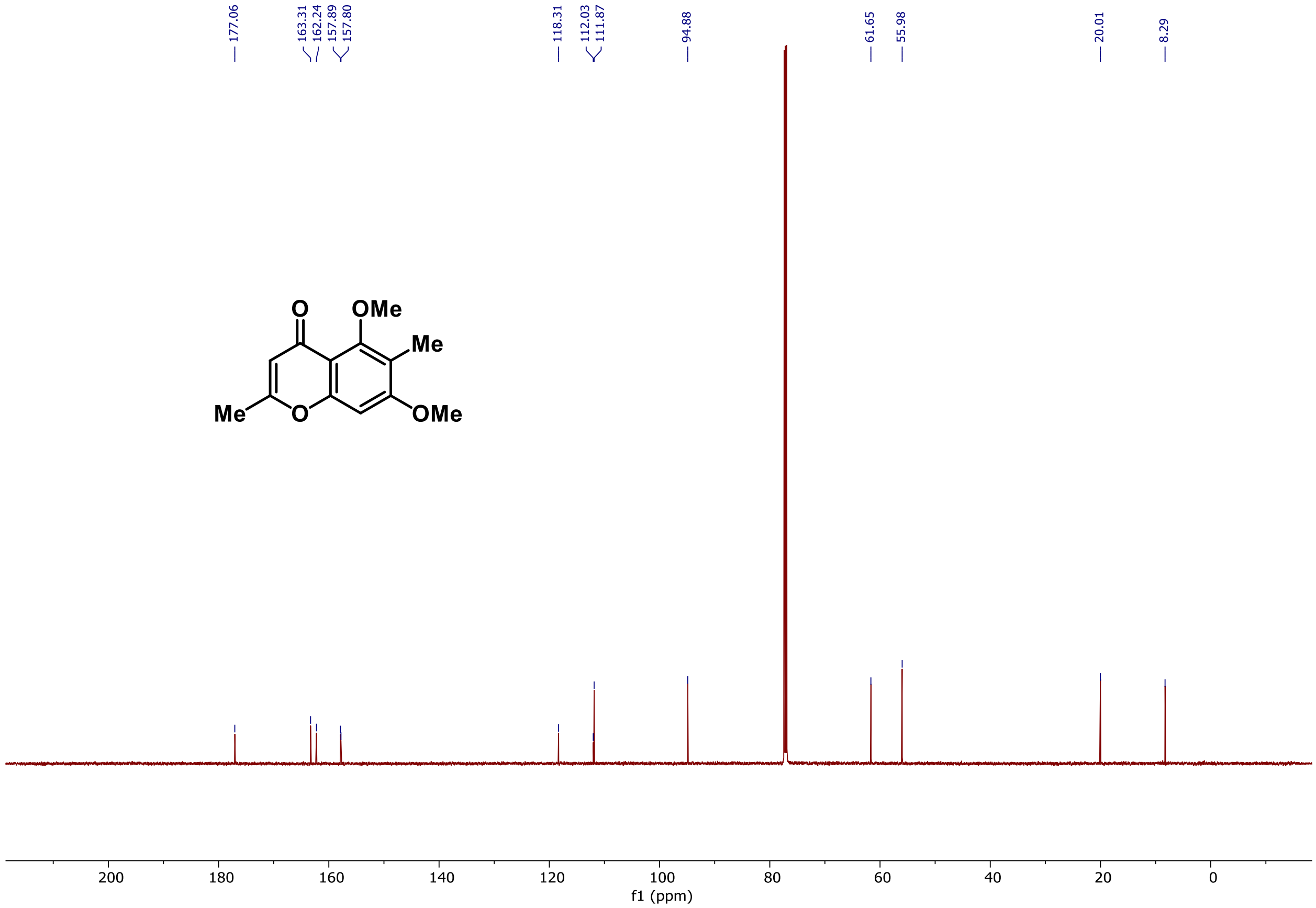

^{1}H-NMR (599 MHz, $CDCl_3$) of 5-hydroxy-7-methoxy-2,6-dimethyl-4H-chromen-4-one (7):

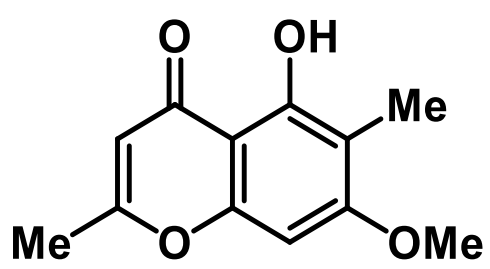

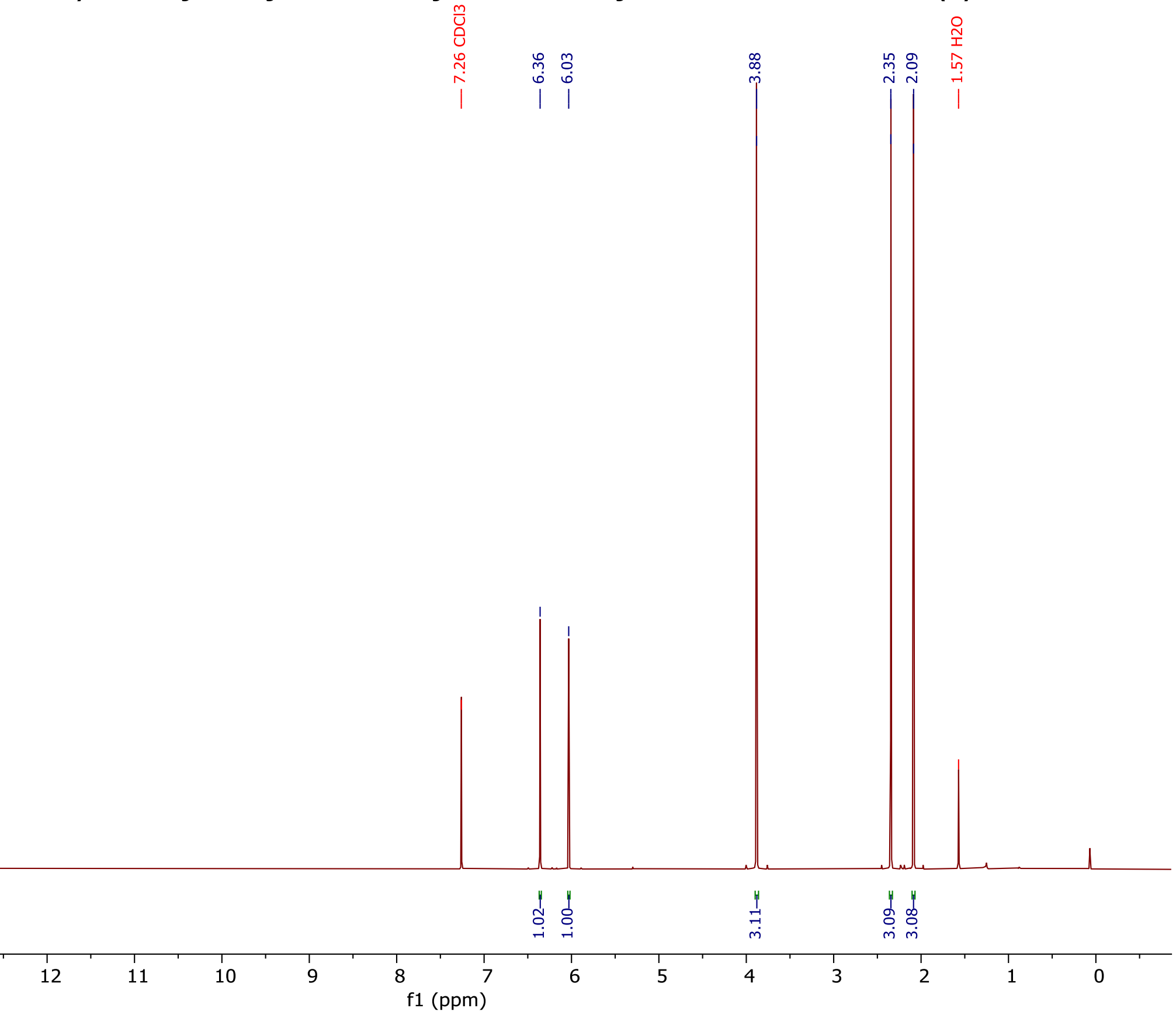

^{13}C-NMR (151 MHz, $CDCl_3$) of 5-hydroxy-7-methoxy-2,6-dimethyl-4H-chromen-4-one (7):

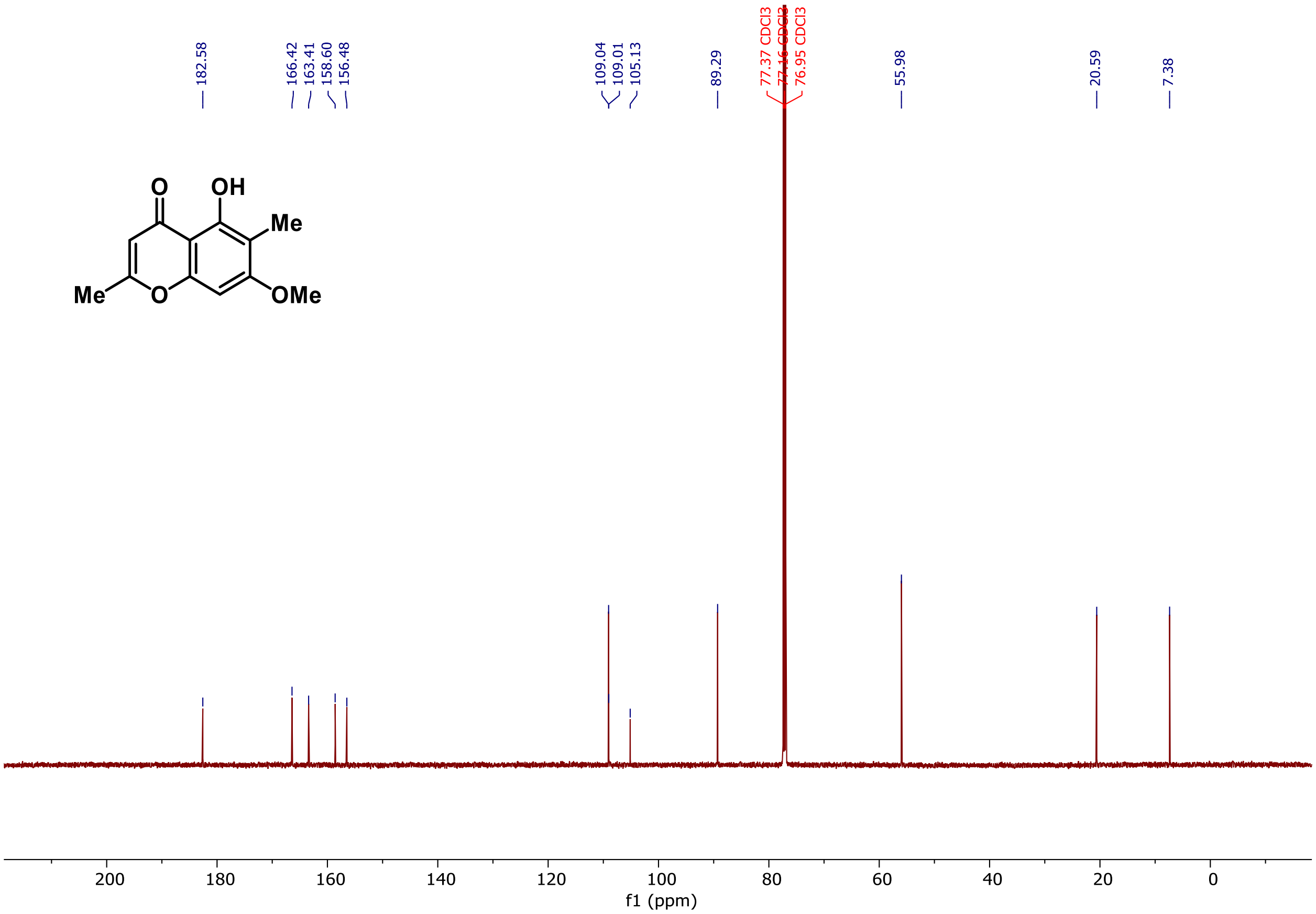

^{1}H-NMR (599 MHz, $CDCl_3$) of 6-Hydroxymethyleugenin (12):

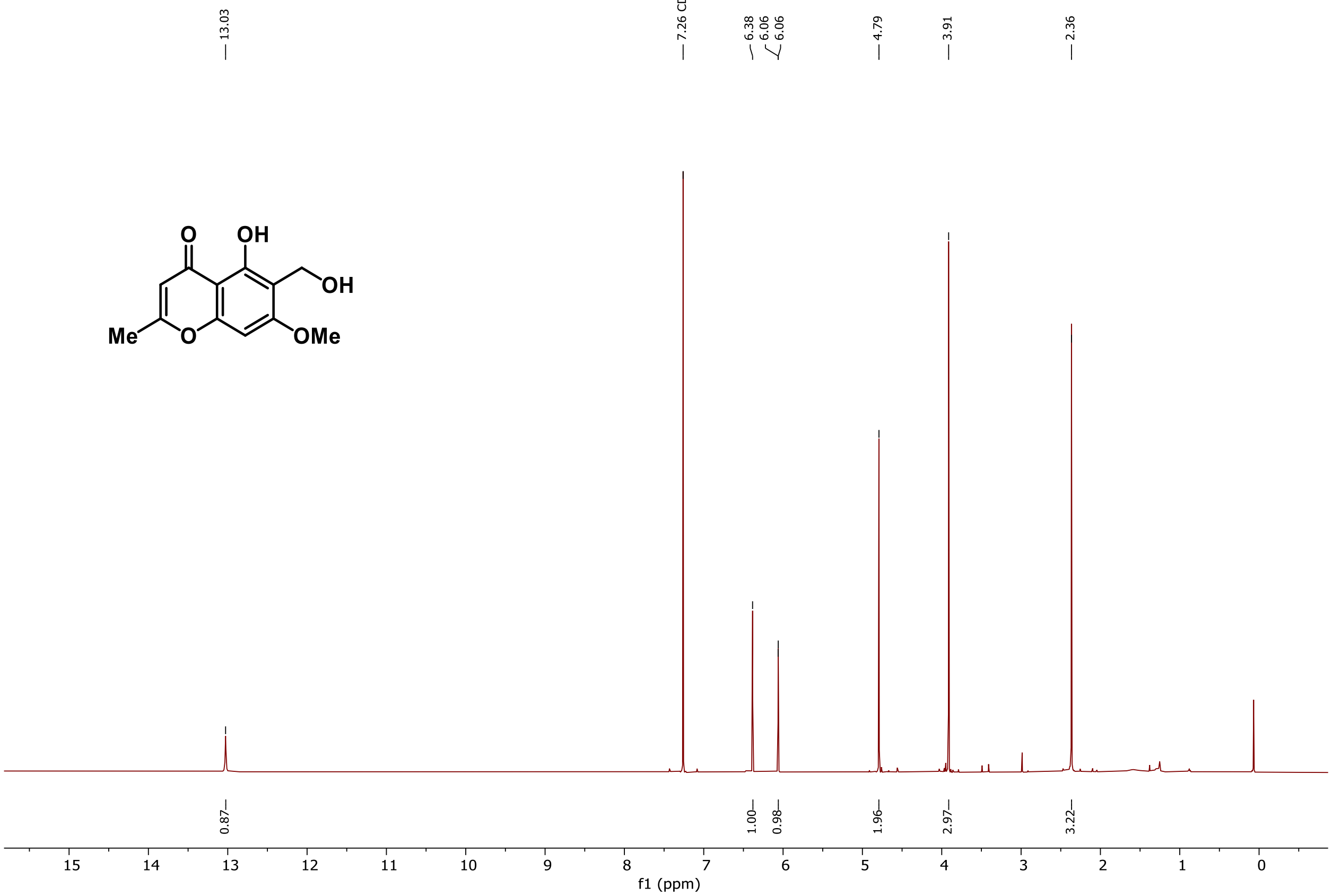

^{13}C-NMR (151 MHz, $CDCl_3$) of 6-Hydroxymethyleugenin (12):

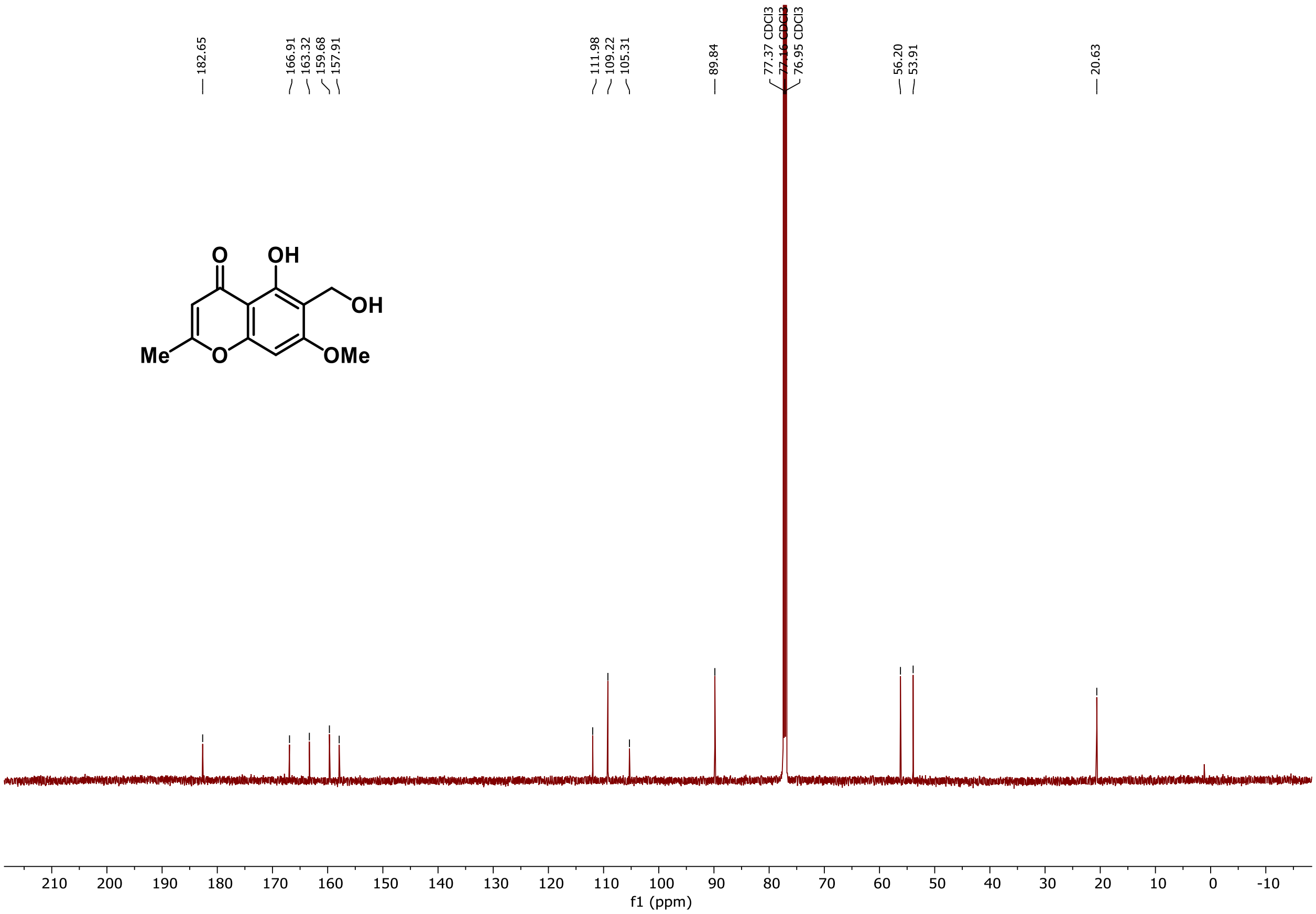

HSQC of 6-Hydroxymethyleugenin (12):

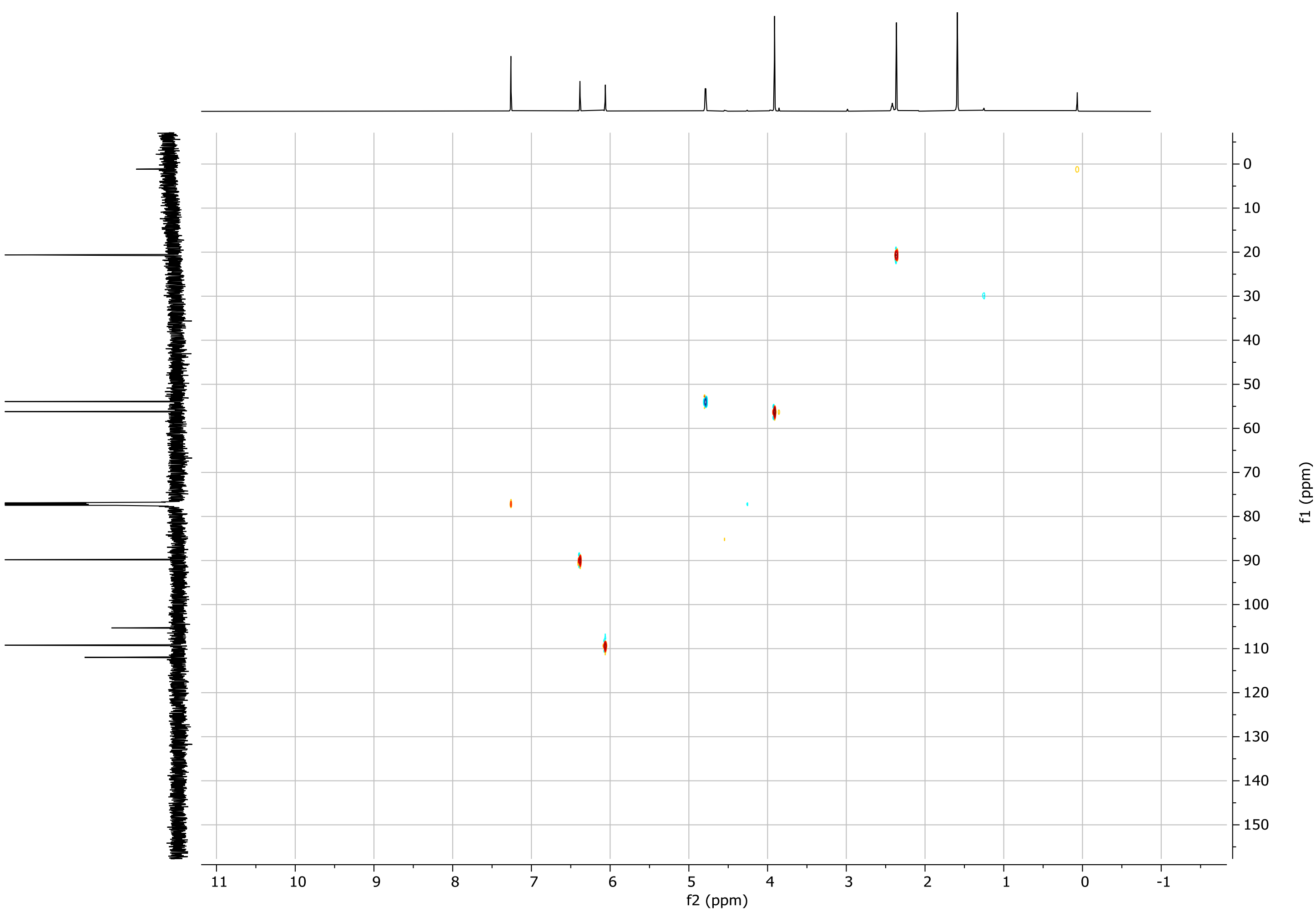

HMBC of 6-Hydroxymethyleugenin (12):

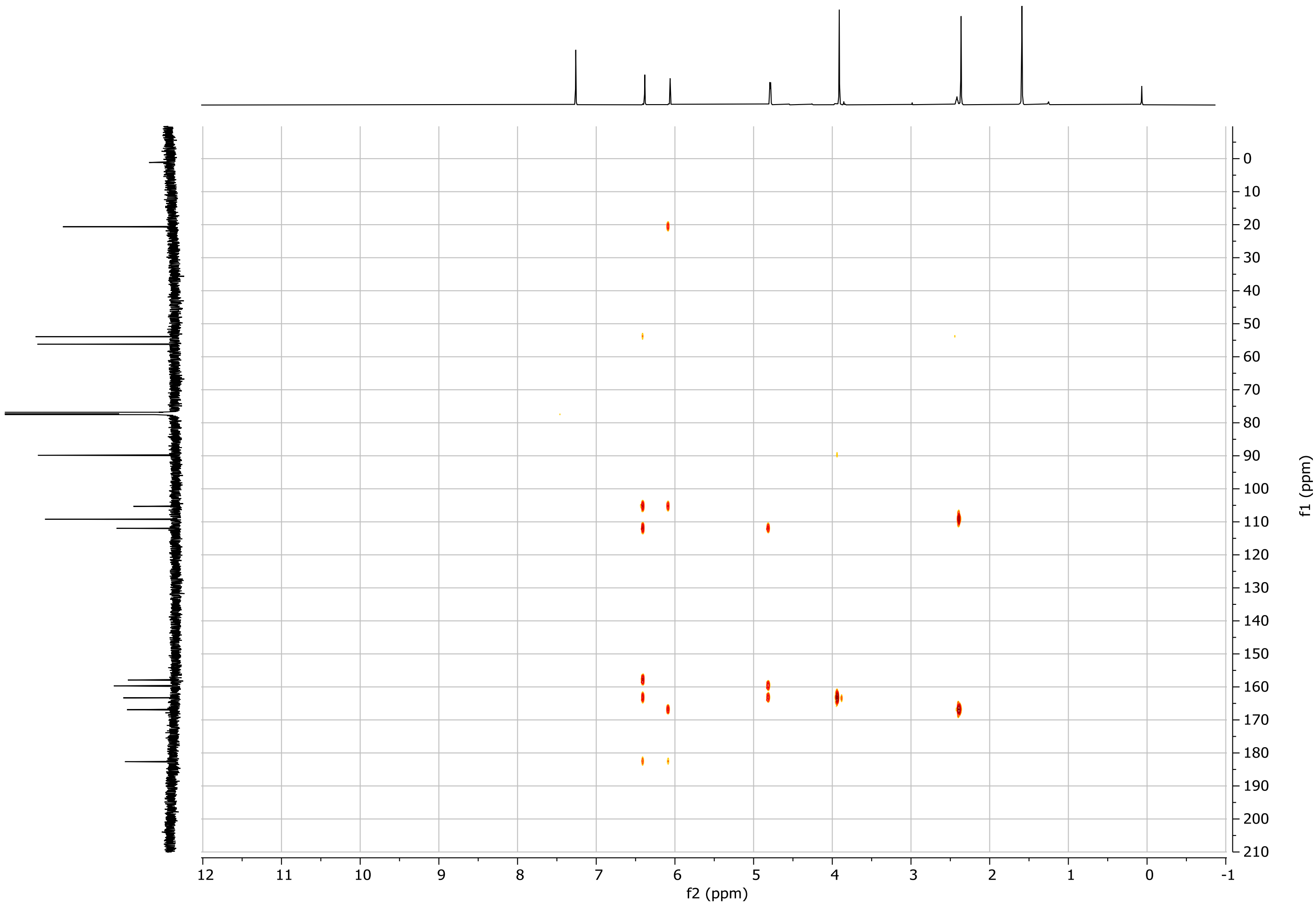

^{1}H-NMR (599 MHz, $CDCl_3$) of methyl (*R*)-2-hydroxy-3-((phenylsulfonyl)thio)propanoate (S12)

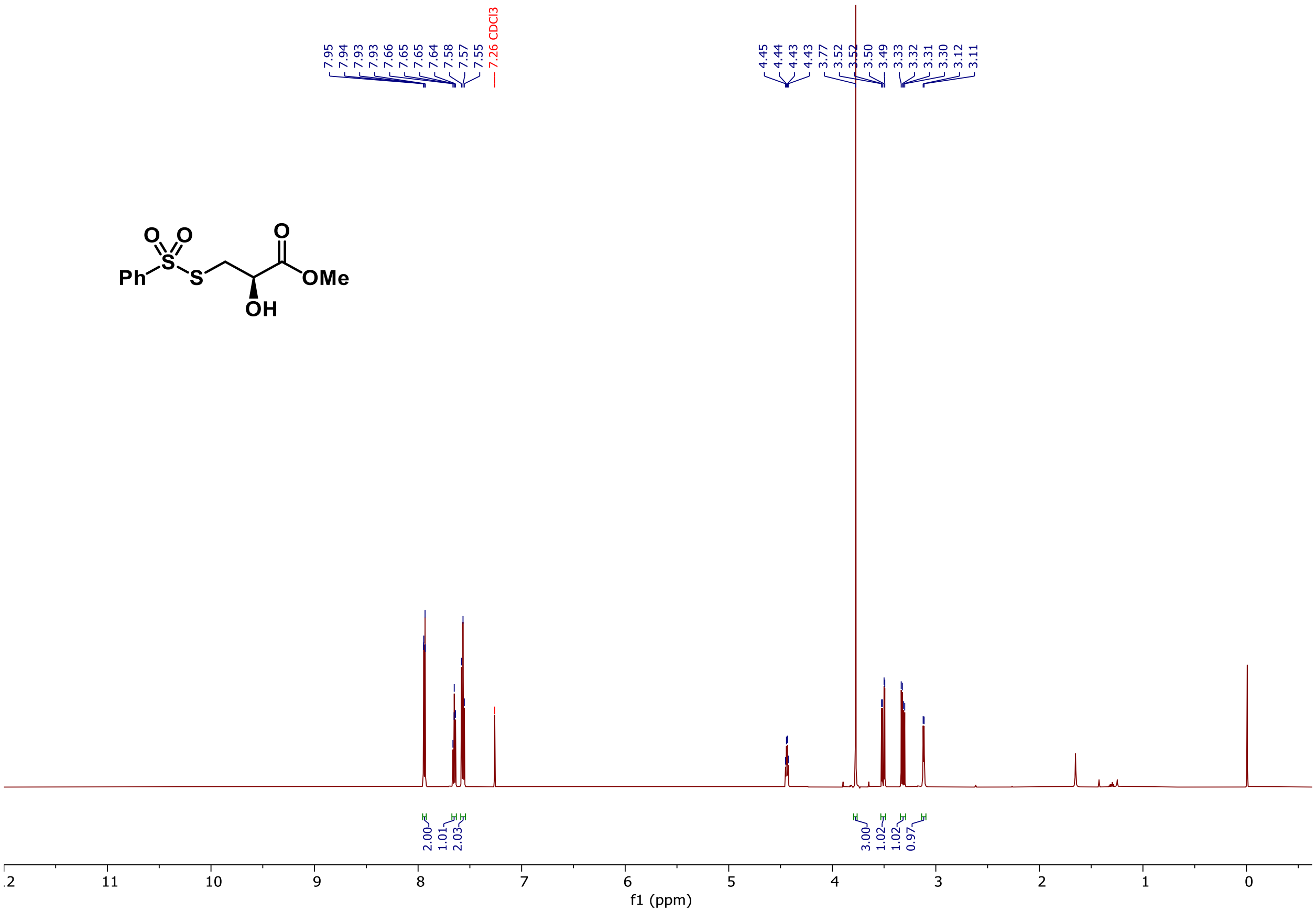

^{13}C-NMR (151 MHz, $CDCl_3$) of methyl (*R*)-2-hydroxy-3-((phenylsulfonyl)thio)propanoate (S12)

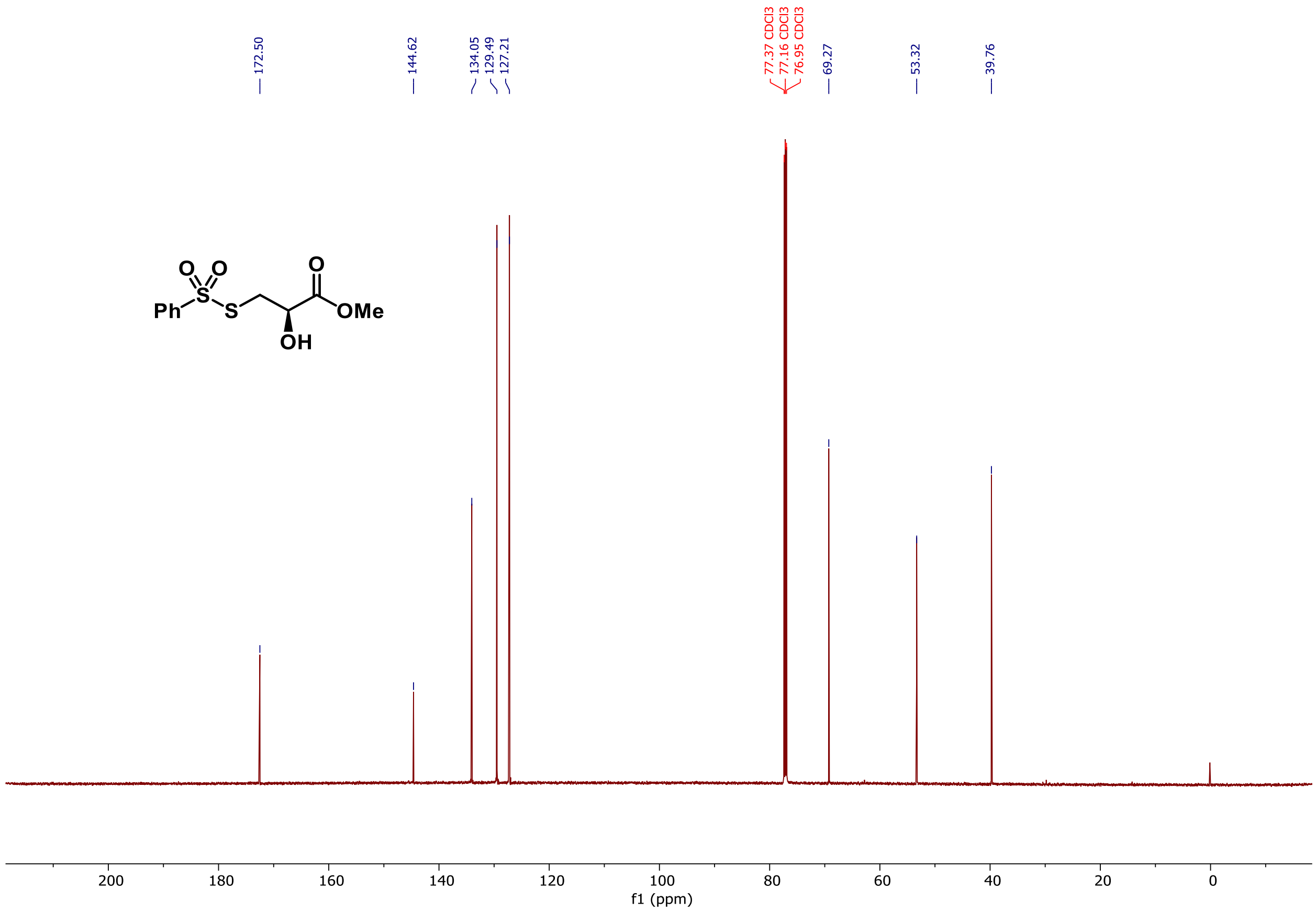

^{1}H-NMR (599 MHz, $CDCl_3$) of methyl (*R*)-2-hydroxy-3-mercaptopropanoate (S13)

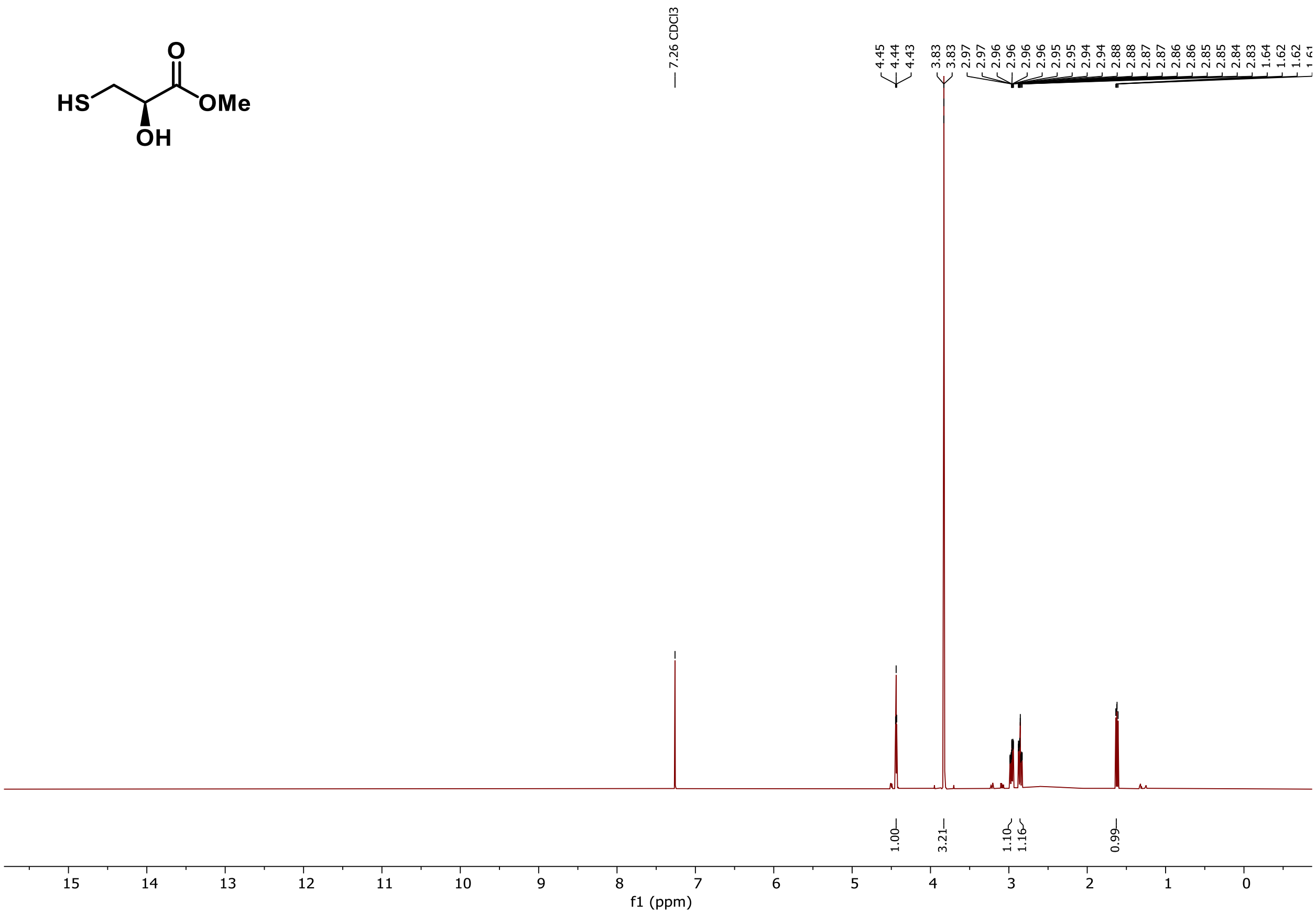

^{13}C-NMR (151 MHz, CDCl$_3$) of methyl (*R*)-2-hydroxy-3-mercaptopropanoate (S13)

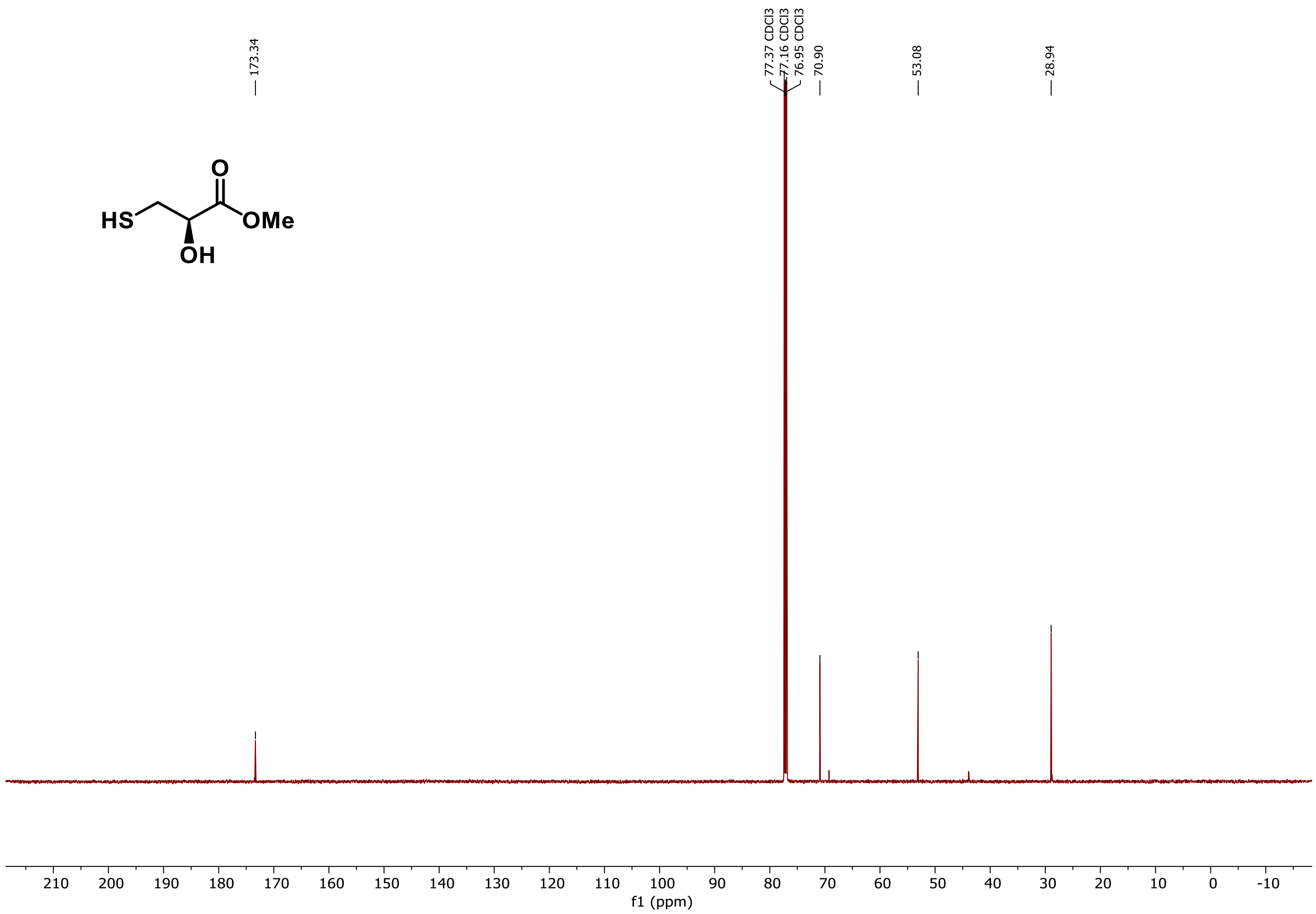

COSY of methyl (*R*)-2-hydroxy-3-mercaptopropanoate (S13)

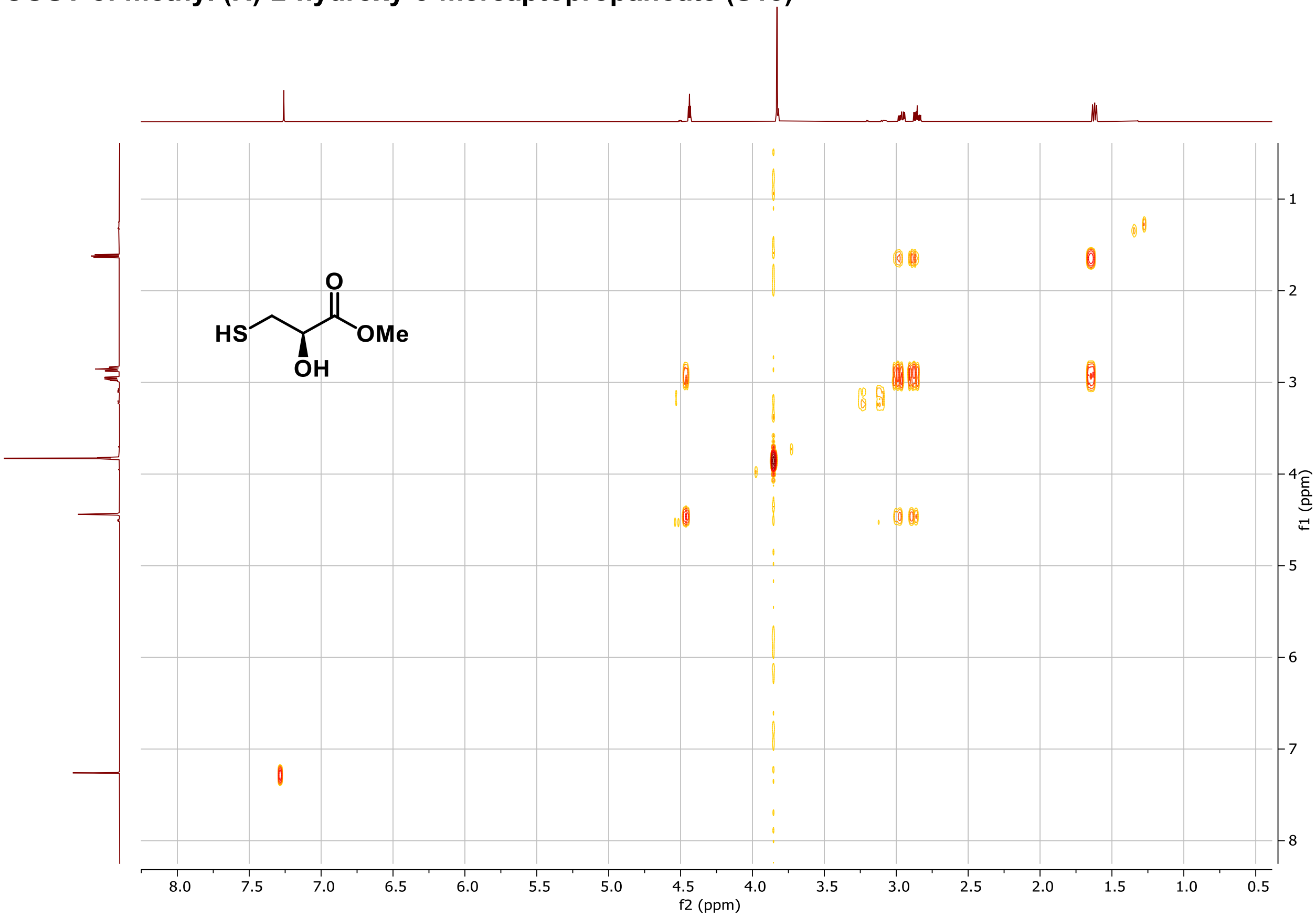

^{1}H-NMR (500 MHz, d_6-DMSO) of (*R*)-2-hydroxy-3-mercaptopropanoic acid (S14):

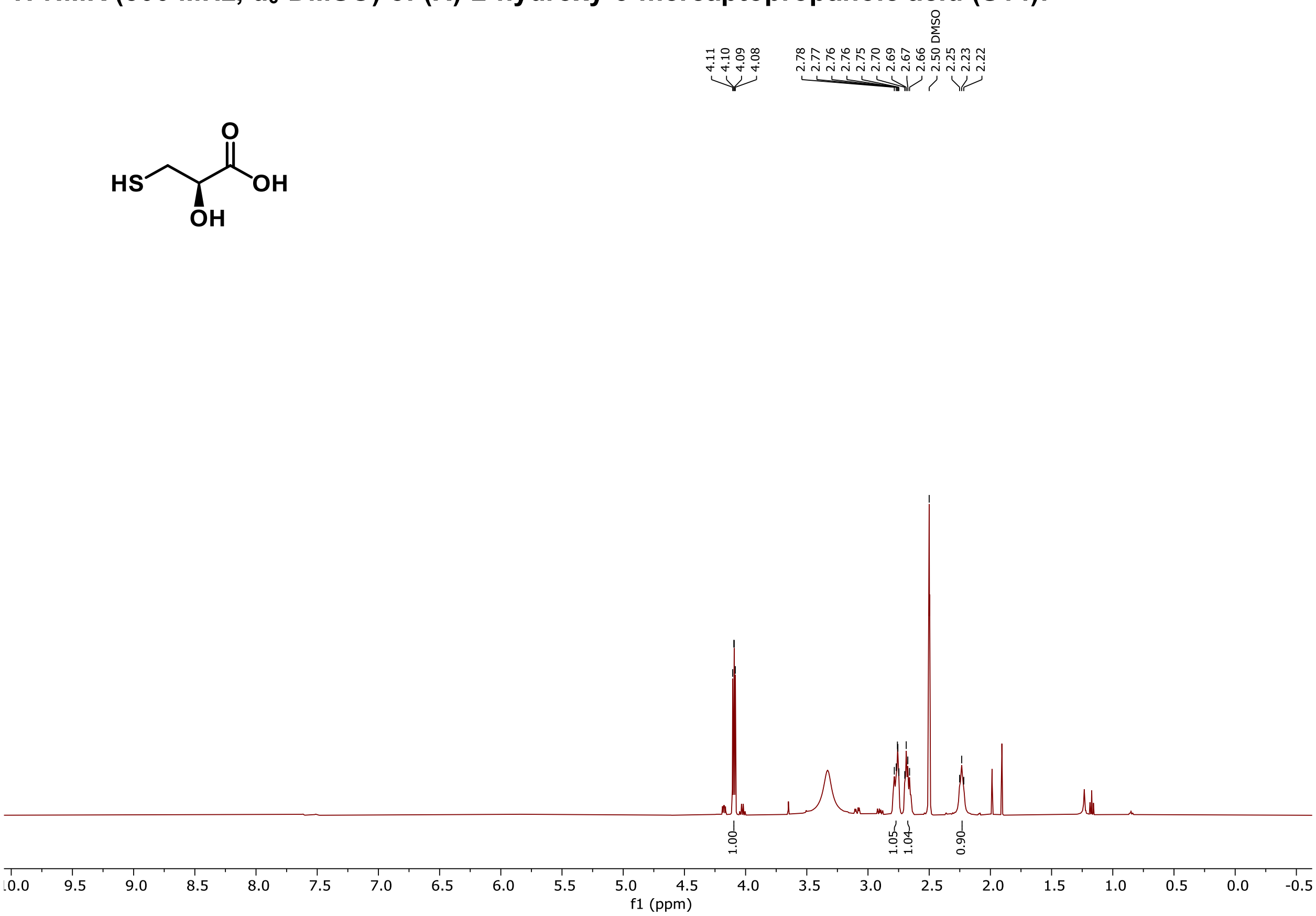

^{13}C-NMR (126 MHz, d_6-DMSO) of (*R*)-2-hydroxy-3-mercaptopropanoic acid (S14):

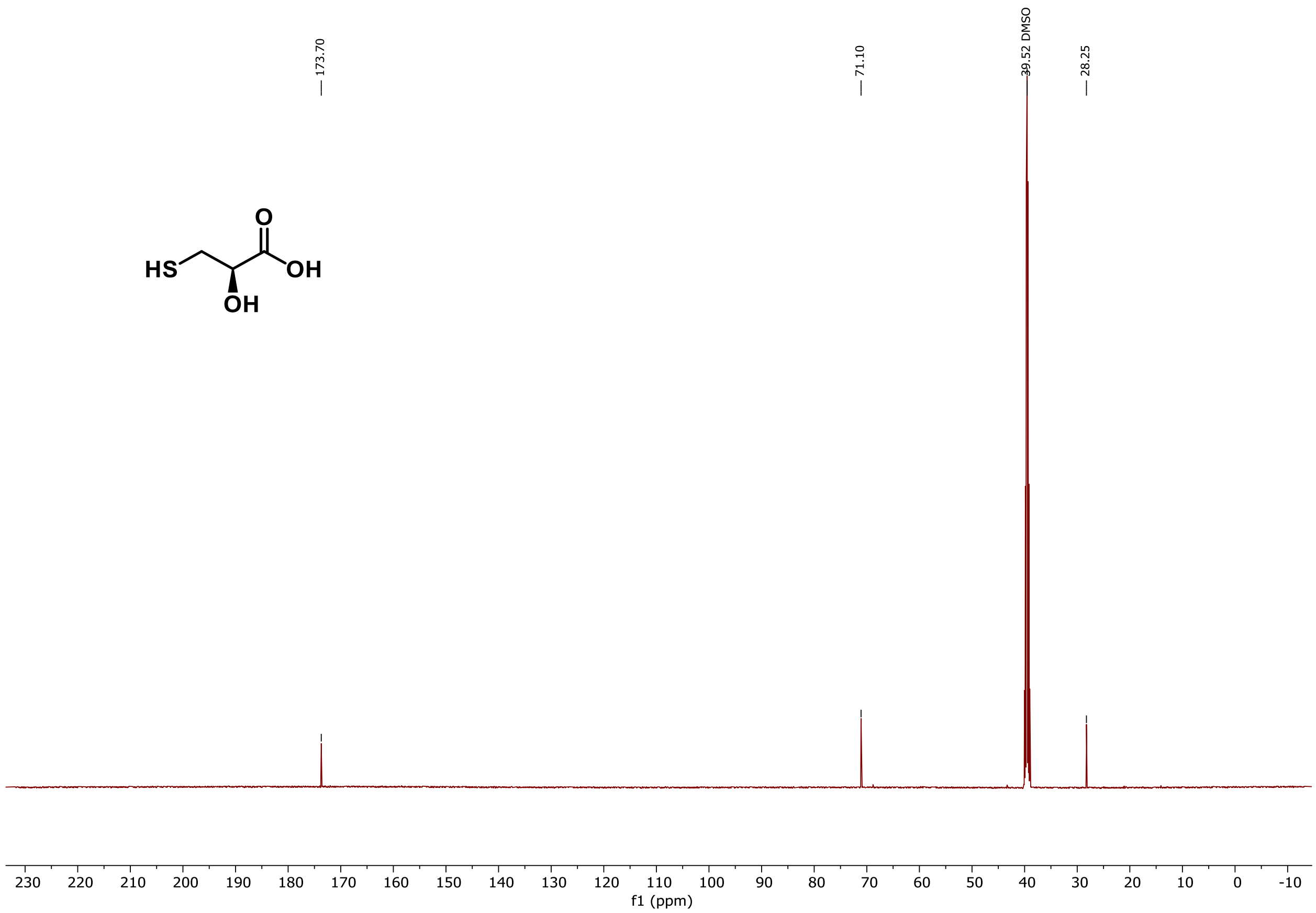

^{1}H-NMR (599 MHz, CDCl$_3$) of methyl (*S*)-2-hydroxy-3-((phenylsulfonyl)thio)propanoate (S15):

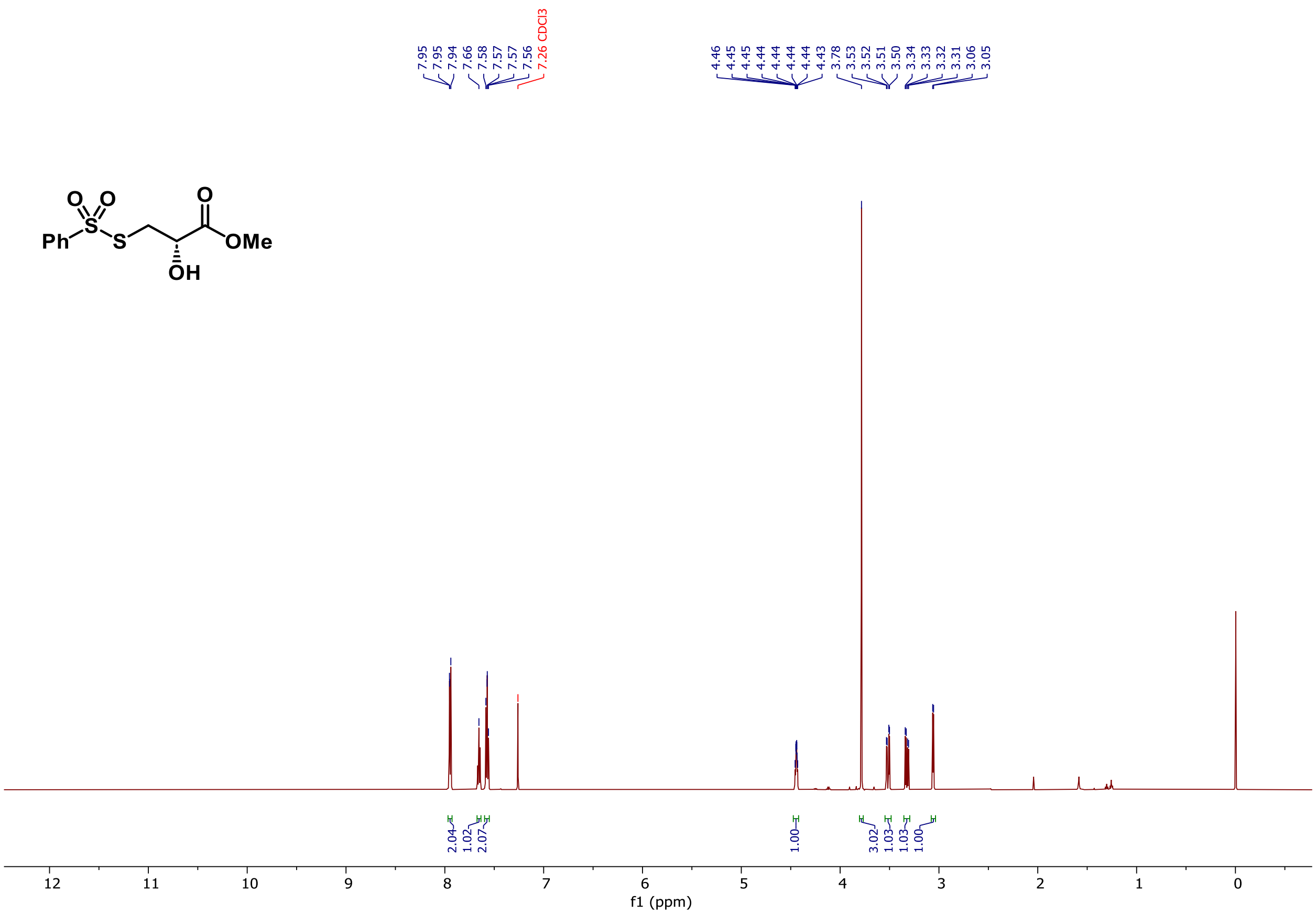

^{13}C-NMR (151 MHz, $CDCl_3$) of methyl (*S*)-2-hydroxy-3-((phenylsulfonyl)thio)propanoate (S15):

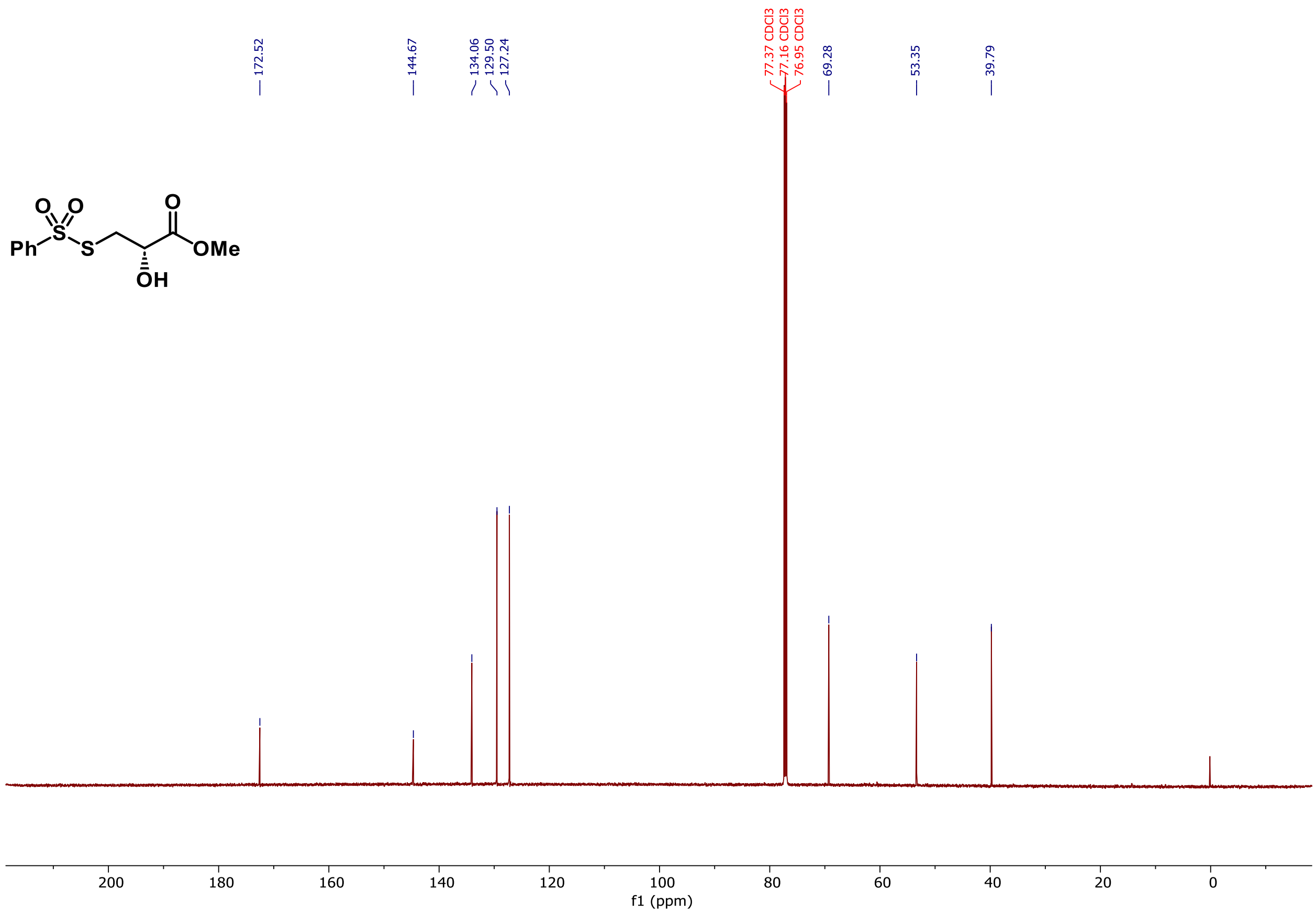

^{1}H-NMR (599 MHz, $CDCl_3$) of methyl (*S*)-2-hydroxy-3-mercaptopropanoate (18):

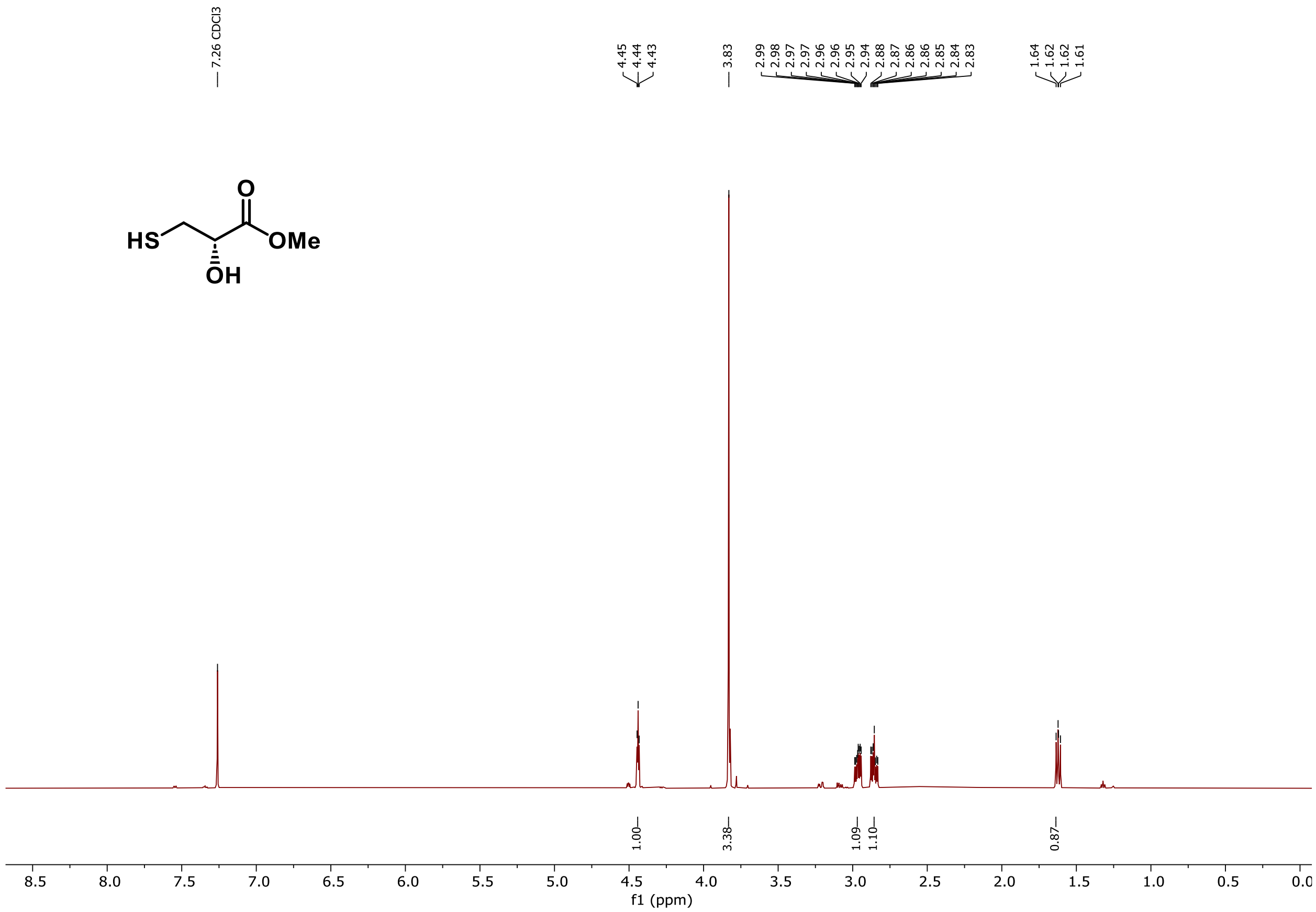

^{13}C-NMR (151 MHz, $CDCl_3$) of methyl (S)-2-hydroxy-3-mercaptopropanoate (18):

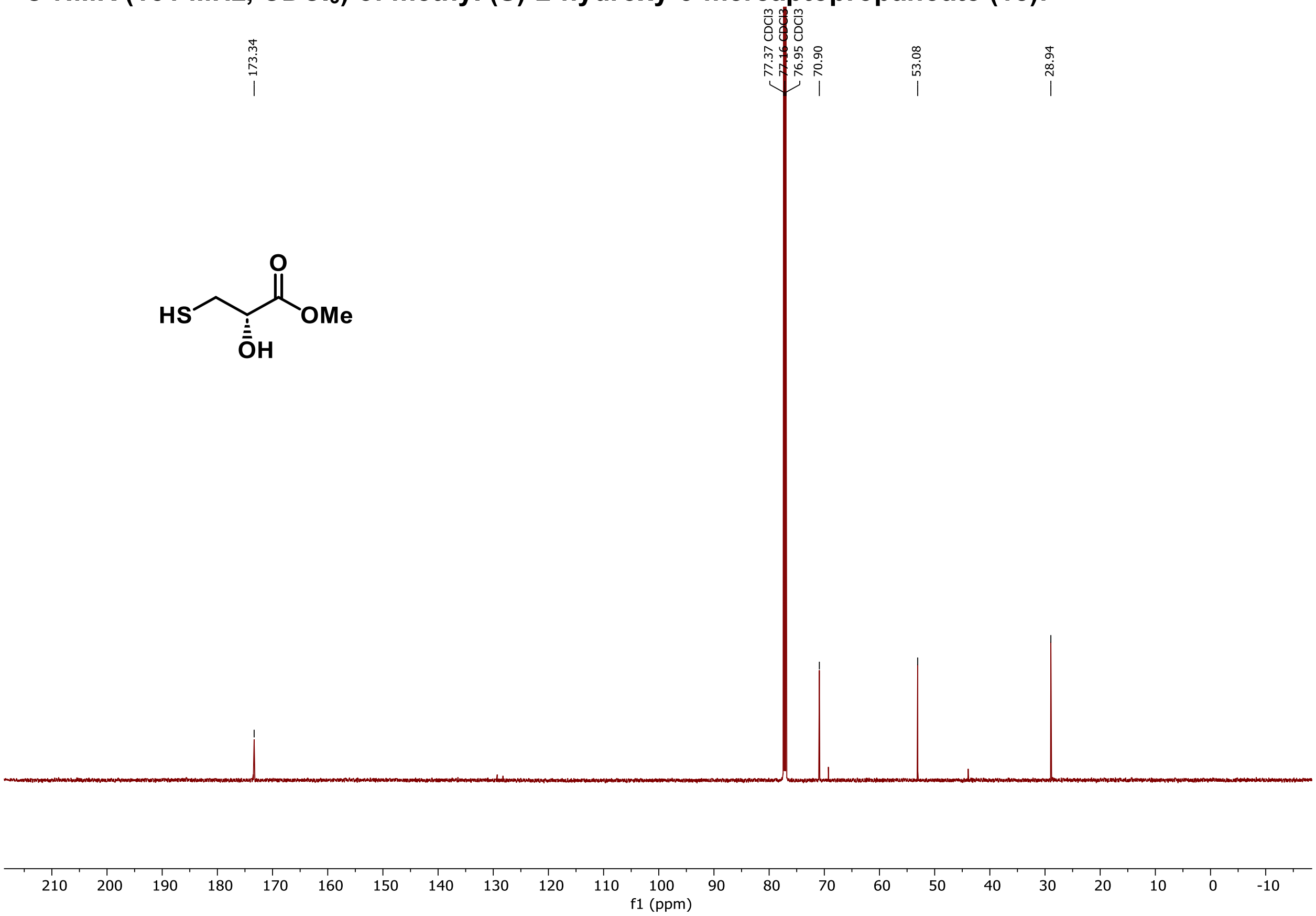

^{1}H-NMR (599 MHz, d$_6$-DMSO) of methyl (*S*)-2-hydroxy-3-mercaptopropanoic acid (19):

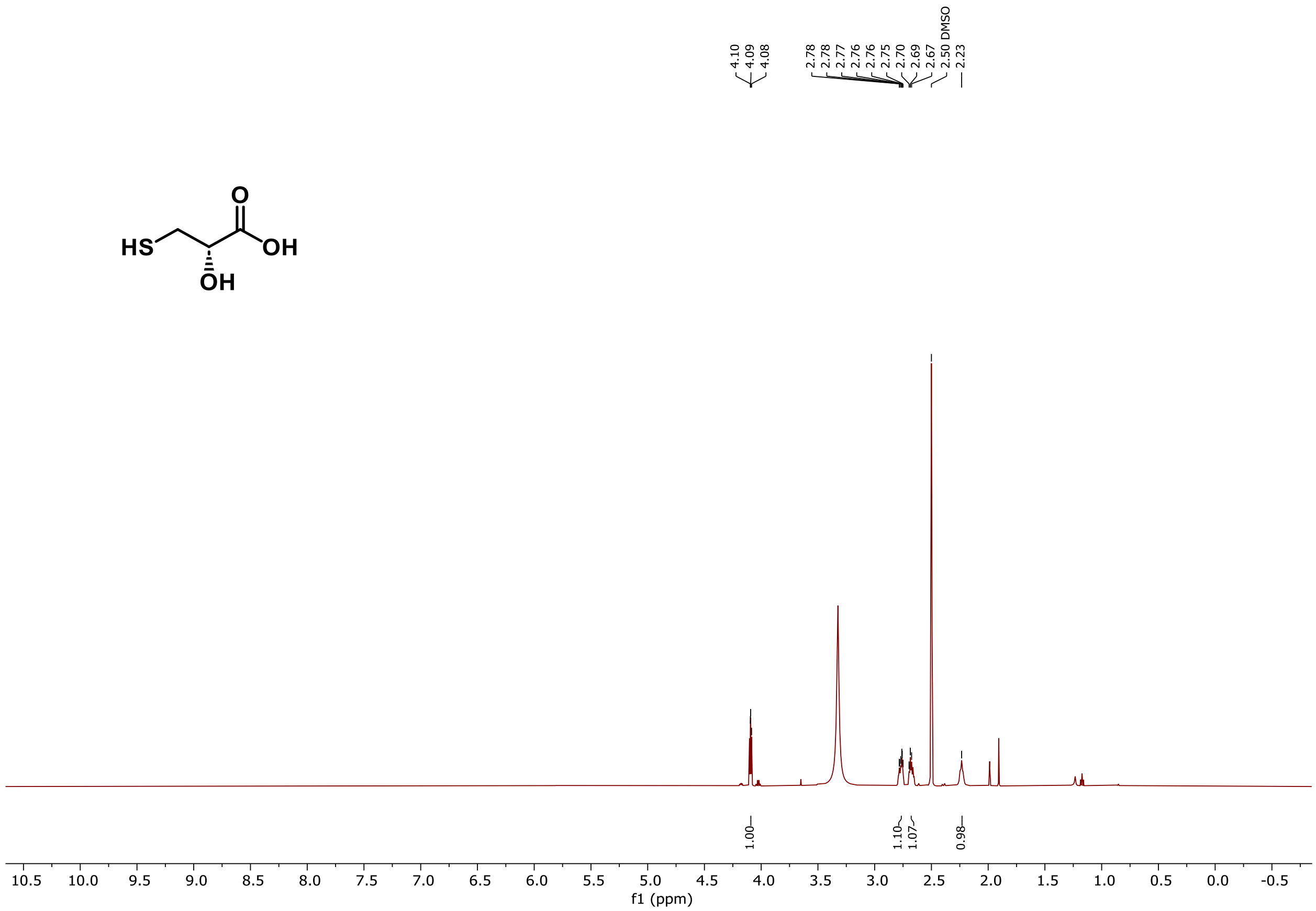

^{13}C-NMR (151 MHz, d$_6$-DMSO) of methyl (*S*)-2-hydroxy-3-mercaptopropanoic acid (19):

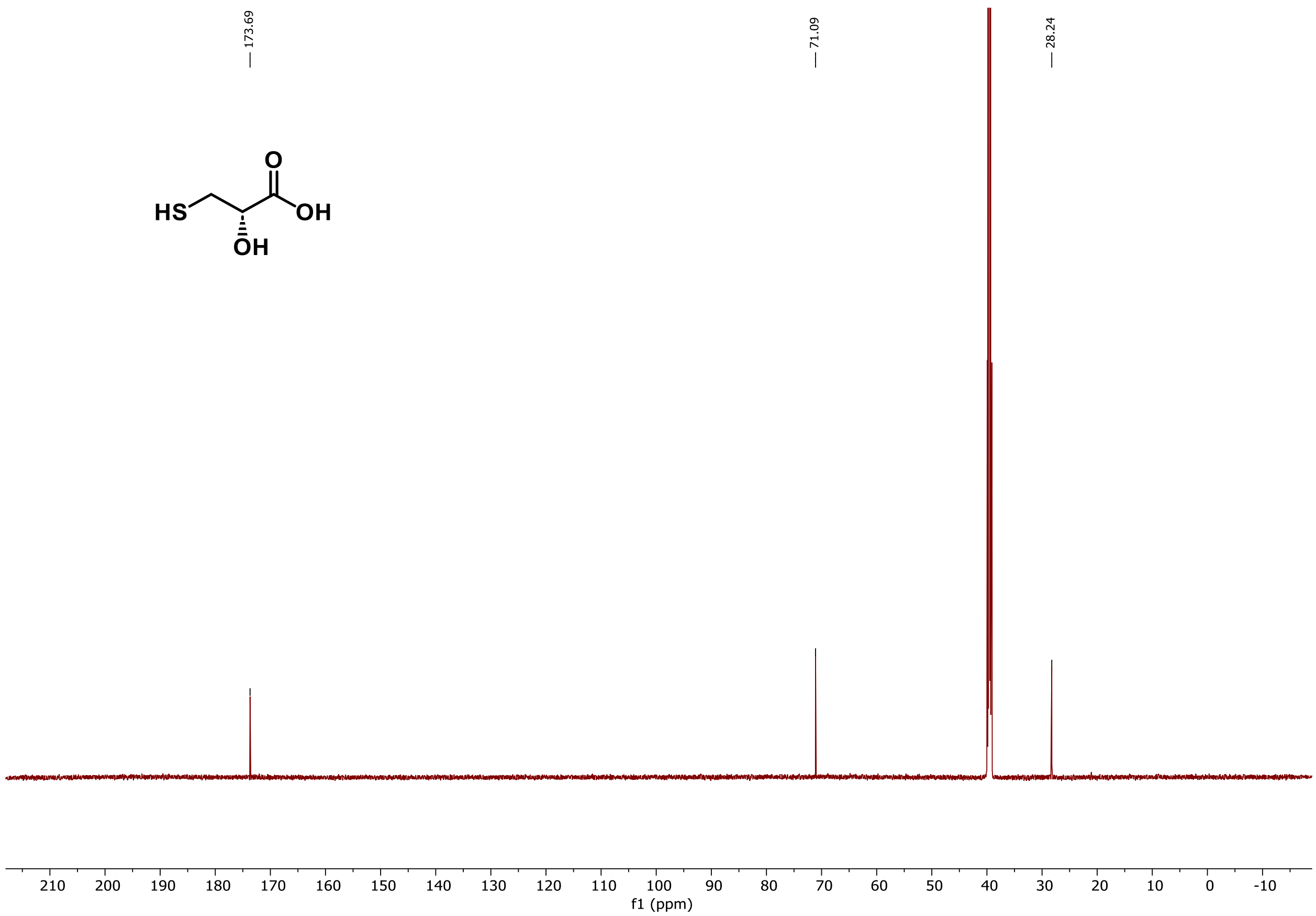

173.69
71.09
28.24
HS
O
OH
OH
210
200
190
180
170
160
150
140
130
120
110
100
90
80
70
60
50
40
30
20
10
0
-10
f1 (ppm)

^{1}H-NMR (599 MHz, $CDCl_3$) of (*ent*)-chaetosemin B (S16):

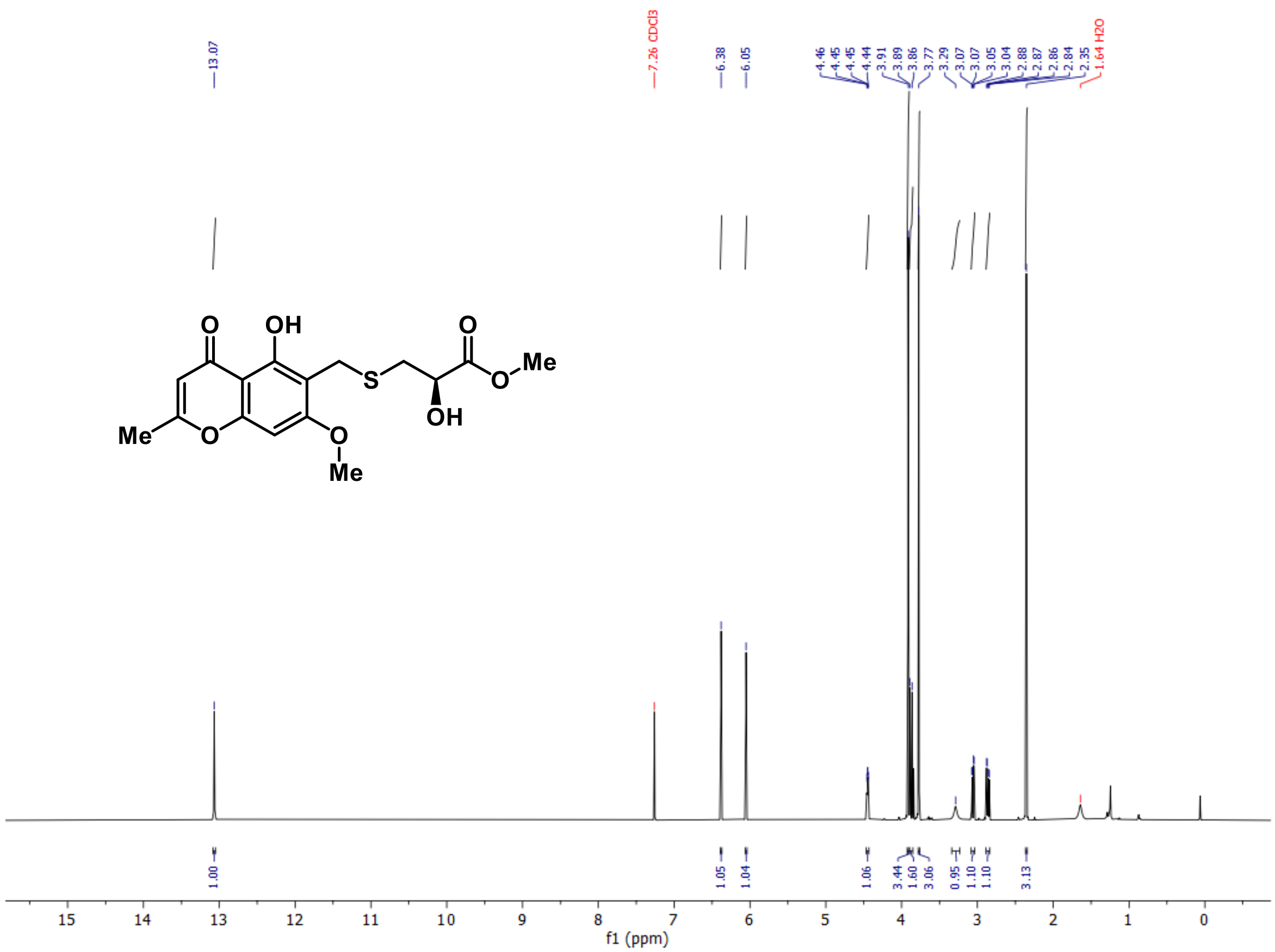

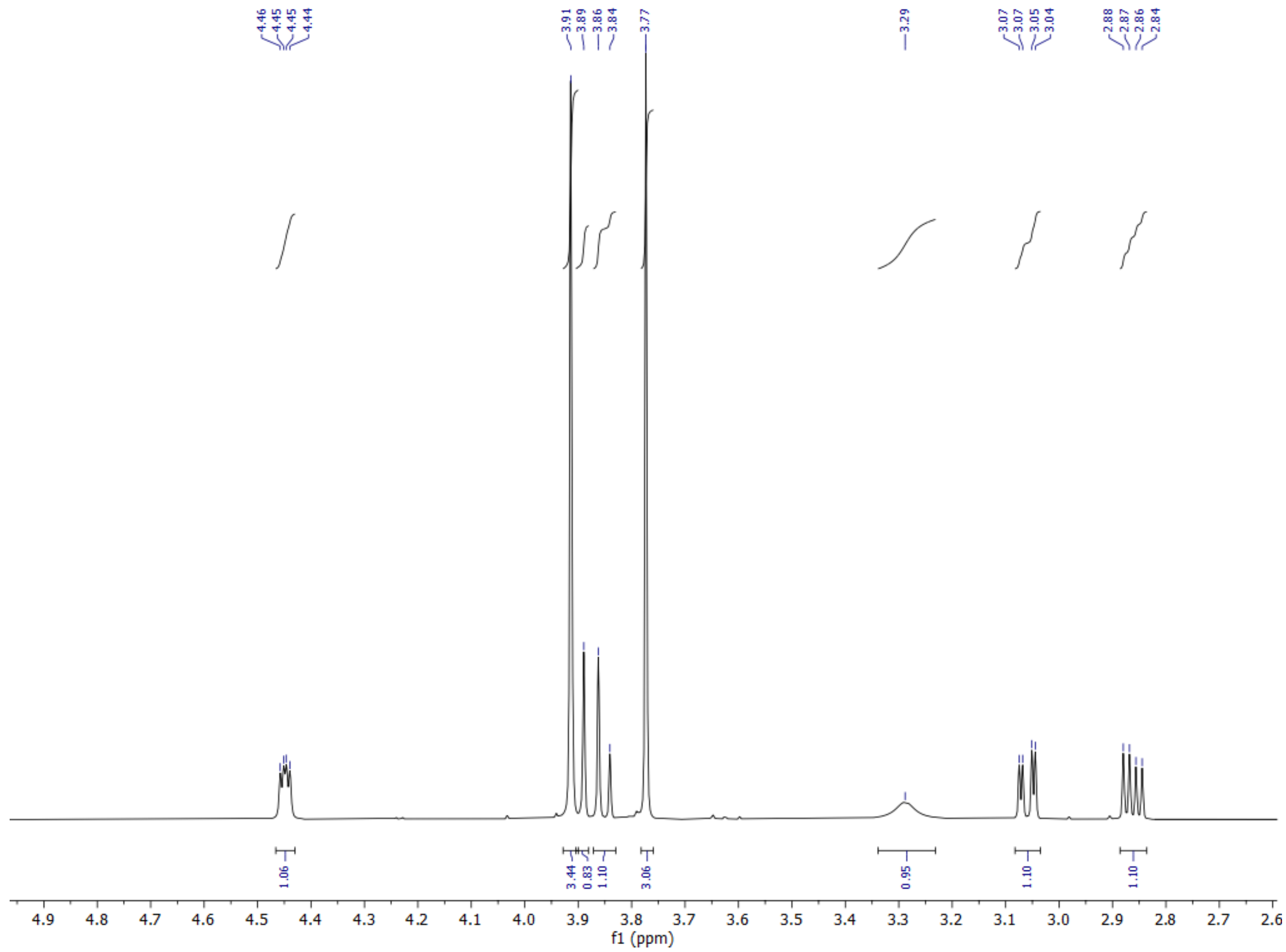

4.46
4.45
4.45
4.44
3.91
3.89
3.86
3.84
3.77
3.29
3.07
3.07
3.05
3.04
2.88
2.87
2.86
2.84
1.06
3.44
0.83
1.10
3.06
0.95
1.10
1.10
4.9
4.8
4.7
4.6
4.5
4.4
4.3
4.2
4.1
4.0
3.9
3.8
3.7
3.6
3.5
3.4
3.3
3.2
3.1
3.0
2.9
2.8
2.7
2.6
f1 (ppm)

^{13}C-NMR (151 MHz, $CDCl_3$) of (*ent*)-chaetosemin B (S16):

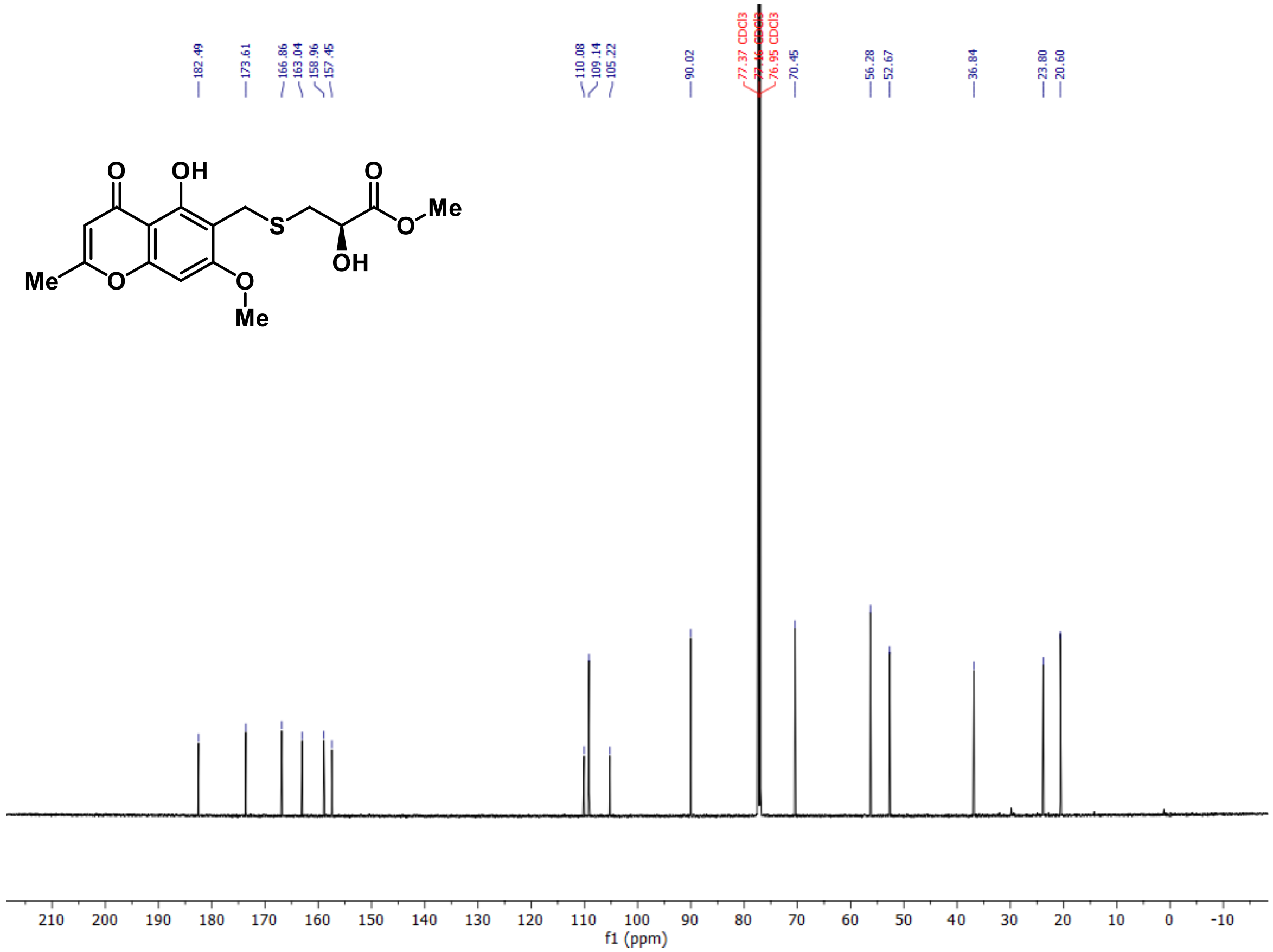

COSY of (*ent*)-chaetosemin B (S16):

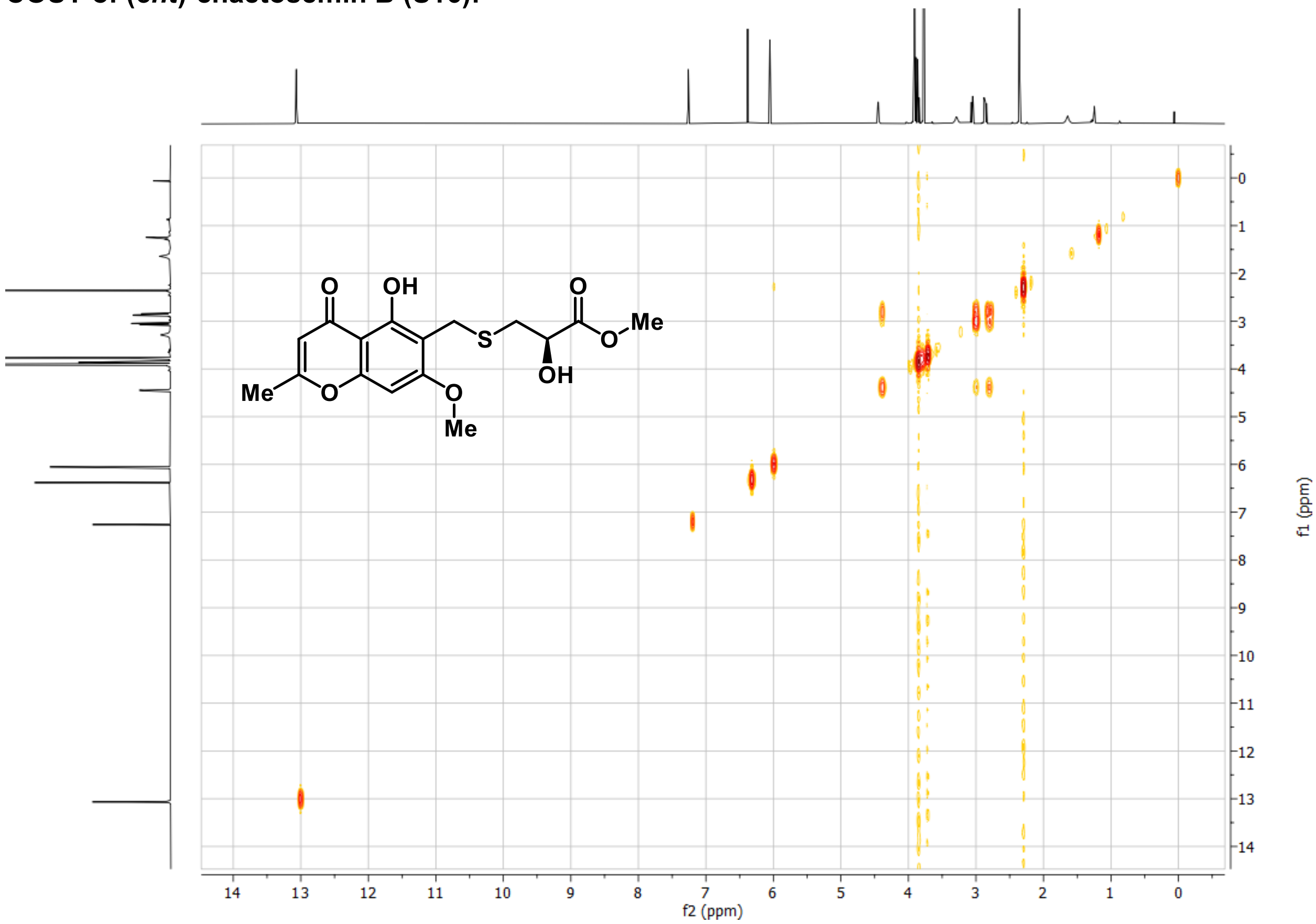

HSQC of (*ent*)-chaetosemin B (S16):

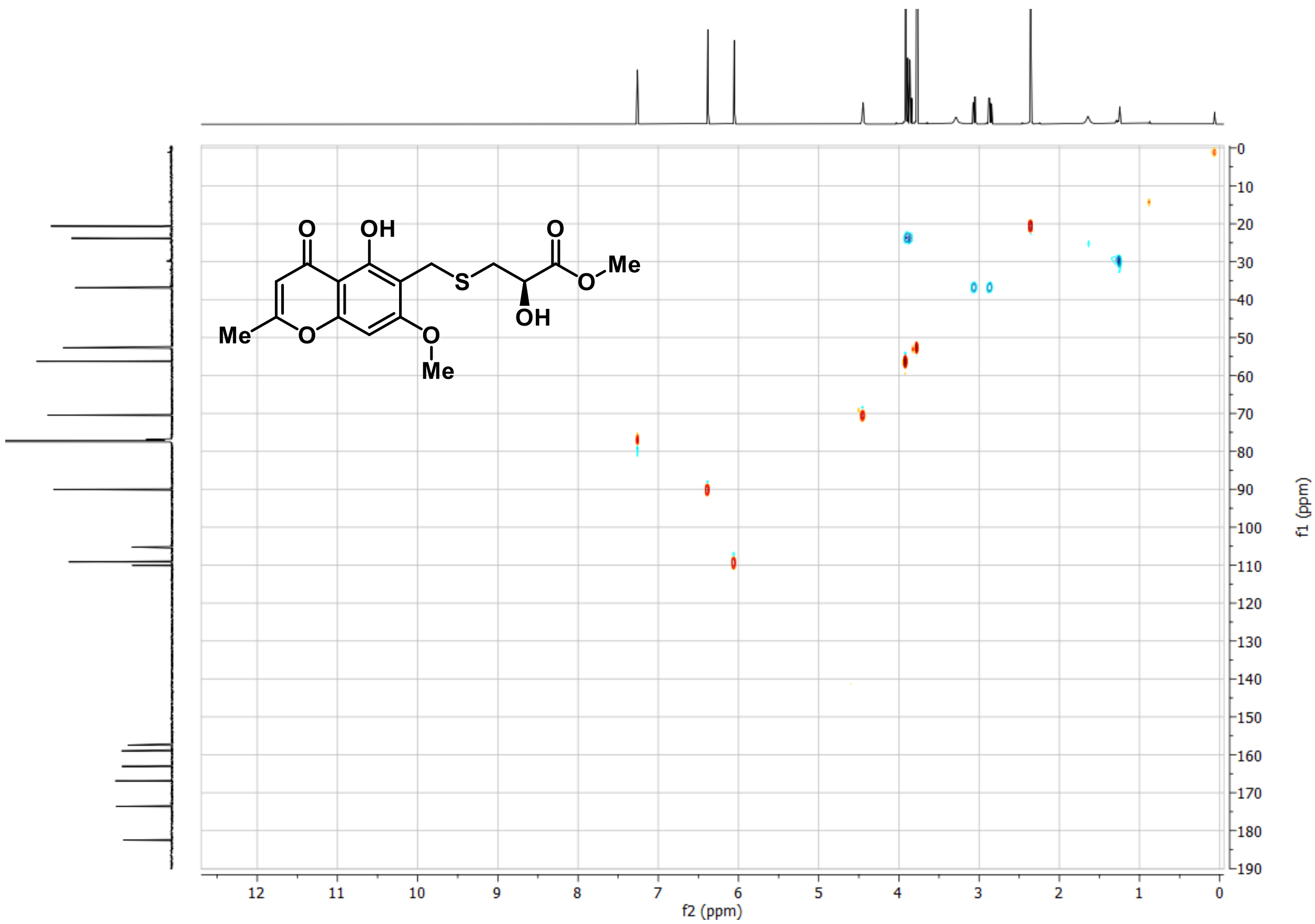

HMBC of (*ent*)-chaetosemin B (S16):

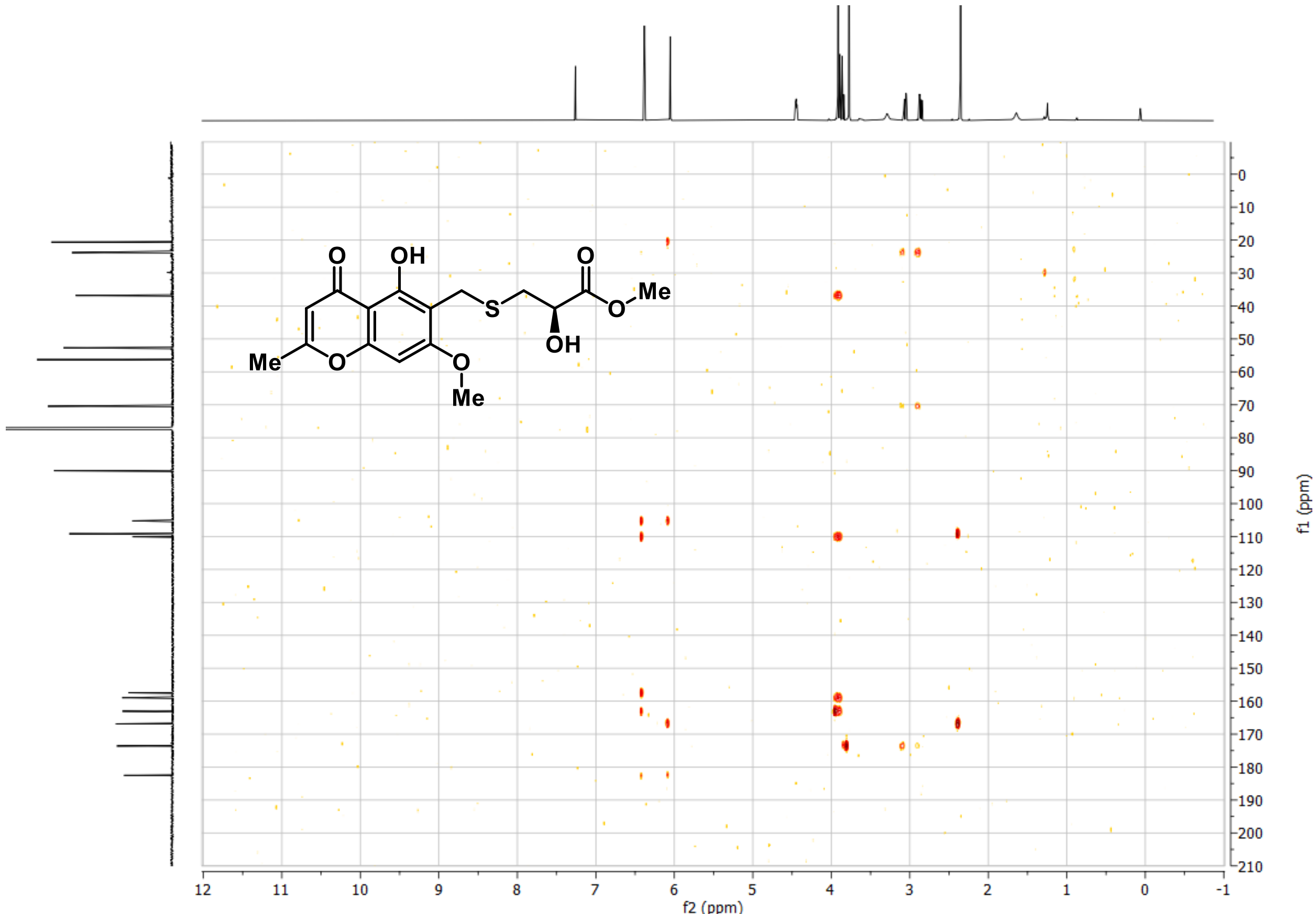

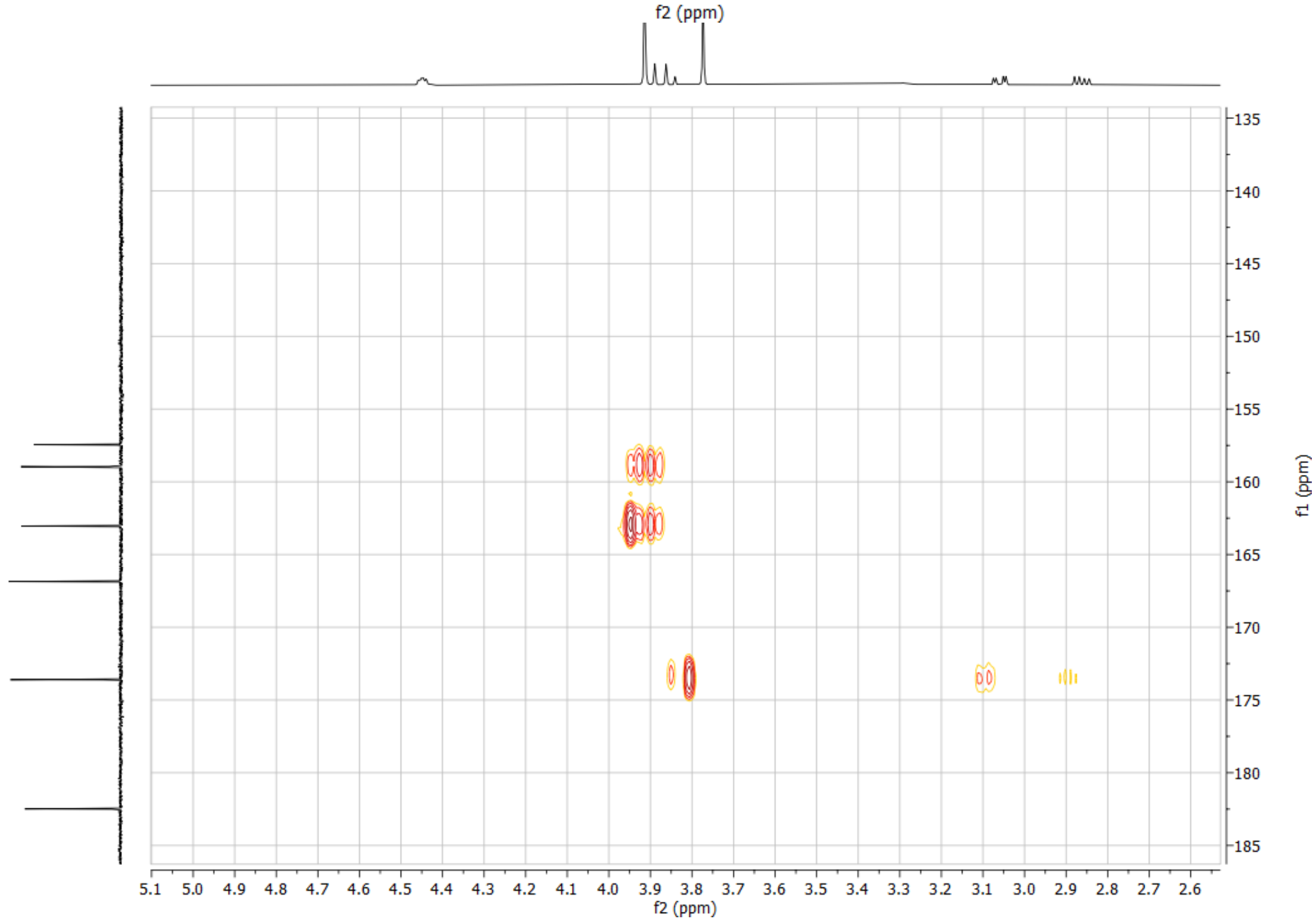

^{1}H-NMR (599 MHz, $CDCl_3$) of chaetosemin B (2, revised):

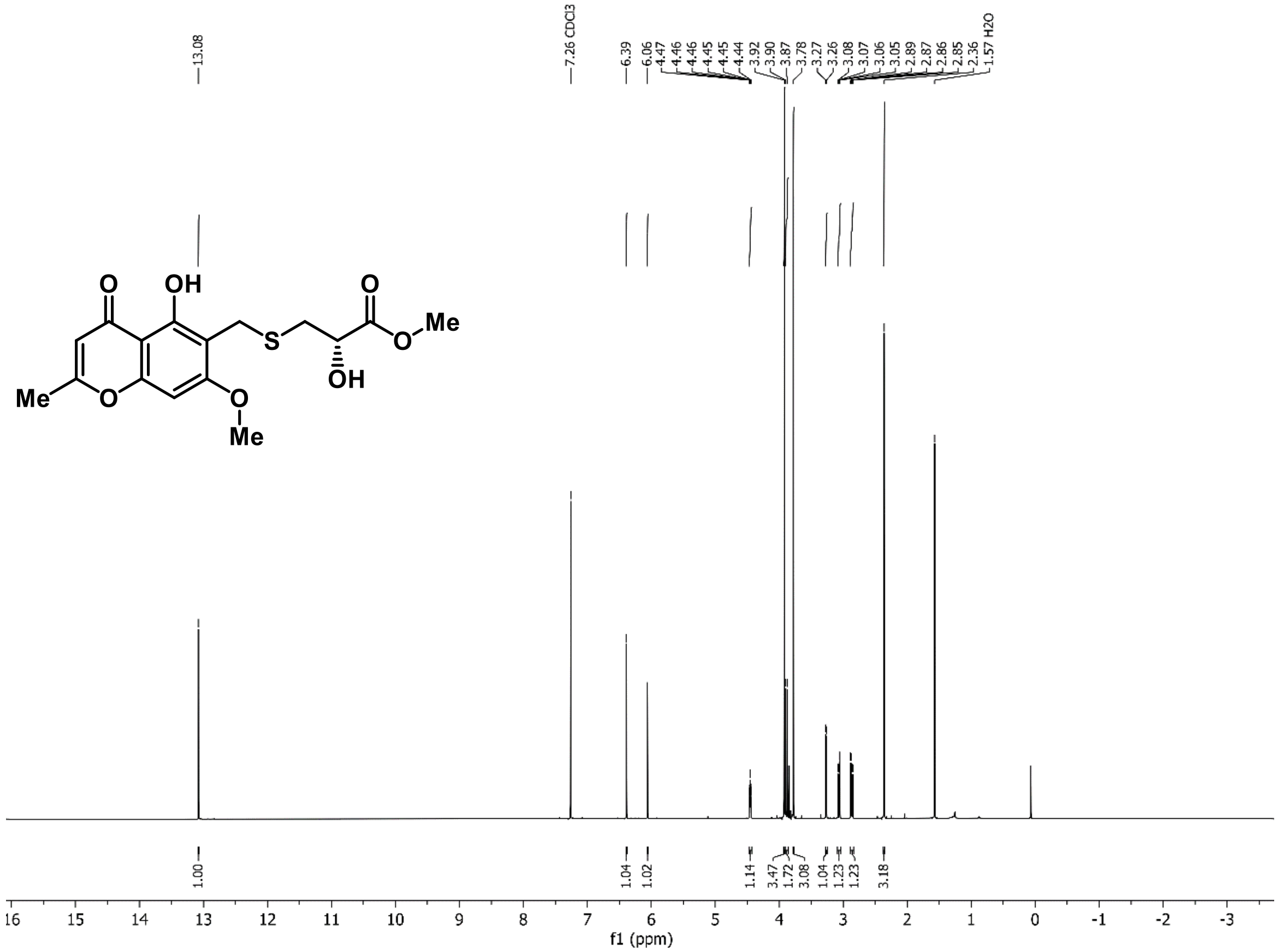

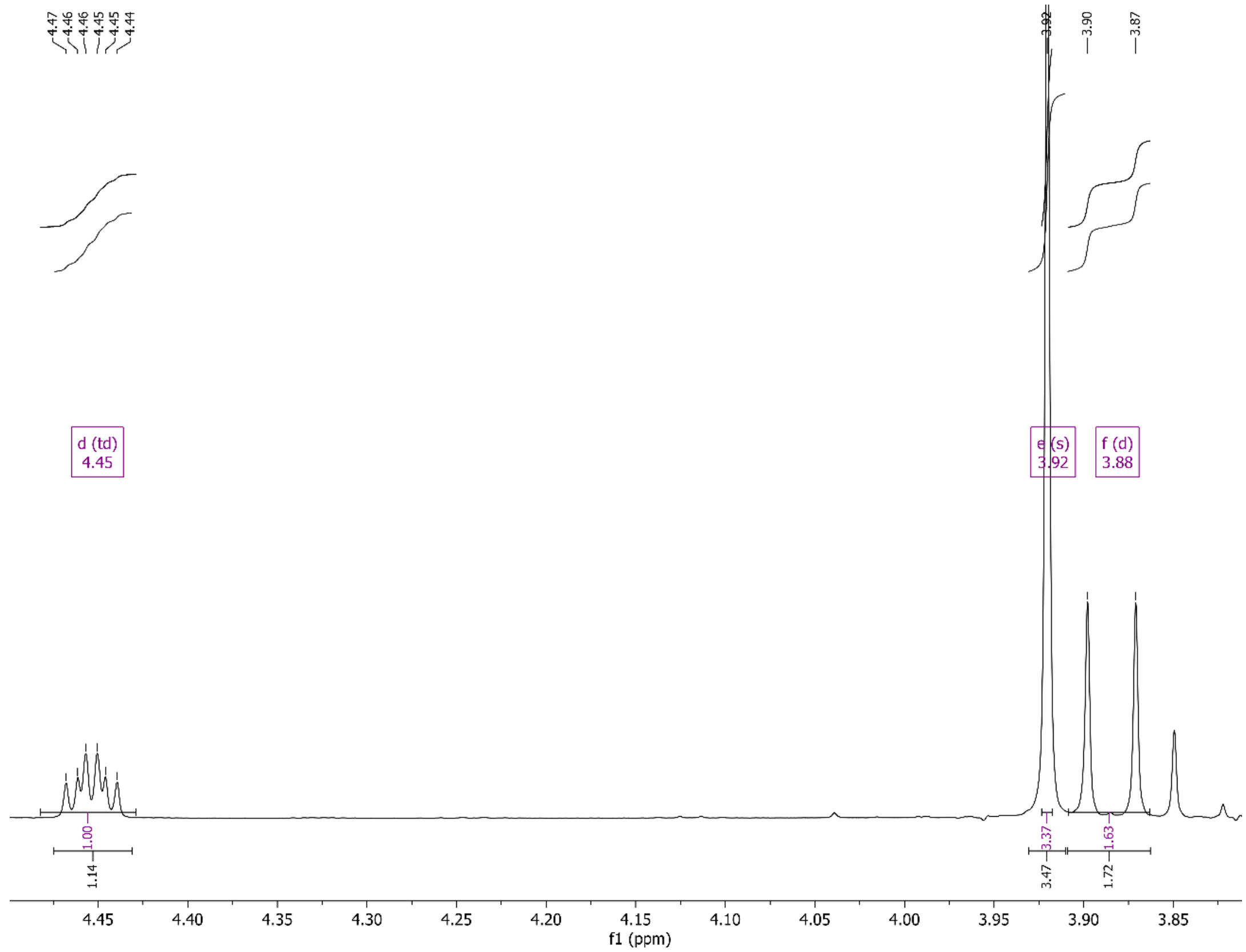
d (td)
4.45
e (s)
3.92
f (d)
3.88
f1 (ppm)

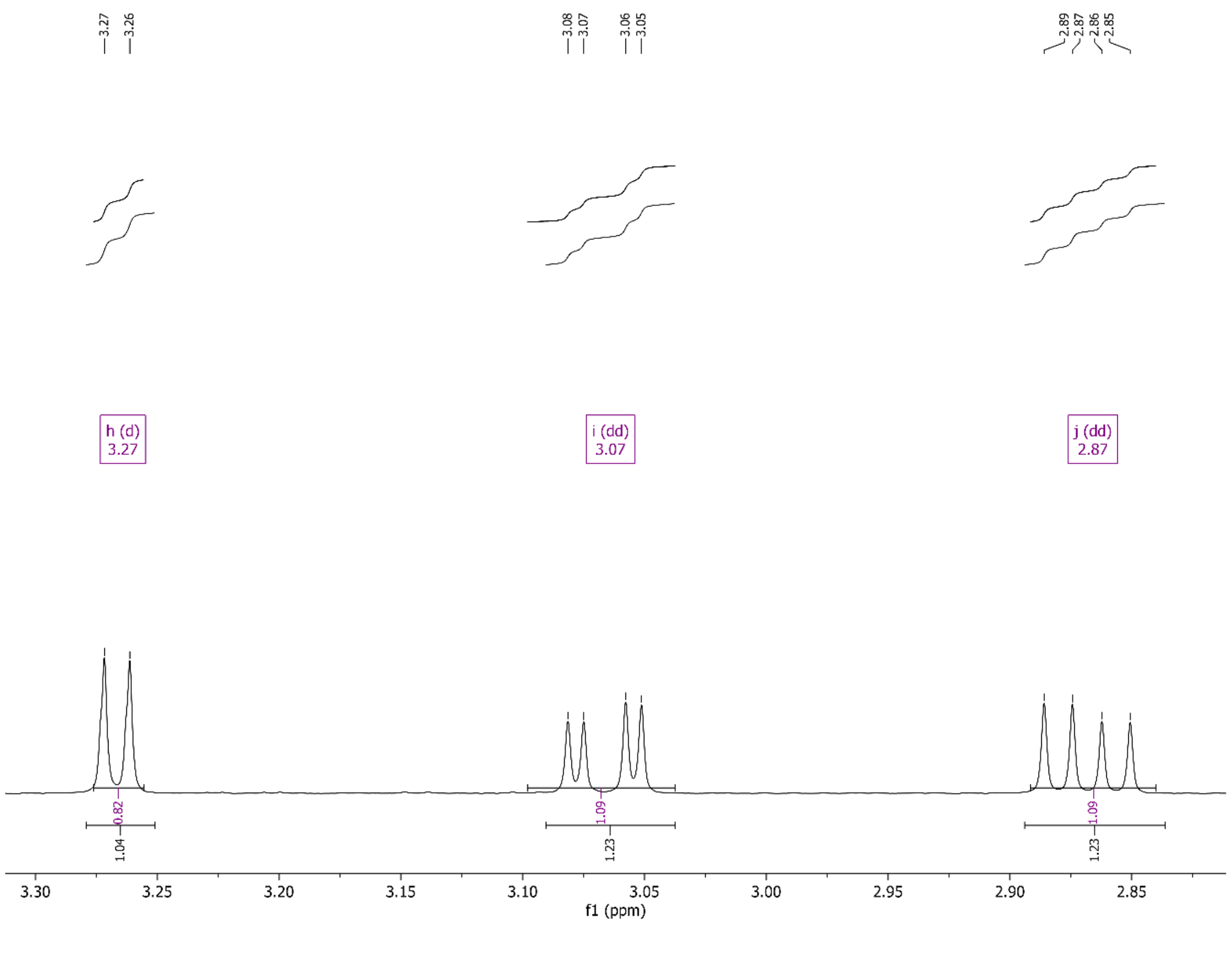
h (d)
3.27
i (dd)
3.07
j (dd)
2.87
f1 (ppm)

^{13}C-NMR (151 MHz, $CDCl_3$) of chaetosemin B (2, revised):

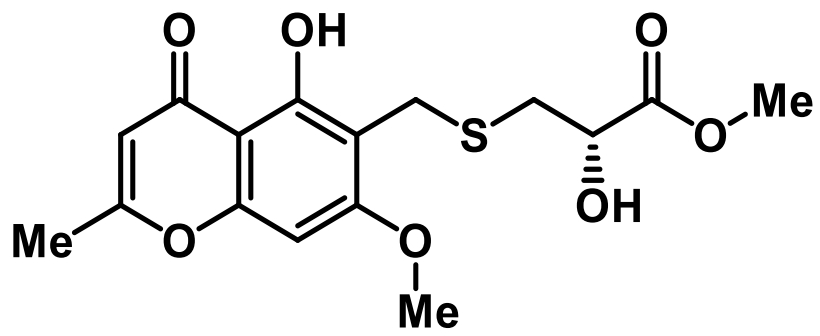

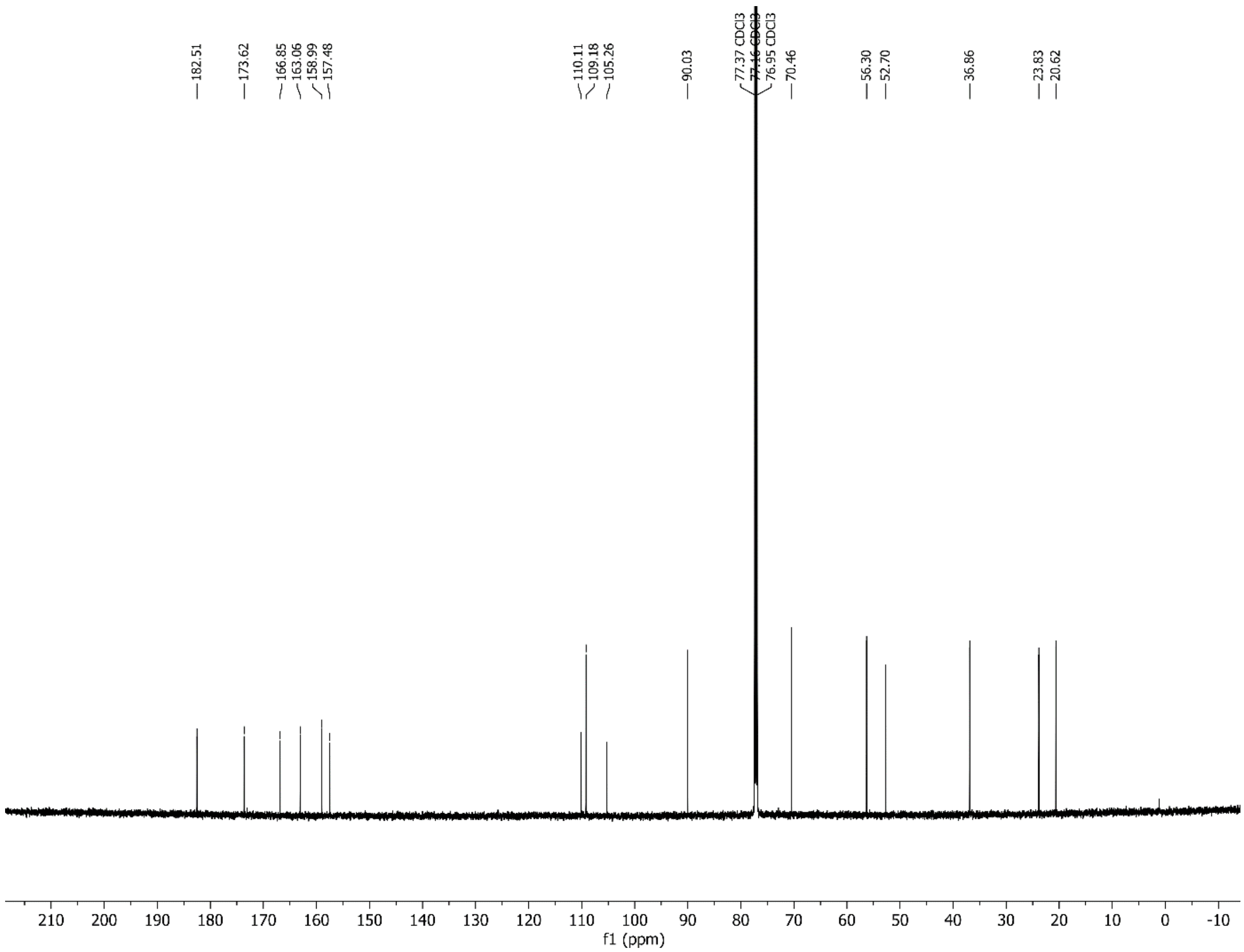

COSY of chaetosemin B (2, revised)

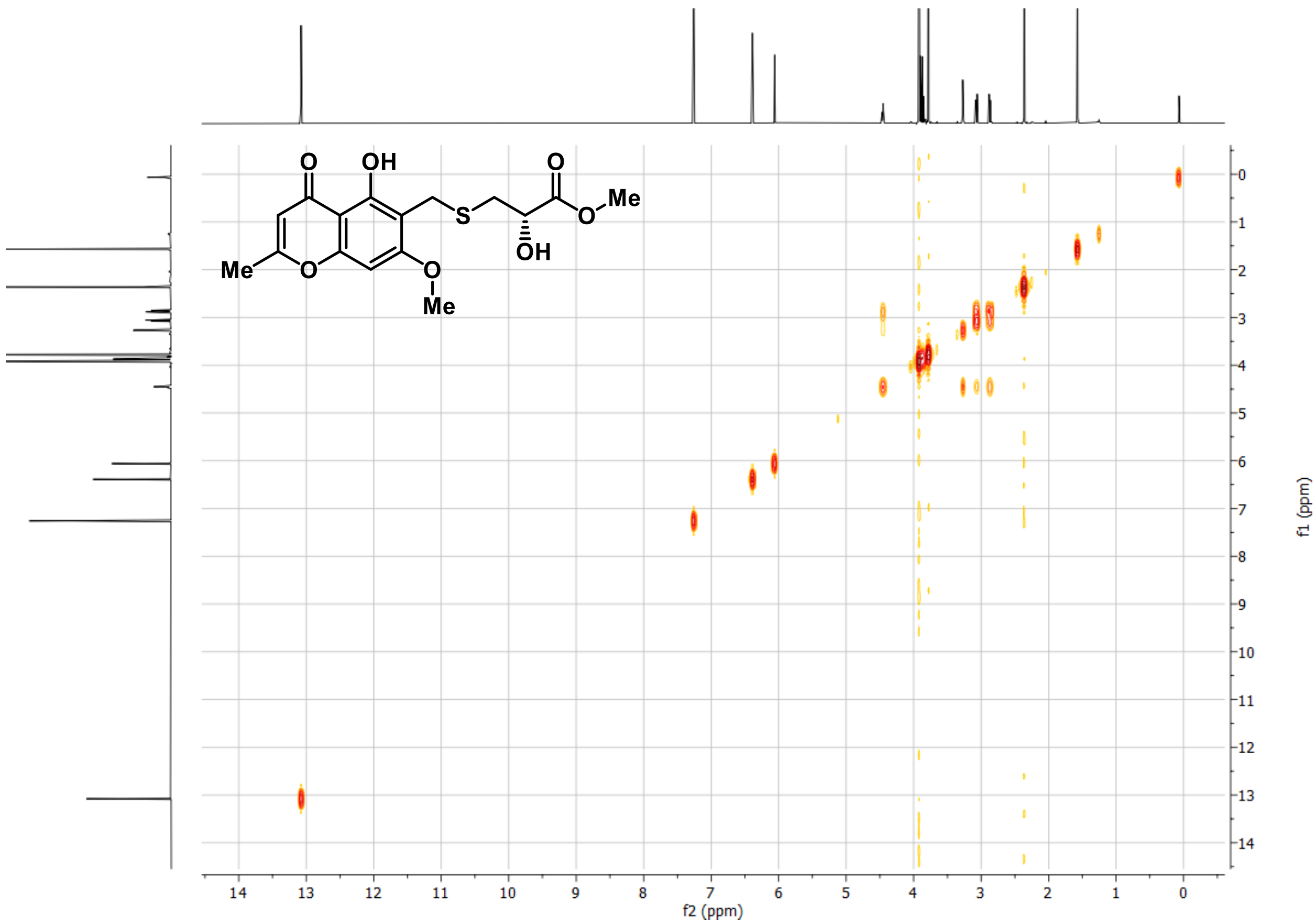

HSQC of chaetosemin B (2, revised):

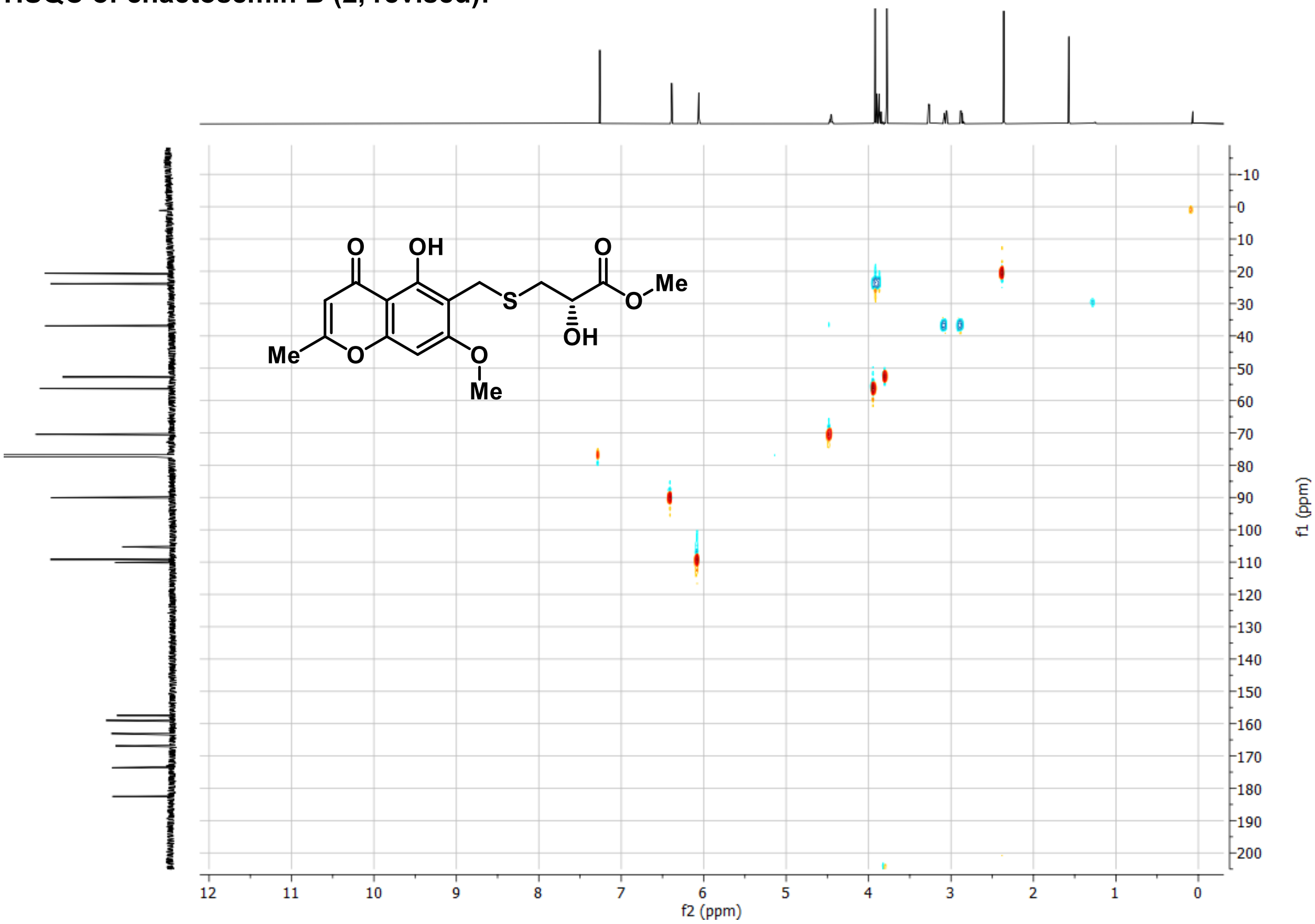

HMBC of chaetosemin B (2, revised):

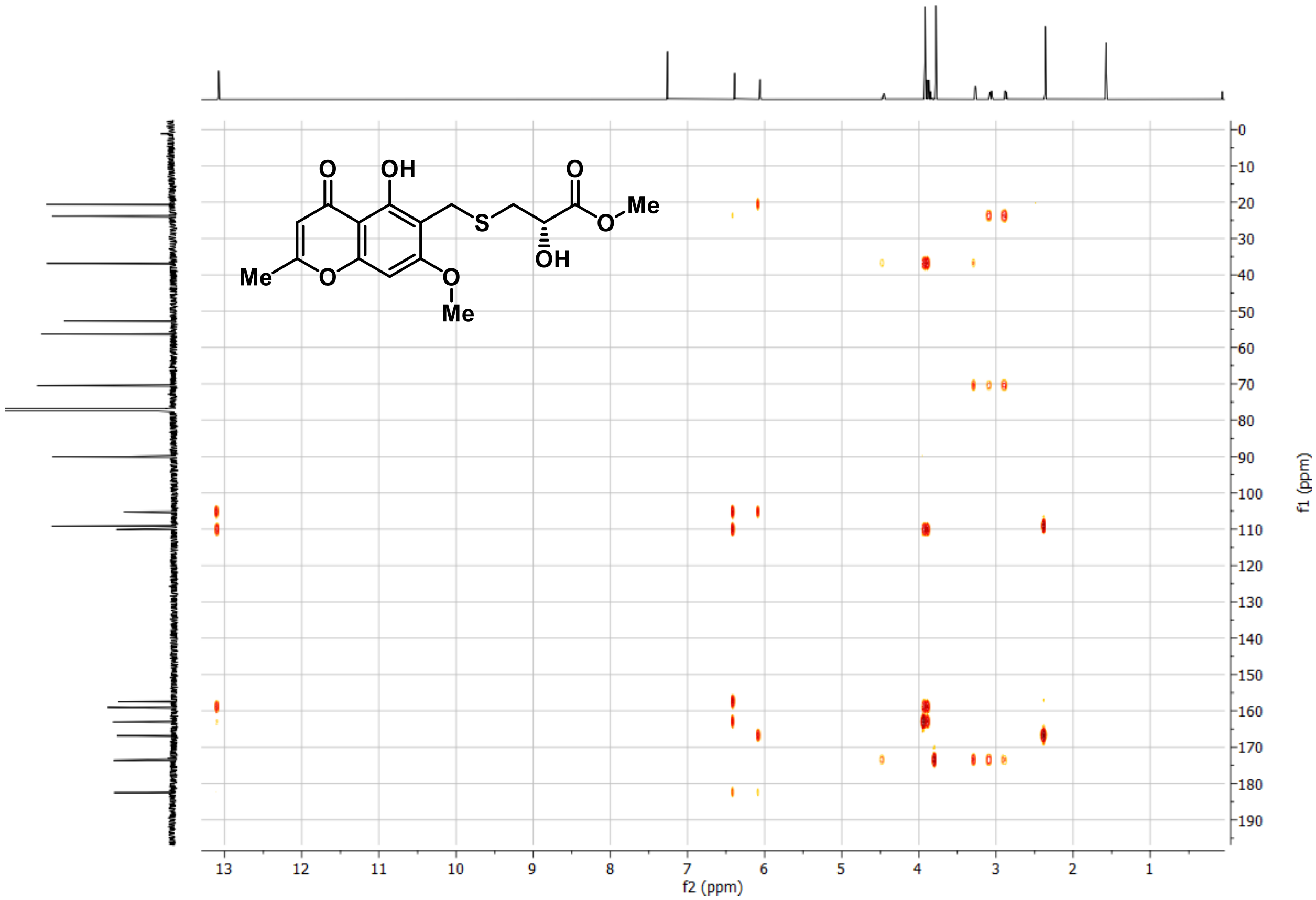

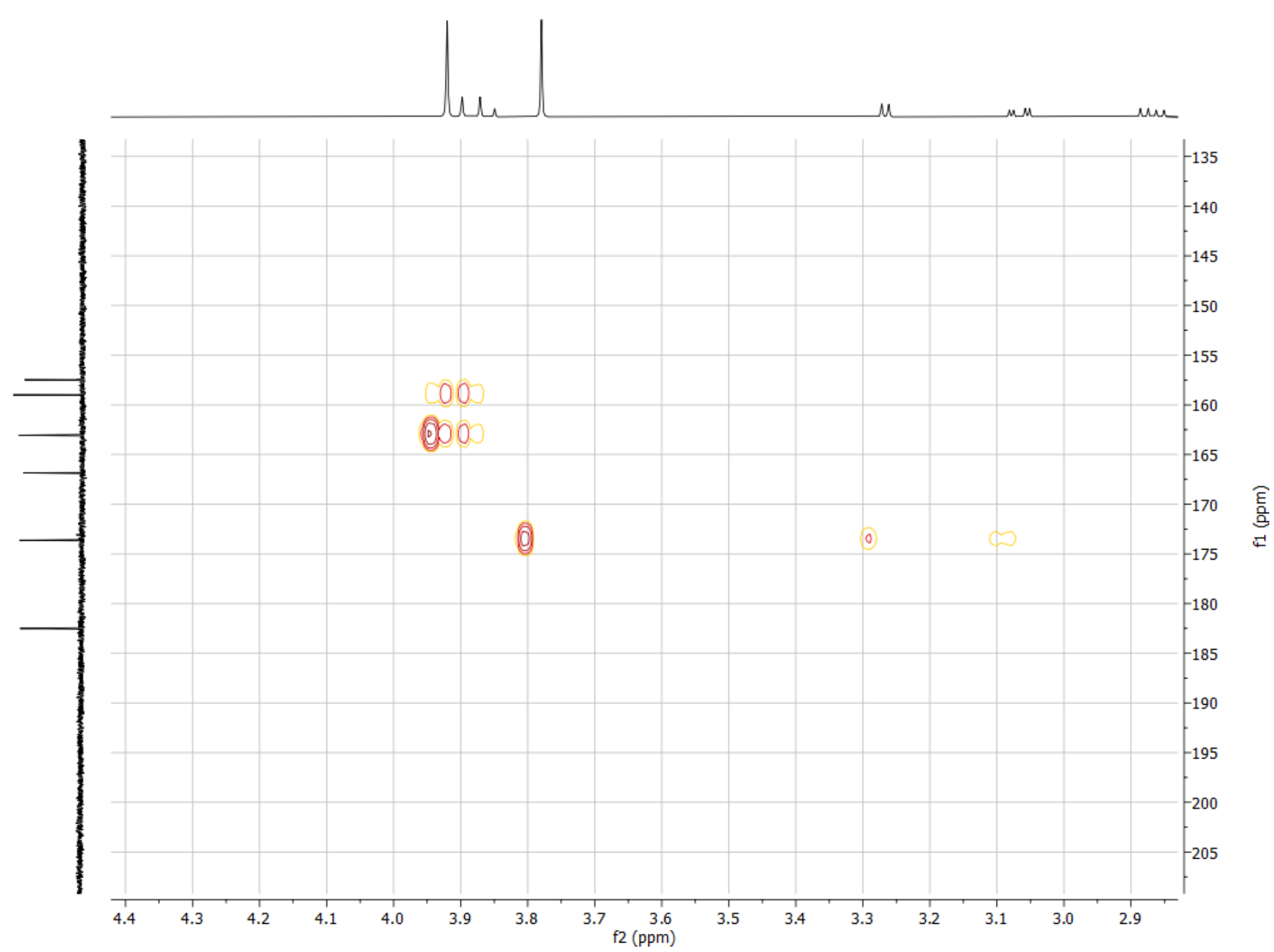

IR (neat, ATR) spectrum of chaetosemin B (2, revised):

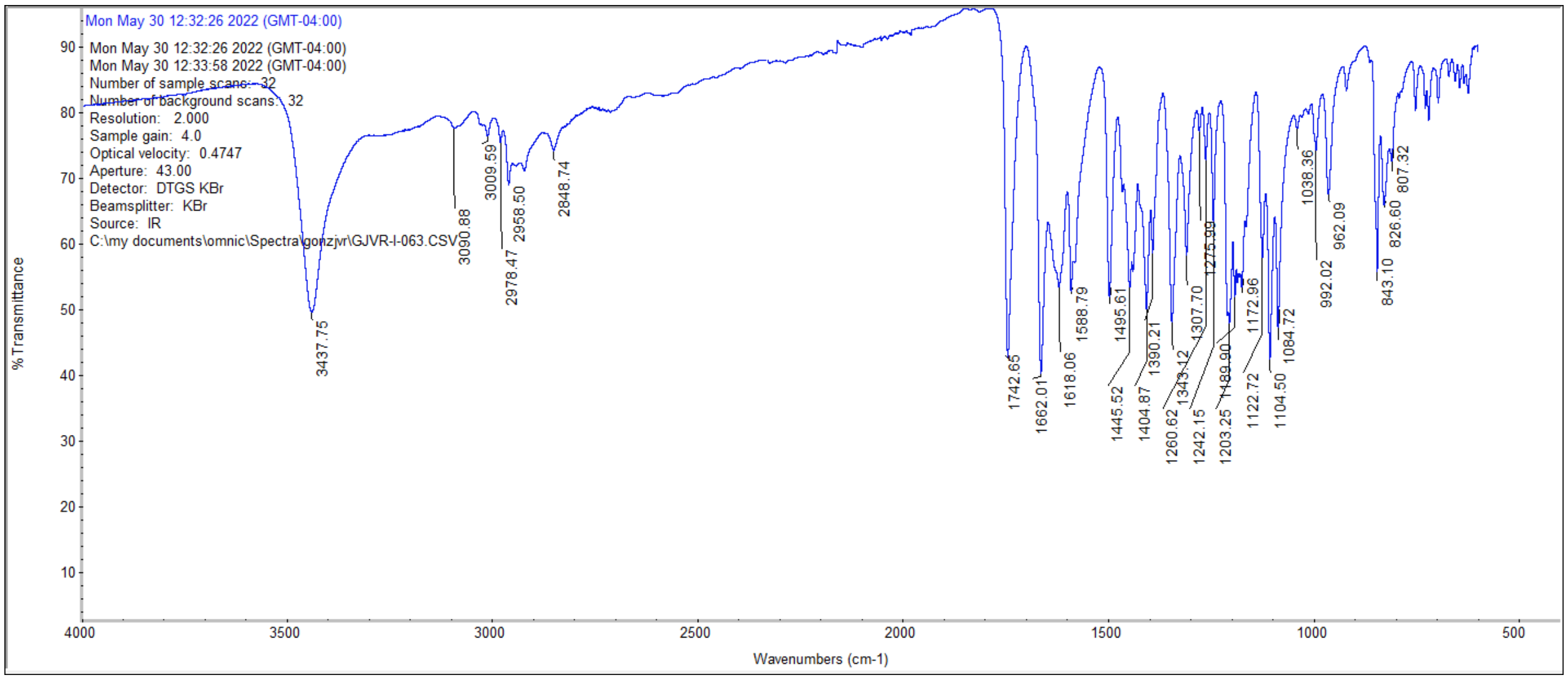

UV-vis spectrum of chaetosemin B (2, revised):

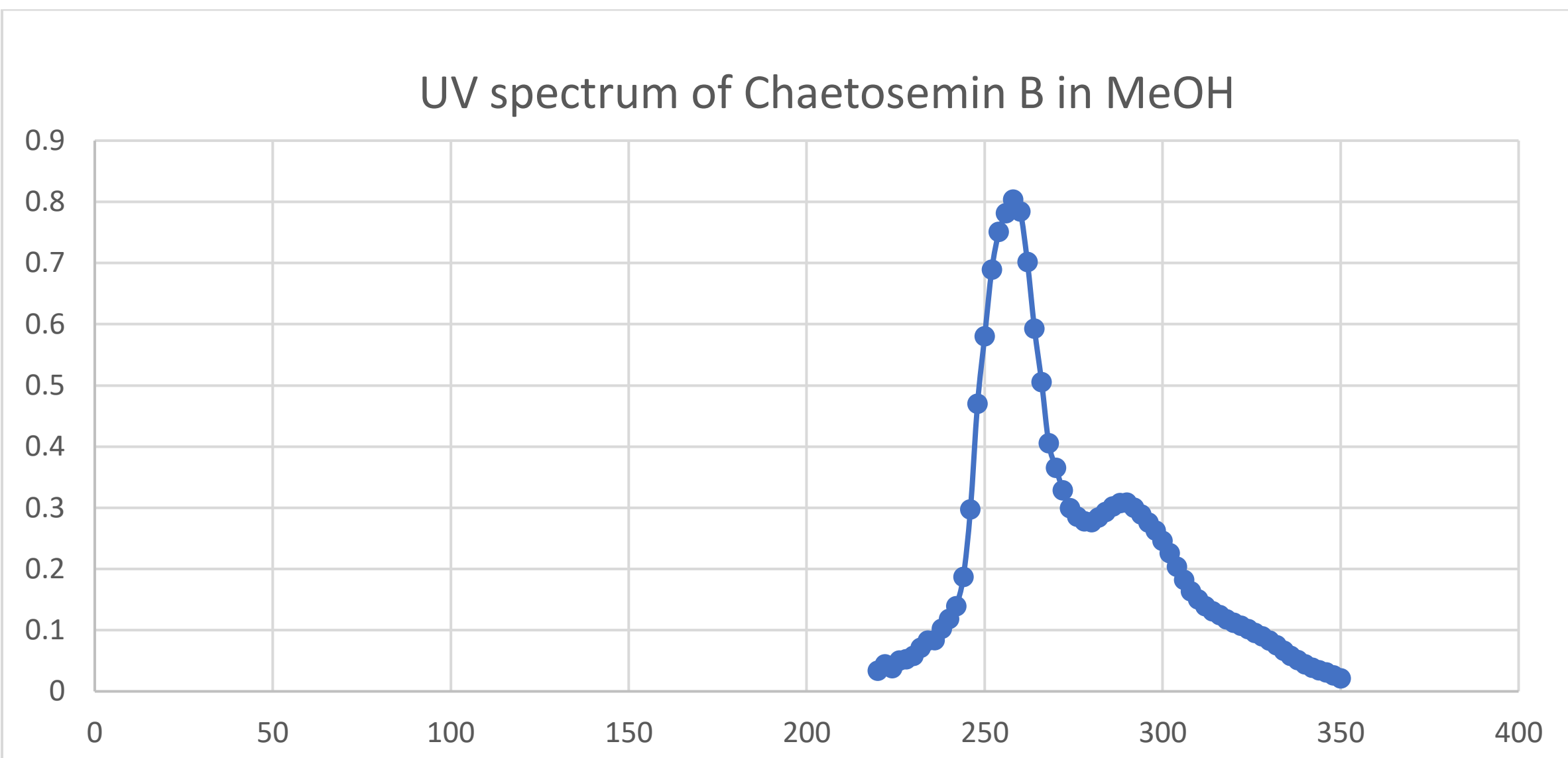

^{1}H-NMR (599 MHz, d_4-MeOH) of chaetosemin A (1):

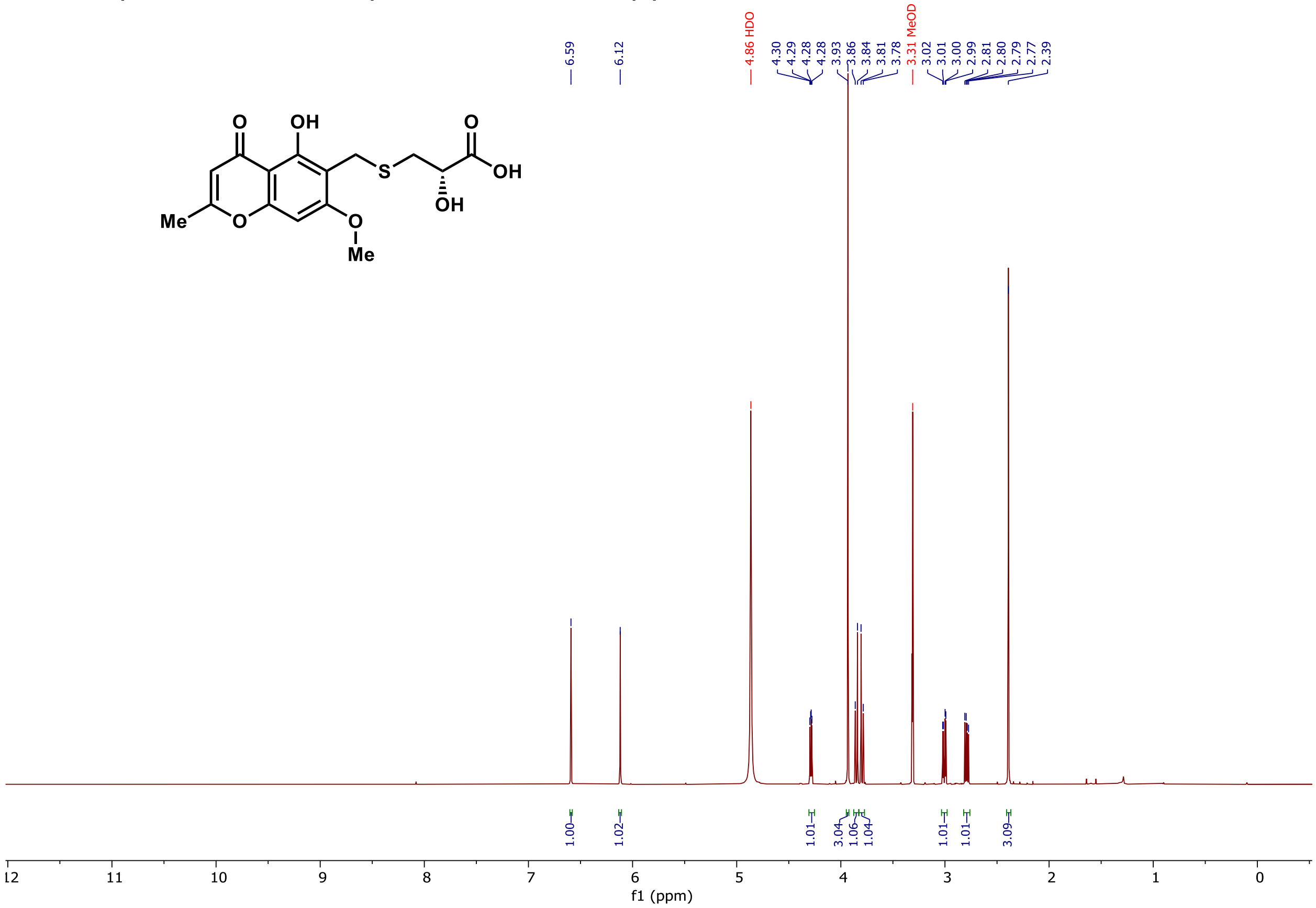

^{13}C-NMR (151 MHz, d_4-MeOH) of chaetosemin A (1):

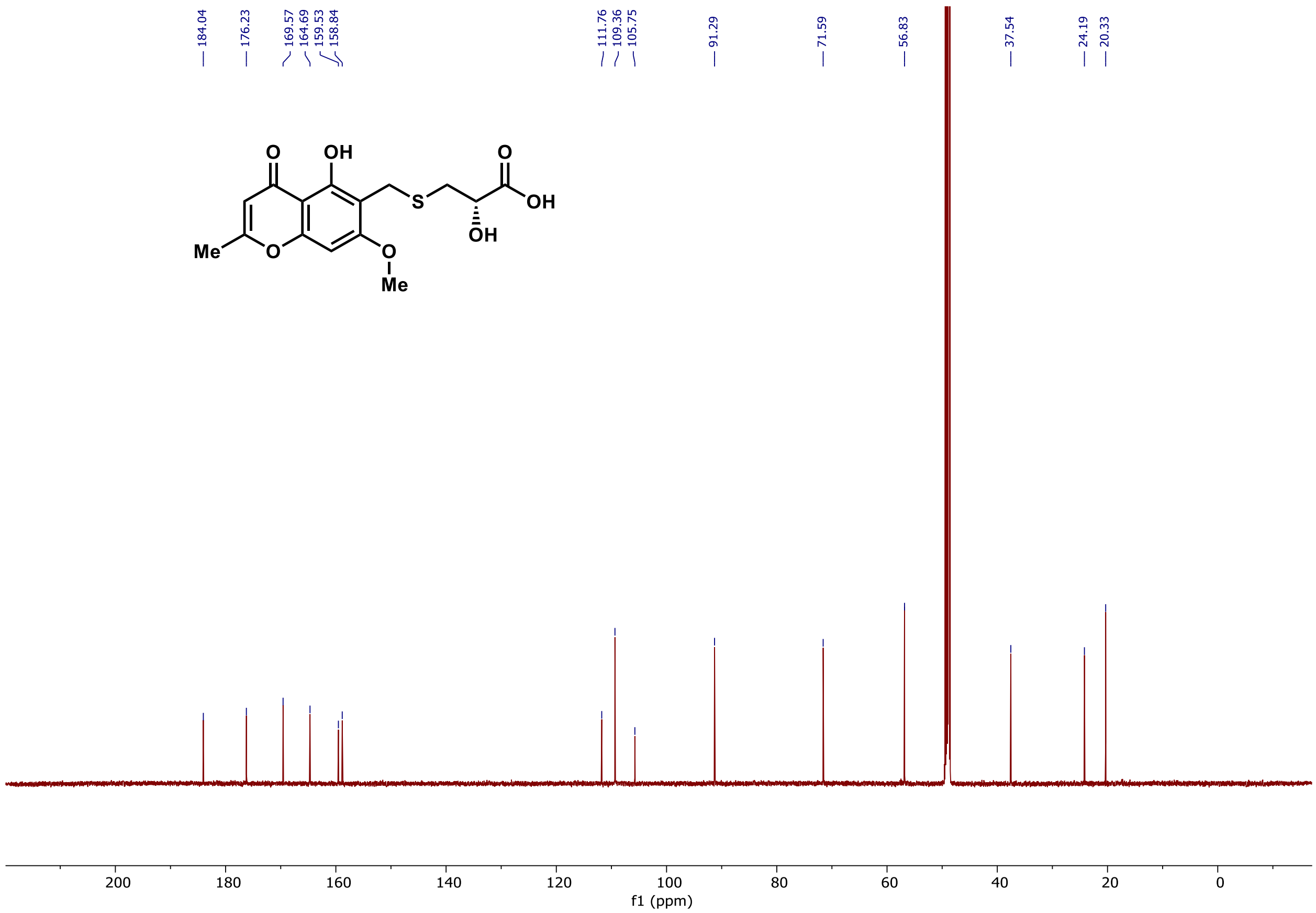

HSQC of chaetosemin A (1):

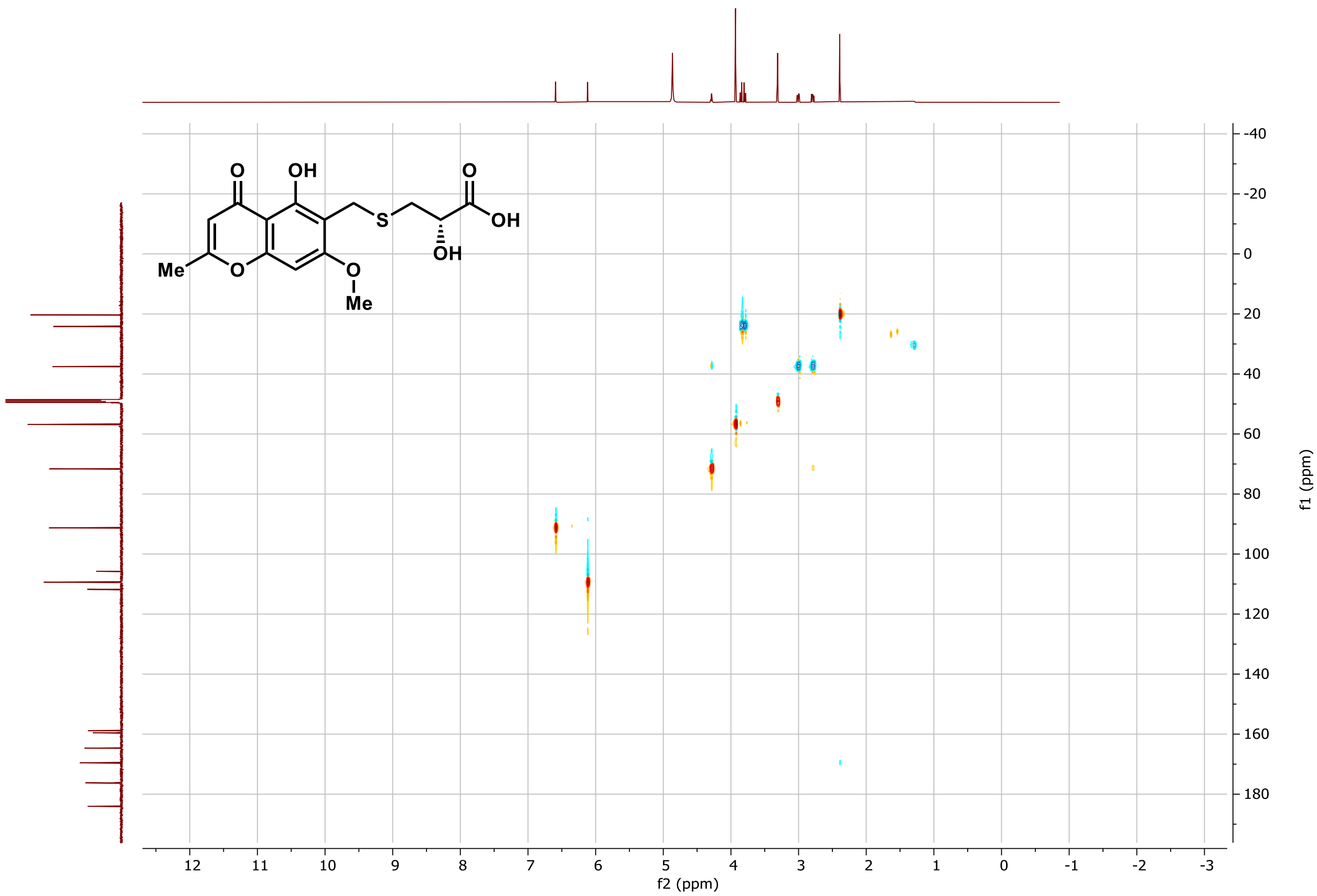

HMBC of chaetosemin A (1):

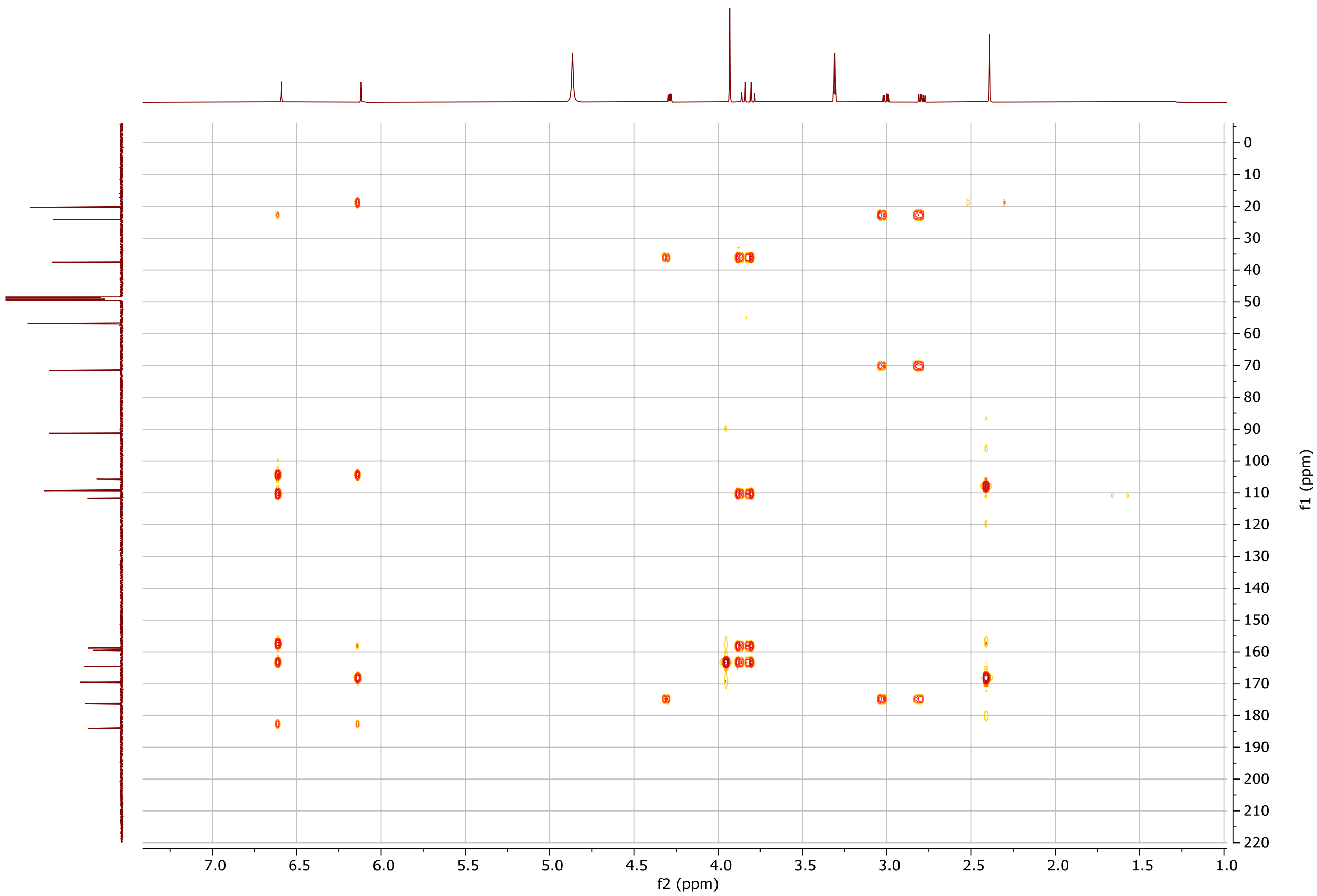

COSY of chaetosemin A (1):

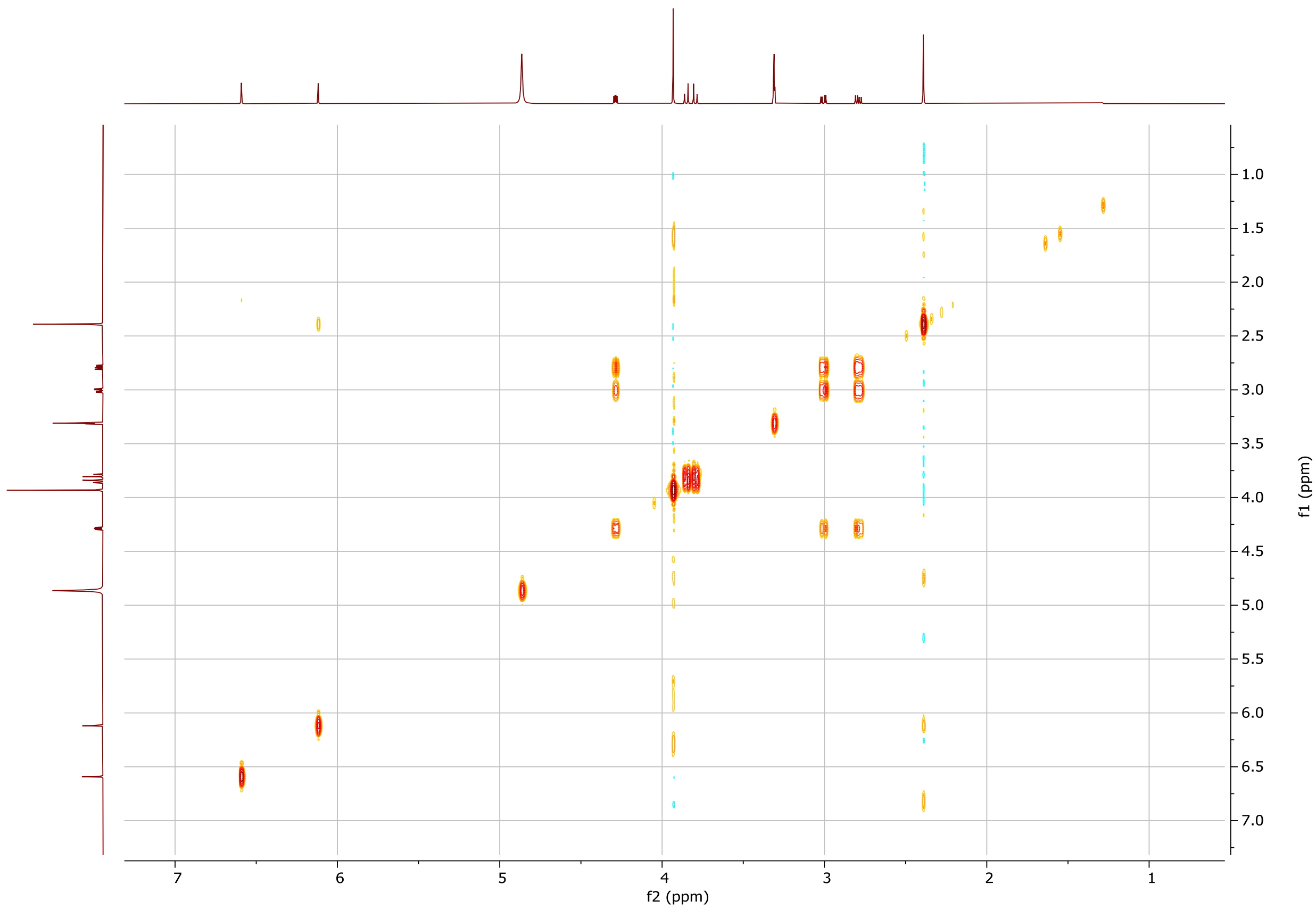

^{1}H-NMR (599 MHz, d_4-MeOH) of (*ent*)-chaetosemin A (S17):

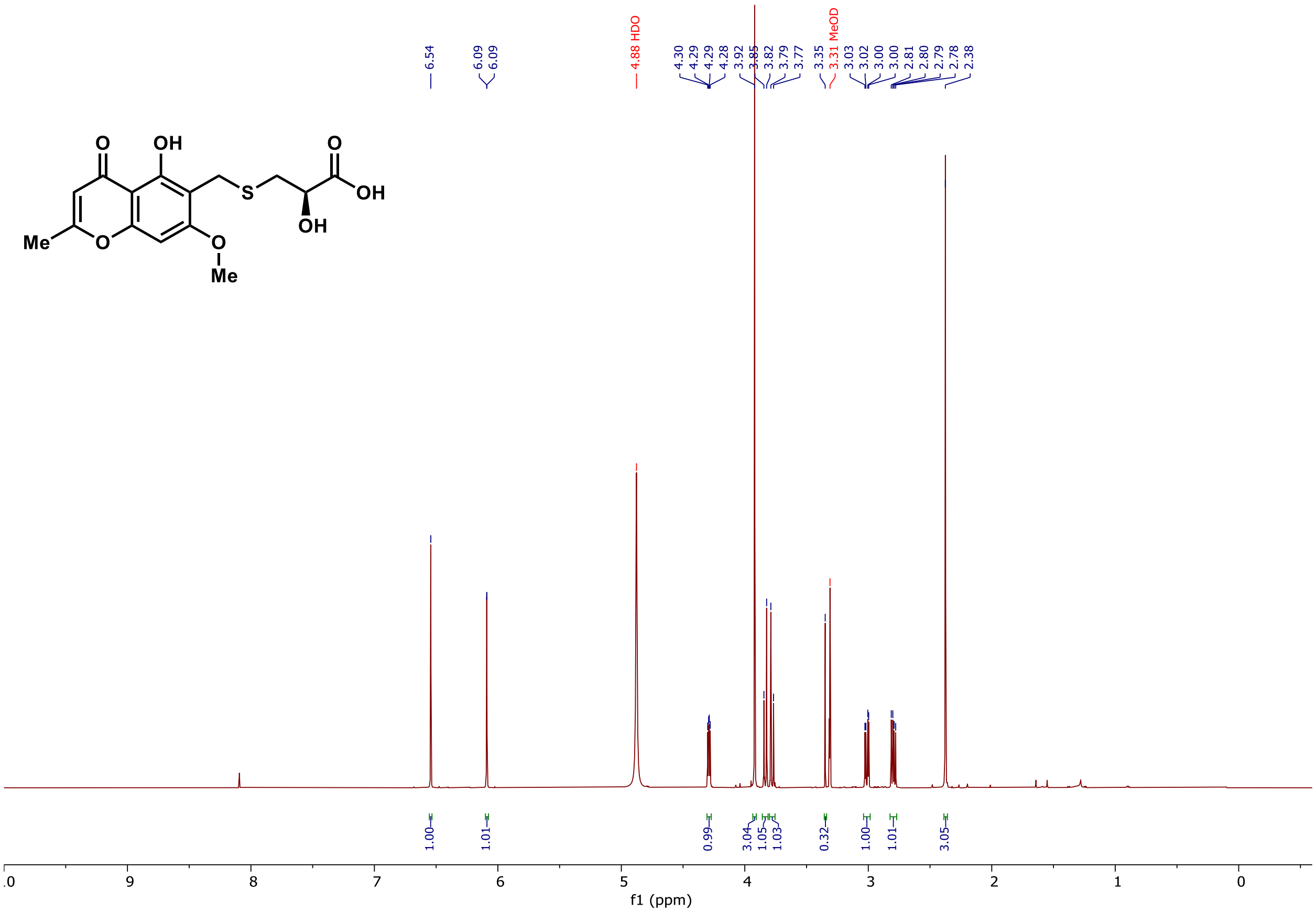

^{13}C-NMR (151 MHz, d_4-MeOH) of (*ent*)-chaetosemin A (S17)

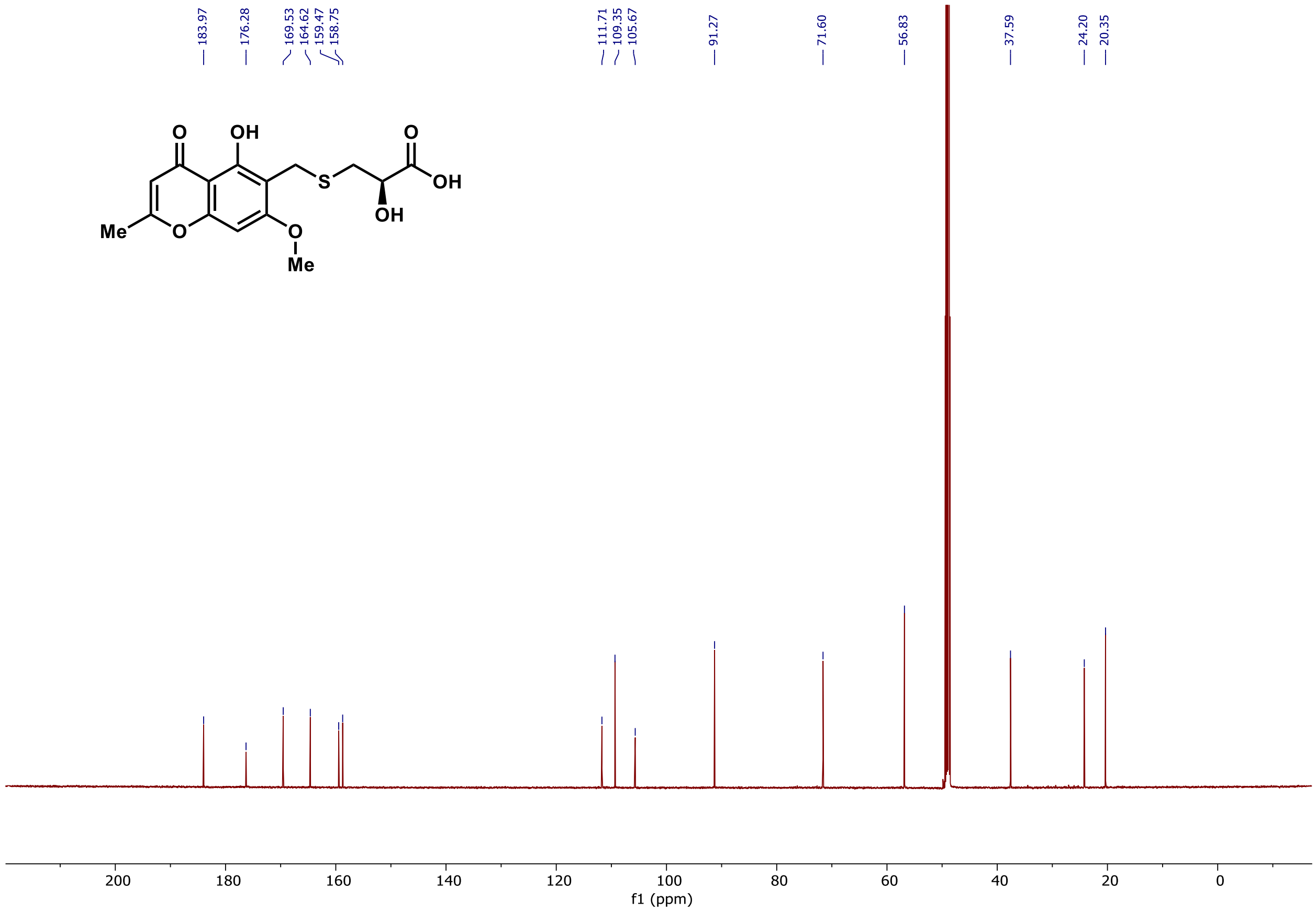

^{1}H-NMR (599 MHz, $CDCl_3$) of 2-(bromomethyl)isoindoline-1,3-dione (S18):

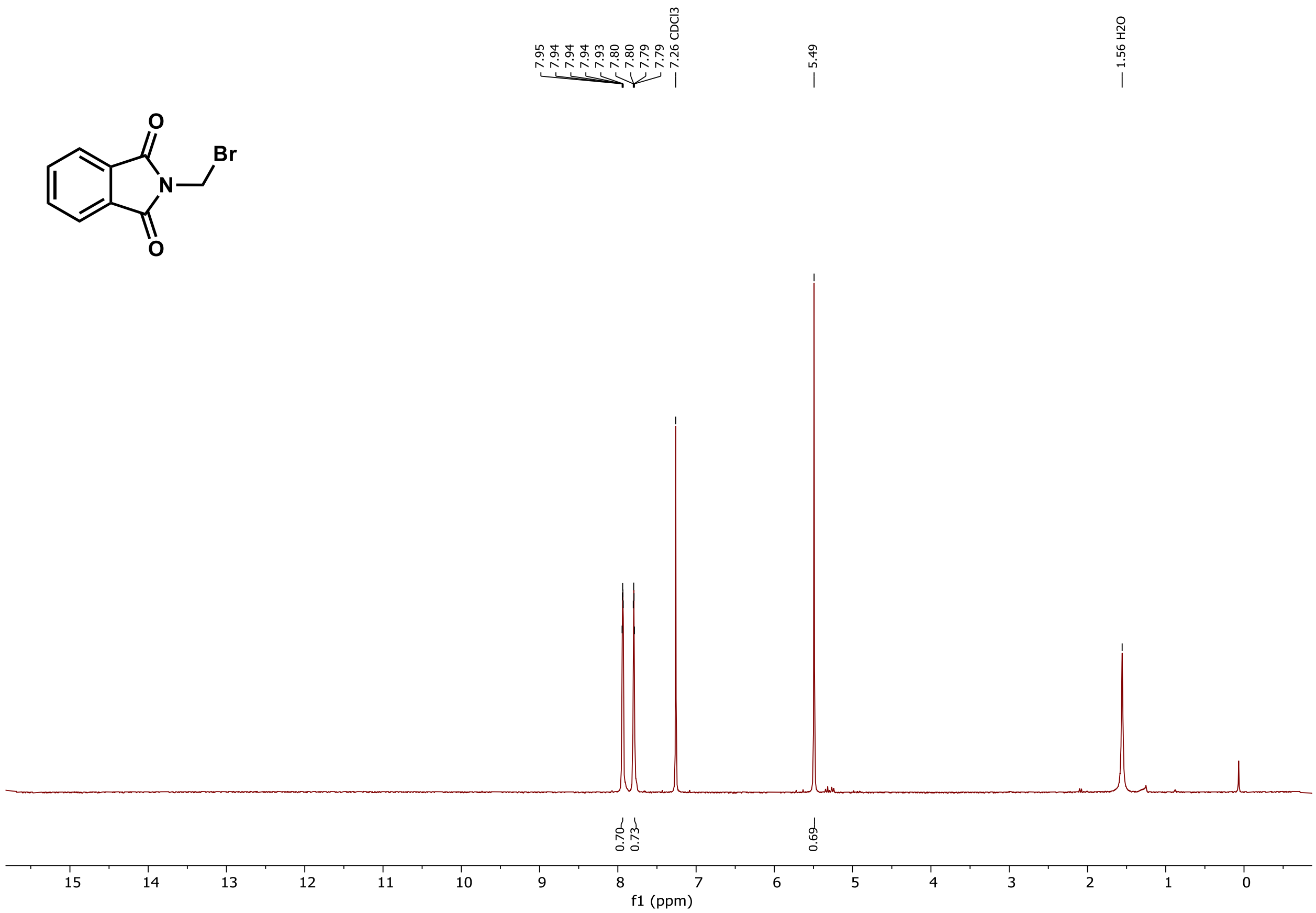

^{1}H-NMR (599 MHz, $CDCl_3$) of *N*-(2-(((1,3-dioxoisoindolin-2-yl)methyl)thio)ethyl)acetamide (S19):

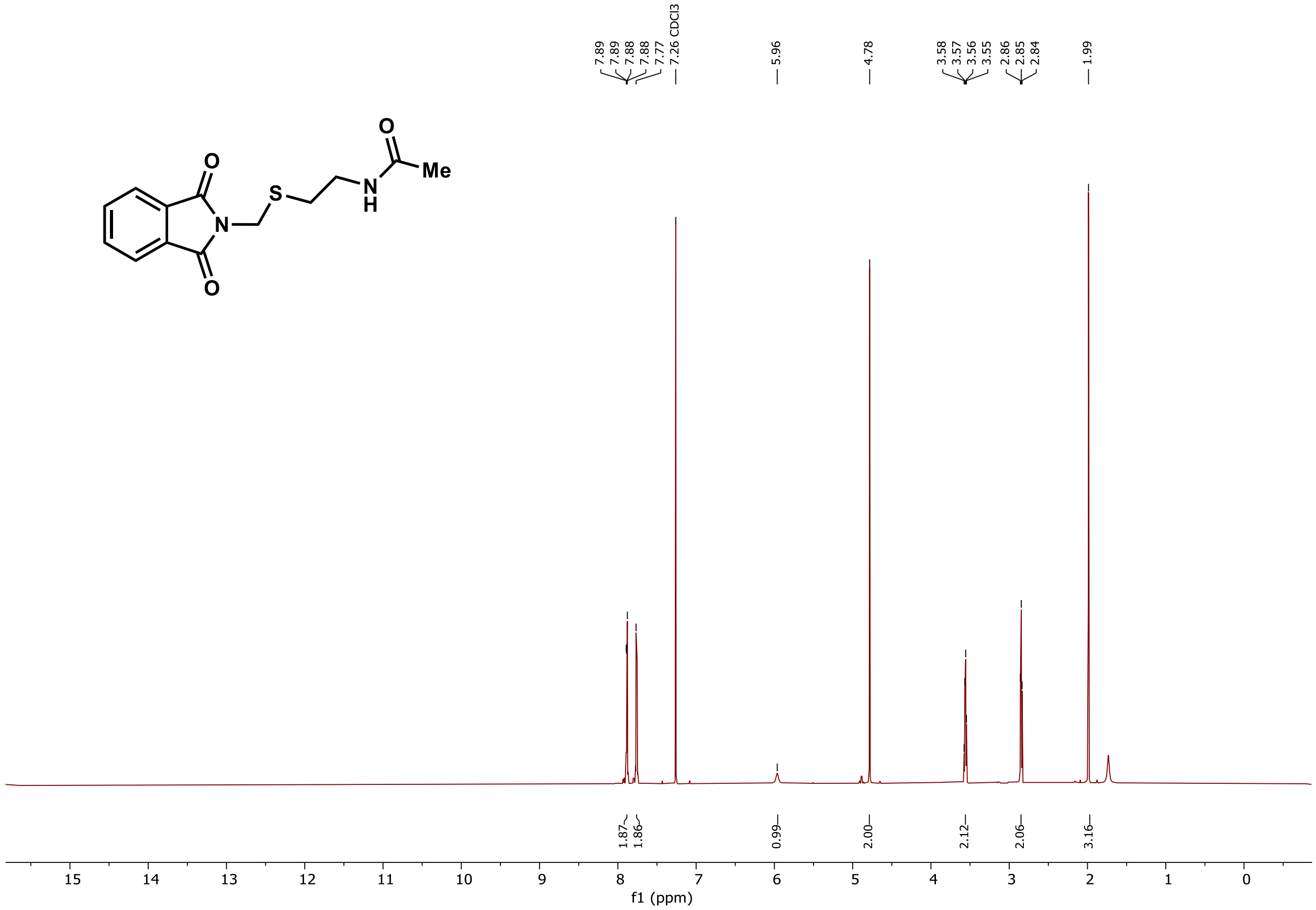

^{13}C-NMR (151 MHz, $CDCl_3$) of *N*-(2-(((1,3-dioxoisoindolin-2-yl)methyl)thio)ethyl)acetamide (S19):

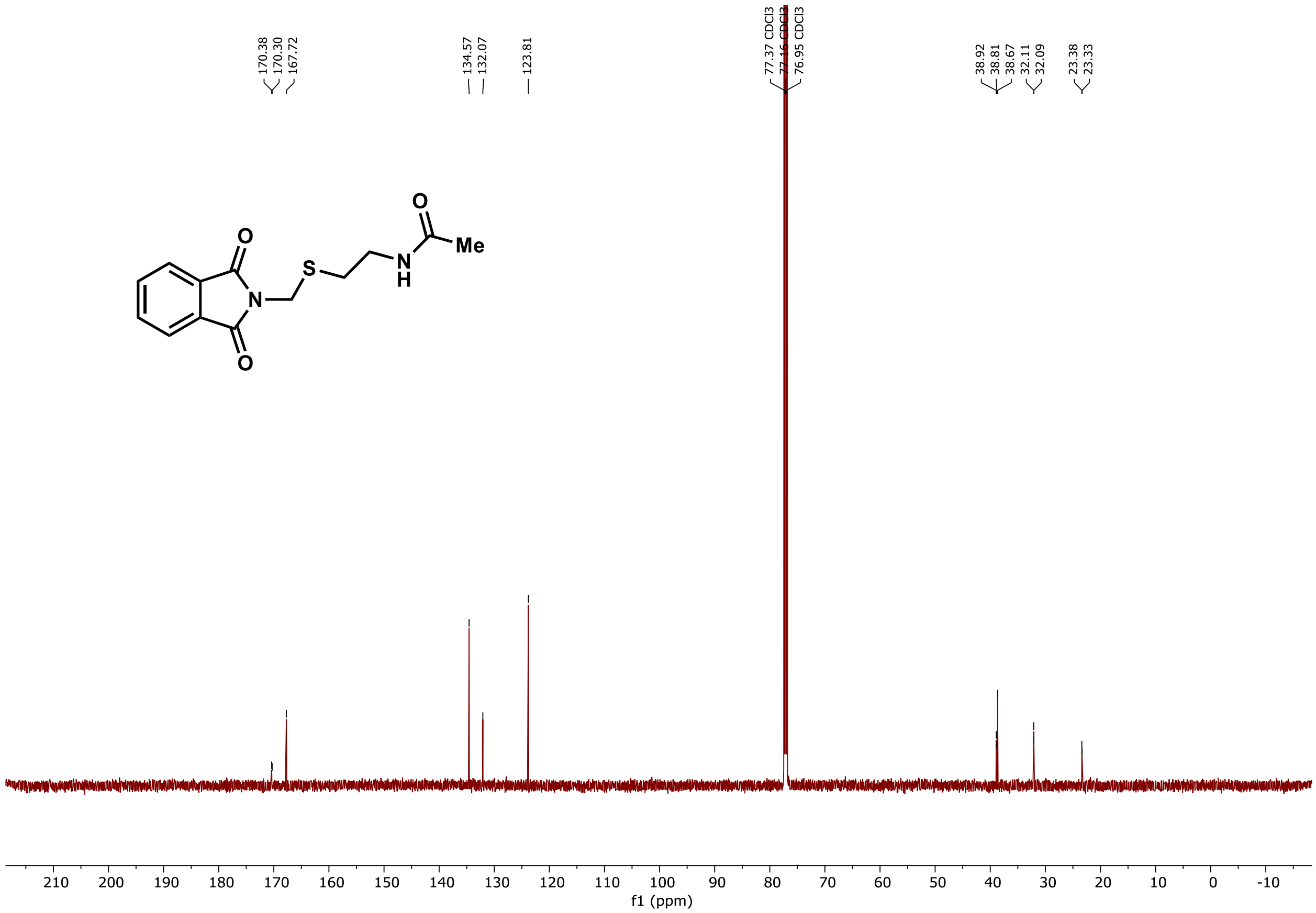

^{1}H-NMR (599 MHz, CDCl$_3$) of *N*-(2-(((1,3-dioxoisoindolin-2-yl)methyl)sulfonyl)ethyl)acetamide (S20):

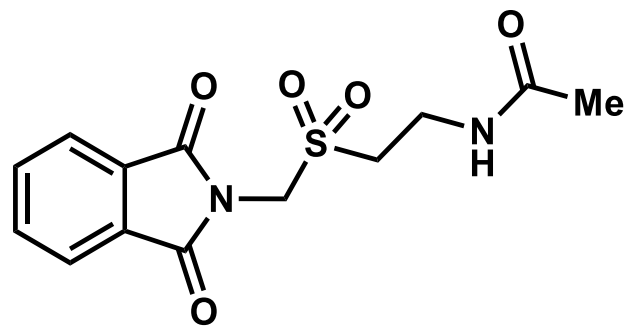

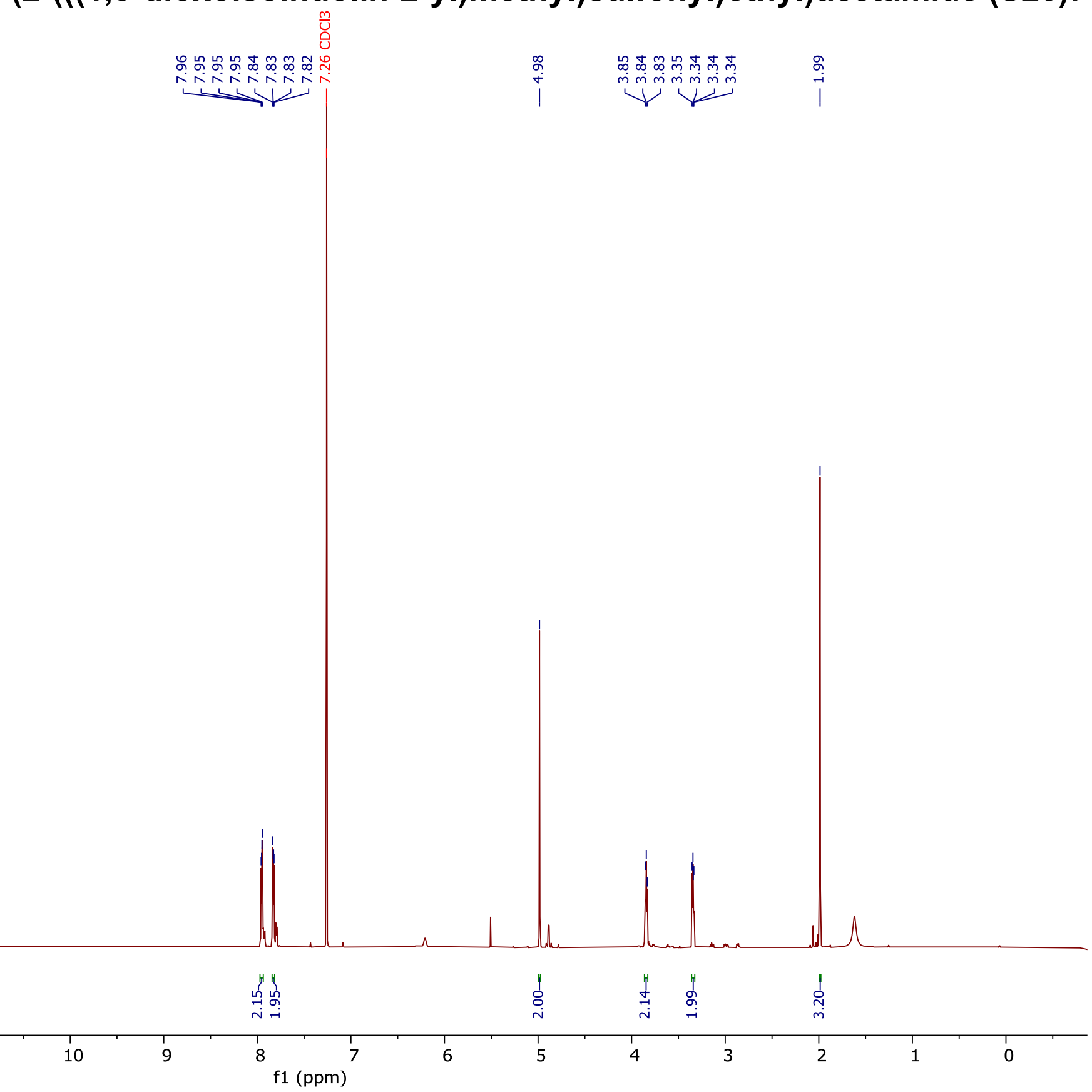

^{13}C-NMR (151 MHz, $CDCl_3$) of *N*-(2-(((1,3-dioxoisoindolin-2-yl)methyl)sulfonyl)ethyl)acetamide (S20):

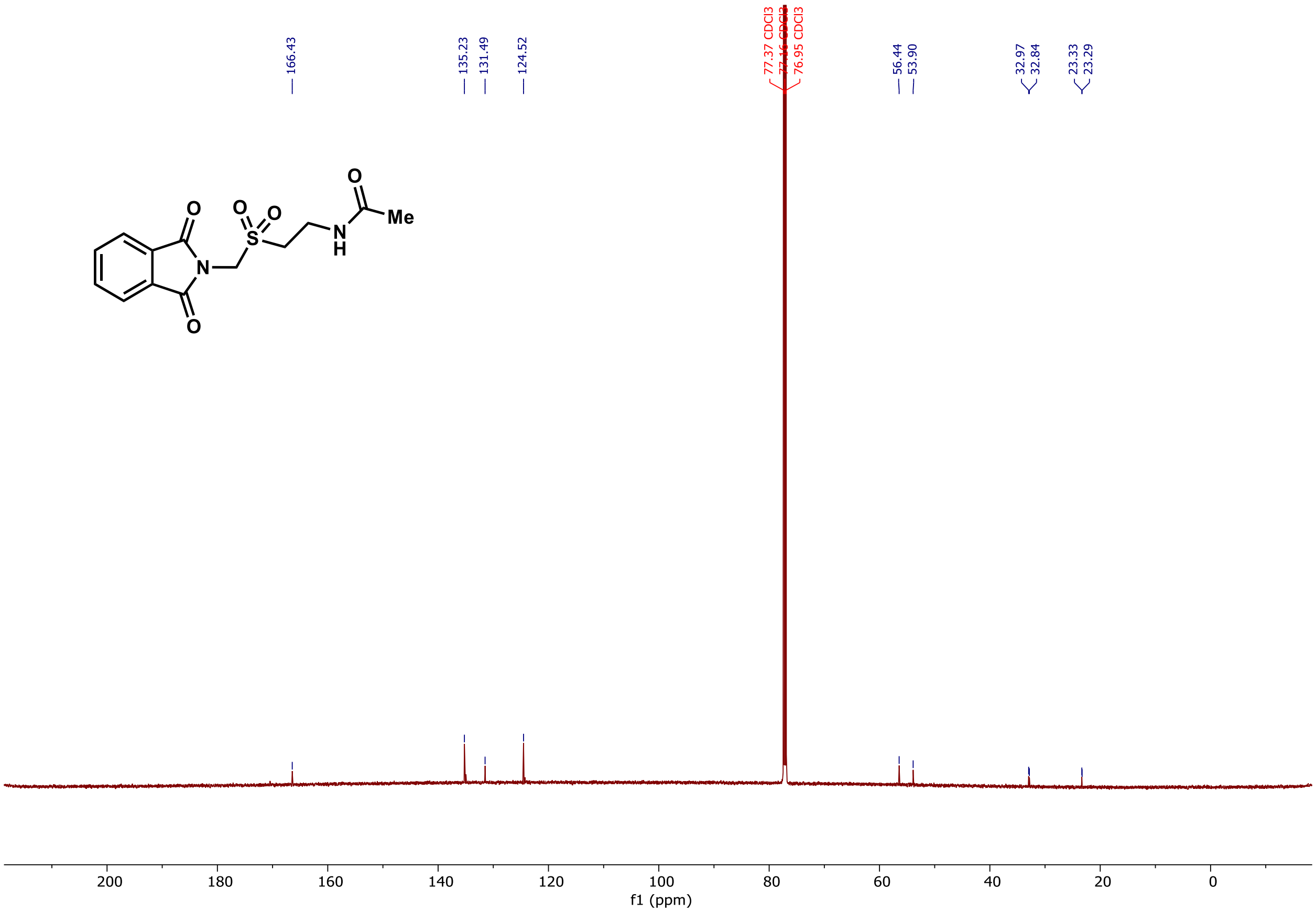

^{1}H-NMR (599 MHz, d_4-MeOH) of sodium 2-acetamidoethane-1-sulfinate (17):

.

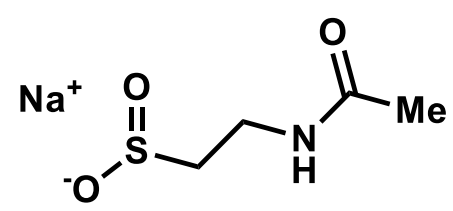

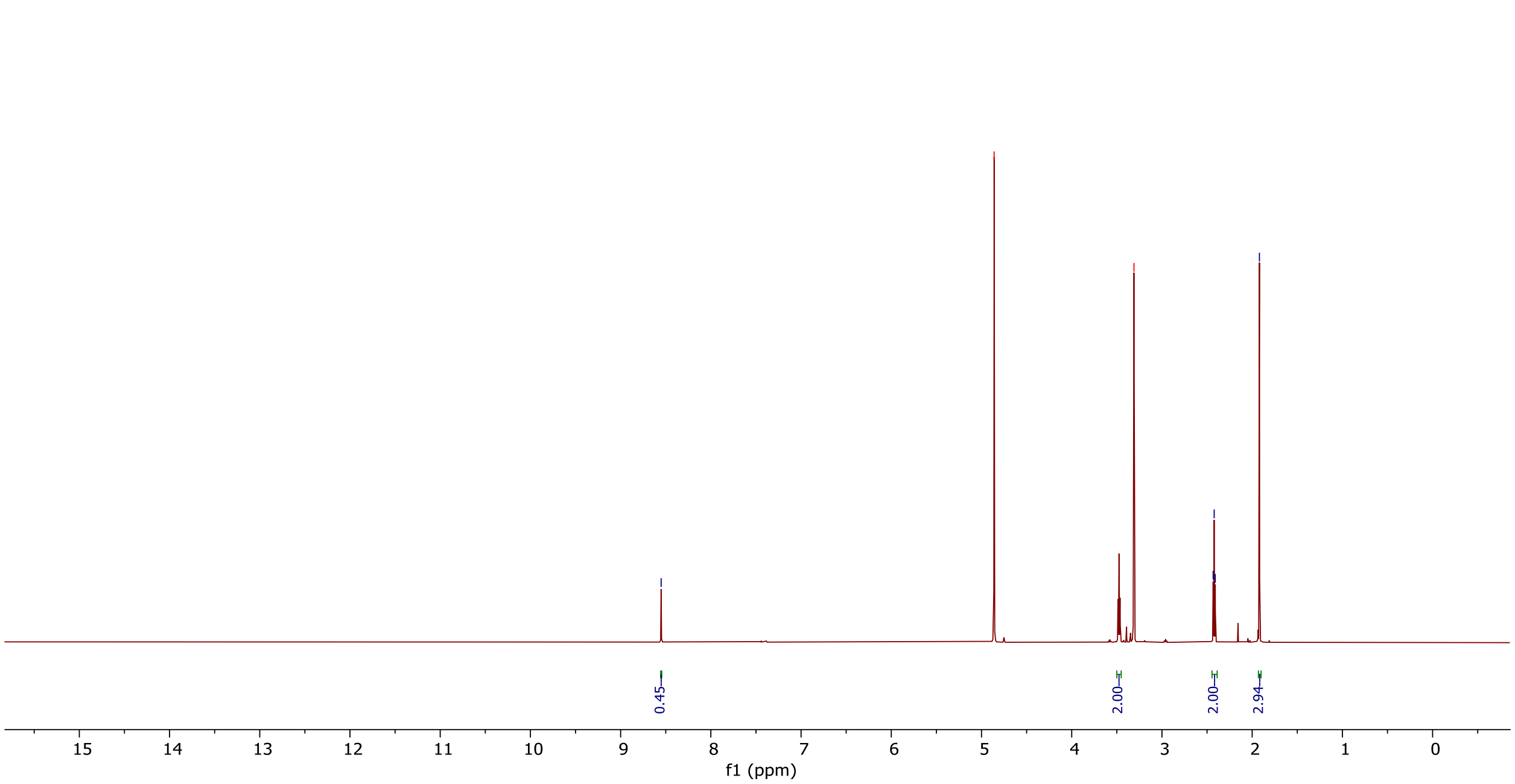

^{13}C-NMR (151 MHz, d_4-MeOH) of Sodium 2-acetamidoethane-1-sulfinate (17):

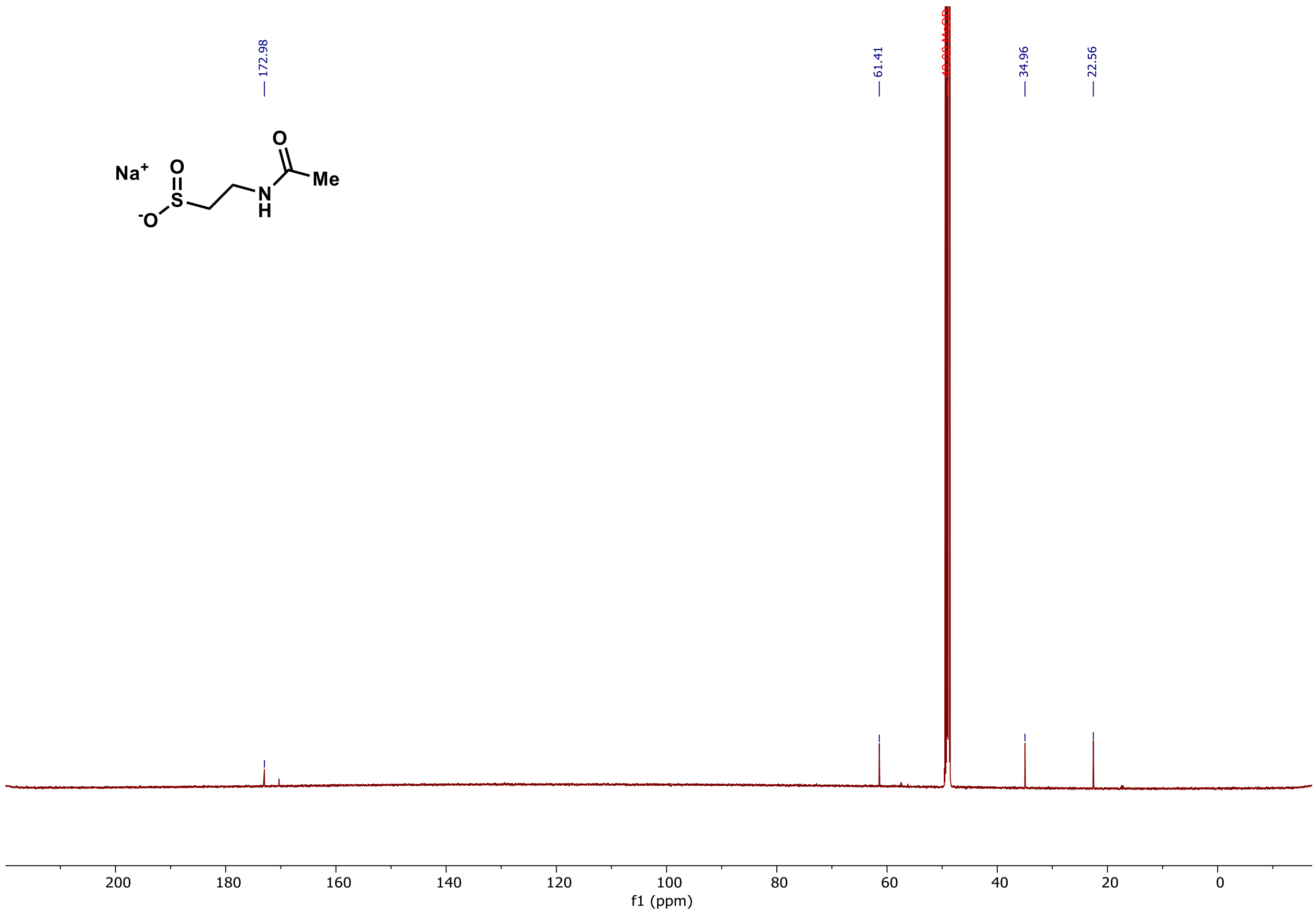

^{1}H-NMR (599 MHz, $CDCl_3$) of chaetoquadrin D (3):

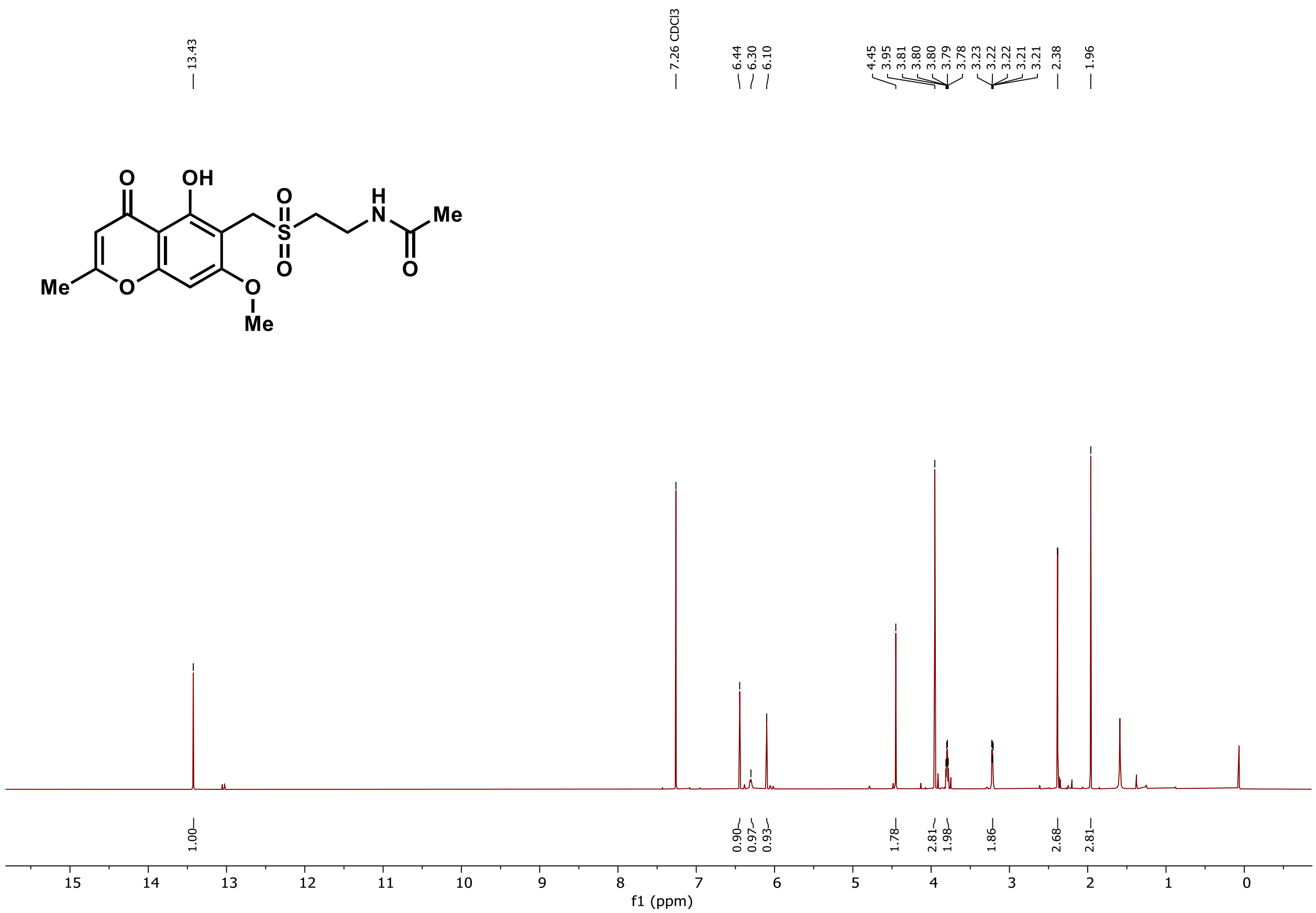

^{13}C-NMR (151 MHz, $CDCl_3$) of chaetoquadrin D (3):

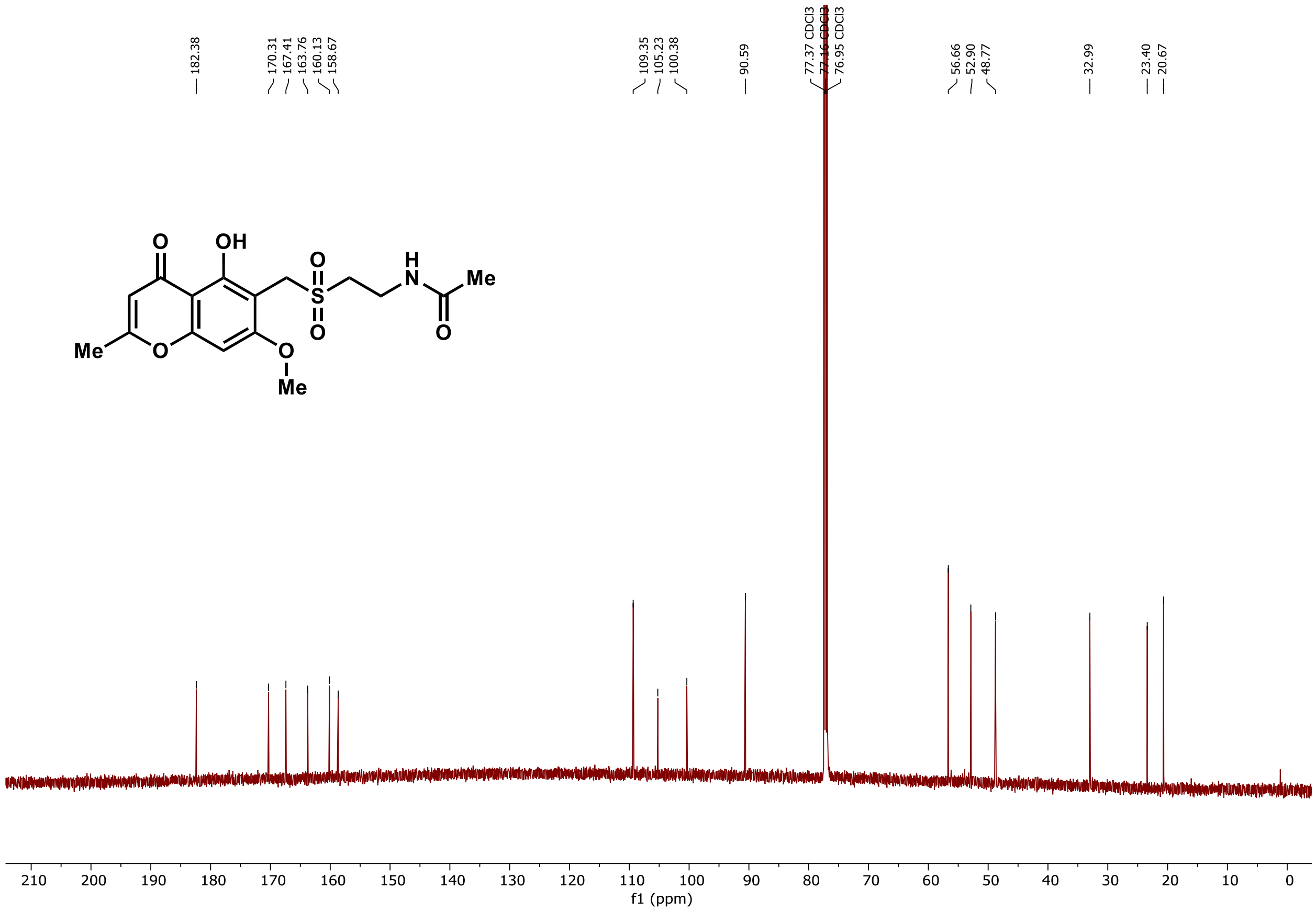

COSY of chaetoquadrin D (3):

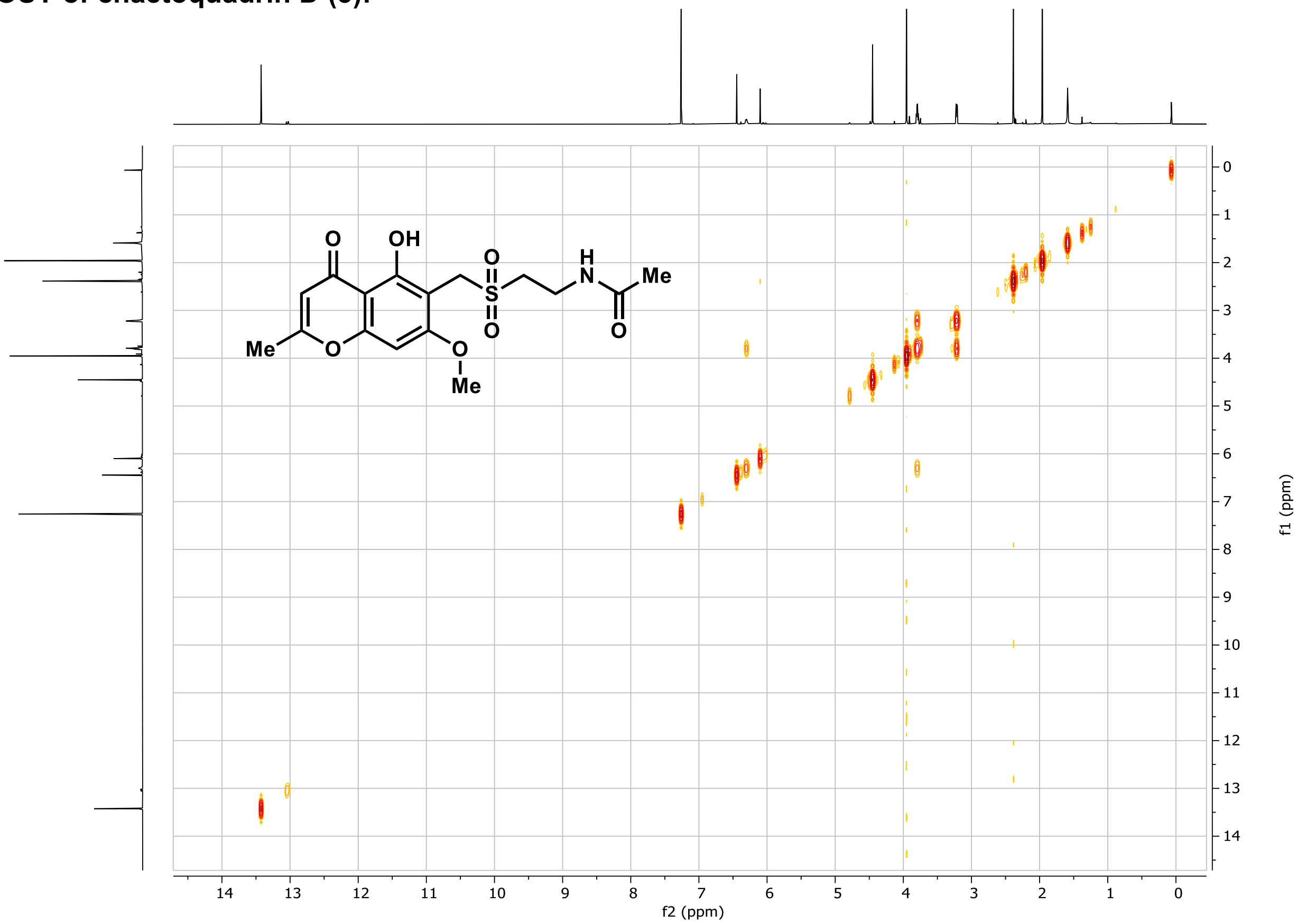

HSQC of chaetoquadrin D (3):

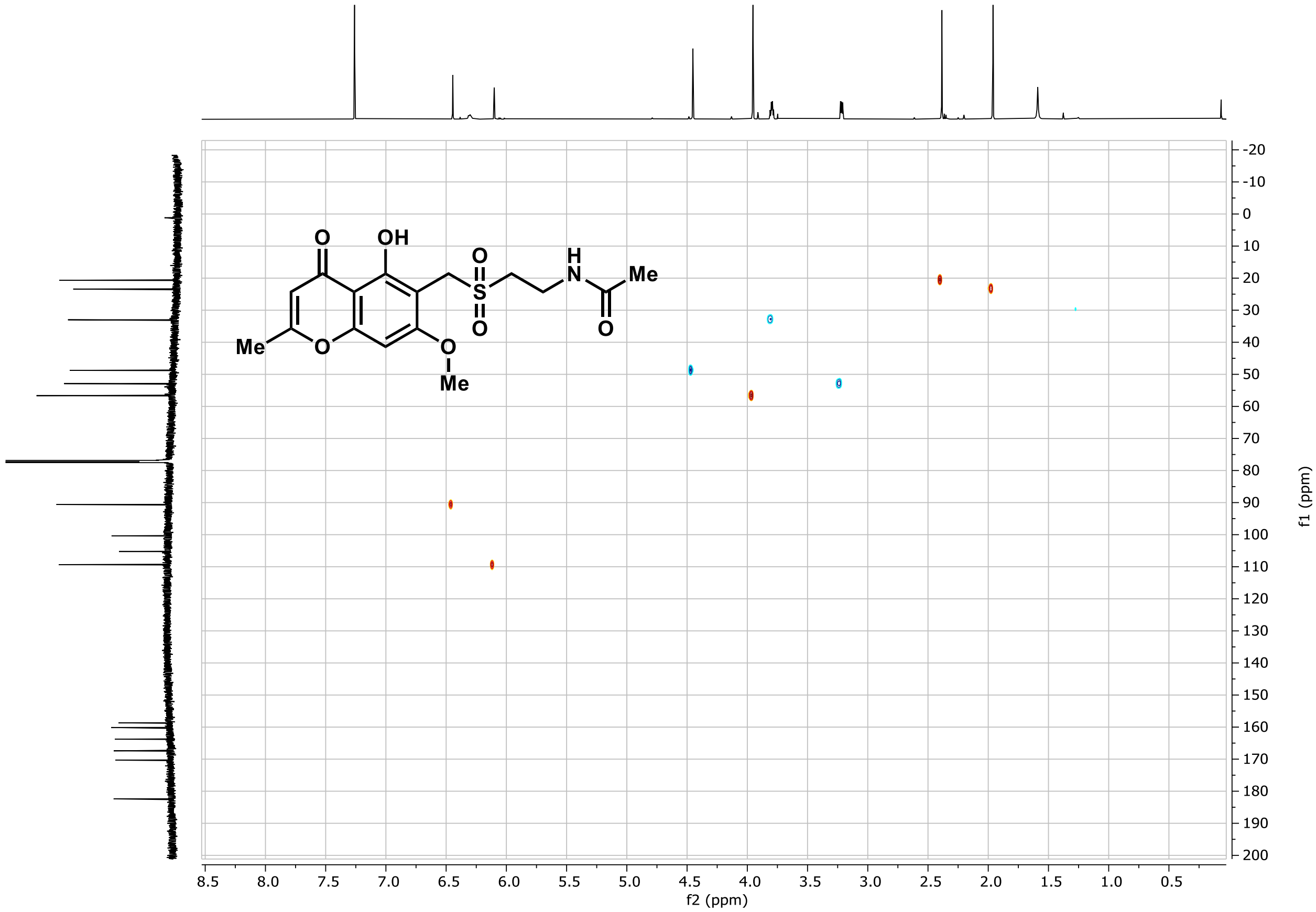

HMBC of chaetoquadrin D (3):

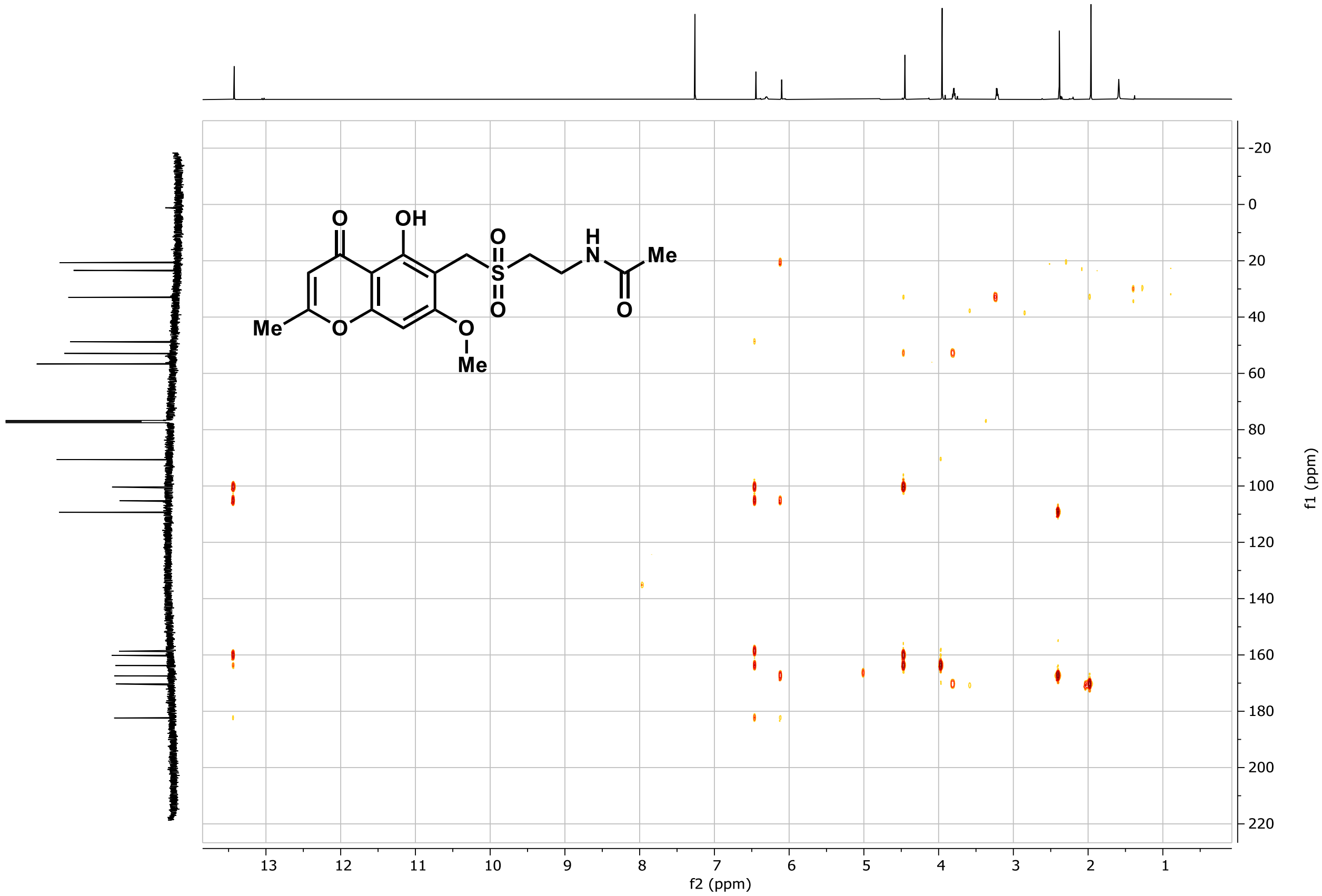

^{1}H-NMR (599 MHz, d_6-DMSO) of 4,6-dihydroxy-2,3-dimethylbenzaldehyde (8):

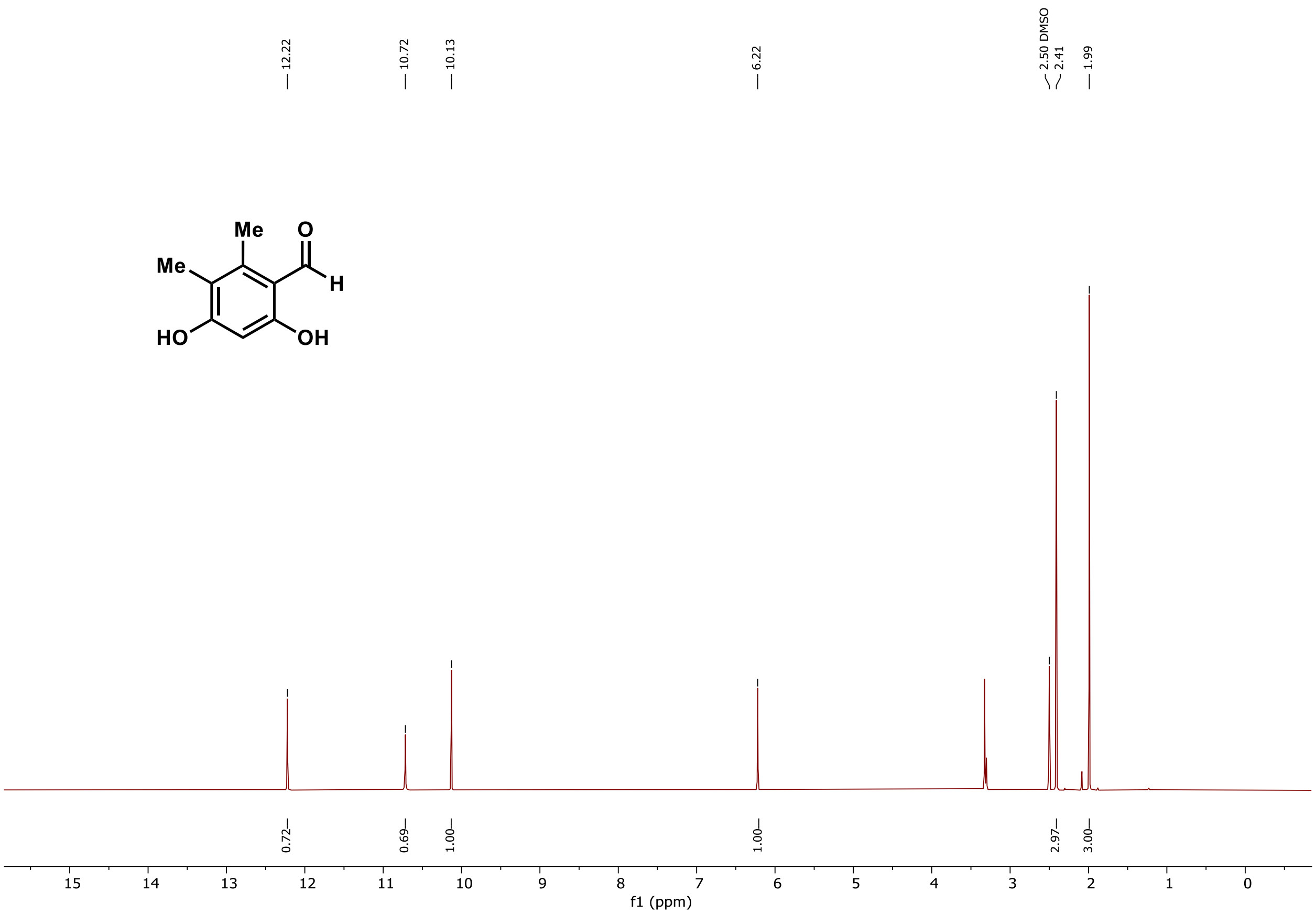

^{1}H-NMR (599 MHz, d$_4$-MeOH) of 4,6-dihydroxy-2,3-dimethylbenzaldehyde (8):

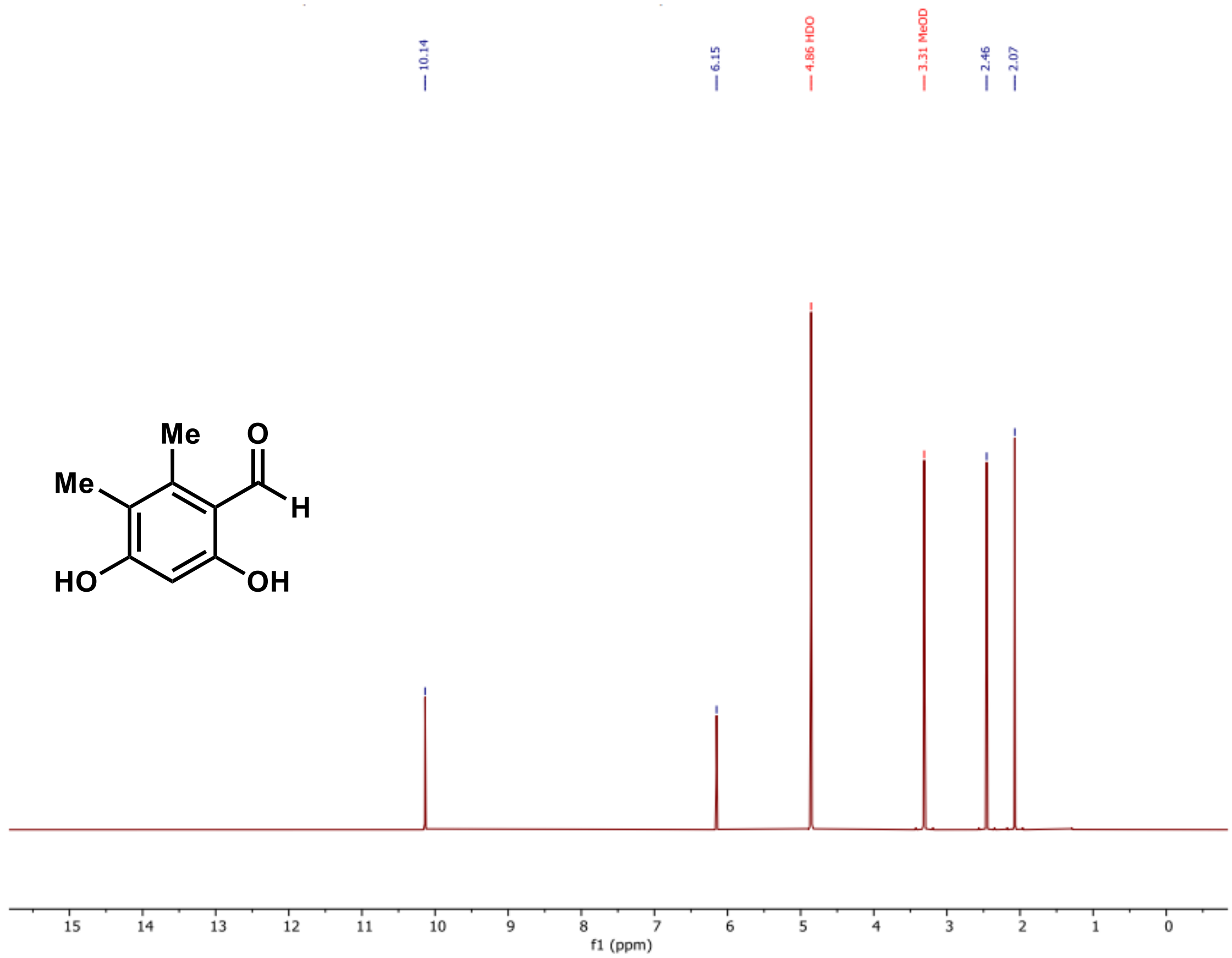

^{13}C-NMR (150 MHz, d_4-MeOH) of 4,6-dihydroxy-2,3-dimethylbenzaldehyde (8):

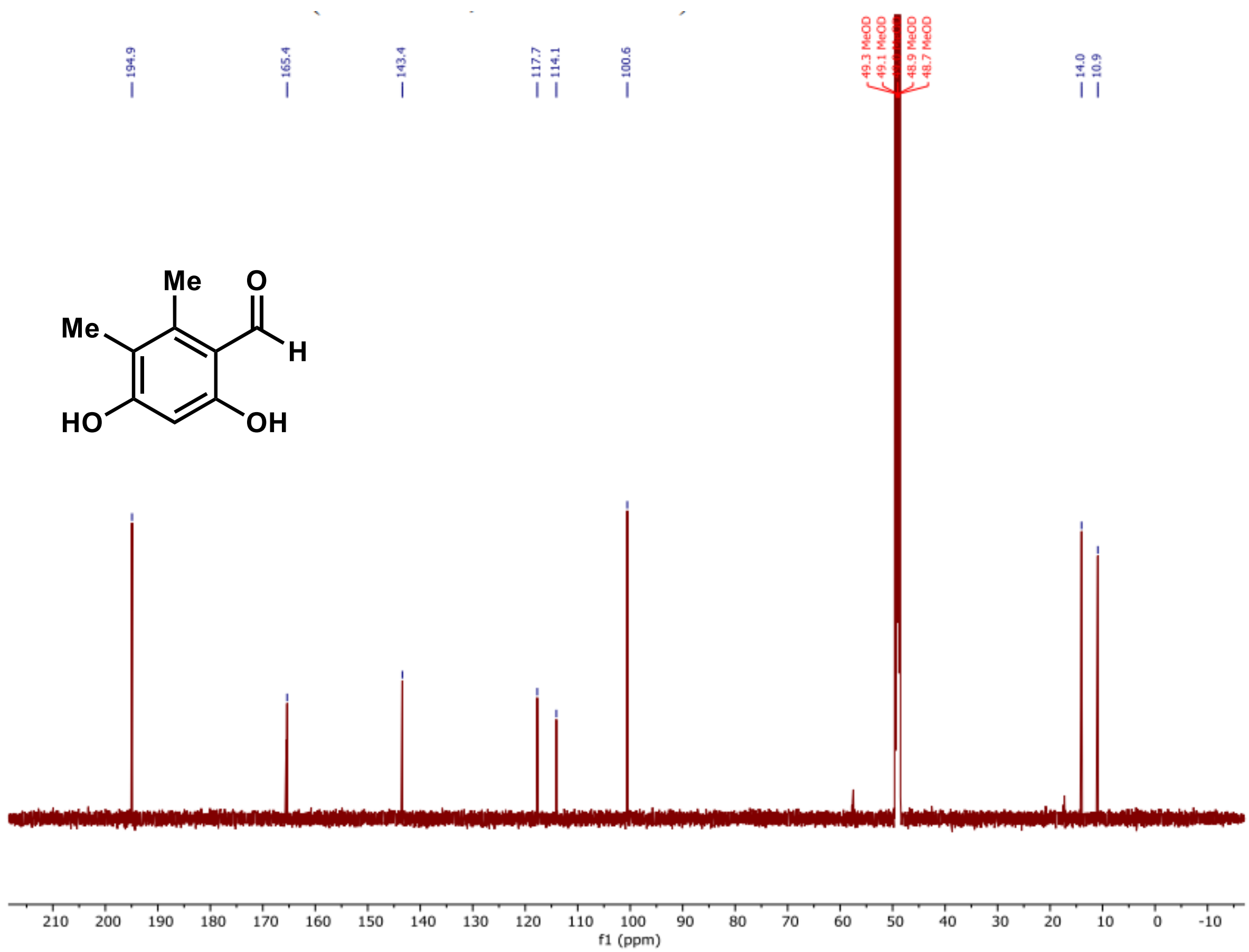

^{1}H-NMR (599 MHz, d_6-DMSO) of 4,6-dihydroxy-3-(hydroxymethyl)-2-methylbenzaldehyde (13)

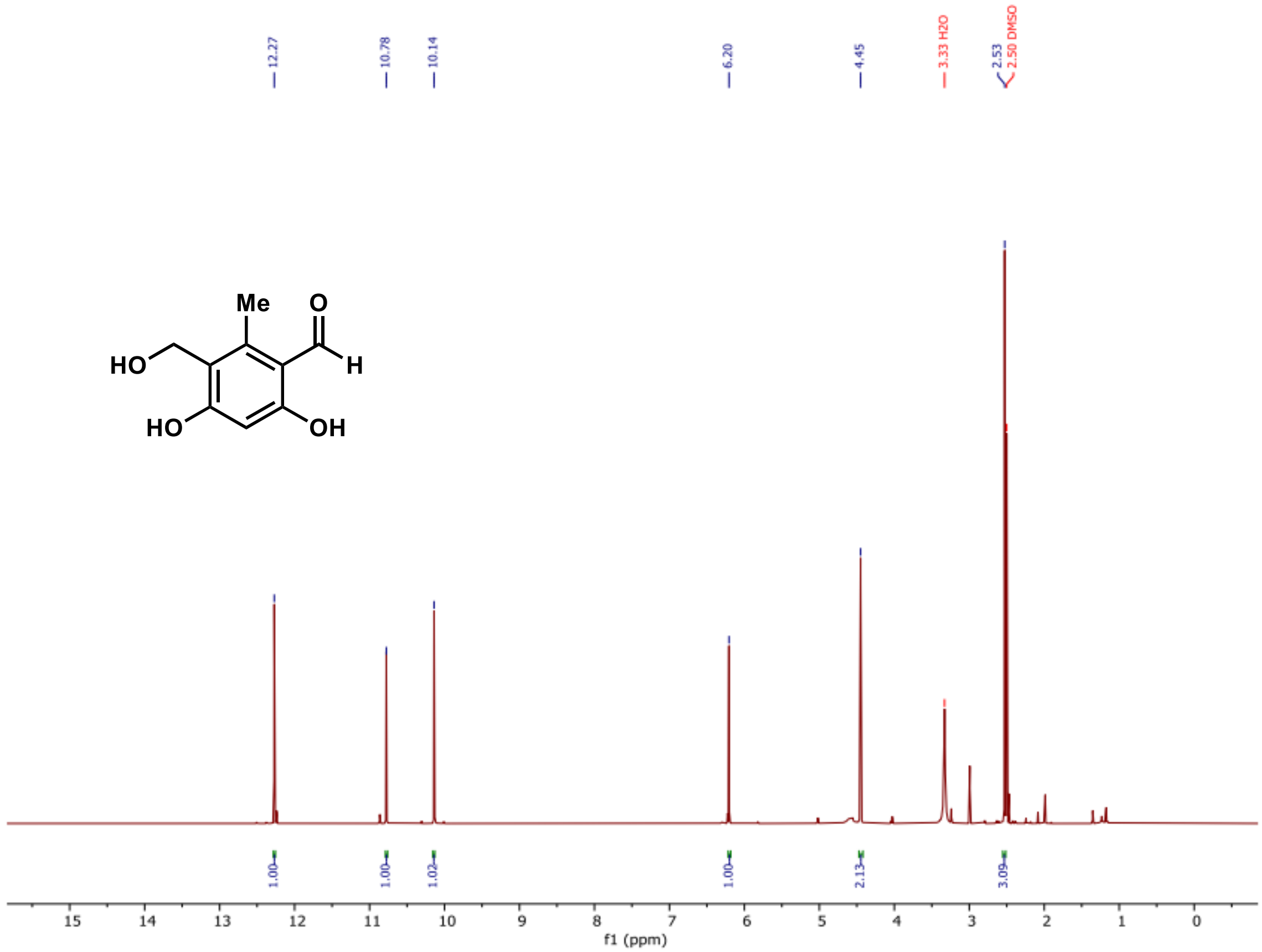

^{13}C-NMR (151 MHz, d_6-DMSO) of 4,6-Dihydroxy-3-(hydroxymethyl)-2-methylbenzaldehyde (13):

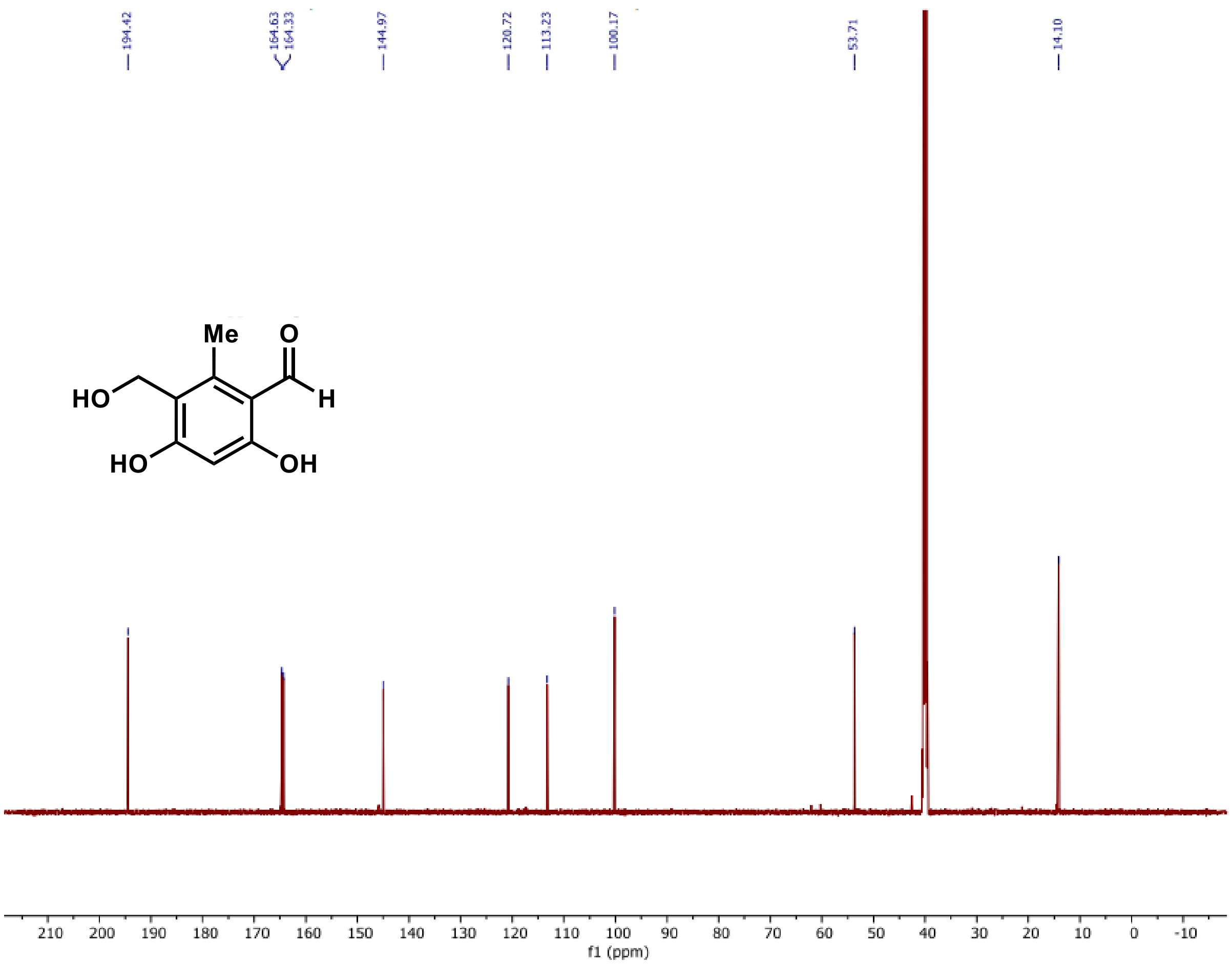

^{1}H-NMR (599 MHz, $CDCl_3$) of (3*R*,3a*R*,9a*R*)-7-hydroxy-3,5,9a-trimethyl-2,3,3a,9a-tetrahydro-4H-furo[2,3-b]chromene-6-carbaldehyde (22):

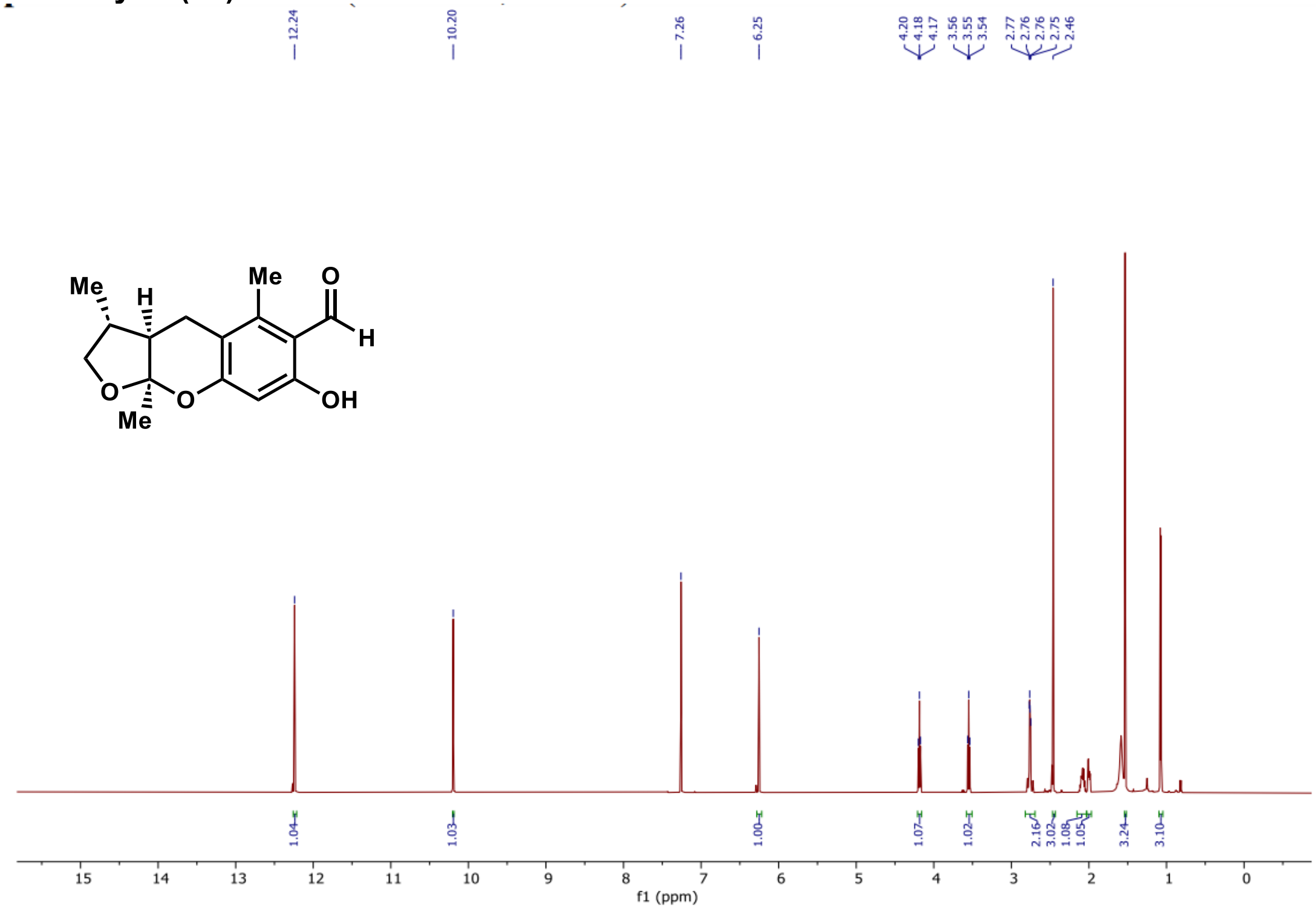

^{13}C-NMR (151 MHz, $CDCl_3$) of (3*R*,3a*R*,9a*R*)-7-hydroxy-3,5,9a-trimethyl-2,3,3a,9a-tetrahydro-4H-furo[2,3-b]chromene-6- carbaldehyde (22):

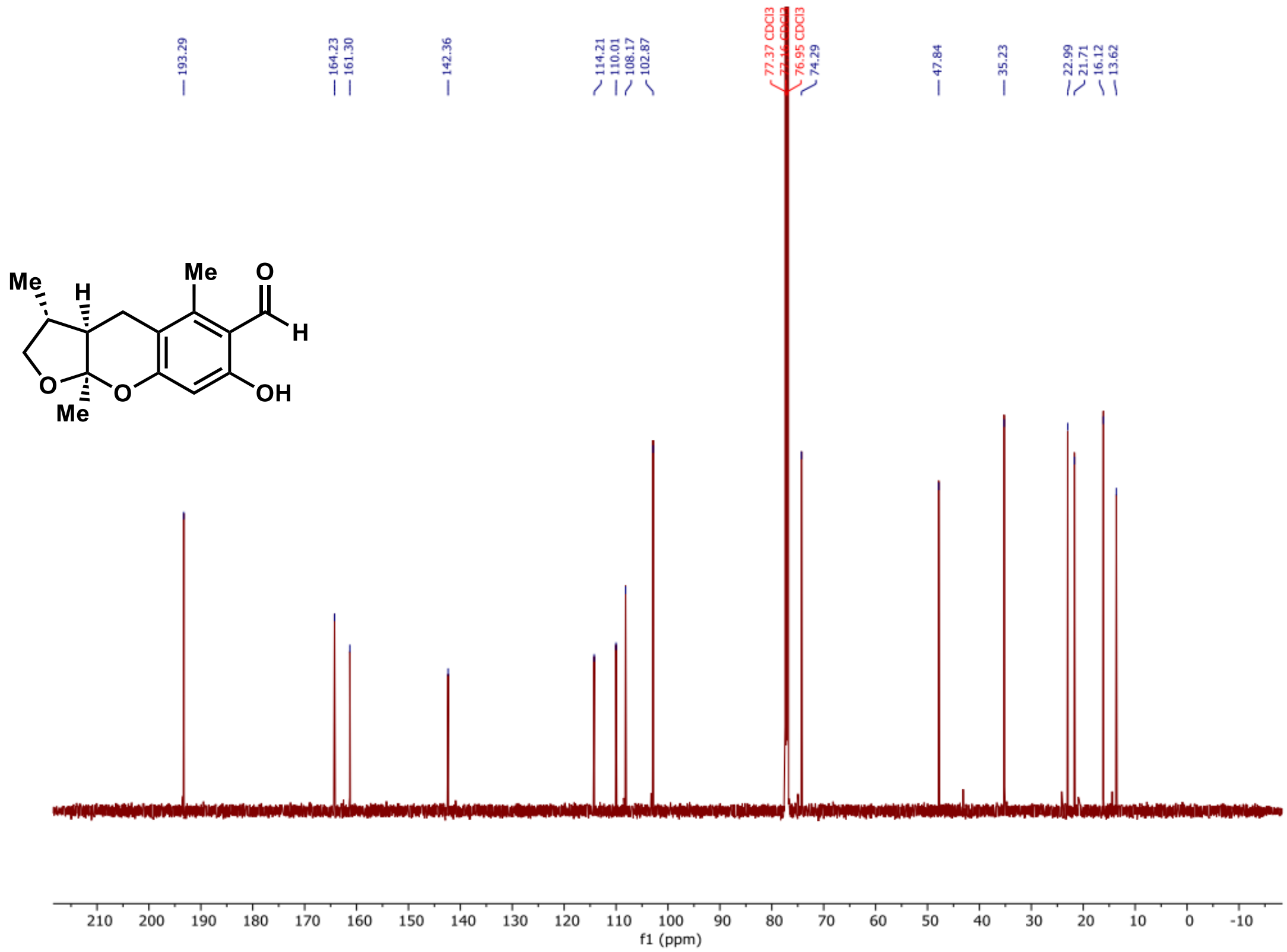

¹H-NMR (599 MHz, d_6-DMSO) of phomeketale B (6):

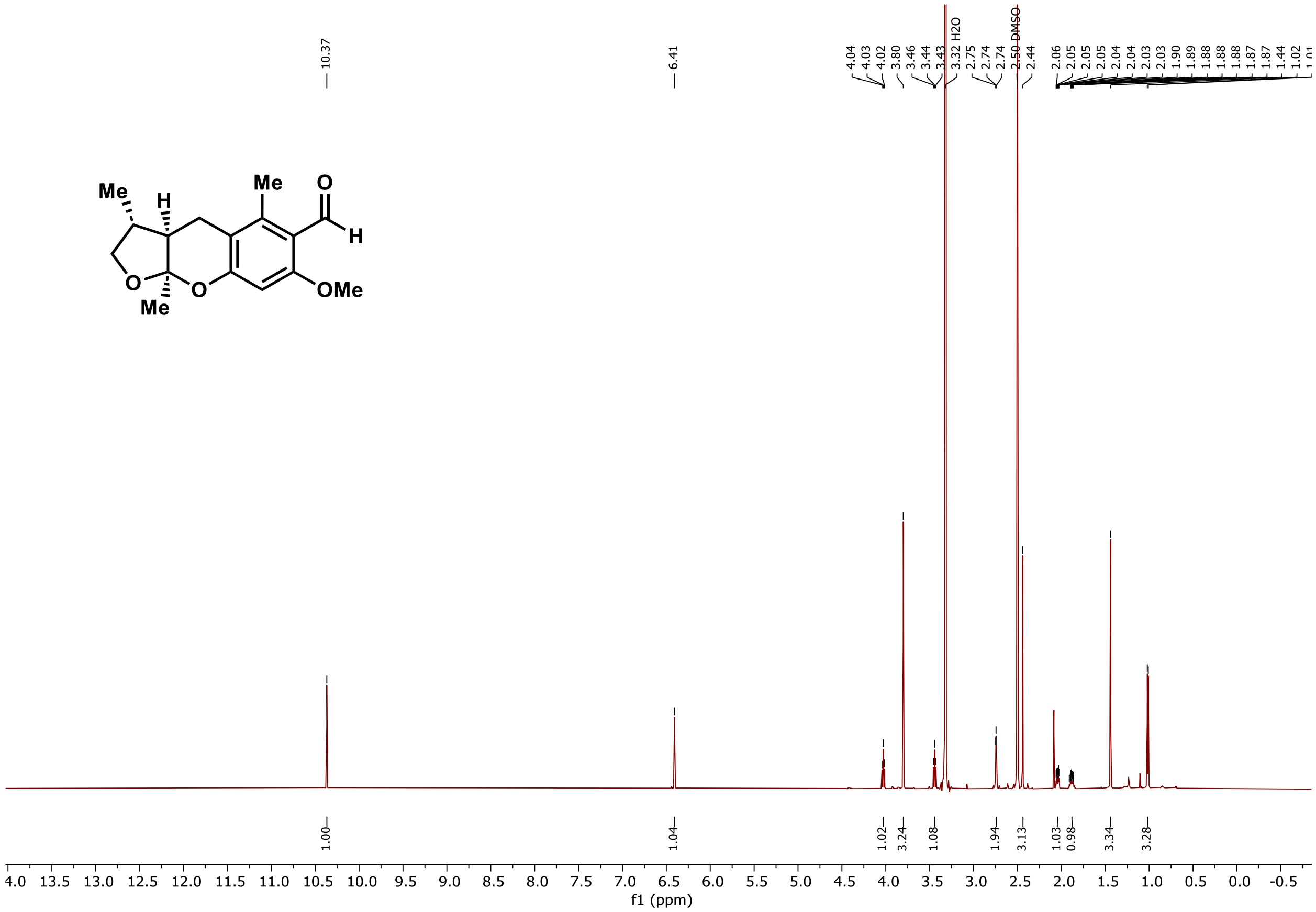

^{13}C-NMR (151 MHz, d_6-DMSO) of phomeketale B:

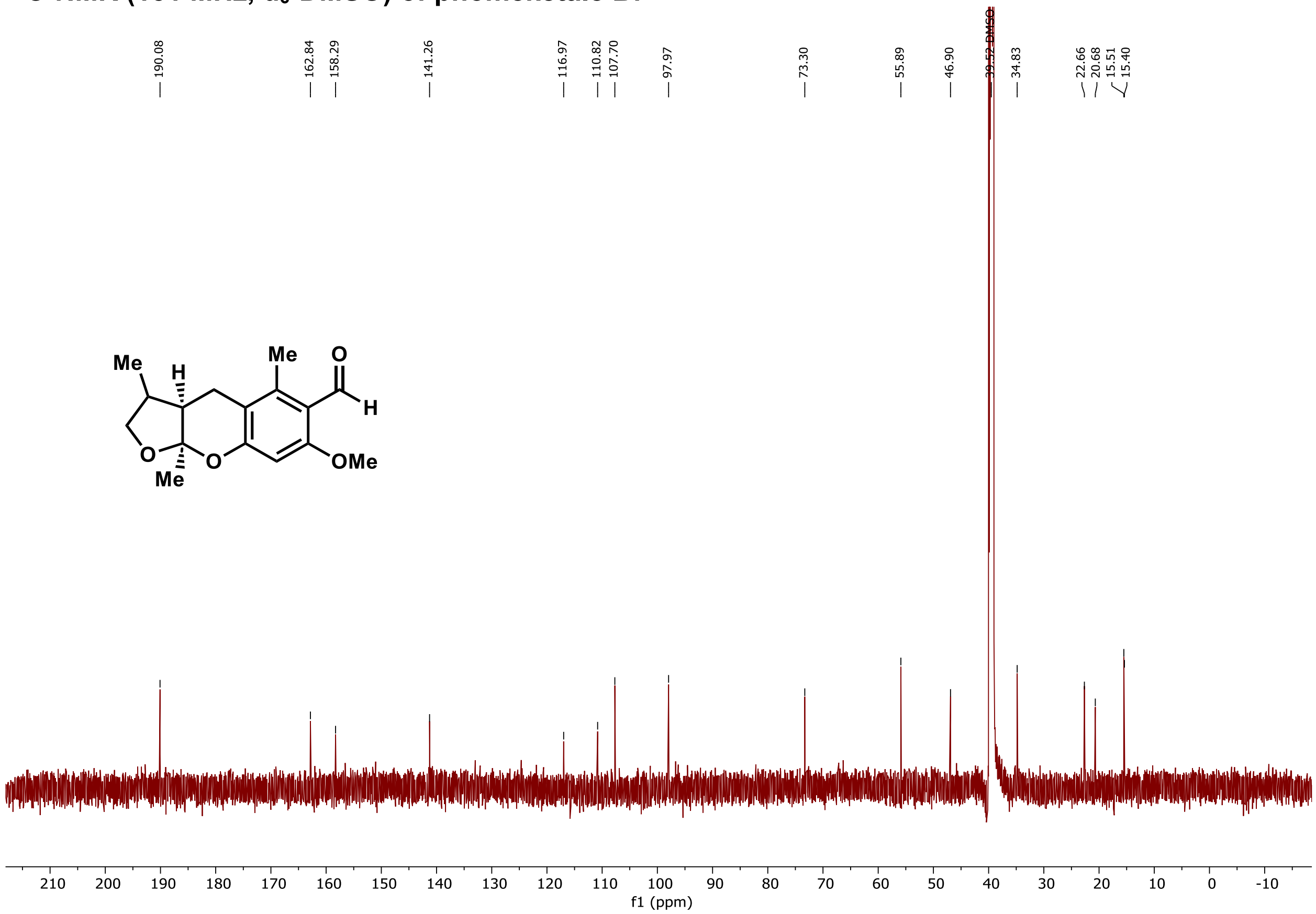

^{1}H-NMR (599 MHz, $CDCl_3$) of methyl 2,4-dihydroxy-3,5,6-trimethylbenzoate (S21):

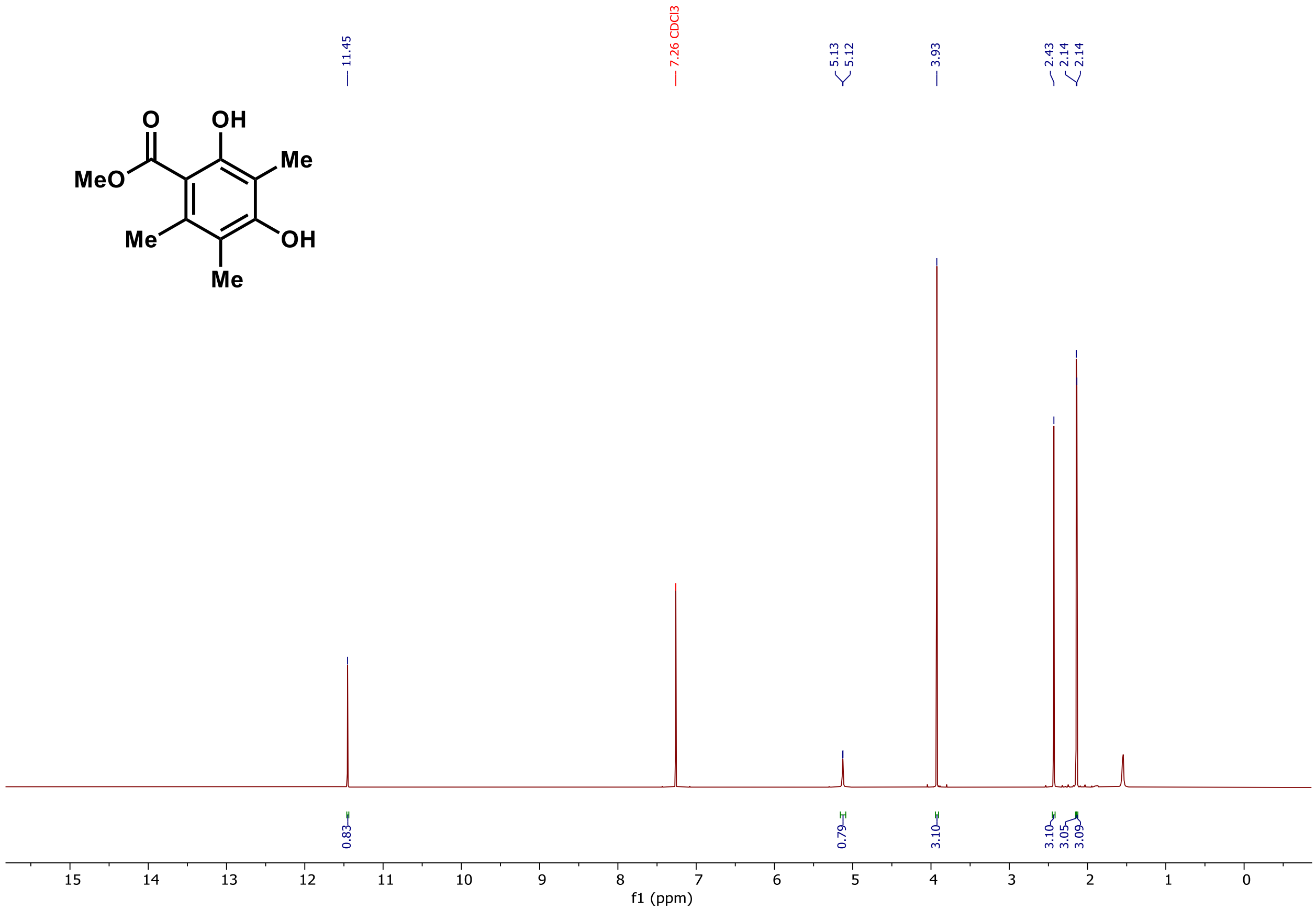

^{1}H-NMR (599 MHz, $CDCl_3$) of methyl 2-hydroxy-4-(methoxymethoxy)-3,5,6-trimethylbenzoate (S22):

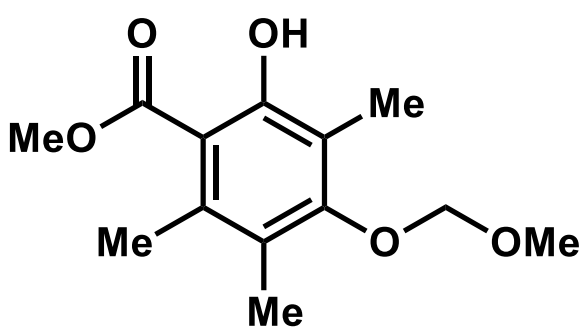

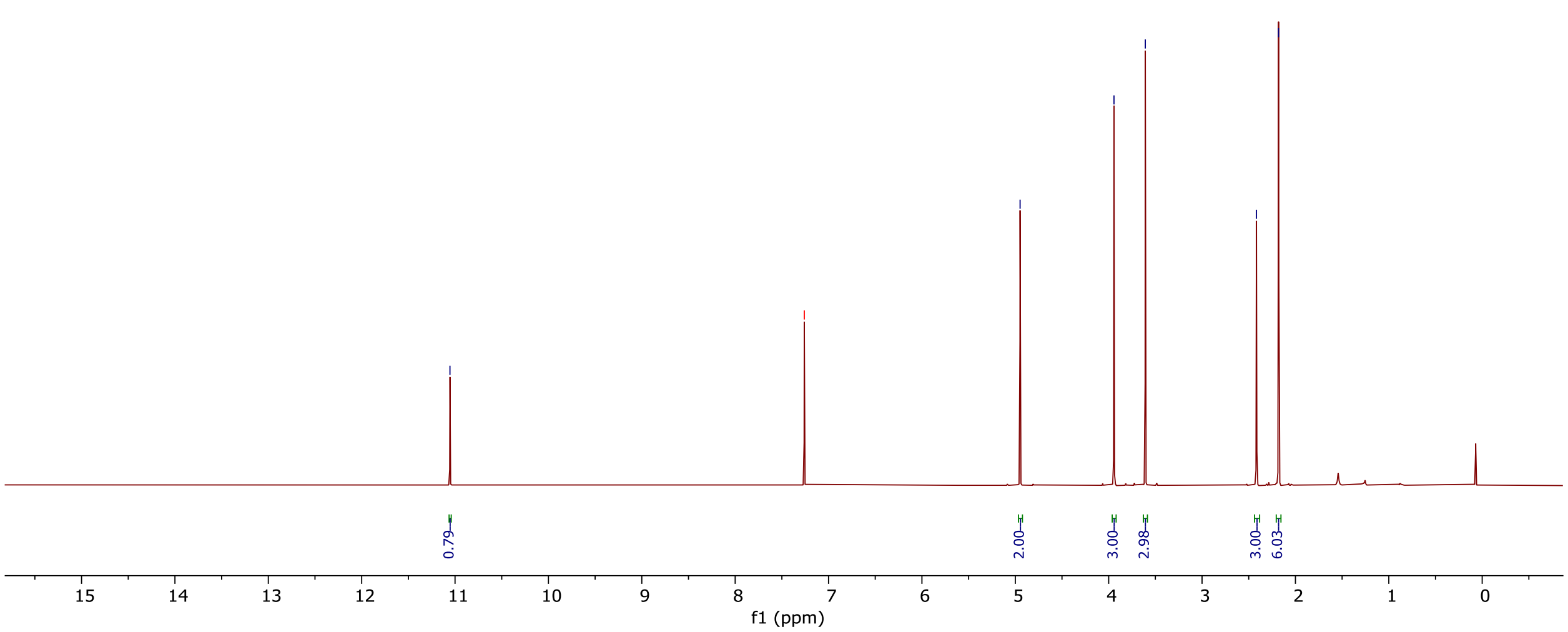

^{13}C-NMR (151 MHz, $CDCl_3$) of methyl 2-hydroxy-4-(methoxymethoxy)-3,5,6-trimethylbenzoate (S22):

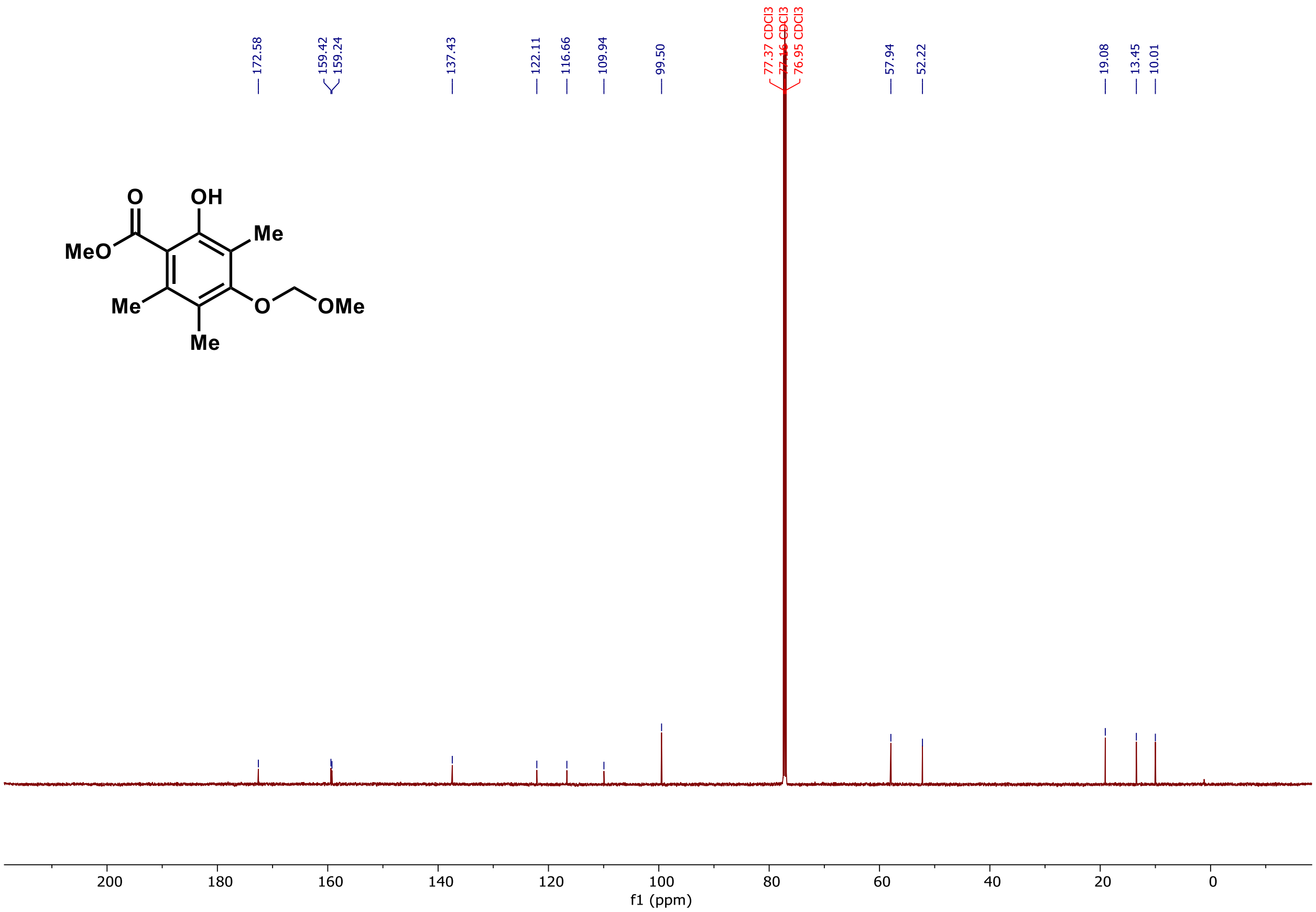

^{1}H-NMR (599 MHz, $CDCl_3$) of methyl 2-methoxy-4-(methoxymethoxy)-3,5,6-trimethylbenzoate (S23)

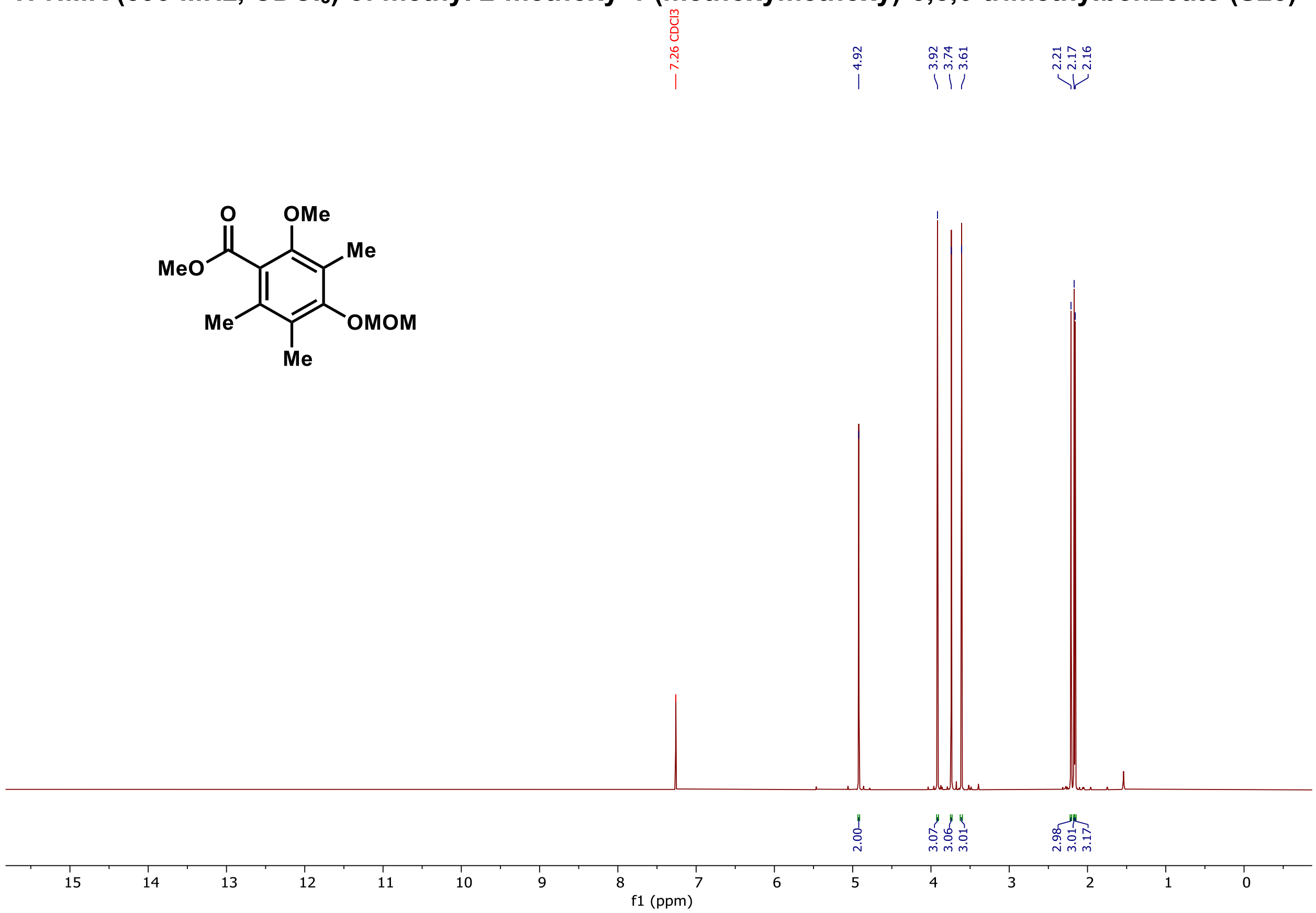

^{13}C-NMR (151 MHz, CDCl$_3$) of methyl 2-methoxy-4-(methoxymethoxy)-3,5,6-trimethylbenzoate (S23):

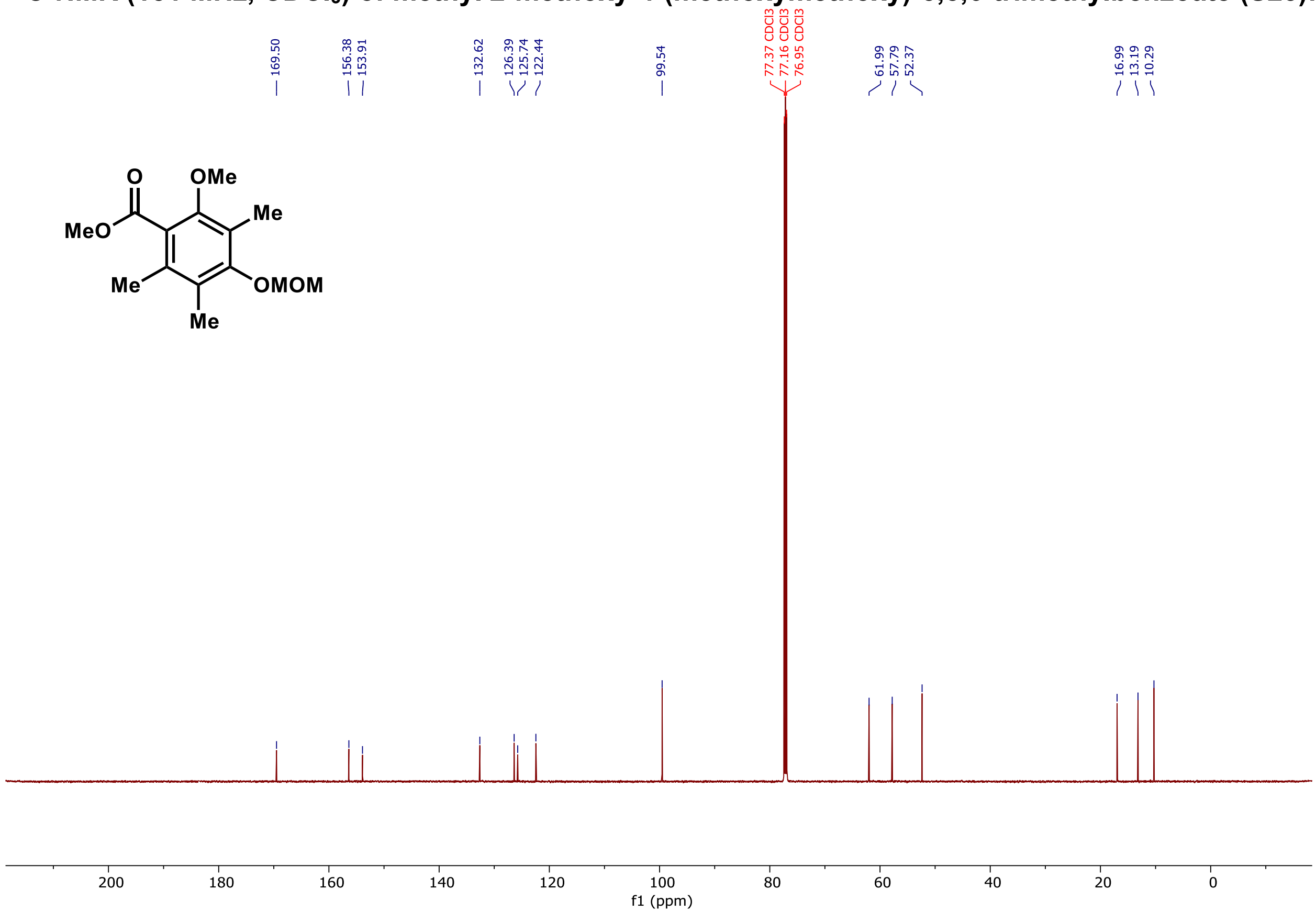

^{1}H-NMR (599 MHz, $CDCl_3$) of 7-methoxy-5-(methoxymethoxy)-4,6-dimethylisobenzofuran-1(3H)-one (S24)

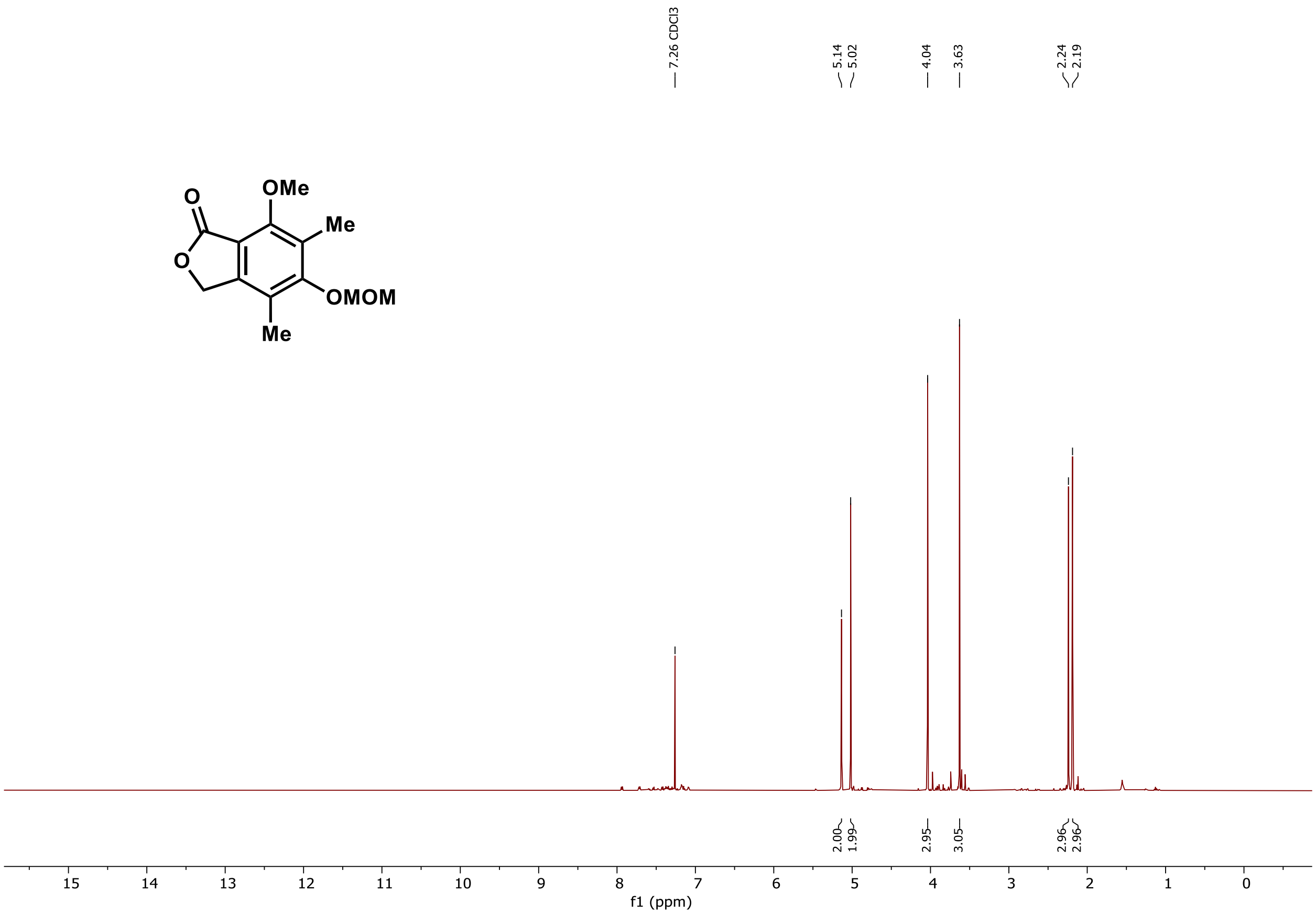

^{13}C-NMR (151 MHz, $CDCl_3$) of 7-methoxy-5-(methoxymethoxy)-4,6-dimethylisobenzofuran-1(3H)-one (S24):

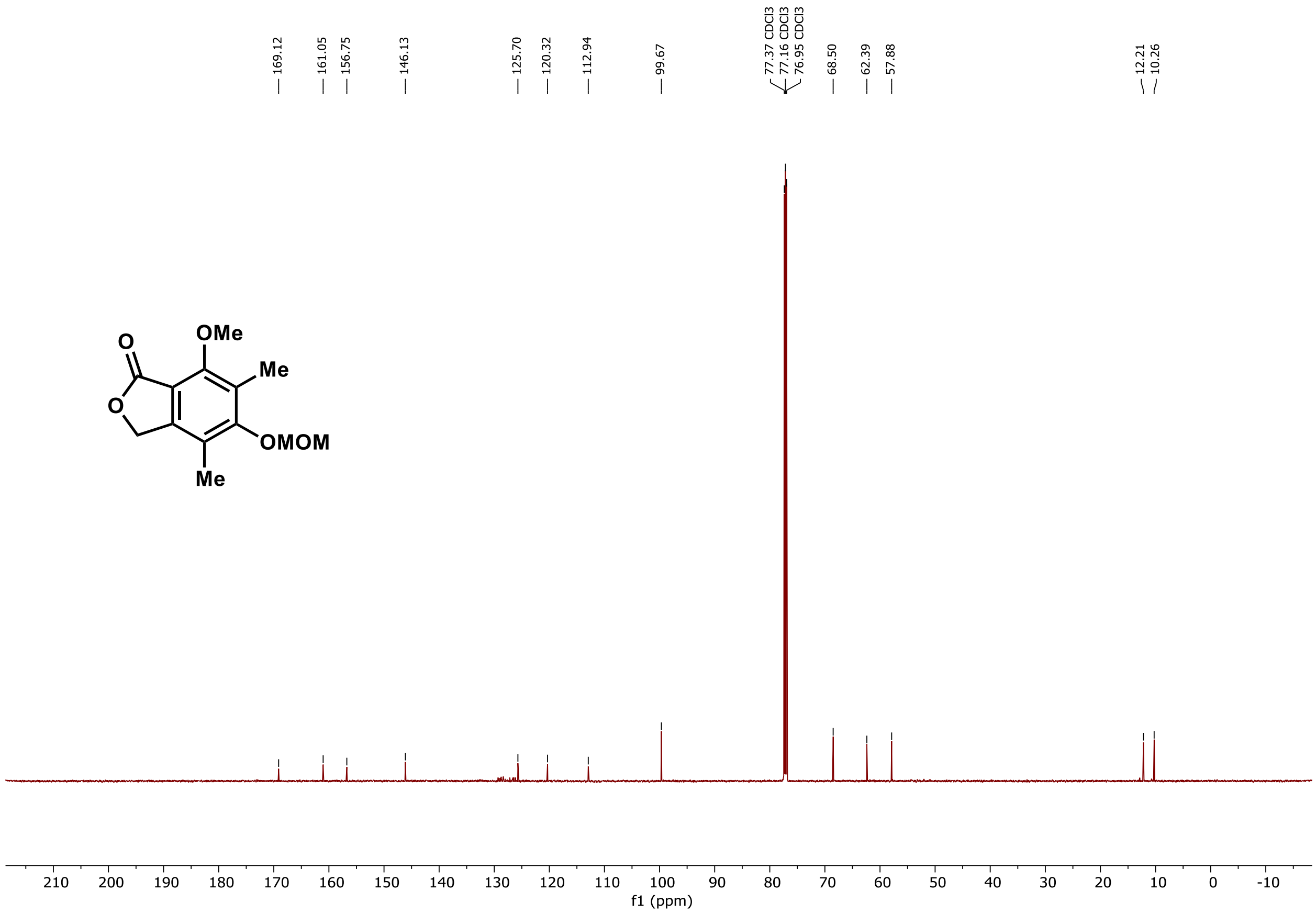

^{1}H-NMR (599 MHz, d_6-acetone) of 5-hydroxy-7-methoxy-4,6-dimethylisobenzofuran-1(3H)-one (9):

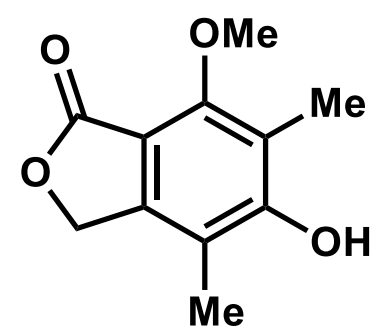

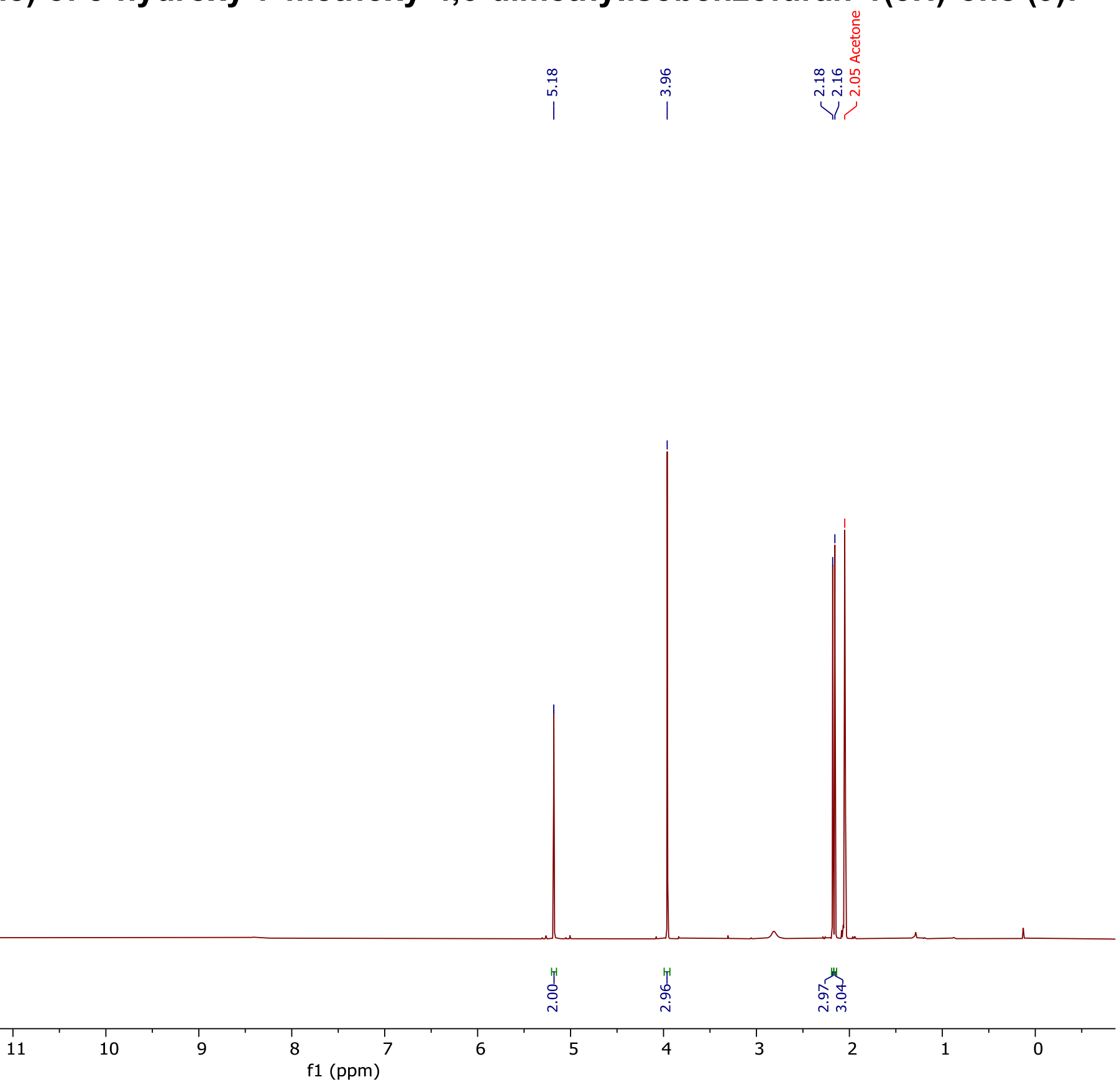

^{13}C-NMR (151 MHz, d_6-acetone) of 5-hydroxy-7-methoxy-4,6-dimethylisobenzofuran-1(3H)-one (9):

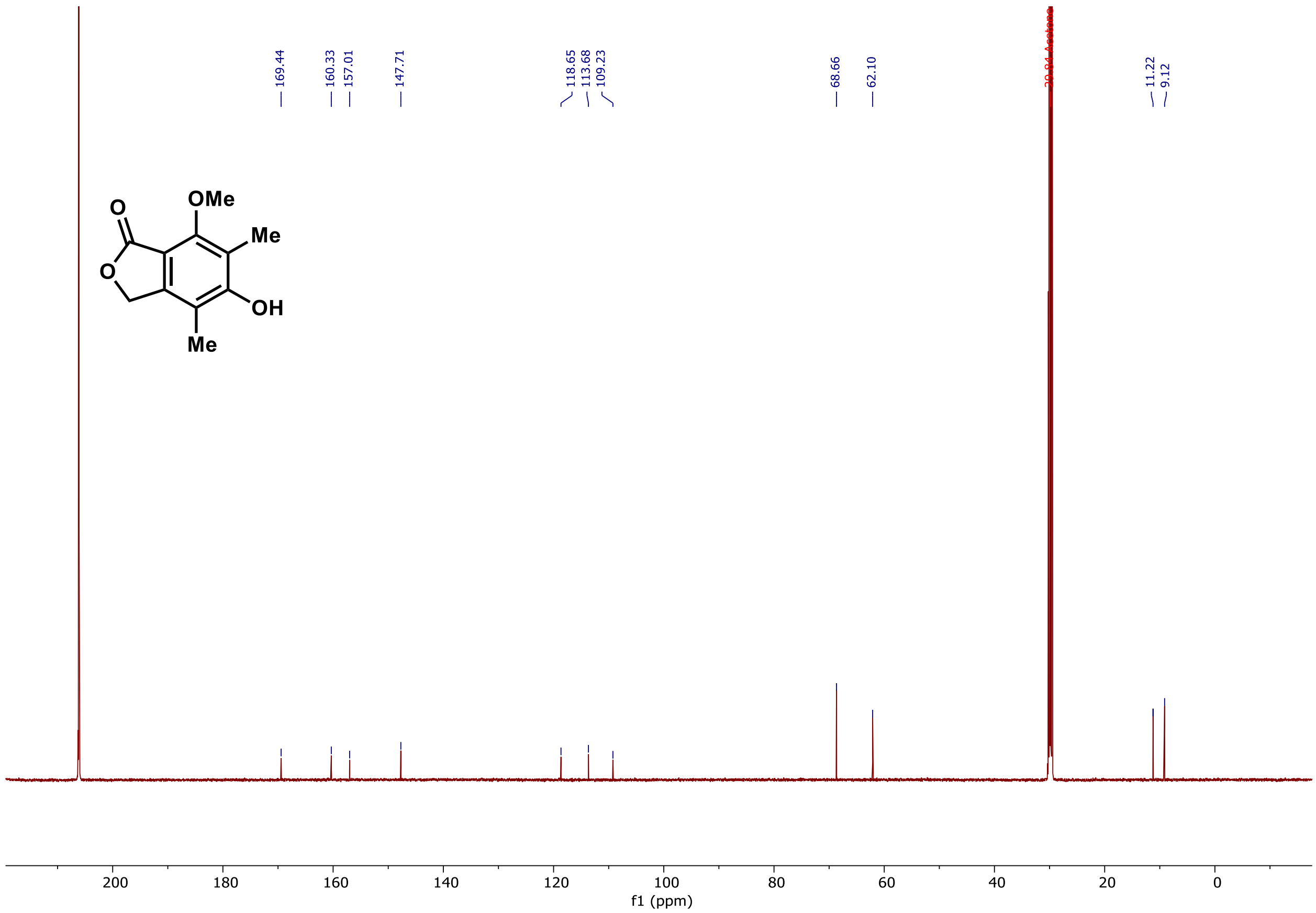

^{1}H-NMR (599 MHz, d_6-acetone) of 5-hydroxy-6-(hydroxymethyl)-7-methoxy-4-methylisobenzofuran-1(3H)-one (14):

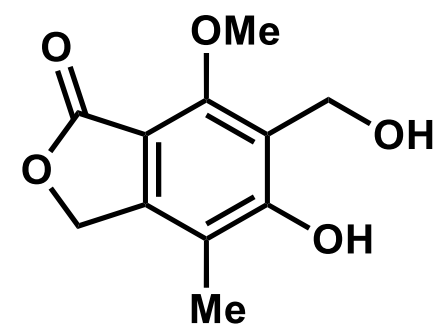

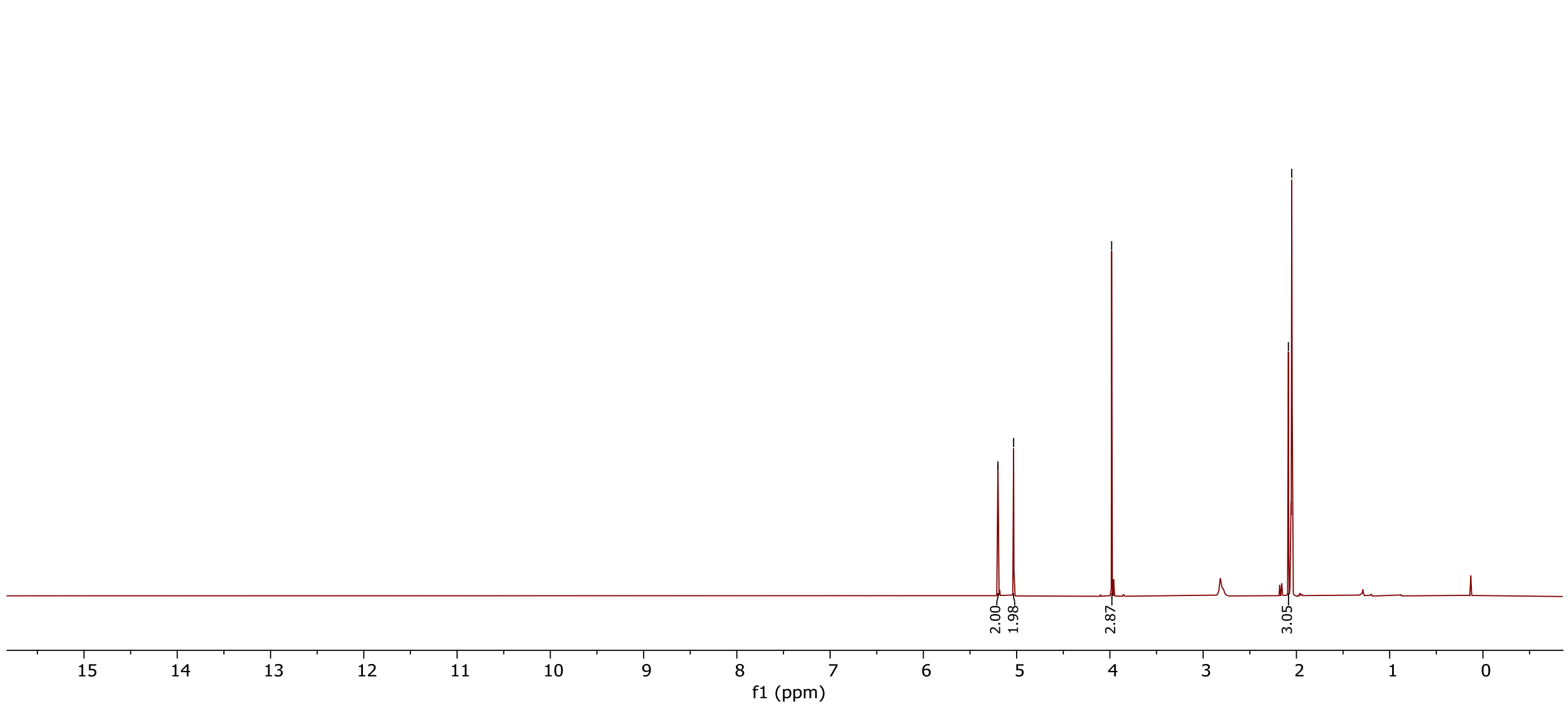

^{13}C-NMR (151 MHz, d_6-acetone) of 5-hydroxy-6-(hydroxymethyl)-7-methoxy-4-methylisobenzofuran-1(3H)-one (14):

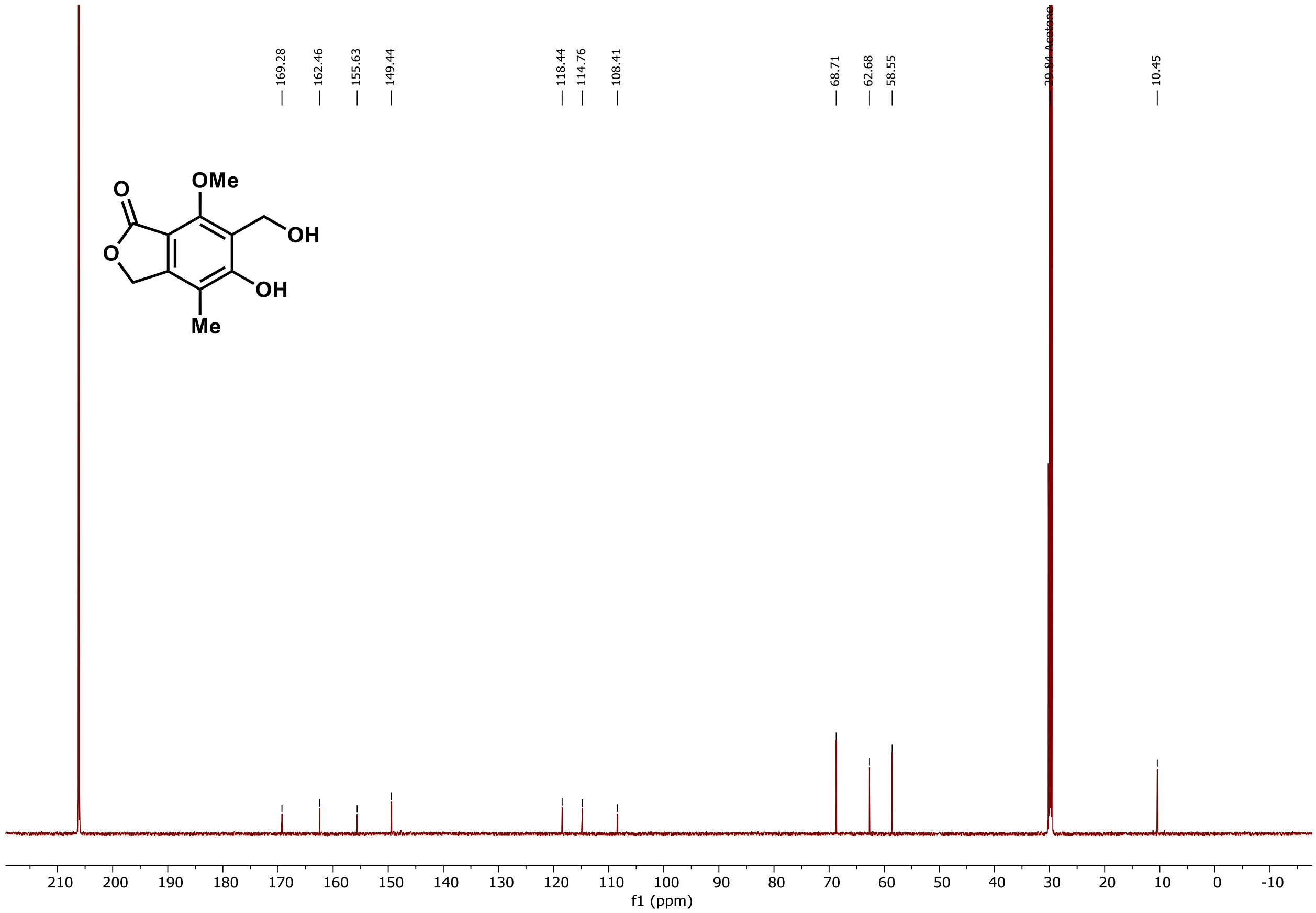

NOESY of 5-hydroxy-6-(hydroxymethyl)-7-methoxy-4-methylisobenzofuran-1(3H)-one (14):

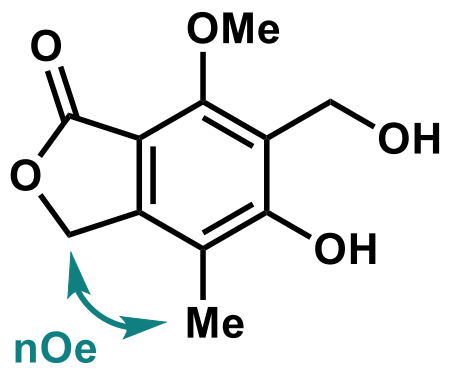

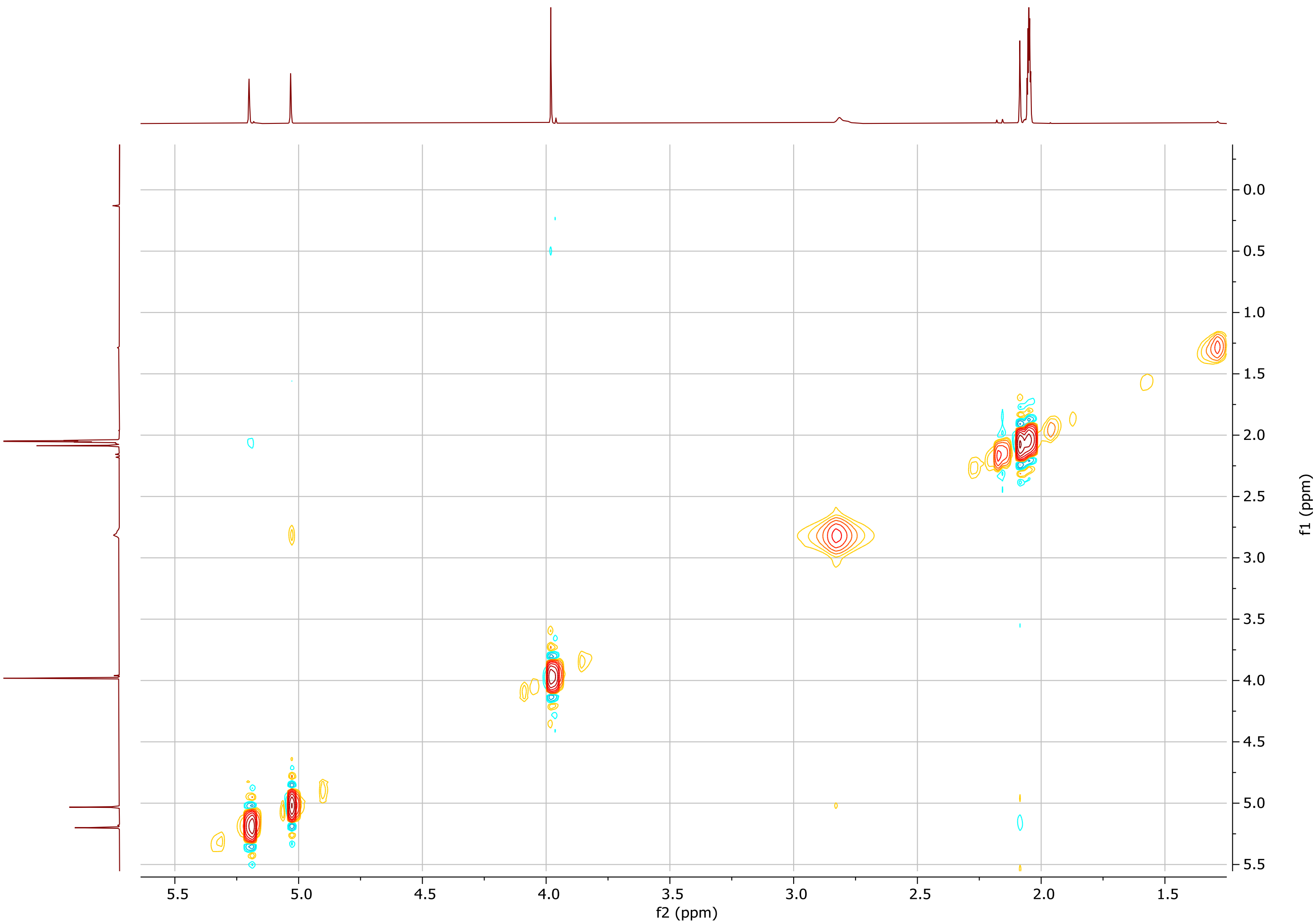

^{1}H-NMR (599 MHz, $CDCl_3$) of 6-allyl-5-hydroxy-7-methoxy-4-methylisobenzofuran-1(3H)-one (23):

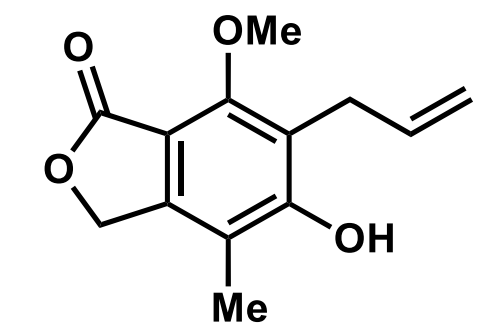

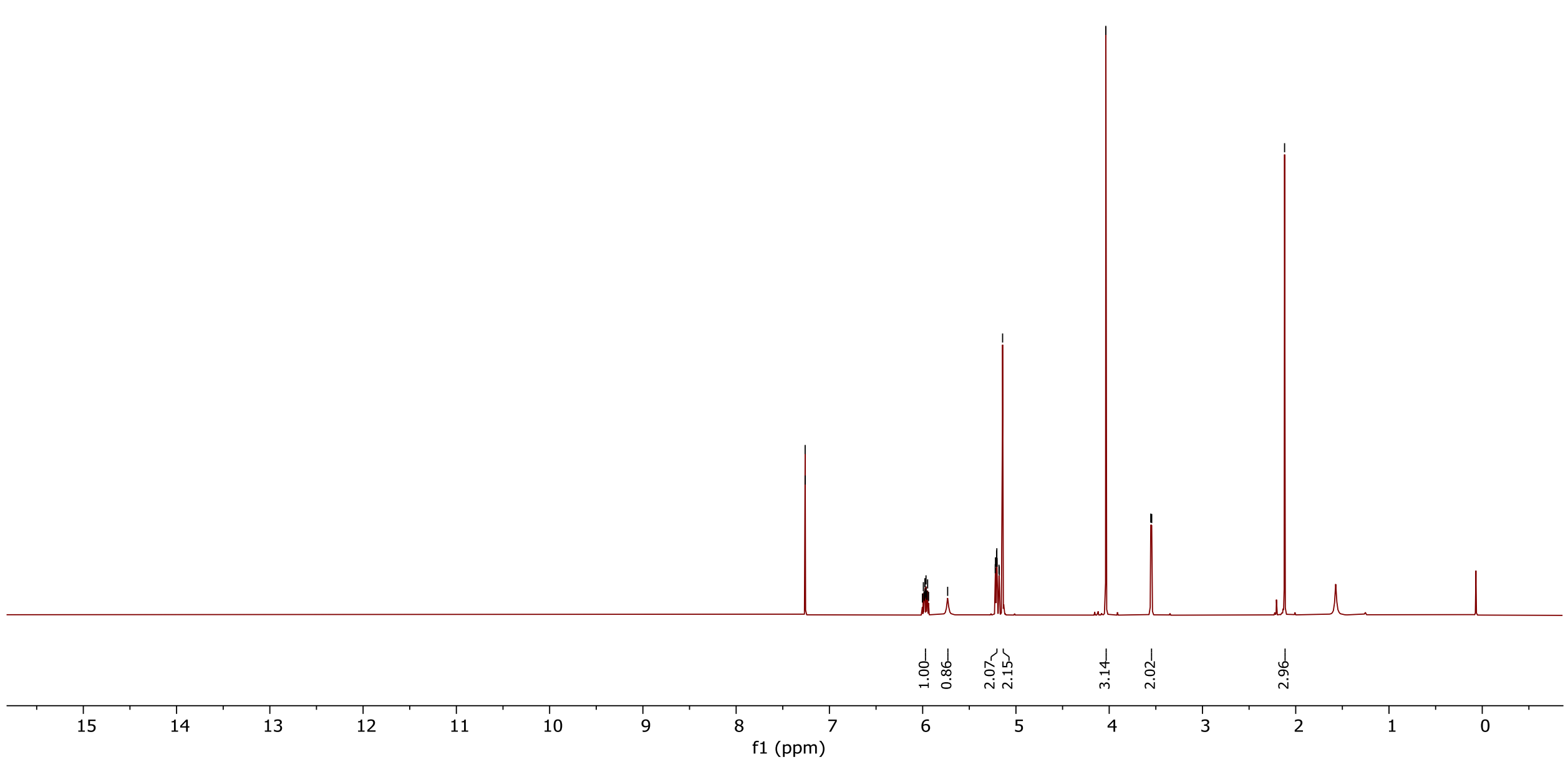

^{13}C-NMR (151 MHz, $CDCl_3$) of 6-allyl-5-hydroxy-7-methoxy-4-methylisobenzofuran-1(3H)-one (23):

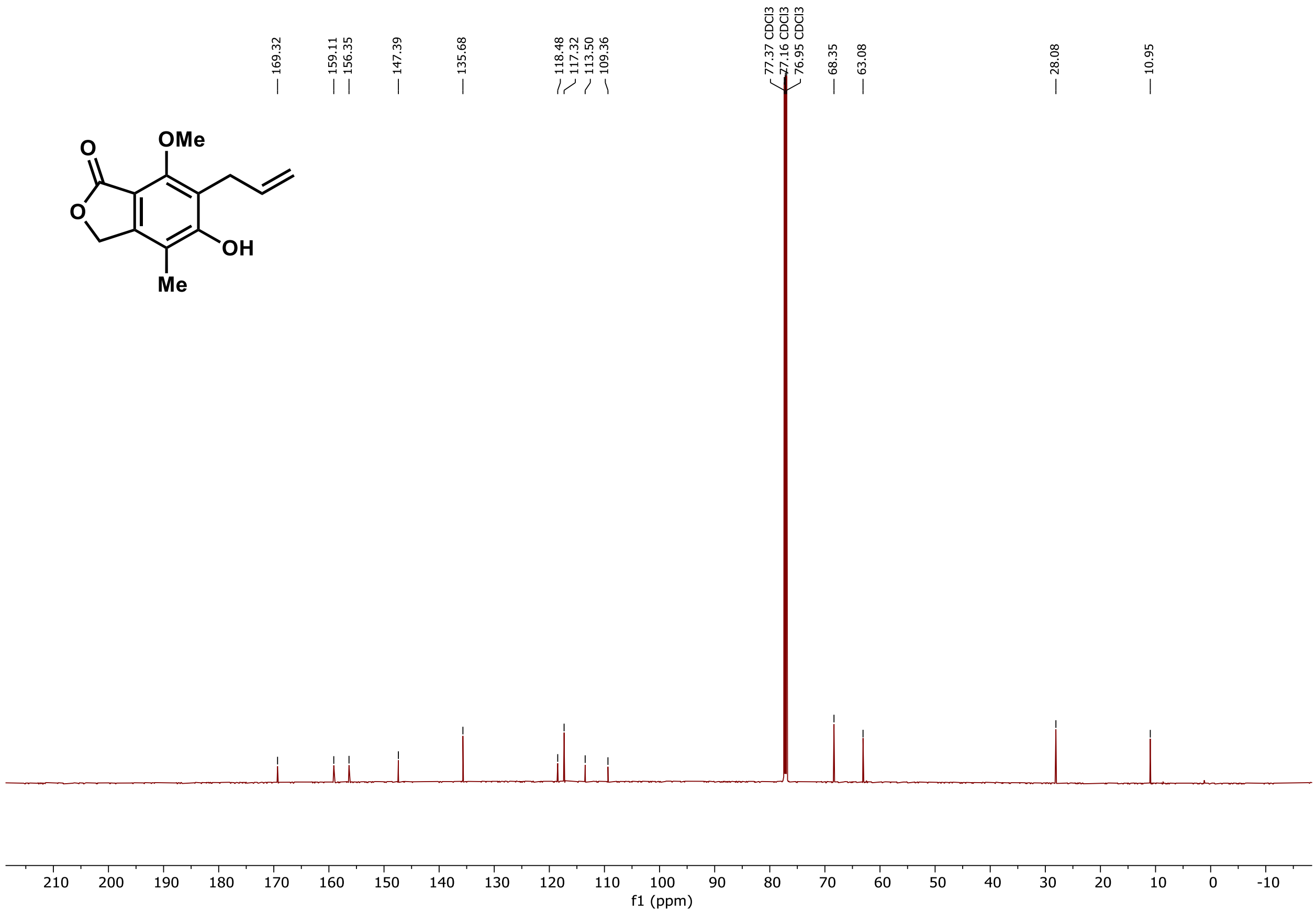

^{1}H-NMR (599 MHz, $CDCl_3$) of 6-allyl-5,7-dimethoxy-4-methylisobenzofuran-1(3H)-one (S25):

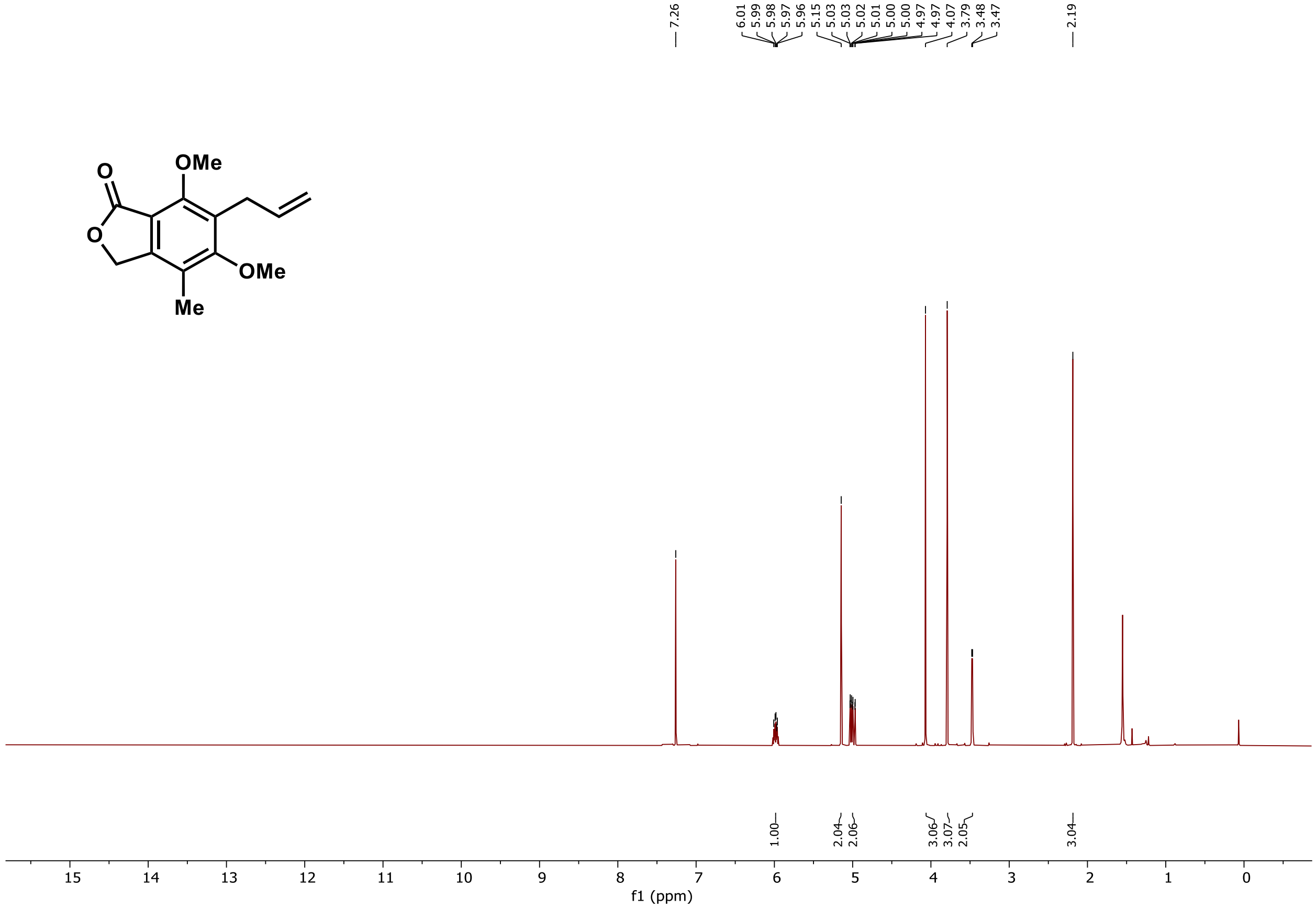

^{13}C-NMR (151 MHz, $CDCl_3$) of 6-allyl-5,7-dimethoxy-4-methylisobenzofuran-1(3H)-one (S25):

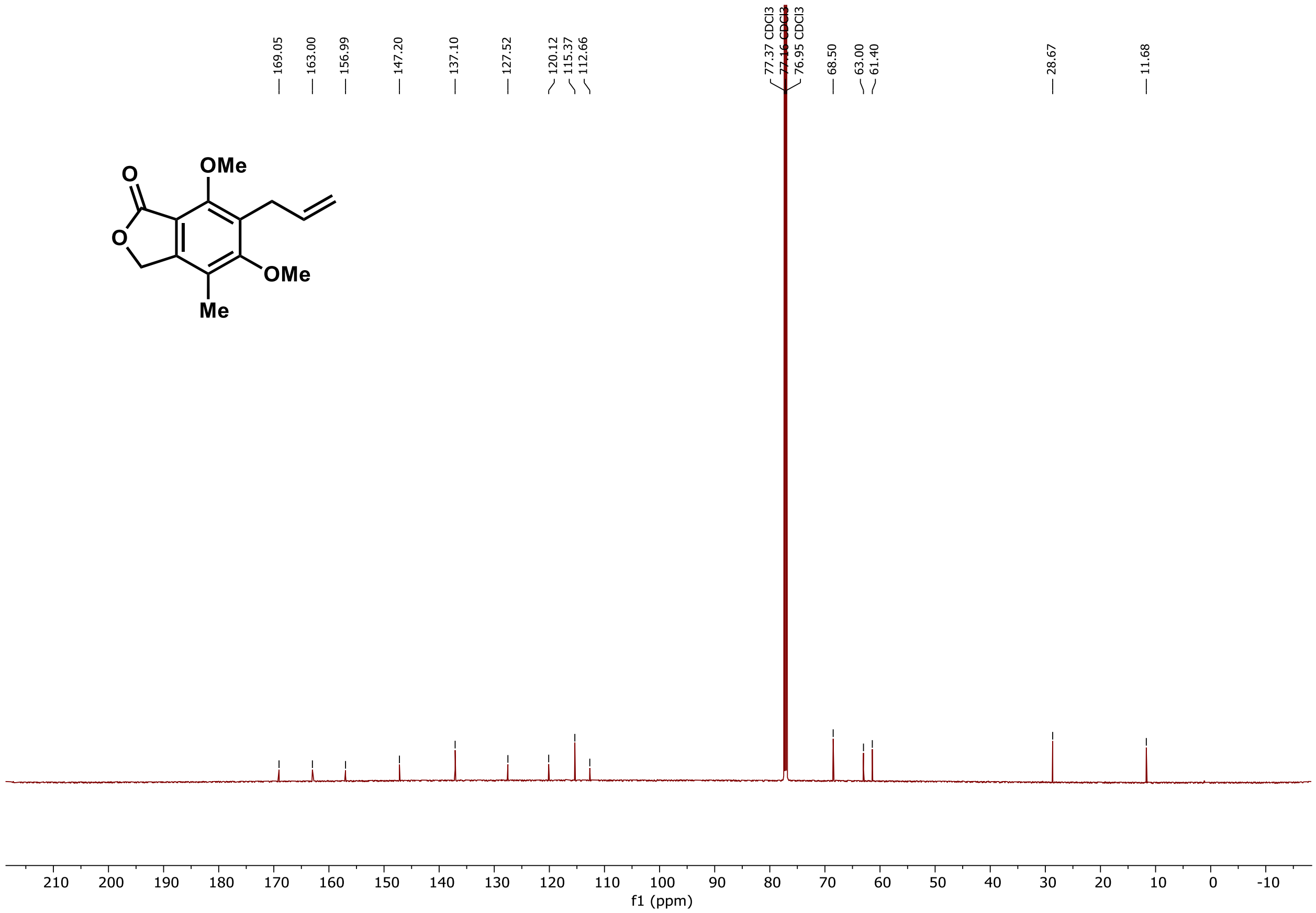

^{1}H-NMR (599 MHz, $CDCl_3$) of 6-allyl-7-hydroxy-5-methoxy-4-methylisobenzofuran-1(3H)-one (24):

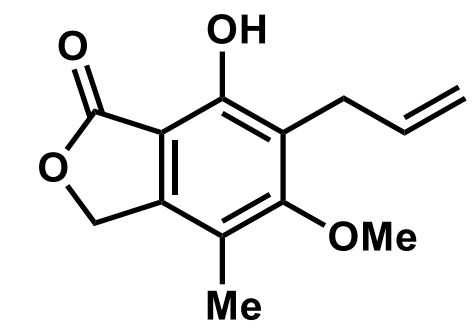

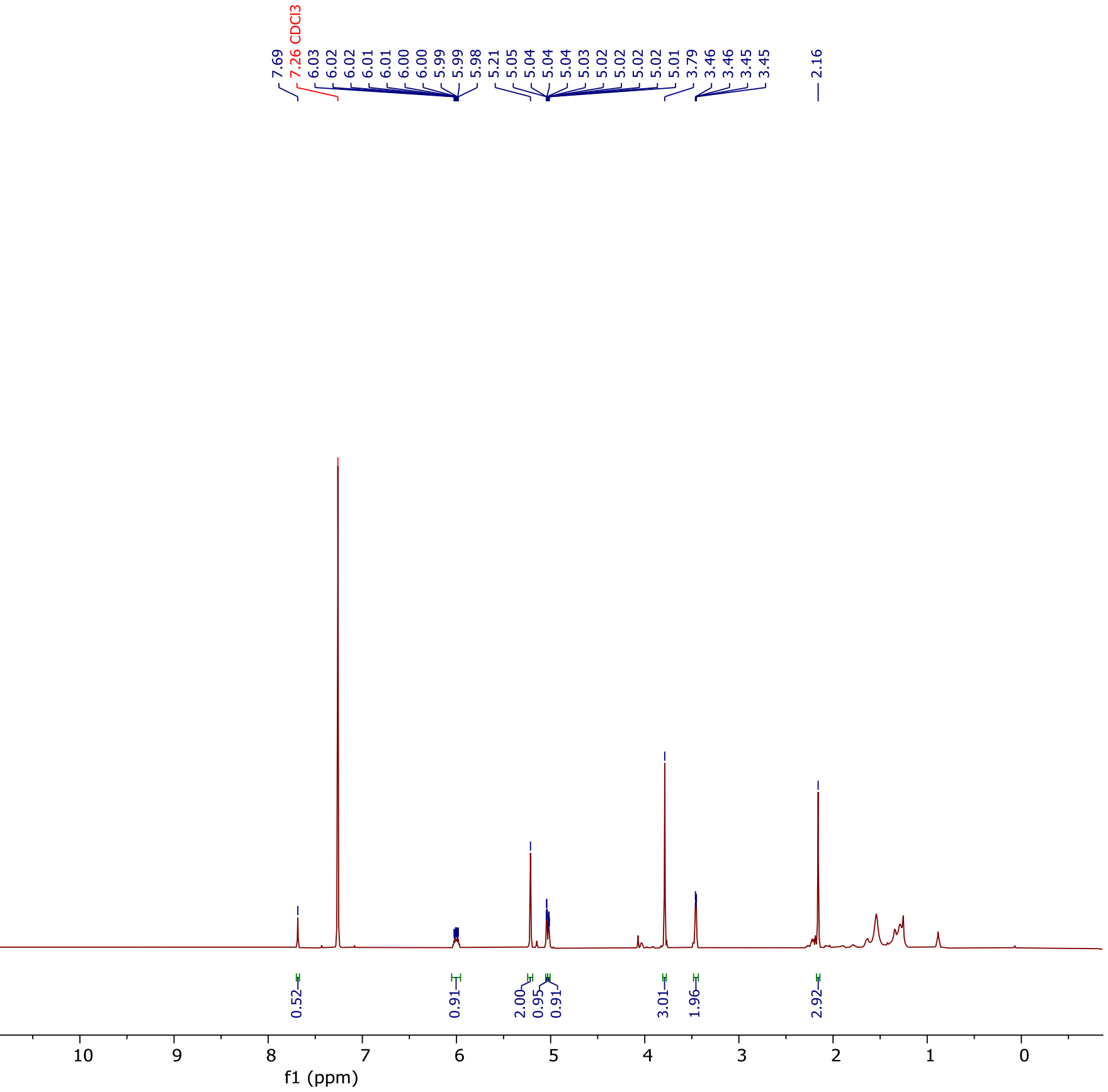

^{13}C-NMR (151 MHz, $CDCl_3$) of 6-allyl-7-hydroxy-5-methoxy-4-methylisobenzofuran-1(3H)-one (24):

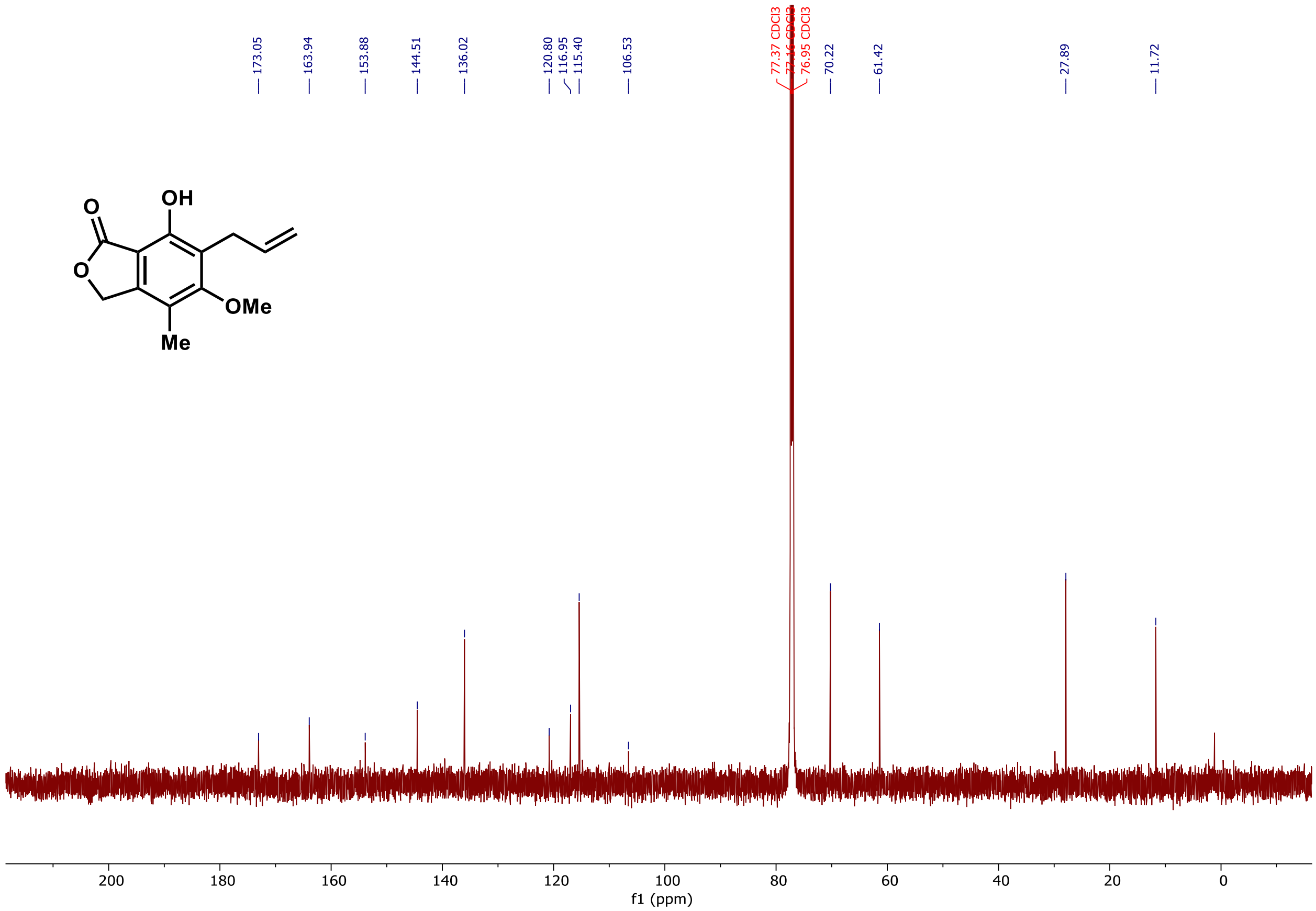

^{1}H-NMR (599 MHz, $CDCl_3$) of 6-(chloromethyl)-2,4-dimethoxy-3-methylbenzaldehyde (S27):

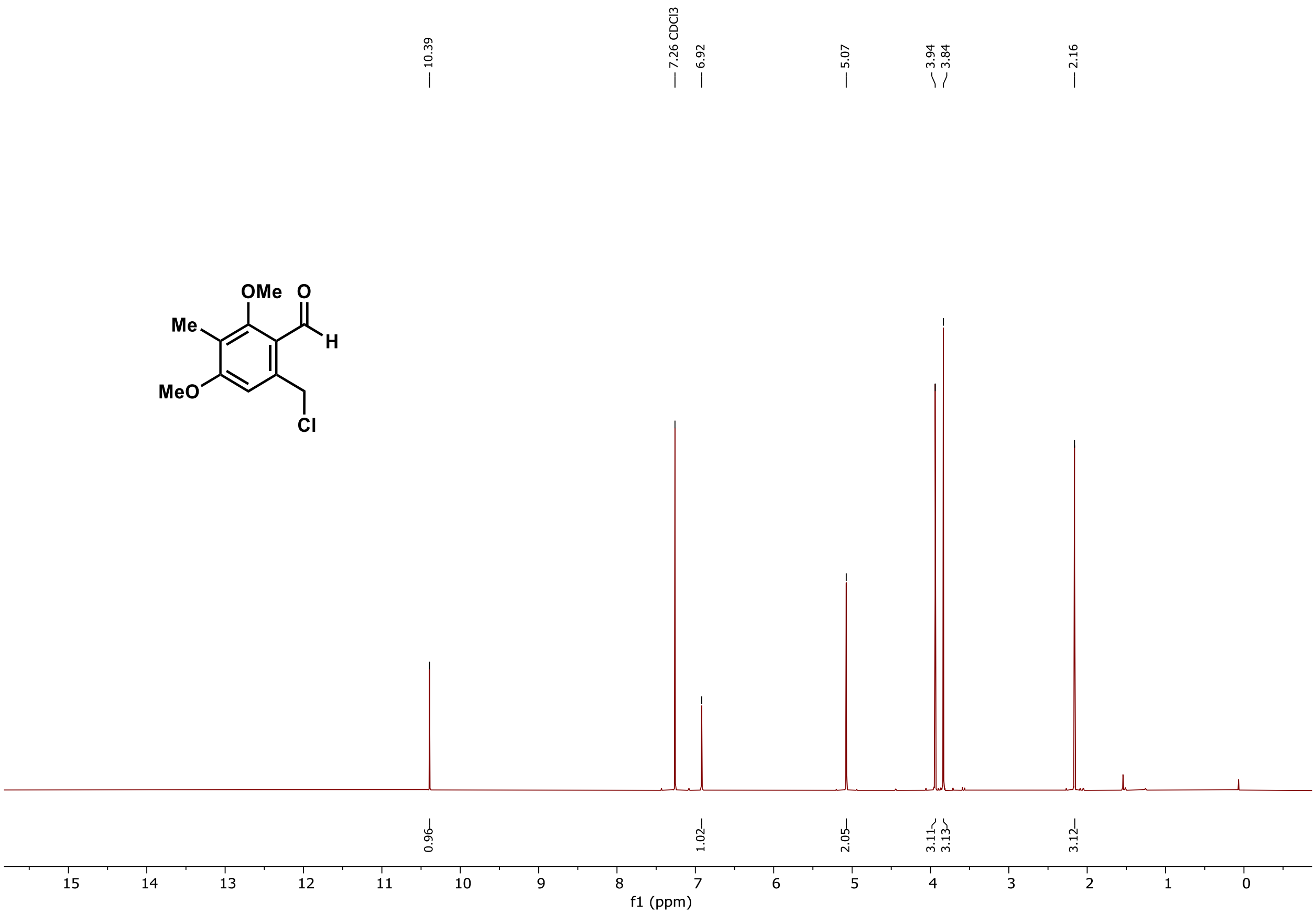

^{13}C-NMR (151 MHz, $CDCl_3$) of 6-(chloromethyl)-2,4-dimethoxy-3-methylbenzaldehyde (S27):

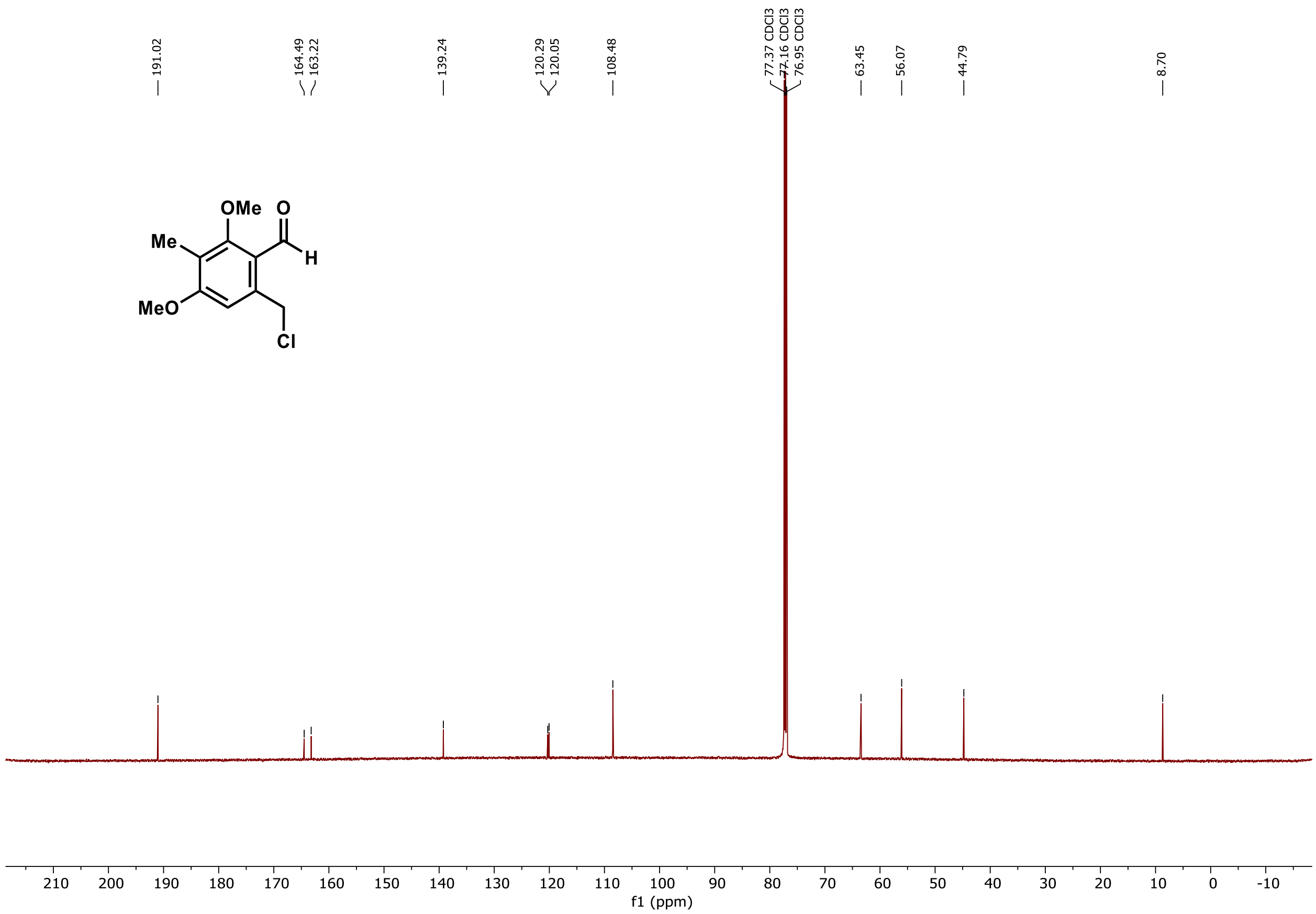

^{1}H-NMR (599 MHz, $CDCl_3$) of 5,7-dimethoxy-6-methylisobenzofuran-1(3H)-one (S28):

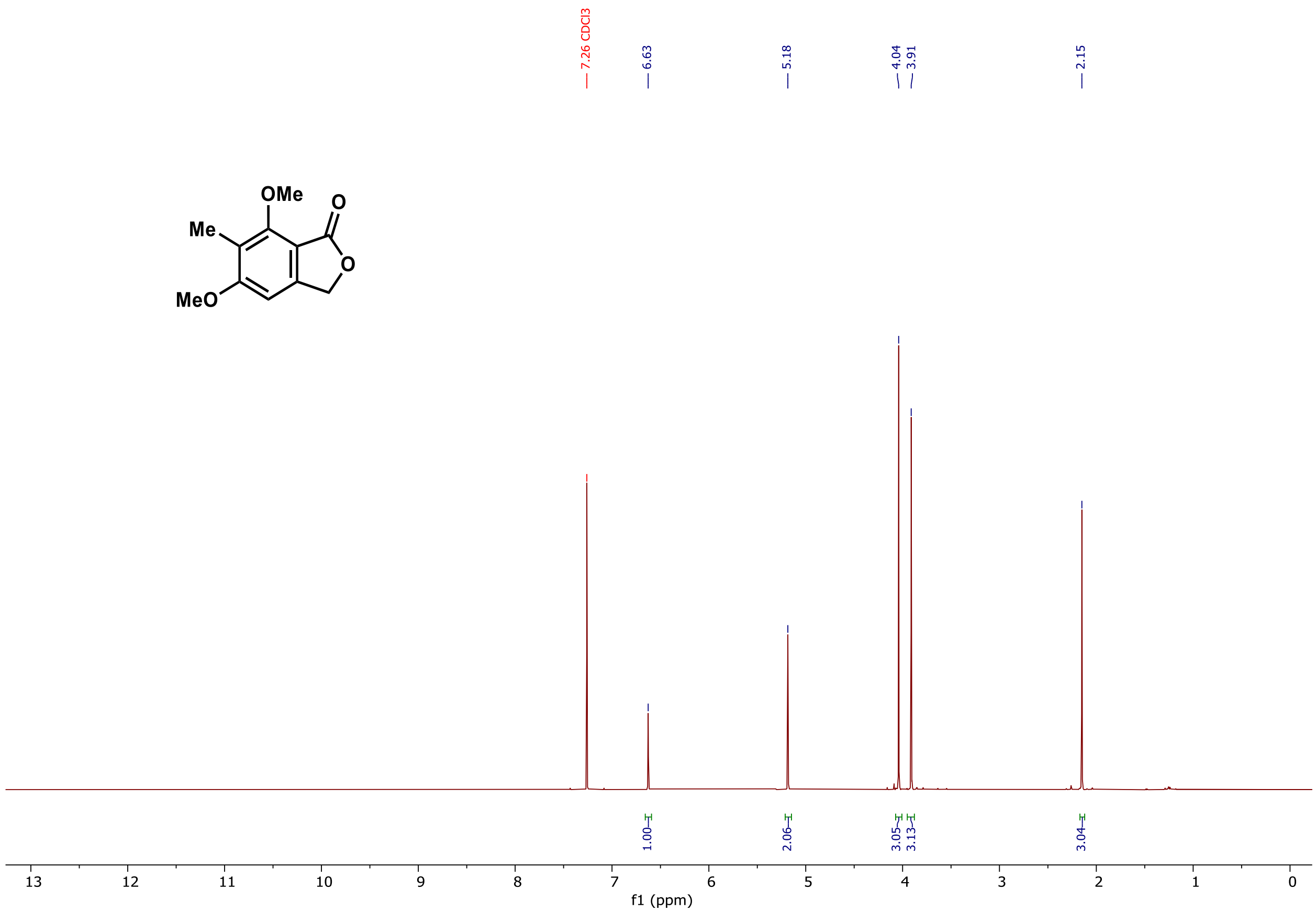

^{13}C-NMR (151 MHz, $CDCl_3$) of 5,7-dimethoxy-6-methylisobenzofuran-1(3H)-one (S28):

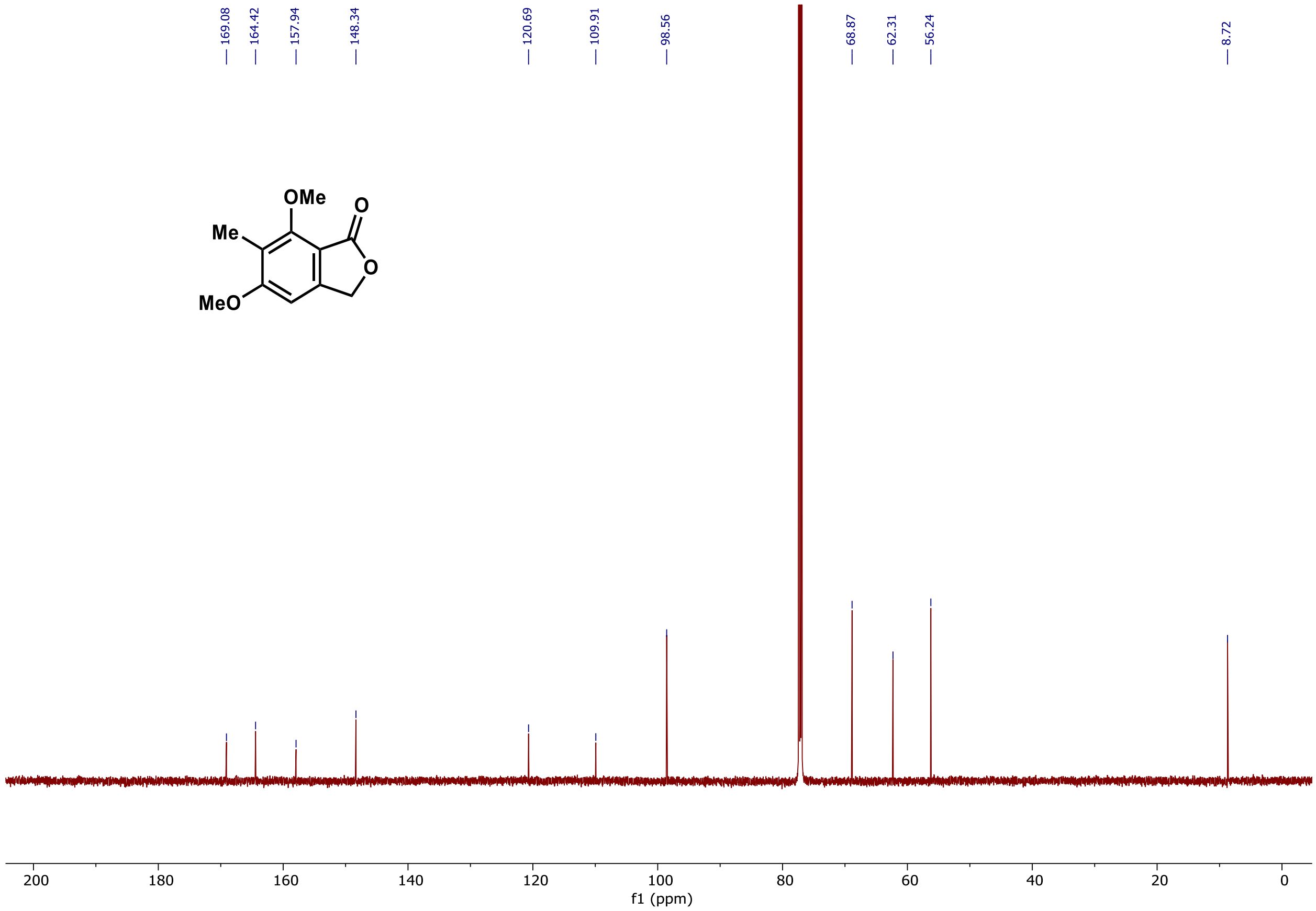

^{1}H-NMR (599 MHz, $CDCl_3$) of 5,7-dihydroxy-6-methylisobenzofuran-1(3H)-one (S29)

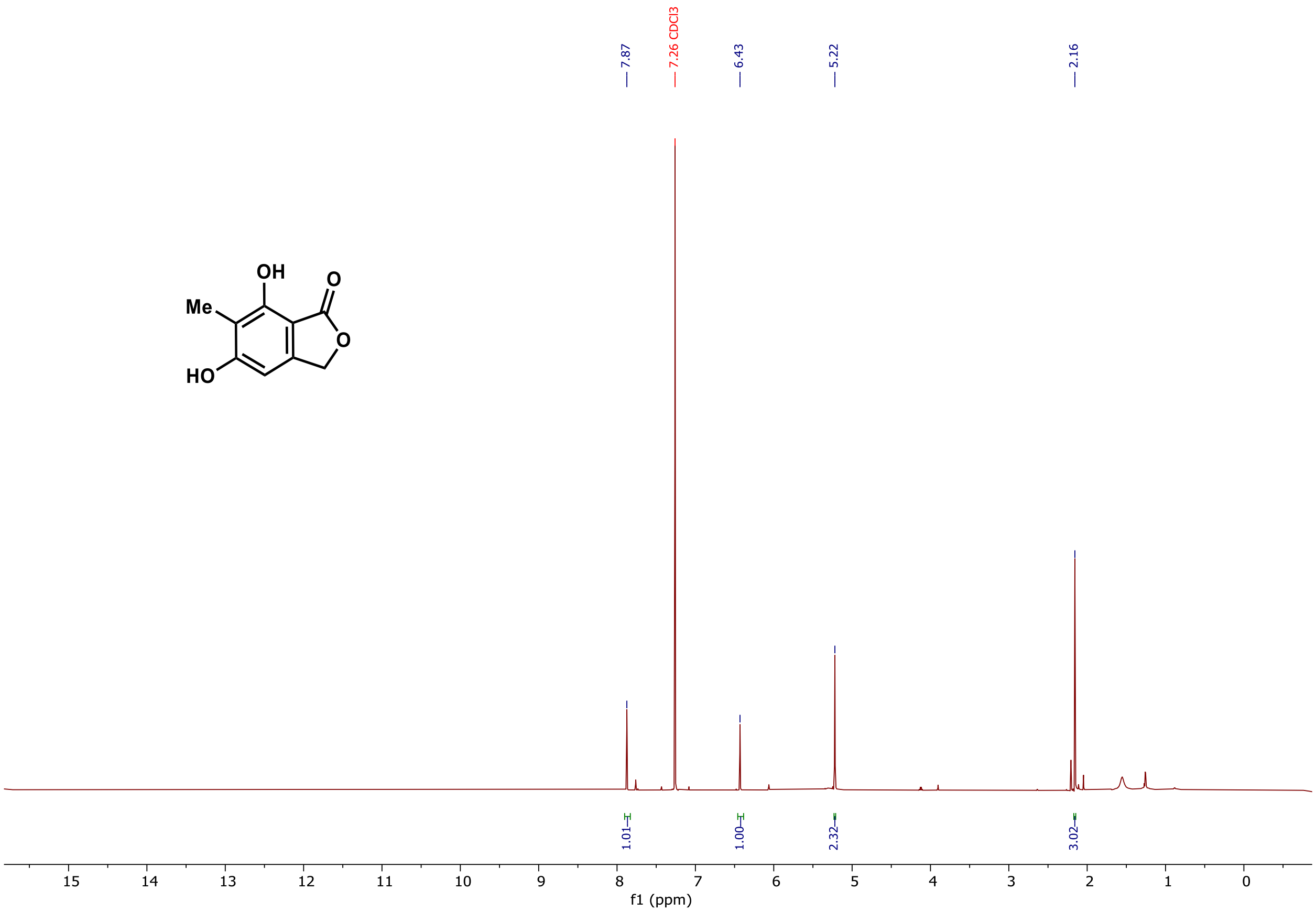

^{13}C-NMR (151 MHz, $CDCl_3$) of 5,7-dihydroxy-6-methylisobenzofuran-1(3H)-one (S29):

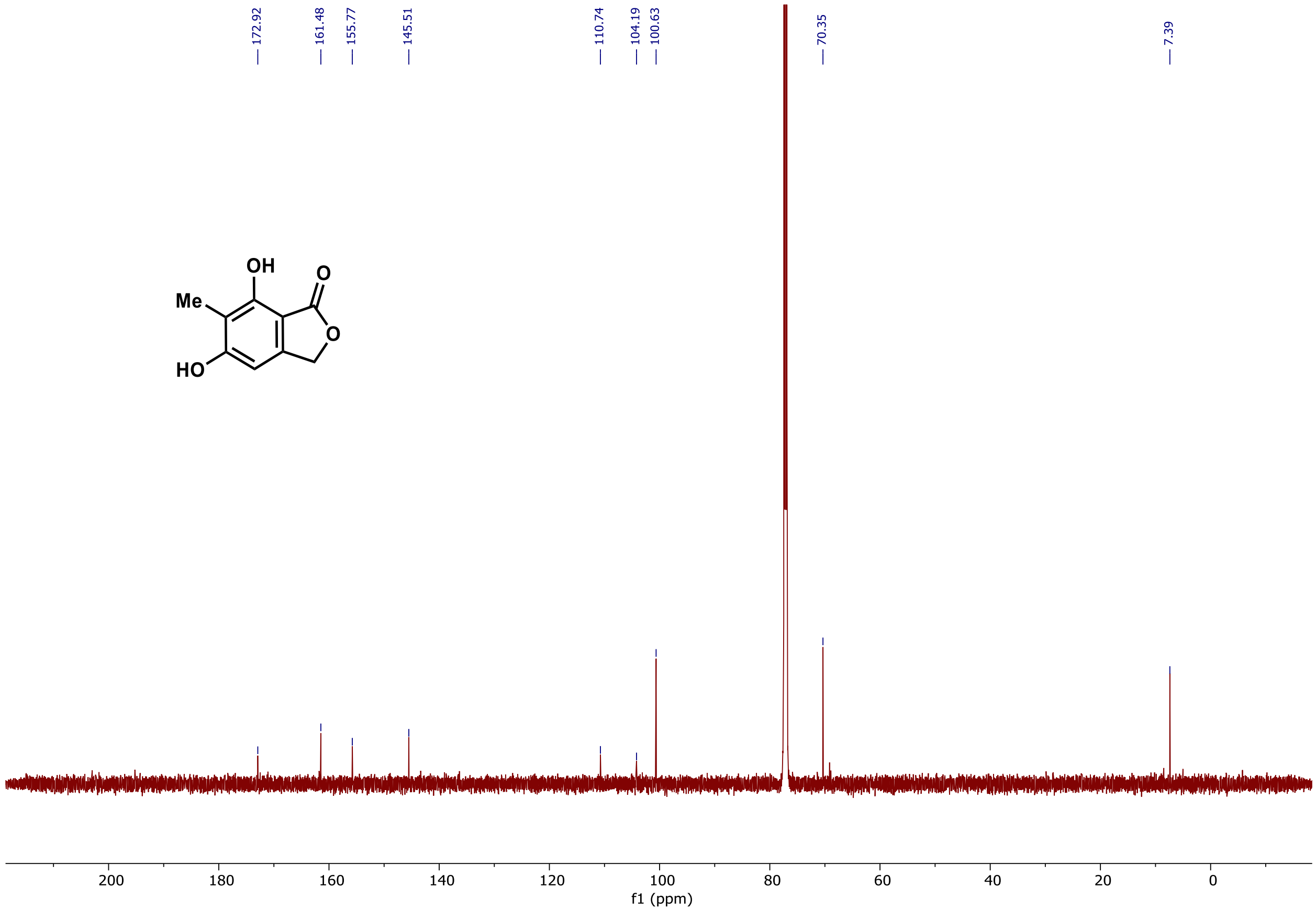

^{1}H-NMR (599 MHz, $CDCl_3$) of 5-((*tert*-butyldimethylsilyl)oxy)-7-hydroxy-6-methylisobenzofuran-1(3H)-one (S30):

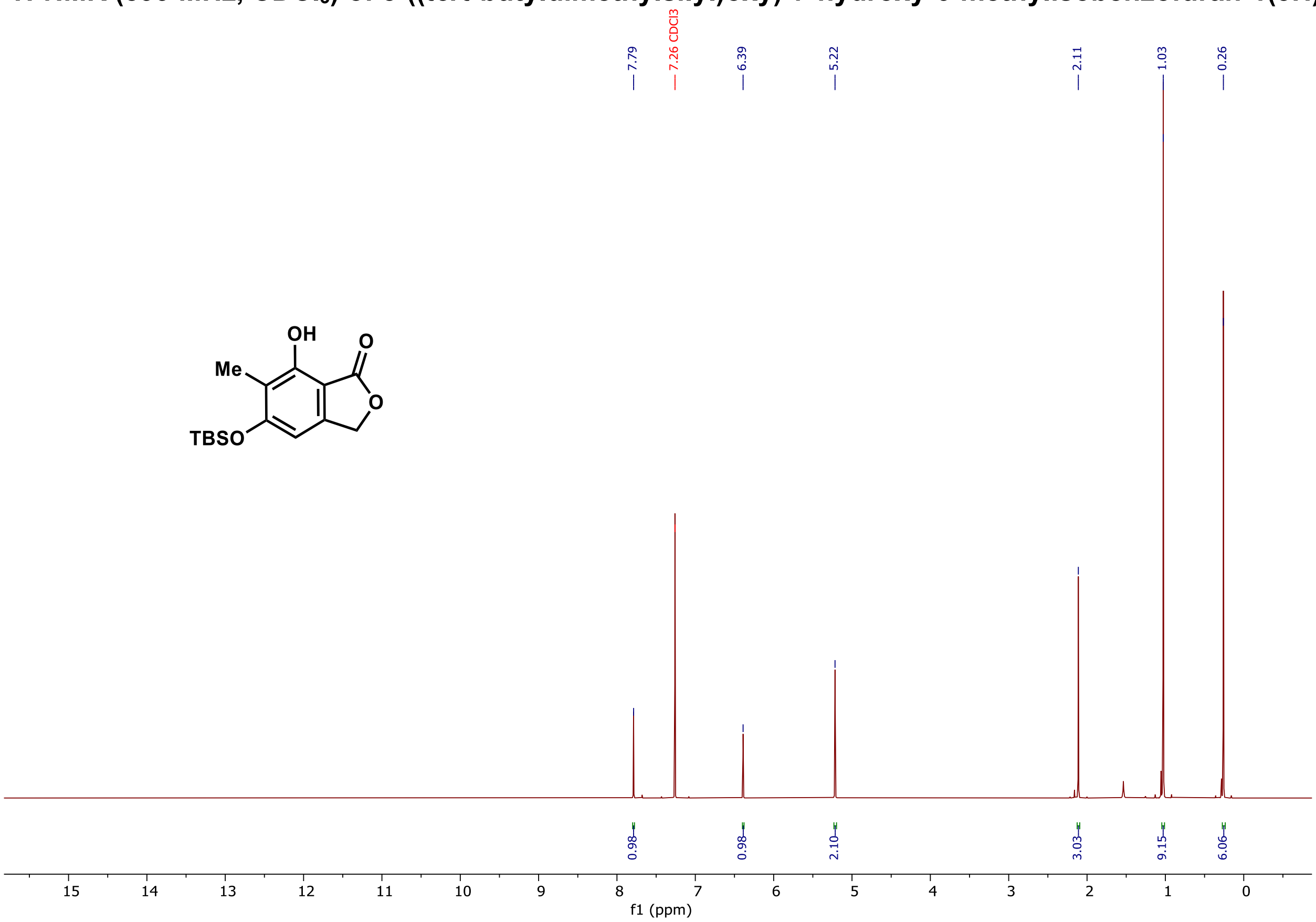

^{13}C-NMR (151 MHz, $CDCl_3$) of 5-((*tert*-butyldimethylsilyl)oxy)-7-hydroxy-6-methylisobenzofuran-1(3H)-one (S30):

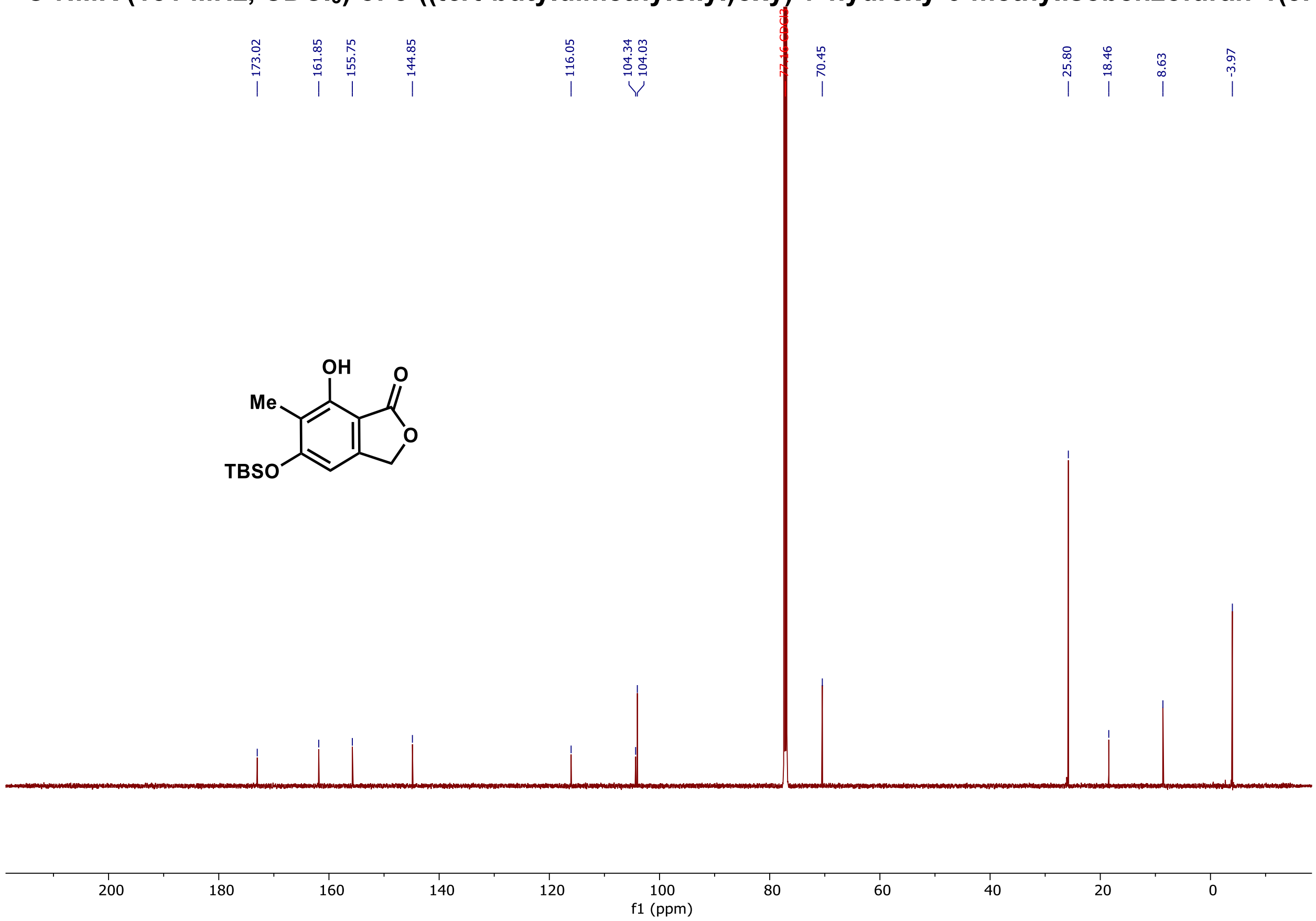

^{1}H-NMR (599 MHz, $CDCl_3$) of 5-((*tert*-butyldimethylsilyl)oxy)-7-methoxy-6-methylisobenzofuran-1(3H)-one (S31):

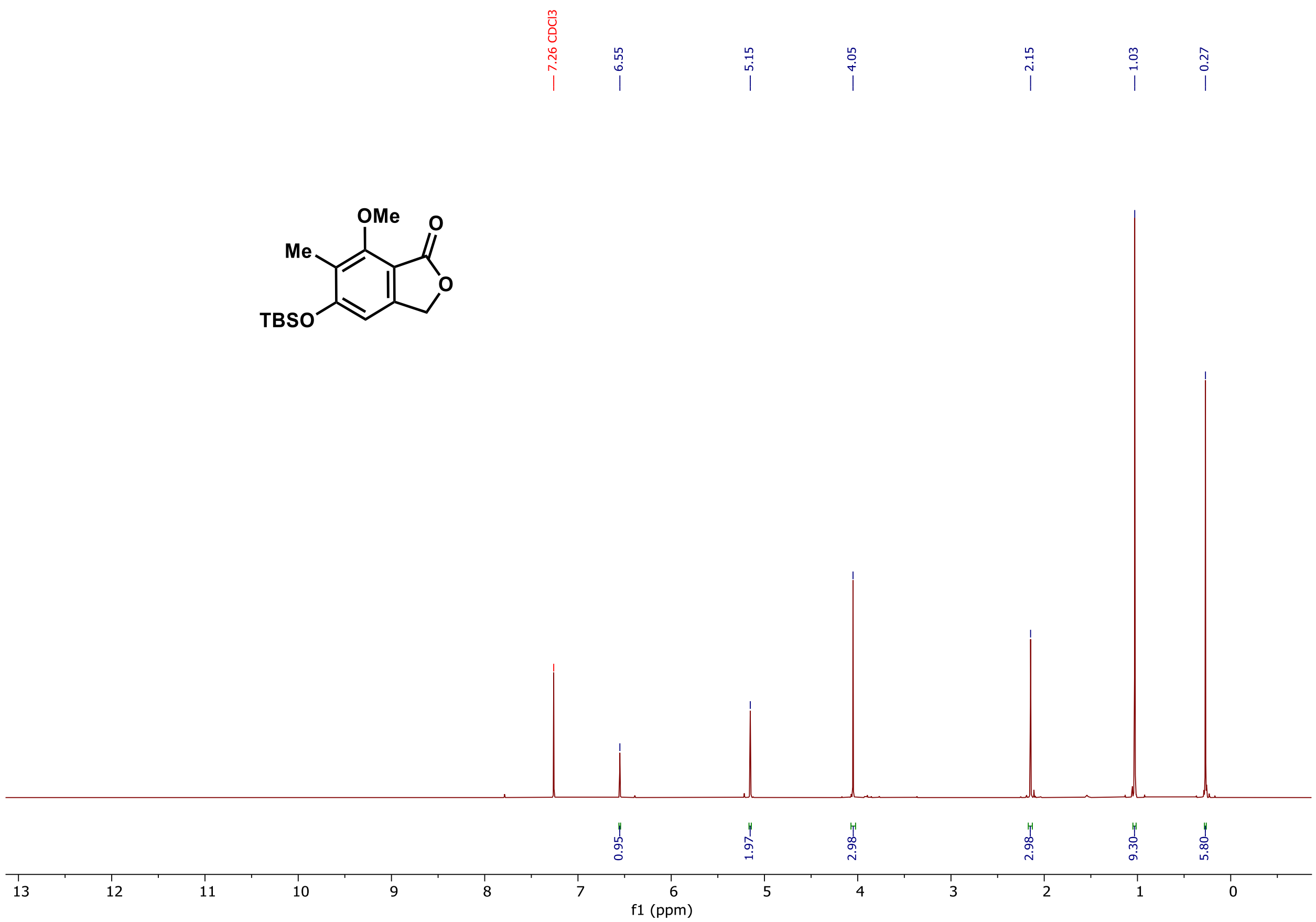

^{13}C-NMR (151 MHz, $CDCl_3$) of 5-((*tert*-butyldimethylsilyl)oxy)-7-methoxy-6-methylisobenzofuran-1(3H)-one (S31):

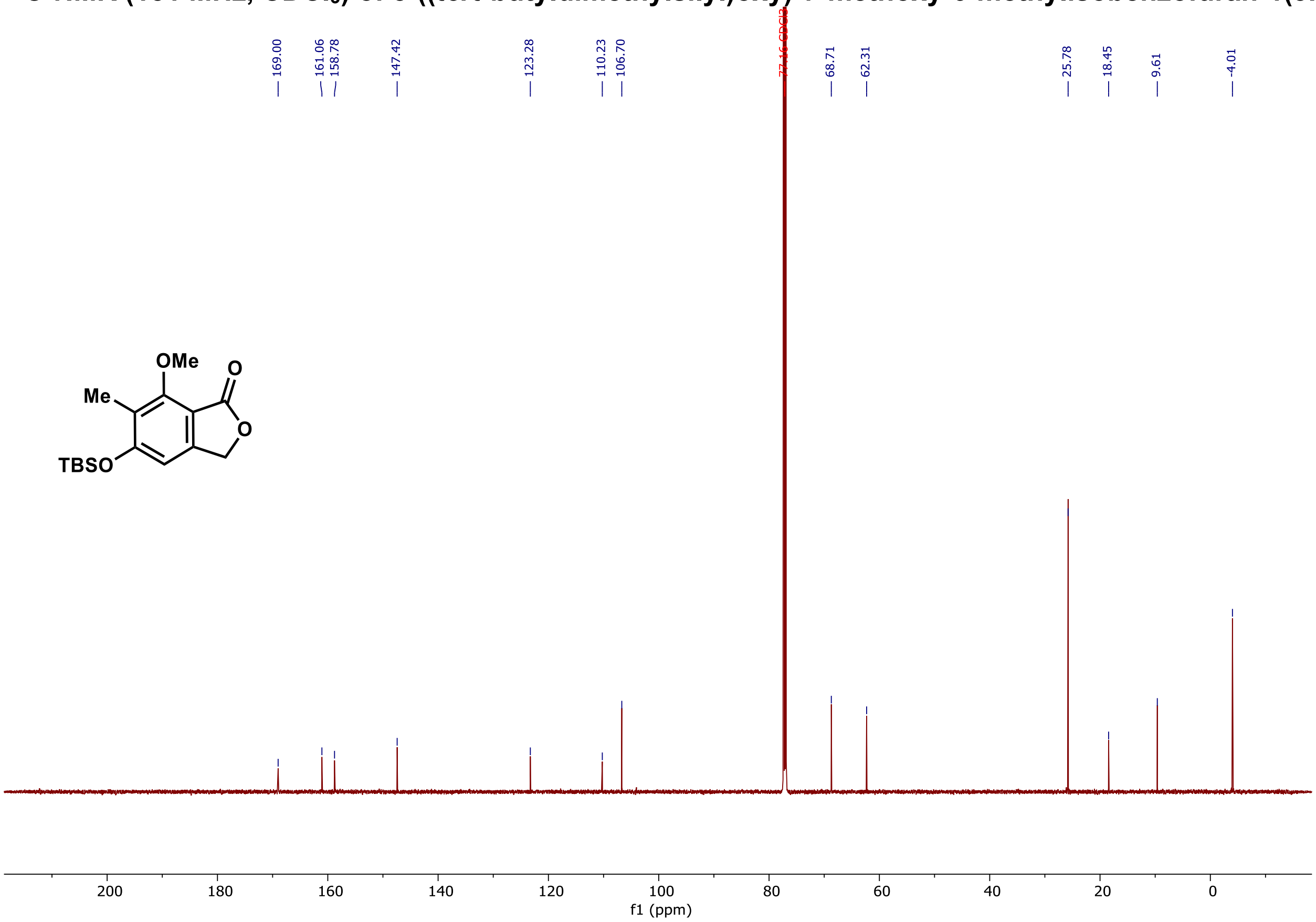

^{1}H-NMR (599 MHz, d_6-acetone) of 5-hydroxy-7-methoxy-6-methylisobenzofuran-1(3H)-one (10):

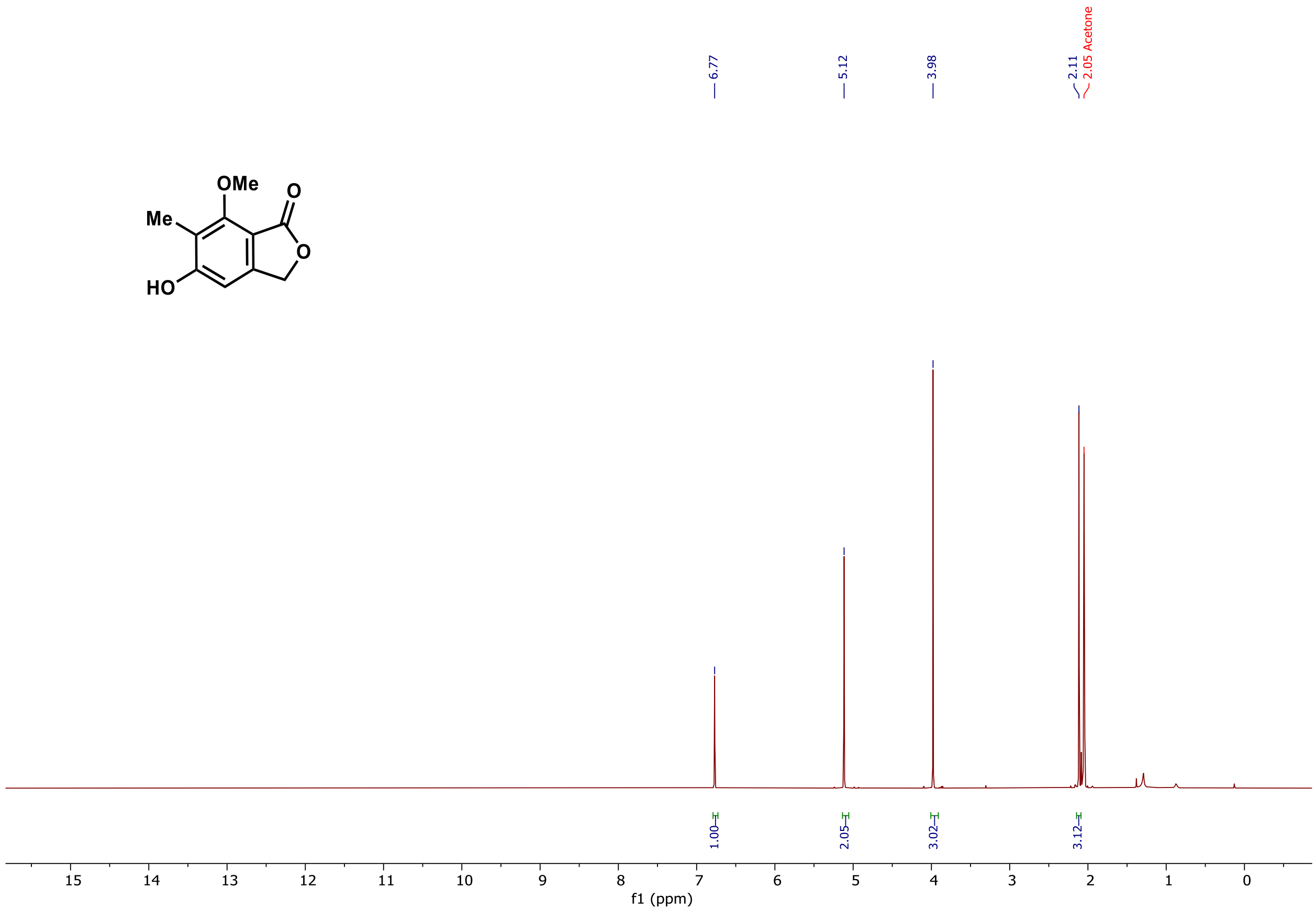

^{13}C-NMR (151 MHz, d_6-acetone) of 5-hydroxy-7-methoxy-6-methylisobenzofuran-1(3H)-one (10):

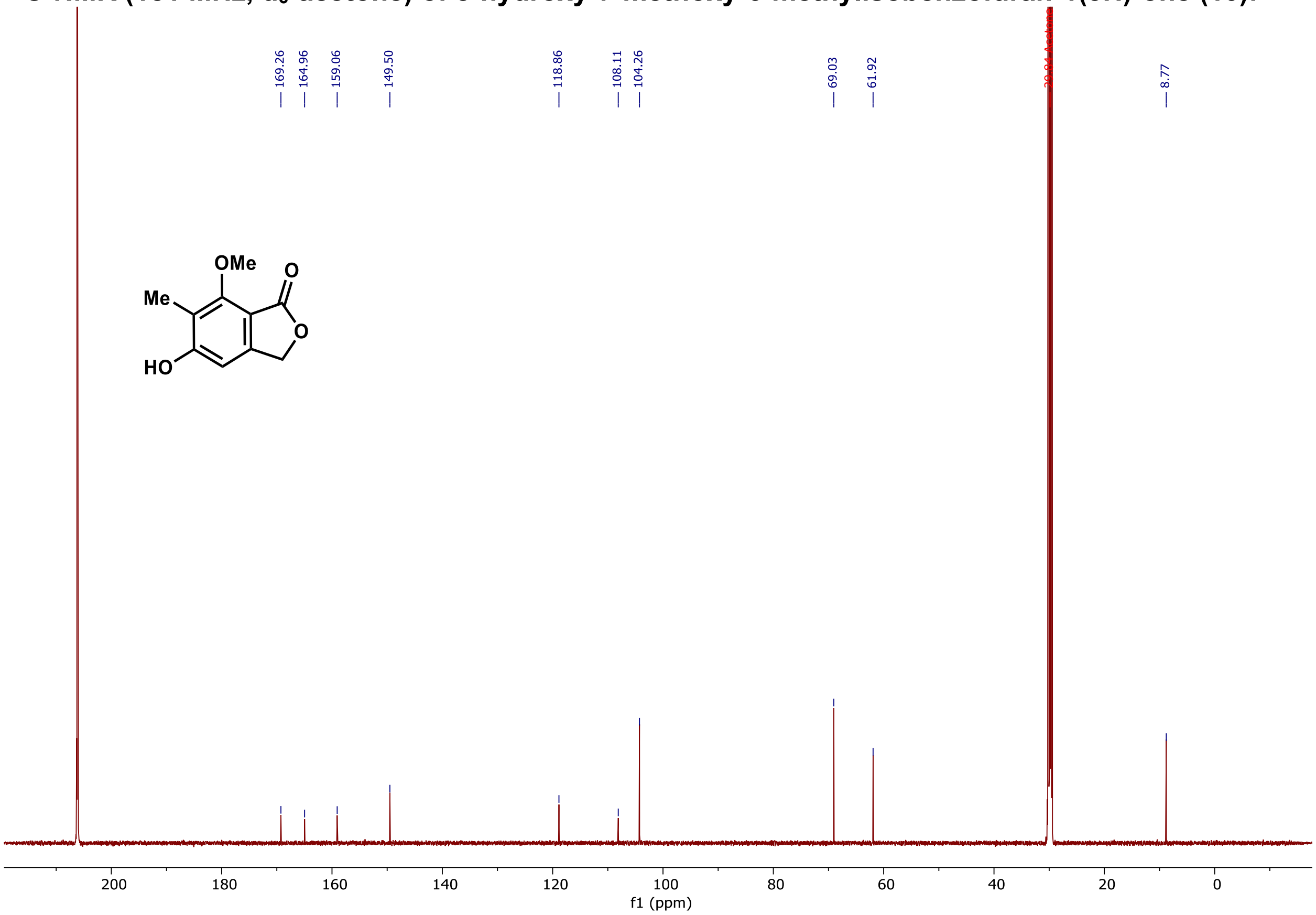

^{1}H-NMR (599 MHz, d_6-acetone) of 5-hydroxy-6-(hydroxymethyl)-7-methoxyisobenzofuran-1(3H)-one (15)

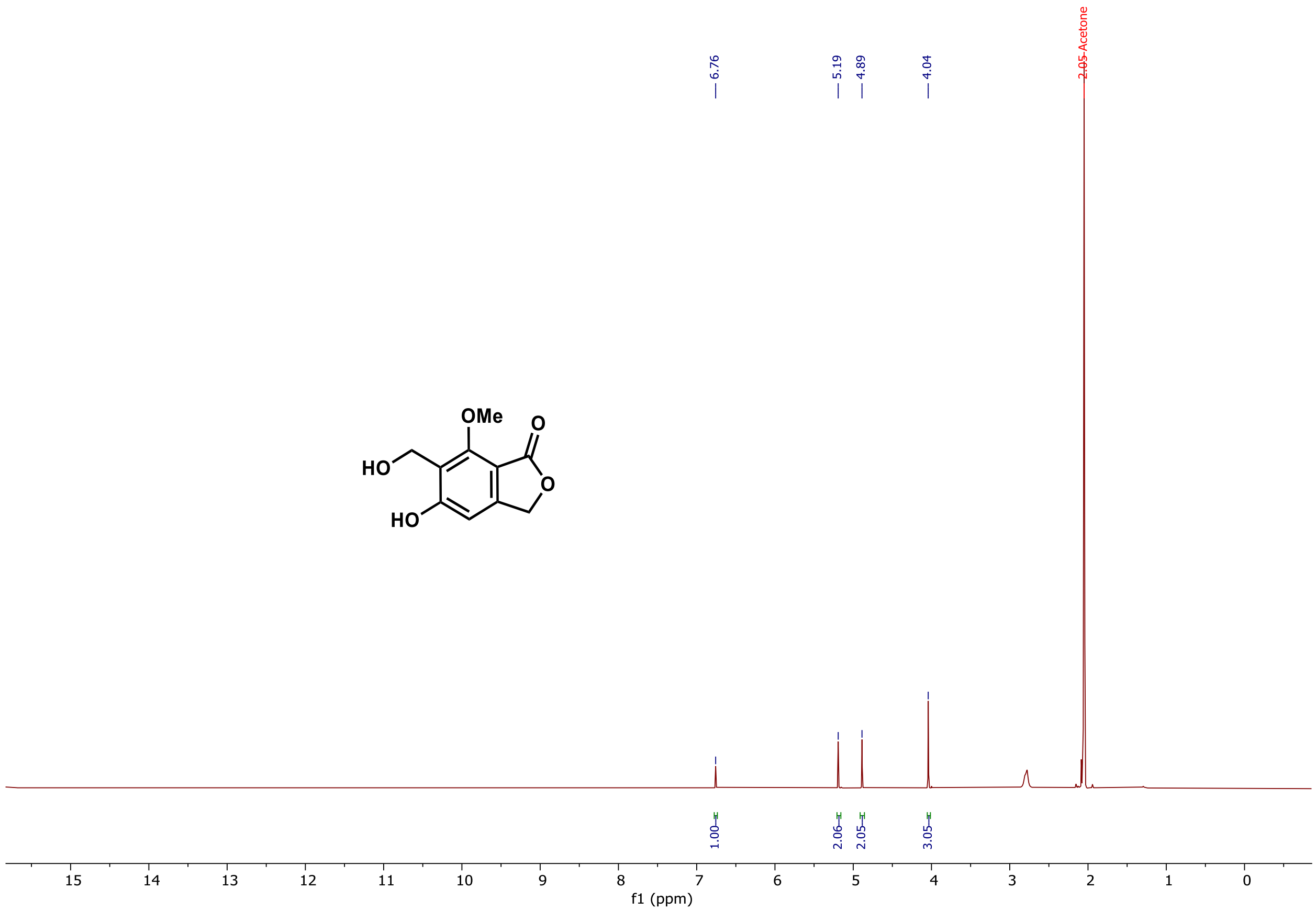

^{13}C-NMR (151 MHz, d_6-acetone) of 5-hydroxy-6-(hydroxymethyl)-7-methoxyisobenzofuran-1(3H)-one (15):

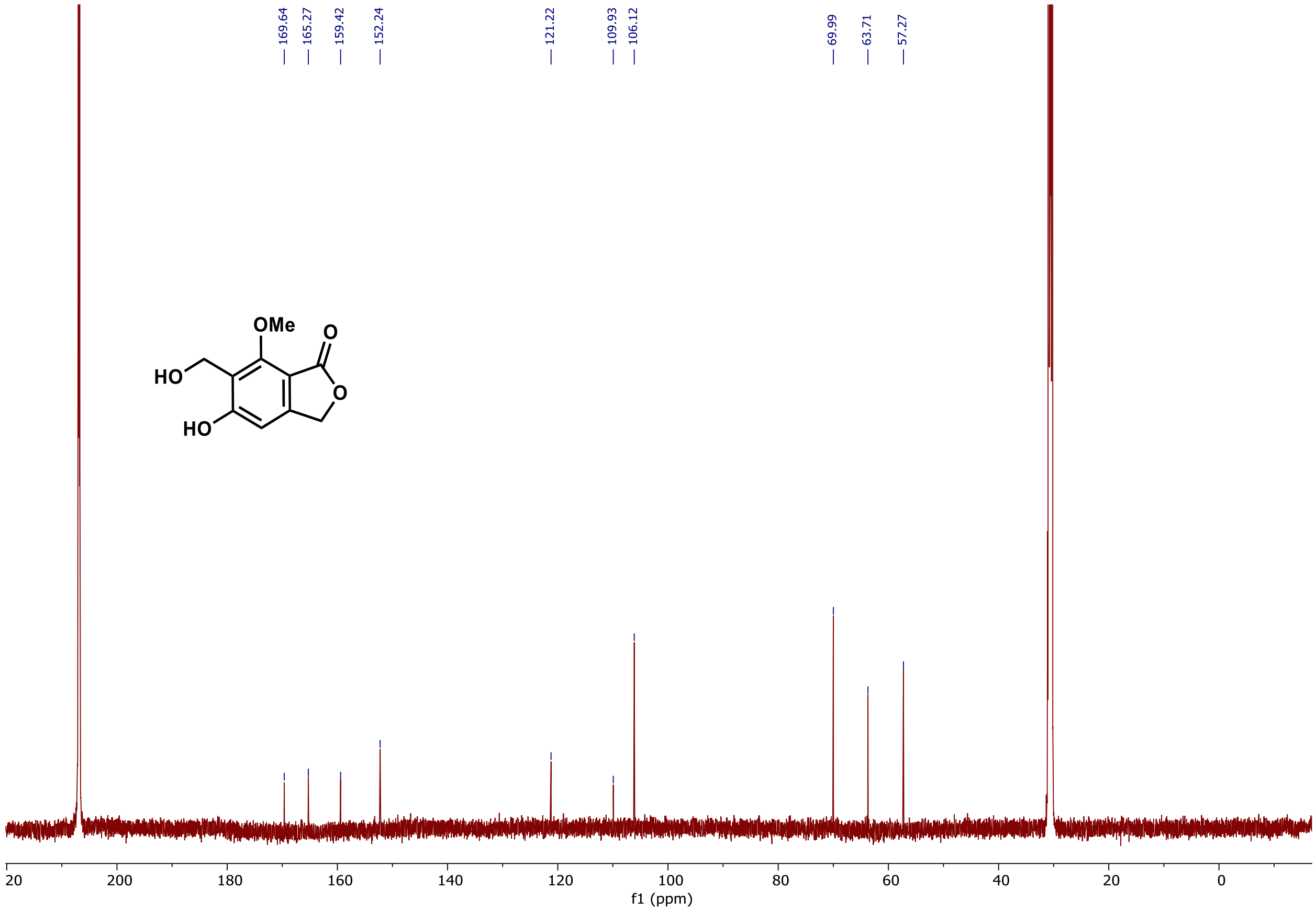

^{1}H-NMR (599 MHz, d_3-acetonitrile) of trifluoro((1*E*,5*E*)-2,6,10-trimethylundeca-1,5,9-trien-1-yl)-λ^4-borane, potassium salt (25):

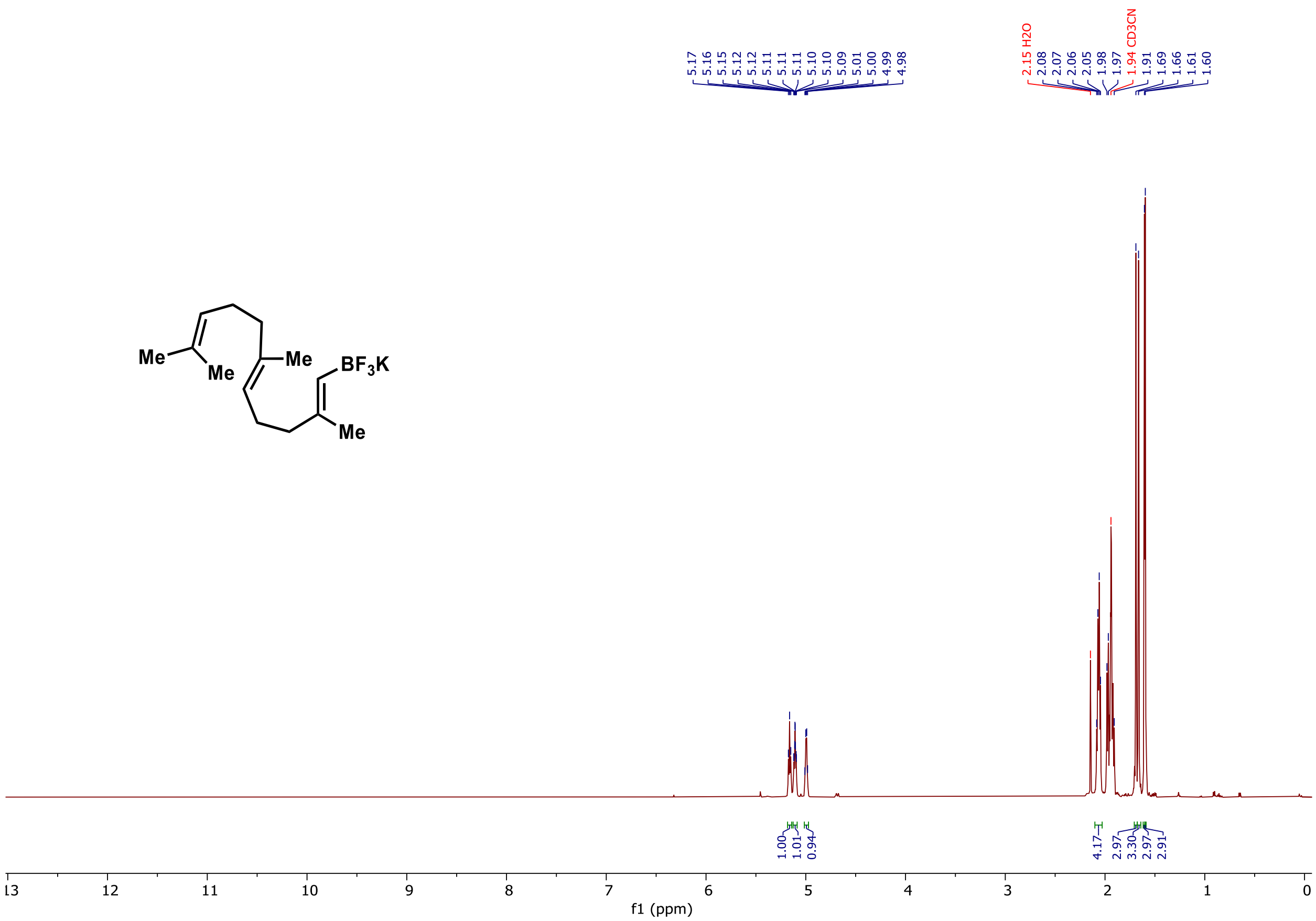

^{1}H-NMR (599 MHz, d$_3$-acetonitrile) of trifluoro((1*E*,5*E*)-2,6,10-trimethylundeca-1,5,9-trien-1-yl)-λ^4-borane, potassium salt (25):

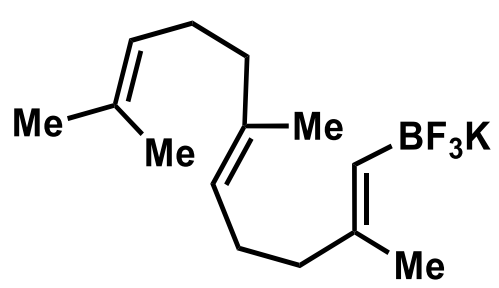

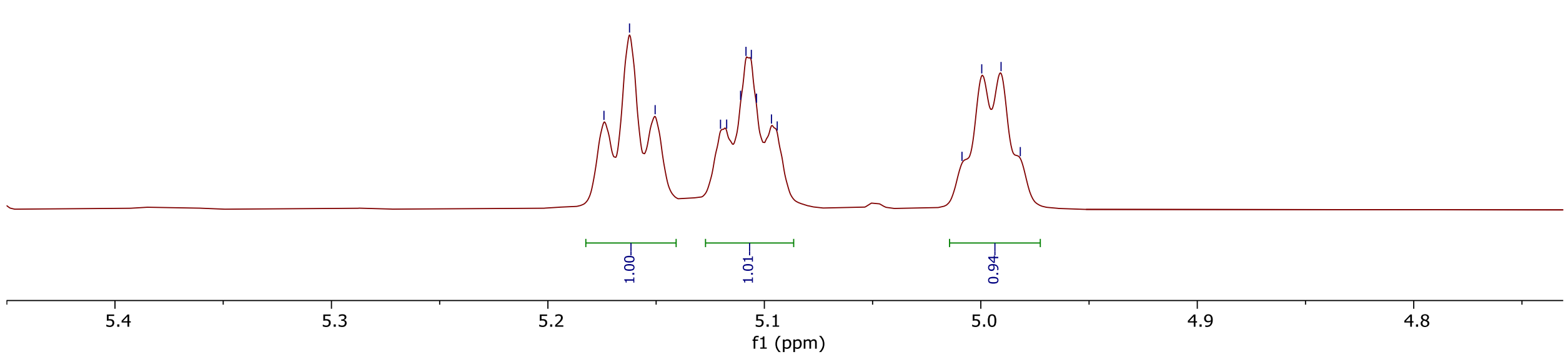

^{1}H-NMR (599 MHz, d_3-acetonitrile) of trifluoro((1*E*,5*E*)-2,6,10-trimethylundeca-1,5,9-trien-1-yl)-λ^4-borane, potassium salt (25):

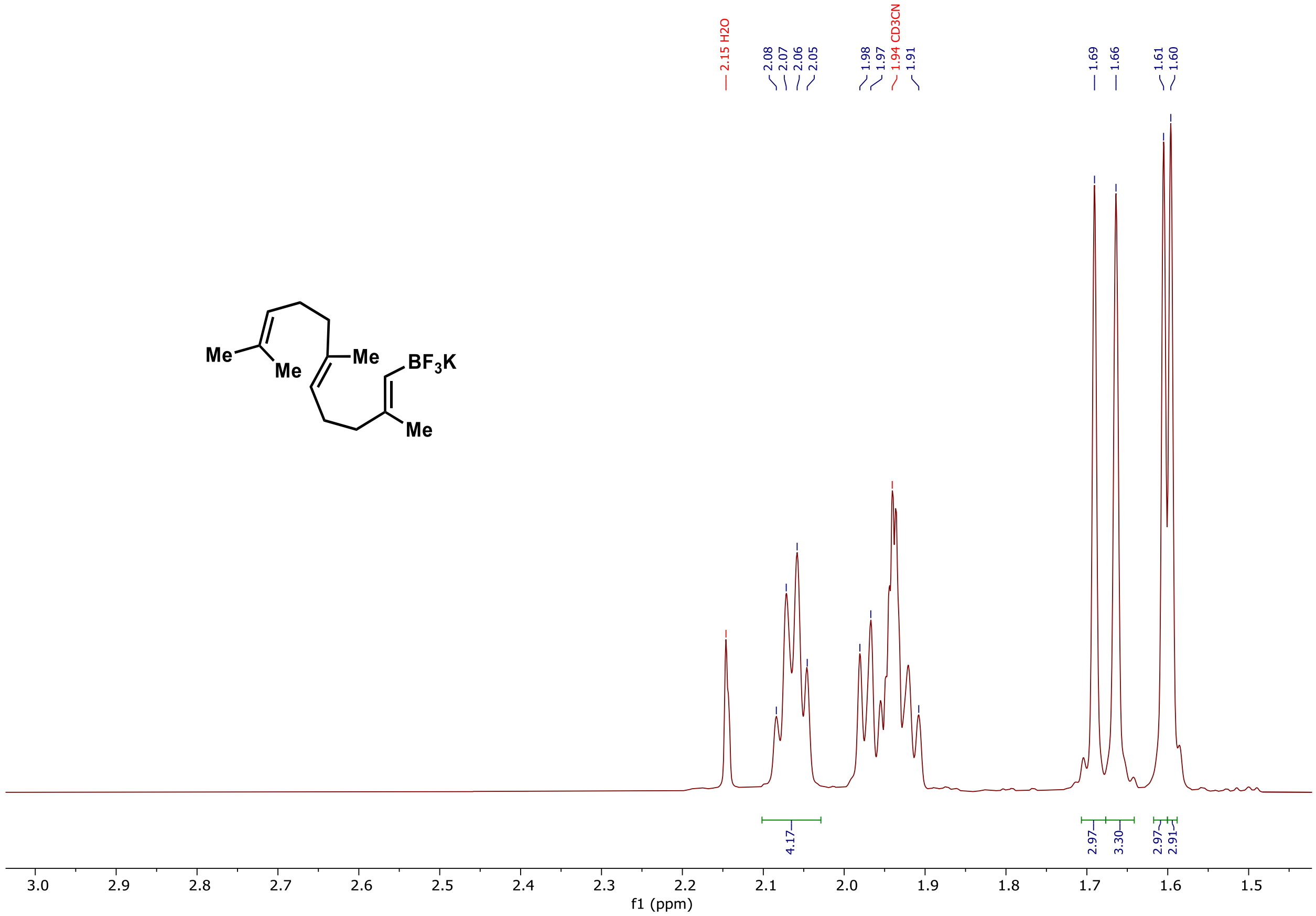

^{13}C-NMR (151 MHz, d$_3$-acetonitrile) of trifluoro((1*E*,5*E*)-2,6,10-trimethylundeca-1,5,9-trien-1-yl)-λ^4-borane, potassium salt (25):

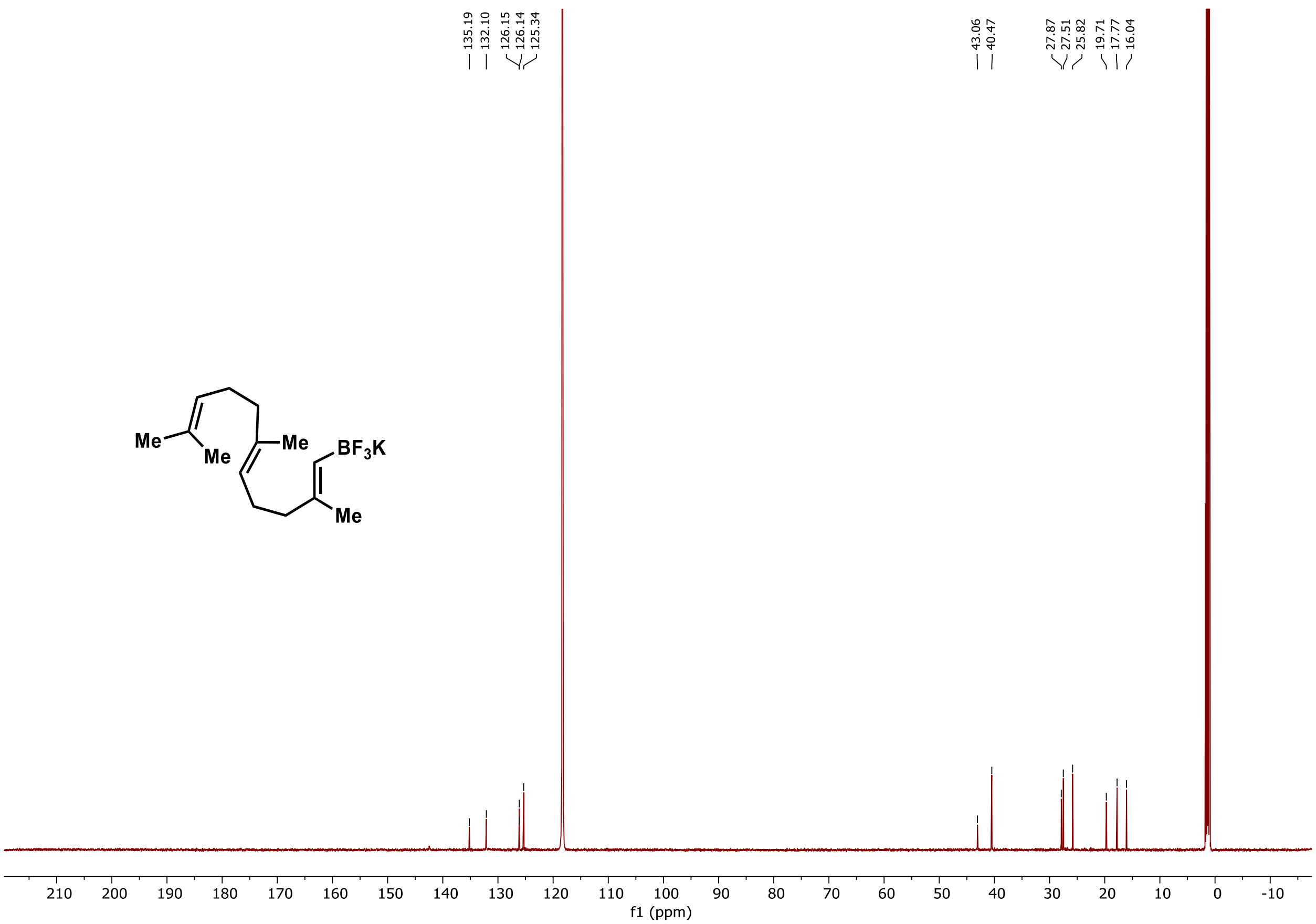

^{11}B-NMR (192 MHz, d_3-acetonitrile) of trifluoro((1*E*,5*E*)-2,6,10-trimethylundeca-1,5,9-trien-1-yl)-λ^4-borane, potassium salt (25):

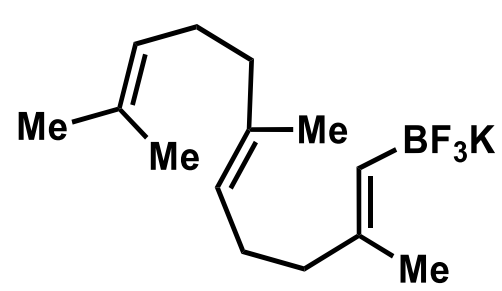

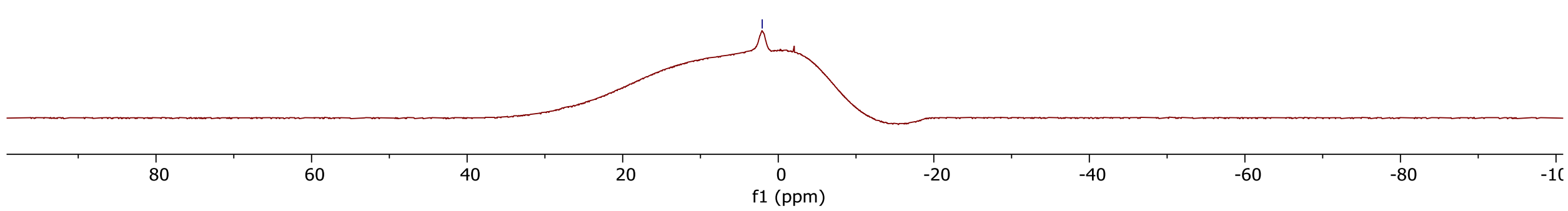

^{19}F-NMR (563 MHz, d$_3$-acetonitrile) of trifluoro((1*E*,5*E*)-2,6,10-trimethylundeca-1,5,9-trien-1-yl)-λ^4-borane, potassium salt (25):

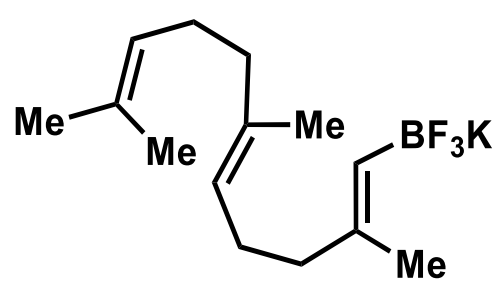

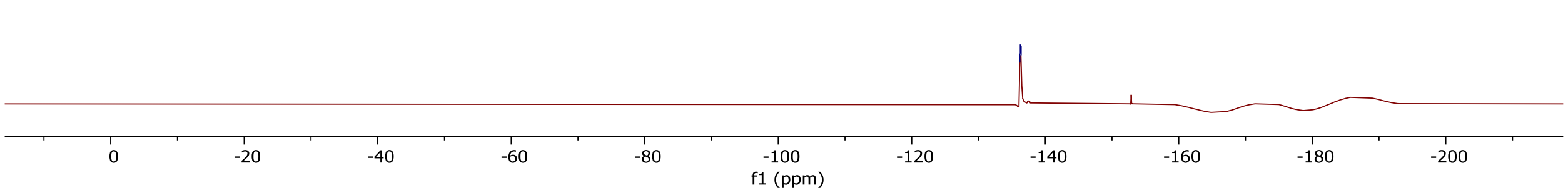

^{1}H-NMR (599 MHz, $CDCl_3$) of 5-hydroxy-7-methoxy-6-((2*E*,6*E*)-3,7,11-trimethyldodeca-2,6,10-trien-1-yl)isobenzofuran-1(3H)-one (26):

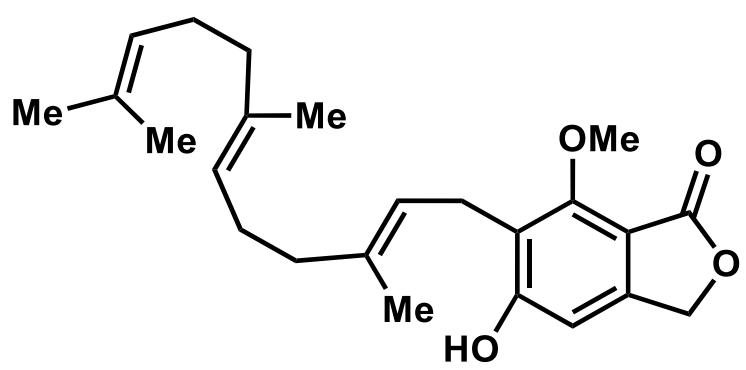

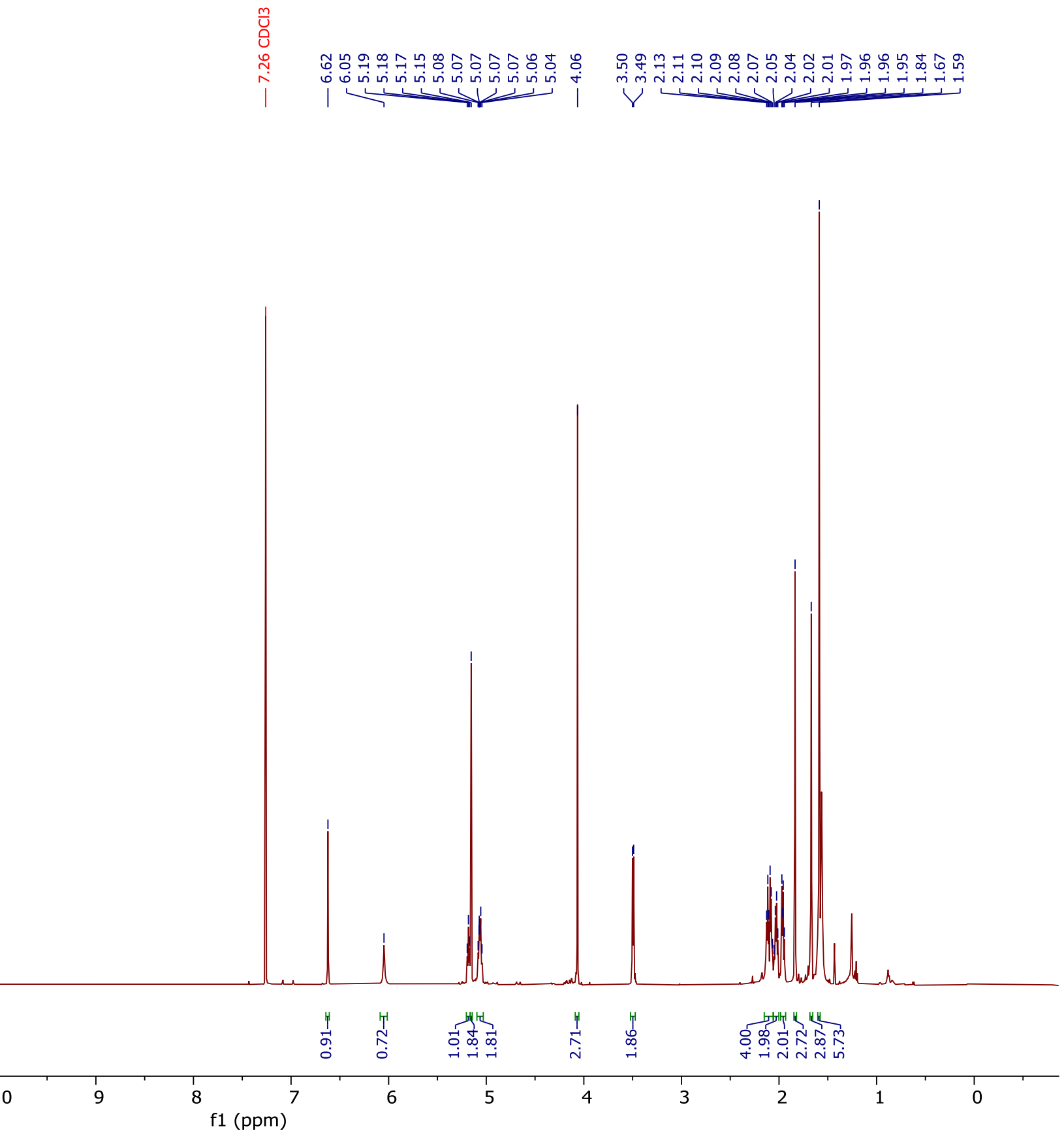

^{1}H-NMR (599 MHz, $CDCl_3$) of 5-hydroxy-7-methoxy-6-((2*E*,6*E*)-3,7,11-trimethyldodeca-2,6,10-trien-1-yl)isobenzofuran-1(3H)-one (26):

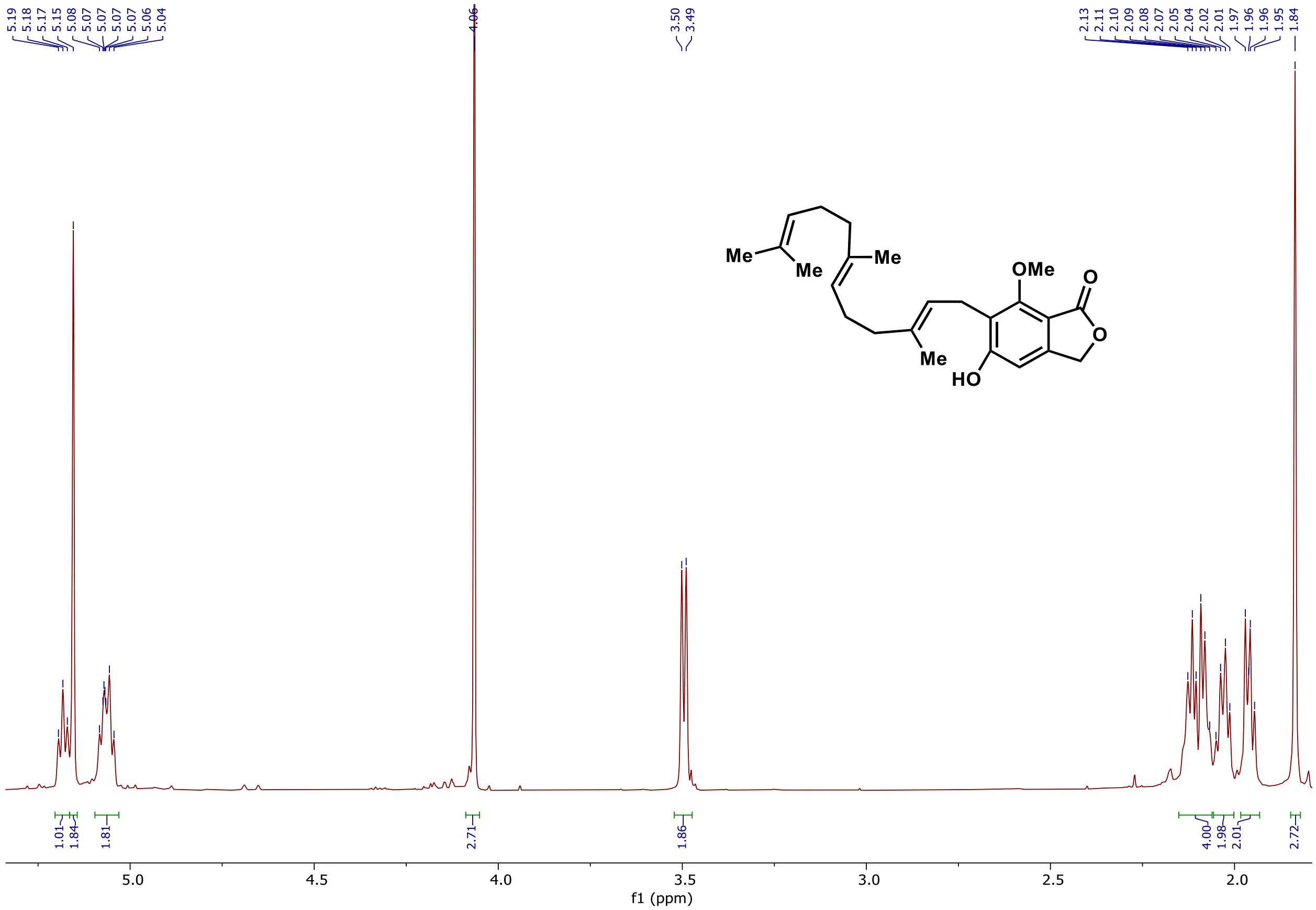

^{13}C-NMR (151 MHz, $CDCl_3$) of 5-hydroxy-7-methoxy-6-((2*E*,6*E*)-3,7,11-trimethyldodeca-2,6,10-trien-1-yl)isobenzofuran-1(3H)-one (26):

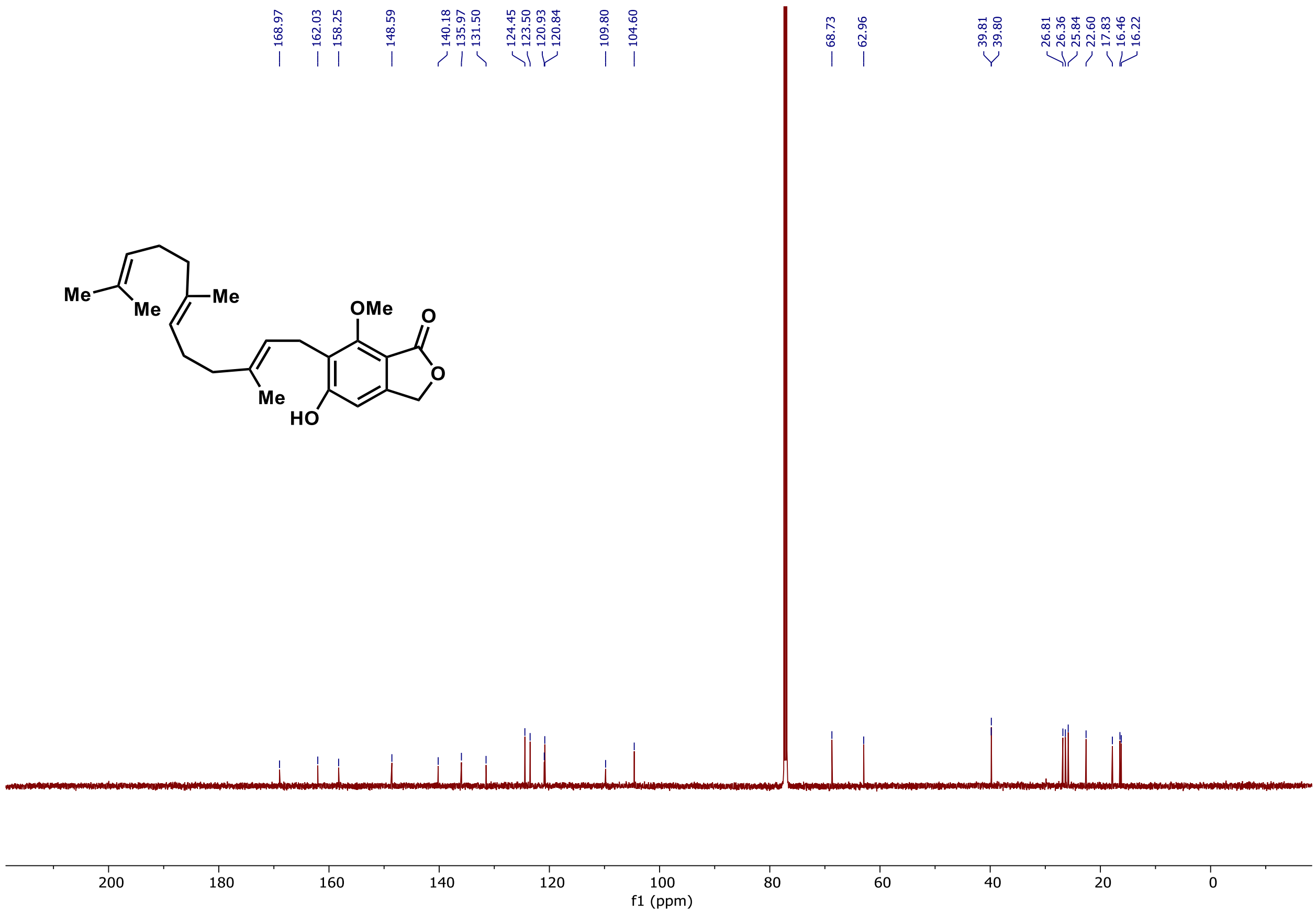

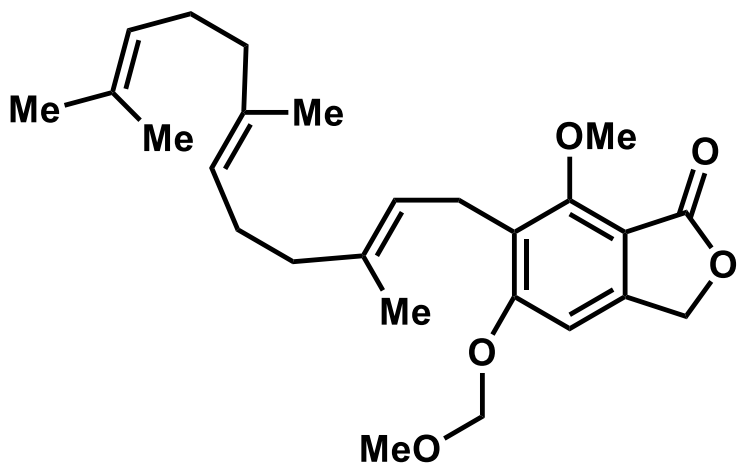

^{1}H-NMR (599 MHz, $CDCl_3$) 7-methoxy-5-(methoxymethoxy)-6-((2*E*,6*E*)-3,7,11-trimethyldodeca-2,6,10-trien-1-yl)isobenzofuran-1(3H)-one (27):

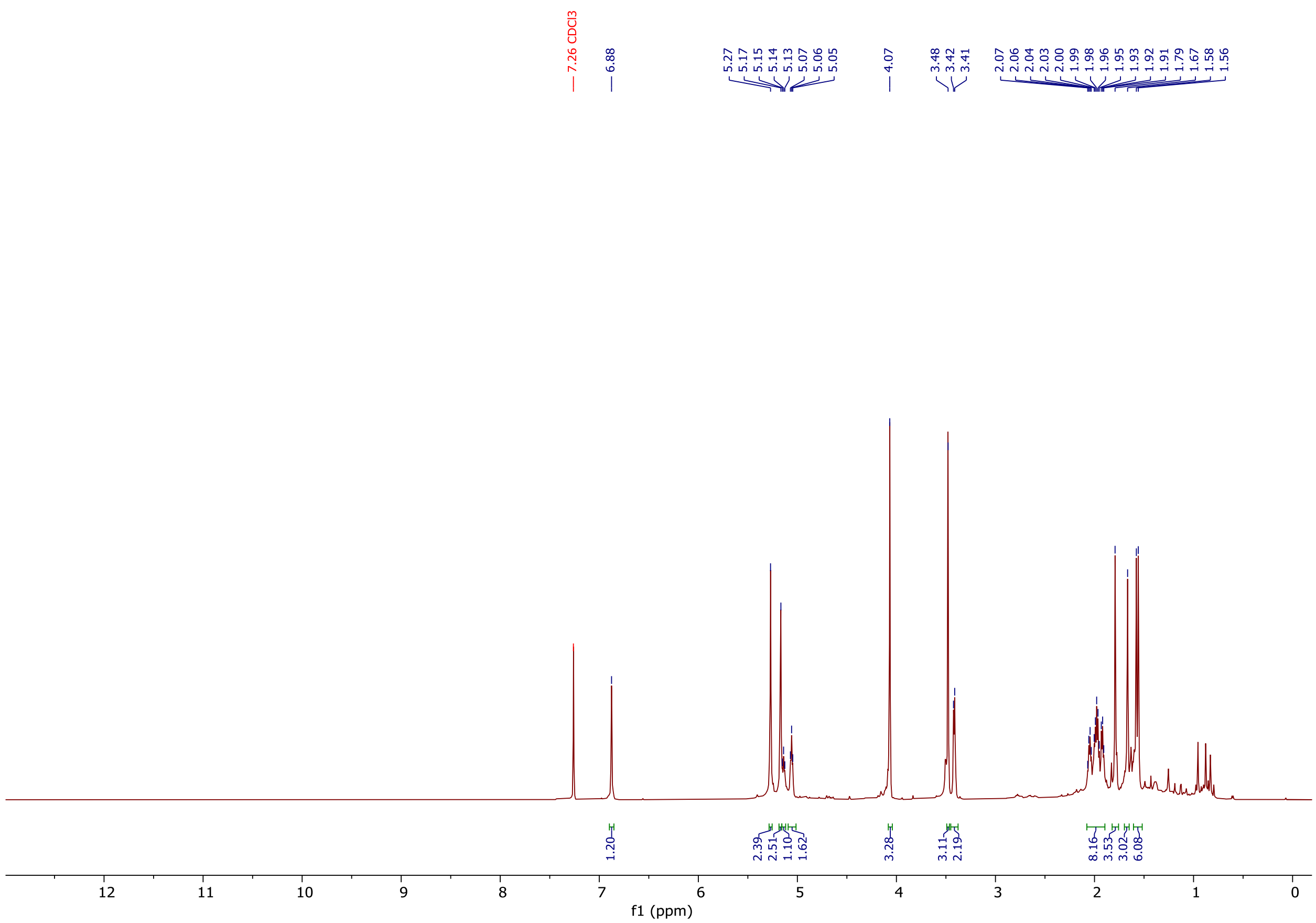

¹H-NMR (599 MHz, $CDCl_3$) 7-methoxy-5-(methoxymethoxy)-6-((2*E*,6*E*)-3,7,11-trimethyldodeca-2,6,10-trien-1-yl)isobenzofuran-1(3H)-one (27):

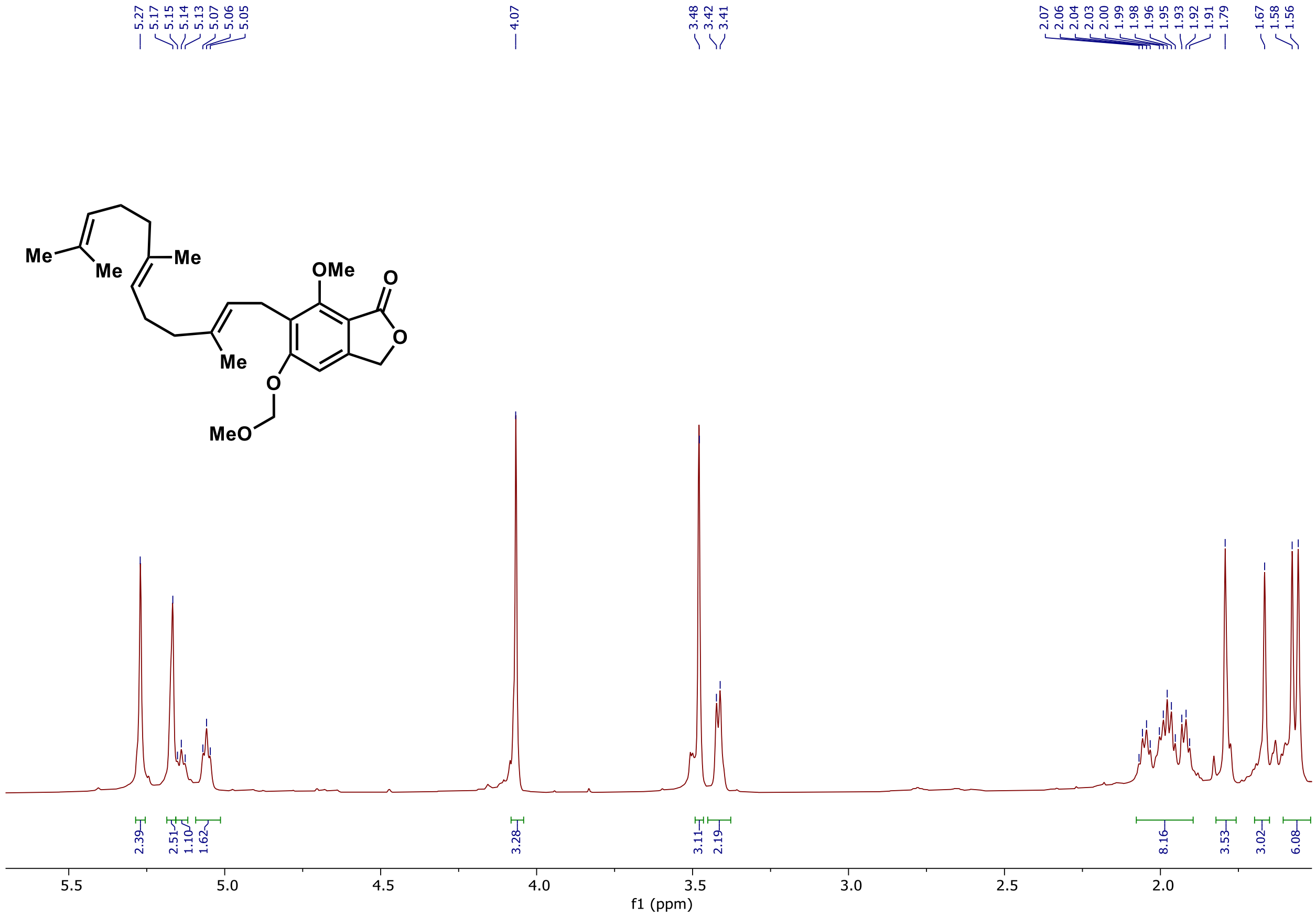

^{13}C-NMR (151 MHz, $CDCl_3$) 7-methoxy-5-(methoxymethoxy)-6-((2*E*,6*E*)-3,7,11-trimethyldodeca-2,6,10-trien-1-yl)isobenzofuran-1(3H)-one (27):

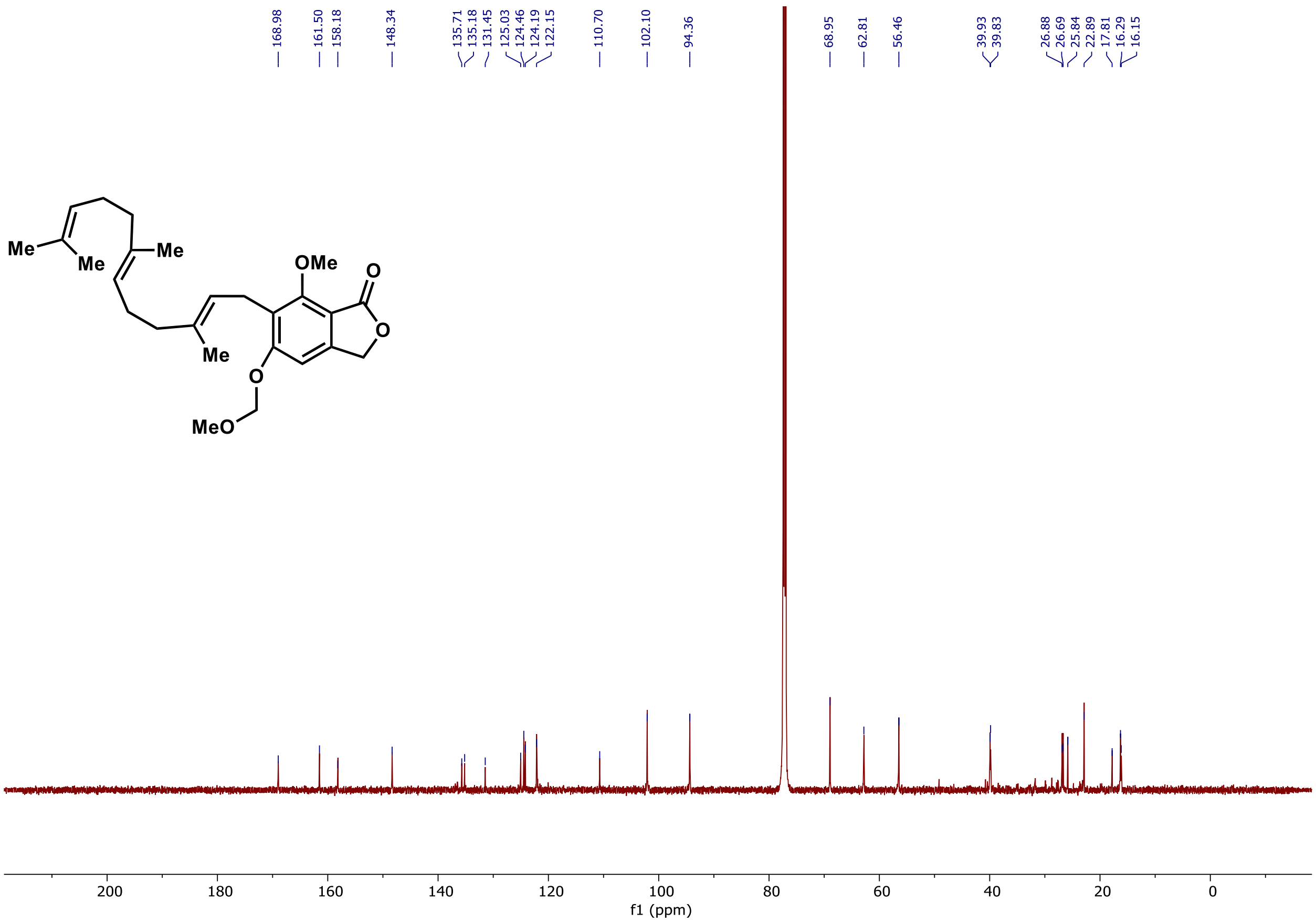

Supplement: Supplementary file 1 [file ANIE-65-e4567477-s001.pdf]
